# Cardiovascular safety of Janus kinase inhibitors in inflammatory bowel disease: a systematic review and network meta-analysis

**DOI:** 10.1080/07853890.2025.2455536

**Published:** 2025-01-21

**Authors:** Huibin Yang, Ting An, Yuxuan Zhao, Xiaojing Shi, Bangmao Wang, Qingyu Zhang

**Affiliations:** Department of Gastroenterology and Hepatology, Tianjin Medical University General Hospital, Tianjin, China

**Keywords:** Inflammatory bowel disease, Janus kinase inhibitor, major adverse cardiovascular events, network meta-analysis, venous thromboembolism events

## Abstract

**Background and Objective:**

Janus kinase (JAK) inhibitors (JAKinibs) are effective for inflammatory bowel disease (IBD), but their cardiovascular safety is inconclusive. We aim to assess the cardiovascular risks associated with JAKinibs in IBD patients.

**Patients and Methods:**

Systematic searches of seven databases and ClinicalTrials.gov from inception to February 2024 were conducted. Outcomes included major adverse cardiovascular events (MACE), venous thromboembolism events (VTE) and cardiovascular events (CVE), which were separately evaluated based on whether or not the dose was considered. P-score was applied to rank interventions.

**Results:**

A total of 26 trials involving 10,537 IBD patients were included, and results showed no significantly increased risk of MACE, VTE and CVE was associated with JAKinibs. However, when the dose was considered, Tofacitinib 5 mg BID (versus placebo) showed a trend towards an increased risk of MACE [odds ratio (OR)=1.05, 95% confidence interval (CI): 0.23–4.82], as well as Upadacitinib 30 mg QD (versus placebo) showed a trend towards increased risks of VTE (OR=1.36, 95% CI: 0.23–8.03) and CVE (OR=1.08, 95% CI: 0.24–4.85), and ranked higher than placebo for the risk of VTE [P-score=0.766 (versus 0.722)]. Notably, Deucravacitinib ranked lowest for all cardiovascular risks, and significantly decreased the risks of VTE (OR=0.03, 95% CI: 0.00-0.87) and CVE (OR=0.03, 95% CI: 0.00-0.87) compared with placebo.

**Conclusions:**

Although a trend of increased cardiovascular risks was found considering dose, no significantly increased cardiovascular risk was associated with JAKinibs in IBD patients, and Deucravacitinib significantly decreased the risks of VTE and CVE.

## KEY MESSAGES

This is the first network meta-analysis specifically assessing the cardiovascular risks associated with JAKinibs in IBD patients, and findings demonstrated that no significantly increased cardiovascular risk was found and Deucravacitinib significantly decreased the cardiovascular risks.

## GRAPHICAL ABSTRACT

**Figure:**
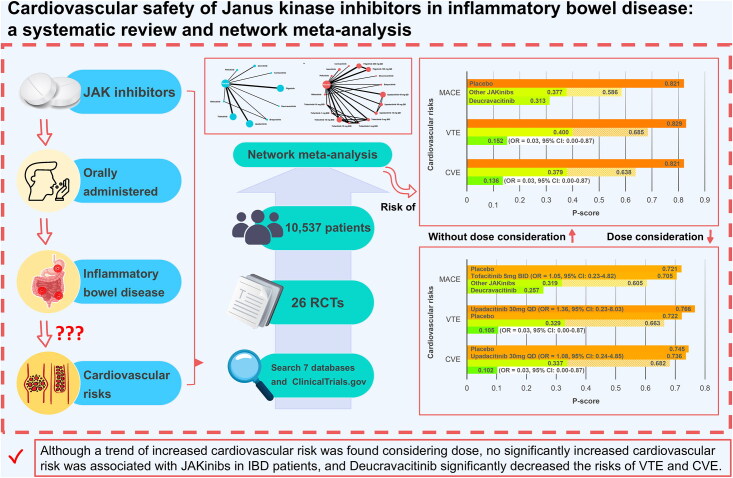

## Introduction

Inflammatory bowel disease (IBD) is a chronic relapsing and remitting disease, affecting the gastrointestinal tract and categorized as ulcerative colitis (UC) and Crohn’s disease (CD). Recurrent abdominal pain and diarrhea may last for many years and even exist throughout life, necessitating effective and long-term treatment [1]. Janus kinase inhibitors (JAKinibs) are an emerging treatment option, and have been proven to be associated with favorable clinical outcomes in IBD patients [2].

However, with the data becoming available from clinical trials and practices, the cardiovascular safety of JAKinibs has been on the spotlight [3]. Although some studies have demonstrated that no significantly increased risk of major adverse cardiovascular events (MACE) and venous thromboembolism events (VTE) was found in patients receiving JAKinibs [4,5], a growing body of evidence indicated that JAKinibs were associated with increased risks of MACE and VTE [6–9]. Among them, a study conducted in patients with rheumatoid arthritis (RA) showed an significantly increased risk of VTE [Hazard Ratio (HR) = 3.52, 95% confidence interval (CI): 1.74–7.12], and a trend towards an increased risk of MACE (HR = 1.33, 95% CI: 0.91–1.94) were associated with the treatment of JAKinibs [9]. In view of these safety concerns, both European Medicines Agency (EMA) [10] and the United States Food and Drug Administration (FDA) [11] released some warnings and added the black box warnings on JAKinibs for related cardiovascular risks.

The mechanisms underlying the cardiovascular events associated with JAKinibs remain unclear. Some studies indicated that these events may be attributable to the low selectivity of JAKinibs because of the pan-JAK blockade [12,13]. The Janus kinase-signal transducer and activator of transcription (JAK-STAT) signaling pathway plays a crucial role in mediating more than 50 signal molecules, including cytokines, hormones and growth factors [14]. Therefore, the broad impact of JAKinibs on this pathway may disrupt the balance between the prothrombotic effect and antithrombotic effect, potentially leading to thrombosis and cardiovascular events [15]. Certain evidence supports this hypothesis to some extent. Deucravacitinib, a recently approved JAKinib, specific inhibits tyrosine kinase 2 (a member of JAKs) through allosteric inhibition rather than competitive inhibition, and to data, no significant cardiovascular events have been documented [16–18]. Nevertheless, given the intricate mechanism of the JAK-STAT signaling pathway and the limited data available regarding post-marketing reports on the clinical use of Deucravacitinib, more clinical data are still needed to explore the relation between JAKinibs and cardiovascular events.

Considering there remain knowledge gaps about the cardiovascular safety in IBD patients treated with JAKinibs [19], and there are potential values to clarify the cardiovascular risks associated with JAKinibs to guide decision-making for clinicians who are considering this therapy, we gathered multiple trials data and carried out a network meta-analysis (NMA) of randomized controlled trials (RCTs) to explore whether JAKinibs increased the risks of cardiovascular adverse events [MACE, VTE and cardiovascular events (CVE)] in IBD patients.

## Patients and methods

### Protocol and registration

This systematic review and network meta-analysis assessed the risk of MACE, VTE and CVE in IBD patients treated with JAKinibs. The protocol was registered in the PROSPERO (International Prospective Register of Systematic Reviews) with the register name “Risk of cardiovascular and venous thromboembolic events with JAK inhibitors in inflammatory bowel diseases: a systematic review and network meta-analysis” and registration number CRD42024524803, as well as reported according to the PRISMA (Preferred Reporting Items for Systematic reviews and Meta-Analyses) 2020 statement [20,21]. The PRISMA checklist was shown in Supplementary Table 1.

### Data source and search strategy

The systematic literature searches were independently performed by three researchers in seven electronic databases (PubMed, Embase, Ovid Medicine, Web of Science, Cochrane Library, ProQuest and Scopus) and ClinicalTrials.gov from their inception to February 2024. The details of search strategy were shown in Supplementary Table 2 and any discrepant issues were resolved through discussion.

### Eligibility criteria and study selection

Eligibility criteria were formulated according to PICOS (Population, Intervention, Comparison, Outcome and Study design): (1) Population: adults (age ≥ 18 years) patients with IBD; (2) Intervention: JAKinibs that were approved or under development without dose restrictions, whether they were given as monotherapy or in combination with other conventional drugs. The basic information of included JAKinibs was shown in Supplementary Table 3; (3) Comparison: placebo or other standard treatment; (4) Outcome: MACE (defined as myocardial infarction and ischemic stroke), VTE (defined as deep vein thrombosis, pulmonary embolism and unclassified or other vein thrombosis) and CVE (defined as the sum of MACE and VTE); (5) Study design: any phase II or III double-blind RCTs. Studies were excluded if they were: (1) duplicate literatures; (2) trails involving pregnant patients; (3) animal or *in vitro* experiments; (4) presented with insufficient data to analyze; (5) published in a language other than English; (6) trials without the control group; (7) reviews, meta-analyses, case reports, observational studies, comments, editorials or letters to the editor. Three researchers independently reviewed the titles and abstracts of all studies after the duplicate studies were removed by automation tools and manually method. Until a consensus was obtained, studies requiring full-text assessment for eligibility were continued to review independently. Any discrepant issues were resolved through discussion.

### Data extraction

Three researchers independently collected and collated the following data from eligible studies: trial data (first author’s name, study date, register number, sample size, study phase, intervention and study duration) and patient data (age, percentage of female, disease duration, outcome and IBD type). We reviewed the full text, supplementary materials and clinical registry data for sufficient data to analyze and preferentially abstracted the data from the full text and supplementary materials. If data in trails were available at many time points, we only extracted the latest data. Any discrepancies were resolved through discussion.

### Quality assessment

Three researchers independently assessed the risk of bias for all included RCTs using the version 2 of the Cochrane tool for assessing risk of bias in randomized trials (RoB 2) [22]. In the RoB 2, judgments contained five domains: (1) randomisation process; (2) deviations from intended interventions; (3) missing outcome data; (4) measurement of the outcome; (5) selection of the reported result. Risk-of-bias judgments were rated as “low risk”, “some concerns” or “high risk”. Any discrepancies were resolved through discussion.

### Outcome measure

The outcomes were cardiovascular adverse events (MACE, VTE and CVE) and the relations between their risks and JAKinibs were evaluated. Outcome measure was presented as odds ratio (OR) with their 95% CI and comparisons were performed among all interventions (JAKinibs and control) to clarify whether JAKinibs increased the risks of MACE, VTE and CVE in IBD patients, and to explore which JAKinib was associated with the highest risks of MACE, VTE and CVE. In addition, considering the varying effects of different doses for the same JAKinib, we separately analyzed outcomes based on whether or not the dose was considered: outcome analysis with dose consideration and outcome analysis without dose consideration (merging data on different doses of JAKinibs).

### Certainty of evidence

The certainty of evidence for each comparison was rated according to the GRADE (Grading of Recommendations, Assessment, Development and Evaluation) approach [23] and the following domains were assessed: (1) risk of bias; (2) inconsistency; (3) indirectness; (4) publication bias; (5) imprecision; (6) intransitivity; (7) incoherence. Certainty of evidence judgments were rated as “high”, “moderate”, “low” or “very low”.

### Statistical analysis

All statistical analyses were performed with the R (R version 4.3.3, R Foundation for Statistical Computing) using the “netmeta” package (v2.9.0) with a random effects model. When zero-count event was reported in one or more groups, a fixed continuity correction of 0.5 was applied to enable the effective calculation [24]. P-value < 0.05 was considered statistically significant. The detailed R codes were shown in Supplementary Table 4.

The network graphs, depicting interventions and direct comparisons, were formed using the “netgraph” function. The nodes represented interventions, and the size of the nodes represented the sample size included for each intervention. The lines connecting the nodes represented direct comparisons between interventions, and the thickness of the lines represented the number of RCTs included for each comparison.

The league tables were formed using “netleague” function and the number was shown as OR (95% CI), which represented the relative effect between the intervention in the column and that in the row. The color in the background represented the certainty of evidence based on the GRADE approach and four colors represented four GRADE ratings (blue represented “high”, green represented “moderate”, yellow represented “low” and red represented “very low”).

The heterogeneity and global inconsistency were assessed using Cochran’s Q, which was divided into “within designs” (heterogeneity) and “between designs” (inconsistency) using “decomp.design” function. The local inconsistency was assessed using I^2^ statistics, and “netsplit” function was used to split the network estimates into the direct evidence and indirect evidence. The local inconsistency was rated as “low”, “moderate” or “high” if the value of I^2^ was less than 50%, 50%-75% or greater than 75%.

P-score and the surface under the cumulative ranking curve (SUCRA) were used to rank interventions. P-score was calculated based on the point estimates and standard errors of the network estimates, measuring the mean extent of certainty that an intervention was better than another intervention, averaged over all competing interventions [25]. SUCRA was based on the posterior distribution and relevant results were generated (the rankogram plots, the cumulative ranking plots and the SUCRA values). SUCRA was unstable due to reliance on simulations, whose number was determined by the argument “nsim”. The interpretation of P-score and SUCRA was comparable, as both had similar numerical values that ranked the interventions for the specific outcome on a continuous scale from 0 (lowest probability) to 1 (highest probability). Given that P-score took precision into account [25] and SUCRA were unstable based on simulations, we preferentially ranked interventions with P-score like another study [26] and also compared the potential differences between SUCRA and P-score.

The key characteristics of included RCTs were summarized and some data were recalculated for analyses. Transitivity was assessed by comparing the mean age of participants, the percentage of female participants, the mean disease duration of participants and the study duration among all interventions, as well as the box plots were used to show the results.

To assess the robustness of the final results, a sensitivity analysis was performed by excluding trials at high risk of bias. In addition, we categorized the research populations into UC patients and CD patients for a subgroup analysis. Meanwhile, to examine how the study duration affected results, we performed another subgroup analysis based on the study phase (induction phase and maintenance phase) because there were obviously different study durations between them, that is, the maintenance phase-treatment was generally longer than the induction phase-treatment.

Publication bias was assessed through visual inspection of the funnel plot and quantitatively evaluated with the Egger’s test.

## Results

### Study selection

**Figure 1.**
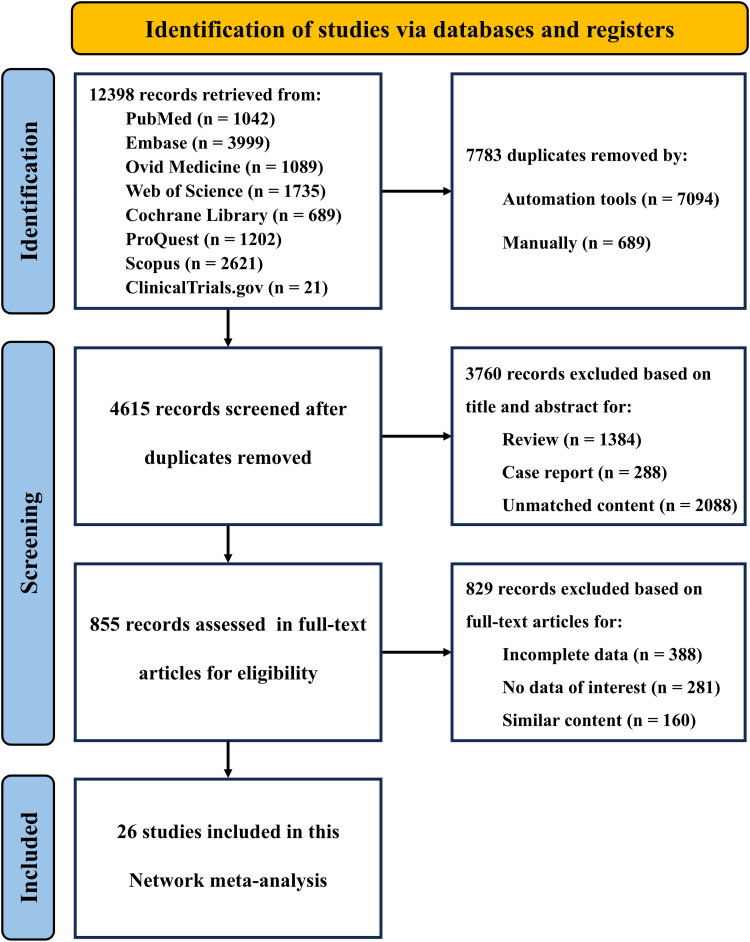
The PRISMA flow diagram of the study selection in the network meta-analysis. PRISMA: the preferred reporting items for systematic reviews and meta-analyses.

The flow diagram was shown in Figure 1. Concisely, we retrieved 12,398 records from the databases and ClinicalTrials.gov. After removing duplicates, the titles and abstracts of 4615 records were reviewed, and 855 were selected for full-text review. Among them, 3 studies (NCT02365649, CTRI/2021/10/037641 and ISRCTN42182437) seemed to meet our inclusion criteria, but their rare doses of JAKinibs limited our network meta-analysis. Ultimately, a total of 26 distinct RCTs met inclusion criteria and were included.

### Study characteristics

Of the 26 included RCTs, 13 RCTs focused on UC patients, 12 on CD patients and 1 on both UC and CD patients. In total, 10,537 IBD patients were included, with 7,608 patients receiving JAKinibs and 2,929 patients receiving the placebo. Among patients receiving JAKinibs, 2,269 received Filgotinib, 2,266 received Upadacitinib, 2,095 received Tofacitinib, 299 received Izencitinib, 176 received Peficitinib, 150 received Ritlecitinib, 142 received Brepocitinib, 123 received Ivarmacitinib and 88 received Deucravacitinib. Together, a total of 17 outcomes of interest were reported: 8 occurred in patients receiving JAKinibs [8 CVE (2 MACE and 6 VTE)] and 9 occurred in patients receiving the placebo [9 CVE (3 MACE and 6 VTE). The main characteristics of the included RCTs were summarized below with median (interquartile ranges). When the dose was not considered, the mean age (years) of participants was 40.80 (39.15-42.45); the percentage (%) of female participants was 43.90% (38.05%-46.65%); the mean disease duration (years) of participants was 7.20 (6.00-9.50) and the study duration (weeks) was 10.00 (8.00-13.00). When the dose was considered, the mean age (years) of participants was 41.00 (39.00-42.70); the percentage (%) of female participants was 44.25% (37.98%-50.00%); the mean disease duration (years) of participants was 7.592 (6.20-10.525) and the study duration (weeks) was 9.00 (8.00-12.00). Supplementary Tables 5 and 6 presented the detailed information of the included RCTs. Transitivity assessment results were shown in Supplementary Figures 1 and 2, revealing generally similar distributions of these key characteristics across all interventions.

### Risks of bias and outcomes for each included RCT

The overall risks of bias for the included RCTs were “low risk”, with 19/26 (73.1%) RCTs rated with “low risk”, 6/26 (23.1%) rated with “some concerns” and 1/26 (3.8%) rated with “high risk”. The common risk of bias arose from the randomisation process. The summary proportions of three ratings for the five domains and the overall bias were shown in Supplementary Figure 3 and the details of the risk of bias for each included RCT were shown in Supplementary Figure 4. Forest plots depicting outcomes of comparisons for each included RCT were shown in Supplementary Figures 5 and 6.

### Network plot

**Figure 2.**
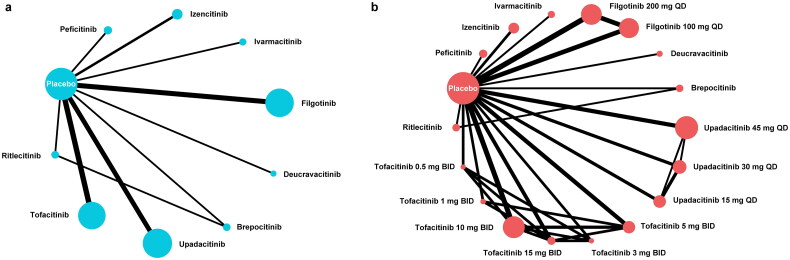
Network plots of the direct comparisons between different interventions, both without (a) and with dose consideration (b).

The network plots (Figure 2) depicted the direct comparisons between different interventions, including 9 JAKinibs and the placebo (without dose consideration) as well as 9 JAKinibs with 17 doses and the placebo (with dose consideration). The evidence distribution of network estimates was shown in Supplementary Figures 7 and 8.

### Network meta-analysis and certainty of evidence

The network meta-analysis results and the corresponding certainty of evidence results were presented in the league tables. The analysis processes of the certainty of evidence based on the GRADE approach were presented in Supplementary Tables 7–10 and the summary direct, indirect and network meta-analysis results, as well as the certainty of evidence were shown in Supplementary Table 11.

### SUCRA and P-score

SUCRA and P-score were calculated to rank the interventions for specific outcomes. The rankogram plots (Supplementary Figures 9 and 10) and cumulative ranking plots (Supplementary Figures 11 and 12) were generated. For SUCRA, the interventions rankings based on SUCRA values were taken as the final rankings to compare with P-score quantitatively (Supplementary Table 12), so as to compare the potential differences between SUCRA and P-score.

### Outcomes

#### MACE (without dose consideration)

**Figure 3.**
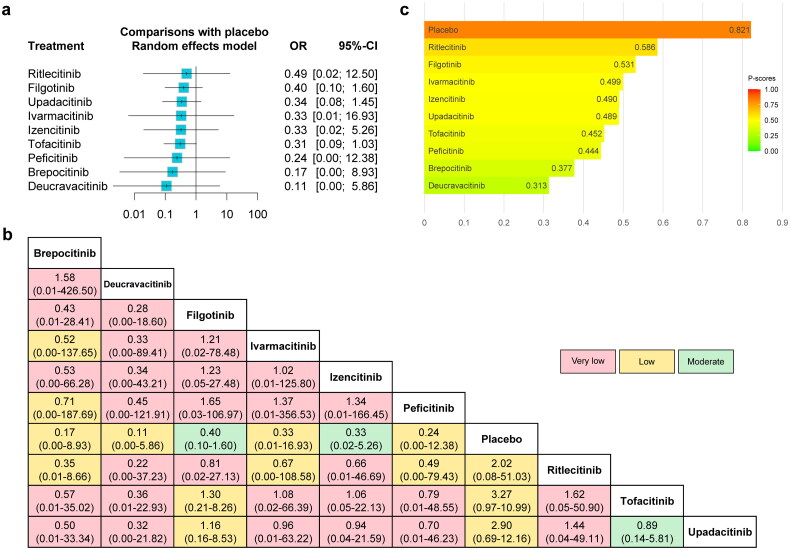
The forest plot (a), league table (b) and P-score plot (c) of different interventions (without dose consideration) for MACE. Annotations: the forest plot represents the comparisons for each treatment with placebo, and ORs higher than 1 favor the corresponding event. The number in the league table is shown as OR (95% CI). The number in the league table represents the relative effect between the intervention in the column and that in the row. The color in the background represents the certainty of evidence according to the GRADE approach. Four colors are used to represent four GRADE ratings (blue represents “high”, green represents “moderate”, yellow represents “low” and red represents “very low”). P-scores are calculated to measure the mean extent of certainty that an intervention is better than another intervention for the specific outcome on a continuous scale from 0 (lowest probability) to 1 (highest probability). Abbreviations: MACE: major adverse cardiovascular events; OR: odds ratio; CI: confidence interval. GRADE: Grading of Recommendations, Assessment, Development and Evaluation.

The forest plot comparing the risk of MACE between JAKinibs and the placebo was presented in Figure 3a, as well as the network meta-analysis results and the certainty of evidence were presented in the league table (Figure 3b). The results showed that JAKinibs were not associated with an increased risk of MACE compared with placebo, and the certainty of evidence ranged from very low to moderate. The results of P-scores (Figure 3c) showed that placebo (P-score = 0.821) ranked highest and Deucravacitinib (0.313) ranked lowest for the risk of MACE compared with other interventions. The SUCRA plots were shown in Supplementary Figures 9a and 11a and the interventions rankings based on SUCRA values demonstrated the same results (Supplementary Table 11). No significant publication bias was found from the funnel plot and the Egger’s test (Supplementary Figure 13a).

#### MACE (with dose consideration)

**Figure 4.**
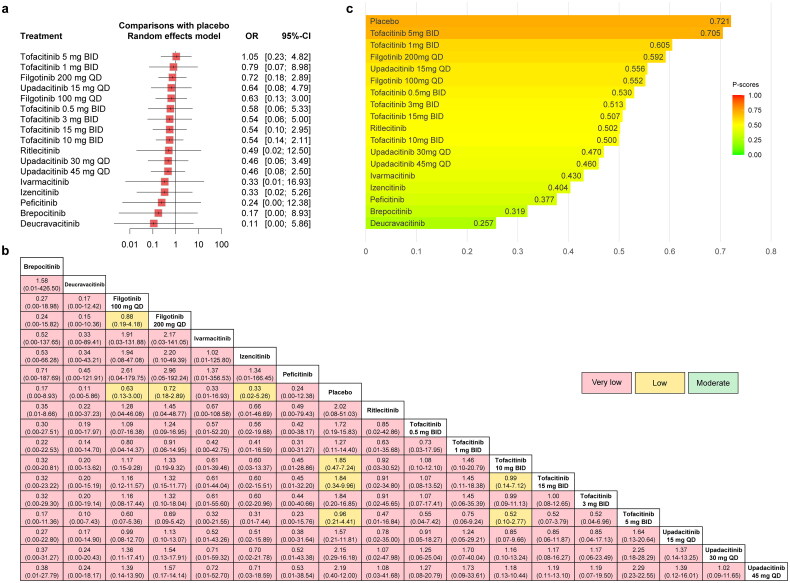
The forest plot (a), league table (b) and P-score plot (c) of different interventions (with dose consideration) for MACE. MACE: major adverse cardiovascular events; OR: odds ratio; CI: confidence interval.

The forest plot comparing the risk of MACE between JAKinibs and the placebo was presented in Figure 4a and the results showed that compared with placebo, Tofacitinib 5 mg BID showed a trend towards an increased risk of MACE (OR = 1.05, 95% CI: 0.23–4.82).

The network meta-analysis results were presented in the league table together with the certainty of evidence (Figure 4b). The results showed that there was no significant variance among comparisons and the certainty of evidence ranged from very low to low. Among them, Tofacitinib 5 mg BID (versus placebo) was rated as low.

The results of P-scores (Figure 4c) showed that placebo (0.721) ranked highest and Deucravacitinib (0.257) ranked lowest for the risk of MACE compared with other interventions, which was the same as the interventions rankings obtained from the analysis when the dose was not considered. The SUCRA plots were shown in Supplementary Figures 10a and 12a and the interventions rankings based on SUCRA values demonstrated similar results (Supplementary Table 11), with slight variations on the rankings of Upadacitinib 15 mg QD (P-score = 0.556; SUCRA = 0.554), Filgotinib 100 mg QD (P-score = 0.552; SUCRA = 0.555), Ritlecitinib (P-score = 0.502; SUCRA = 0.498) and Tofacitinib 10 mg BID (P-score = 0.500; SUCRA = 0.502). Some degree of publication bias was found from the funnel plot and the Egger’s test (*p* = 0.0371, Supplementary Figure 14a).

#### VTE (without dose consideration)

**Figure 5.**
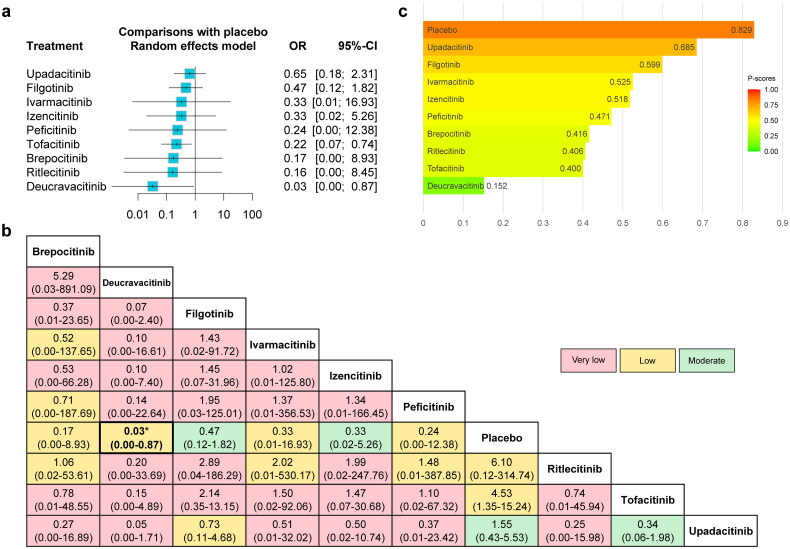
The forest plot (a), league table (b) and P-score plot (c) of different interventions (without dose consideration) for VTE. VTE: venous thromboembolism events; OR: odds ratio; CI: confidence interval.

The forest plot comparing the risk of VTE between JAKinibs and the placebo was presented in Figure 5a, as well as the network meta-analysis results and the certainty of evidence were presented in the league table (Figure 5b). The results showed that compared with placebo, JAKinibs were not associated with an increased risk of VTE, and Deucravacitinib significantly decreased the risk of VTE (OR = 0.03, 95% CI: 0.00-0.87). The certainty of evidence ranged from very low to moderate. Among them, Deucravacitinib (versus placebo) was rated as very low. The results of P-scores (Figure 5c) showed that placebo (0.829) ranked highest and Deucravacitinib (0.152) ranked lowest for the risk of VTE compared with other interventions. The SUCRA plots were shown in Supplementary Figures 9b and 11b and the ­interventions rankings based on SUCRA values demonstrated similar results (Supplementary Table 11), with slight variations on the rankings of Ritlecitinib (P-score = 0.406; SUCRA = 0.402) and Tofacitinib (P-score = 0.400; SUCRA = 0.405). No significant publication bias was found from the funnel plot and the Egger’s test (Supplementary Figure 13b).

#### VTE (with dose consideration)

**Figure 6.**
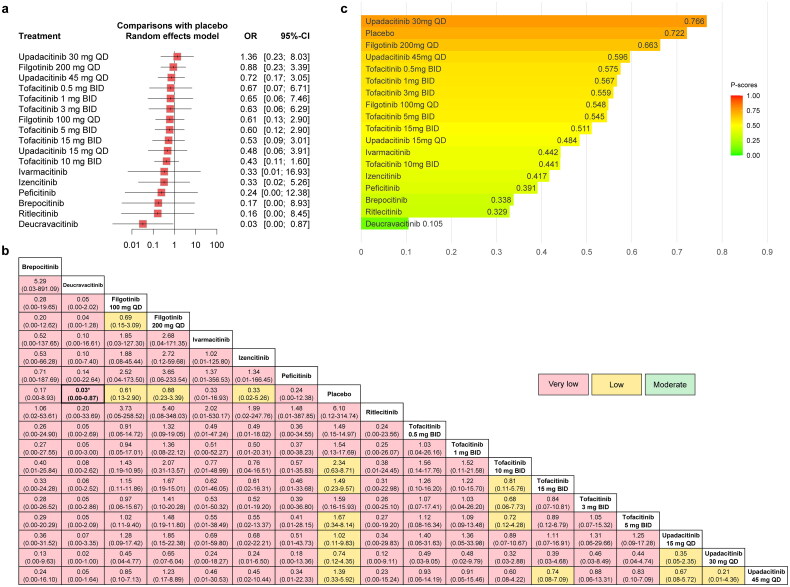
The forest plot (a), league table (b) and P-score plot (c) of different interventions (with dose consideration) for VTE. VTE: venous thromboembolism events; OR: odds ratio; CI: confidence interval.

The forest plot comparing the risk of VTE between JAKinibs and the placebo was presented in Figure 6a and the results showed that compared with placebo, Upadacitinib 30 mg QD showed a trend towards an increased risk of VTE (OR = 1.36, 95% CI: 0.23–8.03), but Deucravacitinib significantly decreased the risk of VTE (OR = 0.03, 95% CI: 0.00-0.87).

The network meta-analysis results were presented in the league table together with the certainty of evidence (Figure 6b). The results showed that only the comparison between Deucravacitinib and the placebo was statistically significant. The certainty of evidence ranged from very low to low. Among them, Upadacitinib 30 mg QD (versus placebo) was rated as low and Deucravacitinib (versus placebo) was rated as very low.

The results of P-scores (Figure 6c) showed that Upadacitinib 30 mg QD (0.766 versus 0.722) ranked higher than placebo for the risk of VTE, which was different from the interventions rankings obtained from the analysis when the dose was not considered. In addition, Deucravacitinib (0.105) still ranked lowest for the risk of VTE compared with other interventions. The SUCRA plots were shown in Supplementary Figures 10b and 12b and the interventions rankings based on SUCRA values demonstrated the same results (Supplementary Table 11). Some degree of publication bias was found from the funnel plot and the Egger’s test (*p* = 0.0198, Supplementary Figure 14b).

#### CVE (without dose consideration)

**Figure 7.**
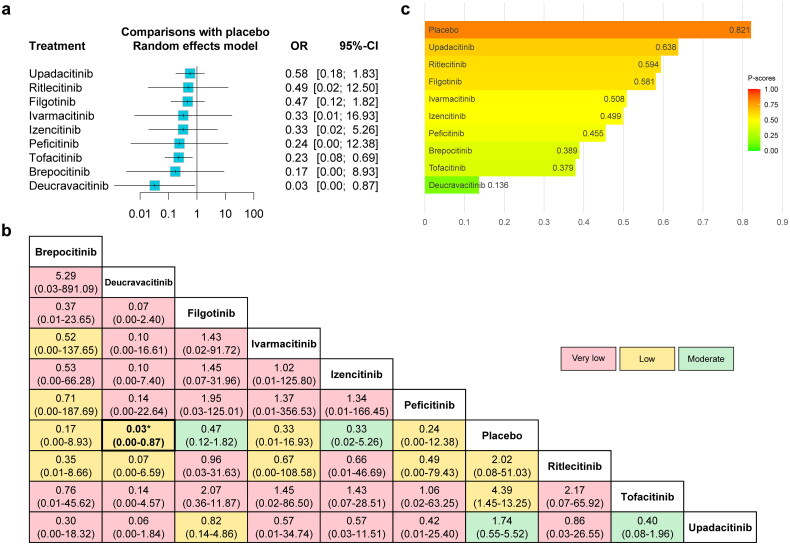
The forest plot (a), league table (b) and P-score plot (c) of different interventions (without dose consideration) for CVE. CVE: cardiovascular events; OR: odds ratio; CI: confidence interval.

The forest plot comparing the risk of CVE between JAKinibs and the placebo was presented in Figure 7a, as well as the network meta-analysis results and the certainty of evidence were presented in the league table (Figure 7b). The results showed that compared with placebo, JAKinibs were not associated with an increased risk of CVE, and Deucravacitinib significantly decreased the risk of CVE (OR = 0.03, 95% CI: 0.00-0.87). The certainty of evidence ranged from very low to moderate. Among them, Deucravacitinib (versus placebo) was rated as very low. The results of P-scores (Figure 7c) showed that placebo (0.821) ranked highest and Deucravacitinib (0.136) ranked lowest for the risk of CVE compared with other interventions. The SUCRA plots were shown in Supplementary Figures 9c and 11c and the interventions rankings based on SUCRA values demonstrated the same results (Supplementary Table 11). No significant publication bias was found from the funnel plot and the Egger’s test (Supplementary Figure 13c).

#### CVE (with dose consideration)

**Figure 8.**
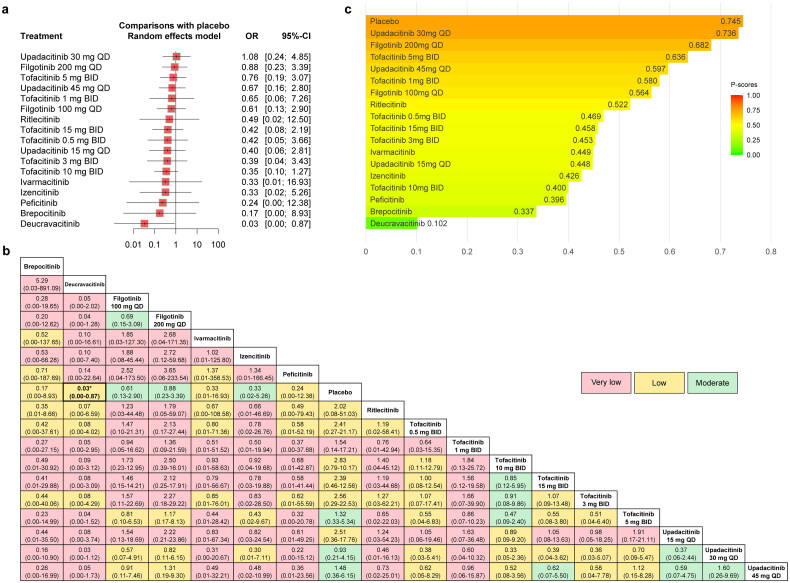
The forest plot (a), league table (b) and P-score plot (c) of different interventions (with dose consideration) for CVE. CVE: cardiovascular events; OR: odds ratio; CI: confidence interval.

The forest plot comparing the risk of CVE between JAKinibs and the placebo was presented in Figure 8a and the results showed that compared with placebo, Upadacitinib 30 mg QD showed a trend towards an increased risk of CVE (OR = 1.08, 95% CI: 0.24–4.85), but Deucravacitinib significantly decreased the risk of CVE (OR = 0.03, 95% CI: 0.00-0.87).

The network meta-analysis results were presented in the league table together with the certainty of evidence (Figure 8b). The results showed that only the comparison between Deucravacitinib and the placebo was statistically significant. The certainty of evidence ranged from very low to moderate. Among them, Upadacitinib 30 mg QD (versus placebo) was rated as moderate and Deucravacitinib (versus Placebo) was rated as low.

The results of P-scores (Figure 8c) showed that placebo (0.745) ranked highest and Deucravacitinib (0.102) ranked lowest for the risk of CVE compared with other interventions, which was the same as the interventions rankings obtained from the analysis when the dose was not considered. The SUCRA plots were shown in Supplementary Figures 10c and 12c and the interventions rankings based on SUCRA values demonstrated similar results (Supplementary Table 11), with slight variations on the rankings of Ivarmacitinib (P-score = 0.449; SUCRA = 0.452) and Upadacitinib 15 mg QD (P-score = 0.448; SUCRA = 0.452). No significant publication bias was found from the funnel plot and the Egger’s test (Supplementary Figure 14c).

#### Heterogeneity and inconsistency analysis

Cochran’s Q was used to assess heterogeneity and global inconsistency, revealing low heterogeneity and no significant global inconsistency (Supplementary Table 13). In addition, I^2^ statistics was used to assess local inconsistency, also showing no significant variances between the direct and indirect evidence (Supplementary Figures 15 and 16).

#### Sensitivity and subgroup analyses

The results were reanalyzed through the sensitivity analysis (excluding a trial at high risk of bias) and subgroup analyses [based on the IBD type (UC patients and CD patients) and study phase (induction phase and maintenance phase)] to assess their robustness. The findings of the sensitivity analysis were similar with the initial results (Supplementary Figure 17). As for the subgroup analysis based on the IBD type, in UC patients, no obvious difference was found compared with the initial results when the dose was not considered, as well as Filgotinib 200 mg QD and Upadacitinib 30 mg QD showed the opposite trend of cardiovascular risks compared with the initial results, despite no statistically significant difference (Supplementary Figure 18). In CD patients, when the dose was not considered, there was also no obvious difference compared with the initial results, as well as Tofacitinib 5 mg BID, Tofacitinib 10 mg and Upadacitinib 15 mg QD showed a trend towards increased cardiovascular risks compared with the initial results, despite only slight increasing trends (OR = 1.01) and no statistically significant difference (Supplementary Figure 19). Similar results were obtained when carrying out subgroup analysis based on the study phase. In the induction phase, Tofacitinib 5 mg BID, Upadacitinib 15 mg QD and Upadacitinib 30 mg QD, as well as in the maintenance phase, Upadacitinib 15 mg QD all showed the opposite trend compared with the initial results, despite no statistically significant difference (Supplementary Figures 20 and 21). Meantime, in the maintenance phase, Upadacitinib showed a trend towards an increased cardiovascular risk with insignificant difference (Supplementary Figure 21). Overall, the results of sensitivity analysis and subgroup analyses were similar with the initial results, indicating good robustness.

## Discussion

This network meta-analysis of 26 RCTs involving 10,537 patients assessed the risks of MACE, VTE and CVE associated with JAKinibs in IBD patients. Our results showed that JAKinibs did not increase the risks of MACE, VTE and CVE in IBD patients, and the initial results did not change when the sensitivity and subgroup analyses were conducted. However, when the dose was considered, Tofacitinib 5 mg BID (versus placebo) showed a trend towards an increased risk of MACE, as well as Upadacitinib 30 mg QD (versus placebo) showed a trend towards increased risks of VTE and CVE, and ranked higher than placebo for the risk of VTE. Notably, Deucravacitinib ranked lowest for the risks of MACE, VTE and CVE, as well as significantly decreased the risks of VTE and CVE.

As a chronic inflammatory disease of the gastrointestinal tract, IBD is associated with extraintestinal manifestations with prevalence rates reported from 15% to 30% [27]. Among them, cardiovascular adverse events are common. Several studies have showed that IBD patients had higher risks of VTE (HR = 3.4, 95% CI: 2.7-4.3) and stroke (HR = 1.29, 95% CI: 1.16-1.43) [28,29] and thereby cardiovascular safety was a key factor in clinical decision-making. As a new kind of drug, JAKinibs have been widely used for their curative effect. However, a 4-year safety end-point trial [Oral Rheumatoid Arthritis Trial (ORAL) surveillance study] involving patients with active rheumatoid arthritis (RA) found cardiovascular risks associated with JAKinibs [9]. Findings demonstrated that Tofacitinib 10 mg BID increased the risk of VTE (HR = 3.52, 95% CI: 1.74–7.12), and associated with a trend towards an increased risk of MACE (HR = 1.43, 95% CI: 0.94–2.18), although the increased risks of VTE (HR = 1.66, 95% CI: 0.76–3.63) and MACE (HR = 1.24, 95% CI: 0.81–1.91) in the patients treated with Tofacitinib 5 mg BID were both insignificantly. These findings raised concerns about cardiovascular risks associated with JAKinibs, and both the EMA and the FDA issued relevant black box warnings. Nonetheless, it was uncertain whether cardiovascular adverse events were the effect of JAKinibs. A multi-database study demonstrated that treatment with Baricitinib was associated with a significantly increased risk of VTE compared with the TNF inhibitor [incidence rate ratio (IRR) = 1.51, 95% CI: 1.10-2.08], although no significantly increased risk of MACE was observed (IRR = 1.54, 95% CI: 0.93-2.54) [6]. Another nationwide cohort study indicated no significant difference in the risks of VTE [Weighted hazard ratio (HRw) = 1.2, 95% CI: 0.7-1.8] and MACE (HRw = 1.1, 95% CI: 0.7-1.8) between Baricitinib and Adalimumab (a TNF inhibitor), and no significantly increased risk of VTE (HRw = 1.1, 95% CI: 0.6-1.8) and MACE (HRw = 0.8, 95% CI: 0.4-1.4) was found when Tofacitinib was compared with Adalimumab [5].

Therefore, given the uncertain relation between JAKinibs and cardiovascular adverse events, coupled with insufficient research on this matter in IBD patients, this network meta-analysis pooled all relevant RCTs and did not find sufficient evidence to support the increased cardiovascular risks associated with JAKinibs in IBD patients. Some factors might explain different findings. Patients in the ORAL surveillance study were older (mean age > 60 years) and had at least one additional cardiovascular risk factor (current cigarette smoker, hypertension, high-density lipoprotein cholesterol level of < 40 mg per deciliter, diabetes mellitus, family history of premature coronary heart disease, extraarticular rheumatoid arthritis, or history of coronary artery disease), along with long study durations (mean study duration > 38 months). In contrast, Patients in our included RCTs were younger (mean age < 50 years), along with shorter study durations (the longest study duration = 52 weeks). Similarly, studies based on databases also had the characteristics of long study durations. These differences enabled these studies to effectively identify cardiovascular adverse events that were rare and needed time to develop. In addition, the ORAL surveillance study was concluded by comparing JAKinibs with the TNF inhibitor (Adalimumab), while the results in our study were concluded by comparing with the placebo. Differences in the disease type, age composition, cardiovascular risk factors, study duration and control group might help to explain different findings. Therefore, any potential harms associated with JAKinibs should be comprehensively evaluated after recognizing the potential influences of these factors.

Our findings were consistent with the majority of meta-analyses on JAKinibs and cardiovascular risks [30–33], except for a study conducted by Muhammad Haisum Maqsood et al. This study demonstrated that treatment with JAKinibs were associated with a trend towards an increased risk of VTE in patients with immune-mediated inflammatory diseases (OR = 1.65, 95% CI: 0.97-2.79) and the increased risk became significant when trials lasting over 12 months were analyzed separately (OR = 2.17, 95% CI: 1.16-4.05) [34]. However, the primary results of this meta-analysis came from a long-term study involving patients with cardiovascular risk factors (ORAL surveillance study) [9], which might potentially bias the results towards a signal-to-harm across JAKinibs. Therefore, to better explore the relation between confounding factors and cardiovascular adverse events, we conducted a subgroup analysis based on the study phase (induction phase and maintenance phase). Nevertheless, the results were consistent with the initial analysis, likely because the longest study duration in the included RCTs was only 52 weeks, much shorter than studies such as the ORAL surveillance study (mean study duration > 38 months). In addition, age and cardiovascular risk factors might also contribute to the different findings. However, the included RCTs did not provide sufficient data on cardiovascular risk factors, and the age variation of participants in the included RCTs was minimal, limiting the further study. Therefore, to develop a more thorough understanding of the cardiovascular adverse events associated with JAKinibs, the cardiovascular risk factors needed to be taken into account and the underlying mechanisms between JAKinibs and cardiovascular adverse events also needed to be explored in the future.

In general, there is a positive relation between well-controlled inflammatory state and decreased cardiovascular risks [35–37]. Therefore, under this cognitive framework, JAKinibs, which significantly reduce inflammation, should mitigate the cardiovascular risks, but obviously, there was a complicated relation between them. Current JAKinibs act by competitively blocking the adenosine triphosphate (ATP) site in the JAK homology (JH) 1 domain [12]. However, due to the high conservation of the JH1 domain among JAK subtypes, high doses of JAKinibs are closely related to the nonselective pan-JAK blockade and undesirable adverse reactions (off-target effects) [12, 38]. Therefore, considering the selective targeting of the JAKinibs was dose-dependent, we assumed that the relation between JAKinibs and cardiovascular adverse events would be mediated by high-dose JAKinibs-related off-target effects, and thereby analyzed based on whether or not the dose was considered. Findings demonstrated that when the dose was no considered, placebo ranked highest for the risks of MACE, VTE and CVE. However, when the dose was considered, Tofacitinib 5 mg BID showed a trend towards an increased risk of MACE, as well as Upadacitinib 30 mg QD showed a trend towards increased risks of VTE and CVE, and ranked higher than placebo for the risk of VTE. Nevertheless, no significantly increased cardiovascular risk was found, and especially no high-dose JAKinibs-related off-target effects were found, which still needed more studies to verify in the future. In view of the fact that the withdrawal of JAKinibs was often due to adverse reactions rather than ineffectiveness [39], combination therapy might be a good treatment option [40]. At present, this therapy has been applied to patients with immune-mediated inflammatory diseases (NCT04870203), and findings showed that the combination of Baricitinib and the TNF inhibitor improved treatment efficacy for RA patients without obvious acute adverse effects. Therefore, further researches in IBD patients are warranted.

Currently, the development of JAKinibs focuses on selectively targeting specific JAK subtypes to maximize clinical efficacy, and minimize nonselective pan-JAK blockade and undesirable off-target effects [41]. In addition, researchers are increasingly focusing on the development of TYK2 (Tyrosine kinase 2) inhibitors because TYK2 primarily works in the immune-related signaling pathways [42], compared to other JAK subtypes (JAK1, JAK2 and JAK3) involving a wide range of systemic signaling pathways, ensuring the safety of targeted inhibition of TYK2 [16]. At present, the only TYK2 inhibitor studied in IBD patients is Deucravacitinib, which targets the JH2 pseudokinase regulatory domain of TYK2 and suppresses its catalytic activity through allosteric regulation, allowing for more precise regulating [16]. As is known to us, Deucravacitinib was the only JAKinib without any black box warning and previous studies also demonstrated that it could effectively improve skin injuries in psoriasis patients without serious adverse events like thrombosis or death [17,18]. Similar to previous findings, our study demonstrated that Deucravacitinib ranked lowest for the risks of MACE, VTE and CVE, and was the only JAKinib that significantly decreased the risks of VTE and CVE in IBD patients. All these results provided a new direction for the development and utilization of JAKinibs in IBD patients. Besides the RCTs included in our study, several ongoing trials of Deucravacitinib (NCT06136403, NCT03920267, NCT04772079) are expected to provide further insights.

Considering the uncertain relation between JAKinibs and cardiovascular adverse events, as well as the urgent need for rational clinical administration of JAKinibs, developing a suitable monitoring and assessment system may be an effective method to predict and prevent cardiovascular adverse events. An early clinical trial found that patients treated with the JAKinib had an elevated platelet level [43]. The ORAL surveillance study found the serum lipid levels (fasting triglycerides, high density lipoprotein cholesterol, low density lipoprotein cholesterol and total cholesterol) were higher in RA patients treated with Tofacitinib than in those treated with the TNF inhibitor, which might be associated with an increased risk of VTE (HR = 3.52, 95% CI: 1.74–7.12) and a trend towards an increased risk of MACE (HR = 1.43, 95% CI: 0.94–2.18) found in patients treated with the JAKinib [9]. These findings provided initial predictors of cardiovascular adverse events associated with JAKinibs and relevant researches were also carried out. A cross-sectional cohort study of patients with autoimmune rheumatic diseases found that a model combining age and deprivation decile effectively predicted cardiovascular adverse events in those treated with the JAKinib [44]. Another exploratory post hoc analysis found the month 12 D-dimer level was positively correlated with the risk of subsequent VTE in the Tofacitinib group [45]. Although this was not the main objective of our study, we believed that developing a monitoring and assessment system for cardiovascular adverse events in patients treated with JAKinibs would help to improve the clinical prognosis.

Our study had several strengths. First, the present study filled the important literature gap. To our knowledge, this was the first comprehensive network meta-analysis specifically assessing the risks of MACE, VTE and CVE associated with JAKinibs in IBD patients. Second, this study encompassed all relevant RCTs of JAKinibs in IBD patients, integrating both published and unpublished results for comprehensiveness. Third, network meta-analysis provided the estimates for comparisons among all interventions even without direct head-to-head comparisons in RCTs, as well as the additional interventions rankings and the certainty of evidence provided more relevant information, allowing clinicians to make better decisions. Fourth, sensitivity and subgroup analyses were performed to assess the potential influence of relevant factors on the results. Despite the multitude of RCTs, the consistent findings indicated robust results. Last, effective visualization and comprehensive reporting of processes and results increased the trustworthiness of our study.

However, our study also had some limitations. First, although RCTs provide the highest level of evidence, their strict inclusion criteria mean the study populations in our study may not represent the diverse patient populations in clinical practice, thus limiting the external validity of our results. Second, short-term safety data from RCTs may not provide enough evidence for the analysis of long-term adverse reactions of JAKinibs in clinical practice, especially cardiovascular adverse events that needed time to develop. Third, the included RCTs may not primarily focus on cardiovascular adverse events, making it challenging to get enough information like the cardiovascular risk factors, racial characteristics, disease activity, concomitant medication and occurrence time of these events. The lack of relevant information made it difficult to attribute the increased cardiovascular risks solely to JAKinibs, affecting the accurate assessment of their relation. Fourth, although the included RCTs encompassed all JAKinibs for IBD patients, few studies evaluated two or more JAKinibs simultaneously. As a result, many effect estimates in our study were based on indirect comparisons, decreasing the certainty of the results. Fifth, all control groups in our included RCTs were treated with placebo, and their ongoing active inflammation might increase the cardiovascular risks, potentially counteracting the risks that might be associated with JAKinibs in the comparison. Sixth, our study failed to summarize the results of some uncontrolled trials due to the difficulty of drawing general conclusions without the control group. Seventh, despite conducting a thorough literature search, we found limited numbers of RCTs and outcomes of interest, particularly for the newly developed JAKinibs like Deucravacitinib. Consequently, we merged data on different doses of these newly developed JAKinibs and assessed the cardiovascular risks of JAKinibs without considering dose, as well as we also assessed cardiovascular risks of JAKinibs considering dose for a better understanding of dose effects on specific outcomes. Although merging data on different doses increased the sample size of a certain JAKinib, it might influence the accurate risk assessment and dose-risk analysis. Last, we included RCTs that reported no cardiovascular adverse events to minimize selection bias, but some publication biases for specific outcomes still persisted. Based on this, we applied a fixed continuity correction and analyzed most data to enable the effective calculation for zero-count event, which might reduce the statistical power and impact the accuracy of the results. Given these limitations, the results of our study should be interpreted cautiously. Nevertheless, our study provided the latest evidence on the cardiovascular risks in IBD patients treated with JAKinibs and suggested feasible directions for future study.

## Conclusions

In summary, our evaluation of cardiovascular safety was reassuring and supported that the use of JAKinibs in IBD patients will not increase the risks of MACE, VTE and CVE, despite some minor dose-related increasing trends. Encouragingly, Deucravacitinib, a newly developed selective TYK2 inhibitor, ranked lowest for the risks of MACE, VTE and CVE, as well as significantly decreased the risks of VTE and CVE. More data was required to validate our findings. Fortunately, several ongoing clinical trials with unpublished results (NCT04870203, NCT06136403, NCT03920267 and NCT04772079) will help to improve the understanding of the relation between JAKinibs and cardiovascular adverse events, and provide clinicians with more valuable insights for JAKinibs therapy.

## Supplementary Material

Supplemental Material

## Data Availability

The protocol is available on PROSPERO (ID: CRD42024524803). Data can be obtained by e-mailing the corresponding author (Bryan_yang323@163.com) for valid reasons.
