# Supplementary material for: Cardiovascular safety of Janus kinase inhibitors in inflammatory bowel disease: a systematic review and network meta-analysis
Source: Ann Med. 2025 Jan 21;57(1):2455536. doi: 10.1080/07853890.2025.2455536 (PMC11755742; doi:10.1080/07853890.2025.2455536)
Supplement: Supplemental Material [file IANN_A_2455536_SM7544.zip › suppl_data/Annex 1.docx]

| Number | Study | Register number | Study phase | Intervention | Sample size | Race | | | | | | | | | | | | | | |
| --- | --- | --- | --- | --- | --- | --- | --- | --- | --- | --- | --- | --- | --- | --- | --- | --- | --- | --- | --- | --- |
|  |  |  |  |  |  | White | Black | Asian | Others | Black or African  American | American Indian or  Alaska Native | Native Hawaiian and  other Pacific Islander | Multiple | American Indian or Alaska Native, Black  or African American, or not permitted | Caucasian | Not Permitted | Unknown or Not Reported | More than one race | Hispanic or Latino | Not Hispanic or Latino |
| 1 | William J. Sandborn 2013 | NCT00615199 | Induction | Placebo BID | 34 | 32 (94.1) |  |  |  |  |  |  |  |  |  |  |  |  |  |  |
|  |  |  |  | Tofacitinib 1 mg BID | 36 | 34 (94.4) |  |  |  |  |  |  |  |  |  |  |  |  |  |  |
|  |  |  |  | Tofacitinib 5 mg BID | 34 | 28 (82.4) |  |  |  |  |  |  |  |  |  |  |  |  |  |  |
|  |  |  |  | Tofacitinib 15 mg BID | 35 | 31 (88.6) |  |  |  |  |  |  |  |  |  |  |  |  |  |  |
| 2 | William J. Sandborn 2013 | NCT00787202 | Induction | Placebo BID | 48 | 43 (90.0) |  |  |  |  |  |  |  |  |  |  |  |  |  |  |
|  |  |  |  | Tofacitinib 0.5 mg BID | 31 | 28 (90.0) |  |  |  |  |  |  |  |  |  |  |  |  |  |  |
|  |  |  |  | Tofacitinib 3 mg BID | 33 | 30 (90.9) |  |  |  |  |  |  |  |  |  |  |  |  |  |  |
|  |  |  |  | Tofacitinib 10 mg BID | 33 | 30 (90.9) |  |  |  |  |  |  |  |  |  |  |  |  |  |  |
|  |  |  |  | Tofacitinib 15 mg BID | 49 | 45 (91.8) |  |  |  |  |  |  |  |  |  |  |  |  |  |  |
| 3 | Julian Panés 2017 | NCT01393626 | Induction | Placebo BID | 91 | 78 (85.7) | 6 (6.6) | 5 (5.5) | 2 (2.2) |  |  |  |  |  |  |  |  |  |  |  |
|  |  |  |  | Tofacitinib 5 mg BID | 86 | 71 (82.6) | 1 (1.2) | 12 (14.0) | 2 (2.3) |  |  |  |  |  |  |  |  |  |  |  |
|  |  |  |  | Tofacitinib 10 mg BID | 86 | 72 (83.7) | 2 (2.3) | 11 (12.8) | 1 (1.2) |  |  |  |  |  |  |  |  |  |  |  |
| 4 | Julian Panés 2016 | NCT01393899 | Maintenance | Placebo BID | 59 | 44 (74.6) | 3 (5.1) | 10 (16.9) | 2 (3.4) |  |  |  |  |  |  |  |  |  |  |  |
|  |  |  |  | Tofacitinib 5 mg BID | 60 | 50 (83.3) | 0 | 9 (15.0) | 1 (1.7) |  |  |  |  |  |  |  |  |  |  |  |
|  |  |  |  | Tofacitinib 10 mg BID | 61 | 50 (82.0) | 3 (4.9) | 8 (13.1) | 0 (0) |  |  |  |  |  |  |  |  |  |  |  |
| 5 | William J. Sandborn, 2017 | NCT01458574 | Maintenance | Placebo BID | 198 | No relevant content |  |  |  |  |  |  |  |  |  |  |  |  |  |  |
|  |  |  |  | Tofacitinib 5 mg BID | 198 | No relevant content |  |  |  |  |  |  |  |  |  |  |  |  |  |  |
|  |  |  |  | Tofacitinib 10 mg BID | 197 | No relevant content |  |  |  |  |  |  |  |  |  |  |  |  |  |  |
| 6 | William J. Sandborn, 2016 | NCT01458951 | Induction | Placebo BID | 112 | No relevant content |  |  |  |  |  |  |  |  |  |  |  |  |  |  |
|  |  |  |  | Tofacitinib 10 mg BID | 429 | No relevant content |  |  |  |  |  |  |  |  |  |  |  |  |  |  |
| 7 | William J. Sandborn 2016 | NCT01465763 | Induction | Placebo BID | 122 | No relevant content |  |  |  |  |  |  |  |  |  |  |  |  |  |  |
|  |  |  |  | Tofacitinib 10 mg BID | 476 | No relevant content |  |  |  |  |  |  |  |  |  |  |  |  |  |  |
| 8.1 | William J. Sandborn 2022 | NCT02819635 | Induction (Substudy 1) | Placebo QD | 46 | 37 (80.4) |  | 8 (17.4) |  | 0 | 0 | 0 | 1 (2.2) |  |  |  |  |  |  |  |
|  |  |  |  | Upadacitinib 15 mg QD | 49 | 38 (77.6) |  | 10 (20.4) |  | 1 (2.0) | 0 | 0 | 0 |  |  |  |  |  |  |  |
|  |  |  |  | Upadacitinib 30 mg QD | 117 | 88 (75.2) |  | 23 (19.7) |  | 3 (2.6) | 1 (0.9) | 0 | 2 (1.7) |  |  |  |  |  |  |  |
|  |  |  |  | Upadacitinib 45 mg QD | 123 | 90 (73.2) |  | 28 (22.8) |  | 2 (1.6) | 0 | 0 | 3 (2.4) |  |  |  |  |  |  |  |
| 8.2 |  |  | Induction (Substudy 2) | Placebo QD | 155 | 100 (64.9) |  | 46 (29.9) |  | 4 (2.6) | 2 (1.3) | 0 | 2 (1.3) |  |  |  |  |  |  |  |
|  |  |  |  | Upadacitinib 45 mg QD | 319 | 206 (64.6) |  | 95 (29.8) |  | 12 (3.8) | 0 | 1 (0.3) | 5 (1.6) |  |  |  |  |  |  |  |
| 8.3 |  |  | Maintenance | Placebo QD | 149 | 93 (62.4) |  | 42 (28.2) |  | 6 (4.0) | 0 | 1 (0.7) | 7 (4.7) |  |  |  |  |  |  |  |
|  |  |  |  | Upadacitinib 15 mg QD | 148 | 97 (65.5) |  | 44 (29.7) |  | 7 (4.7) | 0 | 0 | 0 |  |  |  |  |  |  |  |
|  |  |  |  | Upadacitinib 30 mg QD | 154 | 101 (65.6) |  | 48 (31.2) |  | 3 (1.9) | 0 | 1 (0.6) | 1 (0.6) |  |  |  |  |  |  |  |
| 9.1 | Brian G Feagan 2021 | NCT02914522 | Induction  (biologic-naive patients) | Placebo QD | 137 | 95 (69.3) |  | 38 (27.7) | 2 (1.5) | 1 (0.7) | 0 |  |  |  |  | 1 (0.7) |  |  |  |  |
|  |  |  |  | Filgotinib 100 mg QD | 277 | 192 (69.3) |  | 79 (28.5) | 2 (0.7) | 3 (1.1) | 0 |  |  |  |  | 1 (0.4) |  |  |  |  |
|  |  |  |  | Filgotinib 200 mg QD | 245 | 165 (67.3) |  | 77 (31.4) | 0 | 2 (0.8) | 1 (0.4) |  |  |  |  | 0 |  |  |  |  |
| 9.2 |  |  | Induction  (biologic-experienced patients) | Placebo QD | 142 | 98 (69.0) |  | 27 (19.0) | 1 (0.7) | 3 (2.1) | 0 |  |  |  |  | 13 (9.2) |  |  |  |  |
|  |  |  |  | Filgotinib 100 mg QD | 285 | 212 (74.4) |  | 51 (17.9) | 0 | 6 (2.1) | 0 |  |  |  |  | 16 (5.6) |  |  |  |  |
|  |  |  |  | Filgotinib 200 mg QD | 262 | 190 (72.5) |  | 50 (19.1) | 0 | 4 (1.5) | 0 |  |  |  |  | 18 (6.9) |  |  |  |  |
| 10.1 | Séverine Vermeire 2023 | NCT02914561 | Induction (biologic-naive patients) | Placebo QD | 239 | 185 (77.4) |  | 44 (18.4) | 2 (0.8) | 4 (1.7) | 0 | 0 |  |  |  |  | 4 (1.7) |  |  |  |
|  |  |  |  | Filgotinib 100 mg QD | 245 | 179 (73.1) |  | 52 (21.2) | 3 (1.2) | 3 (1.2) | 0 | 0 |  |  |  |  | 5 (2.0) |  |  |  |
|  |  |  |  | Filgotinib 200 mg QD | 223 | 166 (74.4) |  | 45 (20.2) | 2 (0.9) | 4 (1.8) | 0 | 0 |  |  |  |  | 7 (3.1) |  |  |  |
| 10.2 |  |  | Induction  (biologic-experienced patients) | Placebo QD | 231 | 178 (77.1) |  | 31 (13.4) | 1 (0.4) | 6 (2.6) | 0 | 2 (0.9) |  |  |  |  | 13 (5.6) |  |  |  |
|  |  |  |  | Filgotinib 100 mg QD | 230 | 182 (79.1) |  | 25 (10.9) | 0 | 9 (3.9) | 1 (0.4) | 1 (0.4) |  |  |  |  | 12 (5.2) |  |  |  |
|  |  |  |  | Filgotinib 200 mg QD | 204 | 158 (77.5) |  | 24 (11.8) | 3 (1.5) | 6 (2.9) | 0 | 0 |  |  |  |  | 13 (6.4) |  |  |  |
| 11 | Walter Reinisch 2022 | NCT03077412 | Induction | Placebo QD | 15 | 14 (93.3) |  | 0 | 0 | 1 (6.7) | 0 | 0 |  |  |  |  | 0 |  |  |  |
|  |  |  |  | Filgotinib 100 mg QD | 25 | 19 (76.0) |  | 2 (8.0) | 0 | 2 (8.0) | 0 | 0 |  |  |  |  | 2 (8.0) |  |  |  |
|  |  |  |  | Filgotinib 200 mg QD | 17 | 15 (88.2) |  | 0 | 0 | 1 (5.9) | 0 | 0 |  |  |  |  | 1 (5.9) |  |  |  |
| 12 | William J. Sandborn 2022 | NCT02958865 | Induction | Placebo QD | 25 | 22 (88.0) |  | 1 (4.0) | 1 (4.0) | 1 (4.0) |  |  |  |  |  |  |  |  |  |  |
|  |  |  |  | Ritlecitinib 20 mg QD | 51 | 46 (90.2) |  | 3 (5.9) | 1 (2.0) | 1 (2.0) |  |  |  |  |  |  |  |  |  |  |
|  |  |  |  | Ritlecitinib 70 mg QD | 49 | 46 (93.9) |  | 1 (2.0) | 0 | 2 (4.1) |  |  |  |  |  |  |  |  |  |  |
|  |  |  |  | Ritlecitinib 200 mg QD | 50 | 48 (96.0) |  | 0 | 1 (2.0) | 1 (2.0) |  |  |  |  |  |  |  |  |  |  |
|  |  |  |  | Brepocitinib 10 mg QD | 48 | 44 (91.7) |  | 3 (6.3) | 1 (2.1) | 0 |  |  |  |  |  |  |  |  |  |  |
|  |  |  |  | Brepocitinib 30 mg QD | 47 | 44 (93.6) |  | 3 (6.4) | 0 | 0 |  |  |  |  |  |  |  |  |  |  |
|  |  |  |  | Brepocitinib 60 mg QD | 47 | 45 (95.7) |  | 0 | 1 (2.1) | 1 (2.1) |  |  |  |  |  |  |  |  |  |  |
| 13 | Geert R D'Haens 2021 | NCT03046056 | Induction | Placebo QD | 18 | 16 (88.9) |  | 0 | 0 | 2 (11.1) | 0 | 0 |  |  |  |  | 0 |  |  |  |
|  |  |  |  | Filgotinib 100 mg QD | 32 | 28 (87.5) |  | 0 | 0 | 4 (12.5) | 0 | 0 |  |  |  |  | 0 |  |  |  |
|  |  |  |  | Filgotinib 200 mg QD | 28 | 25 (89.3) |  | 0 | 1 (3.6) | 2 (7.1) | 0 | 0 |  |  |  |  | 0 |  |  |  |
| 14 | Walter Reinisch 2023 | NCT03201445 | Induction | Placebo QD | 67 | 31 (46.3) |  | 35 (52.2) |  |  |  |  |  | 1 (1.5) |  |  |  |  |  |  |
|  |  |  |  | Filgotinib 200 mg QD | 66 | 26 (39.4) |  | 37 (56.1) |  |  |  |  |  | 3 (4.5) |  |  |  |  |  |  |
| 15 | Edward V Loftus Jr 2022 | NCT03345836 | Induction | Placebo QD | 171 | 126 (73.7) |  | 38 (22.2) |  | 6 (3.5) | 1 (0.6) | 0 |  |  |  |  | 0 | 0 |  |  |
|  |  |  |  | Upadacitinib 45 mg QD | 324 | 230 (71.0) |  | 69 (21.3) |  | 19 (5.9) | 1 (0.3) | 0 |  |  |  |  | 0 | 5 (1.5) |  |  |
| 16 | Edward V Loftus Jr 2022 | NCT03345849 | Induction | Placebo QD | 176 | 130 (73.9) |  | 36 (20.5) |  | 4 (2.3) | 0 | 0 |  |  |  |  | 0 | 6 (3.4) |  |  |
|  |  |  |  | Upadacitinib 45 mg QD | 350 | 258 (73.7) |  | 73 (20.9) |  | 17 (4.9) | 0 | 0 |  |  |  |  | 0 | 2 (0.6) |  |  |
| 17 | Edward V Loftus Jr 2023 | NCT03345823 | Maintenance | Placebo QD | 165 | No relevant content |  |  |  |  |  |  |  |  |  |  |  |  |  |  |
|  |  |  |  | Upadacitinib 15 mg QD | 169 | No relevant content |  |  |  |  |  |  |  |  |  |  |  |  |  |  |
|  |  |  |  | Upadacitinib 30 mg QD | 168 | No relevant content |  |  |  |  |  |  |  |  |  |  |  |  |  |  |
| 18 | Theravance Biopharma 2023 | NCT03635112 | Induction | Placebo QD | 38 |  |  |  |  |  |  |  |  |  |  |  | 0 |  | 2 (5.3) | 36 (94.7) |
|  |  |  |  | Izencitinib 80 mg QD | 58 |  |  |  |  |  |  |  |  |  |  |  | 0 |  | 1 (1.7) | 57 (98.3) |
|  |  |  |  | Izencitinib 200 mg QD | 63 |  |  |  |  |  |  |  |  |  |  |  | 3 (4.8) |  | 0 | 60 (95.2) |
| 19 | Silvio Danese 2022 | NCT03653026 | Induction | Placebo QD | 177 | 127 (71.8) |  | 41 (23.2) |  | 6 (3.4) | 1 (0.6) | 1 (0.6) | 1 (0.6) |  |  |  |  |  |  |  |
|  |  |  |  | Upadacitinib 45 mg QD | 345 | 238 (69.0) |  | 94 (27.2) |  | 11 (3.2) | 0 | 0 | 2 (0.6) |  |  |  |  |  |  |  |
| 20 | Baili Chen 2022 | NCT03675477 | Induction | Placebo | 41 | 17 (41.5) |  | 24 (58.5) |  |  |  |  |  |  |  |  |  |  |  |  |
|  |  |  |  | Ivarmacitinib 4 mg QD | 41 | 16 (39.0) |  | 25 (61.0) |  |  |  |  |  |  |  |  |  |  |  |  |
|  |  |  |  | Ivarmacitinib 4 mg BID | 41 | 16 (39.0) |  | 25 (61.0) |  |  |  |  |  |  |  |  |  |  |  |  |
|  |  |  |  | Ivarmacitinib 8 mg QD | 41 | 12 (29.3) |  | 29 (70.7) |  |  |  |  |  |  |  |  |  |  |  |  |
| 21 | Theravance Biopharma 2021 | NCT03758443 | Induction | Placebo QD | 61 | 48 (78.7) |  | 9 (14.8) | 3 (4.9) | 1 (1.6) |  |  |  |  |  |  |  |  |  |  |
|  |  |  |  | Izencitinib 20 mg QD | 61 | 50 (82.0) |  | 11 (18.0) | 0 | 0 |  |  |  |  |  |  |  |  |  |  |
|  |  |  |  | Izencitinib 80 mg QD | 59 | 51 (86.4) |  | 6 (10.2) | 2 (3.3) | 0 |  |  |  |  |  |  |  |  |  |  |
|  |  |  |  | Izencitinib 200 mg QD | 58 | 51 (87.9) |  | 6 (10.3) | 1 (1.7) | 0 |  |  |  |  |  |  |  |  |  |  |
| 22 | S. Danese 2024 | NCT03934216 | Induction | Placebo QD | 43 | 34 (79.1) |  | 7 |  | 2 |  |  |  |  |  |  |  |  |  |  |
|  |  |  |  | Deucravacitinib 6mg BID | 88 | 80 (90.9) |  | 3 |  | 5 |  |  |  |  |  |  |  |  |  |  |
| 23 | Séverine Vermeire 2016 | NCT02048618 | Induction | Placebo QD | 44 | No relevant content |  |  |  |  |  |  |  |  |  |  |  |  |  |  |
|  |  |  |  | Filgotinib 200 mg QD | 130 | No relevant content |  |  |  |  |  |  |  |  |  |  |  |  |  |  |
| 24 | Bruce E. Sands 2019 | NCT01959282 | Induction | Placebo | 43 |  | 1 (2.3) | 1 (2.3) | 1 (2.3) |  |  |  |  |  | 40 (93.0) |  |  |  |  |  |
|  |  |  |  | Peficitinib 25 mg QD | 44 |  | 0 | 0 | 1 (2.3) |  |  |  |  |  | 43 (97.7) |  |  |  |  |  |
|  |  |  |  | Peficitinib 75 mg QD | 44 |  | 0 | 0 | 2 (4.5) |  |  |  |  |  | 42 (95.5) |  |  |  |  |  |
|  |  |  |  | Peficitinib 150 mg QD | 44 |  | 0 | 0 | 0 |  |  |  |  |  | 44 (100.0) |  |  |  |  |  |
|  |  |  |  | Peficitinib 75 mg BID | 44 |  | 3 (6.8) | 0 | 0 |  |  |  |  |  | 41 (93.2) |  |  |  |  |  |
| 25 | Pfizer 2009 | EudraCT: 2008-003571-45 | Induction | Placebo BID | 34 | 32 (94.1) | 2 (5.9) | 0 | 0 |  |  |  |  |  |  |  |  |  |  |  |
|  |  |  |  | Tofacitinib 1 mg BID | 36 | 34 (94.4) | 0 | 0 | 2 (5.6) |  |  |  |  |  |  |  |  |  |  |  |
|  |  |  |  | Tofacitinib 5 mg BID | 34 | 28 (82.4) | 5 (14.7) | 0 | 1 (2.9) |  |  |  |  |  |  |  |  |  |  |  |
|  |  |  |  | Tofacitinib 15 mg BID | 35 | 31 (88.6) | 2 (5.7) | 1 (2.9) | 1 (2.9) |  |  |  |  |  |  |  |  |  |  |  |
| 26 | Pfizer 2010 | EudraCT: 2008-004564-40 | Induction | Placebo BID | 49 | 43 (87.8) | 1 (2.0) |  | 5 (10.2) |  |  |  |  |  |  |  |  |  |  |  |
|  |  |  |  | Tofacitinib 0.5 mg BID | 31 | 28 (90.3) | 0 |  | 3 (9.7) |  |  |  |  |  |  |  |  |  |  |  |
|  |  |  |  | Tofacitinib 3 mg BID | 33 | 30 (90.9) | 1 (3.0) |  | 2 (6.1) |  |  |  |  |  |  |  |  |  |  |  |
|  |  |  |  | Tofacitinib 10 mg BID | 33 | 30 (90.9) | 0 () |  | 3 (9.1) |  |  |  |  |  |  |  |  |  |  |  |
|  |  |  |  | Tofacitinib 15 mg BID | 49 | 45 (91.8) | 1 (2.0) |  | 3 (6.1) |  |  |  |  |  |  |  |  |  |  |  |
