# Supplementary material for: Cardiovascular safety of Janus kinase inhibitors in inflammatory bowel disease: a systematic review and network meta-analysis
Source: Ann Med. 2025 Jan 21;57(1):2455536. doi: 10.1080/07853890.2025.2455536 (PMC11755742; doi:10.1080/07853890.2025.2455536)
Supplement: Supplemental Material [file IANN_A_2455536_SM7544.zip › suppl_data/Annex 2.docx]

**Table 1.** **Key events regarding the cardiovascular risks related to Baricitinib reported by the European Medicines Agency (EMA) and the United States Food and Drug Administration (FDA).**

| **Number** | **Title/Link** | **Data** | **Main content** | **Relevant research** |
| --- | --- | --- | --- | --- |
| Key events regarding the cardiovascular risks related to **Baricitinib** reported by the **European Medicines Agency (EMA)** | | | | |
| **1** | **New oral treatment for rheumatoid arthritis**  <https://www.ema.europa.eu/en/news/new-oral-treatment-rheumatoid-arthritis> | 2016.12.16 | The European Medicines Agency (EMA) has recommended granting a marketing authorisation in the European Union (EU) for Olumiant (baricitinib) for the treatment of adults with moderate to severe active rheumatoid arthritis. It is to be used to treat patients who have not responded adequately to, or who are unable to tolerate one or more disease-modifying anti-rheumatic drugs (DMARDs). Olumiant, which is taken by mouth, can be used on its own or in combination with methotrexate. | Four randomised controlled trials in 3,100 adults with moderate to severe active rheumatoid arthritis. One trial compared Olumiant to methotrexate, another compared Olumiant to adalimumab, and two compared Olumiant to placebo. Overall, Olumiant was more effective at reducing disease activity in patients with moderate to severe rheumatoid arthritis, compared to those treated with methotrexate and adalimumab. |
| **2** | **European Commission** **Approves Once-Daily Olumiant Tablets for Treatment of Adults with Moderate-to-Severe Active Rheumatoid Arthritis** (not the official report from the EMA)  <https://investor.lilly.com/news-releases/news-release-details/european-commission-approves-once-daily-olumiant-tablets>  <https://www.prnewswire.com/news-releases/european-commission-approves-once-daily-olumiant-tablets-for-treatment-of-adults-with-moderate-to-severe-active-rheumatoid-arthritis-300406478.html>  <https://go.drugbank.com/articles/A248395> | 2017.2.13 | INDIANAPOLIS, Feb. 13, 2017 /CNW/ -- Eli Lilly and Company (NYSE: LLY) and Incyte Corporation (NASDAQ: INCY) announced today that the European Commission has granted marketing authorisation for Olumiant^®^ (baricitinib) 4 mg and 2 mg film-coated tablets in Europe for the treatment of moderate-to-severe active rheumatoid arthritis (RA) in adult patients who have responded inadequately to, or who are intolerant to, one or more disease-modifying antirheumatic drugs (DMARDs).  This is the first regulatory approval around the world for Olumiant -- the first JAK inhibitor approved to treat RA in the European Union -- which may be used as monotherapy or in combination with methotrexate. In clinical studies, Olumiant has demonstrated significant improvement in the signs and symptoms of RA compared to standard of care therapies such as methotrexate and Humira^®^* (adalimumab) with background methotrexate. | Baricitinib's phase 3 program includes four completed clinical studies in a wide range of adult patients with RA, from treatment-naïve patients to those who are inadequate responders to TNF inhibitors. Two of those studies, RA-BEGIN and RA-BEAM, included a pre-specified comparison against either methotrexate or Humira with background methotrexate. Patients completing any of the phase 3 studies could enroll in a long-term extension study. |
| **3** | **Olumiant (authorisation for use in the European Union)**  <https://www.ema.europa.eu/en/medicines/human/EPAR/olumiant>  <https://www.ema.europa.eu/en/documents/overview/olumiant-epar-medicine-overview_en.pdf> | 2017.3.16 | - | - |
| **4** | **Scientific conclusions and grounds for the variation to the terms of the marketing authorisation(s) for baricitinib**  <https://www.ema.europa.eu/en/documents/scientific-conclusion/olumiant-psusa-00010578-201902-epar-scientific-conclusions-and-grounds-variation-terms-marketing-authorisation_en.pdf> | 2019.9.19 | The PRAC requests the MAH to update section 4.4 of the SmPC to modify the wording on “Venous Thromboembolism” to indicate that if clinical features of DVT/PE occur, baricitinib treatment should be discontinued and to update section 4.8 of the SmPC to add the adverse reaction pulmonary embolism and deep vein thrombosis with a frequency not known. | An imbalance in reports of VTE with 0% (EAIR of 0.0) for placebo vs. 0.53% (EAIR of 1.3) for baricitinib 4-mg was noted in placebo-controlled trials. VTE reporting rate is increasing. In July 2019 VTE reporting rate was 0.13% compared to 0.09% in February 2019. As of 01 July 2019, 114 VTEs have been reported in 102 cases from post-marketing reports. Moreover, recurrent DVT and subsequent PE observed September 2018 in patient who continued treatment with baricitinib after the first DVT also confirm possible relationship between use of baricitinib and occurrence of VTE. |
| Key events regarding the cardiovascular risks related to **Baricitinib** reported by the **United States Food and Drug Administration (FDA)** | | | | |
| **5** | **FDA stuns Lilly and Incyte with CRL for much-anticipated RA drug baricitinib** (not the official report from the FDA)  <https://www.fiercepharma.com/regulatory/fda-stuns-lilly-and-incyte-crl-for-much-anticipated-ra-drug-baricitinib>  <https://investor.lilly.com/news-releases/news-release-details/us-fda-issues-complete-response-letter-baricitinib>  <https://investor.lilly.com/news-releases/news-release-details/lilly-and-incyte-provide-update-baricitinib?releaseid=1034247> | 2017.4.14-2017.7.25 | INDIANAPOLIS--(BUSINESS WIRE)-- Eli Lilly and Company (NYSE:LLY) and Incyte Corporation (NASDAQ:INCY) announced today that the U.S. Food and Drug Administration (FDA) has issued a complete response letter for the New Drug Application (NDA) of the investigational medicine baricitinib, a once-daily oral medication for the treatment of moderate-to-severe rheumatoid arthritis (RA).  The letter indicates that the FDA is unable to approve the application in its current form. Specifically, the FDA indicated that additional clinical data are needed to determine the most appropriate doses. The FDA also stated that additional data are necessary to further characterize safety concerns across treatment arms. The companies disagree with the Agency's conclusions. The timing of a resubmission will be based on further discussions with the FDA. | The NDA for RA contained the results of four positive Phase 3 clinical trials that met their primary endpoints and in which 3,100 patients were enrolled, across the full spectrum of RA patients from treatment-naïve to highly-treatment refractory. Thromboembolic events - diagnosed as deep venous thrombosis (DVT) and pulmonary embolism (PE) - were reported in five patients receiving baricitinib during the controlled period of two of seven completed Phase 2 or Phase 3 trials in RA. Although an imbalance was observed during the placebo controlled period of the RA clinical trials, the rate of these events in the overall baricitinib clinical program was consistent with that seen among the general population of treated RA patients. |
| **6** | **FDA Advisory Committee Recommends the Approval of Baricitinib 2mg, but not 4mg, for the Treatment of Moderately-to-Severely Active Rheumatoid Arthritis** (not the official report from the FDA)  <https://investor.lilly.com/news-releases/news-release-details/fda-advisory-committee-recommends-approval-baricitinib-2mg-not>  <https://investor.incyte.com/news-releases/news-release-details/fda-advisory-committee-recommends-approval-baricitinib-2mg-not>  <https://www.prnewswire.com/news-releases/fda-advisory-committee-recommends-the-approval-of-baricitinib-2mg-but-not-4mg-for-the-treatment-of-moderately-to-severely-active-rheumatoid-arthritis-300634959.html>  <https://www.biopharmadive.com/news/lilly-baricitinib-fda-adcomm-dosing-safety-recommendation/522014/> | 2018.8.23 | INDIANAPOLIS, April 23, 2018 /PRNewswire/ -- Eli Lilly and Company (NYSE: LLY) and Incyte Corporation (NASDAQ: INCY) announced today that the U.S. Food and Drug Administration's (FDA) Arthritis Advisory Committee recommended approval of the 2-mg dose of baricitinib, a once-daily oral medication for the treatment of moderately-to-severely active rheumatoid arthritis (RA) for adult patients who have had an inadequate response or intolerance to methotrexate. While the Advisory Committee unanimously supported the efficacy of the 4-mg dose of baricitinib, it did not recommend approval of the 4-mg dose of baricitinib for the proposed indication based on the adequacy of the safety and benefit-risk profiles.  For both doses, the Advisory Committee voted to support the assessment that baricitinib's data provide substantial evidence of efficacy. For the 2-mg dose, the Advisory Committee voted in favor of the assessment that baricitinib's safety data adequately support its approval. For the 4-mg dose, the Advisory Committee voted against the assessment that baricitinib's safety data was adequate to support its approval based on the proposed indication. | The Advisory Committee's recommendation was based on baricitinib's global development program, which included four completed Phase 3 studies. In total, 3,492 patients, who represented a range of treatment experiences, received baricitinib in the global RA development program. The Phase 3 studies evaluated baricitinib's treatment impact related to RA signs and symptoms, physical function, joint damage progression and other patient-reported outcomes. The Phase 3 program also evaluated recognized risks for RA patients, including serious infection, malignancy, major adverse cardiovascular events (MACE), venous thromboembolism (VTE), and gastrointestinal perforations, along with key laboratory changes. The safety profile of baricitinib is based on 7,860 patient-years of exposure.  Blood clots were key to the rebuff of the 4 mg dose. Across four pivotal late-stage studies, there was a rate of seven thrombotic events per 100 patient years of exposure, whereas the placebo and 2 mg groups had rates of one and two events, respectively. Committee members did point out, however, that the data pool was smaller for the 2 mg dose, which Lilly investigated in just two Phase 3 studies. |
| **7** | **Approval Package and prescribing information for Olumiant (baricitinib)**  <https://www.accessdata.fda.gov/drugsatfda_docs/nda/2018/207924Orig1s000Approv.pdf>  <https://www.accessdata.fda.gov/drugsatfda_docs/label/2018/207924s000lbl.pdf> | 2018 | - | - |

**Table 2. Key events regarding the cardiovascular risks related to Tofacitinib reported by the European Medicines Agency (EMA) and the United States Food and Drug Administration (FDA).**

| **Number** | **Title/Link** | **Data** | **Main content** | **Relevant research** |
| --- | --- | --- | --- | --- |
| Key events regarding the cardiovascular risks related to **Tofacitinib** reported by the **European Medicines Agency (EMA)** | | | | |
| **1** | **Meeting highlights from the Committee for Medicinal Products for Human Use (CHMP) 22-25 April 2013**  <https://www.ema.europa.eu/en/news/meeting-highlights-committee-medicinal-products-human-use-chmp-22-25-april-2013>  <https://www.ema.europa.eu/en/documents/smop-initial/questions-and-answers-refusal-marketing-authorisation-xeljanz_en.pdf>  <https://www.ema.europa.eu/en/documents/smop-initial/questions-and-answers-refusal-marketing-authorisation-xeljanz-outcome-re-examination_en.pdf> | 2013.4.25-2013.7.25 | Negative recommendations on new medicines: Xeljanz, tofacitinib citrate  On 25 April 2013, the Committee for Medicinal Products for Human Use (CHMP) adopted a negative opinion, recommending the refusal of the marketing authorisation for the medicinal product Xeljanz, intended for the treatment of rheumatoid arthritis.  The company that applied for authorisation is Pfizer Limited. It may request a re-examination of the opinion within 15 days of receipt of notification of this negative opinion.  The applicant requested a re-examination of the opinion. After considering the grounds for this request, the CHMP re-examined the initial opinion, and confirmed the refusal of the marketing authorisation on 25 July 2013. | The Committee considered that, taken together, the data from the five main studies showed that treatment with Xeljanz resulted in an improvement in the signs and symptoms of rheumatoid arthritis and the physical function of patients. However, the studies were not sufficient to show a consistent reduction in disease activity and structural damage to joints, particularly at the lower 5-mg dose of Xeljanz and in the target population of patients in whom treatment with at least two other DMARDs has been unsuccessful.  The CHMP had major concerns about the overall safety profile of Xeljanz. There were significant and unresolved concerns about the risk and type of serious infections seen with tofacitinib, which are related to the immunosuppressant action of the medicine.  These safety concerns also included an increased risk of other severe side effects including certain cancers, gastro-intestinal perforations (holes in the wall of the gut), liver damage and problems with increased lipid (fat) levels in the blood. It was not clear that these risks could be managed successfully in medical practice. Therefore, at that point in time, the CHMP was of the opinion that the benefits of Xeljanz did not outweigh its risks and recommended that it be refused marketing authorisation. |
| **2** | **Pfizer Announces European Medicines Agency Accepted for Review Its Marketing Authorization Application for XELJANZ® (Tofacitinib Citrate) for the Treatment of Moderate to Severe Rheumatoid Arthritis** (not the official report from the EMA)  <https://www.pfizer.com/news/press-release/press-release-detail/pfizer_announces_european_medicines_agency_accepted_for_review_its_marketing_authorization_application_for_xeljanz_tofacitinib_citrate_for_the_treatment_of_moderate_to_severe_rheumatoid_arthritis>  <https://www.europeanpharmaceuticalreview.com/news/39977/ema-xeljanz-arthritis/> | 2016.3.23 | Pfizer Inc. (NYSE:PFE) announced today that the European Medicines Agency (EMA) has accepted for review the Marketing Authorization Application (MAA) for XELJANZ® (tofacitinib citrate) 5 mg tablets twice daily for the treatment of patients with moderate to severe rheumatoid arthritis (RA) who have had an inadequate response or intolerance to methotrexate (MTX). The EMA will now initiate its review of the XELJANZ MAA. | This application provides additional information to the original MAA submission, including data from the Phase 3 ORAL (Oral Rheumatoid Arthritis Phase 3 TriaLs) global development program in RA. This program consisted of six completed clinical trials, in addition to two open-label long-term extension (LTE) studies, one of which is still ongoing. To date, the ORAL development program has accumulated more than 19,400 patient-years of drug exposure having been studied in more than 6,100 patients including follow-up observations of up to eight years in the LTE study. |
| **3** | **Increased risk of blood clots in lungs and death with higher dose of Xeljanz (tofacitinib) for rheumatoid arthritis**  <https://www.ema.europa.eu/en/news/increased-risk-blood-clots-lungs-and-death-higher-dose-xeljanz-tofacitinib-rheumatoid-arthritis> | 2019.3.20 | EMA is advising healthcare professionals and patients not to exceed the recommended dose of Xeljanz (tofacitinib) when treating rheumatoid arthritis. The advice follows early results from an ongoing study (study A3921133) in patients with rheumatoid arthritis which showed an increased risk of blood clots in the lungs and death when the normal dose of 5 mg twice daily was doubled.  In the EU, 5 mg twice daily is the authorised dose for rheumatoid arthritis and psoriatic arthritis. The higher dose of 10 mg twice daily is approved for the initial treatment of patients with ulcerative colitis. | EMA is assessing the early results and will consider if any regulatory action is needed. In the meantime, patients with rheumatoid arthritis who are receiving Xeljanz at 10 mg twice daily in study A3921133 will have their dose reduced to 5 mg twice daily for the remaining duration of the study.  The aim of the study was to look at the risks of heart and circulatory problems with Xeljanz in patients 50 years of age or older who were already at higher risk of these, and to compare its safety with that of another medicine called a TNF inhibitor. |
| **4** | **Restrictions in use of Xeljanz while EMA reviews risk of blood clots in lungs**  <https://www.ema.europa.eu/en/news/restrictions-use-xeljanz-while-ema-reviews-risk-blood-clots-lungs> | 2019.5.17 | The new advice means that, since 10 mg is the only recommended starting dose for ulcerative colitis, patients with this condition who are at high risk of blood clots must not be started on Xeljanz. Patients at high risk currently taking this dose for any condition must be switched to alternative treatments. | Study A3921133: This study showed an increased risk of blood clots in the lungs and death when the 10 mg twice daily dose was used, which is double the recommended dose for rheumatoid arthritis. |
| **5** | **Xeljanz to be used with caution for all patients at high risk of blood clots**  <https://www.ema.europa.eu/en/news/meeting-highlights-pharmacovigilance-risk-assessment-committee-prac-28-31-october-2019>  <https://www.ema.europa.eu/en/documents/referral/xeljanz-article-20-procedure-xeljanz-be-used-caution-all-patients-high-risk-blood-clots_en.pdf> | 2019.10.31 | The PRAC recommended that Xeljanz should be used with caution in patients at high risk of blood clots. In addition, the maintenance doses of 10 mg twice daily should not be used in patients with ulcerative colitis who are at high risk unless there is no suitable alternative treatment. Patients older than 65 years of age should be treated with Xeljanz only when there is no other appropriate treatment. | An ongoing study in patients with rheumatoid arthritis found an increased risk of cardiovascular disease. The study showed an increased risk of blood clots in deep veins and in the lungs with both the 5 mg and 10 mg twice daily doses of Xeljanz as compared with patients taking TNF-inhibitors |
| **6** | **EMA confirms Xeljanz to be used with caution in patients at high risk of blood clots**  <https://www.ema.europa.eu/en/news/ema-confirms-xeljanz-be-used-caution-patients-high-risk-blood-clots> | 2019.11.15 | Xeljanz should be used with caution in all patients at high risk of blood clots. In addition, the maintenance doses of 10 mg twice daily should not be used in patients with ulcerative colitis who are at high risk of blood clots unless there is no suitable alternative treatment. | An ongoing study (study A3921133) in patients with rheumatoid arthritis found an increased risk of cardiovascular disease, plus data from earlier studies and consultation with experts in the field. All data combined showed that the risk of blood clots in deep veins and lungs was higher in patients taking Xeljanz, especially the 10 mg twice daily dose, and in those being treated for an extended period. |
| **7** | **Xeljanz (tofacitinib): increased risk of major adverse cardiovascular events and malignancies with use of tofacitinib relative to TNF-alpha inhibitors**  <https://www.ema.europa.eu/en/medicines/dhpc/xeljanz-0>  <https://www.ema.europa.eu/en/documents/dhpc/direct-healthcare-professional-communication-dhpc-xeljanz-tofacitinib-increased-risk-major-adverse-cardiovascular-events-and-malignancies-use-tofacitinib-relative-tnf-alpha-inhibitors_en.pdf> | 2021.6.7 | Tofacitinib should only be used in patients over 65 years of age, in patients who are current or past smokers, patients with other cardiovascular risk factors, and patients with other malignancy risk factors if no suitable treatment alternatives are available. | In the completed clinical trial (A3921133) in patients with rheumatoid arthritis (RA) who were 50 years of age or older with at least one additional cardiovascular risk factor, an increased incidence of myocardial infarction was observed with tofacitinib compared to TNF alpha inhibitors, despite no statistically significance |
| **8** | **EMA recommends measures to minimise risk of serious side effects with Janus kinase inhibitors for chronic inflammatory disorders**  <https://www.ema.europa.eu/en/news/meeting-highlights-pharmacovigilance-risk-assessment-committee-prac-24-27-october-2022> | 2022.10.28 | The Committee recommended that these medicines should be used in the following patients only if no suitable treatment alternatives are available: those aged 65 years or above, those at increased risk of major cardiovascular problems (such as heart attack or stroke), those who smoke or have done so for a long time in the past and those at increased risk of cancer.  The Committee also recommended using JAK inhibitors with caution in patients with risk factors for blood clots in the lungs and in deep veins (venous thromboembolism, VTE) other than those listed above. Further, the doses should be reduced in some patient groups who may be at risk of VTE, cancer or major cardiovascular problems.  The review confirmed Xeljanz increases the risk of major cardiovascular problems, cancer, VTE, serious infections and death due to any cause when compared with TNF-alpha inhibitors. The PRAC has now concluded that these safety findings apply to all approved uses of JAK inhibitors in chronic inflammatory disorders (rheumatoid arthritis, psoriatic arthritis, juvenile idiopathic arthritis, axial spondyloarthritis, ulcerative colitis, atopic dermatitis and alopecia areata). | The recommendations follow a review of available data, including the final results from a clinical trial1 of the JAK inhibitor Xeljanz (tofacitinib) and preliminary findings from an observational study involving Olumiant (baricitinib), another JAK inhibitor. During the review, the PRAC sought advice from an expert group of rheumatologists, dermatologists, gastroenterologists and patient representatives. |
| **9** | **EMA confirms measures** **to minimise risk of serious side effects with Janus kinase inhibitors for chronic inflammatory disorders**  <https://www.ema.europa.eu/en/medicines/human/referrals/janus-kinase-inhibitors-jaki>  <https://www.ema.europa.eu/en/documents/referral/janus-kinase-inhibitors-jaki-article-20-procedure-ema-confirms-measures-minimise-risk-serious-side-effects-janus-kinase-inhibitors-chronic-inflammatory-disorders_en.pdf-0> | 2023.1.23 | These medicines should be used in the following patients only if no suitable treatment alternatives are available: those aged 65 years or above, those at increased risk of major cardiovascular problems (such as heart attack or stroke), those who smoke or have done so for a long time in the past and those at increased risk of cancer.  JAK inhibitors should be used with caution in patients with risk factors for blood clots in the lungs and in deep veins (venous thromboembolism, VTE) other than those listed above. Further, the doses should be reduced in patient groups who are at risk of VTE, cancer or major cardiovascular problems, where possible.  The review confirmed Xeljanz increases the risk of major cardiovascular problems, cancer, VTE, serious infections and death due to any cause when compared with medicines belonging to the class of TNF-alpha inhibitors. EMA has now concluded that these safety findings apply to all approved uses of JAK inhibitors in chronic inflammatory disorders (rheumatoid arthritis, psoriatic arthritis, juvenile idiopathic arthritis, axial spondyloarthritis, ulcerative colitis, atopic dermatitis and alopecia areata). | The recommendations follow a review of available data, including the final results from a clinical trial1 of the JAK inhibitor Xeljanz (tofacitinib) and preliminary findings from an observational study involving Olumiant. The review also included advice from an expert group of rheumatologists, dermatologists, gastroenterologists and patient representatives. |
| **10** | **List of medicinal products under additional monitoring [filgotinib]**  <https://www.ema.europa.eu/en/documents/additional-monitoring/list-medicinal-products-under-additional-monitoring_en.pdf>  <https://www.ema.europa.eu/en/documents/product-information/jyseleca-epar-product-information_en.pdf> | 2020.8.24 | Major adverse cardiovascular events and venous thromboembolic events are considered significant potential risks on all JAK inhibitors risk management plans, and all these medicines from this class are under additional safety monitoring on these adverse events | Events of MACE have been observed in patients taking filgotinib. In a large randomised active-controlled study of tofacitinib (another JAK inhibitor) in rheumatoid arthritis patients 50 years and older with at least one additional cardiovascular risk factor, a higher rate of major adverse cardiovascular events (MACE), defined as cardiovascular death, non-fatal myocardial infarction (MI) and non-fatal stroke, was observed with tofacitinib compared to TNF inhibitors. Therefore, in patients 65 years of age and older, patients who are current or past long-time smokers, and patients with history of atherosclerotic cardiovascular disease or other cardiovascular risk factors, filgotinib should only be used if no suitable treatment alternatives are available.  Events of deep venous thrombosis (DVT) and pulmonary embolism (PE) have been reported in patients receiving JAK inhibitors including filgotinib. In a large randomised active-controlled study of tofacitinib (another JAK inhibitor) in rheumatoid arthritis patients 50 years and older with at least one additional cardiovascular risk factor, a dose dependent higher rate of VTE including deep venous thrombosis (DVT) and pulmonary embolism (PE) was observed with tofacitinib compared to TNF inhibitors. In patients with cardiovascular or malignancy risk factors (see also section 4.4 “Major adverse cardiovascular events (MACE)” and “Malignancy”) filgotinib should only be used if no suitable treatment alternatives are available. In patients with known VTE risk factors other than cardiovascular or malignancy risk factors, filgotinib should be used with caution. VTE risk factors other than cardiovascular or malignancy risk factors include previous VTE, patients undergoing major surgery, immobilisation, use of combined hormonal contraceptives or hormone replacement therapy, inherited coagulation disorder. |
| Key events regarding the cardiovascular risks related to **Tofacitinib** reported by the **United States Food and Drug Administration (FDA)** | | | | |
| **11** | **U.S. Food And Drug Administration Approves Pfizer’s XELJANZ® (tofacitinib citrate) for Adults with Moderately to Severely Active Rheumatoid Arthritis (RA) Who Have Had an Inadequate Response or Intolerance to Methotrexate**  <https://www.accessdata.fda.gov/drugsatfda_docs/nda/2012/203214Orig1s000TOC.cfm>  <https://www.pfizer.com/news/press-release/press-release-detail/us-food-and-drug-administration-approves-pfizers-xeljanzr>  <https://www.pharmacytimes.com/view/fda-approves-xeljanz-for-rheumatoid-arthritis> | 2012.11.6 | NEW YORK--([BUSINESS WIRE](http://www.businesswire.com/))--Pfizer Inc. (NYSE: PFE) announced today that the U.S. Food and Drug Administration (FDA) has approved XELJANZ^®^ (tofacitinib citrate) 5 mg twice daily for the treatment of adults with moderately to severely active rheumatoid arthritis (RA) who have had an inadequate response or intolerance to methotrexate. XELJANZ may be used as monotherapy or in combination with methotrexate or other non-biologic disease-modifying antirheumatic drugs (DMARDs). XELJANZ should not be used in combination with biologic DMARDs or with potent immunosuppressives, such as azathioprine and cyclosporine. | In the clinical trials, XELJANZ was studied in both a 5 mg and 10 mg twice-daily dosing regimen. The FDA has approved the 5 mg twice-daily dose in the second-line setting and has indicated that further data are required to assess the benefit: risk profile of the 10 mg twice-daily dose. Pfizer will continue to generate additional clinical data on the 10 mg twice-daily dose and work with the FDA to understand the additional data needed for further assessment of the 10 mg twice-daily dose.  Safety findings observed in the overall XELJANZ RA program include serious and other important infections, including tuberculosis and herpes zoster; malignancies, including lymphoma; gastrointestinal perforations; decreased neutrophil and lymphocyte counts; decreased hemoglobin; liver enzyme elevations; and lipid elevations. |
| **12** | **FDA approves new treatment for moderately to severely active ulcerative colitis**  <https://www.fda.gov/news-events/press-announcements/fda-approves-new-treatment-moderately-severely-active-ulcerative-colitis> | 2018.5.30 | The U.S. Food and Drug Administration today expanded the approval of Xeljanz (tofacitinib) to include adults with moderately to severely active ulcerative colitis. Xeljanz is the first oral medication approved for chronic use in this indication. Other FDA-approved treatments for the chronic treatment of moderately to severely active ulcerative colitis must be administered through an intravenous infusion or subcutaneous injection. | The efficacy of Xeljanz for the treatment of moderately to severely active ulcerative colitis was demonstrated in three controlled clinical trials. This included two 8-week placebo-controlled trials that demonstrated that 10 mg of Xeljanz given twice daily induces remission in 17 to 18 percent of patients by week eight. In a placebo-controlled trial among patients who achieved a clinical response by week eight, Xeljanz, at a 5 mg or 10 mg dose given twice daily, was effective in inducing remission by week 52 in 34 percent and 41 percent of patients, respectively. Among patients who achieved remission after 8 weeks of treatment, 35 percent and 47 percent achieved sustained corticosteroid-free remission when treated with 5 mg and 10 mg, respectively. |
| **13** | **Safety trial finds risk of blood clots in the lungs and death with higher dose of tofacitinib (Xeljanz, Xeljanz XR) in rheumatoid arthritis patients; FDA to investigate**  <https://www.fda.gov/drugs/drug-safety-and-availability/safety-trial-finds-risk-blood-clots-lungs-and-death-higher-dose-tofacitinib-xeljanz-xeljanz-xr> | 2019.2.25 | The U.S. Food and Drug Administration (FDA) is alerting the public that a safety clinical trial found an increased risk of blood clots in the lungs and death when a 10 mg twice daily dose of tofacitinib (Xeljanz, Xeljanz XR) was used in patients with rheumatoid arthritis (RA). FDA has not approved this 10 mg twice daily dose for RA; this dose is only approved in the dosing regimen for patients with ulcerative colitis. | When FDA first approved tofacitinib, we required a clinical trial among patients with RA to evaluate the risk of heart-related events, cancer, and opportunistic infections with the medicine at two doses (10 mg twice daily and 5 mg twice daily) in combination with methotrexate in comparison to another drug called a tumor necrosis factor (TNF) inhibitor. RA patients in the trial were required to be at least 50 years old and have at least one cardiovascular risk factor. During the most recent analysis of the trial, an external data safety monitoring committee found an increased occurrence of blood clots in the lungs and death in patients treated with tofacitinib 10 mg twice daily compared to patients treated with tofacitinib 5 mg twice daily or a TNF inhibitor. |
| **14** | **FDA approves Boxed Warning about increased risk of blood clots and death with higher dose of arthritis and ulcerative colitis medicine tofacitinib (Xeljanz, Xeljanz XR)**  <https://www.fda.gov/drugs/drug-safety-and-availability/fda-approves-boxed-warning-about-increased-risk-blood-clots-and-death-higher-dose-arthritis-and>  <https://www.fda.gov/media/129647/download?attachment>  <https://www.fda.gov/drugs/fda-drug-safety-podcasts/fda-approves-boxed-warning-about-increased-risk-blood-clots-and-death-higher-dose-arthritis-and>  <https://www.fda.gov/safety/medical-product-safety-information/xeljanz-xeljanz-xr-tofacitinib-drug-safety-communication-due-increased-risk-blood-clots-and-death> | 2019.7.26 | The U.S. Food and Drug Administration has approved new warnings about an increased risk of blood clots and of death with the 10 mg twice daily dose of tofacitinib (Xeljanz, Xeljanz XR), which is used in patients with ulcerative colitis. In addition, the approved use of tofacitinib for ulcerative colitis will be limited to certain patients who are not treated effectively or who experience severe side effects with certain other medicines. We approved these changes, including adding our most prominent Boxed Warning, after reviewing interim data from an ongoing safety clinical trial of tofacitinib in patients with rheumatoid arthritis (RA) that examined a lower and this higher dose of the medicine.  The 10 mg twice daily dose of tofacitinib is not approved for RA or psoriatic arthritis (PsA). This dose is only approved for ulcerative colitis for initial treatment and for long-term use in limited situations. While the increased risks of blood clots and of death were seen in patients taking this dose for RA, these risks may also apply to those taking tofacitinib for ulcerative colitis.  On July 26, 2019, FDA approved new warnings about an increased risk of blood clots and death with the 10 mg twice daily dose of tofacitinib (brand names Xeljanz, Xeljanz XR), used in patients with ulcerative colitis. The approved use of tofacitinib for ulcerative colitis will also be limited to patients who are not treated effectively or who experience severe side effects with certain other medicines. We approved these changes, adding our most prominent Boxed Warning, after reviewing interim data from an ongoing safety clinical trial of tofacitinib in patients with rheumatoid arthritis (RA) that examined a lower and this higher dose of the medicine. | The interim results of the trial, as of January 2019, have identified the following:  19 cases of blood clots in the lung out of 3,884 patient-years of follow-up in patients who received tofacitinib 10 mg twice daily, compared to 3 cases out of 3,982 patient-years in patients who received TNF blockers  45 cases of death from all causes out of 3,884 patient-years of follow-up in patients who received tofacitinib 10 mg twice daily, compared to 25 cases out of 3,982 patient-years in patients who received TNF blockers |
| **15** | **Initial safety trial results find increased risk of serious heart-related problems and cancer with arthritis and ulcerative colitis medicine Xeljanz, Xeljanz XR (tofacitinib)**  <https://www.fda.gov/drugs/drug-safety-and-availability/initial-safety-trial-results-find-increased-risk-serious-heart-related-problems-and-cancer-arthritis> | 2021.2.4 | The U.S. Food and Drug Administration (FDA) is alerting the public that preliminary results from a safety clinical trial show an increased risk of serious heart-related problems and cancer with the arthritis and ulcerative colitis medicine Xeljanz, Xeljanz XR (tofacitinib) compared to another type of medicine called tumor necrosis factor (TNF) inhibitors. FDA required the safety trial, which also investigated other potential risks including blood clots in the lungs and death. Those final results are not yet available. | When FDA first approved tofacitinib, we required the manufacturer, Pfizer, to conduct a safety clinical trial in patients with RA who were taking methotrexate to evaluate the risk of serious heart-related events, cancer, and infections. The trial studied two doses of tofacitinib (5 mg twice daily, which is the approved dosage for RA, and a higher 10 mg twice daily dosage) in comparison to another type of RA medicine called a TNF inhibitor. Patients in the trial were required to be at least 50 years old and have at least one cardiovascular risk factor. In February 2019 and July 2019, we warned that interim trial results showed an increased risk of blood clots and death with the higher 10 mg twice daily dosage, and as a result, approved a Boxed Warning to the tofacitinib prescribing information. The clinical trial is now complete and initial results show a higher occurrence of serious heart-related events and cancer in RA patients treated with both doses of tofacitinib compared to patients treated with a TNF inhibitor. FDA is awaiting additional results from the trial. |
| **16** | **FDA requires warnings about increased risk of serious heart-related events, cancer, blood clots, and death for JAK inhibitors that treat certain chronic inflammatory conditions**  <https://www.fda.gov/drugs/drug-safety-and-availability/fda-requires-warnings-about-increased-risk-serious-heart-related-events-cancer-blood-clots-and-death>  <https://www.fda.gov/drugs/fda-drug-safety-podcasts/fda-requires-warnings-about-increased-risk-serious-heart-related-events-cancer-blood-clots-and-death> | 2021.9.1 | We are requiring new and updated warnings for two other arthritis medicines in the same drug class as Xeljanz, called Janus kinase (JAK) inhibitors, Olumiant (baricitinib) and Rinvoq (Upadacitinib). Olumiant and Rinvoq have not been studied in trials similar to the large safety clinical trial with Xeljanz, so the risks have not been adequately evaluated. However, since they share mechanisms of action with Xeljanz, FDA considers that these medicines may have similar risks as seen in the Xeljanz safety trial.  Reserve these medicines for patients who have had an inadequate response or intolerance to one or more TNF blockers. | Based on a completed U.S. Food and Drug Administration (FDA) review of a large randomized safety clinical trial, we have concluded there is an increased risk of serious heart-related events such as heart attack or stroke, cancer, blood clots, and death with the arthritis and ulcerative colitis medicines Xeljanz and Xeljanz XR (tofacitinib). This trial compared Xeljanz with another type of medicine used to treat arthritis called tumor necrosis factor (TNF) blockers in patients with rheumatoid arthritis. The trial’s final results also showed an increased risk of blood clots and death with the lower dose of Xeljanz. A prior DSC based upon earlier results from this trial, reported an increased risk of blood clots and death only seen at the higher dose. |
