# Supplementary material for: Cardiovascular safety of Janus kinase inhibitors in inflammatory bowel disease: a systematic review and network meta-analysis
Source: Ann Med. 2025 Jan 21;57(1):2455536. doi: 10.1080/07853890.2025.2455536 (PMC11755742; doi:10.1080/07853890.2025.2455536)
Supplement: Supplemental Material [file IANN_A_2455536_SM7544.zip › suppl_data/Clean copy - Supplementary_table_changes (1).docx]

**Cardiovascular safety of Janus kinase inhibitors in inflammatory bowel disease: a systematic review and network meta-analysis**

**Huibin Yang, Ting An, Yuxuan Zhao, Xiaojing Shi, Bangmao Wang, Qingyu Zhang**

**Tianjin Medical University General Hospital**

**e-mail address: (Huibin Yang)** [**Bryan_yang323@163.com**](mailto:Bryan_yang323@163.com)**; (Qingyu Zhang)** [**zhangqy@tmu.edu.cn**](mailto:zhangqy@tmu.edu.cn)

**Contents**

**Supplementary table 1 The PRISMA checklist.3**

**Supplementary table 2 Search strategies of this network meta-analysis.8**

**Supplementary table 3 Basic information of included JAKinibs.16**

**Supplementary table 4 R codes in the network meta-analysis.17**

Supplementary table 4-1 R codes in the network meta-analysis (without dose consideration).17

Supplementary table 4-2 R codes in the network meta-analysis (with dose consideration).21

**Supplementary table 5 Baseline characteristics and outcomes details of included RCTs.28**

Supplementary table 5-1 Baseline characteristics of included RCTs.28

Supplementary table 5-2 Outcomes details of included RCTs.33

**Supplementary table 6 Summary of key characteristics of included RCTs.38**

**Supplementary table 7 The Risk-of-bias assessment for the direct evidence (without dose consideration).39**

**Supplementary table 8 Certainty of direct and indirect evidence assessment (without dose consideration).40**

Supplementary table 8-1 Certainty of direct evidence assessment (without dose consideration).40

Supplementary table 8-1-1 Certainty of direct evidence assessment for MACE (without dose consideration).40

Supplementary table 8-1-2 Certainty of direct evidence assessment for VTE (without dose consideration).41

Supplementary table 8-1-3 Certainty of direct evidence assessment for CVE (without dose consideration).42

Supplementary table 8-2 Certainty of indirect evidence assessment (without dose consideration).43

Supplementary table 8-2-1 Certainty of indirect evidence assessment for MACE (without dose consideration).43

Supplementary table 8-2-2 Certainty of indirect evidence assessment for VTE (without dose consideration).46

Supplementary table 8-2-3 Certainty of indirect evidence assessment for CVE (without dose consideration).50

**Supplementary table 9 The Risk-of-bias assessment for the direct evidence (with dose consideration).54**

**Supplementary table 10 Certainty of evidence assessment (with dose consideration).57**

Supplementary table 10-1 Certainty of direct evidence assessment (with dose consideration).57

Supplementary table 10-1-1 Certainty of direct evidence assessment for MACE (with dose consideration).57

Supplementary table 10-1-2 Certainty of direct evidence assessment for VTE (with dose consideration).60

Supplementary table 10-1-3 Certainty of direct evidence assessment for CVE (with dose consideration).63

Supplementary table 10-2 Certainty of indirect evidence assessment (with dose consideration).66

Supplementary table 10-2-1 Certainty of indirect evidence assessment for MACE (with dose consideration).66

Supplementary table 10-2-2 Certainty of indirect evidence assessment for VTE (with dose consideration).79

Supplementary table 10-2-3 Certainty of indirect evidence assessment for CVE (with dose consideration).92

Supplementary table 10-3 Certainty of network meta-analysis evidence assessment (with dose consideration).106

Supplementary table 10-3-1 Certainty of network meta-analysis evidence assessment for MACE (with dose consideration)..106

Supplementary table 10-3-2 Certainty of network meta-analysis evidence assessment for VTE (with dose consideration).109

Supplementary table 10-3-3 Certainty of network meta-analysis evidence assessment for CVE (with dose consideration).113

Supplementary table 10-4 Selection of final certainty rating (with dose consideration).117

Supplementary table 10-4-1 Selection of final certainty rating for MACE (with dose consideration).117

Supplementary table 10-4-2 Selection of final certainty rating for VTE (with dose consideration).119

Supplementary table 10-4-3 Selection of final certainty rating for CVE (with dose consideration).121

**Supplementary table 11 Direct, indirect, NMA results and certainty.125**

Supplementary table 11-1 Direct, indirect, NMA results and certainty (without dose consideration).125

Supplementary table 11-1-1 Direct, indirect, NMA results and certainty for MACE (without dose consideration).125

Supplementary table 11-1-2 Direct, indirect, NMA results and certainty for VTE (without dose consideration).126

Supplementary table 11-1-3 Direct, indirect, NMA results and certainty for CVE (without dose consideration).127

Supplementary table 11-2 Direct, indirect and NMA results and certainty (with dose consideration).128

Supplementary table 11-2-1 Direct, indirect, NMA results and certainty for MACE (with dose consideration).128

Supplementary table 11-2-2 Direct, indirect, NMA results and certainty for VTE (with dose consideration).131

Supplementary table 11-2-3 Direct, indirect, NMA results and certainty for CVE (with dose consideration).134

**Supplementary table 12 P-scores and SUCRA values.139**

Supplementary table 12-1 P-scores and SUCRA values for MACE.139

Supplementary table 12-2 P-scores and SUCRA values for VTE.140

Supplementary table 12-3 P-scores and SUCRA values for CTE.141

**Supplementary table 13 Tests of heterogeneity (within designs) and inconsistency (between designs) with Q statistics.142**

**Supplementary table 1 The PRISMA checklist**

| **Section and Topic** | **Item #** | **Checklist item** | **Location where item is reported** |
| --- | --- | --- | --- |
| **TITLE** | | |  |
| Title | 1 | Identify the report as a systematic review. | Lines 1-2 |
| **ABSTRACT** | | |  |
| Abstract | 2 | See the PRISMA 2020 for Abstracts checklist. | Lines 21-50 |
| **INTRODUCTION** | | |  |
| Rationale | 3 | Describe the rationale for the review in the context of existing knowledge. | Lines 62-98 |
| Objectives | 4 | Provide an explicit statement of the objective(s) or question(s) the review addresses. | Lines 100-106 |
| **METHODS** | | |  |
| Eligibility criteria | 5 | Specify the inclusion and exclusion criteria for the review and how studies were grouped for the syntheses. | Lines 127-141 |
| Information sources | 6 | Specify all databases, registers, websites, organisations, reference lists and other sources searched or consulted to identify studies. Specify the date when each source was last searched or consulted. | Lines 120-123 |
| Search strategy | 7 | Present the full search strategies for all databases, registers and websites, including any filters and limits used. | Supplementary table 2 |
| Selection process | 8 | Specify the methods used to decide whether a study met the inclusion criteria of the review, including how many reviewers screened each record and each report retrieved, whether they worked independently, and if applicable, details of automation tools used in the process. | Lines 109-127 |
| Data collection process | 9 | Specify the methods used to collect data from reports, including how many reviewers collected data from each report, whether they worked independently, any processes for obtaining or confirming data from study investigators, and if applicable, details of automation tools used in the process. | Lines 127-145 |
| Data items | 10a | List and define all outcomes for which data were sought. Specify whether all results that were compatible with each outcome domain in each study were sought (e.g. for all measures, time points, analyses), and if not, the methods used to decide which results to collect. | Lines 148-155, 167-168 |
|  | 10b | List and define all other variables for which data were sought (e.g. participant and intervention characteristics, funding sources). Describe any assumptions made about any missing or unclear information. | Lines 148-155 |
| Study risk of bias assessment | 11 | Specify the methods used to assess risk of bias in the included studies, including details of the tool(s) used, how many reviewers assessed each study and whether they worked independently, and if applicable, details of automation tools used in the process. | Lines 158-164 |
| Effect measures | 12 | Specify for each outcome the effect measure(s) (e.g. risk ratio, mean difference) used in the synthesis or presentation of results. | Lines 168-176 |
| Synthesis methods | 13a | Describe the processes used to decide which studies were eligible for each synthesis (e.g. tabulating the study intervention characteristics and comparing against the planned groups for each synthesis (item #5)). | Lines 127-141, 187-192, 225-229 |
|  | 13b | Describe any methods required to prepare the data for presentation or synthesis, such as handling of missing summary statistics, or data conversions. | Lines 148-155, 167-176, 187-192 |
|  | 13c | Describe any methods used to tabulate or visually display results of individual studies and syntheses. | Lines 187-238 |
|  | 13d | Describe any methods used to synthesize results and provide a rationale for the choice(s). If meta-analysis was performed, describe the model(s), method(s) to identify the presence and extent of statistical heterogeneity, and software package(s) used. | Lines 187-238 |
|  | 13e | Describe any methods used to explore possible causes of heterogeneity among study results (e.g. subgroup analysis, meta-regression). | Lines 204-210, 231-236 |
|  | 13f | Describe any sensitivity analyses conducted to assess robustness of the synthesized results. | Lines 230-231 |
| Reporting bias assessment | 14 | Describe any methods used to assess risk of bias due to missing results in a synthesis (arising from reporting biases). | Lines 237-238 |
| Certainty assessment | 15 | Describe any methods used to assess certainty (or confidence) in the body of evidence for an outcome. | Lines 179-184 |
| **RESULTS** | | |  |
| Study selection | 16a | Describe the results of the search and selection process, from the number of records identified in the search to the number of studies included in the review, ideally using a flow diagram. | Lines 241-247, Figure 1 |
|  | 16b | Cite studies that might appear to meet the inclusion criteria, but which were excluded, and explain why they were excluded. | Lines 244-246, Figure 1 |
| Study characteristics | 17 | Cite each included study and present its characteristics. | Lines 250-258, Supplementary table 5 |
| Risk of bias in studies | 18 | Present assessments of risk of bias for each included study. | Lines 273-278, Supplementary figures 3-4 |
| Results of individual studies | 19 | For all outcomes, present, for each study: (a) summary statistics for each group (where appropriate) and (b) an effect estimate and its precision (e.g. confidence/credible interval), ideally using structured tables or plots. | Lines 278-280, Supplementary figures 5-6 |
| Results of syntheses | 20a | For each synthesis, briefly summarise the characteristics and risk of bias among contributing studies. | Lines 258-270, Supplementary figures 1-2, tables 6-7 and 9 |
|  | 20b | Present results of all statistical syntheses conducted. If meta-analysis was done, present for each the summary estimate and its precision (e.g. confidence/credible interval) and measures of statistical heterogeneity. If comparing groups, describe the direction of the effect. | Lines 282-421, Figures 2-8, Supplementary figures 7-16, tables 7-13 |
|  | 20c | Present results of all investigations of possible causes of heterogeneity among study results. | Lines 417-421, 424-445, Supplementary figures 15-16 and 18-21, table 13 |
|  | 20d | Present results of all sensitivity analyses conducted to assess the robustness of the synthesized results. | Lines 424-445, Supplementary figure 17 |
| Reporting biases | 21 | Present assessments of risk of bias due to missing results (arising from reporting biases) for each synthesis assessed. | Lines 315-316, 337-338, 353-354, 374-376, 390-391, 412-414, Supplementary figures 13-14 |
| Certainty of evidence | 22 | Present assessments of certainty (or confidence) in the body of evidence for each outcome assessed. | Lines 310, 325-327, 346-347, 364-367, 384-385, 401-404, Figures 3-8, Supplementary tables 7-11 |
| **DISCUSSION** | | |  |
| Discussion | 23a | Provide a general interpretation of the results in the context of other evidence. | Lines 458-597 |
|  | 23b | Discuss any limitations of the evidence included in the review. | Lines 613-630 |
|  | 23c | Discuss any limitations of the review processes used. | Lines 630-644 |
|  | 23d | Discuss implications of the results for practice, policy, and future research. | Lines 503-505, 525-529, 547-556, 570-576, 595-597, 650-655 |
| **OTHER INFORMATION** | | |  |
| Registration and protocol | 24a | Provide registration information for the review, including register name and registration number, or state that the review was not registered. | Lines 110-114 |
|  | 24b | Indicate where the review protocol can be accessed, or state that a protocol was not prepared. | A protocol was prepared and the information provided at registration could be obtained from https://www.crd.york.ac.uk/PROSPEROFILES/524803_STRATEGY_20240315.pdf |
|  | 24c | Describe and explain any amendments to information provided at registration or in the protocol. | Main differences between the information provided at registration and this review: 1. Main outcome: We added cardiovascular events (defined as the sum of MACE and VTE) for a better understanding of the cardiovascular risks associated with the JAK inhibitors; 2. Measures of effect: We used odds ratio (OR) to replace the risk ratio (RR) because OR was more commonly used and easier to compare with other relevant studies; 3. Strategy for data synthesis: We used “netmeta” package to replace the “GeMTC” package because we could not make cumulative ranking plots with the “GeMTC” package, and these plots were vital for the evaluation of the cardiovascular risks associated with the JAK inhibitors. |
| Support | 25 | Describe sources of financial or non-financial support for the review, and the role of the funders or sponsors in the review. | Lines 675-676: The study was funded by Tianjin Key Medical Discipline (Specialty) Construction Project TJYXZDXK-002A. |
| Competing interests | 26 | Declare any competing interests of review authors. | Line 664: The authors declare no conflict of interest. |
| Availability of data, code and other materials | 27 | Report which of the following are publicly available and where they can be found: template data collection forms; data extracted from included studies; data used for all analyses; analytic code; any other materials used in the review. | All analyses processes and R codes can be obtained from Supplementary Data Content. Other relevant data can be obtained by e-mailing the corresponding author (Bryan_yang323@163.com) for valid reasons. |
| From: Page MJ, McKenzie JE, Bossuyt PM, et al. The PRISMA 2020 statement: an updated guideline for reporting systematic reviews. BMJ 2021;372:n71. doi:10.1136/bmj.n71 | | | |

**Supplementary table 2 Search strategies of this network meta-analysis.**

| Database or ClinicalTrials.gov | Search strategy |
| --- | --- |
| 1.PubMed: | #1: "Inflammatory Bowel Diseases"[Mesh]  #2: ("Inflammatory Bowel Disease"[Title/Abstract]) OR ("Bowel Diseases, Inflammatory"[Title/Abstract])  #3: #1 OR #2  #4: "Crohn Disease"[Mesh]  #5: ((((((((((((((("Crohn's Enteritis"[Title/Abstract]) OR ("Regional Enteritis"[Title/Abstract])) OR ("Crohn's Disease"[Title/Abstract])) OR ("Crohns Disease"[Title/Abstract])) OR ("Inflammatory Bowel Disease 1"[Title/Abstract])) OR ("Enteritis, Granulomatous"[Title/Abstract])) OR ("Granulomatous Enteritis"[Title/Abstract])) OR ("Enteritis, Regional"[Title/Abstract])) OR ("Ileocolitis"[Title/Abstract])) OR ("Colitis, Granulomatous"[Title/Abstract])) OR ("Granulomatous Colitis"[Title/Abstract])) OR ("Ileitis, Terminal"[Title/Abstract])) OR ("Terminal Ileitis"[Title/Abstract])) OR ("Ileitis, Regional"[Title/Abstract])) OR ("Regional Ileitides"[Title/Abstract])) OR ("Regional Ileitis"[Title/Abstract])  #6: #4 OR #5  #7: "Colitis, Ulcerative"[Mesh]  #8: ((("Idiopathic Proctocolitis"[Title/Abstract]) OR ("Ulcerative Colitis"[Title/Abstract])) OR ("Colitis Gravis"[Title/Abstract])) OR ("Inflammatory Bowel Disease, Ulcerative Colitis Type"[Title/Abstract])  #9: #7 OR #8  #10: #3 OR #6 OR #9  #11: "Janus Kinase Inhibitors"[Mesh]  #12: (((((((("Inhibitors, Janus Kinase"[Title/Abstract]) OR ("Kinase Inhibitors, Janus"[Title/Abstract])) OR ("JAK Inhibitors"[Title/Abstract])) OR ("Inhibitors, JAK"[Title/Abstract])) OR ("Janus Kinase Inhibitor"[Title/Abstract])) OR ("Inhibitor, Janus Kinase"[Title/Abstract])) OR ("Kinase Inhibitor, Janus"[Title/Abstract])) OR ("JAK Inhibitor"[Title/Abstract])) OR ("Inhibitor, JAK"[Title/Abstract])  #13: #11 OR #12  #14: "ruxolitinib" [Concept]  #15: (((((((((((((((("ruxolitinib"[Title/Abstract]) OR ("ruxolitinib phosphate"[Title/Abstract])) OR ("ruxolitinib monophosphate"[Title/Abstract])) OR ("INCB-18424 phosphate"[Title/Abstract])) OR ("Jakavi"[Title/Abstract])) OR ("INCB018424 phosphate"[Title/Abstract])) OR ("INCB-018424 salt"[Title/Abstract])) OR ("Jakafi"[Title/Abstract])) OR ("ruxolitinib as phosphate"[Title/Abstract])) OR ("INCB-018424 phosphate"[Title/Abstract])) OR ("opzelura"[Title/Abstract])) OR ("INCB-018424"[Title/Abstract])) OR ("INC-424"[Title/Abstract])) OR ("INCB-18424"[Title/Abstract])) OR ("INC424"[Title/Abstract])) OR ("INCB018424"[Title/Abstract])) OR ("INCA24"[Title/Abstract])  #16: #14 OR #15  #17: "tofacitinib" [Concept]  #18: (((((((("tofacitinib"[Title/Abstract]) OR ("tasocitinib"[Title/Abstract])) OR ("tofacitinib citrate"[Title/Abstract])) OR ("Xeljanz"[Title/Abstract])) OR ("CP 690,550"[Title/Abstract])) OR ("CP690550"[Title/Abstract])) OR ("CP-690550"[Title/Abstract])) OR ("CP 690550"[Title/Abstract])) OR ("CP-690,550"[Title/Abstract])  #19: #17 OR #18  #20: "oclacitinib" [Concept]  #21: ("oclacitinib"[Title/Abstract]) OR ("apoquel"[Title/Abstract])  #22: #20 OR #21  #23: "baricitinib" [Concept]  #24: (((((((("baricitinib"[Title/Abstract]) OR ("INCB-28050"[Title/Abstract])) OR ("Olumiant"[Title/Abstract])) OR ("baricitinib phosphate"[Title/Abstract])) OR ("baricitinib phosphate salt"[Title/Abstract])) OR ("INCB028050"[Title/Abstract])) OR ("INCB-028050"[Title/Abstract])) OR ("LY3009104"[Title/Abstract])) OR ("LY-3009104"[Title/Abstract])  #25: #23 OR #24  #26: "peficitinib" [Concept]  #27: ("peficitinib"[Title/Abstract]) OR ("ASP015K"[Title/Abstract])  #28: #26 OR #27  #29: "fedratinib" [Concept]  #30: (((((((((("fedratinib"[Title/Abstract]) OR ("Inrebic"[Title/Abstract])) OR ("SAR302503"[Title/Abstract])) OR ("SAR-302503A"[Title/Abstract])) OR ("SAR302503A"[Title/Abstract])) OR ("SAR-302503"[Title/Abstract])) OR ("fedratinib hydrochloride"[Title/Abstract])) OR ("fedratinib hydrochloride monohydrate"[Title/Abstract])) OR ("fedratinib dihydrochloride monohydrate"[Title/Abstract])) OR ("TG101348"[Title/Abstract])) OR ("TG-101348"[Title/Abstract])  #31: #29 OR #30  #32: "upadacitinib" [Concept]  #33: (("upadacitinib"[Title/Abstract]) OR ("ABT-494"[Title/Abstract])) OR ("Rinvoq"[Title/Abstract])  #34: #32 OR #33  #35: "GLPG0634" [Concept]  #36: "filgotinib"[Title/Abstract]  #37: #35 OR #36  #38: "delgocitinib" [Concept]  #39: ("delgocitinib"[Title/Abstract]) OR ("JTE-052"[Title/Abstract])  #40: #38 OR #39  #41: "abrocitinib" [Concept]  #42: ("abrocitinib"[Title/Abstract]) OR ("PF-04965842"[Title/Abstract])  #43: #41 OR #42  #44: ("cerdulatinib"[Title/Abstract]) OR ("PRT062070"[Title/Abstract])  #45: "gandotinib"[Title/Abstract]  #46: "lestaurtinib" [Concept]  #47: ((((((((("lestaurtinib"[Title/Abstract]) OR ("SP-924"[Title/Abstract])) OR ("SPM-924"[Title/Abstract])) OR ("SP924"[Title/Abstract])) OR ("CEP-701"[Title/Abstract])) OR ("CEP 701"[Title/Abstract])) OR ("CEP701"[Title/Abstract])) OR ("KT-5555"[Title/Abstract])) OR ("KT5555"[Title/Abstract])) OR ("KT-555"[Title/Abstract])  #48: #46 OR #47  #49: ((("Momelotinib"[Title/Abstract]) OR ("CYT 387"[Title/Abstract])) OR ("CYT387"[Title/Abstract])) OR ("CYT-387"[Title/Abstract])  #50: ((("pacritinib"[Title/Abstract]) OR ("SB 1518"[Title/Abstract])) OR ("SB1518"[Title/Abstract])) OR ("SB-1518"[Title/Abstract])  #51: "deucravacitinib" [Concept]  #52: ("deucravacitinib"[Title/Abstract]) OR ("BMS-986165"[Title/Abstract])  #53: #51 OR #52  #54: "Solcitinib"[Title/Abstract]  #55: ("decernotinib"[Title/Abstract]) OR ("VX-509"[Title/Abstract])  #56: "ritlecitinib"[Title/Abstract]  #57: "izencitinib" [Concept]  #58: (("izencitinib"[Title/Abstract]) OR ("TD-1473"[Title/Abstract])) OR ("TD1473"[Title/Abstract])  #59: #57 OR #58  #60: "gusacitinib" [Concept]  #61: (("gusacitinib"[Title/Abstract]) OR ("ASN002"[Title/Abstract])) OR ("ASN-002"[Title/Abstract])  #62: #60 OR #61  #63: #13 OR #16 OR #19 OR #22 OR #25 OR #28 OR #31 OR #34 OR #37 OR #40 OR #43 OR #44 OR #45 OR #48 OR #49 OR #50 OR #53 OR #54 OR #55 OR #56 OR #59 OR #62  #64: #10 AND #63 |
| 2.Embase: | #1: 'inflammatory bowel disease'/exp OR 'ulcerative colitis'/exp OR 'crohn disease'/exp  #2: 'inflammatory bowel diseases':ab,ti,kw OR 'inflammatory bowel disease':ab,ti,kw OR 'bowel diseases, inflammatory':ab,ti,kw OR 'colitis, ulcerative':ab,ti,kw OR 'idiopathic proctocolitis':ab,ti,kw OR 'ulcerative colitis':ab,ti,kw OR 'colitis gravis':ab,ti,kw OR 'inflammatory bowel disease, ulcerative colitis type':ab,ti,kw OR 'crohn disease':ab,ti,kw OR 'crohn enteritis':ab,ti,kw OR 'crohns enteritis':ab,ti,kw OR 'regional enteritis':ab,ti,kw OR 'crohns disease':ab,ti,kw OR 'inflammatory bowel disease 1':ab,ti,kw OR 'enteritis, granulomatous':ab,ti,kw OR 'granulomatous enteritis':ab,ti,kw OR 'enteritis, regional':ab,ti,kw OR 'ileocolitis':ab,ti,kw OR 'colitis, granulomatous':ab,ti,kw OR 'granulomatous colitis':ab,ti,kw OR 'ileitis, terminal':ab,ti,kw OR 'terminal ileitis':ab,ti,kw OR 'ileitis, regional':ab,ti,kw OR 'regional ileitides':ab,ti,kw OR 'regional ileitis':ab,ti,kw  #3: #1 OR #2  #4: 'janus kinase inhibitor'/exp OR 'ruxolitinib'/exp OR 'tofacitinib'/exp OR 'oclacitinib'/exp OR 'baricitinib'/exp OR 'peficitinib'/exp OR 'fedratinib'/exp OR 'upadacitinib'/exp OR 'filgotinib'/exp OR 'delgocitinib'/exp OR 'abrocitinib'/exp OR 'cerdulatinib'/exp OR 'gandotinib'/exp OR 'lestaurtinib'/exp OR 'momelotinib'/exp OR 'pacritinib'/exp OR 'deucravacitinib'/exp OR 'solcitinib'/exp OR 'decernotinib'/exp OR 'ritlecitinib'/exp OR 'izencitinib'/exp OR 'gusacitinib'/exp  #5: 'janus kinase inhibitors':ab,ti,kw OR 'inhibitors, janus kinase':ab,ti,kw OR 'kinase inhibitors, janus':ab,ti,kw OR 'jak inhibitors':ab,ti,kw OR 'inhibitors, jak':ab,ti,kw OR 'janus kinase inhibitor':ab,ti,kw OR 'inhibitor, janus kinase':ab,ti,kw OR 'kinase inhibitor, janus':ab,ti,kw OR 'jak inhibitor':ab,ti,kw OR 'inhibitor, jak':ab,ti,kw OR 'ruxolitinib':ab,ti,kw OR 'ruxolitinib phosphate':ab,ti,kw OR 'ruxolitinib monophosphate':ab,ti,kw OR 'incb-18424 phosphate':ab,ti,kw OR 'jakavi':ab,ti,kw OR 'incb018424 phosphate':ab,ti,kw OR 'incb-018424 salt':ab,ti,kw OR 'jakafi':ab,ti,kw OR 'ruxolitinib as phosphate':ab,ti,kw OR 'incb-018424 phosphate':ab,ti,kw OR 'opzelura':ab,ti,kw OR 'incb-018424':ab,ti,kw OR 'inc-424':ab,ti,kw OR 'incb-18424':ab,ti,kw OR 'inc424':ab,ti,kw OR 'incb018424':ab,ti,kw OR 'inca24':ab,ti,kw OR 'tofacitinib':ab,ti,kw OR 'tasocitinib':ab,ti,kw OR 'tofacitinib citrate':ab,ti,kw OR 'xeljanz':ab,ti,kw OR 'cp 690,550':ab,ti,kw OR 'cp690550':ab,ti,kw OR 'cp-690550':ab,ti,kw OR 'cp 690550':ab,ti,kw OR 'cp-690,550':ab,ti,kw OR 'oclacitinib':ab,ti,kw OR 'apoquel':ab,ti,kw OR 'baricitinib':ab,ti,kw OR 'incb-28050':ab,ti,kw OR 'olumiant':ab,ti,kw OR 'baricitinib phosphate':ab,ti,kw OR 'baricitinib phosphate salt':ab,ti,kw OR 'incb028050':ab,ti,kw OR 'incb-028050':ab,ti,kw OR 'ly3009104':ab,ti,kw OR 'ly-3009104':ab,ti,kw OR 'peficitinib':ab,ti,kw OR 'asp015k':ab,ti,kw OR 'fedratinib':ab,ti,kw OR 'nrebic':ab,ti,kw OR 'sar302503':ab,ti,kw OR 'sar-302503a':ab,ti,kw OR 'sar302503a':ab,ti,kw OR 'sar-302503':ab,ti,kw OR 'fedratinib hydrochloride':ab,ti,kw OR 'fedratinib hydrochloride monohydrate':ab,ti,kw OR 'fedratinib dihydrochloride monohydrate':ab,ti,kw OR 'tg101348':ab,ti,kw OR 'tg-101348':ab,ti,kw OR 'upadacitinib':ab,ti,kw OR 'abt-494':ab,ti,kw OR 'rinvoq':ab,ti,kw OR 'filgotinib':ab,ti,kw OR 'glpg0634':ab,ti,kw OR 'delgocitinib':ab,ti,kw OR 'jte-052':ab,ti,kw OR 'abrocitinib':ab,ti,kw OR 'pf-04965842':ab,ti,kw OR 'cerdulatinib':ab,ti,kw OR 'prt062070':ab,ti,kw OR 'gandotinib':ab,ti,kw OR 'lestaurtinib':ab,ti,kw OR 'sp-924':ab,ti,kw OR 'spm-924':ab,ti,kw OR 'sp924':ab,ti,kw OR 'cep-701':ab,ti,kw OR 'cep 701':ab,ti,kw OR 'cep701':ab,ti,kw OR 'kt-5555':ab,ti,kw OR 'kt5555':ab,ti,kw OR 'kt-555':ab,ti,kw OR 'momelotinib':ab,ti,kw OR 'cyt 387':ab,ti,kw OR 'cyt387':ab,ti,kw OR 'cyt-387':ab,ti,kw OR 'pacritinib':ab,ti,kw OR 'sb 1518':ab,ti,kw OR 'sb1518':ab,ti,kw OR 'sb-1518':ab,ti,kw OR 'deucravacitinib':ab,ti,kw OR 'bms-986165':ab,ti,kw OR 'solcitinib':ab,ti,kw OR 'vx-509':ab,ti,kw OR 'decernotinib':ab,ti,kw OR 'ritlecitinib':ab,ti,kw OR 'izencitinib':ab,ti,kw OR 'td-1473':ab,ti,kw OR 'td1473':ab,ti,kw OR 'gusacitinib':ab,ti,kw OR 'asn002':ab,ti,kw OR 'asn-002':ab,ti,kw  #6: #4 OR #5  #7: #3 AND #6 |
| 3.Cochrane library: | #1: MeSH descriptor: [Inflammatory Bowel Diseases] explode all trees  #2: MeSH descriptor: [Colitis, Ulcerative] explode all trees  #3: MeSH descriptor: [Crohn Disease] explode all trees  #4: ("Inflammatory Bowel Diseases" OR "Inflammatory Bowel Disease" OR "Bowel Diseases, Inflammatory" OR "Colitis, Ulcerative" OR "Idiopathic Proctocolitis" OR "Ulcerative Colitis" OR "Colitis Gravis" OR "Inflammatory Bowel Disease, Ulcerative Colitis Type" OR "Crohn Disease" OR "Crohn's Enteritis" OR "Regional Enteritis" OR "Crohn's Disease" OR "Crohns Disease" OR "Inflammatory Bowel Disease 1" OR "Enteritis, Granulomatous" OR "Granulomatous Enteritis" OR "Enteritis, Regional" OR "Ileocolitis" OR "Colitis, Granulomatous" OR "Granulomatous Colitis" OR "Ileitis, Terminal" OR "Terminal Ileitis" OR "Ileitis, Regional" OR "Regional Ileitides" OR "Regional Ileitis"):ti,ab,kw  #5: #1 OR #2 OR #3 OR #4  #6: MeSH descriptor: [Janus Kinase Inhibitors] explode all trees  #7 ("Janus Kinase Inhibitors" OR "Inhibitors, Janus Kinase" OR "Kinase Inhibitors, Janus" OR "JAK Inhibitors" OR "Inhibitors, JAK" OR "Janus Kinase Inhibitor" OR "Inhibitor, Janus Kinase" OR "Kinase Inhibitor, Janus" OR "JAK Inhibitor" OR "Inhibitor, JAK" OR "ruxolitinib" OR "ruxolitinib phosphate" OR "ruxolitinib monophosphate" OR "INCB-18424 phosphate" OR "Jakavi" OR "INCB018424 phosphate" OR "INCB-018424 salt" OR "Jakafi" OR "ruxolitinib as phosphate" OR "INCB-018424 phosphate" OR "opzelura" OR "INCB-018424" OR "INC-424" OR "INCB-18424" OR "INC424" OR "INCB018424" OR "INCA24" OR "tofacitinib" OR "tasocitinib" OR "tofacitinib citrate" OR "Xeljanz" OR "CP 690,550" OR "CP690550" OR "CP-690550" OR "CP 690550" OR "CP-690,550" OR "oclacitinib" OR "apoquel" OR "baricitinib" OR "INCB-28050" OR "Olumiant" OR "baricitinib phosphate" OR "baricitinib phosphate salt" OR "INCB028050" OR "INCB-028050" OR "LY3009104" OR "LY-3009104" OR "peficitinib" OR "ASP015K" OR "fedratinib" OR "nrebic" OR "SAR302503" OR "SAR-302503A" OR "SAR302503A" OR "SAR-302503" OR "fedratinib hydrochloride" OR "fedratinib hydrochloride monohydrate" OR "fedratinib dihydrochloride monohydrate" OR "TG101348" OR "TG-101348" OR "upadacitinib" OR "ABT-494" OR "Rinvoq" OR "filgotinib" OR "GLPG0634" OR "delgocitinib" OR "JTE-052" OR "abrocitinib" OR "PF-04965842" OR "cerdulatinib" OR "PRT062070" OR "gandotinib" OR "lestaurtinib" OR "SP-924" OR "SPM-924" OR "SP924" OR "CEP-701" OR "CEP 701" OR "CEP701" OR "KT-5555" OR "KT5555" OR "KT-555" OR "Momelotinib" OR "CYT 387" OR "CYT387" OR "CYT-387" OR "pacritinib" OR "SB 1518" OR "SB1518" OR "SB-1518" OR "deucravacitinib" OR "BMS-986165" OR "Solcitinib" OR "VX-509" OR "decernotinib" OR "ritlecitinib" OR "izencitinib" OR "TD-1473" OR "TD1473" OR "gusacitinib" OR "ASN002" OR "ASN-002"):ti,ab,kw  #8 #6 OR #7  #9 #5 AND #8 |
| 4.Web of Science: | #1: TS=("Inflammatory Bowel Diseases" OR "Inflammatory Bowel Disease" OR "Bowel Diseases, Inflammatory" OR "Colitis, Ulcerative" OR "Idiopathic Proctocolitis" OR "Ulcerative Colitis" OR "Colitis Gravis" OR "Inflammatory Bowel Disease, Ulcerative Colitis Type" OR "Crohn Disease" OR "Crohn's Enteritis" OR "Regional Enteritis" OR "Crohn's Disease" OR "Crohns Disease" OR "Inflammatory Bowel Disease 1" OR "Enteritis, Granulomatous" OR "Granulomatous Enteritis" OR "Enteritis, Regional" OR "Ileocolitis" OR "Colitis, Granulomatous" OR "Granulomatous Colitis" OR "Ileitis, Terminal" OR "Terminal Ileitis" OR "Ileitis, Regional" OR "Regional Ileitides" OR "Regional Ileitis")  #2: TS=("Janus Kinase Inhibitors" OR "Inhibitors, Janus Kinase" OR "Kinase Inhibitors, Janus" OR "JAK Inhibitors" OR "Inhibitors, JAK" OR "Janus Kinase Inhibitor" OR "Inhibitor, Janus Kinase" OR "Kinase Inhibitor, Janus" OR "JAK Inhibitor" OR "Inhibitor, JAK" OR "ruxolitinib" OR "ruxolitinib phosphate" OR "ruxolitinib monophosphate" OR "INCB-18424 phosphate" OR "Jakavi" OR "INCB018424 phosphate" OR "INCB-018424 salt" OR "Jakafi" OR "ruxolitinib as phosphate" OR "INCB-018424 phosphate" OR "opzelura" OR "INCB-018424" OR "INC-424" OR "INCB-18424" OR "INC424" OR "INCB018424" OR "INCA24" OR "tofacitinib" OR "tasocitinib" OR "tofacitinib citrate" OR "Xeljanz" OR "CP 690,550" OR "CP690550" OR "CP-690550" OR "CP 690550" OR "CP-690,550" OR "oclacitinib" OR "apoquel" OR "baricitinib" OR "INCB-28050" OR "Olumiant" OR "baricitinib phosphate" OR "baricitinib phosphate salt" OR "INCB028050" OR "INCB-028050" OR "LY3009104" OR "LY-3009104" OR "peficitinib" OR "ASP015K" OR "fedratinib" OR "nrebic" OR "SAR302503" OR "SAR-302503A" OR "SAR302503A" OR "SAR-302503" OR "fedratinib hydrochloride" OR "fedratinib hydrochloride monohydrate" OR "fedratinib dihydrochloride monohydrate" OR "TG101348" OR "TG-101348" OR "upadacitinib" OR "ABT-494" OR "Rinvoq" OR "filgotinib" OR "GLPG0634" OR "delgocitinib" OR "JTE-052" OR "abrocitinib" OR "PF-04965842" OR "cerdulatinib" OR "PRT062070" OR "gandotinib" OR "lestaurtinib" OR "SP-924" OR "SPM-924" OR "SP924" OR "CEP-701" OR "CEP 701" OR "CEP701" OR "KT-5555" OR "KT5555" OR "KT-555" OR "Momelotinib" OR "CYT 387" OR "CYT387" OR "CYT-387" OR "pacritinib" OR "SB 1518" OR "SB1518" OR "SB-1518" OR "deucravacitinib" OR "BMS-986165" OR "Solcitinib" OR "VX-509" OR "decernotinib" OR "ritlecitinib" OR "izencitinib" OR "TD-1473" OR "TD1473" OR "gusacitinib" OR "ASN002" OR "ASN-002")  #3: #1 AND #2 |
| 5.Ovid MEDLINE: | #1: exp inflammatory bowel diseases/ or exp colitis, ulcerative/ or exp crohn disease/  #2:("Inflammatory Bowel Diseases" or "Inflammatory Bowel Disease" or "Bowel Diseases, Inflammatory" or "Colitis, Ulcerative" or "Idiopathic Proctocolitis" or "Ulcerative Colitis" or "Colitis Gravis" or "Inflammatory Bowel Disease, Ulcerative Colitis Type" or "Crohn Disease" or "Crohn's Enteritis" or "Regional Enteritis" or "Crohn's Disease" or "Crohns Disease" or "Inflammatory Bowel Disease 1" or "Enteritis, Granulomatous" or "Granulomatous Enteritis" or "Enteritis, Regional" or "Ileocolitis" or "Colitis, Granulomatous" or "Granulomatous Colitis" or "Ileitis, Terminal" or "Terminal Ileitis" or "Ileitis, Regional" or "Regional Ileitides" or "Regional Ileitis").ab,kw,ti.  #3: #1 or #2  #4: exp Janus Kinase Inhibitors/  #5: ("Janus Kinase Inhibitors" or "Inhibitors, Janus Kinase" or "Kinase Inhibitors, Janus" or "JAK Inhibitors" or "Inhibitors, JAK" or "Janus Kinase Inhibitor" or "Inhibitor, Janus Kinase" or "Kinase Inhibitor, Janus" or "JAK Inhibitor" or "Inhibitor, JAK" or "ruxolitinib" or "ruxolitinib phosphate" or "ruxolitinib monophosphate" or "INCB-18424 phosphate" or "Jakavi" or "INCB018424 phosphate" or "INCB-018424 salt" or "Jakafi" or "ruxolitinib as phosphate" or "INCB-018424 phosphate" or "opzelura" or "INCB-018424" or "INC-424" or "INCB-18424" or "INC424" or "INCB018424" or "INCA24" or "tofacitinib" or "tasocitinib" or "tofacitinib citrate" or "Xeljanz" or "CP 690,550" or "CP690550" or "CP-690550" or "CP 690550" or "CP-690,550" or "oclacitinib" or "apoquel" or "baricitinib" or "INCB-28050" or "Olumiant" or "baricitinib phosphate" or "baricitinib phosphate salt" or "INCB028050" or "INCB-028050" or "LY3009104" or "LY-3009104" or "peficitinib" or "ASP015K" or "fedratinib" or "nrebic" or "SAR302503" or "SAR-302503A" or "SAR302503A" or "SAR-302503" or "fedratinib hydrochloride" or "fedratinib hydrochloride monohydrate" or "fedratinib dihydrochloride monohydrate" or "TG101348" or "TG-101348" or "upadacitinib" or "ABT-494" or "Rinvoq" or "filgotinib" or "GLPG0634" or "delgocitinib" or "JTE-052" or "abrocitinib" or "PF-04965842" or "cerdulatinib" or "PRT062070" or "gandotinib" or "lestaurtinib" or "SP-924" or "SPM-924" or "SP924" or "CEP-701" or "CEP 701" or "CEP701" or "KT-5555" or "KT5555" or "KT-555" or "Momelotinib" or "CYT 387" or "CYT387" or "CYT-387" or "pacritinib" or "SB 1518" or "SB1518" or "SB-1518" or "deucravacitinib" or "BMS-986165" or "Solcitinib" or "VX-509" or "decernotinib" or "ritlecitinib" or "izencitinib" or "TD-1473" or "TD1473" or "gusacitinib" or "ASN002" or "ASN-002").ab,kw,ti.  #6: #4 or #5  #7: #3 and #6 |
| 6.Scopus: | ( TITLE-ABS-KEY ( "Inflammatory Bowel Diseases" OR "Inflammatory Bowel Disease" OR "Bowel Diseases, Inflammatory" OR "Colitis, Ulcerative" OR "Idiopathic Proctocolitis" OR "Ulcerative Colitis" OR "Colitis Gravis" OR "Inflammatory Bowel Disease, Ulcerative Colitis Type" OR "Crohn Disease" OR "Crohn's Enteritis" OR "Regional Enteritis" OR "Crohn's Disease" OR "Crohns Disease" OR "Inflammatory Bowel Disease 1" OR "Enteritis, Granulomatous" OR "Granulomatous Enteritis" OR "Enteritis, Regional" OR "Ileocolitis" OR "Colitis, Granulomatous" OR "Granulomatous Colitis" OR "Ileitis, Terminal" OR "Terminal Ileitis" OR "Ileitis, Regional" OR "Regional Ileitides" OR "Regional Ileitis" ) AND TITLE-ABS-KEY ( "Janus Kinase Inhibitors" OR "Inhibitors, Janus Kinase" OR "Kinase Inhibitors, Janus" OR "JAK Inhibitors" OR "Inhibitors, JAK" OR "Janus Kinase Inhibitor" OR "Inhibitor, Janus Kinase" OR "Kinase Inhibitor, Janus" OR "JAK Inhibitor" OR "Inhibitor, JAK" OR "ruxolitinib" OR "ruxolitinib phosphate" OR "ruxolitinib monophosphate" OR "INCB-18424 phosphate" OR "Jakavi" OR "INCB018424 phosphate" OR "INCB-018424 salt" OR "Jakafi" OR "ruxolitinib as phosphate" OR "INCB-018424 phosphate" OR "opzelura" OR "INCB-018424" OR "INC-424" OR "INCB-18424" OR "INC424" OR "INCB018424" OR "INCA24" OR "tofacitinib" OR "tasocitinib" OR "tofacitinib citrate" OR "Xeljanz" OR "CP 690,550" OR "CP690550" OR "CP-690550" OR "CP 690550" OR "CP-690,550" OR "oclacitinib" OR "apoquel" OR "baricitinib" OR "INCB-28050" OR "Olumiant" OR "baricitinib phosphate" OR "baricitinib phosphate salt" OR "INCB028050" OR "INCB-028050" OR "LY3009104" OR "LY-3009104" OR "peficitinib" OR "ASP015K" OR "fedratinib" OR "nrebic" OR "SAR302503" OR "SAR-302503A" OR "SAR302503A" OR "SAR-302503" OR "fedratinib hydrochloride" OR "fedratinib hydrochloride monohydrate" OR "fedratinib dihydrochloride monohydrate" OR "TG101348" OR "TG-101348" OR "upadacitinib" OR "ABT-494" OR "Rinvoq" OR "filgotinib" OR "GLPG0634" OR "delgocitinib" OR "JTE-052" OR "abrocitinib" OR "PF-04965842" OR "cerdulatinib" OR "PRT062070" OR "gandotinib" OR "lestaurtinib" OR "SP-924" OR "SPM-924" OR "SP924" OR "CEP-701" OR "CEP 701" OR "CEP701" OR "KT-5555" OR "KT5555" OR "KT-555" OR "Momelotinib" OR "CYT 387" OR "CYT387" OR "CYT-387" OR "pacritinib" OR "SB 1518" OR "SB1518" OR "SB-1518" OR "deucravacitinib" OR "BMS-986165" OR "Solcitinib" OR "VX-509" OR "decernotinib" OR "ritlecitinib" OR "izencitinib" OR "TD-1473" OR "TD1473" OR "gusacitinib" OR "ASN002" OR "ASN-002" ) ) |
| 7.ProQuest: | #1: AB,TI,IF("Inflammatory Bowel Diseases" or "Inflammatory Bowel Disease" or "Bowel Diseases, Inflammatory" or "Colitis, Ulcerative" or "Idiopathic Proctocolitis" or "Ulcerative Colitis" or "Colitis Gravis" or "Inflammatory Bowel Disease, Ulcerative Colitis Type" or "Crohn Disease" or "Crohn's Enteritis" or "Regional Enteritis" or "Crohn's Disease" or "Crohns Disease" or "Inflammatory Bowel Disease 1" or "Enteritis, Granulomatous" or "Granulomatous Enteritis" or "Enteritis, Regional" or "Ileocolitis" or "Colitis, Granulomatous" or "Granulomatous Colitis" or "Ileitis, Terminal" or "Terminal Ileitis" or "Ileitis, Regional" or "Regional Ileitides" or "Regional Ileitis")  #2: AB,TI,IF("Janus Kinase Inhibitors" or "Inhibitors, Janus Kinase" or "Kinase Inhibitors, Janus" or "JAK Inhibitors" or "Inhibitors, JAK" or "Janus Kinase Inhibitor" or "Inhibitor, Janus Kinase" or "Kinase Inhibitor, Janus" or "JAK Inhibitor" or "Inhibitor, JAK" or "ruxolitinib" or "ruxolitinib phosphate" or "ruxolitinib monophosphate" or "INCB-18424 phosphate" or "Jakavi" or "INCB018424 phosphate" or "INCB-018424 salt" or "Jakafi" or "ruxolitinib as phosphate" or "INCB-018424 phosphate" or "opzelura" or "INCB-018424" or "INC-424" or "INCB-18424" or "INC424" or "INCB018424" or "INCA24" or "tofacitinib" or "tasocitinib" or "tofacitinib citrate" or "Xeljanz" or "CP 690,550" or "CP690550" or "CP-690550" or "CP 690550" or "CP-690,550" or "oclacitinib" or "apoquel" or "baricitinib" or "INCB-28050" or "Olumiant" or "baricitinib phosphate" or "baricitinib phosphate salt" or "INCB028050" or "INCB-028050" or "LY3009104" or "LY-3009104" or "peficitinib" or "ASP015K" or "fedratinib" or "nrebic" or "SAR302503" or "SAR-302503A" or "SAR302503A" or "SAR-302503" or "fedratinib hydrochloride" or "fedratinib hydrochloride monohydrate" or "fedratinib dihydrochloride monohydrate" or "TG101348" or "TG-101348" or "upadacitinib" or "ABT-494" or "Rinvoq" or "filgotinib" or "GLPG0634" or "delgocitinib" or "JTE-052" or "abrocitinib" or "PF-04965842" or "cerdulatinib" or "PRT062070" or "gandotinib" or "lestaurtinib" or "SP-924" or "SPM-924" or "SP924" or "CEP-701" or "CEP 701" or "CEP701" or "KT-5555" or "KT5555" or "KT-555" or "Momelotinib" or "CYT 387" or "CYT387" or "CYT-387" or "pacritinib" or "SB 1518" or "SB1518" or "SB-1518" or "deucravacitinib" or "BMS-986165" or "Solcitinib" or "VX-509" or "decernotinib" or "ritlecitinib" or "izencitinib" or "TD-1473" or "TD1473" or "gusacitinib" or "ASN002" or "ASN-002")  #3: #1 AND #2 |
| 8.ClinicalTrials.gov: | Keywords: Inflammatory bowel diseases, Inflammatory Bowel Disease, Ulcerative Colitis, Crohn Disease, Crohn's Disease, Janus Kinase Inhibitors, JAK Inhibitors, ruxolitinib, tofacitinib, oclacitinib, baricitinib, peficitinib, fedratinib, upadacitinib, filgotinib, delgocitinib, abrocitinib, cerdulatinib, gandotinib, lestaurtinib, momelotinib, pacritinib, deucravacitinib, Solcitinib, Decernotinib, ritlecitinib, Izencitinib, Gusacitinib |

**Supplementary table 3 Basic information of included JAKinibs.**

| JAK inhibitors | Research status | JAK selectivity | Characteristic | Approved for IBD | Developing for IBD |
| --- | --- | --- | --- | --- | --- |
| Peficitinib | First-generation | Pan-JAK | - | - | √ (UC) |
| Tofacitinib | First-generation | Pan-JAK | - | √ | - |
| Upadacitinib | Second-generation | JAK1-selective | - | √ | - |
| Filgotinib | Second-generation | JAK1-selective | - | √ | - |
| Deucravacitinib | Third-generation | TYK2-selective | Allosteric regulation | - | √ (UC and CD) |
| Ritlecitinib | New listed | JAK3-selective | - | √ (only in China) | √ (UC and CD) |
| Brepocitinib | Unlisted | JAK1, TYK2-selective | - | - | - |
| Ivarmacitinib | Unlisted | JAK1-selective | - | - | - |
| Izencitinib | Unlisted | Pan-JAK | Gut-selective | - | √ (UC and CD) |

JAKinibs: Janus kinase inhibitors; TYK: tyrosine kinase; IBD: inflammatory bowel disease; UC: ulcerative colitis; CD: Crohn’s disease.

**Supplementary table 4-1 R codes in the network meta-analysis (without dose consideration).**

| R code | Without dose consideration |
| --- | --- |
|  | **# Set working directory**  setwd("D:/data")  **# Call R packages**  library(readxl)  library(dplyr)  library(netmeta)  **# Import data**  datar <- read_excel("sheet.xlsx")  write.csv(datar,"sheet.csv", row.names = FALSE)  data <- read.csv("sheet.csv")  **# Summary data**  sum(data$size) #Total sample of the studies  summary(data$age) #Median (IQR) of mean age  summary(data$pf) #Median (IQR) of proportion of female participants; pf: proportion of female participants  summary(data$sd) #Median (IQR) of study duration; sd: study duration  summary(data$dd) #Median (IQR) of disease duration; dd: disease duration  **# Transitivity assessment**  par(mar = c(4, 4, 4, 1))  boxplot(data$age~data$t, col = "green",  xlab = "Treatment groups", ylab = "Years",  cex.axis = 0.12)  sample.size <- tapply(data$age, data$t, length)  ss.ch <- paste("N = ", sample.size, sep = "")  mtext(ss.ch, at = 1:length(unique(data$t)), line = 2, side = 1, cex = 0.5)  title(" Mean age of the Participants (years)")  par(mar = c(4, 4, 4, 1))  boxplot(data$pf~data$t, col = "green",  xlab = "Treatment groups", ylab = "Percent",  cex.axis = 0.12)  sample.size <- tapply(data$pf, data$t, length)  ss.ch <- paste("N = ", sample.size, sep = "")  mtext(ss.ch, at = 1:length(unique(data$t)), line = 2, side = 1, cex = 0.5)  title("Percentage of female participants")  par(mar = c(4, 4, 4, 1))  boxplot(data$sd~data$t, col = "green",  xlab = "Treatment groups", ylab = "Weeks",  cex.axis = 0.12)  sample.size <- tapply(data$sd, data$t, length)  ss.ch <- paste("N = ", sample.size, sep = "")  mtext(ss.ch, at = 1:length(unique(data$t)), line = 2, side = 1, cex = 0.5)  title("Study duration (weeks)")  **# Data subset**  data.p<- subset(data, id %in% c(1:12,15:21,23:24,26:28,30)) # for the analysis of disease duration because of the existence of missing values  **# Transitivity assessment**  par(mar = c(4, 4, 4, 1))  boxplot(data.p$dd~data.p$t, col = "green",  xlab = "Treatment groups", ylab = "Years",  cex.axis = 0.15)  sample.size <- tapply(data.p$dd, data.p$t, length)  ss.ch <- paste("N = ", sample.size, sep = "")  mtext(ss.ch, at = 1:length(unique(data.p$t)), line = 2, side = 1, cex = 0.5)  title("Mean disease duration of the participants (years)")  # **Netmeta analysis**  **# Import data**  eff <- read.csv("MACE-the final-combine.CSV",  header = TRUE,  stringsAsFactors = FALSE)  **# Perform Network-meta-analysis Random effect model**  deff <- pairwise(treat = t,  n,  event = r,  data = eff,  studlab = id,  sm = "OR")  e.netmeta <-netmeta(deff,  comb.fixed = FALSE,  comb.random = TRUE)  e.netmeta  **# Decomposition of Cochran’s Q**  Q<-decomp.design(e.netmeta)  write.csv(Q$Q.decomp, "Q.csv")  **# Network plot**  netgraph(e.netmeta,  start ="circle",  cex = 1,  col = "black",  plastic = FALSE,  points = TRUE,  col.points = "steelblue",  cex.points =15*sqrt(n.trts/max(n.trts)),  thickness = "number.of.studies",  lwd.max = 12,  lwd.min = 1,  multiarm = F)  dev.off()  **# Forest plot**  npw.p <- netpairwise(e.netmeta)  pdf("Details of forest.pdf",height = 200, width = 30)  forest(npw.p)  dev.off()  forest(e.netmeta,  ref = "Placebo",  pooled = "random",  digits = 2,  col.square = "steelblue",  smlab = "Random effects model",  leftlabs = "Risk",  drop = TRUE,  sortvar = -TE)  dev.off()  **# League table**  league <- netleague(e.netmeta,  backtransf = TRUE,  direct = FALSE,  fixed = FALSE,  digits =2,  bracket = "(",  separator = " to ")  league  write.csv(league$random, "netleague2.csv")  **# Distribution of direct and indirect evidence**  library("ggplot2")  library("gridExtra")  library(dmetar)  d.evidence <- direct.evidence.plot(e.netmeta, random=TRUE)  plot(d.evidence)  dev.off()  **# Node-splitting method**  netsplit(e.netmeta)  projectX<-netsplit(e.netmeta)  print(projectX)  pdf("inconsistency-with direct.pdf",height = 200, width = 30)  forest(projectX, fontsize = 6, spacing = 0.5, addrow.subgroups = FALSE, show = "with.direct")  dev.off()  pdf("inconsistency-all.pdf",height = 200, width = 30)  forest(projectX, fontsize = 10, spacing = 1, addrow.subgroups = FALSE, show = "all")  dev.off()  **# SUCRA and P-score**  netrank1 <-netrank(e.netmeta, method = "SUCRA", small.values = "bad", nsim = 100000)  print(netrank1)  netrank2 <-netrank(e.netmeta, method = "P-score", small.values = "bad", nsim = 100000)  print(netrank2)  plot(netrank2,  low = "green",  mid = "yellow",  high = "red")  dev.off()  **# Funnel plot**  colors <- c(  "Brepocitinib:Placebo" = "mediumvioletred",  "Brepocitinib:Ritlecitinib" = "coral3",  "Deucravacitinib:Placebo" = "purple",  "Filgotinib:Placebo" = "dodgerblue4",  "Ivarmacitinib:Placebo" = "yellow3",  "Izencitinib:Placebo" = "orange",  "Peficitinib:Placebo" = "lightslateblue",  "Placebo:Ritlecitinib" = "pink2",  "Placebo:Tofacitinib" = "lightgreen",  "Placebo:Upadacitinib" = "cadetblue4")  legend <-c(  "Brepocitinib:Placebo",  "Brepocitinib:Ritlecitinib",  "Deucravacitinib:Placebo",  "Filgotinib:Placebo",  "Ivarmacitinib:Placebo",  "Izencitinib:Placebo",  "Peficitinib:Placebo",  "Placebo:Ritlecitinib",  "Placebo:Tofacitinib",  "Placebo:Upadacitinib")  trts_ef <- substr(e.netmeta$trts, 0, 25)  comparison_ef<- trts_ef[trts_ef != "Placebo"]  ord_ef<- c(comparison_ef, "Placebo")  funnel(e.netmeta,  order = ord_ef,  pch = rep(19),  col = colors,  legend = FALSE,  linreg = TRUE,  text.linreg = "(Egger's test)",  pos.tests = "topright")  legend("topleft",  legend = legend,  pch = rep(19),  col = colors,  cex = 0.65)  dev.off()  **# Rankogram**  plot(rankogram(e.netmeta, nsim = 100000, small.values = "bad", cumulative.rankprob = FALSE),sort = TRUE,type = "line")  plot1<-rankogram(e.netmeta, nsim = 100000, small.values = "bad", cumulative.rankprob = FALSE)  plot1  par(mar = c(4, 4, 4, 4))  plot(plot1)  dev.off()  **# Cumulative Rankogram**  plot(rankogram(e.netmeta, nsim = 100000, small.values = "bad", cumulative.rankprob = TRUE),sort = TRUE,type = "line")  plot2<-rankogram(e.netmeta, nsim = 100000, small.values = "bad", cumulative.rankprob = TRUE)  plot2  par(mar = c(4, 4, 4, 4))  plot(plot2)  dev.off() |

**Supplementary table 4-2 R codes in the network meta-analysis (with dose consideration).**

| R code | With dose consideration |
| --- | --- |
|  | **# Set working directory**  setwd("D:/data")  **# Call R packages**  library(readxl)  library(dplyr)  library(netmeta)  **# Import data**  datar <- read_excel("sheet.xlsx")  write.csv(datar,"sheet.csv", row.names = FALSE)  data <- read.csv("sheet.csv")  **# Summary data**  sum(data$size) #Total sample of the studies  summary(data$age) #Median (IQR) of mean age  summary(data$pf) #Median (IQR) of proportion of female  summary(data$sd) #Median (IQR) of study duration  summary(data$dd) #Median (IQR) of disease duration  **# Transitivity assessment**  par(mar = c(4, 4, 4, 1))  boxplot(data$age~data$t, col = "green",  xlab = "Treatment groups", ylab = "Years",  cex.axis = 0.12)  sample.size <- tapply(data$age, data$t, length)  ss.ch <- paste("N = ", sample.size, sep = "")  mtext(ss.ch, at = 1:length(unique(data$t)), line = 2, side = 1, cex = 0.5)  title(" Mean age of the Participants (years)")  par(mar = c(4, 4, 4, 1))  boxplot(data$pf~data$t, col = "green",  xlab = "Treatment groups", ylab = "Percent",  cex.axis = 0.12)  sample.size <- tapply(data$pf, data$t, length)  ss.ch <- paste("N = ", sample.size, sep = "")  mtext(ss.ch, at = 1:length(unique(data$t)), line = 2, side = 1, cex = 0.5)  title("Percentage of female participants")  par(mar = c(4, 4, 4, 1))  boxplot(data$sd~data$t, col = "green",  xlab = "Treatment groups", ylab = "Weeks",  cex.axis = 0.12)  sample.size <- tapply(data$sd, data$t, length)  ss.ch <- paste("N = ", sample.size, sep = "")  mtext(ss.ch, at = 1:length(unique(data$t)), line = 2, side = 1, cex = 0.5)  title("Study duration (weeks)")  **# Data subset**  data.p<- subset(data, id %in% c(1:12,15:21,23:24,26:28,30)) # for proportion analysis  **# Transitivity assessment**  par(mar = c(4, 4, 4, 1))  boxplot(data.p$dd~data.p$t, col = "green",  xlab = "Treatment groups", ylab = "Years",  cex.axis = 0.15)  sample.size <- tapply(data.p$dd, data.p$t, length)  ss.ch <- paste("N = ", sample.size, sep = "")  mtext(ss.ch, at = 1:length(unique(data.p$t)), line = 2, side = 1, cex = 0.5)  title("Mean disease duration of the participants (years)")  # **Netmeta analysis**  **# Import data**  eff <- read.csv("MACE-the final.CSV",  header = TRUE,  stringsAsFactors = FALSE)  **# Perform Network-meta-analysis Random effect model**  deff <- pairwise(treat = t,  n,  event = r,  data = eff,  studlab = id,  sm = "OR")  e.netmeta <-netmeta(deff,  comb.fixed = FALSE,  comb.random = TRUE)  e.netmeta  **# Decomposition of Cochran’s Q**  Q<-decomp.design(e.netmeta)  write.csv(Q$Q.decomp, "Q.csv")  **# Network plot**  netgraph(e.netmeta,  start ="circle",  cex = 1,  col = "black",  plastic = FALSE,  points = TRUE,  col.points = "steelblue",  cex.points =15*sqrt(n.trts/max(n.trts)),  thickness = "number.of.studies",  lwd.max = 12,  lwd.min = 1,  multiarm = F)  dev.off()  **# Forest plot**  npw.p <- netpairwise(e.netmeta)  pdf("Details of forest.pdf",height = 200, width = 30)  forest(npw.p)  dev.off()  forest(e.netmeta,  ref = "Placebo",  pooled = "random",  digits = 2,  col.square = "steelblue",  smlab = "Random effects model",  leftlabs = "Risk",  drop = TRUE,  sortvar = -TE)  dev.off()  **# League table**  league <- netleague(e.netmeta,  backtransf = TRUE,  direct = FALSE,  fixed = FALSE,  digits =2,  bracket = "(",  separator = " to ")  league  write.csv(league$random, "netleague.csv")  **# Distribution of direct and indirect evidence**  library("ggplot2")  library("gridExtra")  library(dmetar)  d.evidence <- direct.evidence.plot(e.netmeta, random=TRUE)  plot(d.evidence)  dev.off()  **# Node-splitting method**  netsplit(e.netmeta)  projectX<-netsplit(e.netmeta)  print(projectX)  pdf("inconsistency-both.pdf",height = 200, width = 30)  forest(projectX, fontsize = 6, spacing = 0.5, addrow.subgroups = FALSE,show = "both")  dev.off()  pdf("inconsistency-with direct.pdf",height = 200, width = 30)  forest(projectX, fontsize = 6, spacing = 0.5, addrow.subgroups = FALSE, show = "with.direct")  dev.off()  pdf("inconsistency-all.pdf",height = 200, width = 30)  forest(projectX, fontsize = 6, spacing = 0.5, addrow.subgroups = FALSE, show = "all")  dev.off()  **# SUCRA and P-score**  netrank1 <-netrank(e.netmeta, method = "SUCRA", small.values = "bad", nsim = 100000)  print(netrank1)  netrank2 <-netrank(e.netmeta, method = "P-score", small.values = "bad", nsim = 100000)  print(netrank2)  plot(netrank2,  low = "green",  mid = "yellow",  high = "red")  dev.off()  **# Funnel plot**  colors <- c(  "Brepocitinib:Placebo" = "thistle",  "Brepocitinib:Ritlecitinib" = "coral3",  "Deucravacitinib:Placebo" = "orange3",  "Filgotinib, 100 mg QD:Filgotinib, 200 mg QD" = "dodgerblue4",  "Filgotinib, 100 mg QD:Placebo" = "yellow3",  "Filgotinib, 200 mg QD:Placebo" = "orange",  "Ivarmacitinib:Placebo" = "lightslateblue",  "Izencitinib:Placebo" = "blue",  "Peficitinib:Placebo" = "lightgreen",  "Placebo:Ritlecitinib" = "cadetblue4",  "Placebo:Tofacitinib, 0.5 mg BID" = "magenta",  "Placebo:Tofacitinib, 1 mg BID" = "pink",  "Placebo:Tofacitinib, 10 mg BID" = "goldenrod4",  "Placebo:Tofacitinib, 15 mg BID" = "darkgrey",  "Placebo:Tofacitinib, 3 mg BID" = "lightblue3",  "Placebo:Tofacitinib, 5 mg BID" = "red",  "Placebo:Upadacitinib, 15 mg QD" = "mediumvioletred",  "Placebo:Upadacitinib, 30 mg QD" = "pink2",  "Placebo:Upadacitinib, 45 mg QD" = "red4",  "Tofacitinib, 0.5 mg BID:Tofacitinib, 10 mg BID" = "purple4",  "Tofacitinib, 0.5 mg BID:Tofacitinib, 15 mg BID" = "purple",  "Tofacitinib, 0.5 mg BID:Tofacitinib, 3 mg BID" = "grey",  "Tofacitinib, 1 mg BID:Tofacitinib, 15 mg BID" = "pink4",  "Tofacitinib, 1 mg BID:Tofacitinib, 5 mg BID" = "pink3",  "Tofacitinib, 10 mg BID:Tofacitinib, 15 mg BID" = "coral4",  "Tofacitinib, 10 mg BID:Tofacitinib, 3 mg BID" = "coral1",  "Tofacitinib, 10 mg BID:Tofacitinib, 5 mg BID" = "lightblue4",  "Tofacitinib, 15 mg BID:Tofacitinib, 3 mg BID" = "darkgreen",  "Tofacitinib, 15 mg BID:Tofacitinib, 5 mg BID" = "green",  "Upadacitinib, 15 mg QD:Upadacitinib, 30 mg QD" = "black",  "Upadacitinib, 15 mg QD:Upadacitinib, 45 mg QD" = "blue4",  "Upadacitinib, 30 mg QD:Upadacitinib, 45 mg QD" = "orange4")  legend <-c("Brepocitinib:Placebo",  "Brepocitinib:Ritlecitinib",  "Deucravacitinib:Placebo",  "Filgotinib, 100 mg QD:Filgotinib, 200 mg QD",  "Filgotinib, 100 mg QD:Placebo",  "Filgotinib, 200 mg QD:Placebo",  "Ivarmacitinib:Placebo",  "Izencitinib:Placebo",  "Peficitinib:Placebo",  "Placebo:Ritlecitinib",  "Placebo:Tofacitinib, 0.5 mg BID",  "Placebo:Tofacitinib, 1 mg BID",  "Placebo:Tofacitinib, 10 mg BID",  "Placebo:Tofacitinib, 15 mg BID",  "Placebo:Tofacitinib, 3 mg BID",  "Placebo:Tofacitinib, 5 mg BID",  "Placebo:Upadacitinib, 15 mg QD",  "Placebo:Upadacitinib, 30 mg QD",  "Placebo:Upadacitinib, 45 mg QD",  "Tofacitinib, 0.5 mg BID:Tofacitinib, 10 mg BID",  "Tofacitinib, 0.5 mg BID:Tofacitinib, 15 mg BID",  "Tofacitinib, 0.5 mg BID:Tofacitinib, 3 mg BID",  "Tofacitinib, 1 mg BID:Tofacitinib, 15 mg BID",  "Tofacitinib, 1 mg BID:Tofacitinib, 5 mg BID",  "Tofacitinib, 10 mg BID:Tofacitinib, 15 mg BID",  "Tofacitinib, 10 mg BID:Tofacitinib, 3 mg BID",  "Tofacitinib, 10 mg BID:Tofacitinib, 5 mg BID",  "Tofacitinib, 15 mg BID:Tofacitinib, 3 mg BID",  "Tofacitinib, 15 mg BID:Tofacitinib, 5 mg BID",  "Upadacitinib, 15 mg QD:Upadacitinib, 30 mg QD",  "Upadacitinib, 15 mg QD:Upadacitinib, 45 mg QD",  "Upadacitinib, 30 mg QD:Upadacitinib, 45 mg QD")  trts_ef <- substr(e.netmeta$trts, 0, 25)  comparison_ef<- trts_ef[trts_ef != "Placebo"]  ord_ef<- c(comparison_ef, "Placebo")  funnel(e.netmeta,  order = ord_ef,  pch = rep(19),  col = colors,  legend = FALSE,  linreg = TRUE,  text.linreg = "(Egger's test)",  pos.tests = "topright")  legend("topleft",  legend = legend,  pch = rep(19),  col = colors,  cex = 0.35)  dev.off()  **# Rankogram**  plot(rankogram(e.netmeta, nsim = 100000, small.values = "bad", cumulative.rankprob = FALSE),sort = TRUE,type = "line")  plot1<-rankogram(e.netmeta, nsim = 100000, small.values = "bad", cumulative.rankprob = FALSE)  plot1  par(mar = c(4, 4, 4, 4))  plot(plot1)  dev.off()  **# Cumulative ranking**  plot(rankogram(e.netmeta, nsim = 100000, small.values = "bad", cumulative.rankprob = TRUE),sort = TRUE,type = "line")  plot2<-rankogram(e.netmeta, nsim = 100000, small.values = "bad", cumulative.rankprob = TRUE)  plot2  par(mar = c(4, 4, 4, 4))  plot(plot2)  dev.off() |

**Supplementary table 5-1. Baseline characteristics of included RCTs.**

| Number | Study | Register number | Study phase | Intervention | Sample size | Age (years) | Percentage of female (%) | Disease duration (years) | Outcome | Study duration (weeks) | IBD type |
| --- | --- | --- | --- | --- | --- | --- | --- | --- | --- | --- | --- |
| 1 | William J. Sandborn 2013 | NCT00615199 | Induction | Placebo BID | 34 | 35.7 ± 12.7 | 64.7 | 8.2 (0.1–35.6) | None | 4 | CD |
|  |  |  |  | Tofacitinib 1 mg BID | 36 | 36.6 ± 12.2 | 30.6 | 11.1 (0.1–28.5) |  |  |  |
|  |  |  |  | Tofacitinib 5 mg BID | 34 | 38.7 ± 10.2 | 58.8 | 10.9 (0.3–29.3) |  |  |  |
|  |  |  |  | Tofacitinib 15 mg BID | 35 | 38.1 ± 11.7 | 48.6 | 11.2 (1.5–36.3) |  |  |  |
| 2 | William J. Sandborn 2013 | NCT00787202 | Induction | Placebo BID | 48 | 42.5 ± 14.7 | 52.1 | 8.8 ± 5.4 | ②③ | 8 | UC |
|  |  |  |  | Tofacitinib 0.5 mg BID | 31 | 43.8 ± 13.4 | 45.2 | 8.8 ± 9.1 |  |  |  |
|  |  |  |  | Tofacitinib 3 mg BID | 33 | 42.5 ± 14.3 | 42.4 | 8.9 ± 8.8 |  |  |  |
|  |  |  |  | Tofacitinib 10 mg BID | 33 | 43.2 ± 12.8 | 36.4 | 10.9 ± 6.6 |  |  |  |
|  |  |  |  | Tofacitinib 15 mg BID | 49 | 41.2 ± 13.5 | 46.9 | 7.6 ± 5.7 |  |  |  |
| 3 | Julian Panés 2017 | NCT01393626 | Induction | Placebo BID | 91 | 37.2 ± 11.7 | 65.9 | 10.9 ± 8.6 | None | 8 | CD |
|  |  |  |  | Tofacitinib 5 mg BID | 86 | 40.2 ± 11.5 | 37.2 | 11.2 ± 8.2 |  |  |  |
|  |  |  |  | Tofacitinib 10 mg BID | 86 | 39.3 ± 13.7 | 54.7 | 11.3 ± 9.7 |  |  |  |
| 4 | Julian Panés 2016 | NCT01393899 | Maintenance | Placebo BID | 59 | 41.5 ± 12.8 | 54.2 | 12.5 ± 9.3 | None | 26 | CD |
|  |  |  |  | Tofacitinib 5 mg BID | 60 | 38.1 ± 11.9 | 50.0 | 11.2 ± 8.3 |  |  |  |
|  |  |  |  | Tofacitinib 10 mg BID | 61 | 39.0 ± 13.1 | 39.3 | 12.6 ± 10.0 |  |  |  |
| 5 | William J. Sandborn, 2017 | NCT01458574 | Maintenance | Placebo BID | 198 | 43.4 ± 14.0 | 41.4 | 7.2 (0.6–42.7) | ①⑤ | 52 | UC |
|  |  |  |  | Tofacitinib 5 mg BID | 198 | 41.9 ± 13.7 | 48.0 | 6.5 (0.6–40.3) |  |  |  |
|  |  |  |  | Tofacitinib 10 mg BID | 197 | 42.9 ± 14.4 | 44.2 | 6.8 (0.6–35.7) |  |  |  |
| 6 | William J. Sandborn, 2016 | NCT01458951 | Induction | Placebo BID | 112 | 40.4±13.2 | 50.9 | 6.2 (0.4–27.9) | None | 8 | UC |
|  |  |  |  | Tofacitinib 10 mg BID | 429 | 41.1±13.5 | 39.6 | 6.0 (0.4–39.4) |  |  |  |
| 7 | William J. Sandborn 2016 | NCT01465763 | Induction | Placebo BID | 122 | 41.8±15.3 | 36.9 | 6.0 (0.5–36.2) | ④ | 8 | UC |
|  |  |  |  | Tofacitinib 10 mg BID | 476 | 41.3±14.1 | 41.8 | 6.5 (0.3–42.5) |  |  |  |
| 8.1 | William J. Sandborn 2022 | NCT02819635 | Induction (Substudy 1) | Placebo QD | 46 | 40 (21–67) | 37.0 | 5.86 (0.4–30.8) | ③④ | 8 | UC |
|  |  |  |  | Upadacitinib 15 mg QD | 49 | 47 (22–71) | 38.8 | 4.58 (0.2–43.0) |  |  |  |
|  |  |  |  | Upadacitinib 30 mg QD | 117 | 41 (19–75) | 40.2 | 7.03 (0.3–28.0) |  |  |  |
|  |  |  |  | Upadacitinib 45 mg QD | 123 | 39 (19–74) | 35.8 | 5.99 (0.2–35.3) |  |  |  |
| 8.2 |  |  | Induction (Substudy 2) | Placebo QD | 154 | 44.5 ± 23.0 | 37.0 | 6.0 ± 10.0 | None | 8 | UC |
|  |  |  |  | Upadacitinib 45 mg QD | 319 | 43.0 ± 23.0 | 38.0 | 6.6 ± 9.6 |  |  |  |
| 8.3 |  |  | Maintenance | Placebo QD | 149 | 40.0 ± 21.0 | 43.0 | 6.2 ± 8.6 | ①③ | 52 | UC |
|  |  |  |  | Upadacitinib 15 mg QD | 148 | 40.0 ± 22.0 | 36.0 | 6.4 ± 10.6 |  |  |  |
|  |  |  |  | Upadacitinib 30 mg QD | 154 | 41.0 ± 7.0 | 44.0 | 6.0 ± 9.7 |  |  |  |
| 9.1 | Brian G Feagan 2021 | NCT02914522 | Induction  (biologic-naive patients) | Placebo QD | 137 | 41 ± 12.9 | 36.5 | 6.4 ± 7.4 | ④ | 10 | UC |
|  |  |  |  | Filgotinib 100 mg QD | 277 | 42 ± 13.3 | 43.3 | 6.7 ± 7.4 |  |  |  |
|  |  |  |  | Filgotinib 200 mg QD | 245 | 42 ± 13.1 | 49.8 | 7.2 ± 6.9 |  |  |  |
| 9.2 |  |  | Induction  (biologic-experienced patients) | Placebo QD | 142 | 44 ± 14.9 | 39.4 | 10.2 ± 8.2 |  |  | UC |
|  |  |  |  | Filgotinib 100 mg QD | 285 | 43 ± 14.3 | 34.7 | 9.7 ± 7.2 |  |  |  |
|  |  |  |  | Filgotinib 200 mg QD | 262 | 43 ± 14.2 | 43.5 | 9.8 ± 7.6 |  |  |  |
| 10.1 | Séverine Vermeire 2023 | NCT02914561 | Induction (biologic-naive patients) | Placebo QD | 239 | 18-65: 229, ≥65: 10 | 54.4 |  | None | 10 | CD |
|  |  |  |  | Filgotinib 100 mg QD | 245 | 233; 12 | 43.3 |  |  |  |  |
|  |  |  |  | Filgotinib 200 mg QD | 223 | 213; 10 | 49.3 |  |  |  |  |
| 10.2 |  |  | Induction  (biologic-experienced patients) | Placebo QD | 231 | 227; 4 | 50.2 |  |  |  | CD |
|  |  |  |  | Filgotinib 100 mg QD | 230 | 220; 10 | 56.1 |  |  |  |  |
|  |  |  |  | Filgotinib 200 mg QD | 204 | 191; 13 | 56.4 |  |  |  |  |
| 11 | Walter Reinisch 2022 | NCT03077412 | Induction | Placebo QD | 15 | 39 ± 11.8 | 26.7 | 7.5 ± 7.9 | None | 24 | CD |
|  |  |  |  | Filgotinib 100 mg QD | 25 | 41 ± 14.0 | 40.0 | 11.9 ± 11.1 |  |  |  |
|  |  |  |  | Filgotinib 200 mg QD | 17 | 39 ± 11.2 | 52.9 | 10.3 ± 8.3 |  |  |  |
| 12 | William J. Sandborn 2022 | NCT02958865 | Induction | Placebo QD | 25 | 42.8 ± 15.45 | 44.0 | 4.1 (0.5–14.0) | ① | 8 | UC |
|  |  |  |  | Ritlecitinib 20 mg QD | 51 | 41.3 ± 14.03 | 33.3 | 4.7 (0.4–20.9) |  |  |  |
|  |  |  |  | Ritlecitinib 70 mg QD | 49 | 40.2 ± 13.31 | 51.0 | 4.4 (0.4–35.2) |  |  |  |
|  |  |  |  | Ritlecitinib 200 mg QD | 50 | 37.3 ± 15.67 | 36.0 | 3.2 (0.5–36.5) |  |  |  |
|  |  |  |  | Brepocitinib 10 mg QD | 48 | 40.8 ± 13.04 | 37.5 | 4.9 (0.4–18.5) |  |  |  |
|  |  |  |  | Brepocitinib 30 mg QD | 47 | 40.9 ± 13.03 | 51.1 | 6.2 (0.2–25.1) |  |  |  |
|  |  |  |  | Brepocitinib 60 mg QD | 47 | 40.3 ± 12.80 | 46.8 | 5.3 (0.3–25.4) |  |  |  |
| 13 | Geert R D'Haens 2021 | NCT03046056 | Induction | Placebo QD | 18 | 45 ± 12.9 | 50.0 | 11.2 ± 9.1 | None | 24 | CD |
|  |  |  |  | Filgotinib 100 mg QD | 32 | 42 ± 12.9 | 71.9 | 14.6 ± 13.7 |  |  |  |
|  |  |  |  | Filgotinib 200 mg QD | 28 | 46 ± 16.3 | 67.9 | 10.6 ± 8.4 |  |  |  |
| 14 | Walter Reinisch 2023 | NCT03201445 | Induction | Placebo QD | 67 | 35 ± 8.4 | 0.0 | 5.5 ± 5.4 | None | 13 | UC+CD |
|  |  |  |  | Filgotinib 200 mg QD | 66 | 36 ± 8.4 | 0.0 | 5.1 ± 5.05 |  |  |  |
| 15 | Edward V Loftus Jr 2022 | NCT03345836 | Induction | Placebo QD | 171 | 37.5 ± 12.1 | 43.9 | 9.8 (0.6–46.1) | None | 12 | CD |
|  |  |  |  | Upadacitinib 45 mg QD | 324 | 38.4 ± 13.7 | 47.8 | 9.3 (0.5–55.2) |  |  |  |
| 16 | Edward V Loftus Jr 2022 | NCT03345849 | Induction | Placebo QD | 176 | 39.3 ± 13.63 | 46.6 | 5.7 (0.3–46.3) | None | 12 | CD |
|  |  |  |  | Upadacitinib 45 mg QD | 350 | 39.7 ± 13.71 | 46.0 | 6.7 (0.1–52.1) |  |  |  |
| 17 | Edward V Loftus Jr 2023 | NCT03345823 | Maintenance | Placebo QD | 165 | 38.1 ± 13.0 | 46.7 | 7.6 (0.3–48.7) | ⑤ | 52 | CD |
|  |  |  |  | Upadacitinib 15 mg QD | 169 | 38.1 ± 13.5 | 39.6 | 7.9 (0.3–40.1) |  |  |  |
|  |  |  |  | Upadacitinib 30 mg QD | 168 | 37.0 ± 13.3 | 44.6 | 7.2 (0.3–44.9) |  |  |  |
| 18 | Theravance Biopharma 2023 | NCT03635112 | Induction | Placebo QD | 38 | 39.5 ± 14.85 | 47.4 |  | None | 12 | CD |
|  |  |  |  | Izencitinib 80 mg QD | 58 | 37.1 ± 12.45 | 48.3 |  |  |  |  |
|  |  |  |  | Izencitinib 200 mg QD | 63 | 40.0 ± 13.64 | 49.2 |  |  |  |  |
| 19 | Silvio Danese 2022 | NCT03653026 | Induction | Placebo QD | 177 | 42.2 ± 14.44 | 37.9 | 7.584 ± 7.6701 | ④⑤ | 8 | UC |
|  |  |  |  | Upadacitinib 45 mg QD | 345 | 42.2 ± 14.73 | 37.4 | 7.273 ± 6.4459 |  |  |  |
| 20 | Baili Chen 2022 | NCT03675477 | Induction | Placebo | 41 | 42.7 ± 12.9 | 53.7 | 5.8 ± 5.2 | None | 8 | UC |
|  |  |  |  | Ivarmacitinib 4 mg QD | 41 | 39.6 ± 10.0 | 31.7 | 5.2 ± 5.2 |  |  |  |
|  |  |  |  | Ivarmacitinib 4 mg BID | 41 | 40.3 ± 12.3 | 36.6 | 5.6 ± 5.6 |  |  |  |
|  |  |  |  | Ivarmacitinib 8 mg QD | 41 | 39.6 ± 13.3 | 34.1 | 5.1 ± 4.2 |  |  |  |
| 21 | Theravance Biopharma 2021 | NCT03758443 | Induction | Placebo QD | 61 | 40.92 ± 15.390 | 44.3 |  | None | 8 | UC |
|  |  |  |  | Izencitinib 20 mg QD | 61 | 38.87 ± 14.576 | 27.9 |  |  |  |  |
|  |  |  |  | Izencitinib 80 mg QD | 59 | 42.02 ± 15.317 | 33.9 |  |  |  |  |
|  |  |  |  | Izencitinib 200 mg QD | 58 | 44.38 ± 14.122 | 50.0 |  |  |  |  |
| 22 | S. Danese 2024 | NCT03934216 | Induction | Placebo QD | 43 | 40.3 ± 13.91 | 32.6 | 7.6 ± 7.29 | ④ | 12 | UC |
|  |  |  |  | Deucravacitinib 6mg BID | 88 | 41.6 ± 14.81 | 45.5 | 8.3 ± 6.47 |  |  |  |
| 23 | Séverine Vermeire 2016 | NCT02048618 | Induction | Placebo QD | 44 | 35.1 ± 11.8 | 59.1 | 6.8 ± 5.7 | None | 10 | CD |
|  |  |  |  | Filgotinib 200 mg QD | 130 | 37.4 ± 11.6 | 54.6 | 8.8 ± 8.5 |  |  |  |
| 24 | Bruce E. Sands 2019 | NCT01959282 | Induction | Placebo | 43 | 40.8 ± 14.63 | 25.6 | 4.0 | None | 8 | UC |
|  |  |  |  | Peficitinib 25 mg QD | 44 | 44.2 ± 12.77 | 43.2 | 6.0 |  |  |  |
|  |  |  |  | Peficitinib 75 mg QD | 44 | 42.7 ± 14.35 | 43.2 | 6.5 |  |  |  |
|  |  |  |  | Peficitinib 150 mg QD | 44 | 38 ± 13.69 | 50.0 | 5.0 |  |  |  |
|  |  |  |  | Peficitinib 75 mg BID | 44 | 40.5 ± 13.22 | 40.9 | 5.0 |  |  |  |
| 25 | Pfizer 2009 | EudraCT: 2008-003571-45 | Induction | Placebo BID | 34 | 35.7 ± 12.7 | 64.7 |  | None | 4 | CD |
|  |  |  |  | Tofacitinib 1 mg BID | 36 | 36.6 ± 12.2 | 30.6 |  |  |  |  |
|  |  |  |  | Tofacitinib 5 mg BID | 34 | 38.7 ± 10.2 | 58.8 |  |  |  |  |
|  |  |  |  | Tofacitinib 15 mg BID | 35 | 38.1 ± 11.7 | 48.6 |  |  |  |  |
| 26 | Pfizer 2010 | EudraCT: 2008-004564-40 | Induction | Placebo BID | 49 | 42.8 ± 14.7 | 53.1 | 14 | ② | 8 | UC |
|  |  |  |  | Tofacitinib 0.5 mg BID | 31 | 43.8 ± 13.4 | 45.2 | 15 |  |  |  |
|  |  |  |  | Tofacitinib 3 mg BID | 33 | 42.5 ± 14.3 | 42.4 | 12 |  |  |  |
|  |  |  |  | Tofacitinib 10 mg BID | 33 | 43.2 ± 12.8 | 36.4 | 8 |  |  |  |
|  |  |  |  | Tofacitinib 15 mg BID | 49 | 41.2 ± 13.5 | 46.9 | 15 |  |  |  |

RCTs: randomized controlled trials; ①:myocardial infarction; ②: ischemic stroke; ③: deep vein thrombosis; ④: pulmonary embolism; ⑤: unclassified vein thrombosis; QD: once daily; BID: twice daily; IBD: inflammatory bowel disease; UC: ulcerative colitis; CD: Crohn’s disease.

**Supplementary table 5-1. Outcomes details of included RCTs.**

| Number | Study | Register number | Intervention | Sample size | Myocardial infarction | Ischemic stroke | Deep vein thrombosis | Pulmonary embolism | Unclassified or other vein thrombosis |
| --- | --- | --- | --- | --- | --- | --- | --- | --- | --- |
| 1 | William J. Sandborn 2013 | NCT00615199 | Placebo BID | 34 | 0/34(0.00%) | 0/34 (0.00%) | 0/34 (0.00%) | 0/34 (0.00%) | 0/34 (0.00%) |
|  |  |  | Tofacitinib 1 mg BID | 36 | 0/36 (0.00%) | 0/36 (0.00%) | 0/36 (0.00%) | 0/36 (0.00%) | 0/36 (0.00%) |
|  |  |  | Tofacitinib 5 mg BID | 34 | 0/34 (0.00%) | 0/34 (0.00%) | 0/34 (0.00%) | 0/34 (0.00%) | 0/34 (0.00%) |
|  |  |  | Tofacitinib 15 mg BID | 35 | 0/35 (0.00%) | 0/35 (0.00%) | 0/35 (0.00%) | 0/35 (0.00%) | 0/35 (0.00%) |
| 2 | William J. Sandborn 2013 | NCT00787202 | Placebo BID | 48 |  | 1/48 (2.08%) | 1/48 (2.08%) |  |  |
|  |  |  | Tofacitinib 0.5 mg BID | 31 |  | 0/31 (0.00%) | 0/31 (0.00%) |  |  |
|  |  |  | Tofacitinib 3 mg BID | 33 |  | 0/33 (0.00%) | 0/33 (0.00%) |  |  |
|  |  |  | Tofacitinib 10 mg BID | 33 |  | 0/33 (0.00%) | 0/33 (0.00%) |  |  |
|  |  |  | Tofacitinib 15 mg BID | 49 |  | 0/49 (0.00%) | 0/49 (0.00%) |  |  |
| 3 | Julian Panés 2017 | NCT01393626 | Placebo BID | 91 | 0/91 (0.00%) | 0/91 (0.00%) | 0/91 (0.00%) | 0/91 (0.00%) | 0/91 (0.00%) |
|  |  |  | Tofacitinib 5 mg BID | 86 | 0/86 (0.00%) | 0/86 (0.00%) | 0/86 (0.00%) | 0/86 (0.00%) | 0/86 (0.00%) |
|  |  |  | Tofacitinib 10 mg BID | 86 | 0/86 (0.00%) | 0/86 (0.00%) | 0/86 (0.00%) | 0/86 (0.00%) | 0/86 (0.00%) |
| 4 | Julian Panés 2016 | NCT01393899 | Placebo BID | 59 | 0/59 (0.00%) | 0/59 (0.00%) | 0/59 (0.00%) | 0/59 (0.00%) | 0/59 (0.00%) |
|  |  |  | Tofacitinib 5 mg BID | 60 | 0/60 (0.00%) | 0/60 (0.00%) | 0/60 (0.00%) | 0/60 (0.00%) | 0/60 (0.00%) |
|  |  |  | Tofacitinib 10 mg BID | 61 | 0/61 (0.00%) | 0/61 (0.00%) | 0/61 (0.00%) | 0/61 (0.00%) | 0/61 (0.00%) |
| 5 | William J. Sandborn, 2017 | NCT01458574 | Placebo BID | 198 | 0/198 (0.00%) |  |  |  | 1/198 (0.51%) |
|  |  |  | Tofacitinib 5 mg BID | 198 | 1/198 (0.51%) |  |  |  | 0/198 (0.00%) |
|  |  |  | Tofacitinib 10 mg BID | 196 | 0/196 (0.00%) |  |  |  | 0/196 (0.00%) |
| 6 | William J. Sandborn, 2016 | NCT01458951 | Placebo BID | 112 | 0/112 (0.00%) | 0/112 (0.00%) | 0/112 (0.00%) | 0/112 (0.00%) | 0/112 (0.00%) |
|  |  |  | Tofacitinib 10 mg BID | 429 | 0/429 (0.00%) | 0/429 (0.00%) | 0/429 (0.00%) | 0/429 (0.00%) | 0/429 (0.00%) |
| 7 | William J. Sandborn 2016 | NCT01465763 | Placebo BID | 122 |  |  |  | 1/122 (0.82%) |  |
|  |  |  | Tofacitinib 10 mg BID | 476 |  |  |  | 0/476 (0.00%) |  |
| 8.1 | William J. Sandborn 2022 | NCT02819635 | Placebo QD | 46 |  |  | 0/46 (0.00%) | 0/46 (0.00%) |  |
|  |  |  | Upadacitinib 15 mg QD | 49 |  |  | 0/49 (0.00%) | 0/49 (0.00%) |  |
|  |  |  | Upadacitinib 30 mg QD | 117 |  |  | 0/117 (0.00%) | 0/117 (0.00%) |  |
|  |  |  | Upadacitinib 45 mg QD | 123 |  |  | 1/123 (0.81%) | 1/123 (0.81%) |  |
| 8.2 |  |  | Placebo QD | 155 | 0/155 (0.00%) | 0/155 (0.00%) | 0/155 (0.00%) | 0/155 (0.00%) | 0/155 (0.00%) |
|  |  |  | Upadacitinib 45 mg QD | 319 | 0/319 (0.00%) | 0/319 (0.00%) | 0/319 (0.00%) | 0/319 (0.00%) | 0/319 (0.00%) |
| 8.3 |  |  | Placebo QD | 149 | 1/149 (0.67%) |  | 0/149 (0.00%) |  |  |
|  |  |  | Upadacitinib 15 mg QD | 148 | 0/148 (0.00%) |  | 0/148 (0.00%) |  |  |
|  |  |  | Upadacitinib 30 mg QD | 154 | 0/154 (0.00%) |  | 2/154 (1.30%) |  |  |
| 9.1 | Brian G Feagan 2021 | NCT02914522 | Placebo QD | 137 |  |  |  | 0/137 (0.00%) |  |
|  |  |  | Filgotinib 100 mg QD | 277 |  |  |  | 0/277 (0.00%) |  |
|  |  |  | Filgotinib 200 mg QD | 245 |  |  |  | 0/245 (0.00%) |  |
| 9.2 |  |  | Placebo QD | 142 |  |  |  | 0/142 (0.00%) |  |
|  |  |  | Filgotinib 100 mg QD | 285 |  |  |  | 0/285 (0.00%) |  |
|  |  |  | Filgotinib 200 mg QD | 262 |  |  |  | 1/262 (0.38%) |  |
| 10.1 | Séverine Vermeire 2023 | NCT02914561 | Placebo QD | 237 | 0/237 (0.00%) | 0/237 (0.00%) | 0/237 (0.00%) | 0/237 (0.00%) | 0/237 (0.00%) |
|  |  |  | Filgotinib 100 mg QD | 245 | 0/245 (0.00%) | 0/245 (0.00%) | 0/245 (0.00%) | 0/245 (0.00%) | 0/245 (0.00%) |
|  |  |  | Filgotinib 200 mg QD | 222 | 0/222 (0.00%) | 0/222 (0.00%) | 0/222 (0.00%) | 0/222 (0.00%) | 0/222 (0.00%) |
| 10.2 |  |  | Placebo QD | 229 | 0/229 (0.00%) | 0/229 (0.00%) | 0/229 (0.00%) | 0/229 (0.00%) | 0/229 (0.00%) |
|  |  |  | Filgotinib 100 mg QD | 228 | 0/228 (0.00%) | 0/228 (0.00%) | 0/228 (0.00%) | 0/228 (0.00%) | 0/228 (0.00%) |
|  |  |  | Filgotinib 200 mg QD | 202 | 0/202 (0.00%) | 0/202 (0.00%) | 0/202 (0.00%) | 0/202 (0.00%) | 0/202 (0.00%) |
| 11 | Walter Reinisch 2022 | NCT03077412 | Placebo QD | 15 | 0/15 (0.00%) | 0/15 (0.00%) | 0/15 (0.00%) | 0/15 (0.00%) | 0/15 (0.00%) |
|  |  |  | Filgotinib 100 mg QD | 25 | 0/25 (0.00%) | 0/25 (0.00%) | 0/25 (0.00%) | 0/25 (0.00%) | 0/25 (0.00%) |
|  |  |  | Filgotinib 200 mg QD | 17 | 0/17 (0.00%) | 0/17 (0.00%) | 0/17 (0.00%) | 0/17 (0.00%) | 0/17 (0.00%) |
| 12 | William J. Sandborn 2022 | NCT02958865 | Placebo QD | 25 | 0/25 (0.00%) |  |  |  |  |
|  |  |  | Ritlecitinib 20 mg QD | 51 | 1/51 (1.96%) |  |  |  |  |
|  |  |  | Ritlecitinib 70 mg QD | 49 | 0/49 (0.00%) |  |  |  |  |
|  |  |  | Ritlecitinib 200 mg QD | 50 | 0/50 (0.00%) |  |  |  |  |
|  |  |  | Brepocitinib 10 mg QD | 48 | 0/48 (0.00%) |  |  |  |  |
|  |  |  | Brepocitinib 30 mg QD | 47 | 0/47 (0.00%) |  |  |  |  |
|  |  |  | Brepocitinib 60 mg QD | 47 | 0/47 (0.00%) |  |  |  |  |
| 13 | Geert R D'Haens 2021 | NCT03046056 | Placebo QD | 18 | 0/18 (0.00%) | 0/18 (0.00%) | 0/18 (0.00%) | 0/18 (0.00%) | 0/18 (0.00%) |
|  |  |  | Filgotinib 100 mg QD | 32 | 0/32 (0.00%) | 0/32 (0.00%) | 0/32 (0.00%) | 0/32 (0.00%) | 0/32 (0.00%) |
|  |  |  | Filgotinib 200 mg QD | 28 | 0/28 (0.00%) | 0/28 (0.00%) | 0/28 (0.00%) | 0/28 (0.00%) | 0/28 (0.00%) |
| 14 | Walter Reinisch 2023 | NCT03201445 | Placebo QD | 67 | 0/67 (0.00%) | 0/67 (0.00%) | 0/67 (0.00%) | 0/67 (0.00%) | 0/67 (0.00%) |
|  |  |  | Filgotinib 200 mg QD | 66 | 0/66 (0.00%) | 0/66 (0.00%) | 0/66 (0.00%) | 0/66 (0.00%) | 0/66 (0.00%) |
| 15 | Edward V Loftus Jr 2022 | NCT03345836 | Placebo QD | 171 | 0/171 (0.00%) | 0/171 (0.00%) | 0/171 (0.00%) | 0/171 (0.00%) | 0/171 (0.00%) |
|  |  |  | Upadacitinib 45 mg QD | 324 | 0/324 (0.00%) | 0/324 (0.00%) | 0/324 (0.00%) | 0/324 (0.00%) | 0/324 (0.00%) |
| 16 | Edward V Loftus Jr 2022 | NCT03345849 | Placebo QD | 176 | 0/176 (0.00%) | 0/176 (0.00%) | 0/176 (0.00%) | 0/176 (0.00%) | 0/176 (0.00%) |
|  |  |  | Upadacitinib 45 mg QD | 350 | 0/350 (0.00%) | 0/350 (0.00%) | 0/350 (0.00%) | 0/350 (0.00%) | 0/350 (0.00%) |
| 17 | Edward V Loftus Jr 2023 | NCT03345823 | Placebo QD | 223 |  |  |  |  | 0/223 (0.00%) |
|  |  |  | Upadacitinib 15 mg QD | 221 |  |  |  |  | 0/221 (0.00%) |
|  |  |  | Upadacitinib 30 mg QD | 229 |  |  |  |  | 1/229 (0.44%) |
| 18 | Theravance Biopharma 2023 | NCT03635112 | Placebo QD | 38 | 0/38 (0.00%) | 0/38 (0.00%) | 0/38 (0.00%) | 0/38 (0.00%) | 0/38 (0.00%) |
|  |  |  | Izencitinib 80 mg QD | 58 | 0/58 (0.00%) | 0/58 (0.00%) | 0/58 (0.00%) | 0/58 (0.00%) | 0/58 (0.00%) |
|  |  |  | Izencitinib 200 mg QD | 63 | 0/63 (0.00%) | 0/63 (0.00%) | 0/63 (0.00%) | 0/63 (0.00%) | 0/63 (0.00%) |
| 19 | Silvio Danese 2022 | NCT03653026 | Placebo QD | 177 |  |  |  | 1/177 (0.56%) | 1/177 (0.56%) |
|  |  |  | Upadacitinib 45 mg QD | 344 |  |  |  | 0/344 (0.00%) | 0/344 (0.00%) |
| 20 | Baili Chen 2022 | NCT03675477 | Placebo | 41 | 0/41 (0.00%) | 0/41 (0.00%) | 0/41 (0.00%) | 0/41 (0.00%) | 0/41 (0.00%) |
|  |  |  | Ivarmacitinib 4 mg QD | 41 | 0/41 (0.00%) | 0/41 (0.00%) | 0/41 (0.00%) | 0/41 (0.00%) | 0/41 (0.00%) |
|  |  |  | Ivarmacitinib 4 mg BID | 41 | 0/41 (0.00%) | 0/41 (0.00%) | 0/41 (0.00%) | 0/41 (0.00%) | 0/41 (0.00%) |
|  |  |  | Ivarmacitinib 8 mg QD | 41 | 0/41 (0.00%) | 0/41 (0.00%) | 0/41 (0.00%) | 0/41 (0.00%) | 0/41 (0.00%) |
| 21 | Theravance Biopharma 2021 | NCT03758443 | Placebo QD | 61 | 0/61 (0.00%) | 0/61 (0.00%) | 0/61 (0.00%) | 0/61 (0.00%) | 0/61 (0.00%) |
|  |  |  | Izencitinib 20 mg QD | 61 | 0/61 (0.00%) | 0/61 (0.00%) | 0/61 (0.00%) | 0/61 (0.00%) | 0/61 (0.00%) |
|  |  |  | Izencitinib 80 mg QD | 59 | 0/59 (0.00%) | 0/59 (0.00%) | 0/59 (0.00%) | 0/59 (0.00%) | 0/59 (0.00%) |
|  |  |  | Izencitinib 200 mg QD | 58 | 0/58 (0.00%) | 0/58 (0.00%) | 0/58 (0.00%) | 0/58 (0.00%) | 0/58 (0.00%) |
| 22 | S. Danese 2024 | NCT03934216 | Placebo QD | 10 |  |  |  | 1/10 (10.00%) |  |
|  |  |  | Deucravacitinib 6 mg BID | 87 |  |  |  | 0/87 (0.00%) |  |
| 23 | Séverine Vermeire 2016 | NCT02048618 | Placebo QD | 44 | 0/44 (0.00%) | 0/44 (0.00%) | 0/44 (0.00%) | 0/44 (0.00%) | 0/44 (0.00%) |
|  |  |  | Filgotinib 200 mg QD | 130 | 0/130 (0.00%) | 0/130 (0.00%) | 0/130 (0.00%) | 0/130 (0.00%) | 0/130 (0.00%) |
| 24 | Bruce E. Sands 2019 | NCT01959282 | Placebo | 43 | 0/43 (0.00%) | 0/43 (0.00%) | 0/43 (0.00%) | 0/43 (0.00%) | 0/43 (0.00%) |
|  |  |  | Peficitinib 25 mg QD | 44 | 0/44 (0.00%) | 0/44 (0.00%) | 0/44 (0.00%) | 0/44 (0.00%) | 0/44 (0.00%) |
|  |  |  | Peficitinib 75 mg QD | 44 | 0/44 (0.00%) | 0/44 (0.00%) | 0/44 (0.00%) | 0/44 (0.00%) | 0/44 (0.00%) |
|  |  |  | Peficitinib 150 mg QD | 44 | 0/44 (0.00%) | 0/44 (0.00%) | 0/44 (0.00%) | 0/44 (0.00%) | 0/44 (0.00%) |
|  |  |  | Peficitinib 75 mg BID | 44 | 0/44 (0.00%) | 0/44 (0.00%) | 0/44 (0.00%) | 0/44 (0.00%) | 0/44 (0.00%) |
| 25 | Pfizer 2009 | EudraCT: 2008-003571-45 | Placebo BID | 34 | 0/34 (0.00%) | 0/34 (0.00%) | 0/34 (0.00%) | 0/34 (0.00%) | 0/34 (0.00%) |
|  |  |  | Tofacitinib 1 mg BID | 36 | 0/36 (0.00%) | 0/36 (0.00%) | 0/36 (0.00%) | 0/36 (0.00%) | 0/36 (0.00%) |
|  |  |  | Tofacitinib 5 mg BID | 34 | 0/34 (0.00%) | 0/34 (0.00%) | 0/34 (0.00%) | 0/34 (0.00%) | 0/34 (0.00%) |
|  |  |  | Tofacitinib 15 mg BID | 35 | 0/35 (0.00%) | 0/35 (0.00%) | 0/35 (0.00%) | 0/35 (0.00%) | 0/35 (0.00%) |
| 26 | Pfizer 2010 | EudraCT: 2008-004564-40 | Placebo BID | 48 |  | 1/48 (2.10%) |  |  |  |
|  |  |  | Tofacitinib 0.5 mg BID | 31 |  | 0/31 (0.00%) |  |  |  |
|  |  |  | Tofacitinib 3 mg BID | 33 |  | 0/33 (0.00%) |  |  |  |
|  |  |  | Tofacitinib 10 mg BID | 33 |  | 0/33 (0.00%) |  |  |  |
|  |  |  | Tofacitinib 15 mg BID | 49 |  | 0/49 (0.00%) |  |  |  |

RCTs: randomized controlled trials; QD: once daily; BID: twice daily.

**Supplementary table 6 Summary of key characteristics of included RCTs.**

| Key characteristic | Minimum | First Quartile | Median | Mean | Third Quartile | Maximum |
| --- | --- | --- | --- | --- | --- | --- |
| Without dose consideration | | | | | | |
| Mean age | 35.00 | 39.00 | 40.92 | 40.52 | 42.50 | 45.00 |
| Proportion of female | 0.00 | 38.90 | 44.60 | 43.89 | 48.80 | 70.00 |
| Disease duration | 4.00 | 6.00 | 7.20 | 7.84 | 9.50 | 14.00 |
| Study duration | 4.00 | 8.00 | 10.00 | 14.72 | 12.00 | 52.00 |
| With dose consideration | | | | | | |
| Mean age | 35.00 | 39.00 | 41.00 | 40.69 | 42.70 | 47.00 |
| Proportion of female | 0.00 | 37.98 | 44.25 | 44.28 | 50.00 | 71.90 |
| Disease duration | 4.000 | 6.200 | 7.592 | 8.309 | 10.525 | 15.000 |
| Study duration | 4.00 | 8.00 | 9.00 | 14.95 | 12.00 | 52.00 |

RCTs: randomized controlled trials.

**Supplementary table 7 The Risk-of-bias assessment for the direct evidence (without dose consideration).**

| Comparison | No. of total RCTs (%) | No. of low RoB RCTs (%) | No. of moderate RoB RCTs (%) | No. of high RoB RCTs (%) | Risk-of-bias assessment |
| --- | --- | --- | --- | --- | --- |
| Placebo vs. Brepocitinib | 1 | 1 (100%) | 0 | 0 | No downgrade |
| Ritlecitinib vs. Brepocitinib | 1 | 1 (100%) | 0 | 0 | No downgrade |
| Deucravacitinib vs. Placebo | 1 | 0 | 1 (100%) | 0 | Downgrade |
| Filgotinib vs. Placebo | 6 | 4 (66.67%) | 1 (16.67%) | 1 (16.67%) | No downgrade |
| Ivarmacitinib vs. Placebo | 1 | 1 (100%) | 0 | 0 | No downgrade |
| Izencitinib vs. Placebo | 2 | 0 | 2 (100%) | 0 | Downgrade |
| Peficitinib vs. Placebo | 1 | 1 (100%) | 0 | 0 | No downgrade |
| Placebo vs. Ritlecitinib | 1 | 1 (100%) | 0 | 0 | No downgrade |
| Placebo vs. Tofacitinib | 9 | 7 (77.78%) | 2 (22.22%) | 0 | No downgrade |
| Placebo vs. Upadacitinib | 5 | 5 (100%) | 0 | 0 | No downgrade |

No.: numero = number; RoB: Risk of bias; RCT: randomized controlled trials. The Risk-of-bias will be downgraded by one level when the contributions from low RoB comparisons were less than 30% and contributions from moderate RoB comparisons were 70% or greater.

**Supplementary table 8-1-1 Certainty of direct evidence assessment for MACE (without dose consideration).**

| Comparison | Risk of bias | Inconsistency | Indirectness | Publication bias | Preliminary certainty rating |
| --- | --- | --- | --- | --- | --- |
| Placebo vs. Brepocitinib | No downgrade | No downgrade | Downgrade | No downgrade | Moderate ⨁⨁⨁◯ |
| Ritlecitinib vs. Brepocitinib | No downgrade | No downgrade | Downgrade | No downgrade | Moderate ⨁⨁⨁◯ |
| Deucravacitinib vs. Placebo | Downgrade | No downgrade | Downgrade | No downgrade | Low ⨁⨁◯◯ |
| Filgotinib vs. Placebo | No downgrade | No downgrade | Downgrade | No downgrade | Moderate ⨁⨁⨁◯ |
| Ivarmacitinib vs. Placebo | No downgrade | No downgrade | Downgrade | No downgrade | Moderate ⨁⨁⨁◯ |
| Izencitinib vs. Placebo | Downgrade | No downgrade | No downgrade | No downgrade | Moderate ⨁⨁⨁◯ |
| Peficitinib vs. Placebo | No downgrade | No downgrade | Downgrade | No downgrade | Moderate ⨁⨁⨁◯ |
| Placebo vs. Ritlecitinib | No downgrade | No downgrade | Downgrade | No downgrade | Moderate ⨁⨁⨁◯ |
| Placebo vs. Tofacitinib | No downgrade | No downgrade | Downgrade | No downgrade | Moderate ⨁⨁⨁◯ |
| Placebo vs. Upadacitinib | No downgrade | No downgrade | Downgrade | No downgrade | Moderate ⨁⨁⨁◯ |

MACE: major adverse cardiovascular events.

**Supplementary table 8-1-2 Certainty of direct evidence assessment for VTE (without dose consideration).**

| Comparison | Risk of bias | Inconsistency | Indirectness | Publication bias | Preliminary certainty rating |
| --- | --- | --- | --- | --- | --- |
| Placebo vs. Brepocitinib | No downgrade | No downgrade | Downgrade | No downgrade | Moderate ⨁⨁⨁◯ |
| Ritlecitinib vs. Brepocitinib | No downgrade | No downgrade | Downgrade | No downgrade | Moderate ⨁⨁⨁◯ |
| Deucravacitinib vs. Placebo | Downgrade | No downgrade | Downgrade | No downgrade | Low ⨁⨁◯◯ |
| Filgotinib vs. Placebo | No downgrade | No downgrade | Downgrade | No downgrade | Moderate ⨁⨁⨁◯ |
| Ivarmacitinib vs. Placebo | No downgrade | No downgrade | Downgrade | No downgrade | Moderate ⨁⨁⨁◯ |
| Izencitinib vs. Placebo | Downgrade | No downgrade | No downgrade | No downgrade | Moderate ⨁⨁⨁◯ |
| Peficitinib vs. Placebo | No downgrade | No downgrade | Downgrade | No downgrade | Moderate ⨁⨁⨁◯ |
| Placebo vs. Ritlecitinib | No downgrade | No downgrade | Downgrade | No downgrade | Moderate ⨁⨁⨁◯ |
| Placebo vs. Tofacitinib | No downgrade | No downgrade | Downgrade | No downgrade | Moderate ⨁⨁⨁◯ |
| Placebo vs. Upadacitinib | No downgrade | No downgrade | Downgrade | No downgrade | Moderate ⨁⨁⨁◯ |

VTE: venous thromboembolism events.

**Supplementary table 8-1-3 Certainty of direct evidence assessment for CVE (without dose consideration).**

| Comparison | Risk of bias | Inconsistency | Indirectness | Publication bias | Preliminary certainty rating |
| --- | --- | --- | --- | --- | --- |
| Placebo vs. Brepocitinib | No downgrade | No downgrade | Downgrade | No downgrade | Moderate ⨁⨁⨁◯ |
| Ritlecitinib vs. Brepocitinib | No downgrade | No downgrade | Downgrade | No downgrade | Moderate ⨁⨁⨁◯ |
| Deucravacitinib vs. Placebo | Downgrade | No downgrade | Downgrade | No downgrade | Low ⨁⨁◯◯ |
| Filgotinib vs. Placebo | No downgrade | No downgrade | Downgrade | No downgrade | Moderate ⨁⨁⨁◯ |
| Ivarmacitinib vs. Placebo | No downgrade | No downgrade | Downgrade | No downgrade | Moderate ⨁⨁⨁◯ |
| Izencitinib vs. Placebo | Downgrade | No downgrade | No downgrade | No downgrade | Moderate ⨁⨁⨁◯ |
| Peficitinib vs. Placebo | No downgrade | No downgrade | Downgrade | No downgrade | Moderate ⨁⨁⨁◯ |
| Placebo vs. Ritlecitinib | No downgrade | No downgrade | Downgrade | No downgrade | Moderate ⨁⨁⨁◯ |
| Placebo vs. Tofacitinib | No downgrade | No downgrade | Downgrade | No downgrade | Moderate ⨁⨁⨁◯ |
| Placebo vs. Upadacitinib | No downgrade | No downgrade | Downgrade | No downgrade | Moderate ⨁⨁⨁◯ |

CVE: cardiovascular events.

**Supplementary table 8-2-1 Certainty of indirect evidence assessment for MACE (without dose consideration).**

| Comparison | First order loop | Start rating | | | Intransitivity | Preliminary certainty rating |
| --- | --- | --- | --- | --- | --- | --- |
|  |  | A→B Direct preliminary certainty rating | B→C Direct Preliminary certainty rating | Lowest certainty of the first loop components |  |  |
| Deucravacitinib vs. Brepocitinib | Deucravacitinib vs. Placebo vs. Brepocitinib | Low ⨁⨁◯◯ | Moderate ⨁⨁⨁◯ | Low ⨁⨁◯◯ | No downgrade | Low ⨁⨁◯◯ |
| Filgotinib vs. Brepocitinib | Filgotinib vs. Placebo vs. Brepocitinib | Moderate ⨁⨁⨁◯ | Moderate ⨁⨁⨁◯ | Moderate ⨁⨁⨁◯ | Downgrade | Low ⨁⨁◯◯ |
| Ivarmacitinib vs. Brepocitinib | Ivarmacitinib vs. Placebo vs. Brepocitinib | Moderate ⨁⨁⨁◯ | Moderate ⨁⨁⨁◯ | Moderate ⨁⨁⨁◯ | No downgrade | Moderate ⨁⨁⨁◯ |
| Izencitinib vs. Brepocitinib | Izencitinib vs. Placebo vs. Brepocitinib | Moderate ⨁⨁⨁◯ | Moderate ⨁⨁⨁◯ | Moderate ⨁⨁⨁◯ | Downgrade | Low ⨁⨁◯◯ |
| Peficitinib vs. Brepocitinib | Peficitinib vs. Placebo vs. Brepocitinib | Moderate ⨁⨁⨁◯ | Moderate ⨁⨁⨁◯ | Moderate ⨁⨁⨁◯ | No downgrade | Moderate ⨁⨁⨁◯ |
| Tofacitinib vs. Brepocitinib | Tofacitinib vs. Placebo vs. Brepocitinib | Moderate ⨁⨁⨁◯ | Moderate ⨁⨁⨁◯ | Moderate ⨁⨁⨁◯ | Downgrade | Low ⨁⨁◯◯ |
| Upadacitinib vs. Brepocitinib | Upadacitinib vs. Placebo vs. Brepocitinib | Moderate ⨁⨁⨁◯ | Moderate ⨁⨁⨁◯ | Moderate ⨁⨁⨁◯ | Downgrade | Low ⨁⨁◯◯ |
| Deucravacitinib vs. Filgotinib | Deucravacitinib vs. Placebo vs. Filgotinib | Low ⨁⨁◯◯ | Moderate ⨁⨁⨁◯ | Low ⨁⨁◯◯ | Downgrade | Very low ⨁◯◯◯ |
| Deucravacitinib vs. Ivarmacitinib | Deucravacitinib vs. Placebo vs. Ivarmacitinib | Low ⨁⨁◯◯ | Moderate ⨁⨁⨁◯ | Low ⨁⨁◯◯ | No downgrade | Low ⨁⨁◯◯ |
| Deucravacitinib vs. Izencitinib | Deucravacitinib vs. Placebo vs. Izencitinib | Low ⨁⨁◯◯ | Moderate ⨁⨁⨁◯ | Low ⨁⨁◯◯ | Downgrade | Very low ⨁◯◯◯ |
| Deucravacitinib vs. Peficitinib | Deucravacitinib vs. Placebo vs. Peficitinib | Low ⨁⨁◯◯ | Moderate ⨁⨁⨁◯ | Low ⨁⨁◯◯ | No downgrade | Low ⨁⨁◯◯ |
| Deucravacitinib vs. Ritlecitinib | Deucravacitinib vs. Placebo vs. Ritlecitinib | Low ⨁⨁◯◯ | Moderate ⨁⨁⨁◯ | Low ⨁⨁◯◯ | No downgrade | Low ⨁⨁◯◯ |
| Deucravacitinib vs. Tofacitinib | Deucravacitinib vs. Placebo vs. Tofacitinib | Low ⨁⨁◯◯ | Moderate ⨁⨁⨁◯ | Low ⨁⨁◯◯ | Downgrade | Very low ⨁◯◯◯ |
| Deucravacitinib vs. Upadacitinib | Deucravacitinib vs. Placebo vs. Upadacitinib | Low ⨁⨁◯◯ | Moderate ⨁⨁⨁◯ | Low ⨁⨁◯◯ | Downgrade | Very low ⨁◯◯◯ |
| Filgotinib vs. Ivarmacitinib | Filgotinib vs. Placebo vs. Ivarmacitinib | Moderate ⨁⨁⨁◯ | Moderate ⨁⨁⨁◯ | Moderate ⨁⨁⨁◯ | Downgrade | Low ⨁⨁◯◯ |
| Filgotinib vs. Izencitinib | Filgotinib vs. Placebo vs. Izencitinib | Moderate ⨁⨁⨁◯ | Moderate ⨁⨁⨁◯ | Moderate ⨁⨁⨁◯ | Downgrade | Low ⨁⨁◯◯ |
| Filgotinib vs. Peficitinib | Filgotinib vs. Placebo vs. Peficitinib | Moderate ⨁⨁⨁◯ | Moderate ⨁⨁⨁◯ | Moderate ⨁⨁⨁◯ | Downgrade | Low ⨁⨁◯◯ |
| Filgotinib vs. Ritlecitinib | Filgotinib vs. Placebo vs. Ritlecitinib | Moderate ⨁⨁⨁◯ | Moderate ⨁⨁⨁◯ | Moderate ⨁⨁⨁◯ | Downgrade | Low ⨁⨁◯◯ |
| Filgotinib vs. Tofacitinib | Filgotinib vs. Placebo vs. Tofacitinib | Moderate ⨁⨁⨁◯ | Moderate ⨁⨁⨁◯ | Moderate ⨁⨁⨁◯ | Downgrade | Low ⨁⨁◯◯ |
| Filgotinib vs. Upadacitinib | Filgotinib vs. Placebo vs. Upadacitinib | Moderate ⨁⨁⨁◯ | Moderate ⨁⨁⨁◯ | Moderate ⨁⨁⨁◯ | Downgrade | Low ⨁⨁◯◯ |
| Ivarmacitinib vs. Izencitinib | Ivarmacitinib vs. Placebo vs. Izencitinib | Moderate ⨁⨁⨁◯ | Moderate ⨁⨁⨁◯ | Moderate ⨁⨁⨁◯ | Downgrade | Low ⨁⨁◯◯ |
| Ivarmacitinib vs. Peficitinib | Ivarmacitinib vs. Placebo vs. Peficitinib | Moderate ⨁⨁⨁◯ | Moderate ⨁⨁⨁◯ | Moderate ⨁⨁⨁◯ | Downgrade | Low ⨁⨁◯◯ |
| Ivarmacitinib vs. Ritlecitinib | Ivarmacitinib vs. Placebo vs. Ritlecitinib | Moderate ⨁⨁⨁◯ | Moderate ⨁⨁⨁◯ | Moderate ⨁⨁⨁◯ | No downgrade | Moderate ⨁⨁⨁◯ |
| Ivarmacitinib vs. Tofacitinib | Ivarmacitinib vs. Placebo vs. Tofacitinib | Moderate ⨁⨁⨁◯ | Moderate ⨁⨁⨁◯ | Moderate ⨁⨁⨁◯ | Downgrade | Low ⨁⨁◯◯ |
| Ivarmacitinib vs. Upadacitinib | Ivarmacitinib vs. Placebo vs. Upadacitinib | Moderate ⨁⨁⨁◯ | Moderate ⨁⨁⨁◯ | Moderate ⨁⨁⨁◯ | Downgrade | Low ⨁⨁◯◯ |
| Izencitinib vs. Peficitinib | Izencitinib vs. Placebo vs. Peficitinib | Moderate ⨁⨁⨁◯ | Moderate ⨁⨁⨁◯ | Moderate ⨁⨁⨁◯ | Downgrade | Low ⨁⨁◯◯ |
| Izencitinib vs. Ritlecitinib | Izencitinib vs. Placebo vs. Ritlecitinib | Moderate ⨁⨁⨁◯ | Moderate ⨁⨁⨁◯ | Moderate ⨁⨁⨁◯ | Downgrade | Low ⨁⨁◯◯ |
| Izencitinib vs. Tofacitinib | Izencitinib vs. Placebo vs. Tofacitinib | Moderate ⨁⨁⨁◯ | Moderate ⨁⨁⨁◯ | Moderate ⨁⨁⨁◯ | Downgrade | Low ⨁⨁◯◯ |
| Izencitinib vs. Upadacitinib | Izencitinib vs. Placebo vs. Upadacitinib | Moderate ⨁⨁⨁◯ | Moderate ⨁⨁⨁◯ | Moderate ⨁⨁⨁◯ | Downgrade | Low ⨁⨁◯◯ |
| Peficitinib vs. Ritlecitinib | Peficitinib vs. Placebo vs. Ritlecitinib | Moderate ⨁⨁⨁◯ | Moderate ⨁⨁⨁◯ | Moderate ⨁⨁⨁◯ | No downgrade | Moderate ⨁⨁⨁◯ |
| Peficitinib vs. Tofacitinib | Peficitinib vs. Placebo vs. Tofacitinib | Moderate ⨁⨁⨁◯ | Moderate ⨁⨁⨁◯ | Moderate ⨁⨁⨁◯ | Downgrade | Low ⨁⨁◯◯ |
| Peficitinib vs. Upadacitinib | Peficitinib vs. Placebo vs. Upadacitinib | Moderate ⨁⨁⨁◯ | Moderate ⨁⨁⨁◯ | Moderate ⨁⨁⨁◯ | Downgrade | Low ⨁⨁◯◯ |
| Ritlecitinib vs. Tofacitinib | Ritlecitinib vs. Placebo vs. Tofacitinib | Moderate ⨁⨁⨁◯ | Moderate ⨁⨁⨁◯ | Moderate ⨁⨁⨁◯ | Downgrade | Low ⨁⨁◯◯ |
| Ritlecitinib vs. Upadacitinib | Ritlecitinib vs. Placebo vs. Upadacitinib | Moderate ⨁⨁⨁◯ | Moderate ⨁⨁⨁◯ | Moderate ⨁⨁⨁◯ | Downgrade | Low ⨁⨁◯◯ |
| Tofacitinib vs. Upadacitinib | Tofacitinib vs. Placebo vs. Upadacitinib | Moderate ⨁⨁⨁◯ | Moderate ⨁⨁⨁◯ | Moderate ⨁⨁⨁◯ | No downgrade | Moderate ⨁⨁⨁◯ |

MACE: major adverse cardiovascular events.

**Supplementary table 8-2-2 Certainty of indirect evidence assessment for VTE (without dose consideration).**

| Comparison | First order loop | Start rating | | | Intransitivity | Preliminary certainty rating |
| --- | --- | --- | --- | --- | --- | --- |
|  |  | A→B Direct preliminary certainty rating | B→C Direct Preliminary certainty rating | Lowest certainty of the first loop components |  |  |
| Deucravacitinib vs. Brepocitinib | Deucravacitinib vs. Placebo vs. Brepocitinib | Low ⨁⨁◯◯ | Moderate ⨁⨁⨁◯ | Low ⨁⨁◯◯ | No downgrade | Low ⨁⨁◯◯ |
| Filgotinib vs. Brepocitinib | Filgotinib vs. Placebo vs. Brepocitinib | Moderate ⨁⨁⨁◯ | Moderate ⨁⨁⨁◯ | Moderate ⨁⨁⨁◯ | Downgrade | Low ⨁⨁◯◯ |
| Ivarmacitinib vs. Brepocitinib | Ivarmacitinib vs. Placebo vs. Brepocitinib | Moderate ⨁⨁⨁◯ | Moderate ⨁⨁⨁◯ | Moderate ⨁⨁⨁◯ | No downgrade | Moderate ⨁⨁⨁◯ |
| Izencitinib vs. Brepocitinib | Izencitinib vs. Placebo vs. Brepocitinib | Moderate ⨁⨁⨁◯ | Moderate ⨁⨁⨁◯ | Moderate ⨁⨁⨁◯ | Downgrade | Low ⨁⨁◯◯ |
| Peficitinib vs. Brepocitinib | Peficitinib vs. Placebo vs. Brepocitinib | Moderate ⨁⨁⨁◯ | Moderate ⨁⨁⨁◯ | Moderate ⨁⨁⨁◯ | No downgrade | Moderate ⨁⨁⨁◯ |
| Tofacitinib vs. Brepocitinib | Tofacitinib vs. Placebo vs. Brepocitinib | Moderate ⨁⨁⨁◯ | Moderate ⨁⨁⨁◯ | Moderate ⨁⨁⨁◯ | Downgrade | Low ⨁⨁◯◯ |
| Upadacitinib vs. Brepocitinib | Upadacitinib vs. Placebo vs. Brepocitinib | Moderate ⨁⨁⨁◯ | Moderate ⨁⨁⨁◯ | Moderate ⨁⨁⨁◯ | Downgrade | Low ⨁⨁◯◯ |
| Deucravacitinib vs. Filgotinib | Deucravacitinib vs. Placebo vs. Filgotinib | Low ⨁⨁◯◯ | Moderate ⨁⨁⨁◯ | Low ⨁⨁◯◯ | Downgrade | Very low ⨁◯◯◯ |
| Deucravacitinib vs. Ivarmacitinib | Deucravacitinib vs. Placebo vs. Ivarmacitinib | Low ⨁⨁◯◯ | Moderate ⨁⨁⨁◯ | Low ⨁⨁◯◯ | No downgrade | Low ⨁⨁◯◯ |
| Deucravacitinib vs. Izencitinib | Deucravacitinib vs. Placebo vs. Izencitinib | Low ⨁⨁◯◯ | Moderate ⨁⨁⨁◯ | Low ⨁⨁◯◯ | Downgrade | Very low ⨁◯◯◯ |
| Deucravacitinib vs. Peficitinib | Deucravacitinib vs. Placebo vs. Peficitinib | Low ⨁⨁◯◯ | Moderate ⨁⨁⨁◯ | Low ⨁⨁◯◯ | No downgrade | Low ⨁⨁◯◯ |
| Deucravacitinib vs. Ritlecitinib | Deucravacitinib vs. Placebo vs. Ritlecitinib | Low ⨁⨁◯◯ | Moderate ⨁⨁⨁◯ | Low ⨁⨁◯◯ | No downgrade | Low ⨁⨁◯◯ |
| Deucravacitinib vs. Tofacitinib | Deucravacitinib vs. Placebo vs. Tofacitinib | Low ⨁⨁◯◯ | Moderate ⨁⨁⨁◯ | Low ⨁⨁◯◯ | Downgrade | Very low ⨁◯◯◯ |
| Deucravacitinib vs. Upadacitinib | Deucravacitinib vs. Placebo vs. Upadacitinib | Low ⨁⨁◯◯ | Moderate ⨁⨁⨁◯ | Low ⨁⨁◯◯ | Downgrade | Very low ⨁◯◯◯ |
| Filgotinib vs. Ivarmacitinib | Filgotinib vs. Placebo vs. Ivarmacitinib | Moderate ⨁⨁⨁◯ | Moderate ⨁⨁⨁◯ | Moderate ⨁⨁⨁◯ | Downgrade | Low ⨁⨁◯◯ |
| Filgotinib vs. Izencitinib | Filgotinib vs. Placebo vs. Izencitinib | Moderate ⨁⨁⨁◯ | Moderate ⨁⨁⨁◯ | Moderate ⨁⨁⨁◯ | Downgrade | Low ⨁⨁◯◯ |
| Filgotinib vs. Peficitinib | Filgotinib vs. Placebo vs. Peficitinib | Moderate ⨁⨁⨁◯ | Moderate ⨁⨁⨁◯ | Moderate ⨁⨁⨁◯ | Downgrade | Low ⨁⨁◯◯ |
| Filgotinib vs. Ritlecitinib | Filgotinib vs. Placebo vs. Ritlecitinib | Moderate ⨁⨁⨁◯ | Moderate ⨁⨁⨁◯ | Moderate ⨁⨁⨁◯ | Downgrade | Low ⨁⨁◯◯ |
| Filgotinib vs. Tofacitinib | Filgotinib vs. Placebo vs. Tofacitinib | Moderate ⨁⨁⨁◯ | Moderate ⨁⨁⨁◯ | Moderate ⨁⨁⨁◯ | Downgrade | Low ⨁⨁◯◯ |
| Filgotinib vs. Upadacitinib | Filgotinib vs. Placebo vs. Upadacitinib | Moderate ⨁⨁⨁◯ | Moderate ⨁⨁⨁◯ | Moderate ⨁⨁⨁◯ | Downgrade | Low ⨁⨁◯◯ |
| Ivarmacitinib vs. Izencitinib | Ivarmacitinib vs. Placebo vs. Izencitinib | Moderate ⨁⨁⨁◯ | Moderate ⨁⨁⨁◯ | Moderate ⨁⨁⨁◯ | Downgrade | Low ⨁⨁◯◯ |
| Ivarmacitinib vs. Peficitinib | Ivarmacitinib vs. Placebo vs. Peficitinib | Moderate ⨁⨁⨁◯ | Moderate ⨁⨁⨁◯ | Moderate ⨁⨁⨁◯ | Downgrade | Low ⨁⨁◯◯ |
| Ivarmacitinib vs. Ritlecitinib | Ivarmacitinib vs. Placebo vs. Ritlecitinib | Moderate ⨁⨁⨁◯ | Moderate ⨁⨁⨁◯ | Moderate ⨁⨁⨁◯ | No downgrade | Moderate ⨁⨁⨁◯ |
| Ivarmacitinib vs. Tofacitinib | Ivarmacitinib vs. Placebo vs. Tofacitinib | Moderate ⨁⨁⨁◯ | Moderate ⨁⨁⨁◯ | Moderate ⨁⨁⨁◯ | Downgrade | Low ⨁⨁◯◯ |
| Ivarmacitinib vs. Upadacitinib | Ivarmacitinib vs. Placebo vs. Upadacitinib | Moderate ⨁⨁⨁◯ | Moderate ⨁⨁⨁◯ | Moderate ⨁⨁⨁◯ | Downgrade | Low ⨁⨁◯◯ |
| Izencitinib vs. Peficitinib | Izencitinib vs. Placebo vs. Peficitinib | Moderate ⨁⨁⨁◯ | Moderate ⨁⨁⨁◯ | Moderate ⨁⨁⨁◯ | Downgrade | Low ⨁⨁◯◯ |
| Izencitinib vs. Ritlecitinib | Izencitinib vs. Placebo vs. Ritlecitinib | Moderate ⨁⨁⨁◯ | Moderate ⨁⨁⨁◯ | Moderate ⨁⨁⨁◯ | Downgrade | Low ⨁⨁◯◯ |
| Izencitinib vs. Tofacitinib | Izencitinib vs. Placebo vs. Tofacitinib | Moderate ⨁⨁⨁◯ | Moderate ⨁⨁⨁◯ | Moderate ⨁⨁⨁◯ | Downgrade | Low ⨁⨁◯◯ |
| Izencitinib vs. Upadacitinib | Izencitinib vs. Placebo vs. Upadacitinib | Moderate ⨁⨁⨁◯ | Moderate ⨁⨁⨁◯ | Moderate ⨁⨁⨁◯ | Downgrade | Low ⨁⨁◯◯ |
| Peficitinib vs. Ritlecitinib | Peficitinib vs. Placebo vs. Ritlecitinib | Moderate ⨁⨁⨁◯ | Moderate ⨁⨁⨁◯ | Moderate ⨁⨁⨁◯ | No downgrade | Moderate ⨁⨁⨁◯ |
| Peficitinib vs. Tofacitinib | Peficitinib vs. Placebo vs. Tofacitinib | Moderate ⨁⨁⨁◯ | Moderate ⨁⨁⨁◯ | Moderate ⨁⨁⨁◯ | Downgrade | Low ⨁⨁◯◯ |
| Peficitinib vs. Upadacitinib | Peficitinib vs. Placebo vs. Upadacitinib | Moderate ⨁⨁⨁◯ | Moderate ⨁⨁⨁◯ | Moderate ⨁⨁⨁◯ | Downgrade | Low ⨁⨁◯◯ |
| Ritlecitinib vs. Tofacitinib | Ritlecitinib vs. Placebo vs. Tofacitinib | Moderate ⨁⨁⨁◯ | Moderate ⨁⨁⨁◯ | Moderate ⨁⨁⨁◯ | Downgrade | Low ⨁⨁◯◯ |
| Ritlecitinib vs. Upadacitinib | Ritlecitinib vs. Placebo vs. Upadacitinib | Moderate ⨁⨁⨁◯ | Moderate ⨁⨁⨁◯ | Moderate ⨁⨁⨁◯ | Downgrade | Low ⨁⨁◯◯ |
| Tofacitinib vs. Upadacitinib | Tofacitinib vs. Placebo vs. Upadacitinib | Moderate ⨁⨁⨁◯ | Moderate ⨁⨁⨁◯ | Moderate ⨁⨁⨁◯ | No downgrade | Moderate ⨁⨁⨁◯ |

VTE: venous thromboembolism events.

**Supplementary table 8-2-3 Certainty of indirect evidence assessment for CVE (without dose consideration).**

| Comparison | First order loop | Start rating | | | Intransitivity | Preliminary certainty rating |
| --- | --- | --- | --- | --- | --- | --- |
|  |  | A→B Direct preliminary certainty rating | B→C Direct Preliminary certainty rating | Lowest certainty of the first loop components |  |  |
| Deucravacitinib vs. Brepocitinib | Deucravacitinib vs. Placebo vs. Brepocitinib | Low ⨁⨁◯◯ | Moderate ⨁⨁⨁◯ | Low ⨁⨁◯◯ | No downgrade | Low ⨁⨁◯◯ |
| Filgotinib vs. Brepocitinib | Filgotinib vs. Placebo vs. Brepocitinib | Moderate ⨁⨁⨁◯ | Moderate ⨁⨁⨁◯ | Moderate ⨁⨁⨁◯ | Downgrade | Low ⨁⨁◯◯ |
| Ivarmacitinib vs. Brepocitinib | Ivarmacitinib vs. Placebo vs. Brepocitinib | Moderate ⨁⨁⨁◯ | Moderate ⨁⨁⨁◯ | Moderate ⨁⨁⨁◯ | No downgrade | Moderate ⨁⨁⨁◯ |
| Izencitinib vs. Brepocitinib | Izencitinib vs. Placebo vs. Brepocitinib | Moderate ⨁⨁⨁◯ | Moderate ⨁⨁⨁◯ | Moderate ⨁⨁⨁◯ | Downgrade | Low ⨁⨁◯◯ |
| Peficitinib vs. Brepocitinib | Peficitinib vs. Placebo vs. Brepocitinib | Moderate ⨁⨁⨁◯ | Moderate ⨁⨁⨁◯ | Moderate ⨁⨁⨁◯ | No downgrade | Moderate ⨁⨁⨁◯ |
| Tofacitinib vs. Brepocitinib | Tofacitinib vs. Placebo vs. Brepocitinib | Moderate ⨁⨁⨁◯ | Moderate ⨁⨁⨁◯ | Moderate ⨁⨁⨁◯ | Downgrade | Low ⨁⨁◯◯ |
| Upadacitinib vs. Brepocitinib | Upadacitinib vs. Placebo vs. Brepocitinib | Moderate ⨁⨁⨁◯ | Moderate ⨁⨁⨁◯ | Moderate ⨁⨁⨁◯ | Downgrade | Low ⨁⨁◯◯ |
| Deucravacitinib vs. Filgotinib | Deucravacitinib vs. Placebo vs. Filgotinib | Low ⨁⨁◯◯ | Moderate ⨁⨁⨁◯ | Low ⨁⨁◯◯ | Downgrade | Very low ⨁◯◯◯ |
| Deucravacitinib vs. Ivarmacitinib | Deucravacitinib vs. Placebo vs. Ivarmacitinib | Low ⨁⨁◯◯ | Moderate ⨁⨁⨁◯ | Low ⨁⨁◯◯ | No downgrade | Low ⨁⨁◯◯ |
| Deucravacitinib vs. Izencitinib | Deucravacitinib vs. Placebo vs. Izencitinib | Low ⨁⨁◯◯ | Moderate ⨁⨁⨁◯ | Low ⨁⨁◯◯ | Downgrade | Very low ⨁◯◯◯ |
| Deucravacitinib vs. Peficitinib | Deucravacitinib vs. Placebo vs. Peficitinib | Low ⨁⨁◯◯ | Moderate ⨁⨁⨁◯ | Low ⨁⨁◯◯ | No downgrade | Low ⨁⨁◯◯ |
| Deucravacitinib vs. Ritlecitinib | Deucravacitinib vs. Placebo vs. Ritlecitinib | Low ⨁⨁◯◯ | Moderate ⨁⨁⨁◯ | Low ⨁⨁◯◯ | No downgrade | Low ⨁⨁◯◯ |
| Deucravacitinib vs. Tofacitinib | Deucravacitinib vs. Placebo vs. Tofacitinib | Low ⨁⨁◯◯ | Moderate ⨁⨁⨁◯ | Low ⨁⨁◯◯ | Downgrade | Very low ⨁◯◯◯ |
| Deucravacitinib vs. Upadacitinib | Deucravacitinib vs. Placebo vs. Upadacitinib | Low ⨁⨁◯◯ | Moderate ⨁⨁⨁◯ | Low ⨁⨁◯◯ | Downgrade | Very low ⨁◯◯◯ |
| Filgotinib vs. Ivarmacitinib | Filgotinib vs. Placebo vs. Ivarmacitinib | Moderate ⨁⨁⨁◯ | Moderate ⨁⨁⨁◯ | Moderate ⨁⨁⨁◯ | Downgrade | Low ⨁⨁◯◯ |
| Filgotinib vs. Izencitinib | Filgotinib vs. Placebo vs. Izencitinib | Moderate ⨁⨁⨁◯ | Moderate ⨁⨁⨁◯ | Moderate ⨁⨁⨁◯ | Downgrade | Low ⨁⨁◯◯ |
| Filgotinib vs. Peficitinib | Filgotinib vs. Placebo vs. Peficitinib | Moderate ⨁⨁⨁◯ | Moderate ⨁⨁⨁◯ | Moderate ⨁⨁⨁◯ | Downgrade | Low ⨁⨁◯◯ |
| Filgotinib vs. Ritlecitinib | Filgotinib vs. Placebo vs. Ritlecitinib | Moderate ⨁⨁⨁◯ | Moderate ⨁⨁⨁◯ | Moderate ⨁⨁⨁◯ | Downgrade | Low ⨁⨁◯◯ |
| Filgotinib vs. Tofacitinib | Filgotinib vs. Placebo vs. Tofacitinib | Moderate ⨁⨁⨁◯ | Moderate ⨁⨁⨁◯ | Moderate ⨁⨁⨁◯ | Downgrade | Low ⨁⨁◯◯ |
| Filgotinib vs. Upadacitinib | Filgotinib vs. Placebo vs. Upadacitinib | Moderate ⨁⨁⨁◯ | Moderate ⨁⨁⨁◯ | Moderate ⨁⨁⨁◯ | Downgrade | Low ⨁⨁◯◯ |
| Ivarmacitinib vs. Izencitinib | Ivarmacitinib vs. Placebo vs. Izencitinib | Moderate ⨁⨁⨁◯ | Moderate ⨁⨁⨁◯ | Moderate ⨁⨁⨁◯ | Downgrade | Low ⨁⨁◯◯ |
| Ivarmacitinib vs. Peficitinib | Ivarmacitinib vs. Placebo vs. Peficitinib | Moderate ⨁⨁⨁◯ | Moderate ⨁⨁⨁◯ | Moderate ⨁⨁⨁◯ | Downgrade | Low ⨁⨁◯◯ |
| Ivarmacitinib vs. Ritlecitinib | Ivarmacitinib vs. Placebo vs. Ritlecitinib | Moderate ⨁⨁⨁◯ | Moderate ⨁⨁⨁◯ | Moderate ⨁⨁⨁◯ | No downgrade | Moderate ⨁⨁⨁◯ |
| Ivarmacitinib vs. Tofacitinib | Ivarmacitinib vs. Placebo vs. Tofacitinib | Moderate ⨁⨁⨁◯ | Moderate ⨁⨁⨁◯ | Moderate ⨁⨁⨁◯ | Downgrade | Low ⨁⨁◯◯ |
| Ivarmacitinib vs. Upadacitinib | Ivarmacitinib vs. Placebo vs. Upadacitinib | Moderate ⨁⨁⨁◯ | Moderate ⨁⨁⨁◯ | Moderate ⨁⨁⨁◯ | Downgrade | Low ⨁⨁◯◯ |
| Izencitinib vs. Peficitinib | Izencitinib vs. Placebo vs. Peficitinib | Moderate ⨁⨁⨁◯ | Moderate ⨁⨁⨁◯ | Moderate ⨁⨁⨁◯ | Downgrade | Low ⨁⨁◯◯ |
| Izencitinib vs. Ritlecitinib | Izencitinib vs. Placebo vs. Ritlecitinib | Moderate ⨁⨁⨁◯ | Moderate ⨁⨁⨁◯ | Moderate ⨁⨁⨁◯ | Downgrade | Low ⨁⨁◯◯ |
| Izencitinib vs. Tofacitinib | Izencitinib vs. Placebo vs. Tofacitinib | Moderate ⨁⨁⨁◯ | Moderate ⨁⨁⨁◯ | Moderate ⨁⨁⨁◯ | Downgrade | Low ⨁⨁◯◯ |
| Izencitinib vs. Upadacitinib | Izencitinib vs. Placebo vs. Upadacitinib | Moderate ⨁⨁⨁◯ | Moderate ⨁⨁⨁◯ | Moderate ⨁⨁⨁◯ | Downgrade | Low ⨁⨁◯◯ |
| Peficitinib vs. Ritlecitinib | Peficitinib vs. Placebo vs. Ritlecitinib | Moderate ⨁⨁⨁◯ | Moderate ⨁⨁⨁◯ | Moderate ⨁⨁⨁◯ | No downgrade | Moderate ⨁⨁⨁◯ |
| Peficitinib vs. Tofacitinib | Peficitinib vs. Placebo vs. Tofacitinib | Moderate ⨁⨁⨁◯ | Moderate ⨁⨁⨁◯ | Moderate ⨁⨁⨁◯ | Downgrade | Low ⨁⨁◯◯ |
| Peficitinib vs. Upadacitinib | Peficitinib vs. Placebo vs. Upadacitinib | Moderate ⨁⨁⨁◯ | Moderate ⨁⨁⨁◯ | Moderate ⨁⨁⨁◯ | Downgrade | Low ⨁⨁◯◯ |
| Ritlecitinib vs. Tofacitinib | Ritlecitinib vs. Placebo vs. Tofacitinib | Moderate ⨁⨁⨁◯ | Moderate ⨁⨁⨁◯ | Moderate ⨁⨁⨁◯ | Downgrade | Low ⨁⨁◯◯ |
| Ritlecitinib vs. Upadacitinib | Ritlecitinib vs. Placebo vs. Upadacitinib | Moderate ⨁⨁⨁◯ | Moderate ⨁⨁⨁◯ | Moderate ⨁⨁⨁◯ | Downgrade | Low ⨁⨁◯◯ |
| Tofacitinib vs. Upadacitinib | Tofacitinib vs. Placebo vs. Upadacitinib | Moderate ⨁⨁⨁◯ | Moderate ⨁⨁⨁◯ | Moderate ⨁⨁⨁◯ | No downgrade | Moderate ⨁⨁⨁◯ |

CVE: cardiovascular events.

**Supplementary table 9 The Risk-of-bias assessment for the direct evidence (with dose consideration).**

| Comparison | Number of  total RCTs (%) | Number of  low RoB RCTs (%) | Number of  moderate RoB RCTs (%) | Number of  high RoB RCTs (%) | Risk-of-bias assessment |
| --- | --- | --- | --- | --- | --- |
| Placebo vs. Brepocitinib | 1 | 1 (100%) | 0 | 0 | No downgrade |
| Ritlecitinib vs. Brepocitinib | 1 | 1 (100%) | 0 | 0 | No downgrade |
| Deucravacitinib vs. Placebo | 1 | 0 | 1 (100%) | 0 | Downgrade |
| Filgotinib 100 mg QD vs. Filgotinib 200 mg QD | 4 | 2 (50%) | 1 (25%) | 1 (25%) | No downgrade |
| Filgotinib 100 mg QD vs. Placebo | 4 | 2 (50%) | 1 (25%) | 1 (25%) | No downgrade |
| Filgotinib 200 mg QD vs. Placebo | 5 | 3 (60%) | 1 (20%) | 1 (20%) | No downgrade |
| Ivarmacitinib vs. Placebo | 1 | 1 (100%) | 0 | 0 | No downgrade |
| Izencitinib vs. Placebo | 2 | 0 | 2 (100%) | 0 | Downgrade |
| Peficitinib vs. Placebo | 1 | 1 (100%) | 0 | 0 | No downgrade |
| Placebo vs. Ritlecitinib | 1 | 1 (100%) | 0 | 0 | No downgrade |
| Placebo vs. Tofacitinib 0.5 mg BID | 2 | 2 (100%) | 0 | 0 | No downgrade |
| Placebo vs. Tofacitinib 1 mg BID | 2 | 0 | 2 (100%) | 0 | Downgrade |
| Placebo vs. Tofacitinib 10 mg BID | 7 | 7 (100%) | 0 | 0 | No downgrade |
| Placebo vs. Tofacitinib 15 mg BID | 4 | 2 (50%) | 2 (50%) | 0 | No downgrade |
| Placebo vs. Tofacitinib 3 mg BID | 2 | 2 (100%) | 0 | 0 | No downgrade |
| Placebo vs. Tofacitinib 5 mg BID | 5 | 3 (60%) | 2 (40%) | 0 | No downgrade |
| Placebo vs. Upadacitinib 15 mg QD | 2 | 2 (100%) | 0 | 0 | No downgrade |
| Placebo vs. Upadacitinib 30 mg QD | 2 | 2 (100%) | 0 | 0 | No downgrade |
| Placebo vs. Upadacitinib 45 mg QD | 4 | 4 (100%) | 0 | 0 | No downgrade |
| Tofacitinib 0.5 mg BID vs. Tofacitinib 10 mg BID | 2 | 2 (100%) | 0 | 0 | No downgrade |
| Tofacitinib 0.5 mg BID vs. Tofacitinib 15 mg BID | 2 | 2 (100%) | 0 | 0 | No downgrade |
| Tofacitinib 0.5 mg BID vs. Tofacitinib 3 mg BID | 2 | 2 (100%) | 0 | 0 | No downgrade |
| Tofacitinib 1 mg BID vs. Tofacitinib 15 mg BID | 2 | 0 | 2 (100%) | 0 | Downgrade |
| Tofacitinib 1 mg BID vs. Tofacitinib 5 mg BID | 2 | 0 | 2 (100%) | 0 | Downgrade |
| Tofacitinib 10 mg BID vs. Tofacitinib 15 mg BID | 2 | 2 (100%) | 0 | 0 | No downgrade |
| Tofacitinib 10 mg BID vs. Tofacitinib 3 mg BID | 1 | 1 (100%) | 0 | 0 | No downgrade |
| Tofacitinib 10 mg BID vs. Tofacitinib 5 mg BID | 3 | 3 (100%) | 0 | 0 | No downgrade |
| Tofacitinib 15 mg BID vs. Tofacitinib 3 mg BID | 2 | 2 (100%) | 0 | 0 | No downgrade |
| Tofacitinib 15 mg BID vs. Tofacitinib 5 mg BID | 2 | 0 | 2 (100%) | 0 | Downgrade |
| Upadacitinib 15 mg QD vs. Upadacitinib 30 mg QD | 2 | 2 (100%) | 0 | 0 | No downgrade |
| Upadacitinib 15 mg QD vs. Upadacitinib 45 mg QD | 1 | 1 (100%) | 0 | 0 | No downgrade |
| Upadacitinib 30 mg QD vs. Upadacitinib 45 mg QD | 1 | 1 (100%) | 0 | 0 | No downgrade |

No.: numero = number; RoB: Risk of bias; RCT: randomized controlled trials; QD: once daily; BID: twice daily. The Risk-of-bias will be downgraded by one level when the contributions from low RoB comparisons were less than 30% and contributions from moderate RoB comparisons were 70% or greater.

**Supplementary table 10-1-1 Certainty of direct evidence assessment for MACE (with dose consideration).**

| Comparison | Risk of bias | Inconsistency | Indirectness | Publication bias | Preliminary certainty rating |
| --- | --- | --- | --- | --- | --- |
| Placebo vs. Brepocitinib | No downgrade | No downgrade | Downgrade | Downgrade | Low ⨁⨁◯◯ |
| Ritlecitinib vs. Brepocitinib | No downgrade | No downgrade | Downgrade | Downgrade | Low ⨁⨁◯◯ |
| Deucravacitinib vs. Placebo | Downgrade | No downgrade | Downgrade | Downgrade | Very low ⨁◯◯◯ |
| Filgotinib 100 mg QD vs. Filgotinib 200 mg QD | No downgrade | No downgrade | Downgrade | Downgrade | Low ⨁⨁◯◯ |
| Filgotinib 100 mg QD vs. Placebo | No downgrade | No downgrade | Downgrade | Downgrade | Low ⨁⨁◯◯ |
| Filgotinib 200 mg QD vs. Placebo | No downgrade | No downgrade | Downgrade | Downgrade | Low ⨁⨁◯◯ |
| Ivarmacitinib vs. Placebo | No downgrade | No downgrade | Downgrade | Downgrade | Low ⨁⨁◯◯ |
| Izencitinib vs. Placebo | Downgrade | No downgrade | No downgrade | Downgrade | Low ⨁⨁◯◯ |
| Peficitinib vs. Placebo | No downgrade | No downgrade | Downgrade | Downgrade | Low ⨁⨁◯◯ |
| Placebo vs. Ritlecitinib | No downgrade | No downgrade | Downgrade | Downgrade | Low ⨁⨁◯◯ |
| Placebo vs. Tofacitinib 0.5 mg BID | No downgrade | No downgrade | Downgrade | Downgrade | Low ⨁⨁◯◯ |
| Placebo vs. Tofacitinib 1 mg BID | Downgrade | No downgrade | Downgrade | Downgrade | Very low ⨁◯◯◯ |
| Placebo vs. Tofacitinib 10 mg BID | No downgrade | No downgrade | Downgrade | Downgrade | Low ⨁⨁◯◯ |
| Placebo vs. Tofacitinib 15 mg BID | No downgrade | No downgrade | Downgrade | Downgrade | Low ⨁⨁◯◯ |
| Placebo vs. Tofacitinib 3 mg BID | No downgrade | No downgrade | Downgrade | Downgrade | Low ⨁⨁◯◯ |
| Placebo vs. Tofacitinib 5 mg BID | No downgrade | No downgrade | Downgrade | Downgrade | Low ⨁⨁◯◯ |
| Placebo vs. Upadacitinib 15 mg QD | No downgrade | No downgrade | Downgrade | Downgrade | Low ⨁⨁◯◯ |
| Placebo vs. Upadacitinib 30 mg QD | No downgrade | No downgrade | Downgrade | Downgrade | Low ⨁⨁◯◯ |
| Placebo vs. Upadacitinib 45 mg QD | No downgrade | No downgrade | Downgrade | Downgrade | Low ⨁⨁◯◯ |
| Tofacitinib 0.5 mg BID vs. Tofacitinib 10 mg BID | No downgrade | No downgrade | Downgrade | Downgrade | Low ⨁⨁◯◯ |
| Tofacitinib 0.5 mg BID vs. Tofacitinib 15 mg BID | No downgrade | No downgrade | Downgrade | Downgrade | Low ⨁⨁◯◯ |
| Tofacitinib 0.5 mg BID vs. Tofacitinib 3 mg BID | No downgrade | No downgrade | Downgrade | Downgrade | Low ⨁⨁◯◯ |
| Tofacitinib 1 mg BID vs. Tofacitinib 15 mg BID | Downgrade | No downgrade | Downgrade | Downgrade | Very low ⨁◯◯◯ |
| Tofacitinib 1 mg BID vs. Tofacitinib 5 mg BID | Downgrade | No downgrade | Downgrade | Downgrade | Very low ⨁◯◯◯ |
| Tofacitinib 10 mg BID vs. Tofacitinib 15 mg BID | No downgrade | No downgrade | Downgrade | Downgrade | Low ⨁⨁◯◯ |
| Tofacitinib 10 mg BID vs. Tofacitinib 3 mg BID | No downgrade | No downgrade | Downgrade | Downgrade | Low ⨁⨁◯◯ |
| Tofacitinib 10 mg BID vs. Tofacitinib 5 mg BID | No downgrade | No downgrade | Downgrade | Downgrade | Low ⨁⨁◯◯ |
| Tofacitinib 15 mg BID vs. Tofacitinib 3 mg BID | No downgrade | No downgrade | Downgrade | Downgrade | Low ⨁⨁◯◯ |
| Tofacitinib 15 mg BID vs. Tofacitinib 5 mg BID | Downgrade | No downgrade | Downgrade | Downgrade | Very low ⨁◯◯◯ |
| Upadacitinib 15 mg QD vs. Upadacitinib 30 mg QD | No downgrade | No downgrade | Downgrade | Downgrade | Low ⨁⨁◯◯ |
| Upadacitinib 15 mg QD vs. Upadacitinib 45 mg QD | No downgrade | No downgrade | Downgrade | Downgrade | Low ⨁⨁◯◯ |
| Upadacitinib 30 mg QD vs. Upadacitinib 45 mg QD | No downgrade | No downgrade | Downgrade | Downgrade | Low ⨁⨁◯◯ |

MACE: major adverse cardiovascular events; QD: once daily; BID: twice daily.

**Supplementary table 10-1-2 Certainty of direct evidence assessment for VTE (with dose consideration).**

| Comparison | Risk of bias | Inconsistency | Indirectness | Publication bias | Preliminary certainty rating |
| --- | --- | --- | --- | --- | --- |
| Placebo vs. Brepocitinib | No downgrade | No downgrade | Downgrade | Downgrade | Low ⨁⨁◯◯ |
| Ritlecitinib vs. Brepocitinib | No downgrade | No downgrade | Downgrade | Downgrade | Low ⨁⨁◯◯ |
| Deucravacitinib vs. Placebo | Downgrade | No downgrade | Downgrade | Downgrade | Very low ⨁◯◯◯ |
| Filgotinib 100 mg QD vs. Filgotinib 200 mg QD | No downgrade | No downgrade | Downgrade | Downgrade | Low ⨁⨁◯◯ |
| Filgotinib 100 mg QD vs. Placebo | No downgrade | No downgrade | Downgrade | Downgrade | Low ⨁⨁◯◯ |
| Filgotinib 200 mg QD vs. Placebo | No downgrade | No downgrade | Downgrade | Downgrade | Low ⨁⨁◯◯ |
| Ivarmacitinib vs. Placebo | No downgrade | No downgrade | Downgrade | Downgrade | Low ⨁⨁◯◯ |
| Izencitinib vs. Placebo | Downgrade | No downgrade | No downgrade | Downgrade | Low ⨁⨁◯◯ |
| Peficitinib vs. Placebo | No downgrade | No downgrade | Downgrade | Downgrade | Low ⨁⨁◯◯ |
| Placebo vs. Ritlecitinib | No downgrade | No downgrade | Downgrade | Downgrade | Low ⨁⨁◯◯ |
| Placebo vs. Tofacitinib 0.5 mg BID | No downgrade | No downgrade | Downgrade | Downgrade | Low ⨁⨁◯◯ |
| Placebo vs. Tofacitinib 1 mg BID | Downgrade | No downgrade | Downgrade | Downgrade | Very low ⨁◯◯◯ |
| Placebo vs. Tofacitinib 10 mg BID | No downgrade | No downgrade | Downgrade | Downgrade | Low ⨁⨁◯◯ |
| Placebo vs. Tofacitinib 15 mg BID | No downgrade | No downgrade | Downgrade | Downgrade | Low ⨁⨁◯◯ |
| Placebo vs. Tofacitinib 3 mg BID | No downgrade | No downgrade | Downgrade | Downgrade | Low ⨁⨁◯◯ |
| Placebo vs. Tofacitinib 5 mg BID | No downgrade | No downgrade | Downgrade | Downgrade | Low ⨁⨁◯◯ |
| Placebo vs. Upadacitinib 15 mg QD | No downgrade | No downgrade | Downgrade | Downgrade | Low ⨁⨁◯◯ |
| Placebo vs. Upadacitinib 30 mg QD | No downgrade | No downgrade | Downgrade | Downgrade | Low ⨁⨁◯◯ |
| Placebo vs. Upadacitinib 45 mg QD | No downgrade | No downgrade | Downgrade | Downgrade | Low ⨁⨁◯◯ |
| Tofacitinib 0.5 mg BID vs. Tofacitinib 10 mg BID | No downgrade | No downgrade | Downgrade | Downgrade | Low ⨁⨁◯◯ |
| Tofacitinib 0.5 mg BID vs. Tofacitinib 15 mg BID | No downgrade | No downgrade | Downgrade | Downgrade | Low ⨁⨁◯◯ |
| Tofacitinib 0.5 mg BID vs. Tofacitinib 3 mg BID | No downgrade | No downgrade | Downgrade | Downgrade | Low ⨁⨁◯◯ |
| Tofacitinib 1 mg BID vs. Tofacitinib 15 mg BID | Downgrade | No downgrade | Downgrade | Downgrade | Very low ⨁◯◯◯ |
| Tofacitinib 1 mg BID vs. Tofacitinib 5 mg BID | Downgrade | No downgrade | Downgrade | Downgrade | Very low ⨁◯◯◯ |
| Tofacitinib 10 mg BID vs. Tofacitinib 15 mg BID | No downgrade | No downgrade | Downgrade | Downgrade | Low ⨁⨁◯◯ |
| Tofacitinib 10 mg BID vs. Tofacitinib 3 mg BID | No downgrade | No downgrade | Downgrade | Downgrade | Low ⨁⨁◯◯ |
| Tofacitinib 10 mg BID vs. Tofacitinib 5 mg BID | No downgrade | No downgrade | Downgrade | Downgrade | Low ⨁⨁◯◯ |
| Tofacitinib 15 mg BID vs. Tofacitinib 3 mg BID | No downgrade | No downgrade | Downgrade | Downgrade | Low ⨁⨁◯◯ |
| Tofacitinib 15 mg BID vs. Tofacitinib 5 mg BID | Downgrade | No downgrade | Downgrade | Downgrade | Very low ⨁◯◯◯ |
| Upadacitinib 15 mg QD vs. Upadacitinib 30 mg QD | No downgrade | No downgrade | Downgrade | Downgrade | Low ⨁⨁◯◯ |
| Upadacitinib 15 mg QD vs. Upadacitinib 45 mg QD | No downgrade | No downgrade | Downgrade | Downgrade | Low ⨁⨁◯◯ |
| Upadacitinib 30 mg QD vs. Upadacitinib 45 mg QD | No downgrade | No downgrade | Downgrade | Downgrade | Low ⨁⨁◯◯ |

VTE: venous thromboembolism events; QD: once daily; BID: twice daily.

**Supplementary table 10-1-3 Certainty of direct evidence assessment for CVE (with dose consideration).**

| Comparison | Risk of bias | Inconsistency | Indirectness | Publication bias | Preliminary certainty rating |
| --- | --- | --- | --- | --- | --- |
| Placebo vs. Brepocitinib | No downgrade | No downgrade | Downgrade | No downgrade | Moderate ⨁⨁⨁◯ |
| Ritlecitinib vs. Brepocitinib | No downgrade | No downgrade | Downgrade | No downgrade | Moderate ⨁⨁⨁◯ |
| Deucravacitinib vs. Placebo | Downgrade | No downgrade | Downgrade | No downgrade | Low ⨁⨁◯◯ |
| Filgotinib 100 mg QD vs. Filgotinib 200 mg QD | No downgrade | No downgrade | Downgrade | No downgrade | Moderate ⨁⨁⨁◯ |
| Filgotinib 100 mg QD vs. Placebo | No downgrade | No downgrade | Downgrade | No downgrade | Moderate ⨁⨁⨁◯ |
| Filgotinib 200 mg QD vs. Placebo | No downgrade | No downgrade | Downgrade | No downgrade | Moderate ⨁⨁⨁◯ |
| Ivarmacitinib vs. Placebo | No downgrade | No downgrade | Downgrade | No downgrade | Moderate ⨁⨁⨁◯ |
| Izencitinib vs. Placebo | Downgrade | No downgrade | No downgrade | No downgrade | Moderate ⨁⨁⨁◯ |
| Peficitinib vs. Placebo | No downgrade | No downgrade | Downgrade | No downgrade | Moderate ⨁⨁⨁◯ |
| Placebo vs. Ritlecitinib | No downgrade | No downgrade | Downgrade | No downgrade | Moderate ⨁⨁⨁◯ |
| Placebo vs. Tofacitinib 0.5 mg BID | No downgrade | No downgrade | Downgrade | No downgrade | Moderate ⨁⨁⨁◯ |
| Placebo vs. Tofacitinib 1 mg BID | Downgrade | No downgrade | Downgrade | No downgrade | Low ⨁⨁◯◯ |
| Placebo vs. Tofacitinib 10 mg BID | No downgrade | No downgrade | Downgrade | No downgrade | Moderate ⨁⨁⨁◯ |
| Placebo vs. Tofacitinib 15 mg BID | No downgrade | No downgrade | Downgrade | No downgrade | Moderate ⨁⨁⨁◯ |
| Placebo vs. Tofacitinib 3 mg BID | No downgrade | No downgrade | Downgrade | No downgrade | Moderate ⨁⨁⨁◯ |
| Placebo vs. Tofacitinib 5 mg BID | No downgrade | No downgrade | Downgrade | No downgrade | Moderate ⨁⨁⨁◯ |
| Placebo vs. Upadacitinib 15 mg QD | No downgrade | No downgrade | Downgrade | No downgrade | Moderate ⨁⨁⨁◯ |
| Placebo vs. Upadacitinib 30 mg QD | No downgrade | No downgrade | Downgrade | No downgrade | Moderate ⨁⨁⨁◯ |
| Placebo vs. Upadacitinib 45 mg QD | No downgrade | No downgrade | Downgrade | No downgrade | Moderate ⨁⨁⨁◯ |
| Tofacitinib 0.5 mg BID vs. Tofacitinib 10 mg BID | No downgrade | No downgrade | Downgrade | No downgrade | Moderate ⨁⨁⨁◯ |
| Tofacitinib 0.5 mg BID vs. Tofacitinib 15 mg BID | No downgrade | No downgrade | Downgrade | No downgrade | Moderate ⨁⨁⨁◯ |
| Tofacitinib 0.5 mg BID vs. Tofacitinib 3 mg BID | No downgrade | No downgrade | Downgrade | No downgrade | Moderate ⨁⨁⨁◯ |
| Tofacitinib 1 mg BID vs. Tofacitinib 15 mg BID | Downgrade | No downgrade | Downgrade | No downgrade | Low ⨁⨁◯◯ |
| Tofacitinib 1 mg BID vs. Tofacitinib 5 mg BID | Downgrade | No downgrade | Downgrade | No downgrade | Low ⨁⨁◯◯ |
| Tofacitinib 10 mg BID vs. Tofacitinib 15 mg BID | No downgrade | No downgrade | Downgrade | No downgrade | Moderate ⨁⨁⨁◯ |
| Tofacitinib 10 mg BID vs. Tofacitinib 3 mg BID | No downgrade | No downgrade | Downgrade | No downgrade | Moderate ⨁⨁⨁◯ |
| Tofacitinib 10 mg BID vs. Tofacitinib 5 mg BID | No downgrade | No downgrade | Downgrade | No downgrade | Moderate ⨁⨁⨁◯ |
| Tofacitinib 15 mg BID vs. Tofacitinib 3 mg BID | No downgrade | No downgrade | Downgrade | No downgrade | Moderate ⨁⨁⨁◯ |
| Tofacitinib 15 mg BID vs. Tofacitinib 5 mg BID | Downgrade | No downgrade | Downgrade | No downgrade | Low ⨁⨁◯◯ |
| Upadacitinib 15 mg QD vs. Upadacitinib 30 mg QD | No downgrade | No downgrade | Downgrade | No downgrade | Moderate ⨁⨁⨁◯ |
| Upadacitinib 15 mg QD vs. Upadacitinib 45 mg QD | No downgrade | No downgrade | Downgrade | No downgrade | Moderate ⨁⨁⨁◯ |
| Upadacitinib 30 mg QD vs. Upadacitinib 45 mg QD | No downgrade | No downgrade | Downgrade | No downgrade | Moderate ⨁⨁⨁◯ |

CVE: cardiovascular events; QD: once daily; BID: twice daily.

**Supplementary table 10-2-1 Certainty of indirect evidence assessment for MACE (with dose consideration).**

| Comparison | First order loop | Start rating | | | Intransitivity | Preliminary certainty rating |
| --- | --- | --- | --- | --- | --- | --- |
|  |  | A→B Direct preliminary certainty rating | B→C Direct Preliminary certainty rating | Lowest certainty of the first loop components |  |  |
| Deucravacitinib vs. Brepocitinib | Deucravacitinib vs. Placebo vs. Brepocitinib | Very low ⨁◯◯◯ | Low ⨁⨁◯◯ | Very low ⨁◯◯◯ | No downgrade | Very low ⨁◯◯◯ |
| Filgotinib 100 mg QD vs. Brepocitinib | Filgotinib 100 mg QD vs. Placebo vs. Brepocitinib | Low ⨁⨁◯◯ | Low ⨁⨁◯◯ | Low ⨁⨁◯◯ | Downgrade | Very low ⨁◯◯◯ |
| Filgotinib 200 mg QD vs. Brepocitinib | Filgotinib 200 mg QD vs. Placebo vs. Brepocitinib | Low ⨁⨁◯◯ | Low ⨁⨁◯◯ | Low ⨁⨁◯◯ | Downgrade | Very low ⨁◯◯◯ |
| Ivarmacitinib vs. Brepocitinib | Ivarmacitinib vs. Placebo vs. Brepocitinib | Low ⨁⨁◯◯ | Low ⨁⨁◯◯ | Low ⨁⨁◯◯ | Downgrade | Very low ⨁◯◯◯ |
| Izencitinib vs. Brepocitinib | Izencitinib vs. Placebo vs. Brepocitinib | Low ⨁⨁◯◯ | Low ⨁⨁◯◯ | Low ⨁⨁◯◯ | Downgrade | Very low ⨁◯◯◯ |
| Peficitinib vs. Brepocitinib | Peficitinib vs. Placebo vs. Brepocitinib | Low ⨁⨁◯◯ | Low ⨁⨁◯◯ | Low ⨁⨁◯◯ | No downgrade | Low ⨁⨁◯◯ |
| Tofacitinib 0.5 mg BID vs. Brepocitinib | Tofacitinib 0.5 mg BID vs. Placebo vs. Brepocitinib | Low ⨁⨁◯◯ | Low ⨁⨁◯◯ | Low ⨁⨁◯◯ | No downgrade | Low ⨁⨁◯◯ |
| Tofacitinib 1 mg BID vs. Brepocitinib | Tofacitinib 1 mg BID vs. Placebo vs. Brepocitinib | Very low ⨁◯◯◯ | Low ⨁⨁◯◯ | Very low ⨁◯◯◯ | Downgrade | Very low ⨁◯◯◯ |
| Tofacitinib 10 mg BID vs. Brepocitinib | Tofacitinib 10 mg BID vs. Placebo vs. Brepocitinib | Low ⨁⨁◯◯ | Low ⨁⨁◯◯ | Low ⨁⨁◯◯ | Downgrade | Very low ⨁◯◯◯ |
| Tofacitinib 15 mg BID vs. Brepocitinib | Tofacitinib 15 mg BID vs. Placebo vs. Brepocitinib | Low ⨁⨁◯◯ | Low ⨁⨁◯◯ | Low ⨁⨁◯◯ | Downgrade | Very low ⨁◯◯◯ |
| Tofacitinib 3 mg BID vs. Brepocitinib | Tofacitinib 3 mg BID vs. Placebo vs. Brepocitinib | Low ⨁⨁◯◯ | Low ⨁⨁◯◯ | Low ⨁⨁◯◯ | No downgrade | Low ⨁⨁◯◯ |
| Tofacitinib 5 mg BID vs. Brepocitinib | Tofacitinib 5 mg BID vs. Placebo vs. Brepocitinib | Low ⨁⨁◯◯ | Low ⨁⨁◯◯ | Low ⨁⨁◯◯ | Downgrade | Very low ⨁◯◯◯ |
| Upadacitinib 15 mg QD vs. Brepocitinib | Upadacitinib 15 mg QD vs. Placebo vs. Brepocitinib | Low ⨁⨁◯◯ | Low ⨁⨁◯◯ | Low ⨁⨁◯◯ | Downgrade | Very low ⨁◯◯◯ |
| Upadacitinib 30 mg QD vs. Brepocitinib | Upadacitinib 30 mg QD vs. Placebo vs. Brepocitinib | Low ⨁⨁◯◯ | Low ⨁⨁◯◯ | Low ⨁⨁◯◯ | Downgrade | Very low ⨁◯◯◯ |
| Upadacitinib 45 mg QD vs. Brepocitinib | Upadacitinib 45 mg QD vs. Placebo vs. Brepocitinib | Low ⨁⨁◯◯ | Low ⨁⨁◯◯ | Low ⨁⨁◯◯ | Downgrade | Very low ⨁◯◯◯ |
| Deucravacitinib vs. Filgotinib 100 mg QD | Deucravacitinib vs. Placebo vs. Filgotinib 100 mg QD | Very low ⨁◯◯◯ | Low ⨁⨁◯◯ | Very low ⨁◯◯◯ | Downgrade | Very low ⨁◯◯◯ |
| Deucravacitinib vs. Filgotinib 200 mg QD | Deucravacitinib vs. Placebo vs. Filgotinib 200 mg QD | Very low ⨁◯◯◯ | Low ⨁⨁◯◯ | Very low ⨁◯◯◯ | Downgrade | Very low ⨁◯◯◯ |
| Deucravacitinib vs. Ivarmacitinib | Deucravacitinib vs. Placebo vs. Ivarmacitinib | Very low ⨁◯◯◯ | Low ⨁⨁◯◯ | Very low ⨁◯◯◯ | No downgrade | Very low ⨁◯◯◯ |
| Deucravacitinib vs. Izencitinib | Deucravacitinib vs. Placebo vs. Izencitinib | Very low ⨁◯◯◯ | Low ⨁⨁◯◯ | Very low ⨁◯◯◯ | Downgrade | Very low ⨁◯◯◯ |
| Deucravacitinib vs. Peficitinib | Deucravacitinib vs. Placebo vs. Peficitinib | Very low ⨁◯◯◯ | Low ⨁⨁◯◯ | Very low ⨁◯◯◯ | No downgrade | Very low ⨁◯◯◯ |
| Deucravacitinib vs. Ritlecitinib | Deucravacitinib vs. Placebo vs. Ritlecitinib | Very low ⨁◯◯◯ | Low ⨁⨁◯◯ | Very low ⨁◯◯◯ | No downgrade | Very low ⨁◯◯◯ |
| Deucravacitinib vs. Tofacitinib 0.5 mg BID | Deucravacitinib vs. Placebo vs. Tofacitinib 0.5 mg BID | Very low ⨁◯◯◯ | Low ⨁⨁◯◯ | Very low ⨁◯◯◯ | No downgrade | Very low ⨁◯◯◯ |
| Deucravacitinib vs. Tofacitinib 1 mg BID | Deucravacitinib vs. Placebo vs. Tofacitinib 1 mg BID | Very low ⨁◯◯◯ | Very low ⨁◯◯◯ | Very low ⨁◯◯◯ | Downgrade | Very low ⨁◯◯◯ |
| Deucravacitinib vs. Tofacitinib 10 mg BID | Deucravacitinib vs. Placebo vs. Tofacitinib 10 mg BID | Very low ⨁◯◯◯ | Low ⨁⨁◯◯ | Very low ⨁◯◯◯ | Downgrade | Very low ⨁◯◯◯ |
| Deucravacitinib vs. Tofacitinib 15 mg BID | Deucravacitinib vs. Placebo vs. Tofacitinib 15 mg BID | Very low ⨁◯◯◯ | Low ⨁⨁◯◯ | Very low ⨁◯◯◯ | Downgrade | Very low ⨁◯◯◯ |
| Deucravacitinib vs. Tofacitinib 3 mg BID | Deucravacitinib vs. Placebo vs. Tofacitinib 3 mg BID | Very low ⨁◯◯◯ | Low ⨁⨁◯◯ | Very low ⨁◯◯◯ | No downgrade | Very low ⨁◯◯◯ |
| Deucravacitinib vs. Tofacitinib 5 mg BID | Deucravacitinib vs. Placebo vs. Tofacitinib 5 mg BID | Very low ⨁◯◯◯ | Low ⨁⨁◯◯ | Very low ⨁◯◯◯ | Downgrade | Very low ⨁◯◯◯ |
| Deucravacitinib vs. Upadacitinib 15 mg QD | Deucravacitinib vs. Placebo vs. Upadacitinib15 mg QD | Very low ⨁◯◯◯ | Low ⨁⨁◯◯ | Very low ⨁◯◯◯ | Downgrade | Very low ⨁◯◯◯ |
| Deucravacitinib vs. Upadacitinib 30 mg QD | Deucravacitinib vs. Placebo vs. Upadacitinib 30 mg QD | Very low ⨁◯◯◯ | Low ⨁⨁◯◯ | Very low ⨁◯◯◯ | Downgrade | Very low ⨁◯◯◯ |
| Deucravacitinib vs. Upadacitinib 45 mg QD | Deucravacitinib vs. Placebo vs. Upadacitinib 45 mg QD | Very low ⨁◯◯◯ | Low ⨁⨁◯◯ | Very low ⨁◯◯◯ | Downgrade | Very low ⨁◯◯◯ |
| Filgotinib 100 mg QD vs. Filgotinib 200 mg QD | Filgotinib 100 mg QD vs. Placebo vs. Filgotinib 200 mg QD | Low ⨁⨁◯◯ | Low ⨁⨁◯◯ | Low ⨁⨁◯◯ | No downgrade | Low ⨁⨁◯◯ |
| Filgotinib 100 mg QD vs. Ivarmacitinib | Filgotinib 100 mg QD vs. Placebo vs. Ivarmacitinib | Low ⨁⨁◯◯ | Low ⨁⨁◯◯ | Low ⨁⨁◯◯ | Downgrade | Very low ⨁◯◯◯ |
| Filgotinib 100 mg QD vs. Izencitinib | Filgotinib 100 mg QD vs. Placebo vs. Izencitinib | Low ⨁⨁◯◯ | Low ⨁⨁◯◯ | Low ⨁⨁◯◯ | Downgrade | Very low ⨁◯◯◯ |
| Filgotinib 100 mg QD vs. Peficitinib | Filgotinib 100 mg QD vs. Placebo vs. Peficitinib | Low ⨁⨁◯◯ | Low ⨁⨁◯◯ | Low ⨁⨁◯◯ | Downgrade | Very low ⨁◯◯◯ |
| Filgotinib 100 mg QD vs. Placebo | Filgotinib 100 mg QD vs. Filgotinib 200 vs. Placebo | Low ⨁⨁◯◯ | Low ⨁⨁◯◯ | Low ⨁⨁◯◯ | Downgrade | Very low ⨁◯◯◯ |
| Filgotinib 100 mg QD vs. Ritlecitinib | Filgotinib 100 mg QD vs. Placebo vs. Ritlecitinib | Low ⨁⨁◯◯ | Low ⨁⨁◯◯ | Low ⨁⨁◯◯ | Downgrade | Very low ⨁◯◯◯ |
| Filgotinib 100 mg QD vs. Tofacitinib 0.5 mg BID | Filgotinib 100 mg QD vs. Placebo vs. Tofacitinib 0.5 mg BID | Low ⨁⨁◯◯ | Low ⨁⨁◯◯ | Low ⨁⨁◯◯ | Downgrade | Very low ⨁◯◯◯ |
| Filgotinib 100 mg QD vs. Tofacitinib 1 mg BID | Filgotinib 100 mg QD vs. Placebo vs. Tofacitinib 1 mg BID | Low ⨁⨁◯◯ | Very low ⨁◯◯◯ | Very low ⨁◯◯◯ | Downgrade | Very low ⨁◯◯◯ |
| Filgotinib 100 mg QD vs. Tofacitinib 10 mg BID | Filgotinib 100 mg QD vs. Placebo vs. Tofacitinib 10 mg BID | Low ⨁⨁◯◯ | Low ⨁⨁◯◯ | Low ⨁⨁◯◯ | Downgrade | Very low ⨁◯◯◯ |
| Filgotinib 100 mg QD vs. Tofacitinib 15 mg BID | Filgotinib 100 mg QD vs. Placebo vs. Tofacitinib 15 mg BID | Low ⨁⨁◯◯ | Low ⨁⨁◯◯ | Low ⨁⨁◯◯ | Downgrade | Very low ⨁◯◯◯ |
| Filgotinib 100 mg QD vs. Tofacitinib 3 mg BID | Filgotinib 100 mg QD vs. Placebo vs. Tofacitinib 3 mg BID | Low ⨁⨁◯◯ | Low ⨁⨁◯◯ | Low ⨁⨁◯◯ | Downgrade | Very low ⨁◯◯◯ |
| Filgotinib 100 mg QD vs. Tofacitinib 5 mg BID | Filgotinib 100 mg QD vs. Placebo vs. Tofacitinib 5 mg BID | Low ⨁⨁◯◯ | Low ⨁⨁◯◯ | Low ⨁⨁◯◯ | Downgrade | Very low ⨁◯◯◯ |
| Filgotinib 100 mg QD vs. Upadacitinib 15 mg QD | Filgotinib 100 mg QD vs. Placebo vs. Upadacitinib 15 mg QD | Low ⨁⨁◯◯ | Low ⨁⨁◯◯ | Low ⨁⨁◯◯ | Downgrade | Very low ⨁◯◯◯ |
| Filgotinib 100 mg QD vs. Upadacitinib 30 mg QD | Filgotinib 100 mg QD vs. Placebo vs. Upadacitinib 30 mg QD | Low ⨁⨁◯◯ | Low ⨁⨁◯◯ | Low ⨁⨁◯◯ | Downgrade | Very low ⨁◯◯◯ |
| Filgotinib 100 mg QD vs. Upadacitinib 45 mg QD | Filgotinib 100 mg QD vs. Placebo vs. Upadacitinib 45 mg QD | Low ⨁⨁◯◯ | Low ⨁⨁◯◯ | Low ⨁⨁◯◯ | Downgrade | Very low ⨁◯◯◯ |
| Filgotinib 200 mg QD vs. Ivarmacitinib | Filgotinib 200 mg QD vs. Placebo vs. Ivarmacitinib | Low ⨁⨁◯◯ | Low ⨁⨁◯◯ | Low ⨁⨁◯◯ | Downgrade | Very low ⨁◯◯◯ |
| Filgotinib 200 mg QD vs. Izencitinib | Filgotinib 200 mg QD vs. Placebo vs. Izencitinib | Low ⨁⨁◯◯ | Low ⨁⨁◯◯ | Low ⨁⨁◯◯ | Downgrade | Very low ⨁◯◯◯ |
| Filgotinib 200 mg QD vs. Peficitinib | Filgotinib 200 mg QD vs. Placebo vs. Peficitinib | Low ⨁⨁◯◯ | Low ⨁⨁◯◯ | Low ⨁⨁◯◯ | Downgrade | Very low ⨁◯◯◯ |
| Filgotinib 200 mg QD vs. Ritlecitinib | Filgotinib 200 mg QD vs. Placebo vs. Ritlecitinib | Low ⨁⨁◯◯ | Low ⨁⨁◯◯ | Low ⨁⨁◯◯ | Downgrade | Very low ⨁◯◯◯ |
| Filgotinib 200 mg QD vs. Tofacitinib 0.5 mg BID | Filgotinib 200 mg QD vs. Placebo vs. Tofacitinib 0.5 mg BID | Low ⨁⨁◯◯ | Low ⨁⨁◯◯ | Low ⨁⨁◯◯ | Downgrade | Very low ⨁◯◯◯ |
| Filgotinib 200 mg QD vs. Tofacitinib 1 mg BID | Filgotinib 200 mg QD vs. Placebo vs. Tofacitinib 1 mg BID | Low ⨁⨁◯◯ | Very low ⨁◯◯◯ | Very low ⨁◯◯◯ | Downgrade | Very low ⨁◯◯◯ |
| Filgotinib 200 mg QD vs. Tofacitinib 10 mg BID | Filgotinib 200 mg QD vs. Placebo vs. Tofacitinib 10 mg BID | Low ⨁⨁◯◯ | Low ⨁⨁◯◯ | Low ⨁⨁◯◯ | Downgrade | Very low ⨁◯◯◯ |
| Filgotinib 200 mg QD vs. Tofacitinib 15 mg BID | Filgotinib 200 mg QD vs. Placebo vs. Tofacitinib 15 mg BID | Low ⨁⨁◯◯ | Low ⨁⨁◯◯ | Low ⨁⨁◯◯ | Downgrade | Very low ⨁◯◯◯ |
| Filgotinib 200 mg QD vs. Tofacitinib 3 mg BID | Filgotinib 200 mg QD vs. Placebo vs. Tofacitinib 3 mg BID | Low ⨁⨁◯◯ | Low ⨁⨁◯◯ | Low ⨁⨁◯◯ | Downgrade | Very low ⨁◯◯◯ |
| Filgotinib 200 mg QD vs. Tofacitinib 5 mg BID | Filgotinib 200 mg QD vs. Placebo vs. Tofacitinib 5 mg BID | Low ⨁⨁◯◯ | Low ⨁⨁◯◯ | Low ⨁⨁◯◯ | Downgrade | Very low ⨁◯◯◯ |
| Filgotinib 200 mg QD vs. Upadacitinib 15 mg QD | Filgotinib 200 mg QD vs. Placebo vs. Upadacitinib 15 mg QD | Low ⨁⨁◯◯ | Low ⨁⨁◯◯ | Low ⨁⨁◯◯ | Downgrade | Very low ⨁◯◯◯ |
| Filgotinib 200 mg QD vs. Upadacitinib 30 mg QD | Filgotinib 200 mg QD vs. Placebo vs. Upadacitinib 30 mg QD | Low ⨁⨁◯◯ | Low ⨁⨁◯◯ | Low ⨁⨁◯◯ | Downgrade | Very low ⨁◯◯◯ |
| Filgotinib 200 mg QD vs. Upadacitinib 45 mg QD | Filgotinib 200 mg QD vs. Placebo vs. Upadacitinib 45 mg QD | Low ⨁⨁◯◯ | Low ⨁⨁◯◯ | Low ⨁⨁◯◯ | Downgrade | Very low ⨁◯◯◯ |
| Ivarmacitinib vs. Izencitinib | Ivarmacitinib vs. Placebo vs. Izencitinib | Low ⨁⨁◯◯ | Low ⨁⨁◯◯ | Low ⨁⨁◯◯ | Downgrade | Very low ⨁◯◯◯ |
| Ivarmacitinib vs. Peficitinib | Ivarmacitinib vs. Placebo vs. Peficitinib | Low ⨁⨁◯◯ | Low ⨁⨁◯◯ | Low ⨁⨁◯◯ | No downgrade | Low ⨁⨁◯◯ |
| Ivarmacitinib vs. Ritlecitinib | Ivarmacitinib vs. Placebo vs. Ritlecitinib | Low ⨁⨁◯◯ | Low ⨁⨁◯◯ | Low ⨁⨁◯◯ | No downgrade | Low ⨁⨁◯◯ |
| Ivarmacitinib vs. Tofacitinib 0.5 mg BID | Ivarmacitinib vs. Placebo vs. Tofacitinib 0.5 mg BID | Low ⨁⨁◯◯ | Low ⨁⨁◯◯ | Low ⨁⨁◯◯ | No downgrade | Low ⨁⨁◯◯ |
| Ivarmacitinib vs. Tofacitinib 1 mg BID | Ivarmacitinib vs. Placebo vs. Tofacitinib 1 mg BID | Low ⨁⨁◯◯ | Very low ⨁◯◯◯ | Very low ⨁◯◯◯ | Downgrade | Very low ⨁◯◯◯ |
| Ivarmacitinib vs. Tofacitinib 10 mg BID | Ivarmacitinib vs. Placebo vs. Tofacitinib 10 mg BID | Low ⨁⨁◯◯ | Low ⨁⨁◯◯ | Low ⨁⨁◯◯ | Downgrade | Very low ⨁◯◯◯ |
| Ivarmacitinib vs. Tofacitinib 15 mg BID | Ivarmacitinib vs. Placebo vs. Tofacitinib 15 mg BID | Low ⨁⨁◯◯ | Low ⨁⨁◯◯ | Low ⨁⨁◯◯ | Downgrade | Very low ⨁◯◯◯ |
| Ivarmacitinib vs. Tofacitinib 3 mg BID | Ivarmacitinib vs. Placebo vs. Tofacitinib 3 mg BID | Low ⨁⨁◯◯ | Low ⨁⨁◯◯ | Low ⨁⨁◯◯ | No downgrade | Low ⨁⨁◯◯ |
| Ivarmacitinib vs. Tofacitinib 5 mg BID | Ivarmacitinib vs. Placebo vs. Tofacitinib 5 mg BID | Low ⨁⨁◯◯ | Low ⨁⨁◯◯ | Low ⨁⨁◯◯ | Downgrade | Very low ⨁◯◯◯ |
| Ivarmacitinib vs. Upadacitinib 15 mg QD | Ivarmacitinib vs. Placebo vs. Upadacitinib 15 mg QD | Low ⨁⨁◯◯ | Low ⨁⨁◯◯ | Low ⨁⨁◯◯ | Downgrade | Very low ⨁◯◯◯ |
| Ivarmacitinib vs. Upadacitinib 30 mg QD | Ivarmacitinib vs. Placebo vs. Upadacitinib 30 mg QD | Low ⨁⨁◯◯ | Low ⨁⨁◯◯ | Low ⨁⨁◯◯ | Downgrade | Very low ⨁◯◯◯ |
| Ivarmacitinib vs. Upadacitinib 45 mg QD | Ivarmacitinib vs. Placebo vs. Upadacitinib 45 mg QD | Low ⨁⨁◯◯ | Low ⨁⨁◯◯ | Low ⨁⨁◯◯ | Downgrade | Very low ⨁◯◯◯ |
| Izencitinib vs. Peficitinib | Izencitinib vs. Placebo vs. Peficitinib | Low ⨁⨁◯◯ | Low ⨁⨁◯◯ | Low ⨁⨁◯◯ | Downgrade | Very low ⨁◯◯◯ |
| Izencitinib vs. Ritlecitinib | Izencitinib vs. Placebo vs. Ritlecitinib | Low ⨁⨁◯◯ | Low ⨁⨁◯◯ | Low ⨁⨁◯◯ | Downgrade | Very low ⨁◯◯◯ |
| Izencitinib vs. Tofacitinib 0.5 mg BID | Izencitinib vs. Placebo vs. Tofacitinib 0.5 mg BID | Low ⨁⨁◯◯ | Low ⨁⨁◯◯ | Low ⨁⨁◯◯ | Downgrade | Very low ⨁◯◯◯ |
| Izencitinib vs. Tofacitinib 1 mg BID | Izencitinib vs. Placebo vs. Tofacitinib 1 mg BID | Low ⨁⨁◯◯ | Very low ⨁◯◯◯ | Very low ⨁◯◯◯ | Downgrade | Very low ⨁◯◯◯ |
| Izencitinib vs. Tofacitinib 10 mg BID | Izencitinib vs. Placebo vs. Tofacitinib 10 mg BID | Low ⨁⨁◯◯ | Low ⨁⨁◯◯ | Low ⨁⨁◯◯ | Downgrade | Very low ⨁◯◯◯ |
| Izencitinib vs. Tofacitinib 15 mg BID | Izencitinib vs. Placebo vs. Tofacitinib 15 mg BID | Low ⨁⨁◯◯ | Low ⨁⨁◯◯ | Low ⨁⨁◯◯ | Downgrade | Very low ⨁◯◯◯ |
| Izencitinib vs. Tofacitinib 3 mg BID | Izencitinib vs. Placebo vs. Tofacitinib 3 mg BID | Low ⨁⨁◯◯ | Low ⨁⨁◯◯ | Low ⨁⨁◯◯ | Downgrade | Very low ⨁◯◯◯ |
| Izencitinib vs. Tofacitinib 5 mg BID | Izencitinib vs. Placebo vs. Tofacitinib 5 mg BID | Low ⨁⨁◯◯ | Low ⨁⨁◯◯ | Low ⨁⨁◯◯ | Downgrade | Very low ⨁◯◯◯ |
| Izencitinib vs. Upadacitinib 15 mg QD | Izencitinib vs. Placebo vs. Upadacitinib 15 mg QD | Low ⨁⨁◯◯ | Low ⨁⨁◯◯ | Low ⨁⨁◯◯ | Downgrade | Very low ⨁◯◯◯ |
| Izencitinib vs. Upadacitinib 30 mg QD | Izencitinib vs. Placebo vs. Upadacitinib 30 mg QD | Low ⨁⨁◯◯ | Low ⨁⨁◯◯ | Low ⨁⨁◯◯ | Downgrade | Very low ⨁◯◯◯ |
| Izencitinib vs. Upadacitinib 45 mg QD | Izencitinib vs. Placebo vs. Upadacitinib 45 mg QD | Low ⨁⨁◯◯ | Low ⨁⨁◯◯ | Low ⨁⨁◯◯ | Downgrade | Very low ⨁◯◯◯ |
| Peficitinib vs. Ritlecitinib | Peficitinib vs. Placebo vs. Ritlecitinib | Low ⨁⨁◯◯ | Low ⨁⨁◯◯ | Low ⨁⨁◯◯ | No downgrade | Low ⨁⨁◯◯ |
| Peficitinib vs. Tofacitinib 0.5 mg BID | Peficitinib vs. Placebo vs. Tofacitinib 0.5 mg BID | Low ⨁⨁◯◯ | Low ⨁⨁◯◯ | Low ⨁⨁◯◯ | No downgrade | Low ⨁⨁◯◯ |
| Peficitinib vs. Tofacitinib 1 mg BID | Peficitinib vs. Placebo vs. Tofacitinib 1 mg BID | Low ⨁⨁◯◯ | Very low ⨁◯◯◯ | Very low ⨁◯◯◯ | Downgrade | Very low ⨁◯◯◯ |
| Peficitinib vs. Tofacitinib 10 mg BID | Peficitinib vs. Placebo vs. Tofacitinib 10 mg BID | Low ⨁⨁◯◯ | Low ⨁⨁◯◯ | Low ⨁⨁◯◯ | Downgrade | Very low ⨁◯◯◯ |
| Peficitinib vs. Tofacitinib 15 mg BID | Peficitinib vs. Placebo vs. Tofacitinib 15 mg BID | Low ⨁⨁◯◯ | Low ⨁⨁◯◯ | Low ⨁⨁◯◯ | Downgrade | Very low ⨁◯◯◯ |
| Peficitinib vs. Tofacitinib 3 mg BID | Peficitinib vs. Placebo vs. Tofacitinib 3 mg BID | Low ⨁⨁◯◯ | Low ⨁⨁◯◯ | Low ⨁⨁◯◯ | No downgrade | Low ⨁⨁◯◯ |
| Peficitinib vs. Tofacitinib 5 mg BID | Peficitinib vs. Placebo vs. Tofacitinib 5 mg BID | Low ⨁⨁◯◯ | Low ⨁⨁◯◯ | Low ⨁⨁◯◯ | Downgrade | Very low ⨁◯◯◯ |
| Peficitinib vs. Upadacitinib 15 mg QD | Peficitinib vs. Placebo vs. Upadacitinib 15 mg QD | Low ⨁⨁◯◯ | Low ⨁⨁◯◯ | Low ⨁⨁◯◯ | Downgrade | Very low ⨁◯◯◯ |
| Peficitinib vs. Upadacitinib 30 mg QD | Peficitinib vs. Placebo vs. Upadacitinib 30 mg QD | Low ⨁⨁◯◯ | Low ⨁⨁◯◯ | Low ⨁⨁◯◯ | Downgrade | Very low ⨁◯◯◯ |
| Peficitinib vs. Upadacitinib 45 mg QD | Peficitinib vs. Placebo vs. Upadacitinib 45 mg QD | Low ⨁⨁◯◯ | Low ⨁⨁◯◯ | Low ⨁⨁◯◯ | Downgrade | Very low ⨁◯◯◯ |
| Placebo vs. Tofacitinib 0.5 mg BID | Placebo vs. Tofacitinib 10 mg BID vs. Tofacitinib 0.5 mg BID | Low ⨁⨁◯◯ | Low ⨁⨁◯◯ | Low ⨁⨁◯◯ | Downgrade | Very low ⨁◯◯◯ |
| Placebo vs. Tofacitinib 1 mg BID | Placebo vs. Tofacitinib 5 mg BID vs. Tofacitinib 1 mg BID | Low ⨁⨁◯◯ | Very low ⨁◯◯◯ | Very low ⨁◯◯◯ | Downgrade | Very low ⨁◯◯◯ |
| Placebo vs. Tofacitinib 10 mg BID | Placebo vs. Tofacitinib 5 mg BID vs. Tofacitinib 10 mg BID | Low ⨁⨁◯◯ | Low ⨁⨁◯◯ | Low ⨁⨁◯◯ | Downgrade | Very low ⨁◯◯◯ |
| Placebo vs. Tofacitinib 15 mg BID | Placebo vs. Tofacitinib 10 mg BID vs. Tofacitinib 15 mg BID | Low ⨁⨁◯◯ | Low ⨁⨁◯◯ | Low ⨁⨁◯◯ | Downgrade | Very low ⨁◯◯◯ |
| Placebo vs. Tofacitinib 3 mg BID | Placebo vs. Tofacitinib 10 mg BID vs. Tofacitinib 3 mg BID | Low ⨁⨁◯◯ | Low ⨁⨁◯◯ | Low ⨁⨁◯◯ | Downgrade | Very low ⨁◯◯◯ |
| Placebo vs. Tofacitinib 5 mg BID | Placebo vs. Tofacitinib 10 mg BID vs. Tofacitinib 5 mg BID | Low ⨁⨁◯◯ | Low ⨁⨁◯◯ | Low ⨁⨁◯◯ | Downgrade | Very low ⨁◯◯◯ |
| Placebo vs. Upadacitinib 15 mg QD | Placebo vs. Upadacitinib 45 mg QD vs. Upadacitinib 15 mg QD | Low ⨁⨁◯◯ | Low ⨁⨁◯◯ | Low ⨁⨁◯◯ | Downgrade | Very low ⨁◯◯◯ |
| Placebo vs. Upadacitinib 30 mg QD | Placebo vs. Upadacitinib 45 mg QD vs. Upadacitinib 30 mg QD | Low ⨁⨁◯◯ | Low ⨁⨁◯◯ | Low ⨁⨁◯◯ | Downgrade | Very low ⨁◯◯◯ |
| Placebo vs. Upadacitinib 45 mg QD | Placebo vs. Upadacitinib 15 mg QD vs. Upadacitinib 45 mg QD | Low ⨁⨁◯◯ | Low ⨁⨁◯◯ | Low ⨁⨁◯◯ | Downgrade | Very low ⨁◯◯◯ |
| Ritlecitinib vs. Tofacitinib 0.5 mg BID | Ritlecitinib vs. Placebo vs. Tofacitinib 0.5 mg BID | Low ⨁⨁◯◯ | Low ⨁⨁◯◯ | Low ⨁⨁◯◯ | No downgrade | Low ⨁⨁◯◯ |
| Ritlecitinib vs. Tofacitinib 1 mg BID | Ritlecitinib vs. Placebo vs. Tofacitinib 1 mg BID | Low ⨁⨁◯◯ | Very low ⨁◯◯◯ | Very low ⨁◯◯◯ | Downgrade | Very low ⨁◯◯◯ |
| Ritlecitinib vs. Tofacitinib 10 mg BID | Ritlecitinib vs. Placebo vs. Tofacitinib 10 mg BID | Low ⨁⨁◯◯ | Low ⨁⨁◯◯ | Low ⨁⨁◯◯ | Downgrade | Very low ⨁◯◯◯ |
| Ritlecitinib vs. Tofacitinib 15 mg BID | Ritlecitinib vs. Placebo vs. Tofacitinib 15 mg BID | Low ⨁⨁◯◯ | Low ⨁⨁◯◯ | Low ⨁⨁◯◯ | Downgrade | Very low ⨁◯◯◯ |
| Ritlecitinib vs. Tofacitinib 3 mg BID | Ritlecitinib vs. Placebo vs. Tofacitinib 3 mg BID | Low ⨁⨁◯◯ | Low ⨁⨁◯◯ | Low ⨁⨁◯◯ | No downgrade | Low ⨁⨁◯◯ |
| Ritlecitinib vs. Tofacitinib 5 mg BID | Ritlecitinib vs. Placebo vs. Tofacitinib 5 mg BID | Low ⨁⨁◯◯ | Low ⨁⨁◯◯ | Low ⨁⨁◯◯ | Downgrade | Very low ⨁◯◯◯ |
| Ritlecitinib vs. Upadacitinib 15 mg QD | Ritlecitinib vs. Placebo vs. Upadacitinib 15 mg QD | Low ⨁⨁◯◯ | Low ⨁⨁◯◯ | Low ⨁⨁◯◯ | Downgrade | Very low ⨁◯◯◯ |
| Ritlecitinib vs. Upadacitinib 30 mg QD | Ritlecitinib vs. Placebo vs. Upadacitinib 30 mg QD | Low ⨁⨁◯◯ | Low ⨁⨁◯◯ | Low ⨁⨁◯◯ | Downgrade | Very low ⨁◯◯◯ |
| Ritlecitinib vs. Upadacitinib 45 mg QD | Ritlecitinib vs. Placebo vs. Upadacitinib 45 mg QD | Low ⨁⨁◯◯ | Low ⨁⨁◯◯ | Low ⨁⨁◯◯ | Downgrade | Very low ⨁◯◯◯ |
| Tofacitinib 0.5 mg BID vs. Tofacitinib 1 mg BID | Tofacitinib 0.5 mg BID vs. Placebo vs. Tofacitinib 1 mg BID | Low ⨁⨁◯◯ | Very low ⨁◯◯◯ | Very low ⨁◯◯◯ | Downgrade | Very low ⨁◯◯◯ |
| Tofacitinib 0.5 mg BID vs. Tofacitinib 10 mg BID | Tofacitinib 0.5 mg BID vs. Placebo vs. Tofacitinib 10 mg BID | Low ⨁⨁◯◯ | Low ⨁⨁◯◯ | Low ⨁⨁◯◯ | Downgrade | Very low ⨁◯◯◯ |
| Tofacitinib 0.5 mg BID vs. Tofacitinib 15 mg BID | Tofacitinib 0.5 mg BID vs. Placebo vs. Tofacitinib 15 mg BID | Low ⨁⨁◯◯ | Low ⨁⨁◯◯ | Low ⨁⨁◯◯ | Downgrade | Very low ⨁◯◯◯ |
| Tofacitinib 0.5 mg BID vs. Tofacitinib 5 mg BID | Tofacitinib 0.5 mg BID vs. Placebo vs. Tofacitinib 5 mg BID | Low ⨁⨁◯◯ | Low ⨁⨁◯◯ | Low ⨁⨁◯◯ | Downgrade | Very low ⨁◯◯◯ |
| Tofacitinib 0.5 mg BID vs. Upadacitinib 15 mg QD | Tofacitinib 0.5 mg BID vs. Placebo vs. Upadacitinib 15 mg QD | Low ⨁⨁◯◯ | Low ⨁⨁◯◯ | Low ⨁⨁◯◯ | Downgrade | Very low ⨁◯◯◯ |
| Tofacitinib 0.5 mg BID vs. Upadacitinib 30 mg QD | Tofacitinib 0.5 mg BID vs. Placebo vs. Upadacitinib 30 mg QD | Low ⨁⨁◯◯ | Low ⨁⨁◯◯ | Low ⨁⨁◯◯ | Downgrade | Very low ⨁◯◯◯ |
| Tofacitinib 0.5 mg BID vs. Upadacitinib 45 mg QD | Tofacitinib 0.5 mg BID vs. Placebo vs. Upadacitinib 45 mg QD | Low ⨁⨁◯◯ | Low ⨁⨁◯◯ | Low ⨁⨁◯◯ | Downgrade | Very low ⨁◯◯◯ |
| Tofacitinib 1 mg BID vs. Tofacitinib 10 mg BID | Tofacitinib 1 mg BID vs. Placebo vs. Tofacitinib 10 mg BID | Very low ⨁◯◯◯ | Low ⨁⨁◯◯ | Very low ⨁◯◯◯ | Downgrade | Very low ⨁◯◯◯ |
| Tofacitinib 1 mg BID vs. Tofacitinib 15 mg BID | Tofacitinib 1 mg BID vs. Placebo vs. Tofacitinib 15 mg BID | Very low ⨁◯◯◯ | Low ⨁⨁◯◯ | Very low ⨁◯◯◯ | Downgrade | Very low ⨁◯◯◯ |
| Tofacitinib 1 mg BID vs. Tofacitinib 3 mg BID | Tofacitinib 1 mg BID vs. Placebo vs. Tofacitinib 3 mg BID | Very low ⨁◯◯◯ | Low ⨁⨁◯◯ | Very low ⨁◯◯◯ | Downgrade | Very low ⨁◯◯◯ |
| Tofacitinib 1 mg BID vs. Tofacitinib 5 mg BID | Tofacitinib 1 mg BID vs. Placebo vs. Tofacitinib 5 mg BID | Very low ⨁◯◯◯ | Low ⨁⨁◯◯ | Very low ⨁◯◯◯ | Downgrade | Very low ⨁◯◯◯ |
| Tofacitinib 1 mg BID vs. Upadacitinib 15 mg QD | Tofacitinib 1 mg BID vs. Placebo vs. Upadacitinib 15 mg QD | Very low ⨁◯◯◯ | Low ⨁⨁◯◯ | Very low ⨁◯◯◯ | Downgrade | Very low ⨁◯◯◯ |
| Tofacitinib 1 mg BID vs. Upadacitinib 30 mg QD | Tofacitinib 1 mg BID vs. Placebo vs. Upadacitinib 30 mg QD | Very low ⨁◯◯◯ | Low ⨁⨁◯◯ | Very low ⨁◯◯◯ | Downgrade | Very low ⨁◯◯◯ |
| Tofacitinib 1 mg BID vs. Upadacitinib 45 mg QD | Tofacitinib 1 mg BID vs. Placebo vs. Upadacitinib 45 mg QD | Very low ⨁◯◯◯ | Low ⨁⨁◯◯ | Very low ⨁◯◯◯ | Downgrade | Very low ⨁◯◯◯ |
| Tofacitinib 10 mg BID vs. Tofacitinib 15 mg BID | Tofacitinib 10 mg BID vs. Placebo vs. Tofacitinib 15 mg BID | Low ⨁⨁◯◯ | Low ⨁⨁◯◯ | Low ⨁⨁◯◯ | Downgrade | Very low ⨁◯◯◯ |
| Tofacitinib 10 mg BID vs. Tofacitinib 3 mg BID | Tofacitinib 10 mg BID vs. Placebo vs. Tofacitinib 3 mg BID | Low ⨁⨁◯◯ | Low ⨁⨁◯◯ | Low ⨁⨁◯◯ | Downgrade | Very low ⨁◯◯◯ |
| Tofacitinib 10 mg BID vs. Tofacitinib 5 mg BID | Tofacitinib 10 mg BID vs. Placebo vs. Tofacitinib 5 mg BID | Low ⨁⨁◯◯ | Low ⨁⨁◯◯ | Low ⨁⨁◯◯ | Downgrade | Very low ⨁◯◯◯ |
| Tofacitinib 10 mg BID vs. Upadacitinib 15 mg QD | Tofacitinib 10 mg BID vs. Placebo vs. Upadacitinib 15 mg QD | Low ⨁⨁◯◯ | Low ⨁⨁◯◯ | Low ⨁⨁◯◯ | Downgrade | Very low ⨁◯◯◯ |
| Tofacitinib 10 mg BID vs. Upadacitinib 30 mg QD | Tofacitinib 10 mg BID vs. Placebo vs. Upadacitinib 30 mg QD | Low ⨁⨁◯◯ | Low ⨁⨁◯◯ | Low ⨁⨁◯◯ | Downgrade | Very low ⨁◯◯◯ |
| Tofacitinib 10 mg BID vs. Upadacitinib 45 mg QD | Tofacitinib 10 mg BID vs. Placebo vs. Upadacitinib 45 mg QD | Low ⨁⨁◯◯ | Low ⨁⨁◯◯ | Low ⨁⨁◯◯ | Downgrade | Very low ⨁◯◯◯ |
| Tofacitinib 15 mg BID vs. Tofacitinib 3 mg BID | Tofacitinib 15 mg BID vs. Placebo vs. Tofacitinib 3 mg BID | Low ⨁⨁◯◯ | Low ⨁⨁◯◯ | Low ⨁⨁◯◯ | Downgrade | Very low ⨁◯◯◯ |
| Tofacitinib 15 mg BID vs. Tofacitinib 5 mg BID | Tofacitinib 15 mg BID vs. Placebo vs. Tofacitinib 5 mg BID | Low ⨁⨁◯◯ | Low ⨁⨁◯◯ | Low ⨁⨁◯◯ | Downgrade | Very low ⨁◯◯◯ |
| Tofacitinib 15 mg BID vs. Upadacitinib 15 mg QD | Tofacitinib 15 mg BID vs. Placebo vs. Upadacitinib 15 mg QD | Low ⨁⨁◯◯ | Low ⨁⨁◯◯ | Low ⨁⨁◯◯ | Downgrade | Very low ⨁◯◯◯ |
| Tofacitinib 15 mg BID vs. Upadacitinib 30 mg QD | Tofacitinib 15 mg BID vs. Placebo vs. Upadacitinib 30 mg QD | Low ⨁⨁◯◯ | Low ⨁⨁◯◯ | Low ⨁⨁◯◯ | Downgrade | Very low ⨁◯◯◯ |
| Tofacitinib 15 mg BID vs. Upadacitinib 45 mg QD | Tofacitinib 15 mg BID vs. Placebo vs. Upadacitinib 45 mg QD | Low ⨁⨁◯◯ | Low ⨁⨁◯◯ | Low ⨁⨁◯◯ | No downgrade | Low ⨁⨁◯◯ |
| Tofacitinib 3 mg BID vs. Tofacitinib 5 mg BID | Tofacitinib 3 mg BID vs. Placebo vs. Tofacitinib 5 mg BID | Low ⨁⨁◯◯ | Low ⨁⨁◯◯ | Low ⨁⨁◯◯ | Downgrade | Very low ⨁◯◯◯ |
| Tofacitinib 3 mg BID vs. Upadacitinib 15 mg QD | Tofacitinib 3 mg BID vs. Placebo vs. Upadacitinib 15 mg QD | Low ⨁⨁◯◯ | Low ⨁⨁◯◯ | Low ⨁⨁◯◯ | Downgrade | Very low ⨁◯◯◯ |
| Tofacitinib 3 mg BID vs. Upadacitinib 30 mg QD | Tofacitinib 3 mg BID vs. Placebo vs. Upadacitinib 30 mg QD | Low ⨁⨁◯◯ | Low ⨁⨁◯◯ | Low ⨁⨁◯◯ | Downgrade | Very low ⨁◯◯◯ |
| Tofacitinib 3 mg BID vs. Upadacitinib 45 mg QD | Tofacitinib 3 mg BID vs. Placebo vs. Upadacitinib 45 mg QD | Low ⨁⨁◯◯ | Low ⨁⨁◯◯ | Low ⨁⨁◯◯ | Downgrade | Very low ⨁◯◯◯ |
| Tofacitinib 5 mg BID vs. Upadacitinib 15 mg QD | Tofacitinib 5 mg BID vs. Placebo vs. Upadacitinib 15 mg QD | Low ⨁⨁◯◯ | Low ⨁⨁◯◯ | Low ⨁⨁◯◯ | Downgrade | Very low ⨁◯◯◯ |
| Tofacitinib 5 mg BID vs. Upadacitinib 30 mg QD | Tofacitinib 5 mg BID vs. Placebo vs. Upadacitinib 30 mg QD | Low ⨁⨁◯◯ | Low ⨁⨁◯◯ | Low ⨁⨁◯◯ | Downgrade | Very low ⨁◯◯◯ |
| Tofacitinib 5 mg BID vs. Upadacitinib 45 mg QD | Tofacitinib 5 mg BID vs. Placebo vs. Upadacitinib 45 mg QD | Low ⨁⨁◯◯ | Low ⨁⨁◯◯ | Low ⨁⨁◯◯ | Downgrade | Very low ⨁◯◯◯ |
| Upadacitinib 15 mg QD vs. Upadacitinib 45 mg QD | Upadacitinib 15 mg QD vs. Placebo vs. Upadacitinib 45 mg QD | Low ⨁⨁◯◯ | Low ⨁⨁◯◯ | Low ⨁⨁◯◯ | Downgrade | Very low ⨁◯◯◯ |
| Upadacitinib 30 mg QD vs. Upadacitinib 45 mg QD | Upadacitinib 30 mg QD vs. Placebo vs. Upadacitinib 45 mg QD | Low ⨁⨁◯◯ | Low ⨁⨁◯◯ | Low ⨁⨁◯◯ | Downgrade | Very low ⨁◯◯◯ |

MACE: major adverse cardiovascular events; QD: once daily; BID: twice daily.

**Supplementary table 10-2-2 Certainty of indirect evidence assessment for VTE (with dose consideration).**

| Comparison | First order loop | Start rating | | | Intransitivity | Preliminary certainty rating |
| --- | --- | --- | --- | --- | --- | --- |
|  |  | A→B Direct preliminary certainty rating | B→C Direct Preliminary certainty rating | Lowest certainty of the first loop components |  |  |
| Deucravacitinib vs. Brepocitinib | Deucravacitinib vs. Placebo vs. Brepocitinib | Low ⨁⨁◯◯ | Very low ⨁◯◯◯ | Very low ⨁◯◯◯ | No downgrade | Very low ⨁◯◯◯ |
| Filgotinib 100 mg QD vs. Brepocitinib | Filgotinib 100 mg QD vs. Placebo vs. Brepocitinib | Low ⨁⨁◯◯ | Low ⨁⨁◯◯ | Low ⨁⨁◯◯ | Downgrade | Very low ⨁◯◯◯ |
| Filgotinib 200 mg QD vs. Brepocitinib | Filgotinib 200 mg QD vs. Placebo vs. Brepocitinib | Low ⨁⨁◯◯ | Low ⨁⨁◯◯ | Low ⨁⨁◯◯ | Downgrade | Very low ⨁◯◯◯ |
| Ivarmacitinib vs. Brepocitinib | Ivarmacitinib vs. Placebo vs. Brepocitinib | Low ⨁⨁◯◯ | Low ⨁⨁◯◯ | Low ⨁⨁◯◯ | No downgrade | Low ⨁⨁◯◯ |
| Izencitinib vs. Brepocitinib | Izencitinib vs. Placebo vs. Brepocitinib | Low ⨁⨁◯◯ | Low ⨁⨁◯◯ | Low ⨁⨁◯◯ | Downgrade | Very low ⨁◯◯◯ |
| Peficitinib vs. Brepocitinib | Peficitinib vs. Placebo vs. Brepocitinib | Low ⨁⨁◯◯ | Low ⨁⨁◯◯ | Low ⨁⨁◯◯ | No downgrade | Low ⨁⨁◯◯ |
| Tofacitinib 0.5 mg BID vs. Brepocitinib | Tofacitinib 0.5 mg BID vs. Placebo vs. Brepocitinib | Low ⨁⨁◯◯ | Low ⨁⨁◯◯ | Low ⨁⨁◯◯ | No downgrade | Low ⨁⨁◯◯ |
| Tofacitinib 1 mg BID vs. Brepocitinib | Tofacitinib 1 mg BID vs. Placebo vs. Brepocitinib | Very low ⨁◯◯◯ | Low ⨁⨁◯◯ | Very low ⨁◯◯◯ | Downgrade | Very low ⨁◯◯◯ |
| Tofacitinib 10 mg BID vs. Brepocitinib | Tofacitinib 10 mg BID vs. Placebo vs. Brepocitinib | Low ⨁⨁◯◯ | Low ⨁⨁◯◯ | Low ⨁⨁◯◯ | Downgrade | Very low ⨁◯◯◯ |
| Tofacitinib 15 mg BID vs. Brepocitinib | Tofacitinib 15 mg BID vs. Placebo vs. Brepocitinib | Low ⨁⨁◯◯ | Low ⨁⨁◯◯ | Low ⨁⨁◯◯ | Downgrade | Very low ⨁◯◯◯ |
| Tofacitinib 3 mg BID vs. Brepocitinib | Tofacitinib 3 mg BID vs. Placebo vs. Brepocitinib | Low ⨁⨁◯◯ | Low ⨁⨁◯◯ | Low ⨁⨁◯◯ | No downgrade | Low ⨁⨁◯◯ |
| Tofacitinib 5 mg BID vs. Brepocitinib | Tofacitinib 5 mg BID vs. Placebo vs. Brepocitinib | Low ⨁⨁◯◯ | Low ⨁⨁◯◯ | Low ⨁⨁◯◯ | Downgrade | Very low ⨁◯◯◯ |
| Upadacitinib 15 mg QD vs. Brepocitinib | Upadacitinib 15 mg QD vs. Placebo vs. Brepocitinib | Low ⨁⨁◯◯ | Low ⨁⨁◯◯ | Low ⨁⨁◯◯ | Downgrade | Very low ⨁◯◯◯ |
| Upadacitinib 30 mg QD vs. Brepocitinib | Upadacitinib 30 mg QD vs. Placebo vs. Brepocitinib | Low ⨁⨁◯◯ | Low ⨁⨁◯◯ | Low ⨁⨁◯◯ | Downgrade | Very low ⨁◯◯◯ |
| Upadacitinib 45 mg QD vs. Brepocitinib | Upadacitinib 45 mg QD vs. Placebo vs. Brepocitinib | Low ⨁⨁◯◯ | Low ⨁⨁◯◯ | Low ⨁⨁◯◯ | Downgrade | Very low ⨁◯◯◯ |
| Deucravacitinib vs. Filgotinib 100 mg QD | Deucravacitinib vs. Placebo vs. Filgotinib 100 mg QD | Very low ⨁◯◯◯ | Low ⨁⨁◯◯ | Very low ⨁◯◯◯ | Downgrade | Very low ⨁◯◯◯ |
| Deucravacitinib vs. Filgotinib 200 mg QD | Deucravacitinib vs. Placebo vs. Filgotinib 200 mg QD | Very low ⨁◯◯◯ | Low ⨁⨁◯◯ | Very low ⨁◯◯◯ | Downgrade | Very low ⨁◯◯◯ |
| Deucravacitinib vs. Ivarmacitinib | Deucravacitinib vs. Placebo vs. Ivarmacitinib | Very low ⨁◯◯◯ | Low ⨁⨁◯◯ | Very low ⨁◯◯◯ | No downgrade | Very low ⨁◯◯◯ |
| Deucravacitinib vs. Izencitinib | Deucravacitinib vs. Placebo vs. Izencitinib | Very low ⨁◯◯◯ | Low ⨁⨁◯◯ | Very low ⨁◯◯◯ | Downgrade | Very low ⨁◯◯◯ |
| Deucravacitinib vs. Peficitinib | Deucravacitinib vs. Placebo vs. Peficitinib | Very low ⨁◯◯◯ | Low ⨁⨁◯◯ | Very low ⨁◯◯◯ | No downgrade | Very low ⨁◯◯◯ |
| Deucravacitinib vs. Ritlecitinib | Deucravacitinib vs. Placebo vs. Ritlecitinib | Very low ⨁◯◯◯ | Low ⨁⨁◯◯ | Very low ⨁◯◯◯ | No downgrade | Very low ⨁◯◯◯ |
| Deucravacitinib vs. Tofacitinib 0.5 mg BID | Deucravacitinib vs. Placebo vs. Tofacitinib 0.5 mg BID | Very low ⨁◯◯◯ | Low ⨁⨁◯◯ | Very low ⨁◯◯◯ | No downgrade | Very low ⨁◯◯◯ |
| Deucravacitinib vs. Tofacitinib 1 mg BID | Deucravacitinib vs. Placebo vs. Tofacitinib 1 mg BID | Very low ⨁◯◯◯ | Very low ⨁◯◯◯ | Very low ⨁◯◯◯ | Downgrade | Very low ⨁◯◯◯ |
| Deucravacitinib vs. Tofacitinib 10 mg BID | Deucravacitinib vs. Placebo vs. Tofacitinib 10 mg BID | Very low ⨁◯◯◯ | Low ⨁⨁◯◯ | Very low ⨁◯◯◯ | Downgrade | Very low ⨁◯◯◯ |
| Deucravacitinib vs. Tofacitinib 15 mg BID | Deucravacitinib vs. Placebo vs. Tofacitinib 15 mg BID | Very low ⨁◯◯◯ | Low ⨁⨁◯◯ | Very low ⨁◯◯◯ | Downgrade | Very low ⨁◯◯◯ |
| Deucravacitinib vs. Tofacitinib 3 mg BID | Deucravacitinib vs. Placebo vs. Tofacitinib 3 mg BID | Very low ⨁◯◯◯ | Low ⨁⨁◯◯ | Very low ⨁◯◯◯ | No downgrade | Very low ⨁◯◯◯ |
| Deucravacitinib vs. Tofacitinib 5 mg BID | Deucravacitinib vs. Placebo vs. Tofacitinib 5 mg BID | Very low ⨁◯◯◯ | Low ⨁⨁◯◯ | Very low ⨁◯◯◯ | Downgrade | Very low ⨁◯◯◯ |
| Deucravacitinib vs. Upadacitinib 15 mg QD | Deucravacitinib vs. Placebo vs. Upadacitinib 15 mg QD | Very low ⨁◯◯◯ | Low ⨁⨁◯◯ | Very low ⨁◯◯◯ | Downgrade | Very low ⨁◯◯◯ |
| Deucravacitinib vs. Upadacitinib 30 mg QD | Deucravacitinib vs. Placebo vs. Upadacitinib 30 mg QD | Very low ⨁◯◯◯ | Low ⨁⨁◯◯ | Very low ⨁◯◯◯ | Downgrade | Very low ⨁◯◯◯ |
| Deucravacitinib vs. Upadacitinib 45 mg QD | Deucravacitinib vs. Placebo vs. Upadacitinib 45 mg QD | Very low ⨁◯◯◯ | Low ⨁⨁◯◯ | Very low ⨁◯◯◯ | Downgrade | Very low ⨁◯◯◯ |
| Filgotinib 100 mg QD vs. Filgotinib 200 mg QD | Filgotinib 100 mg QD vs. Placebo vs. Filgotinib 200 mg QD | Low ⨁⨁◯◯ | Low ⨁⨁◯◯ | Low ⨁⨁◯◯ | No downgrade | Low ⨁⨁◯◯ |
| Filgotinib 100 mg QD vs. Ivarmacitinib | Filgotinib 100 mg QD vs. Placebo vs. Ivarmacitinib | Low ⨁⨁◯◯ | Low ⨁⨁◯◯ | Low ⨁⨁◯◯ | Downgrade | Very low ⨁◯◯◯ |
| Filgotinib 100 mg QD vs. Izencitinib | Filgotinib 100 mg QD vs. Placebo vs. Izencitinib | Low ⨁⨁◯◯ | Low ⨁⨁◯◯ | Low ⨁⨁◯◯ | Downgrade | Very low ⨁◯◯◯ |
| Filgotinib 100 mg QD vs. Peficitinib | Filgotinib 100 mg QD vs. Placebo vs. Peficitinib | Low ⨁⨁◯◯ | Low ⨁⨁◯◯ | Low ⨁⨁◯◯ | Downgrade | Very low ⨁◯◯◯ |
| Filgotinib 100 mg QD vs. Placebo | Filgotinib 100 mg QD vs. Filgotinib 200 mg QD vs. Placebo | Low ⨁⨁◯◯ | Low ⨁⨁◯◯ | Low ⨁⨁◯◯ | Downgrade | Very low ⨁◯◯◯ |
| Filgotinib 100 mg QD vs. Ritlecitinib | Filgotinib 100 mg QD vs. Placebo vs. Ritlecitinib | Low ⨁⨁◯◯ | Low ⨁⨁◯◯ | Low ⨁⨁◯◯ | Downgrade | Very low ⨁◯◯◯ |
| Filgotinib 100 mg QD vs. Tofacitinib 0.5 mg BID | Filgotinib 100 mg QD vs. Placebo vs. Tofacitinib 0.5 mg BID | Low ⨁⨁◯◯ | Low ⨁⨁◯◯ | Low ⨁⨁◯◯ | Downgrade | Very low ⨁◯◯◯ |
| Filgotinib 100 mg QD vs. Tofacitinib 1 mg BID | Filgotinib 100 mg QD vs. Placebo vs. Tofacitinib 1 mg BID | Low ⨁⨁◯◯ | Very low ⨁◯◯◯ | Very low ⨁◯◯◯ | Downgrade | Very low ⨁◯◯◯ |
| Filgotinib 100 mg QD vs. Tofacitinib 10 mg BID | Filgotinib 100 mg QD vs. Placebo vs. Tofacitinib 10 mg BID | Low ⨁⨁◯◯ | Low ⨁⨁◯◯ | Low ⨁⨁◯◯ | Downgrade | Very low ⨁◯◯◯ |
| Filgotinib 100 mg QD vs. Tofacitinib 15 mg BID | Filgotinib 100 mg QD vs. Placebo vs. Tofacitinib 15 mg BID | Low ⨁⨁◯◯ | Low ⨁⨁◯◯ | Low ⨁⨁◯◯ | Downgrade | Very low ⨁◯◯◯ |
| Filgotinib 100 mg QD vs. Tofacitinib 3 mg BID | Filgotinib 100 mg QD vs. Placebo vs. Tofacitinib 3 mg BID | Low ⨁⨁◯◯ | Low ⨁⨁◯◯ | Low ⨁⨁◯◯ | Downgrade | Very low ⨁◯◯◯ |
| Filgotinib 100 mg QD vs. Tofacitinib 5 mg BID | Filgotinib 100 mg QD vs. Placebo vs. Tofacitinib 5 mg BID | Low ⨁⨁◯◯ | Low ⨁⨁◯◯ | Low ⨁⨁◯◯ | Downgrade | Very low ⨁◯◯◯ |
| Filgotinib 100 mg QD vs. Upadacitinib 15 mg QD | Filgotinib 100 mg QD vs. Placebo vs. Upadacitinib 15 mg QD | Low ⨁⨁◯◯ | Low ⨁⨁◯◯ | Low ⨁⨁◯◯ | Downgrade | Very low ⨁◯◯◯ |
| Filgotinib 100 mg QD vs. Upadacitinib 30 mg QD | Filgotinib 100 mg QD vs. Placebo vs. Upadacitinib 30 mg QD | Low ⨁⨁◯◯ | Low ⨁⨁◯◯ | Low ⨁⨁◯◯ | Downgrade | Very low ⨁◯◯◯ |
| Filgotinib 100 mg QD vs. Upadacitinib 45 mg QD | Filgotinib 100 mg QD vs. Placebo vs. Upadacitinib 45 mg QD | Low ⨁⨁◯◯ | Low ⨁⨁◯◯ | Low ⨁⨁◯◯ | Downgrade | Very low ⨁◯◯◯ |
| Filgotinib 200 mg QD vs. Ivarmacitinib | Filgotinib 200 mg QD vs. Placebo vs. Ivarmacitinib | Low ⨁⨁◯◯ | Low ⨁⨁◯◯ | Low ⨁⨁◯◯ | Downgrade | Very low ⨁◯◯◯ |
| Filgotinib 200 mg QD vs. Izencitinib | Filgotinib 200 mg QD vs. Placebo vs. Izencitinib | Low ⨁⨁◯◯ | Low ⨁⨁◯◯ | Low ⨁⨁◯◯ | Downgrade | Very low ⨁◯◯◯ |
| Filgotinib 200 mg QD vs. Peficitinib | Filgotinib 200 mg QD vs. Placebo vs. Peficitinib | Low ⨁⨁◯◯ | Low ⨁⨁◯◯ | Low ⨁⨁◯◯ | Downgrade | Very low ⨁◯◯◯ |
| Filgotinib 200 mg QD vs. Placebo | Filgotinib 200 mg QD vs. Filgotinib 100 mg QD vs. Placebo | Low ⨁⨁◯◯ | Low ⨁⨁◯◯ | Low ⨁⨁◯◯ | Downgrade | Very low ⨁◯◯◯ |
| Filgotinib 200 mg QD vs. Ritlecitinib | Filgotinib 200 mg QD vs. Placebo vs. Ritlecitinib | Low ⨁⨁◯◯ | Low ⨁⨁◯◯ | Low ⨁⨁◯◯ | Downgrade | Very low ⨁◯◯◯ |
| Filgotinib 200 mg QD vs. Tofacitinib 0.5 mg BID | Filgotinib 200 mg QD vs. Placebo vs. Tofacitinib 0.5 mg BID | Low ⨁⨁◯◯ | Low ⨁⨁◯◯ | Low ⨁⨁◯◯ | Downgrade | Very low ⨁◯◯◯ |
| Filgotinib 200 mg QD vs. Tofacitinib 1 mg BID | Filgotinib 200 mg QD vs. Placebo vs. Tofacitinib 1 mg BID | Low ⨁⨁◯◯ | Very low ⨁◯◯◯ | Very low ⨁◯◯◯ | Downgrade | Very low ⨁◯◯◯ |
| Filgotinib 200 mg QD vs. Tofacitinib 10 mg BID | Filgotinib 200 mg QD vs. Placebo vs. Tofacitinib 10 mg BID | Low ⨁⨁◯◯ | Low ⨁⨁◯◯ | Low ⨁⨁◯◯ | Downgrade | Very low ⨁◯◯◯ |
| Filgotinib 200 mg QD vs. Tofacitinib 15 mg BID | Filgotinib 200 mg QD vs. Placebo vs. Tofacitinib 15 mg BID | Low ⨁⨁◯◯ | Low ⨁⨁◯◯ | Low ⨁⨁◯◯ | Downgrade | Very low ⨁◯◯◯ |
| Filgotinib 200 mg QD vs. Tofacitinib 3 mg BID | Filgotinib 200 mg QD vs. Placebo vs. Tofacitinib 3 mg BID | Low ⨁⨁◯◯ | Low ⨁⨁◯◯ | Low ⨁⨁◯◯ | Downgrade | Very low ⨁◯◯◯ |
| Filgotinib 200 mg QD vs. Tofacitinib 5 mg BID | Filgotinib 200 mg QD vs. Placebo vs. Tofacitinib 5 mg BID | Low ⨁⨁◯◯ | Low ⨁⨁◯◯ | Low ⨁⨁◯◯ | Downgrade | Very low ⨁◯◯◯ |
| Filgotinib 200 mg QD vs. Upadacitinib 15 mg QD | Filgotinib 200 mg QD vs. Placebo vs. Upadacitinib 15 mg QD | Low ⨁⨁◯◯ | Low ⨁⨁◯◯ | Low ⨁⨁◯◯ | Downgrade | Very low ⨁◯◯◯ |
| Filgotinib 200 mg QD vs. Upadacitinib 30 mg QD | Filgotinib 200 mg QD vs. Placebo vs. Upadacitinib 30 mg QD | Low ⨁⨁◯◯ | Low ⨁⨁◯◯ | Low ⨁⨁◯◯ | Downgrade | Very low ⨁◯◯◯ |
| Filgotinib 200 mg QD vs. Upadacitinib 45 mg QD | Filgotinib 200 mg QD vs. Placebo vs. Upadacitinib 45 mg QD | Low ⨁⨁◯◯ | Low ⨁⨁◯◯ | Low ⨁⨁◯◯ | Downgrade | Very low ⨁◯◯◯ |
| Ivarmacitinib vs. Izencitinib | Ivarmacitinib vs. Placebo vs. Izencitinib | Low ⨁⨁◯◯ | Low ⨁⨁◯◯ | Low ⨁⨁◯◯ | Downgrade | Very low ⨁◯◯◯ |
| Ivarmacitinib vs. Peficitinib | Ivarmacitinib vs. Placebo vs. Peficitinib | Low ⨁⨁◯◯ | Low ⨁⨁◯◯ | Low ⨁⨁◯◯ | No downgrade | Low ⨁⨁◯◯ |
| Ivarmacitinib vs. Ritlecitinib | Ivarmacitinib vs. Placebo vs. Ritlecitinib | Low ⨁⨁◯◯ | Low ⨁⨁◯◯ | Low ⨁⨁◯◯ | No downgrade | Low ⨁⨁◯◯ |
| Ivarmacitinib vs. Tofacitinib 0.5 mg BID | Ivarmacitinib vs. Placebo vs. Tofacitinib 0.5 mg BID | Low ⨁⨁◯◯ | Low ⨁⨁◯◯ | Low ⨁⨁◯◯ | No downgrade | Low ⨁⨁◯◯ |
| Ivarmacitinib vs. Tofacitinib 1 mg BID | Ivarmacitinib vs. Placebo vs. Tofacitinib 1 mg BID | Low ⨁⨁◯◯ | Very low ⨁◯◯◯ | Very low ⨁◯◯◯ | Downgrade | Very low ⨁◯◯◯ |
| Ivarmacitinib vs. Tofacitinib 10 mg BID | Ivarmacitinib vs. Placebo vs. Tofacitinib 10 mg BID | Low ⨁⨁◯◯ | Low ⨁⨁◯◯ | Low ⨁⨁◯◯ | Downgrade | Very low ⨁◯◯◯ |
| Ivarmacitinib vs. Tofacitinib 15 mg BID | Ivarmacitinib vs. Placebo vs. Tofacitinib 15 mg BID | Low ⨁⨁◯◯ | Low ⨁⨁◯◯ | Low ⨁⨁◯◯ | Downgrade | Very low ⨁◯◯◯ |
| Ivarmacitinib vs. Tofacitinib 3 mg BID | Ivarmacitinib vs. Placebo vs. Tofacitinib 3 mg BID | Low ⨁⨁◯◯ | Low ⨁⨁◯◯ | Low ⨁⨁◯◯ | No downgrade | Low ⨁⨁◯◯ |
| Ivarmacitinib vs. Tofacitinib 5 mg BID | Ivarmacitinib vs. Placebo vs. Tofacitinib 5 mg BID | Low ⨁⨁◯◯ | Low ⨁⨁◯◯ | Low ⨁⨁◯◯ | Downgrade | Very low ⨁◯◯◯ |
| Ivarmacitinib vs. Upadacitinib 15 mg QD | Ivarmacitinib vs. Placebo vs. Upadacitinib 15 mg QD | Low ⨁⨁◯◯ | Low ⨁⨁◯◯ | Low ⨁⨁◯◯ | Downgrade | Very low ⨁◯◯◯ |
| Ivarmacitinib vs. Upadacitinib 30 mg QD | Ivarmacitinib vs. Placebo vs. Upadacitinib 30 mg QD | Low ⨁⨁◯◯ | Low ⨁⨁◯◯ | Low ⨁⨁◯◯ | Downgrade | Very low ⨁◯◯◯ |
| Ivarmacitinib vs. Upadacitinib 45 mg QD | Ivarmacitinib vs. Placebo vs. Upadacitinib 45 mg QD | Low ⨁⨁◯◯ | Low ⨁⨁◯◯ | Low ⨁⨁◯◯ | Downgrade | Very low ⨁◯◯◯ |
| Izencitinib vs. Peficitinib | Izencitinib vs. Placebo vs. Peficitinib | Low ⨁⨁◯◯ | Low ⨁⨁◯◯ | Low ⨁⨁◯◯ | Downgrade | Very low ⨁◯◯◯ |
| Izencitinib vs. Ritlecitinib | Izencitinib vs. Placebo vs. Ritlecitinib | Low ⨁⨁◯◯ | Low ⨁⨁◯◯ | Low ⨁⨁◯◯ | Downgrade | Very low ⨁◯◯◯ |
| Izencitinib vs. Tofacitinib 0.5 mg BID | Izencitinib vs. Placebo vs. Tofacitinib 0.5 mg BID | Low ⨁⨁◯◯ | Low ⨁⨁◯◯ | Low ⨁⨁◯◯ | Downgrade | Very low ⨁◯◯◯ |
| Izencitinib vs. Tofacitinib 1 mg BID | Izencitinib vs. Placebo vs. Tofacitinib 1 mg BID | Low ⨁⨁◯◯ | Very low ⨁◯◯◯ | Very low ⨁◯◯◯ | Downgrade | Very low ⨁◯◯◯ |
| Izencitinib vs. Tofacitinib 10 mg BID | Izencitinib vs. Placebo vs. Tofacitinib 10 mg BID | Low ⨁⨁◯◯ | Low ⨁⨁◯◯ | Low ⨁⨁◯◯ | Downgrade | Very low ⨁◯◯◯ |
| Izencitinib vs. Tofacitinib 15 mg BID | Izencitinib vs. Placebo vs. Tofacitinib 15 mg BID | Low ⨁⨁◯◯ | Low ⨁⨁◯◯ | Low ⨁⨁◯◯ | Downgrade | Very low ⨁◯◯◯ |
| Izencitinib vs. Tofacitinib 3 mg BID | Izencitinib vs. Placebo vs. Tofacitinib 3 mg BID | Low ⨁⨁◯◯ | Low ⨁⨁◯◯ | Low ⨁⨁◯◯ | Downgrade | Very low ⨁◯◯◯ |
| Izencitinib vs. Tofacitinib 5 mg BID | Izencitinib vs. Placebo vs. Tofacitinib 5 mg BID | Low ⨁⨁◯◯ | Low ⨁⨁◯◯ | Low ⨁⨁◯◯ | Downgrade | Very low ⨁◯◯◯ |
| Izencitinib vs. Upadacitinib 15 mg QD | Izencitinib vs. Placebo vs. Upadacitinib 15 mg QD | Low ⨁⨁◯◯ | Low ⨁⨁◯◯ | Low ⨁⨁◯◯ | Downgrade | Very low ⨁◯◯◯ |
| Izencitinib vs. Upadacitinib 30 mg QD | Izencitinib vs. Placebo vs. Upadacitinib 30 mg QD | Low ⨁⨁◯◯ | Low ⨁⨁◯◯ | Low ⨁⨁◯◯ | Downgrade | Very low ⨁◯◯◯ |
| Izencitinib vs. Upadacitinib 45 mg QD | Izencitinib vs. Placebo vs. Upadacitinib 45 mg QD | Low ⨁⨁◯◯ | Low ⨁⨁◯◯ | Low ⨁⨁◯◯ | Downgrade | Very low ⨁◯◯◯ |
| Peficitinib vs. Ritlecitinib | Peficitinib vs. Placebo vs. Ritlecitinib | Low ⨁⨁◯◯ | Low ⨁⨁◯◯ | Low ⨁⨁◯◯ | No downgrade | Low ⨁⨁◯◯ |
| Peficitinib vs. Tofacitinib 0.5 mg BID | Peficitinib vs. Placebo vs. Tofacitinib 0.5 mg BID | Low ⨁⨁◯◯ | Low ⨁⨁◯◯ | Low ⨁⨁◯◯ | No downgrade | Low ⨁⨁◯◯ |
| Peficitinib vs. Tofacitinib 1 mg BID | Peficitinib vs. Placebo vs. Tofacitinib 1 mg BID | Low ⨁⨁◯◯ | Very low ⨁◯◯◯ | Very low ⨁◯◯◯ | Downgrade | Very low ⨁◯◯◯ |
| Peficitinib vs. Tofacitinib 10 mg BID | Peficitinib vs. Placebo vs. Tofacitinib 10 mg BID | Low ⨁⨁◯◯ | Low ⨁⨁◯◯ | Low ⨁⨁◯◯ | Downgrade | Very low ⨁◯◯◯ |
| Peficitinib vs. Tofacitinib 15 mg BID | Peficitinib vs. Placebo vs. Tofacitinib 15 mg BID | Low ⨁⨁◯◯ | Low ⨁⨁◯◯ | Low ⨁⨁◯◯ | Downgrade | Very low ⨁◯◯◯ |
| Peficitinib vs. Tofacitinib 3 mg BID | Peficitinib vs. Placebo vs. Tofacitinib 3 mg BID | Low ⨁⨁◯◯ | Low ⨁⨁◯◯ | Low ⨁⨁◯◯ | No downgrade | Low ⨁⨁◯◯ |
| Peficitinib vs. Tofacitinib 5 mg BID | Peficitinib vs. Placebo vs. Tofacitinib 5 mg BID | Low ⨁⨁◯◯ | Low ⨁⨁◯◯ | Low ⨁⨁◯◯ | Downgrade | Very low ⨁◯◯◯ |
| Peficitinib vs. Upadacitinib 15 mg QD | Peficitinib vs. Placebo vs. Upadacitinib 15 mg QD | Low ⨁⨁◯◯ | Low ⨁⨁◯◯ | Low ⨁⨁◯◯ | Downgrade | Very low ⨁◯◯◯ |
| Peficitinib vs. Upadacitinib 30 mg QD | Peficitinib vs. Placebo vs. Upadacitinib 30 mg QD | Low ⨁⨁◯◯ | Low ⨁⨁◯◯ | Low ⨁⨁◯◯ | Downgrade | Very low ⨁◯◯◯ |
| Peficitinib vs. Upadacitinib 45 mg QD | Peficitinib vs. Placebo vs. Upadacitinib 45 mg QD | Low ⨁⨁◯◯ | Low ⨁⨁◯◯ | Low ⨁⨁◯◯ | Downgrade | Very low ⨁◯◯◯ |
| Placebo vs. Tofacitinib 0.5 mg BID | Placebo vs. Tofacitinib 10 mg BID vs. Tofacitinib 0.5 mg BID | Low ⨁⨁◯◯ | Low ⨁⨁◯◯ | Low ⨁⨁◯◯ | Downgrade | Very low ⨁◯◯◯ |
| Placebo vs. Tofacitinib 1 mg BID | Placebo vs. Tofacitinib 5 mg BID vs. Tofacitinib 1 mg BID | Low ⨁⨁◯◯ | Very low ⨁◯◯◯ | Very low ⨁◯◯◯ | Downgrade | Very low ⨁◯◯◯ |
| Placebo vs. Tofacitinib 10 mg BID | Placebo vs. Tofacitinib 5 mg BID vs. Tofacitinib 10 mg BID | Low ⨁⨁◯◯ | Low ⨁⨁◯◯ | Low ⨁⨁◯◯ | Downgrade | Very low ⨁◯◯◯ |
| Placebo vs. Tofacitinib 15 mg BID | Placebo vs. Tofacitinib 10 mg BID vs. Tofacitinib 15 mg BID | Low ⨁⨁◯◯ | Low ⨁⨁◯◯ | Low ⨁⨁◯◯ | Downgrade | Very low ⨁◯◯◯ |
| Placebo vs. Tofacitinib 3 mg BID | Placebo vs. Tofacitinib 10 mg BID vs. Tofacitinib 3 mg BID | Low ⨁⨁◯◯ | Low ⨁⨁◯◯ | Low ⨁⨁◯◯ | Downgrade | Very low ⨁◯◯◯ |
| Placebo vs. Tofacitinib 5 mg BID | Placebo vs. Tofacitinib 10 mg BID vs. Tofacitinib 5 mg BID | Low ⨁⨁◯◯ | Low ⨁⨁◯◯ | Low ⨁⨁◯◯ | Downgrade | Very low ⨁◯◯◯ |
| Placebo vs. Upadacitinib 15 mg QD | Placebo vs. Upadacitinib 45 mg QD vs. Upadacitinib 15 mg QD | Low ⨁⨁◯◯ | Low ⨁⨁◯◯ | Low ⨁⨁◯◯ | Downgrade | Very low ⨁◯◯◯ |
| Placebo vs. Upadacitinib 30 mg QD | Placebo vs. Upadacitinib 45 mg QD vs. Upadacitinib 30 mg QD | Low ⨁⨁◯◯ | Low ⨁⨁◯◯ | Low ⨁⨁◯◯ | Downgrade | Very low ⨁◯◯◯ |
| Placebo vs. Upadacitinib 45 mg QD | Placebo vs. Upadacitinib 15 mg QD vs. Upadacitinib 45 mg QD | Low ⨁⨁◯◯ | Low ⨁⨁◯◯ | Low ⨁⨁◯◯ | Downgrade | Very low ⨁◯◯◯ |
| Ritlecitinib vs. Tofacitinib 0.5 mg BID | Ritlecitinib vs. Placebo vs. Tofacitinib 0.5 mg BID | Low ⨁⨁◯◯ | Low ⨁⨁◯◯ | Low ⨁⨁◯◯ | No downgrade | Low ⨁⨁◯◯ |
| Ritlecitinib vs. Tofacitinib 1 mg BID | Ritlecitinib vs. Placebo vs. Tofacitinib 1 mg BID | Low ⨁⨁◯◯ | Very low ⨁◯◯◯ | Very low ⨁◯◯◯ | Downgrade | Very low ⨁◯◯◯ |
| Ritlecitinib vs. Tofacitinib 10 mg BID | Ritlecitinib vs. Placebo vs. Tofacitinib 10 mg BID | Low ⨁⨁◯◯ | Low ⨁⨁◯◯ | Low ⨁⨁◯◯ | Downgrade | Very low ⨁◯◯◯ |
| Ritlecitinib vs. Tofacitinib 15 mg BID | Ritlecitinib vs. Placebo vs. Tofacitinib 15 mg BID | Low ⨁⨁◯◯ | Low ⨁⨁◯◯ | Low ⨁⨁◯◯ | Downgrade | Very low ⨁◯◯◯ |
| Ritlecitinib vs. Tofacitinib 3 mg BID | Ritlecitinib vs. Placebo vs. Tofacitinib 3 mg BID | Low ⨁⨁◯◯ | Low ⨁⨁◯◯ | Low ⨁⨁◯◯ | No downgrade | Low ⨁⨁◯◯ |
| Ritlecitinib vs. Tofacitinib 5 mg BID | Ritlecitinib vs. Placebo vs. Tofacitinib 5 mg BID | Low ⨁⨁◯◯ | Low ⨁⨁◯◯ | Low ⨁⨁◯◯ | Downgrade | Very low ⨁◯◯◯ |
| Ritlecitinib vs. Upadacitinib 15 mg QD | Ritlecitinib vs. Placebo vs. Upadacitinib 15 mg QD | Low ⨁⨁◯◯ | Low ⨁⨁◯◯ | Low ⨁⨁◯◯ | Downgrade | Very low ⨁◯◯◯ |
| Ritlecitinib vs. Upadacitinib 30 mg QD | Ritlecitinib vs. Placebo vs. Upadacitinib 30 mg QD | Low ⨁⨁◯◯ | Low ⨁⨁◯◯ | Low ⨁⨁◯◯ | Downgrade | Very low ⨁◯◯◯ |
| Ritlecitinib vs. Upadacitinib 45 mg QD | Ritlecitinib vs. Placebo vs. Upadacitinib 45 mg QD | Low ⨁⨁◯◯ | Low ⨁⨁◯◯ | Low ⨁⨁◯◯ | Downgrade | Very low ⨁◯◯◯ |
| Tofacitinib 0.5 mg BID vs. Tofacitinib 1 mg BID | Tofacitinib 0.5 mg BID vs. Placebo vs. Tofacitinib 1 mg BID | Low ⨁⨁◯◯ | Very low ⨁◯◯◯ | Very low ⨁◯◯◯ | Downgrade | Very low ⨁◯◯◯ |
| Tofacitinib 0.5 mg BID vs. Tofacitinib 10 mg BID | Tofacitinib 0.5 mg BID vs. Placebo vs. Tofacitinib 10 mg BID | Low ⨁⨁◯◯ | Low ⨁⨁◯◯ | Low ⨁⨁◯◯ | Downgrade | Very low ⨁◯◯◯ |
| Tofacitinib 0.5 mg BID vs. Tofacitinib 15 mg BID | Tofacitinib 0.5 mg BID vs. Placebo vs. Tofacitinib 15 mg BID | Low ⨁⨁◯◯ | Low ⨁⨁◯◯ | Low ⨁⨁◯◯ | Downgrade | Very low ⨁◯◯◯ |
| Tofacitinib 0.5 mg BID vs. Tofacitinib 5 mg BID | Tofacitinib 0.5 mg BID vs. Placebo vs. Tofacitinib 5 mg BID | Low ⨁⨁◯◯ | Low ⨁⨁◯◯ | Low ⨁⨁◯◯ | Downgrade | Very low ⨁◯◯◯ |
| Tofacitinib 0.5 mg BID vs. Upadacitinib 15 mg QD | Tofacitinib 0.5 mg BID vs. Placebo vs. Upadacitinib 15 mg QD | Low ⨁⨁◯◯ | Low ⨁⨁◯◯ | Low ⨁⨁◯◯ | Downgrade | Very low ⨁◯◯◯ |
| Tofacitinib 0.5 mg BID vs. Upadacitinib 30 mg QD | Tofacitinib 0.5 mg BID vs. Placebo vs. Upadacitinib 30 mg QD | Low ⨁⨁◯◯ | Low ⨁⨁◯◯ | Low ⨁⨁◯◯ | Downgrade | Very low ⨁◯◯◯ |
| Tofacitinib 0.5 mg BID vs. Upadacitinib 45 mg QD | Tofacitinib 0.5 mg BID vs. Placebo vs. Upadacitinib 45 mg QD | Low ⨁⨁◯◯ | Low ⨁⨁◯◯ | Low ⨁⨁◯◯ | Downgrade | Very low ⨁◯◯◯ |
| Tofacitinib 1 mg BID vs. Tofacitinib 10 mg BID | Tofacitinib 1 mg BID vs. Placebo vs. Tofacitinib 10 mg BID | Very low ⨁◯◯◯ | Low ⨁⨁◯◯ | Very low ⨁◯◯◯ | Downgrade | Very low ⨁◯◯◯ |
| Tofacitinib 1 mg BID vs. Tofacitinib 15 mg BID | Tofacitinib 1 mg BID vs. Placebo vs. Tofacitinib 15 mg BID | Very low ⨁◯◯◯ | Low ⨁⨁◯◯ | Very low ⨁◯◯◯ | Downgrade | Very low ⨁◯◯◯ |
| Tofacitinib 1 mg BID vs. Tofacitinib 3 mg BID | Tofacitinib 1 mg BID vs. Placebo vs. Tofacitinib 3 mg BID | Very low ⨁◯◯◯ | Low ⨁⨁◯◯ | Very low ⨁◯◯◯ | Downgrade | Very low ⨁◯◯◯ |
| Tofacitinib 1 mg BID vs. Tofacitinib 5 mg BID | Tofacitinib 1 mg BID vs. Placebo vs. Tofacitinib 5 mg BID | Very low ⨁◯◯◯ | Low ⨁⨁◯◯ | Very low ⨁◯◯◯ | Downgrade | Very low ⨁◯◯◯ |
| Tofacitinib 1 mg BID vs. Upadacitinib 15 mg QD | Tofacitinib 1 mg BID vs. Placebo vs. Upadacitinib 15 mg QD | Very low ⨁◯◯◯ | Low ⨁⨁◯◯ | Very low ⨁◯◯◯ | Downgrade | Very low ⨁◯◯◯ |
| Tofacitinib 1 mg BID vs. Upadacitinib 30 mg QD | Tofacitinib 1 mg BID vs. Placebo vs. Upadacitinib 30 mg QD | Very low ⨁◯◯◯ | Low ⨁⨁◯◯ | Very low ⨁◯◯◯ | Downgrade | Very low ⨁◯◯◯ |
| Tofacitinib 1 mg BID vs. Upadacitinib 45 mg QD | Tofacitinib 1 mg BID vs. Placebo vs. Upadacitinib 45 mg QD | Very low ⨁◯◯◯ | Low ⨁⨁◯◯ | Very low ⨁◯◯◯ | Downgrade | Very low ⨁◯◯◯ |
| Tofacitinib 10 mg BID vs. Tofacitinib 15 mg BID | Tofacitinib 10 mg BID vs. Placebo vs. Tofacitinib 15 mg BID | Low ⨁⨁◯◯ | Low ⨁⨁◯◯ | Low ⨁⨁◯◯ | Downgrade | Very low ⨁◯◯◯ |
| Tofacitinib 10 mg BID vs. Tofacitinib 3 mg BID | Tofacitinib 10 mg BID vs. Placebo vs. Tofacitinib 3 mg BID | Low ⨁⨁◯◯ | Low ⨁⨁◯◯ | Low ⨁⨁◯◯ | Downgrade | Very low ⨁◯◯◯ |
| Tofacitinib 10 mg BID vs. Tofacitinib 5 mg BID | Tofacitinib 10 mg BID vs. Placebo vs. Tofacitinib 5 mg BID | Low ⨁⨁◯◯ | Low ⨁⨁◯◯ | Low ⨁⨁◯◯ | Downgrade | Very low ⨁◯◯◯ |
| Tofacitinib 10 mg BID vs. Upadacitinib 15 mg QD | Tofacitinib 10 mg BID vs. Placebo vs. Upadacitinib 15 mg QD | Low ⨁⨁◯◯ | Low ⨁⨁◯◯ | Low ⨁⨁◯◯ | Downgrade | Very low ⨁◯◯◯ |
| Tofacitinib 10 mg BID vs. Upadacitinib 30 mg QD | Tofacitinib 10 mg BID vs. Placebo vs. Upadacitinib 30 mg QD | Low ⨁⨁◯◯ | Low ⨁⨁◯◯ | Low ⨁⨁◯◯ | Downgrade | Very low ⨁◯◯◯ |
| Tofacitinib 10 mg BID vs. Upadacitinib 45 mg QD | Tofacitinib 10 mg BID vs. Placebo vs. Upadacitinib 45 mg QD | Low ⨁⨁◯◯ | Low ⨁⨁◯◯ | Low ⨁⨁◯◯ | Downgrade | Very low ⨁◯◯◯ |
| Tofacitinib 15 mg BID vs. Tofacitinib 3 mg BID | Tofacitinib 15 mg BID vs. Placebo vs. Tofacitinib 3 mg BID | Low ⨁⨁◯◯ | Low ⨁⨁◯◯ | Low ⨁⨁◯◯ | Downgrade | Very low ⨁◯◯◯ |
| Tofacitinib 15 mg BID vs. Tofacitinib 5 mg BID | Tofacitinib 15 mg BID vs. Placebo vs. Tofacitinib 5 mg BID | Low ⨁⨁◯◯ | Low ⨁⨁◯◯ | Low ⨁⨁◯◯ | Downgrade | Very low ⨁◯◯◯ |
| Tofacitinib 15 mg BID vs. Upadacitinib 15 mg QD | Tofacitinib 15 mg BID vs. Placebo vs. Upadacitinib 15 mg QD | Low ⨁⨁◯◯ | Low ⨁⨁◯◯ | Low ⨁⨁◯◯ | Downgrade | Very low ⨁◯◯◯ |
| Tofacitinib 15 mg BID vs. Upadacitinib 30 mg QD | Tofacitinib 15 mg BID vs. Placebo vs. Upadacitinib 30 mg QD | Low ⨁⨁◯◯ | Low ⨁⨁◯◯ | Low ⨁⨁◯◯ | Downgrade | Very low ⨁◯◯◯ |
| Tofacitinib 15 mg BID vs. Upadacitinib 45 mg QD | Tofacitinib 15 mg BID vs. Placebo vs. Upadacitinib 45 mg QD | Low ⨁⨁◯◯ | Low ⨁⨁◯◯ | Low ⨁⨁◯◯ | No downgrade | Low ⨁⨁◯◯ |
| Tofacitinib 3 mg BID vs. Tofacitinib 5 mg BID | Tofacitinib 3 mg BID vs. Placebo vs. Tofacitinib 5 mg BID | Low ⨁⨁◯◯ | Low ⨁⨁◯◯ | Low ⨁⨁◯◯ | Downgrade | Very low ⨁◯◯◯ |
| Tofacitinib 3 mg BID vs. Upadacitinib 15 mg QD | Tofacitinib 3 mg BID vs. Placebo vs. Upadacitinib 15 mg QD | Low ⨁⨁◯◯ | Low ⨁⨁◯◯ | Low ⨁⨁◯◯ | Downgrade | Very low ⨁◯◯◯ |
| Tofacitinib 3 mg BID vs. Upadacitinib 30 mg QD | Tofacitinib 3 mg BID vs. Placebo vs. Upadacitinib 30 mg QD | Low ⨁⨁◯◯ | Low ⨁⨁◯◯ | Low ⨁⨁◯◯ | Downgrade | Very low ⨁◯◯◯ |
| Tofacitinib 3 mg BID vs. Upadacitinib 45 mg QD | Tofacitinib 3 mg BID vs. Placebo vs. Upadacitinib 45 mg QD | Low ⨁⨁◯◯ | Low ⨁⨁◯◯ | Low ⨁⨁◯◯ | Downgrade | Very low ⨁◯◯◯ |
| Tofacitinib 5 mg BID vs. Upadacitinib 15 mg QD | Tofacitinib 5 mg BID vs. Placebo vs. Upadacitinib 15 mg QD | Low ⨁⨁◯◯ | Low ⨁⨁◯◯ | Low ⨁⨁◯◯ | Downgrade | Very low ⨁◯◯◯ |
| Tofacitinib 5 mg BID vs. Upadacitinib 30 mg QD | Tofacitinib 5 mg BID vs. Placebo vs. Upadacitinib 30 mg QD | Low ⨁⨁◯◯ | Low ⨁⨁◯◯ | Low ⨁⨁◯◯ | Downgrade | Very low ⨁◯◯◯ |
| Tofacitinib 5 mg BID vs. Upadacitinib 45 mg QD | Tofacitinib 5 mg BID vs. Placebo vs. Upadacitinib 45 mg QD | Low ⨁⨁◯◯ | Low ⨁⨁◯◯ | Low ⨁⨁◯◯ | Downgrade | Very low ⨁◯◯◯ |
| Upadacitinib 15 mg QD vs. Upadacitinib 30 mg QD | Upadacitinib 15 mg QD vs. Placebo vs. Upadacitinib 30 mg QD | Low ⨁⨁◯◯ | Low ⨁⨁◯◯ | Low ⨁⨁◯◯ | Downgrade | Very low ⨁◯◯◯ |
| Upadacitinib 15 mg QD vs. Upadacitinib 45 mg QD | Upadacitinib 15 mg QD vs. Placebo vs. Upadacitinib 45 mg QD | Low ⨁⨁◯◯ | Low ⨁⨁◯◯ | Low ⨁⨁◯◯ | Downgrade | Very low ⨁◯◯◯ |
| Upadacitinib 30 mg QD vs. Upadacitinib 45 mg QD | Upadacitinib 30 mg QD vs. Placebo vs. Upadacitinib 45 mg QD | Low ⨁⨁◯◯ | Low ⨁⨁◯◯ | Low ⨁⨁◯◯ | Downgrade | Very low ⨁◯◯◯ |

VTE: venous thromboembolism events; QD: once daily; BID: twice daily.

**Supplementary table 10-2-3 Certainty of indirect evidence assessment for CVE (with dose consideration).**

| Comparison | First order loop | Start rating | | | Intransitivity | Preliminary certainty rating |
| --- | --- | --- | --- | --- | --- | --- |
|  |  | A→B Direct preliminary certainty rating | B→C Direct Preliminary certainty rating | Lowest certainty of the first loop components |  |  |
| Deucravacitinib vs. Brepocitinib | Deucravacitinib vs. Placebo vs. Brepocitinib | Low ⨁⨁◯◯ | Moderate ⨁⨁⨁◯ | Low ⨁⨁◯◯ | No downgrade | Low ⨁⨁◯◯ |
| Filgotinib 100 mg QD vs. Brepocitinib | Filgotinib 100 mg QD vs. Placebo vs. Brepocitinib | Moderate ⨁⨁⨁◯ | Moderate ⨁⨁⨁◯ | Moderate ⨁⨁⨁◯ | Downgrade | Low ⨁⨁◯◯ |
| Filgotinib 200 mg QD vs. Brepocitinib | Filgotinib 200 mg QD vs. Placebo vs. Brepocitinib | Moderate ⨁⨁⨁◯ | Moderate ⨁⨁⨁◯ | Moderate ⨁⨁⨁◯ | Downgrade | Low ⨁⨁◯◯ |
| Ivarmacitinib vs. Brepocitinib | Ivarmacitinib vs. Placebo vs. Brepocitinib | Moderate ⨁⨁⨁◯ | Moderate ⨁⨁⨁◯ | Moderate ⨁⨁⨁◯ | No downgrade | Moderate ⨁⨁⨁◯ |
| Izencitinib vs. Brepocitinib | Izencitinib vs. Placebo vs. Brepocitinib | Moderate ⨁⨁⨁◯ | Moderate ⨁⨁⨁◯ | Moderate ⨁⨁⨁◯ | Downgrade | Low ⨁⨁◯◯ |
| Peficitinib vs. Brepocitinib | Peficitinib vs. Placebo vs. Brepocitinib | Moderate ⨁⨁⨁◯ | Moderate ⨁⨁⨁◯ | Moderate ⨁⨁⨁◯ | No downgrade | Moderate ⨁⨁⨁◯ |
| Tofacitinib 0.5 mg BID vs. Brepocitinib | Tofacitinib 0.5 mg BID vs. Placebo vs. Brepocitinib | Moderate ⨁⨁⨁◯ | Moderate ⨁⨁⨁◯ | Moderate ⨁⨁⨁◯ | No downgrade | Moderate ⨁⨁⨁◯ |
| Tofacitinib 1 mg BID vs. Brepocitinib | Tofacitinib 1 mg BID vs. Placebo vs. Brepocitinib | Low ⨁⨁◯◯ | Moderate ⨁⨁⨁◯ | Low ⨁⨁◯◯ | Downgrade | Very low ⨁◯◯◯ |
| Tofacitinib 10 mg BID vs. Brepocitinib | Tofacitinib 10 mg BID vs. Placebo vs. Brepocitinib | Moderate ⨁⨁⨁◯ | Moderate ⨁⨁⨁◯ | Moderate ⨁⨁⨁◯ | Downgrade | Low ⨁⨁◯◯ |
| Tofacitinib 15 mg BID vs. Brepocitinib | Tofacitinib 15 mg BID vs. Placebo vs. Brepocitinib | Moderate ⨁⨁⨁◯ | Moderate ⨁⨁⨁◯ | Moderate ⨁⨁⨁◯ | Downgrade | Low ⨁⨁◯◯ |
| Tofacitinib 3 mg BID vs. Brepocitinib | Tofacitinib 3 mg BID vs. Placebo vs. Brepocitinib | Moderate ⨁⨁⨁◯ | Moderate ⨁⨁⨁◯ | Moderate ⨁⨁⨁◯ | No downgrade | Moderate ⨁⨁⨁◯ |
| Tofacitinib 5 mg BID vs. Brepocitinib | Tofacitinib 5 mg BID vs. Placebo vs. Brepocitinib | Moderate ⨁⨁⨁◯ | Moderate ⨁⨁⨁◯ | Moderate ⨁⨁⨁◯ | Downgrade | Low ⨁⨁◯◯ |
| Upadacitinib 15 mg QD vs. Brepocitinib | Upadacitinib 15 mg QD vs. Placebo vs. Brepocitinib | Moderate ⨁⨁⨁◯ | Moderate ⨁⨁⨁◯ | Moderate ⨁⨁⨁◯ | Downgrade | Low ⨁⨁◯◯ |
| Upadacitinib 30 mg QD vs. Brepocitinib | Upadacitinib 30 mg QD vs. Placebo vs. Brepocitinib | Moderate ⨁⨁⨁◯ | Moderate ⨁⨁⨁◯ | Moderate ⨁⨁⨁◯ | Downgrade | Low ⨁⨁◯◯ |
| Upadacitinib 45 mg QD vs. Brepocitinib | Upadacitinib 45 mg QD vs. Placebo vs. Brepocitinib | Moderate ⨁⨁⨁◯ | Moderate ⨁⨁⨁◯ | Moderate ⨁⨁⨁◯ | Downgrade | Low ⨁⨁◯◯ |
| Deucravacitinib vs. Filgotinib 100 mg QD | Deucravacitinib vs. Placebo vs. Filgotinib 100 mg QD | Low ⨁⨁◯◯ | Moderate ⨁⨁⨁◯ | Low ⨁⨁◯◯ | Downgrade | Very low ⨁◯◯◯ |
| Deucravacitinib vs. Filgotinib 200 mg QD | Deucravacitinib vs. Placebo vs. Filgotinib 200 mg QD | Low ⨁⨁◯◯ | Moderate ⨁⨁⨁◯ | Low ⨁⨁◯◯ | Downgrade | Very low ⨁◯◯◯ |
| Deucravacitinib vs. Ivarmacitinib | Deucravacitinib vs. Placebo vs. Ivarmacitinib | Low ⨁⨁◯◯ | Moderate ⨁⨁⨁◯ | Low ⨁⨁◯◯ | No downgrade | Low ⨁⨁◯◯ |
| Deucravacitinib vs. Izencitinib | Deucravacitinib vs. Placebo vs. Izencitinib | Low ⨁⨁◯◯ | Moderate ⨁⨁⨁◯ | Low ⨁⨁◯◯ | Downgrade | Very low ⨁◯◯◯ |
| Deucravacitinib vs. Peficitinib | Deucravacitinib vs. Placebo vs. Peficitinib | Low ⨁⨁◯◯ | Moderate ⨁⨁⨁◯ | Low ⨁⨁◯◯ | No downgrade | Low ⨁⨁◯◯ |
| Deucravacitinib vs. Ritlecitinib | Deucravacitinib vs. Placebo vs. Ritlecitinib | Low ⨁⨁◯◯ | Moderate ⨁⨁⨁◯ | Low ⨁⨁◯◯ | No downgrade | Low ⨁⨁◯◯ |
| Deucravacitinib vs. Tofacitinib 0.5 mg BID | Deucravacitinib vs. Placebo vs. Tofacitinib 0.5 mg BID | Low ⨁⨁◯◯ | Moderate ⨁⨁⨁◯ | Low ⨁⨁◯◯ | No downgrade | Low ⨁⨁◯◯ |
| Deucravacitinib vs. Tofacitinib 1 mg BID | Deucravacitinib vs. Placebo vs. Tofacitinib 1 mg BID | Low ⨁⨁◯◯ | Low ⨁⨁◯◯ | Low ⨁⨁◯◯ | Downgrade | Very low ⨁◯◯◯ |
| Deucravacitinib vs. Tofacitinib 10 mg BID | Deucravacitinib vs. Placebo vs. Tofacitinib 10 mg BID | Low ⨁⨁◯◯ | Moderate ⨁⨁⨁◯ | Low ⨁⨁◯◯ | Downgrade | Very low ⨁◯◯◯ |
| Deucravacitinib vs. Tofacitinib 15 mg BID | Deucravacitinib vs. Placebo vs. Tofacitinib 15 mg BID | Low ⨁⨁◯◯ | Moderate ⨁⨁⨁◯ | Low ⨁⨁◯◯ | Downgrade | Very low ⨁◯◯◯ |
| Deucravacitinib vs. Tofacitinib 3 mg BID | Deucravacitinib vs. Placebo vs. Tofacitinib 3 mg BID | Low ⨁⨁◯◯ | Moderate ⨁⨁⨁◯ | Low ⨁⨁◯◯ | No downgrade | Low ⨁⨁◯◯ |
| Deucravacitinib vs. Tofacitinib 5 mg BID | Deucravacitinib vs. Placebo vs. Tofacitinib 5 mg BID | Low ⨁⨁◯◯ | Moderate ⨁⨁⨁◯ | Low ⨁⨁◯◯ | Downgrade | Very low ⨁◯◯◯ |
| Deucravacitinib vs. Upadacitinib 15 mg QD | Deucravacitinib vs. Placebo vs. Upadacitinib 15 mg QD | Low ⨁⨁◯◯ | Moderate ⨁⨁⨁◯ | Low ⨁⨁◯◯ | Downgrade | Very low ⨁◯◯◯ |
| Deucravacitinib vs. Upadacitinib 30 mg QD | Deucravacitinib vs. Placebo vs. Upadacitinib 30 mg QD | Low ⨁⨁◯◯ | Moderate ⨁⨁⨁◯ | Low ⨁⨁◯◯ | Downgrade | Very low ⨁◯◯◯ |
| Deucravacitinib vs. Upadacitinib 45 mg QD | Deucravacitinib vs. Placebo vs. Upadacitinib 45 mg QD | Low ⨁⨁◯◯ | Moderate ⨁⨁⨁◯ | Low ⨁⨁◯◯ | Downgrade | Very low ⨁◯◯◯ |
| Filgotinib 100 mg QD vs. Filgotinib 200 mg QD | Filgotinib 100 mg QD vs. Placebo vs. Filgotinib 200 mg QD | Moderate ⨁⨁⨁◯ | Moderate ⨁⨁⨁◯ | Moderate ⨁⨁⨁◯ | No downgrade | Moderate ⨁⨁⨁◯ |
| Filgotinib 100 mg QD vs. Ivarmacitinib | Filgotinib 100 mg QD vs. Placebo vs. Ivarmacitinib | Moderate ⨁⨁⨁◯ | Moderate ⨁⨁⨁◯ | Moderate ⨁⨁⨁◯ | Downgrade | Low ⨁⨁◯◯ |
| Filgotinib 100 mg QD vs. Izencitinib | Filgotinib 100 mg QD vs. Placebo vs. Izencitinib | Moderate ⨁⨁⨁◯ | Moderate ⨁⨁⨁◯ | Moderate ⨁⨁⨁◯ | Downgrade | Low ⨁⨁◯◯ |
| Filgotinib 100 mg QD vs. Peficitinib | Filgotinib 100 mg QD vs. Placebo vs. Peficitinib | Moderate ⨁⨁⨁◯ | Moderate ⨁⨁⨁◯ | Moderate ⨁⨁⨁◯ | Downgrade | Low ⨁⨁◯◯ |
| Filgotinib 100 mg QD vs. Placebo | Filgotinib 100 mg QD vs. Filgotinib 200 mg QD vs. Placebo | Moderate ⨁⨁⨁◯ | Moderate ⨁⨁⨁◯ | Moderate ⨁⨁⨁◯ | Downgrade | Low ⨁⨁◯◯ |
| Filgotinib 100 mg QD vs. Ritlecitinib | Filgotinib 100 mg QD vs. Placebo vs. Ritlecitinib | Moderate ⨁⨁⨁◯ | Moderate ⨁⨁⨁◯ | Moderate ⨁⨁⨁◯ | Downgrade | Low ⨁⨁◯◯ |
| Filgotinib 100 mg QD vs. Tofacitinib 0.5 mg BID | Filgotinib 100 mg QD vs. Placebo vs. Tofacitinib 0.5 mg BID | Moderate ⨁⨁⨁◯ | Moderate ⨁⨁⨁◯ | Moderate ⨁⨁⨁◯ | Downgrade | Low ⨁⨁◯◯ |
| Filgotinib 100 mg QD vs. Tofacitinib 1 mg BID | Filgotinib 100 mg QD vs. Placebo vs. Tofacitinib 1 mg BID | Moderate ⨁⨁⨁◯ | Low ⨁⨁◯◯ | Low ⨁⨁◯◯ | Downgrade | Very low ⨁◯◯◯ |
| Filgotinib 100 mg QD vs. Tofacitinib 10 mg BID | Filgotinib 100 mg QD vs. Placebo vs. Tofacitinib 10 mg BID | Moderate ⨁⨁⨁◯ | Moderate ⨁⨁⨁◯ | Moderate ⨁⨁⨁◯ | Downgrade | Low ⨁⨁◯◯ |
| Filgotinib 100 mg QD vs. Tofacitinib 15 mg BID | Filgotinib 100 mg QD vs. Placebo vs. Tofacitinib 15 mg BID | Moderate ⨁⨁⨁◯ | Moderate ⨁⨁⨁◯ | Moderate ⨁⨁⨁◯ | Downgrade | Low ⨁⨁◯◯ |
| Filgotinib 100 mg QD vs. Tofacitinib 3 mg BID | Filgotinib 100 mg QD vs. Placebo vs. Tofacitinib 3 mg BID | Moderate ⨁⨁⨁◯ | Moderate ⨁⨁⨁◯ | Moderate ⨁⨁⨁◯ | Downgrade | Low ⨁⨁◯◯ |
| Filgotinib 100 mg QD vs. Tofacitinib 5 mg BID | Filgotinib 100 mg QD vs. Placebo vs. Tofacitinib 5 mg BID | Moderate ⨁⨁⨁◯ | Moderate ⨁⨁⨁◯ | Moderate ⨁⨁⨁◯ | Downgrade | Low ⨁⨁◯◯ |
| Filgotinib 100 mg QD vs. Upadacitinib 15 mg QD | Filgotinib 100 mg QD vs. Placebo vs. Upadacitinib 15 mg QD | Moderate ⨁⨁⨁◯ | Moderate ⨁⨁⨁◯ | Moderate ⨁⨁⨁◯ | Downgrade | Low ⨁⨁◯◯ |
| Filgotinib 100 mg QD vs. Upadacitinib 30 mg QD | Filgotinib 100 mg QD vs. Placebo vs. Upadacitinib 30 mg QD | Moderate ⨁⨁⨁◯ | Moderate ⨁⨁⨁◯ | Moderate ⨁⨁⨁◯ | Downgrade | Low ⨁⨁◯◯ |
| Filgotinib 100 mg QD vs. Upadacitinib 45 mg QD | Filgotinib 100 mg QD vs. Placebo vs. Upadacitinib 45 mg QD | Moderate ⨁⨁⨁◯ | Moderate ⨁⨁⨁◯ | Moderate ⨁⨁⨁◯ | Downgrade | Low ⨁⨁◯◯ |
| Filgotinib 200 mg QD vs. Ivarmacitinib | Filgotinib 200 mg QD vs. Placebo vs. Ivarmacitinib | Moderate ⨁⨁⨁◯ | Moderate ⨁⨁⨁◯ | Moderate ⨁⨁⨁◯ | Downgrade | Low ⨁⨁◯◯ |
| Filgotinib 200 mg QD vs. Izencitinib | Filgotinib 200 mg QD vs. Placebo vs. Izencitinib | Moderate ⨁⨁⨁◯ | Moderate ⨁⨁⨁◯ | Moderate ⨁⨁⨁◯ | Downgrade | Low ⨁⨁◯◯ |
| Filgotinib 200 mg QD vs. Peficitinib | Filgotinib 200 mg QD vs. Placebo vs. Peficitinib | Moderate ⨁⨁⨁◯ | Moderate ⨁⨁⨁◯ | Moderate ⨁⨁⨁◯ | Downgrade | Low ⨁⨁◯◯ |
| Filgotinib 200 mg QD vs. Placebo | Filgotinib 200 mg QD vs. Filgotinib 100 mg QD vs. Placebo | Moderate ⨁⨁⨁◯ | Moderate ⨁⨁⨁◯ | Moderate ⨁⨁⨁◯ | Downgrade | Low ⨁⨁◯◯ |
| Filgotinib 200 mg QD vs. Ritlecitinib | Filgotinib 200 mg QD vs. Placebo vs. Ritlecitinib | Moderate ⨁⨁⨁◯ | Moderate ⨁⨁⨁◯ | Moderate ⨁⨁⨁◯ | Downgrade | Low ⨁⨁◯◯ |
| Filgotinib 200 mg QD vs. Tofacitinib 0.5 mg BID | Filgotinib 200 mg QD vs. Placebo vs. Tofacitinib 0.5 mg BID | Moderate ⨁⨁⨁◯ | Moderate ⨁⨁⨁◯ | Moderate ⨁⨁⨁◯ | Downgrade | Low ⨁⨁◯◯ |
| Filgotinib 200 mg QD vs. Tofacitinib 1 mg BID | Filgotinib 200 mg QD vs. Placebo vs. Tofacitinib 1 mg BID | Moderate ⨁⨁⨁◯ | Low ⨁⨁◯◯ | Low ⨁⨁◯◯ | Downgrade | Very low ⨁◯◯◯ |
| Filgotinib 200 mg QD vs. Tofacitinib 10 mg BID | Filgotinib 200 mg QD vs. Placebo vs. Tofacitinib 10 mg BID | Moderate ⨁⨁⨁◯ | Moderate ⨁⨁⨁◯ | Moderate ⨁⨁⨁◯ | Downgrade | Low ⨁⨁◯◯ |
| Filgotinib 200 mg QD vs. Tofacitinib 15 mg BID | Filgotinib 200 mg QD vs. Placebo vs. Tofacitinib 15 mg BID | Moderate ⨁⨁⨁◯ | Moderate ⨁⨁⨁◯ | Moderate ⨁⨁⨁◯ | Downgrade | Low ⨁⨁◯◯ |
| Filgotinib 200 mg QD vs. Tofacitinib 3 mg BID | Filgotinib 200 mg QD vs. Placebo vs. Tofacitinib 3 mg BID | Moderate ⨁⨁⨁◯ | Moderate ⨁⨁⨁◯ | Moderate ⨁⨁⨁◯ | Downgrade | Low ⨁⨁◯◯ |
| Filgotinib 200 mg QD vs. Tofacitinib 5 mg BID | Filgotinib 200 mg QD vs. Placebo vs. Tofacitinib 5 mg BID | Moderate ⨁⨁⨁◯ | Moderate ⨁⨁⨁◯ | Moderate ⨁⨁⨁◯ | Downgrade | Low ⨁⨁◯◯ |
| Filgotinib 200 mg QD vs. Upadacitinib 15 mg QD | Filgotinib 200 mg QD vs. Placebo vs. Upadacitinib 15 mg QD | Moderate ⨁⨁⨁◯ | Moderate ⨁⨁⨁◯ | Moderate ⨁⨁⨁◯ | Downgrade | Low ⨁⨁◯◯ |
| Filgotinib 200 mg QD vs. Upadacitinib 30 mg QD | Filgotinib 200 mg QD vs. Placebo vs. Upadacitinib 30 mg QD | Moderate ⨁⨁⨁◯ | Moderate ⨁⨁⨁◯ | Moderate ⨁⨁⨁◯ | Downgrade | Low ⨁⨁◯◯ |
| Filgotinib 200 mg QD vs. Upadacitinib 45 mg QD | Filgotinib 200 mg QD vs. Placebo vs. Upadacitinib 45 mg QD | Moderate ⨁⨁⨁◯ | Moderate ⨁⨁⨁◯ | Moderate ⨁⨁⨁◯ | Downgrade | Low ⨁⨁◯◯ |
| Ivarmacitinib vs. Izencitinib | Ivarmacitinib vs. Placebo vs. Izencitinib | Moderate ⨁⨁⨁◯ | Moderate ⨁⨁⨁◯ | Moderate ⨁⨁⨁◯ | Downgrade | Low ⨁⨁◯◯ |
| Ivarmacitinib vs. Peficitinib | Ivarmacitinib vs. Placebo vs. Peficitinib | Moderate ⨁⨁⨁◯ | Moderate ⨁⨁⨁◯ | Moderate ⨁⨁⨁◯ | No downgrade | Moderate ⨁⨁⨁◯ |
| Ivarmacitinib vs. Ritlecitinib | Ivarmacitinib vs. Placebo vs. Ritlecitinib | Moderate ⨁⨁⨁◯ | Moderate ⨁⨁⨁◯ | Moderate ⨁⨁⨁◯ | No downgrade | Moderate ⨁⨁⨁◯ |
| Ivarmacitinib vs. Tofacitinib 0.5 mg BID | Ivarmacitinib vs. Placebo vs. Tofacitinib 0.5 mg BID | Moderate ⨁⨁⨁◯ | Moderate ⨁⨁⨁◯ | Moderate ⨁⨁⨁◯ | No downgrade | Moderate ⨁⨁⨁◯ |
| Ivarmacitinib vs. Tofacitinib 1 mg BID | Ivarmacitinib vs. Placebo vs. Tofacitinib 1 mg BID | Moderate ⨁⨁⨁◯ | Low ⨁⨁◯◯ | Low ⨁⨁◯◯ | Downgrade | Very low ⨁◯◯◯ |
| Ivarmacitinib vs. Tofacitinib 10 mg BID | Ivarmacitinib vs. Placebo vs. Tofacitinib 10 mg BID | Moderate ⨁⨁⨁◯ | Moderate ⨁⨁⨁◯ | Moderate ⨁⨁⨁◯ | Downgrade | Low ⨁⨁◯◯ |
| Ivarmacitinib vs. Tofacitinib 15 mg BID | Ivarmacitinib vs. Placebo vs. Tofacitinib 15 mg BID | Moderate ⨁⨁⨁◯ | Moderate ⨁⨁⨁◯ | Moderate ⨁⨁⨁◯ | Downgrade | Low ⨁⨁◯◯ |
| Ivarmacitinib vs. Tofacitinib 3 mg BID | Ivarmacitinib vs. Placebo vs. Tofacitinib 3 mg BID | Moderate ⨁⨁⨁◯ | Moderate ⨁⨁⨁◯ | Moderate ⨁⨁⨁◯ | No downgrade | Moderate ⨁⨁⨁◯ |
| Ivarmacitinib vs. Tofacitinib 5 mg BID | Ivarmacitinib vs. Placebo vs. Tofacitinib 5 mg BID | Moderate ⨁⨁⨁◯ | Moderate ⨁⨁⨁◯ | Moderate ⨁⨁⨁◯ | Downgrade | Low ⨁⨁◯◯ |
| Ivarmacitinib vs. Upadacitinib 15 mg QD | Ivarmacitinib vs. Placebo vs. Upadacitinib 15 mg QD | Moderate ⨁⨁⨁◯ | Moderate ⨁⨁⨁◯ | Moderate ⨁⨁⨁◯ | Downgrade | Low ⨁⨁◯◯ |
| Ivarmacitinib vs. Upadacitinib 30 mg QD | Ivarmacitinib vs. Placebo vs. Upadacitinib 30 mg QD | Moderate ⨁⨁⨁◯ | Moderate ⨁⨁⨁◯ | Moderate ⨁⨁⨁◯ | Downgrade | Low ⨁⨁◯◯ |
| Ivarmacitinib vs. Upadacitinib 45 mg QD | Ivarmacitinib vs. Placebo vs. Upadacitinib 45 mg QD | Moderate ⨁⨁⨁◯ | Moderate ⨁⨁⨁◯ | Moderate ⨁⨁⨁◯ | Downgrade | Low ⨁⨁◯◯ |
| Izencitinib vs. Peficitinib | Izencitinib vs. Placebo vs. Peficitinib | Moderate ⨁⨁⨁◯ | Moderate ⨁⨁⨁◯ | Moderate ⨁⨁⨁◯ | Downgrade | Low ⨁⨁◯◯ |
| Izencitinib vs. Ritlecitinib | Izencitinib vs. Placebo vs. Ritlecitinib | Moderate ⨁⨁⨁◯ | Moderate ⨁⨁⨁◯ | Moderate ⨁⨁⨁◯ | Downgrade | Low ⨁⨁◯◯ |
| Izencitinib vs. Tofacitinib 0.5 mg BID | Izencitinib vs. Placebo vs. Tofacitinib 0.5 mg BID | Moderate ⨁⨁⨁◯ | Moderate ⨁⨁⨁◯ | Moderate ⨁⨁⨁◯ | Downgrade | Low ⨁⨁◯◯ |
| Izencitinib vs. Tofacitinib 1 mg BID | Izencitinib vs. Placebo vs. Tofacitinib 1 mg BID | Moderate ⨁⨁⨁◯ | Low ⨁⨁◯◯ | Low ⨁⨁◯◯ | Downgrade | Very low ⨁◯◯◯ |
| Izencitinib vs. Tofacitinib 10 mg BID | Izencitinib vs. Placebo vs. Tofacitinib 10 mg BID | Moderate ⨁⨁⨁◯ | Moderate ⨁⨁⨁◯ | Moderate ⨁⨁⨁◯ | Downgrade | Low ⨁⨁◯◯ |
| Izencitinib vs. Tofacitinib 15 mg BID | Izencitinib vs. Placebo vs. Tofacitinib 15 mg BID | Moderate ⨁⨁⨁◯ | Moderate ⨁⨁⨁◯ | Moderate ⨁⨁⨁◯ | Downgrade | Low ⨁⨁◯◯ |
| Izencitinib vs. Tofacitinib 3 mg BID | Izencitinib vs. Placebo vs. Tofacitinib 3 mg BID | Moderate ⨁⨁⨁◯ | Moderate ⨁⨁⨁◯ | Moderate ⨁⨁⨁◯ | Downgrade | Low ⨁⨁◯◯ |
| Izencitinib vs. Tofacitinib 5 mg BID | Izencitinib vs. Placebo vs. Tofacitinib 5 mg BID | Moderate ⨁⨁⨁◯ | Moderate ⨁⨁⨁◯ | Moderate ⨁⨁⨁◯ | Downgrade | Low ⨁⨁◯◯ |
| Izencitinib vs. Upadacitinib 15 mg QD | Izencitinib vs. Placebo vs. Upadacitinib 15 mg QD | Moderate ⨁⨁⨁◯ | Moderate ⨁⨁⨁◯ | Moderate ⨁⨁⨁◯ | Downgrade | Low ⨁⨁◯◯ |
| Izencitinib vs. Upadacitinib 30 mg QD | Izencitinib vs. Placebo vs. Upadacitinib 30 mg QD | Moderate ⨁⨁⨁◯ | Moderate ⨁⨁⨁◯ | Moderate ⨁⨁⨁◯ | Downgrade | Low ⨁⨁◯◯ |
| Izencitinib vs. Upadacitinib 45 mg QD | Izencitinib vs. Placebo vs. Upadacitinib 45 mg QD | Moderate ⨁⨁⨁◯ | Moderate ⨁⨁⨁◯ | Moderate ⨁⨁⨁◯ | Downgrade | Low ⨁⨁◯◯ |
| Peficitinib vs. Ritlecitinib | Peficitinib vs. Placebo vs. Ritlecitinib | Moderate ⨁⨁⨁◯ | Moderate ⨁⨁⨁◯ | Moderate ⨁⨁⨁◯ | No downgrade | Moderate ⨁⨁⨁◯ |
| Peficitinib vs. Tofacitinib 0.5 mg BID | Peficitinib vs. Placebo vs. Tofacitinib 0.5 mg BID | Moderate ⨁⨁⨁◯ | Moderate ⨁⨁⨁◯ | Moderate ⨁⨁⨁◯ | No downgrade | Moderate ⨁⨁⨁◯ |
| Peficitinib vs. Tofacitinib 1 mg BID | Peficitinib vs. Placebo vs. Tofacitinib 1 mg BID | Moderate ⨁⨁⨁◯ | Low ⨁⨁◯◯ | Low ⨁⨁◯◯ | Downgrade | Very low ⨁◯◯◯ |
| Peficitinib vs. Tofacitinib 10 mg BID | Peficitinib vs. Placebo vs. Tofacitinib 10 mg BID | Moderate ⨁⨁⨁◯ | Moderate ⨁⨁⨁◯ | Moderate ⨁⨁⨁◯ | Downgrade | Low ⨁⨁◯◯ |
| Peficitinib vs. Tofacitinib 15 mg BID | Peficitinib vs. Placebo vs. Tofacitinib 15 mg BID | Moderate ⨁⨁⨁◯ | Moderate ⨁⨁⨁◯ | Moderate ⨁⨁⨁◯ | Downgrade | Low ⨁⨁◯◯ |
| Peficitinib vs. Tofacitinib 3 mg BID | Peficitinib vs. Placebo vs. Tofacitinib 3 mg BID | Moderate ⨁⨁⨁◯ | Moderate ⨁⨁⨁◯ | Moderate ⨁⨁⨁◯ | No downgrade | Moderate ⨁⨁⨁◯ |
| Peficitinib vs. Tofacitinib 5 mg BID | Peficitinib vs. Placebo vs. Tofacitinib 5 mg BID | Moderate ⨁⨁⨁◯ | Moderate ⨁⨁⨁◯ | Moderate ⨁⨁⨁◯ | Downgrade | Low ⨁⨁◯◯ |
| Peficitinib vs. Upadacitinib 15 mg QD | Peficitinib vs. Placebo vs. Upadacitinib 15 mg QD | Moderate ⨁⨁⨁◯ | Moderate ⨁⨁⨁◯ | Moderate ⨁⨁⨁◯ | Downgrade | Low ⨁⨁◯◯ |
| Peficitinib vs. Upadacitinib 30 mg QD | Peficitinib vs. Placebo vs. Upadacitinib 30 mg QD | Moderate ⨁⨁⨁◯ | Moderate ⨁⨁⨁◯ | Moderate ⨁⨁⨁◯ | Downgrade | Low ⨁⨁◯◯ |
| Peficitinib vs. Upadacitinib 45 mg QD | Peficitinib vs. Placebo vs. Upadacitinib 45 mg QD | Moderate ⨁⨁⨁◯ | Moderate ⨁⨁⨁◯ | Moderate ⨁⨁⨁◯ | Downgrade | Low ⨁⨁◯◯ |
| Placebo vs. Tofacitinib 0.5 mg BID | Placebo vs. Tofacitinib 10 mg BID vs. Tofacitinib 0.5 mg BID | Moderate ⨁⨁⨁◯ | Moderate ⨁⨁⨁◯ | Moderate ⨁⨁⨁◯ | Downgrade | Low ⨁⨁◯◯ |
| Placebo vs. Tofacitinib 1 mg BID | Placebo vs. Tofacitinib 5 mg BID vs. Tofacitinib 1 mg BID | Moderate ⨁⨁⨁◯ | Low ⨁⨁◯◯ | Low ⨁⨁◯◯ | Downgrade | Very low ⨁◯◯◯ |
| Placebo vs. Tofacitinib 10 mg BID | Placebo vs. Tofacitinib 5 mg BID vs. Tofacitinib 10 mg BID | Moderate ⨁⨁⨁◯ | Moderate ⨁⨁⨁◯ | Moderate ⨁⨁⨁◯ | Downgrade | Low ⨁⨁◯◯ |
| Placebo vs. Tofacitinib 15 mg BID | Placebo vs. Tofacitinib 10 mg BID vs. Tofacitinib 15 mg BID | Moderate ⨁⨁⨁◯ | Moderate ⨁⨁⨁◯ | Moderate ⨁⨁⨁◯ | Downgrade | Low ⨁⨁◯◯ |
| Placebo vs. Tofacitinib 3 mg BID | Placebo vs. Tofacitinib 10 mg BID vs. Tofacitinib 3 mg BID | Moderate ⨁⨁⨁◯ | Moderate ⨁⨁⨁◯ | Moderate ⨁⨁⨁◯ | Downgrade | Low ⨁⨁◯◯ |
| Placebo vs. Tofacitinib 5 mg BID | Placebo vs. Tofacitinib 10 mg BID vs. Tofacitinib 5 mg BID | Moderate ⨁⨁⨁◯ | Moderate ⨁⨁⨁◯ | Moderate ⨁⨁⨁◯ | Downgrade | Low ⨁⨁◯◯ |
| Placebo vs. Upadacitinib 15 mg QD | Placebo vs. Upadacitinib 45 mg QD vs. Upadacitinib 15 mg QD | Moderate ⨁⨁⨁◯ | Moderate ⨁⨁⨁◯ | Moderate ⨁⨁⨁◯ | Downgrade | Low ⨁⨁◯◯ |
| Placebo vs. Upadacitinib 30 mg QD | Placebo vs. Upadacitinib 45 mg QD vs. Upadacitinib 30 mg QD | Moderate ⨁⨁⨁◯ | Moderate ⨁⨁⨁◯ | Moderate ⨁⨁⨁◯ | Downgrade | Low ⨁⨁◯◯ |
| Placebo vs. Upadacitinib 45 mg QD | Placebo vs. Upadacitinib 15 mg QD vs. Upadacitinib 45 mg QD | Moderate ⨁⨁⨁◯ | Moderate ⨁⨁⨁◯ | Moderate ⨁⨁⨁◯ | Downgrade | Low ⨁⨁◯◯ |
| Ritlecitinib vs. Tofacitinib 0.5 mg BID | Ritlecitinib vs. Placebo vs. Tofacitinib 0.5 mg BID | Moderate ⨁⨁⨁◯ | Moderate ⨁⨁⨁◯ | Moderate ⨁⨁⨁◯ | No downgrade | Moderate ⨁⨁⨁◯ |
| Ritlecitinib vs. Tofacitinib 1 mg BID | Ritlecitinib vs. Placebo vs. Tofacitinib 1 mg BID | Moderate ⨁⨁⨁◯ | Low ⨁⨁◯◯ | Low ⨁⨁◯◯ | Downgrade | Very low ⨁◯◯◯ |
| Ritlecitinib vs. Tofacitinib 10 mg BID | Ritlecitinib vs. Placebo vs. Tofacitinib 10 mg BID | Moderate ⨁⨁⨁◯ | Moderate ⨁⨁⨁◯ | Moderate ⨁⨁⨁◯ | Downgrade | Low ⨁⨁◯◯ |
| Ritlecitinib vs. Tofacitinib 15 mg BID | Ritlecitinib vs. Placebo vs. Tofacitinib 15 mg BID | Moderate ⨁⨁⨁◯ | Moderate ⨁⨁⨁◯ | Moderate ⨁⨁⨁◯ | Downgrade | Low ⨁⨁◯◯ |
| Ritlecitinib vs. Tofacitinib 3 mg BID | Ritlecitinib vs. Placebo vs. Tofacitinib 3 mg BID | Moderate ⨁⨁⨁◯ | Moderate ⨁⨁⨁◯ | Moderate ⨁⨁⨁◯ | No downgrade | Moderate ⨁⨁⨁◯ |
| Ritlecitinib vs. Tofacitinib 5 mg BID | Ritlecitinib vs. Placebo vs. Tofacitinib 5 mg BID | Moderate ⨁⨁⨁◯ | Moderate ⨁⨁⨁◯ | Moderate ⨁⨁⨁◯ | Downgrade | Low ⨁⨁◯◯ |
| Ritlecitinib vs. Upadacitinib 15 mg QD | Ritlecitinib vs. Placebo vs. Upadacitinib 15 mg QD | Moderate ⨁⨁⨁◯ | Moderate ⨁⨁⨁◯ | Moderate ⨁⨁⨁◯ | Downgrade | Low ⨁⨁◯◯ |
| Ritlecitinib vs. Upadacitinib 30 mg QD | Ritlecitinib vs. Placebo vs. Upadacitinib 30 mg QD | Moderate ⨁⨁⨁◯ | Moderate ⨁⨁⨁◯ | Moderate ⨁⨁⨁◯ | Downgrade | Low ⨁⨁◯◯ |
| Ritlecitinib vs. Upadacitinib 45 mg QD | Ritlecitinib vs. Placebo vs. Upadacitinib 45 mg QD | Moderate ⨁⨁⨁◯ | Moderate ⨁⨁⨁◯ | Moderate ⨁⨁⨁◯ | Downgrade | Low ⨁⨁◯◯ |
| Tofacitinib 0.5 mg BID vs. Tofacitinib 1 mg BID | Tofacitinib 0.5 mg BID vs. Placebo vs. Tofacitinib 1 mg BID | Moderate ⨁⨁⨁◯ | Low ⨁⨁◯◯ | Low ⨁⨁◯◯ | Downgrade | Very low ⨁◯◯◯ |
| Tofacitinib 0.5 mg BID vs. Tofacitinib 10 mg BID | Tofacitinib 0.5 mg BID vs. Placebo vs. Tofacitinib 10 mg BID | Moderate ⨁⨁⨁◯ | Moderate ⨁⨁⨁◯ | Moderate ⨁⨁⨁◯ | Downgrade | Low ⨁⨁◯◯ |
| Tofacitinib 0.5 mg BID vs. Tofacitinib 15 mg BID | Tofacitinib 0.5 mg BID vs. Placebo vs. Tofacitinib 15 mg BID | Moderate ⨁⨁⨁◯ | Moderate ⨁⨁⨁◯ | Moderate ⨁⨁⨁◯ | Downgrade | Low ⨁⨁◯◯ |
| Tofacitinib 0.5 mg BID vs. Tofacitinib 5 mg BID | Tofacitinib 0.5 mg BID vs. Placebo vs. Tofacitinib 5 mg BID | Moderate ⨁⨁⨁◯ | Moderate ⨁⨁⨁◯ | Moderate ⨁⨁⨁◯ | Downgrade | Low ⨁⨁◯◯ |
| Tofacitinib 0.5 mg BID vs. Upadacitinib 15 mg QD | Tofacitinib 0.5 mg BID vs. Placebo vs. Upadacitinib 15 mg QD | Moderate ⨁⨁⨁◯ | Moderate ⨁⨁⨁◯ | Moderate ⨁⨁⨁◯ | Downgrade | Low ⨁⨁◯◯ |
| Tofacitinib 0.5 mg BID vs. Upadacitinib 30 mg QD | Tofacitinib 0.5 mg BID vs. Placebo vs. Upadacitinib 30 mg QD | Moderate ⨁⨁⨁◯ | Moderate ⨁⨁⨁◯ | Moderate ⨁⨁⨁◯ | Downgrade | Low ⨁⨁◯◯ |
| Tofacitinib 0.5 mg BID vs. Upadacitinib 45 mg QD | Tofacitinib 0.5 mg BID vs. Placebo vs. Upadacitinib 45 mg QD | Moderate ⨁⨁⨁◯ | Moderate ⨁⨁⨁◯ | Moderate ⨁⨁⨁◯ | Downgrade | Low ⨁⨁◯◯ |
| Tofacitinib 1 mg BID vs. Tofacitinib 10 mg BID | Tofacitinib 1 mg BID vs. Placebo vs. Tofacitinib 10 mg BID | Low ⨁⨁◯◯ | Moderate ⨁⨁⨁◯ | Low ⨁⨁◯◯ | Downgrade | Very low ⨁◯◯◯ |
| Tofacitinib 1 mg BID vs. Tofacitinib 15 mg BID | Tofacitinib 1 mg BID vs. Placebo vs. Tofacitinib 15 mg BID | Low ⨁⨁◯◯ | Moderate ⨁⨁⨁◯ | Low ⨁⨁◯◯ | Downgrade | Very low ⨁◯◯◯ |
| Tofacitinib 1 mg BID vs. Tofacitinib 3 mg BID | Tofacitinib 1 mg BID vs. Placebo vs. Tofacitinib 3 mg BID | Low ⨁⨁◯◯ | Moderate ⨁⨁⨁◯ | Low ⨁⨁◯◯ | Downgrade | Very low ⨁◯◯◯ |
| Tofacitinib 1 mg BID vs. Tofacitinib 5 mg BID | Tofacitinib 1 mg BID vs. Placebo vs. Tofacitinib 5 mg BID | Low ⨁⨁◯◯ | Moderate ⨁⨁⨁◯ | Low ⨁⨁◯◯ | Downgrade | Very low ⨁◯◯◯ |
| Tofacitinib 1 mg BID vs. Upadacitinib 15 mg QD | Tofacitinib 1 mg BID vs. Placebo vs. Upadacitinib 15 mg QD | Low ⨁⨁◯◯ | Moderate ⨁⨁⨁◯ | Low ⨁⨁◯◯ | Downgrade | Very low ⨁◯◯◯ |
| Tofacitinib 1 mg BID vs. Upadacitinib 30 mg QD | Tofacitinib 1 mg BID vs. Placebo vs. Upadacitinib 30 mg QD | Low ⨁⨁◯◯ | Moderate ⨁⨁⨁◯ | Low ⨁⨁◯◯ | Downgrade | Very low ⨁◯◯◯ |
| Tofacitinib 1 mg BID vs. Upadacitinib 45 mg QD | Tofacitinib 1 mg BID vs. Placebo vs. Upadacitinib 45 mg QD | Low ⨁⨁◯◯ | Moderate ⨁⨁⨁◯ | Low ⨁⨁◯◯ | Downgrade | Very low ⨁◯◯◯ |
| Tofacitinib 10 mg BID vs. Tofacitinib 15 mg BID | Tofacitinib 10 mg BID vs. Placebo vs. Tofacitinib 15 mg BID | Moderate ⨁⨁⨁◯ | Moderate ⨁⨁⨁◯ | Moderate ⨁⨁⨁◯ | Downgrade | Low ⨁⨁◯◯ |
| Tofacitinib 10 mg BID vs. Tofacitinib 3 mg BID | Tofacitinib 10 mg BID vs. Placebo vs. Tofacitinib 3 mg BID | Moderate ⨁⨁⨁◯ | Moderate ⨁⨁⨁◯ | Moderate ⨁⨁⨁◯ | Downgrade | Low ⨁⨁◯◯ |
| Tofacitinib 10 mg BID vs. Tofacitinib 5 mg BID | Tofacitinib 10 mg BID vs. Placebo vs. Tofacitinib 5 mg BID | Moderate ⨁⨁⨁◯ | Moderate ⨁⨁⨁◯ | Moderate ⨁⨁⨁◯ | Downgrade | Low ⨁⨁◯◯ |
| Tofacitinib 10 mg BID vs. Upadacitinib 15 mg QD | Tofacitinib 10 mg BID vs. Placebo vs. Upadacitinib 15 mg QD | Moderate ⨁⨁⨁◯ | Moderate ⨁⨁⨁◯ | Moderate ⨁⨁⨁◯ | Downgrade | Low ⨁⨁◯◯ |
| Tofacitinib 10 mg BID vs. Upadacitinib 30 mg QD | Tofacitinib 10 mg BID vs. Placebo vs. Upadacitinib 30 mg QD | Moderate ⨁⨁⨁◯ | Moderate ⨁⨁⨁◯ | Moderate ⨁⨁⨁◯ | Downgrade | Low ⨁⨁◯◯ |
| Tofacitinib 10 mg BID vs. Upadacitinib 45 mg QD | Tofacitinib 10 mg BID vs. Placebo vs. Upadacitinib 45 mg QD | Moderate ⨁⨁⨁◯ | Moderate ⨁⨁⨁◯ | Moderate ⨁⨁⨁◯ | Downgrade | Low ⨁⨁◯◯ |
| Tofacitinib 15 mg BID vs. Tofacitinib 3 mg BID | Tofacitinib 15 mg BID vs. Placebo vs. Tofacitinib 3 mg BID | Moderate ⨁⨁⨁◯ | Moderate ⨁⨁⨁◯ | Moderate ⨁⨁⨁◯ | Downgrade | Low ⨁⨁◯◯ |
| Tofacitinib 15 mg BID vs. Tofacitinib 5 mg BID | Tofacitinib 15 mg BID vs. Placebo vs. Tofacitinib 5 mg BID | Moderate ⨁⨁⨁◯ | Moderate ⨁⨁⨁◯ | Moderate ⨁⨁⨁◯ | Downgrade | Low ⨁⨁◯◯ |
| Tofacitinib 15 mg BID vs. Upadacitinib 15 mg QD | Tofacitinib 15 mg BID vs. Placebo vs. Upadacitinib 15 mg QD | Moderate ⨁⨁⨁◯ | Moderate ⨁⨁⨁◯ | Moderate ⨁⨁⨁◯ | Downgrade | Low ⨁⨁◯◯ |
| Tofacitinib 15 mg BID vs. Upadacitinib 30 mg QD | Tofacitinib 15 mg BID vs. Placebo vs. Upadacitinib 30 mg QD | Moderate ⨁⨁⨁◯ | Moderate ⨁⨁⨁◯ | Moderate ⨁⨁⨁◯ | Downgrade | Low ⨁⨁◯◯ |
| Tofacitinib 15 mg BID vs. Upadacitinib 45 mg QD | Tofacitinib 15 mg BID vs. Placebo vs. Upadacitinib 45 mg QD | Moderate ⨁⨁⨁◯ | Moderate ⨁⨁⨁◯ | Moderate ⨁⨁⨁◯ | No downgrade | Moderate ⨁⨁⨁◯ |
| Tofacitinib 3 mg BID vs. Tofacitinib 5 mg BID | Tofacitinib 3 mg BID vs. Placebo vs. Tofacitinib 5 mg BID | Moderate ⨁⨁⨁◯ | Moderate ⨁⨁⨁◯ | Moderate ⨁⨁⨁◯ | Downgrade | Low ⨁⨁◯◯ |
| Tofacitinib 3 mg BID vs. Upadacitinib 15 mg QD | Tofacitinib 3 mg BID vs. Placebo vs. Upadacitinib 15 mg QD | Moderate ⨁⨁⨁◯ | Moderate ⨁⨁⨁◯ | Moderate ⨁⨁⨁◯ | Downgrade | Low ⨁⨁◯◯ |
| Tofacitinib 3 mg BID vs. Upadacitinib 30 mg QD | Tofacitinib 3 mg BID vs. Placebo vs. Upadacitinib 30 mg QD | Moderate ⨁⨁⨁◯ | Moderate ⨁⨁⨁◯ | Moderate ⨁⨁⨁◯ | Downgrade | Low ⨁⨁◯◯ |
| Tofacitinib 3 mg BID vs. Upadacitinib 45 mg QD | Tofacitinib 3 mg BID vs. Placebo vs. Upadacitinib 45 mg QD | Moderate ⨁⨁⨁◯ | Moderate ⨁⨁⨁◯ | Moderate ⨁⨁⨁◯ | Downgrade | Low ⨁⨁◯◯ |
| Tofacitinib 5 mg BID vs. Upadacitinib 15 mg QD | Tofacitinib 5 mg BID vs. Placebo vs. Upadacitinib 15 mg QD | Moderate ⨁⨁⨁◯ | Moderate ⨁⨁⨁◯ | Moderate ⨁⨁⨁◯ | Downgrade | Low ⨁⨁◯◯ |
| Tofacitinib 5 mg BID vs. Upadacitinib 30 mg QD | Tofacitinib 5 mg BID vs. Placebo vs. Upadacitinib 30 mg QD | Moderate ⨁⨁⨁◯ | Moderate ⨁⨁⨁◯ | Moderate ⨁⨁⨁◯ | Downgrade | Low ⨁⨁◯◯ |
| Tofacitinib 5 mg BID vs. Upadacitinib 45 mg QD | Tofacitinib 5 mg BID vs. Placebo vs. Upadacitinib 45 mg QD | Moderate ⨁⨁⨁◯ | Moderate ⨁⨁⨁◯ | Moderate ⨁⨁⨁◯ | Downgrade | Low ⨁⨁◯◯ |
| Upadacitinib 15 mg QD vs. Upadacitinib 30 mg QD | Upadacitinib 15 mg QD vs. Placebo vs. Upadacitinib 30 mg QD | Moderate ⨁⨁⨁◯ | Moderate ⨁⨁⨁◯ | Moderate ⨁⨁⨁◯ | Downgrade | Low ⨁⨁◯◯ |
| Upadacitinib 15 mg QD vs. Upadacitinib 45 mg QD | Upadacitinib 15 mg QD vs. Placebo vs. Upadacitinib 45 mg QD | Moderate ⨁⨁⨁◯ | Moderate ⨁⨁⨁◯ | Moderate ⨁⨁⨁◯ | Downgrade | Low ⨁⨁◯◯ |
| Upadacitinib 30 mg QD vs. Upadacitinib 45 mg QD | Upadacitinib 30 mg QD vs. Placebo vs. Upadacitinib 45 mg QD | Moderate ⨁⨁⨁◯ | Moderate ⨁⨁⨁◯ | Moderate ⨁⨁⨁◯ | Downgrade | Low ⨁⨁◯◯ |

CVE: cardiovascular events; QD: once daily; BID: twice daily.

**Supplementary table 10-3-1 Certainty of network meta-analysis evidence assessment for MACE (with dose consideration).**

| Comparison | Start rating |  |  | Direct evidence | Indirect evidence | Incoherence | preliminary certainty rating | Odds Ratio (95% CI) | Imprecision | Final certainty rating |
| --- | --- | --- | --- | --- | --- | --- | --- | --- | --- | --- |
|  | Direct preliminary certainty rating | Indirect preliminary certainty rating | Direct/Indirect estimate dominant | Odds Ratio (95% CI) | Odds Ratio (95% CI) |  |  |  |  |  |
| Filgotinib 100 mg QD vs. Filgotinib 200 mg QD | Low ⨁⨁◯◯ | Low ⨁⨁◯◯ | Low ⨁⨁◯◯ | 0.85 (0.17-4.25) | 1.47 (0.00-738.34) | No downgrade | Low ⨁⨁◯◯ | 0.88 (0.19-4.18) | No downgrade | Low ⨁⨁◯◯ |
| Filgotinib 100 mg QD vs. Placebo | Low ⨁⨁◯◯ | Very low ⨁◯◯◯ | Low ⨁⨁◯◯ | 0.65 (0.13-3.27) | 0.38 (0.00-188.29) | No downgrade | Low ⨁⨁◯◯ | 0.63 (0.13-3.00) | No downgrade | Low ⨁⨁◯◯ |
| Placebo vs. Tofacitinib 0.5 mg BID | Low ⨁⨁◯◯ | Very low ⨁◯◯◯ | Low ⨁⨁◯◯ | 1.97 (0.20-19.36) | 0.21 (0.00-1914.59) | No downgrade | Low ⨁⨁◯◯ | 1.72 (0.19-15.83) | Downgrade | Very low ⨁◯◯◯ |
| Placebo vs. Tofacitinib 1 mg BID | Very low ⨁◯◯◯ | Very low ⨁◯◯◯ | Very low ⨁◯◯◯ | 1.06 (0.06-17.28) | 2.21 (0.02-310.98) | No downgrade | Very low ⨁◯◯◯ | 1.27 (0.11-14.40) | Downgrade | Very low ⨁◯◯◯ |
| Placebo vs. Tofacitinib 10 mg BID | Low ⨁⨁◯◯ | Very low ⨁◯◯◯ | Low ⨁⨁◯◯ | 1.84 (0.46-7.43) | 2.07 (0.00-1403.87) | No downgrade | Low ⨁⨁◯◯ | 1.85 (0.47-7.24) | No downgrade | Low ⨁⨁◯◯ |
| Placebo vs. Tofacitinib 15 mg BID | Low ⨁⨁◯◯ | Very low ⨁◯◯◯ | Low ⨁⨁◯◯ | 2.01 (0.34-11.73) | 0.72 (0.00-235.51) | No downgrade | Low ⨁⨁◯◯ | 1.84 (0.34-9.96) | No downgrade | Low ⨁⨁◯◯ |
| Placebo vs. Tofacitinib 3 mg BID | Low ⨁⨁◯◯ | Very low ⨁◯◯◯ | Low ⨁⨁◯◯ | 2.10 (0.21-20.61) | 0.22 (0.00-2029.33) | No downgrade | Low ⨁⨁◯◯ | 1.84 (0.20-16.85) | Downgrade | Very low ⨁◯◯◯ |
| Placebo vs. Tofacitinib 5 mg BID | Low ⨁⨁◯◯ | Very low ⨁◯◯◯ | Low ⨁⨁◯◯ | 0.73 (0.14-3.93) | 3.43 (0.09-137.22) | No downgrade | Low ⨁⨁◯◯ | 0.96 (0.21-4.41) | No downgrade | Low ⨁⨁◯◯ |
| Placebo vs. Upadacitinib 15 mg QD | Low ⨁⨁◯◯ | Very low ⨁◯◯◯ | Low ⨁⨁◯◯ | 1.63 (0.20-13.31) | 1.03 (0.00-1419.71) | No downgrade | Low ⨁⨁◯◯ | 1.57 (0.21-11.81) | Downgrade | Very low ⨁◯◯◯ |
| Placebo vs. Upadacitinib 30 mg QD | Low ⨁⨁◯◯ | Very low ⨁◯◯◯ | Low ⨁⨁◯◯ | 2.15 (0.26-17.53) | 2.25 (0.00-3047.39) | No downgrade | Low ⨁⨁◯◯ | 2.15 (0.29-16.18) | Downgrade | Very low ⨁◯◯◯ |
| Placebo vs. Upadacitinib 45 mg QD | Low ⨁⨁◯◯ | Very low ⨁◯◯◯ | Low ⨁⨁◯◯ | 2.10 (0.36-12.15) | 4.16 (0.00-4000.37) | No downgrade | Low ⨁⨁◯◯ | 2.19 (0.40-12.00) | Downgrade | Very low ⨁◯◯◯ |
| Tofacitinib 0.5 mg BID vs. Tofacitinib 10 mg BID | Low ⨁⨁◯◯ | Very low ⨁◯◯◯ | Low ⨁⨁◯◯ | 1.07 (0.07-17.41) | 1.11 (0.01-140.88) | No downgrade | Low ⨁⨁◯◯ | 1.08 (0.10-12.10) | Downgrade | Very low ⨁◯◯◯ |
| Tofacitinib 0.5 mg BID vs. Tofacitinib 15 mg BID | Low ⨁⨁◯◯ | Very low ⨁◯◯◯ | Low ⨁⨁◯◯ | 1.59 (0.10-25.90) | 0.16 (0.00-71.28) | No downgrade | Low ⨁⨁◯◯ | 1.07 (0.08-13.52) | Downgrade | Very low ⨁◯◯◯ |
| Tofacitinib 1 mg BID vs. Tofacitinib 15 mg BID | Very low ⨁◯◯◯ | Very low ⨁◯◯◯ | Very low ⨁◯◯◯ | 0.97 (0.06-15.85) | 9.89 (0.02-4434.58) | No downgrade | Very low ⨁◯◯◯ | 1.45 (0.11-18.38) | Downgrade | Very low ⨁◯◯◯ |
| Tofacitinib 1 mg BID vs. Tofacitinib 5 mg BID | Very low ⨁◯◯◯ | Very low ⨁◯◯◯ | Very low ⨁◯◯◯ | 0.94 (0.06-15.39) | 0.30 (0.00-87.09) | No downgrade | Very low ⨁◯◯◯ | 0.75 (0.06-9.24) | No downgrade | Very low ⨁◯◯◯ |
| Tofacitinib 10 mg BID vs. Tofacitinib 15 mg BID | Low ⨁⨁◯◯ | Very low ⨁◯◯◯ | Low ⨁⨁◯◯ | 1.49 (0.09-24.29) | 0.66 (0.04-10.71) | No downgrade | Low ⨁⨁◯◯ | 0.99 (0.14-7.12) | No downgrade | Low ⨁⨁◯◯ |
| Tofacitinib 10 mg BID vs. Tofacitinib 3 mg BID | Low ⨁⨁◯◯ | Very low ⨁◯◯◯ | Low ⨁⨁◯◯ | 1.00 (0.06-16.33) | 0.96 (0.01-122.11) | No downgrade | Low ⨁⨁◯◯ | 0.99 (0.09-11.13) | Downgrade | Very low ⨁◯◯◯ |
| Tofacitinib 10 mg BID vs. Tofacitinib 5 mg BID | Low ⨁⨁◯◯ | Very low ⨁◯◯◯ | Low ⨁⨁◯◯ | 0.62 (0.08-5.09) | 0.37 (0.02-6.11) | No downgrade | Low ⨁⨁◯◯ | 0.52 (0.10-2.77) | No downgrade | Low ⨁⨁◯◯ |
| Tofacitinib 15 mg BID vs. Tofacitinib 3 mg BID | Low ⨁⨁◯◯ | Very low ⨁◯◯◯ | Low ⨁⨁◯◯ | 0.67 (0.04-10.91) | 6.86 (0.01-3142.15) | No downgrade | Low ⨁⨁◯◯ | 1.00 (0.08-12.65) | Downgrade | Very low ⨁◯◯◯ |
| Tofacitinib 15 mg BID vs. Tofacitinib 5 mg BID | Very low ⨁◯◯◯ | Very low ⨁◯◯◯ | Very low ⨁◯◯◯ | 0.97 (0.06-15.84) | 0.27 (0.02-4.62) | No downgrade | Very low ⨁◯◯◯ | 0.52 (0.07-3.79) | No downgrade | Very low ⨁◯◯◯ |
| Upadacitinib 15 mg QD vs. Upadacitinib 45 mg QD | Low ⨁⨁◯◯ | Very low ⨁◯◯◯ | Low ⨁⨁◯◯ | 2.53 (0.05-129.10) | 0.96 (0.04-21.60) | No downgrade | Low ⨁⨁◯◯ | 1.39 (0.12-16.01) | Downgrade | Very low ⨁◯◯◯ |
| Upadacitinib 30 mg QD vs. Upadacitinib 45 mg QD | Low ⨁⨁◯◯ | Very low ⨁◯◯◯ | Low ⨁⨁◯◯ | 1.05 (0.02-53.43) | 1.00 (0.04-22.34) | No downgrade | Low ⨁⨁◯◯ | 1.02 (0.09-11.65) | Downgrade | Very low ⨁◯◯◯ |

MACE: major adverse cardiovascular events; QD: once daily; BID: twice daily.

**Supplementary table 10-3-2 Certainty of network meta-analysis evidence assessment for VTE (with dose consideration).**

| Comparison | Start rating | | | Direct evidence | Indirect evidence | Incoherence | preliminary certainty rating | Odds Ratio (95% CI) | Imprecision | Final certainty rating |
| --- | --- | --- | --- | --- | --- | --- | --- | --- | --- | --- |
|  | Direct preliminary certainty rating | Indirect preliminary certainty rating | Direct/Indirect estimate dominant | Odds Ratio (95% CI) | Odds Ratio (95% CI) |  |  |  |  |  |
| Filgotinib 100 mg QD vs. Filgotinib 200 mg QD | Low ⨁⨁◯◯ | Low ⨁⨁◯◯ | Low ⨁⨁◯◯ | 0.66 (0.14-3.11) | 1.28 (0.00-599.74) | No downgrade | Low ⨁⨁◯◯ | 0.69 (0.15-3.09) | No downgrade | Low ⨁⨁◯◯ |
| Filgotinib 100 mg QD vs. Placebo | Low ⨁⨁◯◯ | Very low ⨁◯◯◯ | Low ⨁⨁◯◯ | 0.65 (0.13-3.27) | 0.22 (0.00-102.92) | No downgrade | Low ⨁⨁◯◯ | 0.61 (0.13-2.90) | No downgrade | Low ⨁⨁◯◯ |
| Filgotinib 200 mg QD vs. Placebo | Low ⨁⨁◯◯ | Very low ⨁◯◯◯ | Low ⨁⨁◯◯ | 0.86 (0.22-3.31) | 24.48 (0.00-26509352.12) | No downgrade | Low ⨁⨁◯◯ | 0.88 (0.23-3.39) | No downgrade | Low ⨁⨁◯◯ |
| Placebo vs. Tofacitinib 0.5 mg BID | Low ⨁⨁◯◯ | Very low ⨁◯◯◯ | Low ⨁⨁◯◯ | 1.25 (0.10-15.30) | 3.98 (0.01- 1511.73) | No downgrade | Low ⨁⨁◯◯ | 1.49 (0.15-14.97) | Downgrade | Very low ⨁◯◯◯ |
| Placebo vs. Tofacitinib 1 mg BID | Very low ⨁◯◯◯ | Very low ⨁◯◯◯ | Very low ⨁◯◯◯ | 1.06 (0.06-17.28) | 5.19 (0.03- 796.74) | No downgrade | Very low ⨁◯◯◯ | 1.54 (0.13-17.69) | Downgrade | Very low ⨁◯◯◯ |
| Placebo vs. Tofacitinib 10 mg BID | Low ⨁⨁◯◯ | Very low ⨁◯◯◯ | Low ⨁⨁◯◯ | 2.39 (0.62-9.24) | 1.43 (0.00- 566.68) | No downgrade | Low ⨁⨁◯◯ | 2.34 (0.63-8.71) | No downgrade | Low ⨁⨁◯◯ |
| Placebo vs. Tofacitinib 15 mg BID | Low ⨁⨁◯◯ | Very low ⨁◯◯◯ | Low ⨁⨁◯◯ | 1.49 (0.23- 9.57) | 9.37 (0.07- 1184.68) | No downgrade | Low ⨁⨁◯◯ | 1.89 (0.33-10.71) | Downgrade | Very low ⨁◯◯◯ |
| Placebo vs. Tofacitinib 3 mg BID | Low ⨁⨁◯◯ | Very low ⨁◯◯◯ | Low ⨁⨁◯◯ | 1.34 (0.11-16.29) | 4.24 (0.01- 1603.58) | No downgrade | Low ⨁⨁◯◯ | 1.59 (0.16-15.93) | Downgrade | Very low ⨁◯◯◯ |
| Placebo vs. Tofacitinib 5 mg BID | Low ⨁⨁◯◯ | Very low ⨁◯◯◯ | Low ⨁⨁◯◯ | 1.34 (0.25-7.20) | 9.36 (0.09- 1021.81) | No downgrade | Low ⨁⨁◯◯ | 1.67 (0.34-8.14) | No downgrade | Low ⨁⨁◯◯ |
| Placebo vs. Upadacitinib 15 mg QD | Low ⨁⨁◯◯ | Very low ⨁◯◯◯ | Low ⨁⨁◯◯ | 1.02 (0.11-9.83) | 156.27 (0.61-40362.65) | No downgrade | Low ⨁⨁◯◯ | 2.09 (0.26-17.06) | Downgrade | Very low ⨁◯◯◯ |
| Placebo vs. Upadacitinib 30 mg QD | Low ⨁⨁◯◯ | Very low ⨁◯◯◯ | Low ⨁⨁◯◯ | 0.45 (0.07-3.09) | 12.30 (0.12-1230.16) | No downgrade | Low ⨁⨁◯◯ | 0.74 (0.12-4.35) | No downgrade | Low ⨁⨁◯◯ |
| Placebo vs. Upadacitinib 45 mg QD | Low ⨁⨁◯◯ | Very low ⨁◯◯◯ | Low ⨁⨁◯◯ | 2.14 (0.45-10.21) | 0.11 (0.00-4.86) | No downgrade | Low ⨁⨁◯◯ | 1.39 (0.33-5.92) | No downgrade | Low ⨁⨁◯◯ |
| Tofacitinib 0.5 mg BID vs. Tofacitinib 10 mg BID | Low ⨁⨁◯◯ | Very low ⨁◯◯◯ | Low ⨁⨁◯◯ | 1.07 (0.07-17.41) | 5.15 (0.04- 709.65) | No downgrade | Low ⨁⨁◯◯ | 1.56 (0.14-17.76) | Downgrade | Very low ⨁◯◯◯ |
| Tofacitinib 0.5 mg BID vs. Tofacitinib 15 mg BID | Low ⨁⨁◯◯ | Very low ⨁◯◯◯ | Low ⨁⨁◯◯ | 1.59 (0.10-25.90) | 0.39 (0.00- 213.00) | No downgrade | Low ⨁⨁◯◯ | 1.26 (0.10-16.20) | Downgrade | Very low ⨁◯◯◯ |
| Tofacitinib 1 mg BID vs. Tofacitinib 15 mg BID | Very low ⨁◯◯◯ | Very low ⨁◯◯◯ | Very low ⨁◯◯◯ | 0.97 (0.06-15.85) | 3.95 (0.01-2107.01) | No downgrade | Very low ⨁◯◯◯ | 1.22 (0.10-15.70) | Downgrade | Very low ⨁◯◯◯ |
| Tofacitinib 1 mg BID vs. Tofacitinib 5 mg BID | Very low ⨁◯◯◯ | Very low ⨁◯◯◯ | Very low ⨁◯◯◯ | 0.94 (0.06-15.39) | 2.01 (0.01-676.42) | No downgrade | Very low ⨁◯◯◯ | 1.09 (0.09-13.48) | Downgrade | Very low ⨁◯◯◯ |
| Tofacitinib 10 mg BID vs. Tofacitinib 15 mg BID | Low ⨁⨁◯◯ | Very low ⨁◯◯◯ | Low ⨁⨁◯◯ | 1.49 (0.09-24.29) | 0.44 (0.03-7.02) | No downgrade | Low ⨁⨁◯◯ | 0.81 (0.11-5.76) | No downgrade | Low ⨁⨁◯◯ |
| Tofacitinib 10 mg BID vs. Tofacitinib 3 mg BID | Low ⨁⨁◯◯ | Very low ⨁◯◯◯ | Low ⨁⨁◯◯ | 1.00 (0.06-16.33) | 0.21 (0.00-28.43) | No downgrade | Low ⨁⨁◯◯ | 0.68 (0.06-7.73) | No downgrade | Low ⨁⨁◯◯ |
| Tofacitinib 10 mg BID vs. Tofacitinib 5 mg BID | Low ⨁⨁◯◯ | Very low ⨁◯◯◯ | Low ⨁⨁◯◯ | 1.00 (0.10-9.65) | 0.42 (0.02-7.59) | No downgrade | Low ⨁⨁◯◯ | 0.72 (0.12-4.28) | No downgrade | Low ⨁⨁◯◯ |
| Tofacitinib 15 mg BID vs. Tofacitinib 3 mg BID | Low ⨁⨁◯◯ | Very low ⨁◯◯◯ | Low ⨁⨁◯◯ | 0.67 (0.04-10.91) | 2.73 (0.01-1489.75) | No downgrade | Low ⨁⨁◯◯ | 0.84 (0.07-10.81) | Downgrade | Very low ⨁◯◯◯ |
| Tofacitinib 15 mg BID vs. Tofacitinib 5 mg BID | Very low ⨁◯◯◯ | Very low ⨁◯◯◯ | Very low ⨁◯◯◯ | 0.97 (0.06-15.84) | 0.80 (0.04-15.63) | No downgrade | Very low ⨁◯◯◯ | 0.89 (0.12-6.79) | No downgrade | Very low ⨁◯◯◯ |
| Upadacitinib 15 mg QD vs. Upadacitinib 30 mg QD | Low ⨁⨁◯◯ | Very low ⨁◯◯◯ | Low ⨁⨁◯◯ | 0.45 (0.07-3.06) | 0.00 (0.00-8.35) | No downgrade | Low ⨁⨁◯◯ | 0.35 (0.05-2.35) | No downgrade | Low ⨁⨁◯◯ |
| Upadacitinib 15 mg QD vs. Upadacitinib 45 mg QD | Low ⨁⨁◯◯ | Very low ⨁◯◯◯ | Low ⨁⨁◯◯ | 0.50 (0.02-10.54) | 0.89 (0.04-18.30) | No downgrade | Low ⨁⨁◯◯ | 0.67 (0.08-5.72) | No downgrade | Low ⨁⨁◯◯ |
| Upadacitinib 30 mg QD vs. Upadacitinib 45 mg QD | Low ⨁⨁◯◯ | Very low ⨁◯◯◯ | Low ⨁⨁◯◯ | 0.21 (0.01-4.36) | 8.36 (0.69-101.31) | No downgrade | Low ⨁⨁◯◯ | 1.89 (0.27-13.05) | Downgrade | Very low ⨁◯◯◯ |

VTE: venous thromboembolism events; QD: once daily; BID: twice daily.

**Supplementary table 10-3-3 Certainty of network meta-analysis evidence assessment for CVE (with dose consideration).**

| Comparison | Start rating | | | Direct evidence | Indirect evidence | Incoherence | preliminary certainty rating | Odds Ratio (95% CI) | Imprecision | Final certainty rating |
| --- | --- | --- | --- | --- | --- | --- | --- | --- | --- | --- |
|  | Direct preliminary certainty rating | Indirect preliminary certainty rating | Direct/Indirect estimate dominant | Odds Ratio (95% CI) | Odds Ratio (95% CI) |  |  |  |  |  |
| Filgotinib 100 mg QD vs. Filgotinib 200 mg QD | Moderate ⨁⨁⨁◯ | Moderate ⨁⨁⨁◯ | Moderate ⨁⨁⨁◯ | 0.66 (0.14-3.11) | 1.28 (0.00-599.74) | No downgrade | Moderate ⨁⨁⨁◯ | 0.69 (0.15-3.09) | No downgrade | Moderate ⨁⨁⨁◯ |
| Filgotinib 100 mg QD vs. Placebo | Moderate ⨁⨁⨁◯ | Low ⨁⨁◯◯ | Moderate ⨁⨁⨁◯ | 0.65 (0.13-3.27) | 0.22 (0.00-102.92) | No downgrade | Moderate ⨁⨁⨁◯ | 0.61 (0.13-2.90) | No downgrade | Moderate ⨁⨁⨁◯ |
| Filgotinib 200 mg QD vs. Placebo | Moderate ⨁⨁⨁◯ | Low ⨁⨁◯◯ | Moderate ⨁⨁⨁◯ | 0.86 (0.22-3.31) | 24.48 (0.00-26509352.12) | No downgrade | Moderate ⨁⨁⨁◯ | 0.88 (0.23-3.39) | No downgrade | Moderate ⨁⨁⨁◯ |
| Placebo vs. Tofacitinib 0.5 mg BID | Moderate ⨁⨁⨁◯ | Low ⨁⨁◯◯ | Moderate ⨁⨁⨁◯ | 2.60 (0.28-24.13) | 0.47 (0.00-11812.40) | No downgrade | Moderate ⨁⨁⨁◯ | 2.41 (0.27-21.17) | Downgrade | Low ⨁⨁◯◯ |
| Placebo vs. Tofacitinib 1 mg BID | Low ⨁⨁◯◯ | Very low ⨁◯◯◯ | Low ⨁⨁◯◯ | 1.06 (0.06-17.28) | 4.65 (0.04-567.94) | No downgrade | Low ⨁⨁◯◯ | 1.54 (0.14-17.21) | Downgrade | Very low ⨁◯◯◯ |
| Placebo vs. Tofacitinib 10 mg BID | Moderate ⨁⨁⨁◯ | Low ⨁⨁◯◯ | Moderate ⨁⨁⨁◯ | 2.95 (0.80-10.83) | 0.80 (0.00-979.59) | No downgrade | Moderate ⨁⨁⨁◯ | 2.83 (0.79-10.17) | Downgrade | Low ⨁⨁◯◯ |
| Placebo vs. Tofacitinib 15 mg BID | Moderate ⨁⨁⨁◯ | Low ⨁⨁◯◯ | Moderate ⨁⨁⨁◯ | 2.41 (0.42-13.73) | 2.20 (0.01-543.29) | No downgrade | Moderate ⨁⨁⨁◯ | 2.39 (0.46-12.56) | Downgrade | Low ⨁⨁◯◯ |
| Placebo vs. Tofacitinib 3 mg BID | Moderate ⨁⨁⨁◯ | Low ⨁⨁◯◯ | Moderate ⨁⨁⨁◯ | 2.77 (0.30-25.69) | 0.50 (0.00-12501.05) | No downgrade | Moderate ⨁⨁⨁◯ | 2.56 (0.29-22.53) | Downgrade | Low ⨁⨁◯◯ |
| Placebo vs. Tofacitinib 5 mg BID | Moderate ⨁⨁⨁◯ | Low ⨁⨁◯◯ | Moderate ⨁⨁⨁◯ | 0.99 (0.22-4.41) | 11.29 (0.19-685.36) | No downgrade | Moderate ⨁⨁⨁◯ | 1.32 (0.33-5.34) | No downgrade | Moderate ⨁⨁⨁◯ |
| Placebo vs. Upadacitinib 15 mg QD | Moderate ⨁⨁⨁◯ | Low ⨁⨁◯◯ | Moderate ⨁⨁⨁◯ | 1.63 (0.20-13.31) | 41.54 (0.20-8629.56) | No downgrade | Moderate ⨁⨁⨁◯ | 2.51 (0.36-17.76) | Downgrade | Low ⨁⨁◯◯ |
| Placebo vs. Upadacitinib 30 mg QD | Moderate ⨁⨁⨁◯ | Low ⨁⨁◯◯ | Moderate ⨁⨁⨁◯ | 0.67 (0.14-3.24) | 22.05 (0.16-3096.34) | No downgrade | Moderate ⨁⨁⨁◯ | 0.93 (0.21-4.15) | No downgrade | Moderate ⨁⨁⨁◯ |
| Placebo vs. Upadacitinib 45 mg QD | Moderate ⨁⨁⨁◯ | Low ⨁⨁◯◯ | Moderate ⨁⨁⨁◯ | 2.14 (0.45-10.21) | 0.24 (0.01-7.74) | No downgrade | Moderate ⨁⨁⨁◯ | 1.48 (0.36-6.15) | No downgrade | Moderate ⨁⨁⨁◯ |
| Tofacitinib 0.5 mg BID vs. Tofacitinib 10 mg BID | Moderate ⨁⨁⨁◯ | Low ⨁⨁◯◯ | Moderate ⨁⨁⨁◯ | 1.07 (0.07-17.41) | 1.53 (0.02-151.77) | No downgrade | Moderate ⨁⨁⨁◯ | 1.18 (0.11-12.79) | Downgrade | Low ⨁⨁◯◯ |
| Tofacitinib 0.5 mg BID vs. Tofacitinib 15 mg BID | Moderate ⨁⨁⨁◯ | Low ⨁⨁◯◯ | Moderate ⨁⨁⨁◯ | 1.59 (0.10-25.90) | 0.11 (0.00-46.71) | No downgrade | Moderate ⨁⨁⨁◯ | 1.00 (0.08-12.54) | Downgrade | Low ⨁⨁◯◯ |
| Tofacitinib 1 mg BID vs. Tofacitinib 15 mg BID | Low ⨁⨁◯◯ | Very low ⨁◯◯◯ | Low ⨁⨁◯◯ | 0.97 (0.06-15.85) | 13.92 (0.03-5768.77) | No downgrade | Low ⨁⨁◯◯ | 1.56 (0.12-19.58) | Downgrade | Very low ⨁◯◯◯ |
| Tofacitinib 1 mg BID vs. Tofacitinib 5 mg BID | Low ⨁⨁◯◯ | Very low ⨁◯◯◯ | Low ⨁⨁◯◯ | 0.94 (0.06-15.39) | 0.60 (0.00-132.70) | No downgrade | Low ⨁⨁◯◯ | 0.86 (0.07-10.23) | Downgrade | Very low ⨁◯◯◯ |
| Tofacitinib 10 mg BID vs. Tofacitinib 15 mg BID | Moderate ⨁⨁⨁◯ | Low ⨁⨁◯◯ | Moderate ⨁⨁⨁◯ | 1.49 (0.09-24.29) | 0.49 (0.03-7.54) | No downgrade | Moderate ⨁⨁⨁◯ | 0.85 (0.12-5.95) | No downgrade | Moderate ⨁⨁⨁◯ |
| Tofacitinib 10 mg BID vs. Tofacitinib 3 mg BID | Moderate ⨁⨁⨁◯ | Low ⨁⨁◯◯ | Moderate ⨁⨁⨁◯ | 1.00 (0.06-16.33) | 0.70 (0.01-68.76) | No downgrade | Moderate ⨁⨁⨁◯ | 0.91 (0.08-9.86) | No downgrade | Moderate ⨁⨁⨁◯ |
| Tofacitinib 10 mg BID vs. Tofacitinib 5 mg BID | Moderate ⨁⨁⨁◯ | Low ⨁⨁◯◯ | Moderate ⨁⨁⨁◯ | 0.62 (0.08-5.09) | 0.30 (0.02-4.09) | No downgrade | Moderate ⨁⨁⨁◯ | 0.47 (0.09-2.40) | No downgrade | Moderate ⨁⨁⨁◯ |
| Tofacitinib 15 mg BID vs. Tofacitinib 3 mg BID | Moderate ⨁⨁⨁◯ | Low ⨁⨁◯◯ | Moderate ⨁⨁⨁◯ | 0.67 (0.04-10.91) | 9.67 (0.02-4090.65) | No downgrade | Moderate ⨁⨁⨁◯ | 1.07 (0.09-13.48) | Downgrade | Low ⨁⨁◯◯ |
| Tofacitinib 15 mg BID vs. Tofacitinib 5 mg BID | Low ⨁⨁◯◯ | Low ⨁⨁◯◯ | Low ⨁⨁◯◯ | 0.97 (0.06-15.84) | 0.33 (0.02-4.74) | No downgrade | Low ⨁⨁◯◯ | 0.55 (0.08-3.80) | No downgrade | Low ⨁⨁◯◯ |
| Upadacitinib 15 mg QD vs. Upadacitinib 30 mg QD | Moderate ⨁⨁⨁◯ | Low ⨁⨁◯◯ | Moderate ⨁⨁⨁◯ | 0.45 (0.07-3.06) | 0.00 (0.00-47.29) | No downgrade | Moderate ⨁⨁⨁◯ | 0.37 (0.06-2.44) | No downgrade | Moderate ⨁⨁⨁◯ |
| Upadacitinib 15 mg QD vs. Upadacitinib 45 mg QD | Moderate ⨁⨁⨁◯ | Low ⨁⨁◯◯ | Moderate ⨁⨁⨁◯ | 0.50 (0.02-10.54) | 0.69 (0.04-11.90) | No downgrade | Moderate ⨁⨁⨁◯ | 0.59 (0.07-4.75) | No downgrade | Moderate ⨁⨁⨁◯ |
| Upadacitinib 30 mg QD vs. Upadacitinib 45 mg QD | Moderate ⨁⨁⨁◯ | Low ⨁⨁◯◯ | Moderate ⨁⨁⨁◯ | 0.21 (0.01-4.36) | 4.80 (0.52-44.73) | No downgrade | Moderate ⨁⨁⨁◯ | 1.60 (0.26-9.69) | No downgrade | Moderate ⨁⨁⨁◯ |

CVE: cardiovascular events; QD: once daily; BID: twice daily.

**Supplementary table 10-4-1 Selection of final certainty rating for MACE (with dose consideration).**

| Comparison | Final certainty ratings | | | | | | Best estimate and certainty rating |
| --- | --- | --- | --- | --- | --- | --- | --- |
|  | Final direct certainty rating | Odds Ratio (95% CI) | Final indirect certainty rating | Odds Ratio (95% CI) | Final NMA certainty rating | Odds Ratio (95% CI) |  |
| Filgotinib 100 mg QD vs. Filgotinib 200 mg QD | Low ⨁⨁◯◯ | 0.85 (0.17-4.25) | Very low ⨁◯◯◯ | 1.47 (0.00-738.34) | Low ⨁⨁◯◯ | 0.88 (0.19-4.18) | Low ⨁⨁◯◯ |
| Filgotinib 100 mg QD vs. Placebo | Low ⨁⨁◯◯ | 0.65 (0.13-3.27) | Very low ⨁◯◯◯ | 0.38 (0.00-188.29) | Low ⨁⨁◯◯ | 0.63 (0.13-3.00) | Low ⨁⨁◯◯ |
| Placebo vs. Tofacitinib 0.5 mg BID | Very low ⨁◯◯◯ | 1.97 (0.20-19.36) | Very low ⨁◯◯◯ | 0.21 (0.00-1914.59) | Very low ⨁◯◯◯ | 1.72 (0.19-15.83) | Very low ⨁◯◯◯ |
| Placebo vs. Tofacitinib 1 mg BID | Very low ⨁◯◯◯ | 1.06 (0.06-17.28) | Very low ⨁◯◯◯ | 2.21 (0.02-310.98) | Very low ⨁◯◯◯ | 1.27 (0.11-14.40) | Very low ⨁◯◯◯ |
| Placebo vs. Tofacitinib 10 mg BID | Low ⨁⨁◯◯ | 1.84 (0.46-7.43) | Very low ⨁◯◯◯ | 2.07 (0.00-1403.87) | Low ⨁⨁◯◯ | 1.85 (0.47-7.24) | Low ⨁⨁◯◯ |
| Placebo vs. Tofacitinib 15 mg BID | Very low ⨁◯◯◯ | 2.01 (0.34-11.73) | Very low ⨁◯◯◯ | 0.72 (0.00-235.51) | Low ⨁⨁◯◯ | 1.84 (0.34-9.96) | Low ⨁⨁◯◯ |
| Placebo vs. Tofacitinib 3 mg BID | Very low ⨁◯◯◯ | 2.10 (0.21-20.61) | Very low ⨁◯◯◯ | 0.22 (0.00-2029.33) | Very low ⨁◯◯◯ | 1.84 (0.20-16.85) | Very low ⨁◯◯◯ |
| Placebo vs. Tofacitinib 5 mg BID | Low ⨁⨁◯◯ | 0.73 (0.14-3.93) | Very low ⨁◯◯◯ | 3.43 (0.09-137.22) | Low ⨁⨁◯◯ | 0.96 (0.21-4.41) | Low ⨁⨁◯◯ |
| Placebo vs. Upadacitinib 15 mg QD | Very low ⨁◯◯◯ | 1.63 (0.20-13.31) | Very low ⨁◯◯◯ | 1.03 (0.00-1419.71) | Very low ⨁◯◯◯ | 1.57 (0.21-11.81) | Very low ⨁◯◯◯ |
| Placebo vs. Upadacitinib 30 mg QD | Very low ⨁◯◯◯ | 2.15 (0.26-17.53) | Very low ⨁◯◯◯ | 2.25 (0.00-3047.39) | Very low ⨁◯◯◯ | 2.15 (0.29-16.18) | Very low ⨁◯◯◯ |
| Placebo vs. Upadacitinib 45 mg QD | Very low ⨁◯◯◯ | 2.10 (0.36-12.15) | Very low ⨁◯◯◯ | 4.16 (0.00-4000.37) | Very low ⨁◯◯◯ | 2.19 (0.40-12.00) | Very low ⨁◯◯◯ |
| Tofacitinib 0.5 mg BID vs. Tofacitinib 10 mg BID | Very low ⨁◯◯◯ | 1.07 (0.07-17.41) | Very low ⨁◯◯◯ | 1.11 (0.01-140.88) | Very low ⨁◯◯◯ | 1.08 (0.10-12.10) | Very low ⨁◯◯◯ |
| Tofacitinib 0.5 mg BID vs. Tofacitinib 15 mg BID | Very low ⨁◯◯◯ | 1.59 (0.10-25.90) | Very low ⨁◯◯◯ | 0.16 (0.00-71.28) | Very low ⨁◯◯◯ | 1.07 (0.08-13.52) | Very low ⨁◯◯◯ |
| Tofacitinib 1 mg BID vs. Tofacitinib 15 mg BID | Very low ⨁◯◯◯ | 0.97 (0.06-15.85) | Very low ⨁◯◯◯ | 9.89 (0.02-4434.58) | Very low ⨁◯◯◯ | 1.45 (0.11-18.38) | Very low ⨁◯◯◯ |
| Tofacitinib 1 mg BID vs. Tofacitinib 5 mg BID | Very low ⨁◯◯◯ | 0.94 (0.06-15.39) | Very low ⨁◯◯◯ | 0.30 (0.00-87.09) | Very low ⨁◯◯◯ | 0.75 (0.06-9.24) | Very low ⨁◯◯◯ |
| Tofacitinib 10 mg BID vs. Tofacitinib 15 mg BID | Very low ⨁◯◯◯ | 1.49 (0.09-24.29) | Very low ⨁◯◯◯ | 0.66 (0.04-10.71) | Low ⨁⨁◯◯ | 0.99 (0.14-7.12) | Low ⨁⨁◯◯ |
| Tofacitinib 10 mg BID vs. Tofacitinib 3 mg BID | Very low ⨁◯◯◯ | 1.00 (0.06-16.33) | Very low ⨁◯◯◯ | 0.96 (0.01-122.11) | Very low ⨁◯◯◯ | 0.99 (0.09-11.13) | Very low ⨁◯◯◯ |
| Tofacitinib 10 mg BID vs. Tofacitinib 5 mg BID | Low ⨁⨁◯◯ | 0.62 (0.08-5.09) | Very low ⨁◯◯◯ | 0.37 (0.02-6.11) | Low ⨁⨁◯◯ | 0.52 (0.10-2.77) | Low ⨁⨁◯◯ |
| Tofacitinib 15 mg BID vs. Tofacitinib 3 mg BID | Very low ⨁◯◯◯ | 0.67 (0.04-10.91) | Very low ⨁◯◯◯ | 6.86 (0.01-3142.15) | Very low ⨁◯◯◯ | 1.00 (0.08-12.65) | Very low ⨁◯◯◯ |
| Tofacitinib 15 mg BID vs. Tofacitinib 5 mg BID | Very low ⨁◯◯◯ | 0.97 (0.06-15.84) | Very low ⨁◯◯◯ | 0.27 (0.02-4.62) | Very low ⨁◯◯◯ | 0.52 (0.07-3.79) | Very low ⨁◯◯◯ |
| Upadacitinib 15 mg QD vs. Upadacitinib 45 mg QD | Very low ⨁◯◯◯ | 2.53 (0.05-129.10) | Very low ⨁◯◯◯ | 0.96 (0.04-21.60) | Very low ⨁◯◯◯ | 1.39 (0.12-16.01) | Very low ⨁◯◯◯ |
| Upadacitinib 30 mg QD vs. Upadacitinib 45 mg QD | Very low ⨁◯◯◯ | 1.05 (0.02-53.43) | Very low ⨁◯◯◯ | 1.00 (0.04-22.34) | Very low ⨁◯◯◯ | 1.02 (0.09-11.65) | Very low ⨁◯◯◯ |

MACE: major adverse cardiovascular events; QD: once daily; BID: twice daily.

**Supplementary table 10-4-2 Selection of final certainty rating for VTE (with dose consideration).**

| Comparison | Final certainty ratings | | | | | | Best estimate and certainty rating |
| --- | --- | --- | --- | --- | --- | --- | --- |
|  | Final direct certainty rating | Odds Ratio (95% CI) | Final indirect certainty rating | Odds Ratio (95% CI) | Final NMA certainty rating | Odds Ratio (95% CI) |  |
| Filgotinib 100 mg QD vs. Filgotinib 200 mg QD | Low ⨁⨁◯◯ | 0.66 (0.14-3.11) | Very low ⨁◯◯◯ | 1.28 (0.00-599.74) | Low ⨁⨁◯◯ | 0.69 (0.15-3.09) | Low ⨁⨁◯◯ |
| Filgotinib 100 mg QD vs. Placebo | Low ⨁⨁◯◯ | 0.65 (0.13-3.27) | Very low ⨁◯◯◯ | 0.22 (0.00-102.92) | Low ⨁⨁◯◯ | 0.61 (0.13-2.90) | Low ⨁⨁◯◯ |
| Filgotinib 200 mg QD vs. Placebo | Low ⨁⨁◯◯ | 0.86 (0.22-3.31) | Very low ⨁◯◯◯ | 24.48 (0.00-26509352.12) | Low ⨁⨁◯◯ | 0.88 (0.23-3.39) | Low ⨁⨁◯◯ |
| Placebo vs. Tofacitinib 0.5 mg BID | Very low ⨁◯◯◯ | 1.25 (0.10-15.30) | Very low ⨁◯◯◯ | 3.98 (0.01- 1511.73) | Very low ⨁◯◯◯ | 1.49 (0.15-14.97) | Very low ⨁◯◯◯ |
| Placebo vs. Tofacitinib 1 mg BID | Very low ⨁◯◯◯ | 1.06 (0.06-17.28) | Very low ⨁◯◯◯ | 5.19 (0.03- 796.74) | Very low ⨁◯◯◯ | 1.54 (0.13-17.69) | Very low ⨁◯◯◯ |
| Placebo vs. Tofacitinib 10 mg BID | Low ⨁⨁◯◯ | 2.39 (0.62-9.24) | Very low ⨁◯◯◯ | 1.43 (0.00- 566.68) | Low ⨁⨁◯◯ | 2.34 (0.63-8.71) | Low ⨁⨁◯◯ |
| Placebo vs. Tofacitinib 15 mg BID | Low ⨁⨁◯◯ | 1.49 (0.23- 9.57) | Very low ⨁◯◯◯ | 9.37 (0.07- 1184.68) | Very low ⨁◯◯◯ | 1.89 (0.33-10.71) | Low ⨁⨁◯◯ |
| Placebo vs. Tofacitinib 3 mg BID | Very low ⨁◯◯◯ | 1.34 (0.11-16.29) | Very low ⨁◯◯◯ | 4.24 (0.01- 1603.58) | Very low ⨁◯◯◯ | 1.59 (0.16-15.93) | Very low ⨁◯◯◯ |
| Placebo vs. Tofacitinib 5 mg BID | Low ⨁⨁◯◯ | 1.34 (0.25-7.20) | Very low ⨁◯◯◯ | 9.36 (0.09- 1021.81) | Low ⨁⨁◯◯ | 1.67 (0.34-8.14) | Low ⨁⨁◯◯ |
| Placebo vs. Upadacitinib 15 mg QD | Low ⨁⨁◯◯ | 1.02 (0.11-9.83) | Very low ⨁◯◯◯ | 156.27 (0.61-40362.65) | Very low ⨁◯◯◯ | 2.09 (0.26-17.06) | Low ⨁⨁◯◯ |
| Placebo vs. Upadacitinib 30 mg QD | Low ⨁⨁◯◯ | 0.45 (0.07-3.09) | Very low ⨁◯◯◯ | 12.30 (0.12-1230.16) | Low ⨁⨁◯◯ | 0.74 (0.12-4.35) | Low ⨁⨁◯◯ |
| Placebo vs. Upadacitinib 45 mg QD | Very low ⨁◯◯◯ | 2.14 (0.45-10.21) | Very low ⨁◯◯◯ | 0.11 (0.00-4.86) | Low ⨁⨁◯◯ | 1.39 (0.33-5.92) | Low ⨁⨁◯◯ |
| Tofacitinib 0.5 mg BID vs. Tofacitinib 10 mg BID | Very low ⨁◯◯◯ | 1.07 (0.07-17.41) | Very low ⨁◯◯◯ | 5.15 (0.04- 709.65) | Very low ⨁◯◯◯ | 1.56 (0.14-17.76) | Very low ⨁◯◯◯ |
| Tofacitinib 0.5 mg BID vs. Tofacitinib 15 mg BID | Very low ⨁◯◯◯ | 1.59 (0.10-25.90) | Very low ⨁◯◯◯ | 0.39 (0.00- 213.00) | Very low ⨁◯◯◯ | 1.26 (0.10-16.20) | Very low ⨁◯◯◯ |
| Tofacitinib 1 mg BID vs. Tofacitinib 15 mg BID | Very low ⨁◯◯◯ | 0.97 (0.06-15.85) | Very low ⨁◯◯◯ | 3.95 (0.01-2107.01) | Very low ⨁◯◯◯ | 1.22 (0.10-15.70) | Very low ⨁◯◯◯ |
| Tofacitinib 1 mg BID vs. Tofacitinib 5 mg BID | Very low ⨁◯◯◯ | 0.94 (0.06-15.39) | Very low ⨁◯◯◯ | 2.01 (0.01-676.42) | Very low ⨁◯◯◯ | 1.09 (0.09-13.48) | Very low ⨁◯◯◯ |
| Tofacitinib 10 mg BID vs. Tofacitinib 15 mg BID | Very low ⨁◯◯◯ | 1.49 (0.09-24.29) | Very low ⨁◯◯◯ | 0.44 (0.03-7.02) | Low ⨁⨁◯◯ | 0.81 (0.11-5.76) | Low ⨁⨁◯◯ |
| Tofacitinib 10 mg BID vs. Tofacitinib 3 mg BID | Very low ⨁◯◯◯ | 1.00 (0.06-16.33) | Very low ⨁◯◯◯ | 0.21 (0.00-28.43) | Low ⨁⨁◯◯ | 0.68 (0.06-7.73) | Low ⨁⨁◯◯ |
| Tofacitinib 10 mg BID vs. Tofacitinib 5 mg BID | Low ⨁⨁◯◯ | 1.00 (0.10-9.65) | Very low ⨁◯◯◯ | 0.42 (0.02-7.59) | Low ⨁⨁◯◯ | 0.72 (0.12-4.28) | Low ⨁⨁◯◯ |
| Tofacitinib 15 mg BID vs. Tofacitinib 3 mg BID | Very low ⨁◯◯◯ | 0.67 (0.04-10.91) | Very low ⨁◯◯◯ | 2.73 (0.01-1489.75) | Very low ⨁◯◯◯ | 0.84 (0.07-10.81) | Very low ⨁◯◯◯ |
| Tofacitinib 15 mg BID vs. Tofacitinib 5 mg BID | Very low ⨁◯◯◯ | 0.97 (0.06-15.84) | Very low ⨁◯◯◯ | 0.80 (0.04-15.63) | Very low ⨁◯◯◯ | 0.89 (0.12-6.79) | Very low ⨁◯◯◯ |
| Upadacitinib 15 mg QD vs. Upadacitinib 30 mg QD | Low ⨁⨁◯◯ | 0.45 (0.07-3.06) | Very low ⨁◯◯◯ | 0.00 (0.00-8.35) | Low ⨁⨁◯◯ | 0.35 (0.05-2.35) | Low ⨁⨁◯◯ |
| Upadacitinib 15 mg QD vs. Upadacitinib 45 mg QD | Very low ⨁◯◯◯ | 0.50 (0.02-10.54) | Very low ⨁◯◯◯ | 0.89 (0.04-18.30) | Low ⨁⨁◯◯ | 0.67 (0.08-5.72) | Low ⨁⨁◯◯ |
| Upadacitinib 30 mg QD vs. Upadacitinib 45 mg QD | Low ⨁⨁◯◯ | 0.21 (0.01-4.36) | Very low ⨁◯◯◯ | 8.36 (0.69-101.31) | Very low ⨁◯◯◯ | 1.89 (0.27-13.05) | Low ⨁⨁◯◯ |

VTE: venous thromboembolism events; QD: once daily; BID: twice daily.

**Supplementary table 10-4-3 Selection of final certainty rating for CVE (with dose consideration).**

| Comparison | Final certainty ratings | | | | | | Best estimate and certainty rating |
| --- | --- | --- | --- | --- | --- | --- | --- |
|  | Final direct certainty rating | Odds Ratio (95% CI) | Final indirect certainty rating | Odds Ratio (95% CI) | Final NMA certainty rating | Odds Ratio (95% CI) |  |
| Filgotinib 100 mg QD vs. Filgotinib 200 mg QD | Moderate ⨁⨁⨁◯ | 0.66 (0.14-3.11) | Low ⨁⨁◯◯ | 1.28 (0.00-599.74) | Moderate ⨁⨁⨁◯ | 0.69 (0.15-3.09) | Moderate ⨁⨁⨁◯ |
| Filgotinib 100 mg QD vs. Placebo | Moderate ⨁⨁⨁◯ | 0.65 (0.13-3.27) | Very low ⨁◯◯◯ | 0.22 (0.00-102.92) | Moderate ⨁⨁⨁◯ | 0.61 (0.13-2.90) | Moderate ⨁⨁⨁◯ |
| Filgotinib 200 mg QD vs. Placebo | Moderate ⨁⨁⨁◯ | 0.86 (0.22-3.31) | Very low ⨁◯◯◯ | 24.48 (0.00-26509352.12) | Moderate ⨁⨁⨁◯ | 0.88 (0.23-3.39) | Moderate ⨁⨁⨁◯ |
| Placebo vs. Tofacitinib 0.5 mg BID | Low ⨁⨁◯◯ | 2.60 (0.28-24.13) | Very low ⨁◯◯◯ | 0.47 (0.00-11812.40) | Low ⨁⨁◯◯ | 2.41 (0.27-21.17) | Low ⨁⨁◯◯ |
| Placebo vs. Tofacitinib 1 mg BID | Very low ⨁◯◯◯ | 1.06 (0.06-17.28) | Very low ⨁◯◯◯ | 4.65 (0.04-567.94) | Very low ⨁◯◯◯ | 1.54 (0.14-17.21) | Very low ⨁◯◯◯ |
| Placebo vs. Tofacitinib 10 mg BID | Low ⨁⨁◯◯ | 2.95 (0.80-10.83) | Very low ⨁◯◯◯ | 0.80 (0.00-979.59) | Low ⨁⨁◯◯ | 2.83 (0.79-10.17) | Low ⨁⨁◯◯ |
| Placebo vs. Tofacitinib 15 mg BID | Low ⨁⨁◯◯ | 2.41 (0.42-13.73) | Very low ⨁◯◯◯ | 2.20 (0.01-543.29) | Low ⨁⨁◯◯ | 2.39 (0.46-12.56) | Low ⨁⨁◯◯ |
| Placebo vs. Tofacitinib 3 mg BID | Low ⨁⨁◯◯ | 2.77 (0.30-25.69) | Very low ⨁◯◯◯ | 0.50 (0.00-12501.05) | Low ⨁⨁◯◯ | 2.56 (0.29-22.53) | Low ⨁⨁◯◯ |
| Placebo vs. Tofacitinib 5 mg BID | Moderate ⨁⨁⨁◯ | 0.99 (0.22-4.41) | Very low ⨁◯◯◯ | 11.29 (0.19-685.36) | Moderate ⨁⨁⨁◯ | 1.32 (0.33-5.34) | Moderate ⨁⨁⨁◯ |
| Placebo vs. Upadacitinib 15 mg QD | Low ⨁⨁◯◯ | 1.63 (0.20-13.31) | Very low ⨁◯◯◯ | 41.54 (0.20-8629.56) | Low ⨁⨁◯◯ | 2.51 (0.36-17.76) | Low ⨁⨁◯◯ |
| Placebo vs. Upadacitinib 30 mg QD | Moderate ⨁⨁⨁◯ | 0.67 (0.14-3.24) | Very low ⨁◯◯◯ | 22.05 (0.16-3096.34) | Moderate ⨁⨁⨁◯ | 0.93 (0.21-4.15) | Moderate ⨁⨁⨁◯ |
| Placebo vs. Upadacitinib 45 mg QD | Low ⨁⨁◯◯ | 2.14 (0.45-10.21) | Low ⨁⨁◯◯ | 0.24 (0.01-7.74) | Moderate ⨁⨁⨁◯ | 1.48 (0.36-6.15) | Moderate ⨁⨁⨁◯ |
| Tofacitinib 0.5 mg BID vs. Tofacitinib 10 mg BID | Low ⨁⨁◯◯ | 1.07 (0.07-17.41) | Very low ⨁◯◯◯ | 1.53 (0.02-151.77) | Low ⨁⨁◯◯ | 1.18 (0.11-12.79) | Low ⨁⨁◯◯ |
| Tofacitinib 0.5 mg BID vs. Tofacitinib 15 mg BID | Low ⨁⨁◯◯ | 1.59 (0.10-25.90) | Very low ⨁◯◯◯ | 0.11 (0.00-46.71) | Low ⨁⨁◯◯ | 1.00 (0.08-12.54) | Low ⨁⨁◯◯ |
| Tofacitinib 1 mg BID vs. Tofacitinib 15 mg BID | Very low ⨁◯◯◯ | 0.97 (0.06-15.85) | Very low ⨁◯◯◯ | 13.92 (0.03-5768.77) | Very low ⨁◯◯◯ | 1.56 (0.12-19.58) | Very low ⨁◯◯◯ |
| Tofacitinib 1 mg BID vs. Tofacitinib 5 mg BID | Very low ⨁◯◯◯ | 0.94 (0.06-15.39) | Very low ⨁◯◯◯ | 0.60 (0.00-132.70) | Very low ⨁◯◯◯ | 0.86 (0.07-10.23) | Very low ⨁◯◯◯ |
| Tofacitinib 10 mg BID vs. Tofacitinib 15 mg BID | Low ⨁⨁◯◯ | 1.49 (0.09-24.29) | Low ⨁⨁◯◯ | 0.49 (0.03-7.54) | Moderate ⨁⨁⨁◯ | 0.85 (0.12-5.95) | Moderate ⨁⨁⨁◯ |
| Tofacitinib 10 mg BID vs. Tofacitinib 3 mg BID | Low ⨁⨁◯◯ | 1.00 (0.06-16.33) | Very low ⨁◯◯◯ | 0.70 (0.01-68.76) | Moderate ⨁⨁⨁◯ | 0.91 (0.08-9.86) | Moderate ⨁⨁⨁◯ |
| Tofacitinib 10 mg BID vs. Tofacitinib 5 mg BID | Moderate ⨁⨁⨁◯ | 0.62 (0.08-5.09) | Low ⨁⨁◯◯ | 0.30 (0.02-4.09) | Moderate ⨁⨁⨁◯ | 0.47 (0.09-2.40) | Moderate ⨁⨁⨁◯ |
| Tofacitinib 15 mg BID vs. Tofacitinib 3 mg BID | Low ⨁⨁◯◯ | 0.67 (0.04-10.91) | Very low ⨁◯◯◯ | 9.67 (0.02-4090.65) | Low ⨁⨁◯◯ | 1.07 (0.09-13.48) | Low ⨁⨁◯◯ |
| Tofacitinib 15 mg BID vs. Tofacitinib 5 mg BID | Very low ⨁◯◯◯ | 0.97 (0.06-15.84) | Low ⨁⨁◯◯ | 0.33 (0.02-4.74) | Low ⨁⨁◯◯ | 0.55 (0.08-3.80) | Low ⨁⨁◯◯ |
| Upadacitinib 15 mg QD vs. Upadacitinib 30 mg QD | Moderate ⨁⨁⨁◯ | 0.45 (0.07-3.06) | Very low ⨁◯◯◯ | 0.00 (0.00-47.29) | Moderate ⨁⨁⨁◯ | 0.37 (0.06-2.44) | Moderate ⨁⨁⨁◯ |
| Upadacitinib 15 mg QD vs. Upadacitinib 45 mg QD | Low ⨁⨁◯◯ | 0.50 (0.02-10.54) | Very low ⨁◯◯◯ | 0.69 (0.04-11.90) | Moderate ⨁⨁⨁◯ | 0.59 (0.07-4.75) | Moderate ⨁⨁⨁◯ |
| Upadacitinib 30 mg QD vs. Upadacitinib 45 mg QD | Moderate ⨁⨁⨁◯ | 0.21 (0.01-4.36) | Very low ⨁◯◯◯ | 4.80 (0.52-44.73) | Moderate ⨁⨁⨁◯ | 1.60 (0.26-9.69) | Moderate ⨁⨁⨁◯ |

CVE: cardiovascular events; QD: once daily; BID: twice daily.

**Supplementary table 11-1-1 Direct, indirect, NMA results and certainty for MACE (without dose consideration).**

| Number | Comparison | Direct estimate | | | Indirect estimate | | | Network estimate | |
| --- | --- | --- | --- | --- | --- | --- | --- | --- | --- |
|  |  | Preliminary-GRADE | OR (95% CI) | Final-GRADE | Preliminary-GRADE | OR (95% CI) | Final-GRADE | OR (95% CI) | Final-GRADE |
| 1 | Deucravacitinib:Brepocitinib |  | - |  | Low | 0.63 (0-171.6) | Very low | 0.63 (0-171.6) | Very low |
| 2 | Filgotinib:Brepocitinib |  | - |  | Low | 2.3 (0.04-150.9) | Very low | 2.3 (0.04-150.9) | Very low |
| 3 | Ivarmacitinib:Brepocitinib |  | - |  | Moderate | 1.91 (0.01-501.87) | Low | 1.91 (0.01-501.87) | Low |
| 4 | Izencitinib:Brepocitinib |  | - |  | Low | 1.88 (0.02-234.53) | Very low | 1.88 (0.02-234.53) | Very low |
| 5 | Peficitinib:Brepocitinib |  | - |  | Moderate | 1.4 (0.01-367.15) | Low | 1.4 (0.01-367.15) | Low |
| 6 | Placebo:Brepocitinib | Moderate | 5.78 (0.11-297.96) | Low |  | - |  | 5.78 (0.11-297.96) | Low |
| 7 | Ritlecitinib:Brepocitinib | Moderate | 2.86 (0.12-70.75) | Low |  | - |  | 2.86 (0.12-70.75) | Low |
| 8 | Tofacitinib:Brepocitinib |  | - |  | Low | 1.77 (0.03-109.51) | Very low | 1.77 (0.03-109.51) | Very low |
| 9 | Upadacitinib:Brepocitinib |  | - |  | Low | 1.99 (0.03-132.28) | Very low | 1.99 (0.03-132.28) | Very low |
| 10 | Deucravacitinib:Filgotinib |  | - |  | Very low | 0.28 (0-18.6) | Very low | 0.28 (0-18.6) | Very low |
| 11 | Deucravacitinib:Ivarmacitinib |  | - |  | Low | 0.33 (0-89.41) | Very low | 0.33 (0-89.41) | Very low |
| 12 | Deucravacitinib:Izencitinib |  | - |  | Very low | 0.34 (0-43.21) | Very low | 0.34 (0-43.21) | Very low |
| 13 | Deucravacitinib:Peficitinib |  | - |  | Low | 0.45 (0-121.91) | Very low | 0.45 (0-121.91) | Very low |
| 14 | Deucravacitinib:Placebo | Low | 0.11 (0-5.86) | Low |  | - |  | 0.11 (0-5.86) | Low |
| 15 | Deucravacitinib:Ritlecitinib |  | - |  | Low | 0.22 (0-37.23) | Very low | 0.22 (0-37.23) | Very low |
| 16 | Deucravacitinib:Tofacitinib |  | - |  | Very low | 0.36 (0.01-22.93) | Very low | 0.36 (0.01-22.93) | Very low |
| 17 | Deucravacitinib:Upadacitinib |  | - |  | Very low | 0.32 (0-21.82) | Very low | 0.32 (0-21.82) | Very low |
| 18 | Filgotinib:Ivarmacitinib |  | - |  | Low | 1.21 (0.02-78.48) | Very low | 1.21 (0.02-78.48) | Very low |
| 19 | Filgotinib:Izencitinib |  | - |  | Low | 1.23 (0.05-27.48) | Very low | 1.23 (0.05-27.48) | Very low |
| 20 | Filgotinib:Peficitinib |  | - |  | Low | 1.65 (0.03-106.97) | Very low | 1.65 (0.03-106.97) | Very low |
| 21 | Filgotinib:Placebo | Moderate | 0.4 (0.1-1.6) | Moderate |  | - |  | 0.4 (0.1-1.6) | Moderate |
| 22 | Filgotinib:Ritlecitinib |  | - |  | Low | 0.81 (0.02-27.13) | Very low | 0.81 (0.02-27.13) | Very low |
| 23 | Filgotinib:Tofacitinib |  | - |  | Low | 1.3 (0.21-8.26) | Low | 1.3 (0.21-8.26) | Low |
| 24 | Filgotinib:Upadacitinib |  | - |  | Low | 1.16 (0.16-8.53) | Low | 1.16 (0.16-8.53) | Low |
| 25 | Ivarmacitinib:Izencitinib |  | - |  | Low | 1.02 (0.01-125.8) | Very low | 1.02 (0.01-125.8) | Very low |
| 26 | Ivarmacitinib:Peficitinib |  | - |  | Low | 1.37 (0.01-356.53) | Very low | 1.37 (0.01-356.53) | Very low |
| 27 | Ivarmacitinib:Placebo | Moderate | 0.33 (0.01-16.93) | Low |  | - |  | 0.33 (0.01-16.93) | Low |
| 28 | Ivarmacitinib:Ritlecitinib |  | - |  | Moderate | 0.67 (0-108.58) | Low | 0.67 (0-108.58) | Low |
| 29 | Ivarmacitinib:Tofacitinib |  | - |  | Low | 1.08 (0.02-66.39) | Very low | 1.08 (0.02-66.39) | Very low |
| 30 | Ivarmacitinib:Upadacitinib |  | - |  | Low | 0.96 (0.01-63.22) | Very low | 0.96 (0.01-63.22) | Very low |
| 31 | Izencitinib:Peficitinib |  | - |  | Low | 1.34 (0.01-166.45) | Very low | 1.34 (0.01-166.45) | Very low |
| 32 | Izencitinib:Placebo | Moderate | 0.33 (0.02-5.26) | Moderate |  | - |  | 0.33 (0.02-5.26) | Moderate |
| 33 | Izencitinib:Ritlecitinib |  | - |  | Low | 0.66 (0.01-46.69) | Very low | 0.66 (0.01-46.69) | Very low |
| 34 | Izencitinib:Tofacitinib |  | - |  | Low | 1.06 (0.05-22.13) | Very low | 1.06 (0.05-22.13) | Very low |
| 35 | Izencitinib:Upadacitinib |  | - |  | Low | 0.94 (0.04-21.59) | Very low | 0.94 (0.04-21.59) | Very low |
| 36 | Peficitinib:Placebo | Moderate | 0.24 (0-12.38) | Low |  | - |  | 0.24 (0-12.38) | Low |
| 37 | Peficitinib:Ritlecitinib |  | - |  | Moderate | 0.49 (0-79.43) | Low | 0.49 (0-79.43) | Low |
| 38 | Peficitinib:Tofacitinib |  | - |  | Low | 0.79 (0.01-48.55) | Very low | 0.79 (0.01-48.55) | Very low |
| 39 | Peficitinib:Upadacitinib |  | - |  | Low | 0.7 (0.01-46.23) | Very low | 0.7 (0.01-46.23) | Very low |
| 40 | Placebo:Ritlecitinib | Moderate | 2.02 (0.08-51.03) | Low |  | - |  | 2.02 (0.08-51.03) | Low |
| 41 | Placebo:Tofacitinib | Moderate | 3.27 (0.97-10.99) | Low |  | - |  | 3.27 (0.97-10.99) | Low |
| 42 | Placebo:Upadacitinib | Moderate | 2.9 (0.69-12.16) | Low |  | - |  | 2.9 (0.69-12.16) | Low |
| 43 | Ritlecitinib:Tofacitinib |  | - |  | Low | 1.62 (0.05-50.9) | Very low | 1.62 (0.05-50.9) | Very low |
| 44 | Ritlecitinib:Upadacitinib |  | - |  | Low | 1.44 (0.04-49.11) | Very low | 1.44 (0.04-49.11) | Very low |
| 45 | Tofacitinib:Upadacitinib |  | - |  | Moderate | 0.89 (0.14-5.81) | Moderate | 0.89 (0.14-5.81) | Moderate |

NMA: network meta-analysis; MACE: major adverse cardiovascular events; GRADE: Grading of Recommendations, Assessment, Development and Evaluation; OR: odds ratio; CI: confidence interval.

**Supplementary table 11-1-2 Direct, indirect, NMA results and certainty for VTE (without dose consideration).**

| Number | Comparison | Direct estimate | | | Indirect estimate | | | Network estimate | |
| --- | --- | --- | --- | --- | --- | --- | --- | --- | --- |
|  |  | Preliminary-GRADE | OR (95% CI) | Final-GRADE | Preliminary-GRADE | OR (95% CI) | Final-GRADE | OR (95% CI) | Final-GRADE |
| 1 | Deucravacitinib:Brepocitinib |  | - |  | Low | 0.19 (0-31.89) | Very low | 0.19 (0-31.89) | Very low |
| 2 | Filgotinib:Brepocitinib |  | - |  | Low | 2.73 (0.04-176.36) | Very low | 2.73 (0.04-176.36) | Very low |
| 3 | Ivarmacitinib:Brepocitinib |  | - |  | Moderate | 1.91 (0.01-501.87) | Low | 1.91 (0.01-501.87) | Low |
| 4 | Izencitinib:Brepocitinib |  | - |  | Low | 1.88 (0.02-234.53) | Very low | 1.88 (0.02-234.53) | Very low |
| 5 | Peficitinib:Brepocitinib |  | - |  | Moderate | 1.4 (0.01-367.15) | Low | 1.4 (0.01-367.15) | Low |
| 6 | Placebo:Brepocitinib | Moderate | 5.78 (0.11-297.96) | Low |  | - |  | 5.78 (0.11-297.96) | Low |
| 7 | Ritlecitinib:Brepocitinib | Moderate | 0.95 (0.02-48.02) | Low |  | - |  | 0.95 (0.02-48.02) | Low |
| 8 | Tofacitinib:Brepocitinib |  | - |  | Low | 1.28 (0.02-78.97) | Very low | 1.28 (0.02-78.97) | Very low |
| 9 | Upadacitinib:Brepocitinib |  | - |  | Low | 3.73 (0.06-235.52) | Very low | 3.73 (0.06-235.52) | Very low |
| 10 | Deucravacitinib:Filgotinib |  | - |  | Very low | 0.07 (0-2.4) | Very low | 0.07 (0-2.4) | Very low |
| 11 | Deucravacitinib:Ivarmacitinib |  | - |  | Low | 0.1 (0-16.61) | Very low | 0.1 (0-16.61) | Very low |
| 12 | Deucravacitinib:Izencitinib |  | - |  | Very low | 0.1 (0-7.4) | Very low | 0.1 (0-7.4) | Very low |
| 13 | Deucravacitinib:Peficitinib |  | - |  | Low | 0.14 (0-22.64) | Very low | 0.14 (0-22.64) | Very low |
| 14 | Deucravacitinib:Placebo | Low | 0.03 (0-0.87) | Low |  | - |  | 0.03 (0-0.87) | Low |
| 15 | Deucravacitinib:Ritlecitinib |  | - |  | Low | 0.2 (0-33.69) | Very low | 0.2 (0-33.69) | Very low |
| 16 | Deucravacitinib:Tofacitinib |  | - |  | Very low | 0.15 (0-4.89) | Very low | 0.15 (0-4.89) | Very low |
| 17 | Deucravacitinib:Upadacitinib |  | - |  | Very low | 0.05 (0-1.71) | Very low | 0.05 (0-1.71) | Very low |
| 18 | Filgotinib:Ivarmacitinib |  | - |  | Low | 1.43 (0.02-91.72) | Very low | 1.43 (0.02-91.72) | Very low |
| 19 | Filgotinib:Izencitinib |  | - |  | Low | 1.45 (0.07-31.96) | Very low | 1.45 (0.07-31.96) | Very low |
| 20 | Filgotinib:Peficitinib |  | - |  | Low | 1.95 (0.03-125.01) | Very low | 1.95 (0.03-125.01) | Very low |
| 21 | Filgotinib:Placebo | Moderate | 0.47 (0.12-1.82) | Moderate |  | - |  | 0.47 (0.12-1.82) | Moderate |
| 22 | Filgotinib:Ritlecitinib |  | - |  | Low | 2.89 (0.04-186.29) | Very low | 2.89 (0.04-186.29) | Very low |
| 23 | Filgotinib:Tofacitinib |  | - |  | Low | 2.14 (0.35-13.15) | Very low | 2.14 (0.35-13.15) | Very low |
| 24 | Filgotinib:Upadacitinib |  | - |  | Low | 0.73 (0.11-4.68) | Low | 0.73 (0.11-4.68) | Low |
| 25 | Ivarmacitinib:Izencitinib |  | - |  | Low | 1.02 (0.01-125.8) | Very low | 1.02 (0.01-125.8) | Very low |
| 26 | Ivarmacitinib:Peficitinib |  | - |  | Low | 1.37 (0.01-356.53) | Very low | 1.37 (0.01-356.53) | Very low |
| 27 | Ivarmacitinib:Placebo | Moderate | 0.33 (0.01-16.93) | Low |  | - |  | 0.33 (0.01-16.93) | Low |
| 28 | Ivarmacitinib:Ritlecitinib |  | - |  | Moderate | 2.02 (0.01-530.17) | Low | 2.02 (0.01-530.17) | Low |
| 29 | Ivarmacitinib:Tofacitinib |  | - |  | Low | 1.5 (0.02-92.06) | Very low | 1.5 (0.02-92.06) | Very low |
| 30 | Ivarmacitinib:Upadacitinib |  | - |  | Low | 0.51 (0.01-32.02) | Very low | 0.51 (0.01-32.02) | Very low |
| 31 | Izencitinib:Peficitinib |  | - |  | Low | 1.34 (0.01-166.45) | Very low | 1.34 (0.01-166.45) | Very low |
| 32 | Izencitinib:Placebo | Moderate | 0.33 (0.02-5.26) | Moderate |  | - |  | 0.33 (0.02-5.26) | Moderate |
| 33 | Izencitinib:Ritlecitinib |  | - |  | Low | 1.99 (0.02-247.76) | Very low | 1.99 (0.02-247.76) | Very low |
| 34 | Izencitinib:Tofacitinib |  | - |  | Low | 1.47 (0.07-30.68) | Very low | 1.47 (0.07-30.68) | Very low |
| 35 | Izencitinib:Upadacitinib |  | - |  | Low | 0.5 (0.02-10.74) | Very low | 0.5 (0.02-10.74) | Very low |
| 36 | Peficitinib:Placebo | Moderate | 0.24 (0-12.38) | Low |  | - |  | 0.24 (0-12.38) | Low |
| 37 | Peficitinib:Ritlecitinib |  | - |  | Moderate | 1.48 (0.01-387.85) | Low | 1.48 (0.01-387.85) | Low |
| 38 | Peficitinib:Tofacitinib |  | - |  | Low | 1.1 (0.02-67.32) | Very low | 1.1 (0.02-67.32) | Very low |
| 39 | Peficitinib:Upadacitinib |  | - |  | Low | 0.37 (0.01-23.42) | Very low | 0.37 (0.01-23.42) | Very low |
| 40 | Placebo:Ritlecitinib | Moderate | 6.1 (0.12-314.74) | Low |  | - |  | 6.1 (0.12-314.74) | Low |
| 41 | Placebo:Tofacitinib | Moderate | 4.53 (1.35-15.24) | Low |  | - |  | 4.53 (1.35-15.24) | Low |
| 42 | Placebo:Upadacitinib | Moderate | 1.55 (0.43-5.53) | Moderate |  | - |  | 1.55 (0.43-5.53) | Moderate |
| 43 | Ritlecitinib:Tofacitinib |  | - |  | Low | 0.74 (0.01-45.94) | Very low | 0.74 (0.01-45.94) | Very low |
| 44 | Ritlecitinib:Upadacitinib |  | - |  | Low | 0.25 (0-15.98) | Very low | 0.25 (0-15.98) | Very low |
| 45 | Tofacitinib:Upadacitinib |  | - |  | Moderate | 0.34 (0.06-1.98) | Moderate | 0.34 (0.06-1.98) | Moderate |

NMA: network meta-analysis; VTE: venous thromboembolism events; GRADE: Grading of Recommendations, Assessment, Development and Evaluation; OR: odds ratio; CI: confidence interval.

**Supplementary table 11-1-3 Direct, indirect, NMA results and certainty for CVE (without dose consideration).**

| Number | Comparison | Direct estimate | | | Indirect estimate | | | Network estimate | |
| --- | --- | --- | --- | --- | --- | --- | --- | --- | --- |
|  |  | Preliminary-GRADE | OR (95% CI) | Final-GRADE | Preliminary-GRADE | OR (95% CI) | Final-GRADE | OR (95% CI) | Final-GRADE |
| 1 | Deucravacitinib:Brepocitinib |  | - |  | Low | 0.19 (0-31.89) | Very low | 0.19 (0-31.89) | Very low |
| 2 | Filgotinib:Brepocitinib |  | - |  | Low | 2.73 (0.04-176.36) | Very low | 2.73 (0.04-176.36) | Very low |
| 3 | Ivarmacitinib:Brepocitinib |  | - |  | Moderate | 1.91 (0.01-501.87) | Low | 1.91 (0.01-501.87) | Low |
| 4 | Izencitinib:Brepocitinib |  | - |  | Low | 1.88 (0.02-234.53) | Very low | 1.88 (0.02-234.53) | Very low |
| 5 | Peficitinib:Brepocitinib |  | - |  | Moderate | 1.4 (0.01-367.15) | Low | 1.4 (0.01-367.15) | Low |
| 6 | Placebo:Brepocitinib | Moderate | 5.78 (0.11-297.96) | Low |  | - |  | 5.78 (0.11-297.96) | Low |
| 7 | Ritlecitinib:Brepocitinib | Moderate | 2.86 (0.12-70.75) | Low |  | - |  | 2.86 (0.12-70.75) | Low |
| 8 | Tofacitinib:Brepocitinib |  | - |  | Low | 1.32 (0.02-79.06) | Very low | 1.32 (0.02-79.06) | Very low |
| 9 | Upadacitinib:Brepocitinib |  | - |  | Low | 3.32 (0.05-202.33) | Very low | 3.32 (0.05-202.33) | Very low |
| 10 | Deucravacitinib:Filgotinib |  | - |  | Very low | 0.07 (0-2.4) | Very low | 0.07 (0-2.4) | Very low |
| 11 | Deucravacitinib:Ivarmacitinib |  | - |  | Low | 0.1 (0-16.61) | Very low | 0.1 (0-16.61) | Very low |
| 12 | Deucravacitinib:Izencitinib |  | - |  | Very low | 0.1 (0-7.4) | Very low | 0.1 (0-7.4) | Very low |
| 13 | Deucravacitinib:Peficitinib |  | - |  | Low | 0.14 (0-22.64) | Very low | 0.14 (0-22.64) | Very low |
| 14 | Deucravacitinib:Placebo | Low | 0.03 (0-0.87) | Low |  | - |  | 0.03 (0-0.87) | Low |
| 15 | Deucravacitinib:Ritlecitinib |  | - |  | Low | 0.07 (0-6.59) | Low | 0.07 (0-6.59) | Low |
| 16 | Deucravacitinib:Tofacitinib |  | - |  | Very low | 0.14 (0-4.57) | Very low | 0.14 (0-4.57) | Very low |
| 17 | Deucravacitinib:Upadacitinib |  | - |  | Very low | 0.06 (0-1.84) | Very low | 0.06 (0-1.84) | Very low |
| 18 | Filgotinib:Ivarmacitinib |  | - |  | Low | 1.43 (0.02-91.72) | Very low | 1.43 (0.02-91.72) | Very low |
| 19 | Filgotinib:Izencitinib |  | - |  | Low | 1.45 (0.07-31.96) | Very low | 1.45 (0.07-31.96) | Very low |
| 20 | Filgotinib:Peficitinib |  | - |  | Low | 1.95 (0.03-125.01) | Very low | 1.95 (0.03-125.01) | Very low |
| 21 | Filgotinib:Placebo | Moderate | 0.47 (0.12-1.82) | Moderate |  | - |  | 0.47 (0.12-1.82) | Moderate |
| 22 | Filgotinib:Ritlecitinib |  | - |  | Low | 0.96 (0.03-31.63) | Very low | 0.96 (0.03-31.63) | Very low |
| 23 | Filgotinib:Tofacitinib |  | - |  | Low | 2.07 (0.36-11.87) | Very low | 2.07 (0.36-11.87) | Very low |
| 24 | Filgotinib:Upadacitinib |  | - |  | Low | 0.82 (0.14-4.86) | Low | 0.82 (0.14-4.86) | Low |
| 25 | Ivarmacitinib:Izencitinib |  | - |  | Low | 1.02 (0.01-125.8) | Very low | 1.02 (0.01-125.8) | Very low |
| 26 | Ivarmacitinib:Peficitinib |  | - |  | Low | 1.37 (0.01-356.53) | Very low | 1.37 (0.01-356.53) | Very low |
| 27 | Ivarmacitinib:Placebo | Moderate | 0.33 (0.01-16.93) | Low |  | - |  | 0.33 (0.01-16.93) | Low |
| 28 | Ivarmacitinib:Ritlecitinib |  | - |  | Moderate | 0.67 (0-108.58) | Low | 0.67 (0-108.58) | Low |
| 29 | Ivarmacitinib:Tofacitinib |  | - |  | Low | 1.45 (0.02-86.5) | Very low | 1.45 (0.02-86.5) | Very low |
| 30 | Ivarmacitinib:Upadacitinib |  | - |  | Low | 0.57 (0.01-34.74) | Very low | 0.57 (0.01-34.74) | Very low |
| 31 | Izencitinib:Peficitinib |  | - |  | Low | 1.34 (0.01-166.45) | Very low | 1.34 (0.01-166.45) | Very low |
| 32 | Izencitinib:Placebo | Moderate | 0.33 (0.02-5.26) | Moderate |  | - |  | 0.33 (0.02-5.26) | Moderate |
| 33 | Izencitinib:Ritlecitinib |  | - |  | Low | 0.66 (0.01-46.69) | Very low | 0.66 (0.01-46.69) | Very low |
| 34 | Izencitinib:Tofacitinib |  | - |  | Low | 1.43 (0.07-28.51) | Very low | 1.43 (0.07-28.51) | Very low |
| 35 | Izencitinib:Upadacitinib |  | - |  | Low | 0.57 (0.03-11.51) | Very low | 0.57 (0.03-11.51) | Very low |
| 36 | Peficitinib:Placebo | Moderate | 0.24 (0-12.38) | Low |  | - |  | 0.24 (0-12.38) | Low |
| 37 | Peficitinib:Ritlecitinib |  | - |  | Moderate | 0.49 (0-79.43) | Low | 0.49 (0-79.43) | Low |
| 38 | Peficitinib:Tofacitinib |  | - |  | Low | 1.06 (0.02-63.25) | Very low | 1.06 (0.02-63.25) | Very low |
| 39 | Peficitinib:Upadacitinib |  | - |  | Low | 0.42 (0.01-25.4) | Very low | 0.42 (0.01-25.4) | Very low |
| 40 | Placebo:Ritlecitinib | Moderate | 2.02 (0.08-51.03) | Low |  | - |  | 2.02 (0.08-51.03) | Low |
| 41 | Placebo:Tofacitinib | Moderate | 4.39 (1.45-13.25) | Low |  | - |  | 4.39 (1.45-13.25) | Low |
| 42 | Placebo:Upadacitinib | Moderate | 1.74 (0.55-5.52) | Moderate |  | - |  | 1.74 (0.55-5.52) | Moderate |
| 43 | Ritlecitinib:Tofacitinib |  | - |  | Low | 2.17 (0.07-65.92) | Very low | 2.17 (0.07-65.92) | Very low |
| 44 | Ritlecitinib:Upadacitinib |  | - |  | Low | 0.86 (0.03-26.55) | Very low | 0.86 (0.03-26.55) | Very low |
| 45 | Tofacitinib:Upadacitinib |  | - |  | Moderate | 0.4 (0.08-1.96) | Moderate | 0.4 (0.08-1.96) | Moderate |

NMA: network meta-analysis; CVE: cardiovascular events; GRADE: Grading of Recommendations, Assessment, Development and Evaluation; OR: odds ratio; CI: confidence interval.

**Supplementary table 11-2-1 Direct, indirect, NMA results and certainty for MACE (with dose consideration).**

| Number | Comparison | Direct estimate | | | Indirect estimate | | | Network estimate | |
| --- | --- | --- | --- | --- | --- | --- | --- | --- | --- |
|  |  | Preliminary-GRADE | OR (95% CI) | Final-GRADE | Preliminary-GRADE | OR (95% CI) | Final-GRADE | OR (95% CI) | Final-GRADE |
| 1 | Deucravacitinib:Brepocitinib |  | - |  | Very low | 0.63 (0-171.6) | Very low | 0.63 (0-171.6) | Very low |
| 2 | Filgotinib 100 mg QD:Brepocitinib |  | - |  | Very low | 3.65 (0.05-253.54) | Very low | 3.65 (0.05-253.54) | Very low |
| 3 | Filgotinib 200 mg QD:Brepocitinib |  | - |  | Very low | 4.14 (0.06-271.19) | Very low | 4.14 (0.06-271.19) | Very low |
| 4 | Ivarmacitinib:Brepocitinib |  | - |  | Very low | 1.91 (0.01-501.87) | Very low | 1.91 (0.01-501.87) | Very low |
| 5 | Izencitinib:Brepocitinib |  | - |  | Very low | 1.88 (0.02-234.53) | Very low | 1.88 (0.02-234.53) | Very low |
| 6 | Peficitinib:Brepocitinib |  | - |  | Low | 1.4 (0.01-367.15) | Very low | 1.4 (0.01-367.15) | Very low |
| 7 | Placebo:Brepocitinib | Low | 5.78 (0.11-297.96) | Very low |  | - |  | 5.78 (0.11-297.96) | Very low |
| 8 | Ritlecitinib:Brepocitinib | Low | 2.86 (0.12-70.75) | Very low |  | - |  | 2.86 (0.12-70.75) | Very low |
| 9 | Tofacitinib 0.5 mg BID:Brepocitinib |  | - |  | Low | 3.35 (0.04-309.1) | Very low | 3.35 (0.04-309.1) | Very low |
| 10 | Tofacitinib 1 mg BID:Brepocitinib |  | - |  | Very low | 4.56 (0.04-468.8) | Very low | 4.56 (0.04-468.8) | Very low |
| 11 | Tofacitinib 10 mg BID:Brepocitinib |  | - |  | Very low | 3.12 (0.05-202.03) | Very low | 3.12 (0.05-202.03) | Very low |
| 12 | Tofacitinib 15 mg BID:Brepocitinib |  | - |  | Very low | 3.14 (0.04-229.34) | Very low | 3.14 (0.04-229.34) | Very low |
| 13 | Tofacitinib 3 mg BID:Brepocitinib |  | - |  | Low | 3.15 (0.03-289.96) | Very low | 3.15 (0.03-289.96) | Very low |
| 14 | Tofacitinib 5 mg BID:Brepocitinib |  | - |  | Very low | 6.04 (0.09-414.86) | Very low | 6.04 (0.09-414.86) | Very low |
| 15 | Upadacitinib 15 mg QD:Brepocitinib |  | - |  | Very low | 3.68 (0.04-308.7) | Very low | 3.68 (0.04-308.7) | Very low |
| 16 | Upadacitinib 30 mg QD:Brepocitinib |  | - |  | Very low | 2.68 (0.03-224.87) | Very low | 2.68 (0.03-224.87) | Very low |
| 17 | Upadacitinib 45 mg QD:Brepocitinib |  | - |  | Very low | 2.64 (0.04-193.4) | Very low | 2.64 (0.04-193.4) | Very low |
| 18 | Deucravacitinib:Filgotinib 100 mg QD |  | - |  | Very low | 0.17 (0-12.42) | Very low | 0.17 (0-12.42) | Very low |
| 19 | Deucravacitinib:Filgotinib 200 mg QD |  | - |  | Very low | 0.15 (0-10.36) | Very low | 0.15 (0-10.36) | Very low |
| 20 | Deucravacitinib:Ivarmacitinib |  | - |  | Very low | 0.33 (0-89.41) | Very low | 0.33 (0-89.41) | Very low |
| 21 | Deucravacitinib:Izencitinib |  | - |  | Very low | 0.34 (0-43.21) | Very low | 0.34 (0-43.21) | Very low |
| 22 | Deucravacitinib:Peficitinib |  | - |  | Very low | 0.45 (0-121.91) | Very low | 0.45 (0-121.91) | Very low |
| 23 | Deucravacitinib:Placebo | Very low | 0.11 (0-5.86) | Very low |  | - |  | 0.11 (0-5.86) | Very low |
| 24 | Deucravacitinib:Ritlecitinib |  | - |  | Very low | 0.22 (0-37.23) | Very low | 0.22 (0-37.23) | Very low |
| 25 | Deucravacitinib:Tofacitinib 0.5 mg BID |  | - |  | Very low | 0.19 (0-17.97) | Very low | 0.19 (0-17.97) | Very low |
| 26 | Deucravacitinib:Tofacitinib 1 mg BID |  | - |  | Very low | 0.14 (0-14.7) | Very low | 0.14 (0-14.7) | Very low |
| 27 | Deucravacitinib:Tofacitinib 10 mg BID |  | - |  | Very low | 0.2 (0-13.62) | Very low | 0.2 (0-13.62) | Very low |
| 28 | Deucravacitinib:Tofacitinib 15 mg BID |  | - |  | Very low | 0.2 (0-15.19) | Very low | 0.2 (0-15.19) | Very low |
| 29 | Deucravacitinib:Tofacitinib 3 mg BID |  | - |  | Very low | 0.2 (0-19.14) | Very low | 0.2 (0-19.14) | Very low |
| 30 | Deucravacitinib:Tofacitinib 5 mg BID |  | - |  | Very low | 0.1 (0-7.43) | Very low | 0.1 (0-7.43) | Very low |
| 31 | Deucravacitinib:Upadacitinib 15 mg QD |  | - |  | Very low | 0.17 (0-14.9) | Very low | 0.17 (0-14.9) | Very low |
| 32 | Deucravacitinib:Upadacitinib 30 mg QD |  | - |  | Very low | 0.24 (0-20.43) | Very low | 0.24 (0-20.43) | Very low |
| 33 | Deucravacitinib:Upadacitinib 45 mg QD |  | - |  | Very low | 0.24 (0-18.17) | Very low | 0.24 (0-18.17) | Very low |
| 34 | Filgotinib 100 mg QD:Filgotinib 200 mg QD | Low | 0.85 (0.17-4.25) | Low | Low | 1.47 (0-738.34) | Very low | 0.88 (0.19-4.18) | Low |
| 35 | Filgotinib 100 mg QD:Ivarmacitinib |  | - |  | Very low | 1.91 (0.03-131.88) | Very low | 1.91 (0.03-131.88) | Very low |
| 36 | Filgotinib 100 mg QD:Izencitinib |  | - |  | Very low | 1.94 (0.08-47.08) | Very low | 1.94 (0.08-47.08) | Very low |
| 37 | Filgotinib 100 mg QD:Peficitinib |  | - |  | Very low | 2.61 (0.04-179.75) | Very low | 2.61 (0.04-179.75) | Very low |
| 38 | Filgotinib 100 mg QD:Placebo | Low | 0.65 (0.13-3.27) | Low | Very low | 0.38 (0-188.29) | Very low | 0.63 (0.13-3) | Low |
| 39 | Filgotinib 100 mg QD:Ritlecitinib |  | - |  | Very low | 1.28 (0.04-46.08) | Very low | 1.28 (0.04-46.08) | Very low |
| 40 | Filgotinib 100 mg QD:Tofacitinib 0.5 mg BID |  | - |  | Very low | 1.09 (0.07-16.38) | Very low | 1.09 (0.07-16.38) | Very low |
| 41 | Filgotinib 100 mg QD:Tofacitinib 1 mg BID |  | - |  | Very low | 0.8 (0.04-14.37) | Very low | 0.8 (0.04-14.37) | Very low |
| 42 | Filgotinib 100 mg QD:Tofacitinib 10 mg BID |  | - |  | Very low | 1.17 (0.15-9.28) | Very low | 1.17 (0.15-9.28) | Very low |
| 43 | Filgotinib 100 mg QD:Tofacitinib 15 mg BID |  | - |  | Very low | 1.16 (0.12-11.57) | Very low | 1.16 (0.12-11.57) | Very low |
| 44 | Filgotinib 100 mg QD:Tofacitinib 3 mg BID |  | - |  | Very low | 1.16 (0.08-17.44) | Very low | 1.16 (0.08-17.44) | Very low |
| 45 | Filgotinib 100 mg QD:Tofacitinib 5 mg BID |  | - |  | Very low | 0.6 (0.07-5.36) | Very low | 0.6 (0.07-5.36) | Very low |
| 46 | Filgotinib 100 mg QD:Upadacitinib 15 mg QD |  | - |  | Very low | 0.99 (0.08-12.7) | Very low | 0.99 (0.08-12.7) | Very low |
| 47 | Filgotinib 100 mg QD:Upadacitinib 30 mg QD |  | - |  | Very low | 1.36 (0.11-17.41) | Very low | 1.36 (0.11-17.41) | Very low |
| 48 | Filgotinib 100 mg QD:Upadacitinib 45 mg QD |  | - |  | Very low | 1.39 (0.14-13.9) | Very low | 1.39 (0.14-13.9) | Very low |
| 49 | Filgotinib 200 mg QD:Ivarmacitinib |  | - |  | Very low | 2.17 (0.03-141.05) | Very low | 2.17 (0.03-141.05) | Very low |
| 50 | Filgotinib 200 mg QD:Izencitinib |  | - |  | Very low | 2.2 (0.1-49.39) | Very low | 2.2 (0.1-49.39) | Very low |
| 51 | Filgotinib 200 mg QD:Peficitinib |  | - |  | Very low | 2.96 (0.05-192.24) | Very low | 2.96 (0.05-192.24) | Very low |
| 52 | Filgotinib 200 mg QD:Placebo | Low | 0.72 (0.18-2.89) | Low |  | - |  | 0.72 (0.18-2.89) | Low |
| 53 | Filgotinib 200 mg QD:Ritlecitinib |  | - |  | Very low | 1.45 (0.04-48.77) | Very low | 1.45 (0.04-48.77) | Very low |
| 54 | Filgotinib 200 mg QD:Tofacitinib 0.5 mg BID |  | - |  | Very low | 1.24 (0.09-16.95) | Very low | 1.24 (0.09-16.95) | Very low |
| 55 | Filgotinib 200 mg QD:Tofacitinib 1 mg BID |  | - |  | Very low | 0.91 (0.06-14.95) | Very low | 0.91 (0.06-14.95) | Very low |
| 56 | Filgotinib 200 mg QD:Tofacitinib 10 mg BID |  | - |  | Very low | 1.33 (0.19-9.32) | Very low | 1.33 (0.19-9.32) | Very low |
| 57 | Filgotinib 200 mg QD:Tofacitinib 15 mg BID |  | - |  | Very low | 1.32 (0.15-11.77) | Very low | 1.32 (0.15-11.77) | Very low |
| 58 | Filgotinib 200 mg QD:Tofacitinib 3 mg BID |  | - |  | Very low | 1.32 (0.1-18.04) | Very low | 1.32 (0.1-18.04) | Very low |
| 59 | Filgotinib 200 mg QD:Tofacitinib 5 mg BID |  | - |  | Very low | 0.69 (0.09-5.42) | Very low | 0.69 (0.09-5.42) | Very low |
| 60 | Filgotinib 200 mg QD:Upadacitinib 15 mg QD |  | - |  | Very low | 1.13 (0.1-13.07) | Very low | 1.13 (0.1-13.07) | Very low |
| 61 | Filgotinib 200 mg QD:Upadacitinib 30 mg QD |  | - |  | Very low | 1.54 (0.13-17.91) | Very low | 1.54 (0.13-17.91) | Very low |
| 62 | Filgotinib 200 mg QD:Upadacitinib 45 mg QD |  | - |  | Very low | 1.57 (0.17-14.14) | Very low | 1.57 (0.17-14.14) | Very low |
| 63 | Ivarmacitinib:Izencitinib |  | - |  | Very low | 1.02 (0.01-125.8) | Very low | 1.02 (0.01-125.8) | Very low |
| 64 | Ivarmacitinib:Peficitinib |  | - |  | Low | 1.37 (0.01-356.53) | Very low | 1.37 (0.01-356.53) | Very low |
| 65 | Ivarmacitinib:Placebo | Low | 0.33 (0.01-16.93) | Very low |  | - |  | 0.33 (0.01-16.93) | Very low |
| 66 | Ivarmacitinib:Ritlecitinib |  | - |  | Low | 0.67 (0-108.58) | Very low | 0.67 (0-108.58) | Very low |
| 67 | Ivarmacitinib:Tofacitinib 0.5 mg BID |  | - |  | Low | 0.57 (0.01-52.2) | Very low | 0.57 (0.01-52.2) | Very low |
| 68 | Ivarmacitinib:Tofacitinib 1 mg BID |  | - |  | Very low | 0.42 (0-42.75) | Very low | 0.42 (0-42.75) | Very low |
| 69 | Ivarmacitinib:Tofacitinib 10 mg BID |  | - |  | Very low | 0.61 (0.01-39.46) | Very low | 0.61 (0.01-39.46) | Very low |
| 70 | Ivarmacitinib:Tofacitinib 15 mg BID |  | - |  | Very low | 0.61 (0.01-44.04) | Very low | 0.61 (0.01-44.04) | Very low |
| 71 | Ivarmacitinib:Tofacitinib 3 mg BID |  | - |  | Low | 0.61 (0.01-55.6) | Very low | 0.61 (0.01-55.6) | Very low |
| 72 | Ivarmacitinib:Tofacitinib 5 mg BID |  | - |  | Very low | 0.32 (0-21.55) | Very low | 0.32 (0-21.55) | Very low |
| 73 | Ivarmacitinib:Upadacitinib 15 mg QD |  | - |  | Very low | 0.52 (0.01-43.26) | Very low | 0.52 (0.01-43.26) | Very low |
| 74 | Ivarmacitinib:Upadacitinib 30 mg QD |  | - |  | Very low | 0.71 (0.01-59.32) | Very low | 0.71 (0.01-59.32) | Very low |
| 75 | Ivarmacitinib:Upadacitinib 45 mg QD |  | - |  | Very low | 0.72 (0.01-52.7) | Very low | 0.72 (0.01-52.7) | Very low |
| 76 | Izencitinib:Peficitinib |  | - |  | Very low | 1.34 (0.01-166.45) | Very low | 1.34 (0.01-166.45) | Very low |
| 77 | Izencitinib:Placebo | Low | 0.33 (0.02-5.26) | Low |  | - |  | 0.33 (0.02-5.26) | Low |
| 78 | Izencitinib:Ritlecitinib |  | - |  | Very low | 0.66 (0.01-46.69) | Very low | 0.66 (0.01-46.69) | Very low |
| 79 | Izencitinib:Tofacitinib 0.5 mg BID |  | - |  | Very low | 0.56 (0.02-19.68) | Very low | 0.56 (0.02-19.68) | Very low |
| 80 | Izencitinib:Tofacitinib 1 mg BID |  | - |  | Very low | 0.41 (0.01-16.59) | Very low | 0.41 (0.01-16.59) | Very low |
| 81 | Izencitinib:Tofacitinib 10 mg BID |  | - |  | Very low | 0.6 (0.03-13.37) | Very low | 0.6 (0.03-13.37) | Very low |
| 82 | Izencitinib:Tofacitinib 15 mg BID |  | - |  | Very low | 0.6 (0.02-15.51) | Very low | 0.6 (0.02-15.51) | Very low |
| 83 | Izencitinib:Tofacitinib 3 mg BID |  | - |  | Very low | 0.6 (0.02-20.96) | Very low | 0.6 (0.02-20.96) | Very low |
| 84 | Izencitinib:Tofacitinib 5 mg BID |  | - |  | Very low | 0.31 (0.01-7.44) | Very low | 0.31 (0.01-7.44) | Very low |
| 85 | Izencitinib:Upadacitinib 15 mg QD |  | - |  | Very low | 0.51 (0.02-15.89) | Very low | 0.51 (0.02-15.89) | Very low |
| 86 | Izencitinib:Upadacitinib 30 mg QD |  | - |  | Very low | 0.7 (0.02-21.78) | Very low | 0.7 (0.02-21.78) | Very low |
| 87 | Izencitinib:Upadacitinib 45 mg QD |  | - |  | Very low | 0.71 (0.03-18.59) | Very low | 0.71 (0.03-18.59) | Very low |
| 88 | Peficitinib:Placebo | Low | 0.24 (0-12.38) | Very low |  | - |  | 0.24 (0-12.38) | Very low |
| 89 | Peficitinib:Ritlecitinib |  | - |  | Low | 0.49 (0-79.43) | Very low | 0.49 (0-79.43) | Very low |
| 90 | Peficitinib:Tofacitinib 0.5 mg BID |  | - |  | Low | 0.42 (0-38.17) | Very low | 0.42 (0-38.17) | Very low |
| 91 | Peficitinib:Tofacitinib 1 mg BID |  | - |  | Very low | 0.31 (0-31.27) | Very low | 0.31 (0-31.27) | Very low |
| 92 | Peficitinib:Tofacitinib 10 mg BID |  | - |  | Very low | 0.45 (0.01-28.86) | Very low | 0.45 (0.01-28.86) | Very low |
| 93 | Peficitinib:Tofacitinib 15 mg BID |  | - |  | Very low | 0.45 (0.01-32.2) | Very low | 0.45 (0.01-32.2) | Very low |
| 94 | Peficitinib:Tofacitinib 3 mg BID |  | - |  | Low | 0.44 (0-40.66) | Very low | 0.44 (0-40.66) | Very low |
| 95 | Peficitinib:Tofacitinib 5 mg BID |  | - |  | Very low | 0.23 (0-15.76) | Very low | 0.23 (0-15.76) | Very low |
| 96 | Peficitinib:Upadacitinib 15 mg QD |  | - |  | Very low | 0.38 (0-31.64) | Very low | 0.38 (0-31.64) | Very low |
| 97 | Peficitinib:Upadacitinib 30 mg QD |  | - |  | Very low | 0.52 (0.01-43.38) | Very low | 0.52 (0.01-43.38) | Very low |
| 98 | Peficitinib:Upadacitinib 45 mg QD |  | - |  | Very low | 0.53 (0.01-38.54) | Very low | 0.53 (0.01-38.54) | Very low |
| 99 | Placebo:Ritlecitinib | Low | 2.02 (0.08-51.03) | Very low |  | - |  | 2.02 (0.08-51.03) | Very low |
| 100 | Placebo:Tofacitinib 0.5 mg BID | Low | 1.97 (0.2-19.36) | Very low | Very low | 0.21 (0-1914.59) | Very low | 1.72 (0.19-15.83) | Very low |
| 101 | Placebo:Tofacitinib 1 mg BID | Very low | 1.06 (0.06-17.28) | Very low | Very low | 2.21 (0.02-310.98) | Very low | 1.27 (0.11-14.4) | Very low |
| 102 | Placebo:Tofacitinib 10 mg BID | Low | 1.84 (0.46-7.43) | Low | Very low | 2.07 (0-1403.87) | Very low | 1.85 (0.47-7.24) | Low |
| 103 | Placebo:Tofacitinib 15 mg BID | Low | 2.01 (0.34-11.73) | Very low | Very low | 0.72 (0-235.51) | Very low | 1.84 (0.34-9.96) | Low |
| 104 | Placebo:Tofacitinib 3 mg BID | Low | 2.1 (0.21-20.61) | Very low | Very low | 0.22 (0-2029.33) | Very low | 1.84 (0.2-16.85) | Very low |
| 105 | Placebo:Tofacitinib 5 mg BID | Low | 0.73 (0.14-3.93) | Low | Very low | 3.43 (0.09-137.22) | Very low | 0.96 (0.21-4.41) | Low |
| 106 | Placebo:Upadacitinib 15 mg QD | Low | 1.63 (0.2-13.31) | Very low | Very low | 1.03 (0-1419.71) | Very low | 1.57 (0.21-11.81) | Very low |
| 107 | Placebo:Upadacitinib 30 mg QD | Low | 2.15 (0.26-17.53) | Very low | Very low | 2.25 (0-3047.39) | Very low | 2.15 (0.29-16.18) | Very low |
| 108 | Placebo:Upadacitinib 45 mg QD | Low | 2.1 (0.36-12.15) | Very low | Very low | 4.16 (0-4000.37) | Very low | 2.19 (0.4-12) | Very low |
| 109 | Ritlecitinib:Tofacitinib 0.5 mg BID |  | - |  | Low | 0.85 (0.02-42.86) | Very low | 0.85 (0.02-42.86) | Very low |
| 110 | Ritlecitinib:Tofacitinib 1 mg BID |  | - |  | Very low | 0.63 (0.01-35.68) | Very low | 0.63 (0.01-35.68) | Very low |
| 111 | Ritlecitinib:Tofacitinib 10 mg BID |  | - |  | Very low | 0.92 (0.03-30.52) | Very low | 0.92 (0.03-30.52) | Very low |
| 112 | Ritlecitinib:Tofacitinib 15 mg BID |  | - |  | Very low | 0.91 (0.02-34.8) | Very low | 0.91 (0.02-34.8) | Very low |
| 113 | Ritlecitinib:Tofacitinib 3 mg BID |  | - |  | Low | 0.91 (0.02-45.65) | Very low | 0.91 (0.02-45.65) | Very low |
| 114 | Ritlecitinib:Tofacitinib 5 mg BID |  | - |  | Very low | 0.47 (0.01-16.84) | Very low | 0.47 (0.01-16.84) | Very low |
| 115 | Ritlecitinib:Upadacitinib 15 mg QD |  | - |  | Very low | 0.78 (0.02-35) | Very low | 0.78 (0.02-35) | Very low |
| 116 | Ritlecitinib:Upadacitinib 30 mg QD |  | - |  | Very low | 1.07 (0.02-47.98) | Very low | 1.07 (0.02-47.98) | Very low |
| 117 | Ritlecitinib:Upadacitinib 45 mg QD |  | - |  | Very low | 1.08 (0.03-41.68) | Very low | 1.08 (0.03-41.68) | Very low |
| 118 | Tofacitinib 0.5 mg BID:Tofacitinib 1 mg BID |  | - |  | Very low | 0.73 (0.03-17.95) | Very low | 0.73 (0.03-17.95) | Very low |
| 119 | Tofacitinib 0.5 mg BID:Tofacitinib 10 mg BID | Low | 1.07 (0.07-17.41) | Very low | Very low | 1.11 (0.01-140.88) | Very low | 1.08 (0.1-12.1) | Very low |
| 120 | Tofacitinib 0.5 mg BID:Tofacitinib 15 mg BID | Low | 1.59 (0.1-25.9) | Very low | Very low | 0.16 (0-71.28) | Very low | 1.07 (0.08-13.52) | Very low |
| 121 | Tofacitinib 0.5 mg BID:Tofacitinib 3 mg BID | Low | 1.07 (0.07-17.41) | Very low |  | - |  | 1.07 (0.07-17.41) | Very low |
| 122 | Tofacitinib 0.5 mg BID:Tofacitinib 5 mg BID |  | - |  | Very low | 0.55 (0.04-7.42) | Very low | 0.55 (0.04-7.42) | Very low |
| 123 | Tofacitinib 0.5 mg BID:Upadacitinib 15 mg QD |  | - |  | Very low | 0.91 (0.05-18.27) | Very low | 0.91 (0.05-18.27) | Very low |
| 124 | Tofacitinib 0.5 mg BID:Upadacitinib 30 mg QD |  | - |  | Very low | 1.25 (0.06-25.04) | Very low | 1.25 (0.06-25.04) | Very low |
| 125 | Tofacitinib 0.5 mg BID:Upadacitinib 45 mg QD |  | - |  | Very low | 1.27 (0.08-20.79) | Very low | 1.27 (0.08-20.79) | Very low |
| 126 | Tofacitinib 1 mg BID:Tofacitinib 10 mg BID |  | - |  | Very low | 1.46 (0.1-20.79) | Very low | 1.46 (0.1-20.79) | Very low |
| 127 | Tofacitinib 1 mg BID:Tofacitinib 15 mg BID | Very low | 0.97 (0.06-15.85) | Very low | Very low | 9.89 (0.02-4434.58) | Very low | 1.45 (0.11-18.38) | Very low |
| 128 | Tofacitinib 1 mg BID:Tofacitinib 3 mg BID |  | - |  | Very low | 1.45 (0.06-35.39) | Very low | 1.45 (0.06-35.39) | Very low |
| 129 | Tofacitinib 1 mg BID:Tofacitinib 5 mg BID | Very low | 0.94 (0.06-15.39) | Very low | Very low | 0.3 (0-87.09) | Very low | 0.75 (0.06-9.24) | Very low |
| 130 | Tofacitinib 1 mg BID:Upadacitinib 15 mg QD |  | - |  | Very low | 1.24 (0.05-29.21) | Very low | 1.24 (0.05-29.21) | Very low |
| 131 | Tofacitinib 1 mg BID:Upadacitinib 30 mg QD |  | - |  | Very low | 1.7 (0.07-40.04) | Very low | 1.7 (0.07-40.04) | Very low |
| 132 | Tofacitinib 1 mg BID:Upadacitinib 45 mg QD |  | - |  | Very low | 1.73 (0.09-33.61) | Very low | 1.73 (0.09-33.61) | Very low |
| 133 | Tofacitinib 10 mg BID:Tofacitinib 15 mg BID | Low | 1.49 (0.09-24.29) | Very low | Very low | 0.66 (0.04-10.71) | Very low | 0.99 (0.14-7.12) | Low |
| 134 | Tofacitinib 10 mg BID:Tofacitinib 3 mg BID | Low | 1 (0.06-16.33) | Very low | Very low | 0.96 (0.01-122.11) | Very low | 0.99 (0.09-11.13) | Very low |
| 135 | Tofacitinib 10 mg BID:Tofacitinib 5 mg BID | Low | 0.62 (0.08-5.09) | Low | Very low | 0.37 (0.02-6.11) | Very low | 0.52 (0.1-2.77) | Low |
| 136 | Tofacitinib 10 mg BID:Upadacitinib 15 mg QD |  | - |  | Very low | 0.85 (0.07-9.66) | Very low | 0.85 (0.07-9.66) | Very low |
| 137 | Tofacitinib 10 mg BID:Upadacitinib 30 mg QD |  | - |  | Very low | 1.16 (0.1-13.24) | Very low | 1.16 (0.1-13.24) | Very low |
| 138 | Tofacitinib 10 mg BID:Upadacitinib 45 mg QD |  | - |  | Very low | 1.18 (0.13-10.44) | Very low | 1.18 (0.13-10.44) | Very low |
| 139 | Tofacitinib 15 mg BID:Tofacitinib 3 mg BID | Low | 0.67 (0.04-10.91) | Very low | Very low | 6.86 (0.01-3142.15) | Very low | 1 (0.08-12.65) | Very low |
| 140 | Tofacitinib 15 mg BID:Tofacitinib 5 mg BID | Very low | 0.97 (0.06-15.84) | Very low | Very low | 0.27 (0.02-4.62) | Very low | 0.52 (0.07-3.79) | Very low |
| 141 | Tofacitinib 15 mg BID:Upadacitinib 15 mg QD |  | - |  | Very low | 0.85 (0.06-11.87) | Very low | 0.85 (0.06-11.87) | Very low |
| 142 | Tofacitinib 15 mg BID:Upadacitinib 30 mg QD |  | - |  | Very low | 1.17 (0.08-16.27) | Very low | 1.17 (0.08-16.27) | Very low |
| 143 | Tofacitinib 15 mg BID:Upadacitinib 45 mg QD |  | - |  | Low | 1.19 (0.11-13.1) | Very low | 1.19 (0.11-13.1) | Very low |
| 144 | Tofacitinib 3 mg BID:Tofacitinib 5 mg BID |  | - |  | Very low | 0.52 (0.04-6.96) | Very low | 0.52 (0.04-6.96) | Very low |
| 145 | Tofacitinib 3 mg BID:Upadacitinib 15 mg QD |  | - |  | Very low | 0.85 (0.04-17.13) | Very low | 0.85 (0.04-17.13) | Very low |
| 146 | Tofacitinib 3 mg BID:Upadacitinib 30 mg QD |  | - |  | Very low | 1.17 (0.06-23.49) | Very low | 1.17 (0.06-23.49) | Very low |
| 147 | Tofacitinib 3 mg BID:Upadacitinib 45 mg QD |  | - |  | Very low | 1.19 (0.07-19.5) | Very low | 1.19 (0.07-19.5) | Very low |
| 148 | Tofacitinib 5 mg BID:Upadacitinib 15 mg QD |  | - |  | Very low | 1.64 (0.13-20.64) | Very low | 1.64 (0.13-20.64) | Very low |
| 149 | Tofacitinib 5 mg BID:Upadacitinib 30 mg QD |  | - |  | Very low | 2.25 (0.18-28.29) | Very low | 2.25 (0.18-28.29) | Very low |
| 150 | Tofacitinib 5 mg BID:Upadacitinib 45 mg QD |  | - |  | Very low | 2.29 (0.23-22.55) | Very low | 2.29 (0.23-22.55) | Very low |
| 151 | Upadacitinib 15 mg QD:Upadacitinib 30 mg QD | Low | 1.37 (0.14-13.26) | Very low |  | - |  | 1.37 (0.14-13.25) | Very low |
| 152 | Upadacitinib 15 mg QD:Upadacitinib 45 mg QD | Low | 2.53 (0.05-129.1) | Very low | Very low | 0.96 (0.04-21.6) | Very low | 1.39 (0.12-16.01) | Very low |
| 153 | Upadacitinib 30 mg QD:Upadacitinib 45 mg QD | Low | 1.05 (0.02-53.43) | Very low | Very low | 1 (0.04-22.34) | Very low | 1.02 (0.09-11.65) | Very low |

NMA: network meta-analysis; MACE: major adverse cardiovascular events; GRADE: Grading of Recommendations, Assessment, Development and Evaluation; OR: odds ratio; CI: confidence interval; QD: once daily; BID: twice daily.

**Supplementary table 11-2-2 Direct, indirect, NMA results and certainty for VTE (with dose consideration).**

| Number | Comparison | Direct estimate | | | Indirect estimate | | | Network estimate | |
| --- | --- | --- | --- | --- | --- | --- | --- | --- | --- |
|  |  | Preliminary-GRADE | OR (95% CI) | Final-GRADE | Preliminary-GRADE | OR (95% CI) | Final-GRADE | OR (95% CI) | Final-GRADE |
| 1 | Deucravacitinib:Brepocitinib |  | - |  | Very low | 0.19 (0-31.89) | Very low | 0.19 (0-31.89) | Very low |
| 2 | Filgotinib 100 mg QD:Brepocitinib |  | - |  | Very low | 3.53 (0.05-244.73) | Very low | 3.53 (0.05-244.73) | Very low |
| 3 | Filgotinib 200 mg QD:Brepocitinib |  | - |  | Very low | 5.11 (0.08-329.46) | Very low | 5.11 (0.08-329.46) | Very low |
| 4 | Ivarmacitinib:Brepocitinib |  | - |  | Low | 1.91 (0.01-501.87) | Very low | 1.91 (0.01-501.87) | Very low |
| 5 | Izencitinib:Brepocitinib |  | - |  | Very low | 1.88 (0.02-234.53) | Very low | 1.88 (0.02-234.53) | Very low |
| 6 | Peficitinib:Brepocitinib |  | - |  | Low | 1.4 (0.01-367.15) | Very low | 1.4 (0.01-367.15) | Very low |
| 7 | Placebo:Brepocitinib | Low | 5.78 (0.11-297.96) | Very low |  | - |  | 5.78 (0.11-297.96) | Very low |
| 8 | Ritlecitinib:Brepocitinib | Low | 0.95 (0.02-48.02) | Very low |  | - |  | 0.95 (0.02-48.02) | Very low |
| 9 | Tofacitinib 0.5 mg BID:Brepocitinib |  | - |  | Low | 3.87 (0.04-372.51) | Very low | 3.87 (0.04-372.51) | Very low |
| 10 | Tofacitinib 1 mg BID:Brepocitinib |  | - |  | Very low | 3.75 (0.04-387.65) | Very low | 3.75 (0.04-387.65) | Very low |
| 11 | Tofacitinib 10 mg BID:Brepocitinib |  | - |  | Very low | 2.47 (0.04-158.07) | Very low | 2.47 (0.04-158.07) | Very low |
| 12 | Tofacitinib 15 mg BID:Brepocitinib |  | - |  | Very low | 3.06 (0.04-227.74) | Very low | 3.06 (0.04-227.74) | Very low |
| 13 | Tofacitinib 3 mg BID:Brepocitinib |  | - |  | Low | 3.63 (0.04-349.43) | Very low | 3.63 (0.04-349.43) | Very low |
| 14 | Tofacitinib 5 mg BID:Brepocitinib |  | - |  | Very low | 3.45 (0.05-241.4) | Very low | 3.45 (0.05-241.4) | Very low |
| 15 | Upadacitinib 15 mg QD:Brepocitinib |  | - |  | Very low | 2.77 (0.03-241.12) | Very low | 2.77 (0.03-241.12) | Very low |
| 16 | Upadacitinib 30 mg QD:Brepocitinib |  | - |  | Very low | 7.85 (0.1-592.85) | Very low | 7.85 (0.1-592.85) | Very low |
| 17 | Upadacitinib 45 mg QD:Brepocitinib |  | - |  | Very low | 4.14 (0.06-276.26) | Very low | 4.14 (0.06-276.26) | Very low |
| 18 | Deucravacitinib:Filgotinib 100 mg QD |  | - |  | Very low | 0.05 (0-2.02) | Very low | 0.05 (0-2.02) | Very low |
| 19 | Deucravacitinib:Filgotinib 200 mg QD |  | - |  | Very low | 0.04 (0-1.28) | Very low | 0.04 (0-1.28) | Very low |
| 20 | Deucravacitinib:Ivarmacitinib |  | - |  | Very low | 0.1 (0-16.61) | Very low | 0.1 (0-16.61) | Very low |
| 21 | Deucravacitinib:Izencitinib |  | - |  | Very low | 0.1 (0-7.4) | Very low | 0.1 (0-7.4) | Very low |
| 22 | Deucravacitinib:Peficitinib |  | - |  | Very low | 0.14 (0-22.64) | Very low | 0.14 (0-22.64) | Very low |
| 23 | Deucravacitinib:Placebo | Very low | 0.03 (0-0.87) | Very low |  | - |  | 0.03 (0-0.87) | Very low |
| 24 | Deucravacitinib:Ritlecitinib |  | - |  | Very low | 0.2 (0-33.69) | Very low | 0.2 (0-33.69) | Very low |
| 25 | Deucravacitinib:Tofacitinib 0.5 mg BID |  | - |  | Very low | 0.05 (0-2.69) | Very low | 0.05 (0-2.69) | Very low |
| 26 | Deucravacitinib:Tofacitinib 1 mg BID |  | - |  | Very low | 0.05 (0-3) | Very low | 0.05 (0-3) | Very low |
| 27 | Deucravacitinib:Tofacitinib 10 mg BID |  | - |  | Very low | 0.08 (0-2.62) | Very low | 0.08 (0-2.62) | Very low |
| 28 | Deucravacitinib:Tofacitinib 15 mg BID |  | - |  | Very low | 0.06 (0-2.52) | Very low | 0.06 (0-2.52) | Very low |
| 29 | Deucravacitinib:Tofacitinib 3 mg BID |  | - |  | Very low | 0.05 (0-2.86) | Very low | 0.05 (0-2.86) | Very low |
| 30 | Deucravacitinib:Tofacitinib 5 mg BID |  | - |  | Very low | 0.05 (0-2.09) | Very low | 0.05 (0-2.09) | Very low |
| 31 | Deucravacitinib:Upadacitinib 15 mg QD |  | - |  | Very low | 0.07 (0-3.35) | Very low | 0.07 (0-3.35) | Very low |
| 32 | Deucravacitinib:Upadacitinib 30 mg QD |  | - |  | Very low | 0.02 (0-1) | Very low | 0.02 (0-1) | Very low |
| 33 | Deucravacitinib:Upadacitinib 45 mg QD |  | - |  | Very low | 0.05 (0-1.64) | Very low | 0.05 (0-1.64) | Very low |
| 34 | Filgotinib 100 mg QD:Filgotinib 200 mg QD | Low | 0.66 (0.14-3.11) | Low | Low | 1.28 (0-599.74) | Very low | 0.69 (0.15-3.09) | Low |
| 35 | Filgotinib 100 mg QD:Ivarmacitinib |  | - |  | Very low | 1.85 (0.03-127.3) | Very low | 1.85 (0.03-127.3) | Very low |
| 36 | Filgotinib 100 mg QD:Izencitinib |  | - |  | Very low | 1.88 (0.08-45.44) | Very low | 1.88 (0.08-45.44) | Very low |
| 37 | Filgotinib 100 mg QD:Peficitinib |  | - |  | Very low | 2.52 (0.04-173.5) | Very low | 2.52 (0.04-173.5) | Very low |
| 38 | Filgotinib 100 mg QD:Placebo | Low | 0.65 (0.13-3.27) | Low | Very low | 0.22 (0-102.92) | Very low | 0.61 (0.13-2.9) | Low |
| 39 | Filgotinib 100 mg QD:Ritlecitinib |  | - |  | Very low | 3.73 (0.05-258.52) | Very low | 3.73 (0.05-258.52) | Very low |
| 40 | Filgotinib 100 mg QD:Tofacitinib 0.5 mg BID |  | - |  | Very low | 0.91 (0.06-14.72) | Very low | 0.91 (0.06-14.72) | Very low |
| 41 | Filgotinib 100 mg QD:Tofacitinib 1 mg BID |  | - |  | Very low | 0.94 (0.05-17.01) | Very low | 0.94 (0.05-17.01) | Very low |
| 42 | Filgotinib 100 mg QD:Tofacitinib 10 mg BID |  | - |  | Very low | 1.43 (0.19-10.95) | Very low | 1.43 (0.19-10.95) | Very low |
| 43 | Filgotinib 100 mg QD:Tofacitinib 15 mg BID |  | - |  | Very low | 1.15 (0.11-11.86) | Very low | 1.15 (0.11-11.86) | Very low |
| 44 | Filgotinib 100 mg QD:Tofacitinib 3 mg BID |  | - |  | Very low | 0.97 (0.06-15.67) | Very low | 0.97 (0.06-15.67) | Very low |
| 45 | Filgotinib 100 mg QD:Tofacitinib 5 mg BID |  | - |  | Very low | 1.02 (0.11-9.4) | Very low | 1.02 (0.11-9.4) | Very low |
| 46 | Filgotinib 100 mg QD:Upadacitinib 15 mg QD |  | - |  | Very low | 1.28 (0.09-17.42) | Very low | 1.28 (0.09-17.42) | Very low |
| 47 | Filgotinib 100 mg QD:Upadacitinib 30 mg QD |  | - |  | Very low | 0.45 (0.04-4.77) | Very low | 0.45 (0.04-4.77) | Very low |
| 48 | Filgotinib 100 mg QD:Upadacitinib 45 mg QD |  | - |  | Very low | 0.85 (0.1-7.13) | Very low | 0.85 (0.1-7.13) | Very low |
| 49 | Filgotinib 200 mg QD:Ivarmacitinib |  | - |  | Very low | 2.68 (0.04-171.35) | Very low | 2.68 (0.04-171.35) | Very low |
| 50 | Filgotinib 200 mg QD:Izencitinib |  | - |  | Very low | 2.72 (0.12-59.68) | Very low | 2.72 (0.12-59.68) | Very low |
| 51 | Filgotinib 200 mg QD:Peficitinib |  | - |  | Very low | 3.65 (0.06-233.54) | Very low | 3.65 (0.06-233.54) | Very low |
| 52 | Filgotinib 200 mg QD:Placebo | Low | 0.86 (0.22-3.31) | Low | Very low | 24.48 (0-26509352.12) | Very low | 0.88 (0.23-3.39) | Low |
| 53 | Filgotinib 200 mg QD:Ritlecitinib |  | - |  | Very low | 5.4 (0.08-348.03) | Very low | 5.4 (0.08-348.03) | Very low |
| 54 | Filgotinib 200 mg QD:Tofacitinib 0.5 mg BID |  | - |  | Very low | 1.32 (0.09-19.05) | Very low | 1.32 (0.09-19.05) | Very low |
| 55 | Filgotinib 200 mg QD:Tofacitinib 1 mg BID |  | - |  | Very low | 1.36 (0.08-22.12) | Very low | 1.36 (0.08-22.12) | Very low |
| 56 | Filgotinib 200 mg QD:Tofacitinib 10 mg BID |  | - |  | Very low | 2.07 (0.31-13.57) | Very low | 2.07 (0.31-13.57) | Very low |
| 57 | Filgotinib 200 mg QD:Tofacitinib 15 mg BID |  | - |  | Very low | 1.67 (0.19-15.01) | Very low | 1.67 (0.19-15.01) | Very low |
| 58 | Filgotinib 200 mg QD:Tofacitinib 3 mg BID |  | - |  | Very low | 1.41 (0.1-20.28) | Very low | 1.41 (0.1-20.28) | Very low |
| 59 | Filgotinib 200 mg QD:Tofacitinib 5 mg BID |  | - |  | Very low | 1.48 (0.19-11.8) | Very low | 1.48 (0.19-11.8) | Very low |
| 60 | Filgotinib 200 mg QD:Upadacitinib 15 mg QD |  | - |  | Very low | 1.85 (0.15-22.38) | Very low | 1.85 (0.15-22.38) | Very low |
| 61 | Filgotinib 200 mg QD:Upadacitinib 30 mg QD |  | - |  | Very low | 0.65 (0.07-6.04) | Very low | 0.65 (0.07-6.04) | Very low |
| 62 | Filgotinib 200 mg QD:Upadacitinib 45 mg QD |  | - |  | Very low | 1.23 (0.17-8.89) | Very low | 1.23 (0.17-8.89) | Very low |
| 63 | Ivarmacitinib:Izencitinib |  | - |  | Very low | 1.02 (0.01-125.8) | Very low | 1.02 (0.01-125.8) | Very low |
| 64 | Ivarmacitinib:Peficitinib |  | - |  | Low | 1.37 (0.01-356.53) | Very low | 1.37 (0.01-356.53) | Very low |
| 65 | Ivarmacitinib:Placebo | Low | 0.33 (0.01-16.93) | Very low |  | - |  | 0.33 (0.01-16.93) | Very low |
| 66 | Ivarmacitinib:Ritlecitinib |  | - |  | Low | 2.02 (0.01-530.17) | Very low | 2.02 (0.01-530.17) | Very low |
| 67 | Ivarmacitinib:Tofacitinib 0.5 mg BID |  | - |  | Low | 0.49 (0.01-47.24) | Very low | 0.49 (0.01-47.24) | Very low |
| 68 | Ivarmacitinib:Tofacitinib 1 mg BID |  | - |  | Very low | 0.51 (0-52.27) | Very low | 0.51 (0-52.27) | Very low |
| 69 | Ivarmacitinib:Tofacitinib 10 mg BID |  | - |  | Very low | 0.77 (0.01-48.99) | Very low | 0.77 (0.01-48.99) | Very low |
| 70 | Ivarmacitinib:Tofacitinib 15 mg BID |  | - |  | Very low | 0.62 (0.01-46.05) | Very low | 0.62 (0.01-46.05) | Very low |
| 71 | Ivarmacitinib:Tofacitinib 3 mg BID |  | - |  | Low | 0.53 (0.01-50.32) | Very low | 0.53 (0.01-50.32) | Very low |
| 72 | Ivarmacitinib:Tofacitinib 5 mg BID |  | - |  | Very low | 0.55 (0.01-38.49) | Very low | 0.55 (0.01-38.49) | Very low |
| 73 | Ivarmacitinib:Upadacitinib 15 mg QD |  | - |  | Very low | 0.69 (0.01-59.8) | Very low | 0.69 (0.01-59.8) | Very low |
| 74 | Ivarmacitinib:Upadacitinib 30 mg QD |  | - |  | Very low | 0.24 (0-18.27) | Very low | 0.24 (0-18.27) | Very low |
| 75 | Ivarmacitinib:Upadacitinib 45 mg QD |  | - |  | Very low | 0.46 (0.01-30.53) | Very low | 0.46 (0.01-30.53) | Very low |
| 76 | Izencitinib:Peficitinib |  | - |  | Very low | 1.34 (0.01-166.45) | Very low | 1.34 (0.01-166.45) | Very low |
| 77 | Izencitinib:Placebo | Low | 0.33 (0.02-5.26) | Low |  | - |  | 0.33 (0.02-5.26) | Low |
| 78 | Izencitinib:Ritlecitinib |  | - |  | Very low | 1.99 (0.02-247.76) | Very low | 1.99 (0.02-247.76) | Very low |
| 79 | Izencitinib:Tofacitinib 0.5 mg BID |  | - |  | Very low | 0.49 (0.01-18.02) | Very low | 0.49 (0.01-18.02) | Very low |
| 80 | Izencitinib:Tofacitinib 1 mg BID |  | - |  | Very low | 0.5 (0.01-20.31) | Very low | 0.5 (0.01-20.31) | Very low |
| 81 | Izencitinib:Tofacitinib 10 mg BID |  | - |  | Very low | 0.76 (0.04-16.51) | Very low | 0.76 (0.04-16.51) | Very low |
| 82 | Izencitinib:Tofacitinib 15 mg BID |  | - |  | Very low | 0.61 (0.02-16.31) | Very low | 0.61 (0.02-16.31) | Very low |
| 83 | Izencitinib:Tofacitinib 3 mg BID |  | - |  | Very low | 0.52 (0.01-19.2) | Very low | 0.52 (0.01-19.2) | Very low |
| 84 | Izencitinib:Tofacitinib 5 mg BID |  | - |  | Very low | 0.55 (0.02-13.37) | Very low | 0.55 (0.02-13.37) | Very low |
| 85 | Izencitinib:Upadacitinib 15 mg QD |  | - |  | Very low | 0.68 (0.02-22.21) | Very low | 0.68 (0.02-22.21) | Very low |
| 86 | Izencitinib:Upadacitinib 30 mg QD |  | - |  | Very low | 0.24 (0.01-6.5) | Very low | 0.24 (0.01-6.5) | Very low |
| 87 | Izencitinib:Upadacitinib 45 mg QD |  | - |  | Very low | 0.45 (0.02-10.44) | Very low | 0.45 (0.02-10.44) | Very low |
| 88 | Peficitinib:Placebo | Low | 0.24 (0-12.38) | Very low |  | - |  | 0.24 (0-12.38) | Very low |
| 89 | Peficitinib:Ritlecitinib |  | - |  | Low | 1.48 (0.01-387.85) | Very low | 1.48 (0.01-387.85) | Very low |
| 90 | Peficitinib:Tofacitinib 0.5 mg BID |  | - |  | Low | 0.36 (0-34.55) | Very low | 0.36 (0-34.55) | Very low |
| 91 | Peficitinib:Tofacitinib 1 mg BID |  | - |  | Very low | 0.37 (0-38.23) | Very low | 0.37 (0-38.23) | Very low |
| 92 | Peficitinib:Tofacitinib 10 mg BID |  | - |  | Very low | 0.57 (0.01-35.83) | Very low | 0.57 (0.01-35.83) | Very low |
| 93 | Peficitinib:Tofacitinib 15 mg BID |  | - |  | Very low | 0.46 (0.01-33.68) | Very low | 0.46 (0.01-33.68) | Very low |
| 94 | Peficitinib:Tofacitinib 3 mg BID |  | - |  | Low | 0.39 (0-36.8) | Very low | 0.39 (0-36.8) | Very low |
| 95 | Peficitinib:Tofacitinib 5 mg BID |  | - |  | Very low | 0.41 (0.01-28.15) | Very low | 0.41 (0.01-28.15) | Very low |
| 96 | Peficitinib:Upadacitinib 15 mg QD |  | - |  | Very low | 0.51 (0.01-43.73) | Very low | 0.51 (0.01-43.73) | Very low |
| 97 | Peficitinib:Upadacitinib 30 mg QD |  | - |  | Very low | 0.18 (0-13.36) | Very low | 0.18 (0-13.36) | Very low |
| 98 | Peficitinib:Upadacitinib 45 mg QD |  | - |  | Very low | 0.34 (0.01-22.33) | Very low | 0.34 (0.01-22.33) | Very low |
| 99 | Placebo:Ritlecitinib | Low | 6.1 (0.12-314.74) | Very low |  | - |  | 6.1 (0.12-314.74) | Very low |
| 100 | Placebo:Tofacitinib 0.5 mg BID | Low | 1.25 (0.1-15.3) | Very low | Very low | 3.98 (0.01-1511.73) | Very low | 1.49 (0.15-14.97) | Very low |
| 101 | Placebo:Tofacitinib 1 mg BID | Very low | 1.06 (0.06-17.28) | Very low | Very low | 5.19 (0.03-796.74) | Very low | 1.54 (0.13-17.69) | Very low |
| 102 | Placebo:Tofacitinib 10 mg BID | Low | 2.39 (0.62-9.24) | Low | Very low | 1.43 (0-566.68) | Very low | 2.34 (0.63-8.71) | Low |
| 103 | Placebo:Tofacitinib 15 mg BID | Low | 1.49 (0.23-9.57) | Low | Very low | 9.37 (0.07-1184.68) | Very low | 1.89 (0.33-10.71) | Very low |
| 104 | Placebo:Tofacitinib 3 mg BID | Low | 1.34 (0.11-16.29) | Very low | Very low | 4.24 (0.01-1603.58) | Very low | 1.59 (0.16-15.93) | Very low |
| 105 | Placebo:Tofacitinib 5 mg BID | Low | 1.34 (0.25-7.2) | Low | Very low | 9.36 (0.09-1021.81) | Very low | 1.67 (0.34-8.14) | Low |
| 106 | Placebo:Upadacitinib 15 mg QD | Low | 1.02 (0.11-9.83) | Low | Very low | 156.27 (0.61-40362.65) | Very low | 2.09 (0.26-17.06) | Very low |
| 107 | Placebo:Upadacitinib 30 mg QD | Low | 0.45 (0.07-3.09) | Low | Very low | 12.3 (0.12-1230.16) | Very low | 0.74 (0.12-4.35) | Low |
| 108 | Placebo:Upadacitinib 45 mg QD | Low | 2.14 (0.45-10.21) | Very low | Very low | 0.11 (0-4.86) | Very low | 1.39 (0.33-5.92) | Low |
| 109 | Ritlecitinib:Tofacitinib 0.5 mg BID |  | - |  | Low | 0.24 (0-23.56) | Very low | 0.24 (0-23.56) | Very low |
| 110 | Ritlecitinib:Tofacitinib 1 mg BID |  | - |  | Very low | 0.25 (0-26.07) | Very low | 0.25 (0-26.07) | Very low |
| 111 | Ritlecitinib:Tofacitinib 10 mg BID |  | - |  | Very low | 0.38 (0.01-24.45) | Very low | 0.38 (0.01-24.45) | Very low |
| 112 | Ritlecitinib:Tofacitinib 15 mg BID |  | - |  | Very low | 0.31 (0-22.98) | Very low | 0.31 (0-22.98) | Very low |
| 113 | Ritlecitinib:Tofacitinib 3 mg BID |  | - |  | Low | 0.26 (0-25.1) | Very low | 0.26 (0-25.1) | Very low |
| 114 | Ritlecitinib:Tofacitinib 5 mg BID |  | - |  | Very low | 0.27 (0-19.2) | Very low | 0.27 (0-19.2) | Very low |
| 115 | Ritlecitinib:Upadacitinib 15 mg QD |  | - |  | Very low | 0.34 (0-29.83) | Very low | 0.34 (0-29.83) | Very low |
| 116 | Ritlecitinib:Upadacitinib 30 mg QD |  | - |  | Very low | 0.12 (0-9.11) | Very low | 0.12 (0-9.11) | Very low |
| 117 | Ritlecitinib:Upadacitinib 45 mg QD |  | - |  | Very low | 0.23 (0-15.24) | Very low | 0.23 (0-15.24) | Very low |
| 118 | Tofacitinib 0.5 mg BID:Tofacitinib 1 mg BID |  | - |  | Very low | 1.03 (0.04-26.16) | Very low | 1.03 (0.04-26.16) | Very low |
| 119 | Tofacitinib 0.5 mg BID:Tofacitinib 10 mg BID | Low | 1.07 (0.07-17.41) | Very low | Very low | 5.15 (0.04-709.65) | Very low | 1.56 (0.14-17.76) | Very low |
| 120 | Tofacitinib 0.5 mg BID:Tofacitinib 15 mg BID | Low | 1.59 (0.1-25.9) | Very low | Very low | 0.39 (0-213) | Very low | 1.26 (0.1-16.2) | Very low |
| 121 | Tofacitinib 0.5 mg BID:Tofacitinib 3 mg BID | Low | 1.07 (0.07-17.41) | Very low |  | - |  | 1.07 (0.07-17.41) | Very low |
| 122 | Tofacitinib 0.5 mg BID:Tofacitinib 5 mg BID |  | - |  | Very low | 1.12 (0.08-16.34) | Very low | 1.12 (0.08-16.34) | Very low |
| 123 | Tofacitinib 0.5 mg BID:Upadacitinib 15 mg QD |  | - |  | Very low | 1.4 (0.06-31.63) | Very low | 1.4 (0.06-31.63) | Very low |
| 124 | Tofacitinib 0.5 mg BID:Upadacitinib 30 mg QD |  | - |  | Very low | 0.49 (0.03-9.05) | Very low | 0.49 (0.03-9.05) | Very low |
| 125 | Tofacitinib 0.5 mg BID:Upadacitinib 45 mg QD |  | - |  | Very low | 0.93 (0.06-14.19) | Very low | 0.93 (0.06-14.19) | Very low |
| 126 | Tofacitinib 1 mg BID:Tofacitinib 10 mg BID |  | - |  | Very low | 1.52 (0.11-21.58) | Very low | 1.52 (0.11-21.58) | Very low |
| 127 | Tofacitinib 1 mg BID:Tofacitinib 15 mg BID | Very low | 0.97 (0.06-15.85) | Very low | Very low | 3.95 (0.01-2107.01) | Very low | 1.22 (0.1-15.7) | Very low |
| 128 | Tofacitinib 1 mg BID:Tofacitinib 3 mg BID |  | - |  | Very low | 1.03 (0.04-26.2) | Very low | 1.03 (0.04-26.2) | Very low |
| 129 | Tofacitinib 1 mg BID:Tofacitinib 5 mg BID | Very low | 0.94 (0.06-15.39) | Very low | Very low | 2.01 (0.01-676.42) | Very low | 1.09 (0.09-13.48) | Very low |
| 130 | Tofacitinib 1 mg BID:Upadacitinib 15 mg QD |  | - |  | Very low | 1.36 (0.05-33.98) | Very low | 1.36 (0.05-33.98) | Very low |
| 131 | Tofacitinib 1 mg BID:Upadacitinib 30 mg QD |  | - |  | Very low | 0.48 (0.02-9.79) | Very low | 0.48 (0.02-9.79) | Very low |
| 132 | Tofacitinib 1 mg BID:Upadacitinib 45 mg QD |  | - |  | Very low | 0.91 (0.05-15.46) | Very low | 0.91 (0.05-15.46) | Very low |
| 133 | Tofacitinib 10 mg BID:Tofacitinib 15 mg BID | Low | 1.49 (0.09-24.29) | Very low | Very low | 0.44 (0.03-7.02) | Very low | 0.81 (0.11-5.76) | Low |
| 134 | Tofacitinib 10 mg BID:Tofacitinib 3 mg BID | Low | 1 (0.06-16.33) | Very low | Very low | 0.21 (0-28.43) | Very low | 0.68 (0.06-7.73) | Low |
| 135 | Tofacitinib 10 mg BID:Tofacitinib 5 mg BID | Low | 1 (0.1-9.65) | Low | Very low | 0.42 (0.02-7.59) | Very low | 0.72 (0.12-4.28) | Low |
| 136 | Tofacitinib 10 mg BID:Upadacitinib 15 mg QD |  | - |  | Very low | 0.89 (0.07-10.67) | Very low | 0.89 (0.07-10.67) | Very low |
| 137 | Tofacitinib 10 mg BID:Upadacitinib 30 mg QD |  | - |  | Very low | 0.32 (0.03-2.88) | Very low | 0.32 (0.03-2.88) | Very low |
| 138 | Tofacitinib 10 mg BID:Upadacitinib 45 mg QD |  | - |  | Very low | 0.6 (0.08-4.22) | Very low | 0.6 (0.08-4.22) | Very low |
| 139 | Tofacitinib 15 mg BID:Tofacitinib 3 mg BID | Low | 0.67 (0.04-10.91) | Very low | Very low | 2.73 (0.01-1489.75) | Very low | 0.84 (0.07-10.81) | Very low |
| 140 | Tofacitinib 15 mg BID:Tofacitinib 5 mg BID | Very low | 0.97 (0.06-15.84) | Very low | Very low | 0.8 (0.04-15.63) | Very low | 0.89 (0.12-6.79) | Very low |
| 141 | Tofacitinib 15 mg BID:Upadacitinib 15 mg QD |  | - |  | Very low | 1.11 (0.07-16.91) | Very low | 1.11 (0.07-16.91) | Very low |
| 142 | Tofacitinib 15 mg BID:Upadacitinib 30 mg QD |  | - |  | Very low | 0.39 (0.03-4.68) | Very low | 0.39 (0.03-4.68) | Very low |
| 143 | Tofacitinib 15 mg BID:Upadacitinib 45 mg QD |  | - |  | Low | 0.74 (0.08-7.09) | Low | 0.74 (0.08-7.09) | Low |
| 144 | Tofacitinib 3 mg BID:Tofacitinib 5 mg BID |  | - |  | Very low | 1.05 (0.07-15.32) | Very low | 1.05 (0.07-15.32) | Very low |
| 145 | Tofacitinib 3 mg BID:Upadacitinib 15 mg QD |  | - |  | Very low | 1.31 (0.06-29.66) | Very low | 1.31 (0.06-29.66) | Very low |
| 146 | Tofacitinib 3 mg BID:Upadacitinib 30 mg QD |  | - |  | Very low | 0.46 (0.03-8.49) | Very low | 0.46 (0.03-8.49) | Very low |
| 147 | Tofacitinib 3 mg BID:Upadacitinib 45 mg QD |  | - |  | Very low | 0.88 (0.06-13.31) | Very low | 0.88 (0.06-13.31) | Very low |
| 148 | Tofacitinib 5 mg BID:Upadacitinib 15 mg QD |  | - |  | Very low | 1.25 (0.09-17.28) | Very low | 1.25 (0.09-17.28) | Very low |
| 149 | Tofacitinib 5 mg BID:Upadacitinib 30 mg QD |  | - |  | Very low | 0.44 (0.04-4.74) | Very low | 0.44 (0.04-4.74) | Very low |
| 150 | Tofacitinib 5 mg BID:Upadacitinib 45 mg QD |  | - |  | Very low | 0.83 (0.1-7.09) | Very low | 0.83 (0.1-7.09) | Very low |
| 151 | Upadacitinib 15 mg QD:Upadacitinib 30 mg QD | Low | 0.45 (0.07-3.06) | Low | Very low | 0 (0-8.35) | Very low | 0.35 (0.05-2.35) | Low |
| 152 | Upadacitinib 15 mg QD:Upadacitinib 45 mg QD | Low | 0.5 (0.02-10.54) | Very low | Very low | 0.89 (0.04-18.3) | Very low | 0.67 (0.08-5.72) | Low |
| 153 | Upadacitinib 30 mg QD:Upadacitinib 45 mg QD | Low | 0.21 (0.01-4.36) | Low | Very low | 8.36 (0.69-101.31) | Very low | 1.89 (0.27-13.05) | Very low |

NMA: network meta-analysis; VTE: venous thromboembolism events; GRADE: Grading of Recommendations, Assessment, Development and Evaluation; OR: odds ratio; CI: confidence interval; QD: once daily; BID: twice daily.

**Supplementary table 11-2-3 Direct, indirect, NMA results and certainty for CVE (with dose consideration).**

| Number | Comparison | Direct estimate | | | Indirect estimate | | | Network estimate | |
| --- | --- | --- | --- | --- | --- | --- | --- | --- | --- |
|  |  | Preliminary-GRADE | OR (95% CI) | Final-GRADE | Preliminary-GRADE | OR (95% CI) | Final-GRADE | OR (95% CI) | Final-GRADE |
| 1 | Deucravacitinib:Brepocitinib |  | - |  | Low | 0.19 (0-31.89) | Very low | 0.19 (0-31.89) | Very low |
| 2 | Filgotinib 100 mg QD:Brepocitinib |  | - |  | Low | 3.53 (0.05-244.73) | Very low | 3.53 (0.05-244.73) | Very low |
| 3 | Filgotinib 200 mg QD:Brepocitinib |  | - |  | Low | 5.11 (0.08-329.46) | Very low | 5.11 (0.08-329.46) | Very low |
| 4 | Ivarmacitinib:Brepocitinib |  | - |  | Moderate | 1.91 (0.01-501.87) | Low | 1.91 (0.01-501.87) | Low |
| 5 | Izencitinib:Brepocitinib |  | - |  | Low | 1.88 (0.02-234.53) | Very low | 1.88 (0.02-234.53) | Very low |
| 6 | Peficitinib:Brepocitinib |  | - |  | Moderate | 1.4 (0.01-367.15) | Low | 1.4 (0.01-367.15) | Low |
| 7 | Placebo:Brepocitinib | Moderate | 5.78 (0.11-297.96) | Low |  | - |  | 5.78 (0.11-297.96) | Low |
| 8 | Ritlecitinib:Brepocitinib | Moderate | 2.86 (0.12-70.75) | Low |  | - |  | 2.86 (0.12-70.75) | Low |
| 9 | Tofacitinib 0.5 mg BID:Brepocitinib |  | - |  | Moderate | 2.4 (0.03-216.84) | Low | 2.4 (0.03-216.84) | Low |
| 10 | Tofacitinib 1 mg BID:Brepocitinib |  | - |  | Very low | 3.75 (0.04-382.07) | Very low | 3.75 (0.04-382.07) | Very low |
| 11 | Tofacitinib 10 mg BID:Brepocitinib |  | - |  | Low | 2.04 (0.03-129.07) | Very low | 2.04 (0.03-129.07) | Very low |
| 12 | Tofacitinib 15 mg BID:Brepocitinib |  | - |  | Low | 2.41 (0.03-173.82) | Very low | 2.41 (0.03-173.82) | Very low |
| 13 | Tofacitinib 3 mg BID:Brepocitinib |  | - |  | Moderate | 2.25 (0.02-203.41) | Low | 2.25 (0.02-203.41) | Low |
| 14 | Tofacitinib 5 mg BID:Brepocitinib |  | - |  | Low | 4.38 (0.07-287.54) | Very low | 4.38 (0.07-287.54) | Very low |
| 15 | Upadacitinib 15 mg QD:Brepocitinib |  | - |  | Low | 2.3 (0.03-187.42) | Very low | 2.3 (0.03-187.42) | Very low |
| 16 | Upadacitinib 30 mg QD:Brepocitinib |  | - |  | Low | 6.24 (0.09-424.1) | Very low | 6.24 (0.09-424.1) | Very low |
| 17 | Upadacitinib 45 mg QD:Brepocitinib |  | - |  | Low | 3.9 (0.06-257.83) | Very low | 3.9 (0.06-257.83) | Very low |
| 18 | Deucravacitinib:Filgotinib 100 mg QD |  | - |  | Very low | 0.05 (0-2.02) | Very low | 0.05 (0-2.02) | Very low |
| 19 | Deucravacitinib:Filgotinib 200 mg QD |  | - |  | Very low | 0.04 (0-1.28) | Very low | 0.04 (0-1.28) | Very low |
| 20 | Deucravacitinib:Ivarmacitinib |  | - |  | Low | 0.1 (0-16.61) | Very low | 0.1 (0-16.61) | Very low |
| 21 | Deucravacitinib:Izencitinib |  | - |  | Very low | 0.1 (0-7.4) | Very low | 0.1 (0-7.4) | Very low |
| 22 | Deucravacitinib:Peficitinib |  | - |  | Low | 0.14 (0-22.64) | Very low | 0.14 (0-22.64) | Very low |
| 23 | Deucravacitinib:Placebo | Low | 0.03 (0-0.87) | Low |  | - |  | 0.03 (0-0.87) | Low |
| 24 | Deucravacitinib:Ritlecitinib |  | - |  | Low | 0.07 (0-6.59) | Low | 0.07 (0-6.59) | Low |
| 25 | Deucravacitinib:Tofacitinib 0.5 mg BID |  | - |  | Low | 0.08 (0-4.02) | Low | 0.08 (0-4.02) | Low |
| 26 | Deucravacitinib:Tofacitinib 1 mg BID |  | - |  | Very low | 0.05 (0-2.95) | Very low | 0.05 (0-2.95) | Very low |
| 27 | Deucravacitinib:Tofacitinib 10 mg BID |  | - |  | Very low | 0.09 (0-3.12) | Very low | 0.09 (0-3.12) | Very low |
| 28 | Deucravacitinib:Tofacitinib 15 mg BID |  | - |  | Very low | 0.08 (0-3.09) | Very low | 0.08 (0-3.09) | Very low |
| 29 | Deucravacitinib:Tofacitinib 3 mg BID |  | - |  | Low | 0.08 (0-4.29) | Low | 0.08 (0-4.29) | Low |
| 30 | Deucravacitinib:Tofacitinib 5 mg BID |  | - |  | Very low | 0.04 (0-1.52) | Very low | 0.04 (0-1.52) | Very low |
| 31 | Deucravacitinib:Upadacitinib 15 mg QD |  | - |  | Very low | 0.08 (0-3.74) | Very low | 0.08 (0-3.74) | Very low |
| 32 | Deucravacitinib:Upadacitinib 30 mg QD |  | - |  | Very low | 0.03 (0-1.12) | Very low | 0.03 (0-1.12) | Very low |
| 33 | Deucravacitinib:Upadacitinib 45 mg QD |  | - |  | Very low | 0.05 (0-1.73) | Very low | 0.05 (0-1.73) | Very low |
| 34 | Filgotinib 100 mg QD:Filgotinib 200 mg QD | Moderate | 0.66 (0.14-3.11) | Moderate | Moderate | 1.28 (0-599.74) | Low | 0.69 (0.15-3.09) | Moderate |
| 35 | Filgotinib 100 mg QD:Ivarmacitinib |  | - |  | Low | 1.85 (0.03-127.3) | Very low | 1.85 (0.03-127.3) | Very low |
| 36 | Filgotinib 100 mg QD:Izencitinib |  | - |  | Low | 1.88 (0.08-45.44) | Very low | 1.88 (0.08-45.44) | Very low |
| 37 | Filgotinib 100 mg QD:Peficitinib |  | - |  | Low | 2.52 (0.04-173.5) | Very low | 2.52 (0.04-173.5) | Very low |
| 38 | Filgotinib 100 mg QD:Placebo | Moderate | 0.65 (0.13-3.27) | Moderate | Low | 0.22 (0-102.92) | Very low | 0.61 (0.13-2.9) | Moderate |
| 39 | Filgotinib 100 mg QD:Ritlecitinib |  | - |  | Low | 1.23 (0.03-44.48) | Very low | 1.23 (0.03-44.48) | Very low |
| 40 | Filgotinib 100 mg QD:Tofacitinib 0.5 mg BID |  | - |  | Low | 1.47 (0.1-21.31) | Very low | 1.47 (0.1-21.31) | Very low |
| 41 | Filgotinib 100 mg QD:Tofacitinib 1 mg BID |  | - |  | Very low | 0.94 (0.05-16.62) | Very low | 0.94 (0.05-16.62) | Very low |
| 42 | Filgotinib 100 mg QD:Tofacitinib 10 mg BID |  | - |  | Low | 1.73 (0.23-12.95) | Very low | 1.73 (0.23-12.95) | Very low |
| 43 | Filgotinib 100 mg QD:Tofacitinib 15 mg BID |  | - |  | Low | 1.46 (0.15-14.21) | Very low | 1.46 (0.15-14.21) | Very low |
| 44 | Filgotinib 100 mg QD:Tofacitinib 3 mg BID |  | - |  | Low | 1.57 (0.11-22.69) | Very low | 1.57 (0.11-22.69) | Very low |
| 45 | Filgotinib 100 mg QD:Tofacitinib 5 mg BID |  | - |  | Low | 0.81 (0.1-6.53) | Low | 0.81 (0.1-6.53) | Low |
| 46 | Filgotinib 100 mg QD:Upadacitinib 15 mg QD |  | - |  | Low | 1.54 (0.13-18.69) | Very low | 1.54 (0.13-18.69) | Very low |
| 47 | Filgotinib 100 mg QD:Upadacitinib 30 mg QD |  | - |  | Low | 0.57 (0.07-4.91) | Low | 0.57 (0.07-4.91) | Low |
| 48 | Filgotinib 100 mg QD:Upadacitinib 45 mg QD |  | - |  | Low | 0.91 (0.11-7.46) | Low | 0.91 (0.11-7.46) | Low |
| 49 | Filgotinib 200 mg QD:Ivarmacitinib |  | - |  | Low | 2.68 (0.04-171.35) | Very low | 2.68 (0.04-171.35) | Very low |
| 50 | Filgotinib 200 mg QD:Izencitinib |  | - |  | Low | 2.72 (0.12-59.68) | Very low | 2.72 (0.12-59.68) | Very low |
| 51 | Filgotinib 200 mg QD:Peficitinib |  | - |  | Low | 3.65 (0.06-233.54) | Very low | 3.65 (0.06-233.54) | Very low |
| 52 | Filgotinib 200 mg QD:Placebo | Moderate | 0.86 (0.22-3.31) | Moderate | Low | 24.48 (0-26509351.09) | Very low | 0.88 (0.23-3.39) | Moderate |
| 53 | Filgotinib 200 mg QD:Ritlecitinib |  | - |  | Low | 1.79 (0.05-59.07) | Very low | 1.79 (0.05-59.07) | Very low |
| 54 | Filgotinib 200 mg QD:Tofacitinib 0.5 mg BID |  | - |  | Low | 2.13 (0.17-27.44) | Very low | 2.13 (0.17-27.44) | Very low |
| 55 | Filgotinib 200 mg QD:Tofacitinib 1 mg BID |  | - |  | Very low | 1.36 (0.09-21.59) | Very low | 1.36 (0.09-21.59) | Very low |
| 56 | Filgotinib 200 mg QD:Tofacitinib 10 mg BID |  | - |  | Low | 2.5 (0.39-16.01) | Very low | 2.5 (0.39-16.01) | Very low |
| 57 | Filgotinib 200 mg QD:Tofacitinib 15 mg BID |  | - |  | Low | 2.12 (0.25-17.91) | Very low | 2.12 (0.25-17.91) | Very low |
| 58 | Filgotinib 200 mg QD:Tofacitinib 3 mg BID |  | - |  | Low | 2.27 (0.18-29.22) | Very low | 2.27 (0.18-29.22) | Very low |
| 59 | Filgotinib 200 mg QD:Tofacitinib 5 mg BID |  | - |  | Low | 1.17 (0.17-8.13) | Low | 1.17 (0.17-8.13) | Low |
| 60 | Filgotinib 200 mg QD:Upadacitinib 15 mg QD |  | - |  | Low | 2.22 (0.21-23.86) | Very low | 2.22 (0.21-23.86) | Very low |
| 61 | Filgotinib 200 mg QD:Upadacitinib 30 mg QD |  | - |  | Low | 0.82 (0.11-6.15) | Low | 0.82 (0.11-6.15) | Low |
| 62 | Filgotinib 200 mg QD:Upadacitinib 45 mg QD |  | - |  | Low | 1.31 (0.19-9.3) | Low | 1.31 (0.19-9.3) | Low |
| 63 | Ivarmacitinib:Izencitinib |  | - |  | Low | 1.02 (0.01-125.8) | Very low | 1.02 (0.01-125.8) | Very low |
| 64 | Ivarmacitinib:Peficitinib |  | - |  | Moderate | 1.37 (0.01-356.53) | Low | 1.37 (0.01-356.53) | Low |
| 65 | Ivarmacitinib:Placebo | Moderate | 0.33 (0.01-16.93) | Low |  | - |  | 0.33 (0.01-16.93) | Low |
| 66 | Ivarmacitinib:Ritlecitinib |  | - |  | Moderate | 0.67 (0-108.58) | Low | 0.67 (0-108.58) | Low |
| 67 | Ivarmacitinib:Tofacitinib 0.5 mg BID |  | - |  | Moderate | 0.8 (0.01-71.36) | Low | 0.8 (0.01-71.36) | Low |
| 68 | Ivarmacitinib:Tofacitinib 1 mg BID |  | - |  | Very low | 0.51 (0.01-51.52) | Very low | 0.51 (0.01-51.52) | Very low |
| 69 | Ivarmacitinib:Tofacitinib 10 mg BID |  | - |  | Low | 0.93 (0.01-58.63) | Very low | 0.93 (0.01-58.63) | Very low |
| 70 | Ivarmacitinib:Tofacitinib 15 mg BID |  | - |  | Low | 0.79 (0.01-56.67) | Very low | 0.79 (0.01-56.67) | Very low |
| 71 | Ivarmacitinib:Tofacitinib 3 mg BID |  | - |  | Moderate | 0.85 (0.01-76.01) | Low | 0.85 (0.01-76.01) | Low |
| 72 | Ivarmacitinib:Tofacitinib 5 mg BID |  | - |  | Low | 0.44 (0.01-28.42) | Very low | 0.44 (0.01-28.42) | Very low |
| 73 | Ivarmacitinib:Upadacitinib 15 mg QD |  | - |  | Low | 0.83 (0.01-67.34) | Very low | 0.83 (0.01-67.34) | Very low |
| 74 | Ivarmacitinib:Upadacitinib 30 mg QD |  | - |  | Low | 0.31 (0-20.67) | Very low | 0.31 (0-20.67) | Very low |
| 75 | Ivarmacitinib:Upadacitinib 45 mg QD |  | - |  | Low | 0.49 (0.01-32.21) | Very low | 0.49 (0.01-32.21) | Very low |
| 76 | Izencitinib:Peficitinib |  | - |  | Low | 1.34 (0.01-166.45) | Very low | 1.34 (0.01-166.45) | Very low |
| 77 | Izencitinib:Placebo | Moderate | 0.33 (0.02-5.26) | Moderate |  | - |  | 0.33 (0.02-5.26) | Moderate |
| 78 | Izencitinib:Ritlecitinib |  | - |  | Low | 0.66 (0.01-46.69) | Very low | 0.66 (0.01-46.69) | Very low |
| 79 | Izencitinib:Tofacitinib 0.5 mg BID |  | - |  | Low | 0.78 (0.02-26.76) | Very low | 0.78 (0.02-26.76) | Very low |
| 80 | Izencitinib:Tofacitinib 1 mg BID |  | - |  | Very low | 0.5 (0.01-19.94) | Very low | 0.5 (0.01-19.94) | Very low |
| 81 | Izencitinib:Tofacitinib 10 mg BID |  | - |  | Low | 0.92 (0.04-19.68) | Very low | 0.92 (0.04-19.68) | Very low |
| 82 | Izencitinib:Tofacitinib 15 mg BID |  | - |  | Low | 0.78 (0.03-19.88) | Very low | 0.78 (0.03-19.88) | Very low |
| 83 | Izencitinib:Tofacitinib 3 mg BID |  | - |  | Low | 0.83 (0.02-28.5) | Very low | 0.83 (0.02-28.5) | Very low |
| 84 | Izencitinib:Tofacitinib 5 mg BID |  | - |  | Low | 0.43 (0.02-9.67) | Low | 0.43 (0.02-9.67) | Low |
| 85 | Izencitinib:Upadacitinib 15 mg QD |  | - |  | Low | 0.82 (0.03-24.54) | Very low | 0.82 (0.03-24.54) | Very low |
| 86 | Izencitinib:Upadacitinib 30 mg QD |  | - |  | Low | 0.3 (0.01-7.11) | Low | 0.3 (0.01-7.11) | Low |
| 87 | Izencitinib:Upadacitinib 45 mg QD |  | - |  | Low | 0.48 (0.02-10.99) | Very low | 0.48 (0.02-10.99) | Very low |
| 88 | Peficitinib:Placebo | Moderate | 0.24 (0-12.38) | Low |  | - |  | 0.24 (0-12.38) | Low |
| 89 | Peficitinib:Ritlecitinib |  | - |  | Moderate | 0.49 (0-79.43) | Low | 0.49 (0-79.43) | Low |
| 90 | Peficitinib:Tofacitinib 0.5 mg BID |  | - |  | Moderate | 0.58 (0.01-52.19) | Low | 0.58 (0.01-52.19) | Low |
| 91 | Peficitinib:Tofacitinib 1 mg BID |  | - |  | Very low | 0.37 (0-37.68) | Very low | 0.37 (0-37.68) | Very low |
| 92 | Peficitinib:Tofacitinib 10 mg BID |  | - |  | Low | 0.68 (0.01-42.87) | Very low | 0.68 (0.01-42.87) | Very low |
| 93 | Peficitinib:Tofacitinib 15 mg BID |  | - |  | Low | 0.58 (0.01-41.44) | Very low | 0.58 (0.01-41.44) | Very low |
| 94 | Peficitinib:Tofacitinib 3 mg BID |  | - |  | Moderate | 0.62 (0.01-55.59) | Low | 0.62 (0.01-55.59) | Low |
| 95 | Peficitinib:Tofacitinib 5 mg BID |  | - |  | Low | 0.32 (0-20.78) | Very low | 0.32 (0-20.78) | Very low |
| 96 | Peficitinib:Upadacitinib 15 mg QD |  | - |  | Low | 0.61 (0.01-49.25) | Very low | 0.61 (0.01-49.25) | Very low |
| 97 | Peficitinib:Upadacitinib 30 mg QD |  | - |  | Low | 0.22 (0-15.12) | Very low | 0.22 (0-15.12) | Very low |
| 98 | Peficitinib:Upadacitinib 45 mg QD |  | - |  | Low | 0.36 (0.01-23.56) | Very low | 0.36 (0.01-23.56) | Very low |
| 99 | Placebo:Ritlecitinib | Moderate | 2.02 (0.08-51.03) | Low |  | - |  | 2.02 (0.08-51.03) | Low |
| 100 | Placebo:Tofacitinib 0.5 mg BID | Moderate | 2.6 (0.28-24.13) | Low | Low | 0.47 (0-11812.4) | Very low | 2.41 (0.27-21.17) | Low |
| 101 | Placebo:Tofacitinib 1 mg BID | Low | 1.06 (0.06-17.28) | Very low | Very low | 4.65 (0.04-567.94) | Very low | 1.54 (0.14-17.21) | Very low |
| 102 | Placebo:Tofacitinib 10 mg BID | Moderate | 2.95 (0.8-10.83) | Low | Low | 0.8 (0-979.59) | Very low | 2.83 (0.79-10.17) | Low |
| 103 | Placebo:Tofacitinib 15 mg BID | Moderate | 2.41 (0.42-13.73) | Low | Low | 2.2 (0.01-543.29) | Very low | 2.39 (0.46-12.56) | Low |
| 104 | Placebo:Tofacitinib 3 mg BID | Moderate | 2.77 (0.3-25.69) | Low | Low | 0.5 (0-12501.05) | Very low | 2.56 (0.29-22.53) | Low |
| 105 | Placebo:Tofacitinib 5 mg BID | Moderate | 0.99 (0.22-4.41) | Moderate | Low | 11.29 (0.19-685.36) | Very low | 1.32 (0.33-5.34) | Moderate |
| 106 | Placebo:Upadacitinib 15 mg QD | Moderate | 1.63 (0.2-13.31) | Low | Low | 41.54 (0.2-8629.56) | Very low | 2.51 (0.36-17.76) | Low |
| 107 | Placebo:Upadacitinib 30 mg QD | Moderate | 0.67 (0.14-3.24) | Moderate | Low | 22.05 (0.16-3096.34) | Very low | 0.93 (0.21-4.15) | Moderate |
| 108 | Placebo:Upadacitinib 45 mg QD | Moderate | 2.14 (0.45-10.21) | Low | Low | 0.24 (0.01-7.74) | Low | 1.48 (0.36-6.15) | Moderate |
| 109 | Ritlecitinib:Tofacitinib 0.5 mg BID |  | - |  | Moderate | 1.19 (0.02-58.41) | Low | 1.19 (0.02-58.41) | Low |
| 110 | Ritlecitinib:Tofacitinib 1 mg BID |  | - |  | Very low | 0.76 (0.01-42.94) | Very low | 0.76 (0.01-42.94) | Very low |
| 111 | Ritlecitinib:Tofacitinib 10 mg BID |  | - |  | Low | 1.4 (0.04-45.12) | Very low | 1.4 (0.04-45.12) | Very low |
| 112 | Ritlecitinib:Tofacitinib 15 mg BID |  | - |  | Low | 1.19 (0.03-44.68) | Very low | 1.19 (0.03-44.68) | Very low |
| 113 | Ritlecitinib:Tofacitinib 3 mg BID |  | - |  | Moderate | 1.27 (0.03-62.21) | Low | 1.27 (0.03-62.21) | Low |
| 114 | Ritlecitinib:Tofacitinib 5 mg BID |  | - |  | Low | 0.65 (0.02-22.03) | Very low | 0.65 (0.02-22.03) | Very low |
| 115 | Ritlecitinib:Upadacitinib 15 mg QD |  | - |  | Low | 1.24 (0.03-54.23) | Very low | 1.24 (0.03-54.23) | Very low |
| 116 | Ritlecitinib:Upadacitinib 30 mg QD |  | - |  | Low | 0.46 (0.01-16.13) | Very low | 0.46 (0.01-16.13) | Very low |
| 117 | Ritlecitinib:Upadacitinib 45 mg QD |  | - |  | Low | 0.73 (0.02-25.01) | Very low | 0.73 (0.02-25.01) | Very low |
| 118 | Tofacitinib 0.5 mg BID:Tofacitinib 1 mg BID |  | - |  | Very low | 0.64 (0.03-15.35) | Very low | 0.64 (0.03-15.35) | Very low |
| 119 | Tofacitinib 0.5 mg BID:Tofacitinib 10 mg BID | Moderate | 1.07 (0.07-17.41) | Low | Low | 1.53 (0.02-151.77) | Very low | 1.18 (0.11-12.79) | Low |
| 120 | Tofacitinib 0.5 mg BID:Tofacitinib 15 mg BID | Moderate | 1.59 (0.1-25.9) | Low | Low | 0.11 (0-46.71) | Very low | 1 (0.08-12.54) | Low |
| 121 | Tofacitinib 0.5 mg BID:Tofacitinib 3 mg BID | Moderate | 1.07 (0.07-17.41) | Low |  | - |  | 1.07 (0.07-17.41) | Low |
| 122 | Tofacitinib 0.5 mg BID:Tofacitinib 5 mg BID |  | - |  | Low | 0.55 (0.04-6.83) | Low | 0.55 (0.04-6.83) | Low |
| 123 | Tofacitinib 0.5 mg BID:Upadacitinib 15 mg QD |  | - |  | Low | 1.05 (0.06-19.46) | Very low | 1.05 (0.06-19.46) | Very low |
| 124 | Tofacitinib 0.5 mg BID:Upadacitinib 30 mg QD |  | - |  | Low | 0.38 (0.03-5.41) | Low | 0.38 (0.03-5.41) | Low |
| 125 | Tofacitinib 0.5 mg BID:Upadacitinib 45 mg QD |  | - |  | Low | 0.62 (0.05-8.29) | Low | 0.62 (0.05-8.29) | Low |
| 126 | Tofacitinib 1 mg BID:Tofacitinib 10 mg BID |  | - |  | Very low | 1.84 (0.13-25.72) | Very low | 1.84 (0.13-25.72) | Very low |
| 127 | Tofacitinib 1 mg BID:Tofacitinib 15 mg BID | Low | 0.97 (0.06-15.85) | Very low | Very low | 13.92 (0.03-5768.77) | Very low | 1.56 (0.12-19.58) | Very low |
| 128 | Tofacitinib 1 mg BID:Tofacitinib 3 mg BID |  | - |  | Very low | 1.66 (0.07-39.9) | Very low | 1.66 (0.07-39.9) | Very low |
| 129 | Tofacitinib 1 mg BID:Tofacitinib 5 mg BID | Low | 0.94 (0.06-15.39) | Very low | Very low | 0.6 (0-132.7) | Very low | 0.86 (0.07-10.23) | Very low |
| 130 | Tofacitinib 1 mg BID:Upadacitinib 15 mg QD |  | - |  | Very low | 1.63 (0.07-36.48) | Very low | 1.63 (0.07-36.48) | Very low |
| 131 | Tofacitinib 1 mg BID:Upadacitinib 30 mg QD |  | - |  | Very low | 0.6 (0.04-10.32) | Very low | 0.6 (0.04-10.32) | Very low |
| 132 | Tofacitinib 1 mg BID:Upadacitinib 45 mg QD |  | - |  | Very low | 0.96 (0.06-15.87) | Very low | 0.96 (0.06-15.87) | Very low |
| 133 | Tofacitinib 10 mg BID:Tofacitinib 15 mg BID | Moderate | 1.49 (0.09-24.29) | Low | Low | 0.49 (0.03-7.54) | Low | 0.85 (0.12-5.95) | Moderate |
| 134 | Tofacitinib 10 mg BID:Tofacitinib 3 mg BID | Moderate | 1 (0.06-16.33) | Low | Low | 0.7 (0.01-68.76) | Very low | 0.91 (0.08-9.86) | Moderate |
| 135 | Tofacitinib 10 mg BID:Tofacitinib 5 mg BID | Moderate | 0.62 (0.08-5.09) | Moderate | Low | 0.3 (0.02-4.09) | Low | 0.47 (0.09-2.4) | Moderate |
| 136 | Tofacitinib 10 mg BID:Upadacitinib 15 mg QD |  | - |  | Low | 0.89 (0.09-9.2) | Low | 0.89 (0.09-9.2) | Low |
| 137 | Tofacitinib 10 mg BID:Upadacitinib 30 mg QD |  | - |  | Low | 0.33 (0.05-2.36) | Low | 0.33 (0.05-2.36) | Low |
| 138 | Tofacitinib 10 mg BID:Upadacitinib 45 mg QD |  | - |  | Low | 0.52 (0.08-3.56) | Low | 0.52 (0.08-3.56) | Low |
| 139 | Tofacitinib 15 mg BID:Tofacitinib 3 mg BID | Moderate | 0.67 (0.04-10.91) | Low | Low | 9.67 (0.02-4090.65) | Very low | 1.07 (0.09-13.48) | Low |
| 140 | Tofacitinib 15 mg BID:Tofacitinib 5 mg BID | Low | 0.97 (0.06-15.84) | Very low | Low | 0.33 (0.02-4.74) | Low | 0.55 (0.08-3.8) | Low |
| 141 | Tofacitinib 15 mg BID:Upadacitinib 15 mg QD |  | - |  | Low | 1.05 (0.08-13.63) | Very low | 1.05 (0.08-13.63) | Very low |
| 142 | Tofacitinib 15 mg BID:Upadacitinib 30 mg QD |  | - |  | Low | 0.39 (0.04-3.62) | Low | 0.39 (0.04-3.62) | Low |
| 143 | Tofacitinib 15 mg BID:Upadacitinib 45 mg QD |  | - |  | Moderate | 0.62 (0.07-5.5) | Moderate | 0.62 (0.07-5.5) | Moderate |
| 144 | Tofacitinib 3 mg BID:Tofacitinib 5 mg BID |  | - |  | Low | 0.51 (0.04-6.4) | Low | 0.51 (0.04-6.4) | Low |
| 145 | Tofacitinib 3 mg BID:Upadacitinib 15 mg QD |  | - |  | Low | 0.98 (0.05-18.25) | Very low | 0.98 (0.05-18.25) | Very low |
| 146 | Tofacitinib 3 mg BID:Upadacitinib 30 mg QD |  | - |  | Low | 0.36 (0.03-5.07) | Low | 0.36 (0.03-5.07) | Low |
| 147 | Tofacitinib 3 mg BID:Upadacitinib 45 mg QD |  | - |  | Low | 0.58 (0.04-7.78) | Low | 0.58 (0.04-7.78) | Low |
| 148 | Tofacitinib 5 mg BID:Upadacitinib 15 mg QD |  | - |  | Low | 1.91 (0.17-21.11) | Very low | 1.91 (0.17-21.11) | Very low |
| 149 | Tofacitinib 5 mg BID:Upadacitinib 30 mg QD |  | - |  | Low | 0.7 (0.09-5.47) | Low | 0.7 (0.09-5.47) | Low |
| 150 | Tofacitinib 5 mg BID:Upadacitinib 45 mg QD |  | - |  | Low | 1.12 (0.15-8.28) | Low | 1.12 (0.15-8.28) | Low |
| 151 | Upadacitinib 15 mg QD:Upadacitinib 30 mg QD | Moderate | 0.45 (0.07-3.06) | Moderate | Low | 0 (0-47.29) | Very low | 0.37 (0.06-2.44) | Moderate |
| 152 | Upadacitinib 15 mg QD:Upadacitinib 45 mg QD | Moderate | 0.5 (0.02-10.54) | Low | Low | 0.69 (0.04-11.9) | Very low | 0.59 (0.07-4.75) | Moderate |
| 153 | Upadacitinib 30 mg QD:Upadacitinib 45 mg QD | Moderate | 0.21 (0.01-4.36) | Moderate | Low | 4.8 (0.52-44.73) | Very low | 1.6 (0.26-9.69) | Moderate |

NMA: network meta-analysis; CVE: cardiovascular events; GRADE: Grading of Recommendations, Assessment, Development and Evaluation; OR: odds ratio; CI: confidence interval; QD: once daily; BID: twice daily.

**Supplementary table 12-1 P-scores and SUCRA values for MACE.**

|  | Intervention | P-score | Intervention | SUCRA value |
| --- | --- | --- | --- | --- |
| MACE (without dose consideration) | Placebo | 0.821 | Placebo | 0.815 |
|  | Ritlecitinib | 0.586 | Ritlecitinib | 0.581 |
|  | Filgotinib | 0.531 | Filgotinib | 0.532 |
|  | Ivarmacitinib | 0.499 | Ivarmacitinib | 0.498 |
|  | Izencitinib | 0.490 | Izencitinib | 0.491 |
|  | Upadacitinib | 0.489 | Upadacitinib | 0.491 |
|  | Tofacitinib | 0.452 | Tofacitinib | 0.454 |
|  | Peficitinib | 0.444 | Peficitinib | 0.444 |
|  | Brepocitinib | 0.377 | Brepocitinib | 0.381 |
|  | Deucravacitinib | 0.313 | Deucravacitinib | 0.314 |
|  | Intervention | P-score | Intervention | SUCRA value |
| MACE  (with dose consideration) | Placebo | 0.721 | Placebo | 0.720 |
|  | Tofacitinib 5 mg BID | 0.705 | Tofacitinib 5 mg BID | 0.704 |
|  | Tofacitinib 1 mg BID | 0.605 | Tofacitinib 1 mg BID | 0.605 |
|  | Filgotinib 200 mg QD | 0.592 | Filgotinib 200 mg QD | 0.592 |
|  | ***Upadacitinib 15 mg QD**** | ***0.556**** | ***Filgotinib 100 mg QD**** | ***0.555**** |
|  | ***Filgotinib 100 mg QD**** | ***0.552**** | ***Upadacitinib 15 mg QD**** | ***0.554**** |
|  | Tofacitinib 0.5 mg BID | 0.530 | Tofacitinib 0.5 mg BID | 0.531 |
|  | Tofacitinib 3 mg BID | 0.513 | Tofacitinib 3 mg BID | 0.514 |
|  | Tofacitinib 15 mg BID | 0.507 | Tofacitinib 15 mg BID | 0.509 |
|  | ***Ritlecitinib**** | ***0.502**** | ***Tofacitinib 10 mg BID**** | ***0.502**** |
|  | ***Tofacitinib 10 mg BID**** | ***0.500**** | ***Ritlecitinib**** | ***0.498**** |
|  | Upadacitinib 30 mg QD | 0.470 | Upadacitinib 30 mg QD | 0.471 |
|  | Upadacitinib 45 mg QD | 0.460 | Upadacitinib 45 mg QD | 0.461 |
|  | Ivarmacitinib | 0.430 | Ivarmacitinib | 0.430 |
|  | Izencitinib | 0.404 | Izencitinib | 0.403 |
|  | Peficitinib | 0.377 | Peficitinib | 0.378 |
|  | Brepocitinib | 0.319 | Brepocitinib | 0.320 |
|  | Deucravacitinib | 0.257 | Deucravacitinib | 0.255 |

SUCRA: the surface under the cumulative ranking curve; MACE: major adverse cardiovascular events; QD: once daily; BID: twice daily. Bold italics with an asterisk (*) indicate different interventions rankings based on P-scores and SCURA values.

**Supplementary table 12-2 P-scores and SUCRA values for VTE.**

|  | Intervention | P-score | Intervention | SUCRA value |
| --- | --- | --- | --- | --- |
| VTE  (without dose consideration) | Placebo | 0.829 | Placebo | 0.824 |
|  | Upadacitinib | 0.685 | Upadacitinib | 0.687 |
|  | Filgotinib | 0.599 | Filgotinib | 0.602 |
|  | Ivarmacitinib | 0.525 | Ivarmacitinib | 0.525 |
|  | Izencitinib | 0.518 | Izencitinib | 0.520 |
|  | Peficitinib | 0.471 | Peficitinib | 0.470 |
|  | Brepocitinib | 0.416 | Brepocitinib | 0.413 |
|  | ***Ritlecitinib**** | ***0.406**** | ***Tofacitinib**** | ***0.405**** |
|  | ***Tofacitinib**** | ***0.400**** | ***Ritlecitinib**** | ***0.402**** |
|  | Deucravacitinib | 0.152 | Deucravacitinib | 0.153 |
|  | Intervention | P-score | Intervention | SUCRA value |
| VTE  (with dose consideration) | Upadacitinib 30 mg QD | 0.766 | Upadacitinib 30 mg QD | 0.763 |
|  | Placebo | 0.722 | Placebo | 0.722 |
|  | Filgotinib 200 mg QD | 0.663 | Filgotinib 200 mg QD | 0.661 |
|  | Upadacitinib 45 mg QD | 0.596 | Upadacitinib 45 mg QD | 0.600 |
|  | Tofacitinib 0.5 mg BID | 0.575 | Tofacitinib 0.5 mg BID | 0.575 |
|  | Tofacitinib 1 mg BID | 0.567 | Tofacitinib 1 mg BID | 0.567 |
|  | Tofacitinib 3 mg BID | 0.559 | Tofacitinib 3 mg BID | 0.560 |
|  | Filgotinib 100 mg QD | 0.548 | Filgotinib 100 mg QD | 0.553 |
|  | Tofacitinib 5 mg BID | 0.545 | Tofacitinib 5 mg BID | 0.543 |
|  | Tofacitinib 15 mg BID | 0.511 | Tofacitinib 15 mg BID | 0.511 |
|  | Upadacitinib 15 mg QD | 0.484 | Upadacitinib 15 mg QD | 0.491 |
|  | Ivarmacitinib | 0.442 | Ivarmacitinib | 0.444 |
|  | Tofacitinib 10 mg BID | 0.441 | Tofacitinib 10 mg BID | 0.443 |
|  | Izencitinib | 0.417 | Izencitinib | 0.417 |
|  | Peficitinib | 0.391 | Peficitinib | 0.390 |
|  | Brepocitinib | 0.338 | Brepocitinib | 0.332 |
|  | Ritlecitinib | 0.329 | Ritlecitinib | 0.324 |
|  | Deucravacitinib | 0.105 | Deucravacitinib | 0.105 |

SUCRA; the surface under the cumulative ranking curve; VTE: venous thromboembolism events; QD: once daily; BID: twice daily. Bold italics with an asterisk (*) indicate different interventions rankings based on P-scores and SCURA values.

**Supplementary table 12-3 P-scores and SUCRA values for CVE.**

|  | Intervention | P-score | Intervention | SUCRA value |
| --- | --- | --- | --- | --- |
| CVE  (without dose consideration) | Placebo | 0.821 | Placebo | 0.815 |
|  | Upadacitinib | 0.638 | Upadacitinib | 0.639 |
|  | Ritlecitinib | 0.594 | Ritlecitinib | 0.586 |
|  | Filgotinib | 0.581 | Filgotinib | 0.583 |
|  | Ivarmacitinib | 0.508 | Ivarmacitinib | 0.509 |
|  | Izencitinib | 0.499 | Izencitinib | 0.500 |
|  | Peficitinib | 0.455 | Peficitinib | 0.455 |
|  | Brepocitinib | 0.389 | Brepocitinib | 0.393 |
|  | Tofacitinib | 0.379 | Tofacitinib | 0.383 |
|  | Deucravacitinib | 0.136 | Deucravacitinib | 0.136 |
|  | Intervention | P-score | Intervention | SUCRA value |
| CVE  (with dose consideration) | Placebo | 0.745 | Placebo | 0.745 |
|  | Upadacitinib 30 mg QD | 0.736 | Upadacitinib 30 mg QD | 0.732 |
|  | Filgotinib 200 mg QD | 0.682 | Filgotinib 200 mg QD | 0.680 |
|  | Tofacitinib 5 mg BID | 0.636 | Tofacitinib 5 mg BID | 0.635 |
|  | Upadacitinib 45 mg QD | 0.597 | Upadacitinib 45 mg QD | 0.599 |
|  | Tofacitinib 1 mg BID | 0.580 | Tofacitinib 1 mg BID | 0.578 |
|  | Filgotinib 100 mg QD | 0.564 | Filgotinib 100 mg QD | 0.570 |
|  | Ritlecitinib | 0.522 | Ritlecitinib | 0.517 |
|  | Tofacitinib 0.5 mg BID | 0.469 | Tofacitinib 0.5 mg BID | 0.468 |
|  | Tofacitinib 15 mg BID | 0.458 | Tofacitinib 15 mg BID | 0.458 |
|  | Tofacitinib 3 mg BID | 0.453 | Tofacitinib 3 mg BID | 0.453 |
|  | ***Ivarmacitinib**** | ***0.449**** | ***Upadacitinib 15 mg QD**** | ***0.452**** |
|  | ***Upadacitinib 15 mg QD**** | ***0.448**** | ***Ivarmacitinib**** | ***0.452**** |
|  | Izencitinib | 0.426 | Izencitinib | 0.426 |
|  | Tofacitinib 10 mg BID | 0.400 | Tofacitinib 10 mg BID | 0.402 |
|  | Peficitinib | 0.396 | Peficitinib | 0.395 |
|  | Brepocitinib | 0.337 | Brepocitinib | 0.336 |
|  | Deucravacitinib | 0.102 | Deucravacitinib | 0.103 |

SUCRA: the surface under the cumulative ranking curve; CVE: cardiovascular events; QD: once daily; BID: twice daily. Bold italics with an asterisk (*) indicate different interventions rankings based on P-scores and SCURA values.

**Supplementary table 13 Tests of heterogeneity (within designs) and inconsistency (between designs) with Q statistics.**

|  | (without dose consideration) | | | (with dose consideration) | | |
| --- | --- | --- | --- | --- | --- | --- |
|  | Q statistics | Degrees of freedom | P-value | Q statistics | Degrees of freedom | P-value |
| Major adverse cardiovascular events | | | | | | |
| Total | 2.84 | 22 | 1.00 | 2.23 | 37 | 1.00 |
| Within designs | 2.84 | 22 | 1.00 | 0.98 | 29 | 1.00 |
| Between designs | 0.00 | 0 | NA | 1.25 | 8 | 1.00 |
| Venous thromboembolism events | | | | | | |
| Total | 3.96 | 22 | 1.00 | 7.89 | 37 | 1.00 |
| Within designs | 3.96 | 22 | 1.00 | 2.33 | 29 | 1.00 |
| Between designs | 0.00 | 0 | NA | 5.56 | 8 | 0.70 |
| Cardiovascular events | | | | | | |
| Total | 4.31 | 22 | 1.00 | 7.09 | 37 | 1.00 |
| Within designs | 4.31 | 22 | 1.00 | 2.15 | 29 | 1.00 |
| Between designs | 0.00 | 0 | NA | 4.94 | 8 | 0.76 |
