# Supplementary material for: Cardiovascular safety of Janus kinase inhibitors in inflammatory bowel disease: a systematic review and network meta-analysis
Source: Ann Med. 2025 Jan 21;57(1):2455536. doi: 10.1080/07853890.2025.2455536 (PMC11755742; doi:10.1080/07853890.2025.2455536)

**Cardiovascular safety of Janus kinase inhibitors in inflammatory bowel disease: a systematic review and network meta-analysis**

**Huibin Yang, Ting An, Yuxuan Zhao, Xiaojing Shi, Bangmao Wang, Qingyu Zhang**

**Tianjin Medical University General Hospital**

**e-mail address: (Huibin Yang)** [**Bryan_yang323@163.com**](mailto:Bryan_yang323@163.com)**; (Qingyu Zhang)** [**zhangqy@tmu.edu.cn**](mailto:zhangqy@tmu.edu.cn)

**Contents**

**Supplementary figure 1 Box plots of transitivity assessment (without dose consideration)4**

Supplementary figure 1a Box plots of transitivity assessment (without dose consideration) for the mean age of participants4

Supplementary figure 1b Box plots of transitivity assessment (without dose consideration) for the percentage of female participants4

Supplementary figure 1c Box plots of transitivity assessment (without dose consideration) for the mean disease duration of participants5

Supplementary figure 1d Box plots of transitivity assessment (without dose consideration) for the study duration5

**Supplementary figure 2 Box plots of transitivity assessment (with dose consideration)6**

Supplementary figure 2a Box plots of transitivity assessment (with dose consideration) for the mean age of participants6

Supplementary figure 2b Box plots of transitivity assessment (with dose consideration) for the percentage of female participants6

Supplementary figure 2c Box plots of transitivity assessment (with dose consideration) for the mean disease duration of participants7

Supplementary figure 2d Box plots of transitivity assessment (with dose consideration) for the study duration7

**Supplementary figure 3 The summary proportions of the risk of bias8**

**Supplementary figure 4 The details of the risk of bias8**

**Supplementary figure 5 Forest plots of comparisons for each included RCT (without dose consideration)9**

Supplementary figure 5a Forest plots of comparisons for each included RCT (without dose consideration) for MACE9

Supplementary figure 5b Forest plots of comparisons for each included RCT (without dose consideration) for VTE10

Supplementary figure 5c Forest plots of comparisons for each included RCT (without dose consideration) for CVE11

**Supplementary figure 6 Forest plots of comparisons for each included RCT (with dose consideration)12**

Supplementary figure 6a Forest plots of comparisons for each included RCT (with dose consideration) for MACE12

Supplementary figure 6b Forest plots of comparisons for each included RCT (with dose consideration) for VTE13

Supplementary figure 6c Forest plots of comparisons for each included RCT (with dose consideration) for CVE14

**Supplementary figure 7 Evidence proportion plots of network estimates (without dose consideration)15**

**Supplementary figure 8 Evidence proportion plots of network estimates (with dose consideration)16**

Supplementary figure 8a Evidence proportion plots of network estimates (with dose consideration) for MACE16

Supplementary figure 8b Evidence proportion plots of network estimates (with dose consideration) for VTE17

Supplementary figure 8c Evidence proportion plots of network estimates (with dose consideration) for CVE17

Supplementary figure 8d Evidence proportion plots (no direct comparison) of network estimates (with dose consideration)18

**Supplementary figure 9 Rankogram plots of all interventions (without dose consideration)19**

Supplementary figure 9a Rankogram plots of all interventions (without dose consideration) for MACE19

Supplementary figure 9b Rankogram plots of all interventions (without dose consideration) for VTE19

Supplementary figure 9c Rankogram plots of all interventions (without dose consideration) for CVE20

**Supplementary figure 10 Rankogram plots of all interventions (with dose consideration)20**

Supplementary figure 10a Rankogram plots of all interventions (with dose consideration) for MACE20

Supplementary figure 10b Rankogram plots of all interventions (with dose consideration) for VTE21

Supplementary figure 10c Rankogram plots of all interventions (with dose consideration) for CVE21

**Supplementary figure 11 Cumulative ranking plots of all interventions (without dose consideration)22**

Supplementary figure 11a Cumulative ranking plots of all interventions (without dose consideration) for MACE22

Supplementary figure 11b Cumulative ranking plots of all interventions (without dose consideration) for VTE22

Supplementary figure 11c Cumulative ranking plots of all interventions (without dose consideration) for CVE 23

**Supplementary figure 12 Cumulative ranking plots of all interventions** **(with dose consideration)23**

Supplementary figure 12a Cumulative ranking plots of all interventions (with dose consideration) for MACE23

Supplementary figure 12b Cumulative ranking plots of all interventions (with dose consideration) for VTE24

Supplementary figure 12c Cumulative ranking plots of all interventions (with dose consideration) for CVE24

**Supplementary figure 13 Funnel plots and Egger’s tests of the included studies (without dose consideration)25**

Supplementary figure 13a The Funnel plot and Egger’s test of the included studies (without dose consideration) for MACE25

Supplementary figure 13b The Funnel plot and Egger’s test of the included studies (without dose consideration) for VTE25

Supplementary figure 13c The Funnel plot and Egger’s test of the included studies (without dose consideration) for CVE26

**Supplementary figure 14 Funnel plots and Egger’s tests of the included studies (with dose consideration)26**

Supplementary figure 14a The Funnel plot and Egger’s test of the included studies (with dose consideration) for MACE26

Supplementary figure 14b The Funnel plot and Egger’s test of the included studies (with dose consideration) for VTE27

Supplementary figure 14c The Funnel plot and Egger’s test of the included studies (with dose consideration) for CVE27

**Supplementary figure 15 Local inconsistency analyses of comparisons (without dose consideration)28**

Supplementary figure 15a Local inconsistency analyses of comparisons (without dose consideration) for MACE28

Supplementary figure 15b Local inconsistency analyses of comparisons (without dose consideration) for VTE29

Supplementary figure 15c Local inconsistency analyses of comparisons (without dose consideration) for CVE29

**Supplementary figure 16 Local inconsistency analyses of comparisons (with dose consideration)30**

Supplementary figure 16a Local inconsistency analyses of comparisons (with dose consideration) for MACE30

Supplementary figure 16b Local inconsistency analyses of comparisons (with dose consideration) for VTE31

Supplementary figure 16c Local inconsistency analyses of comparisons (with dose consideration) for CVE32

**Supplementary figure 17 Forest plots of the sensitivity analysis (no high risk of bias trial)33**

Supplementary figure 17a Forest plots of the sensitivity analysis (no high risk of bias trial) (without dose consideration) for MACE33

Supplementary figure 17b Forest plots of the sensitivity analysis (no high risk of bias trial) (without dose consideration) for VTE33

Supplementary figure 17c Forest plots of the sensitivity analysis (no high risk of bias trial) (without dose consideration) for CVE34

Supplementary figure 17d Forest plots of the sensitivity analysis (no high risk of bias trial) (with dose consideration) for MACE34

Supplementary figure 17e Forest plots of the sensitivity analysis (no high risk of bias trial) (with dose consideration) for VTE35

Supplementary figure 17f Forest plots of the sensitivity analysis (no high risk of bias trial) (with dose consideration) for CVE35

**Supplementary figure 18 Forest plots of the subgroup analysis (UC patients) based on the IBD type36**

Supplementary figure 18a Forest plots of the subgroup analysis (UC patients) (without dose consideration) for MACE.36

Supplementary figure 18b Forest plots of the subgroup analysis (UC patients) (without dose consideration) for VTE36

Supplementary figure 18c Forest plots of the subgroup analysis (UC patients) (without dose consideration) for CVE37

Supplementary figure 18d Forest plots of the subgroup analysis (UC patients) (with dose consideration) for MACE37

Supplementary figure 18e Forest plots of the subgroup analysis (UC patients) (with dose consideration) for VTE38

Supplementary figure 18f Forest plots of the subgroup analysis (UC patients) (with dose consideration) for CVE38

**Supplementary figure 19 Forest plots of the subgroup analysis (CD patients) based on the IBD type39**

Supplementary figure 19a Forest plots of the subgroup analysis (CD patients) (without dose consideration) for MACE.39

Supplementary figure 19b Forest plots of the subgroup analysis (CD patients) (without dose consideration) for VTE39

Supplementary figure 19c Forest plots of the subgroup analysis (CD patients) (without dose consideration) for CVE39

Supplementary figure 19d Forest plots of the subgroup analysis (CD patients) (with dose consideration) for MACE40

Supplementary figure 19e Forest plots of the subgroup analysis (CD patients) (with dose consideration) for VTE40

Supplementary figure 19f Forest plots of the subgroup analysis (CD patients) (with dose consideration) for CVE41

**Supplementary figure 20 Forest plot of the subgroup analysis (the induction phase) based on the study phase42**

Supplementary figure 20a Forest plots of the subgroup analysis (the induction phase) (without dose consideration) for MACE.42

Supplementary figure 20b Forest plots of the subgroup analysis (the induction phase) (without dose consideration) for VTE42

Supplementary figure 20c Forest plots of the subgroup analysis (the induction phase) (without dose consideration) for CVE43

Supplementary figure 20d Forest plots of the subgroup analysis (the induction phase) (with dose consideration) for MACE43

Supplementary figure 20e Forest plots of the subgroup analysis (the induction phase) (with dose consideration) for VTE44

Supplementary figure 20f Forest plots of the subgroup analysis (the induction phase) (with dose consideration) for CVE44

**Supplementary figure 21 Forest plots of the subgroup analysis (the maintenance** **phase) based on the study phase45**

Supplementary figure 21a Forest plots of the subgroup analysis (the maintenance phase) (without dose consideration) for MACE.45

Supplementary figure 21b Forest plots of the subgroup analysis (the maintenance phase) (without dose consideration) for VTE45

Supplementary figure 21c Forest plots of the subgroup analysis (the maintenance phase) (without dose consideration) for CVE45

Supplementary figure 21d Forest plots of the subgroup analysis (the maintenance phase) (with dose consideration) for MACE46

Supplementary figure 21e Forest plots of the subgroup analysis (the maintenance phase) (with dose consideration) for VTE46

Supplementary figure 21f Forest plots of the subgroup analysis (the maintenance phase) (with dose consideration) for CVE46

**Supplementary figure 1** **Box plots of transitivity assessment (without dose consideration) for the mean age of participants (a), the percentage of female participants (b), the mean disease duration of participants (c) and the study duration (d)**

**
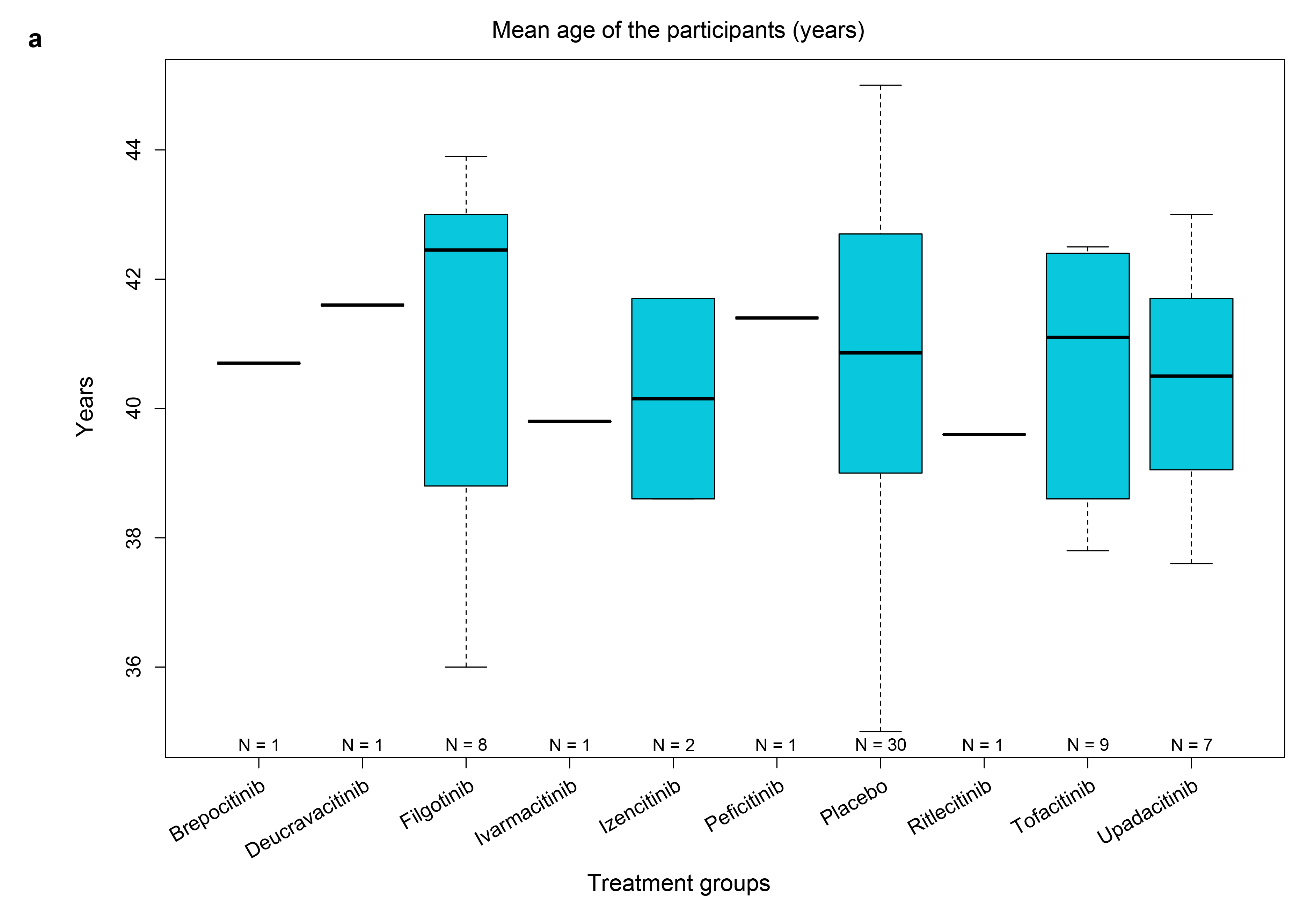
**

**
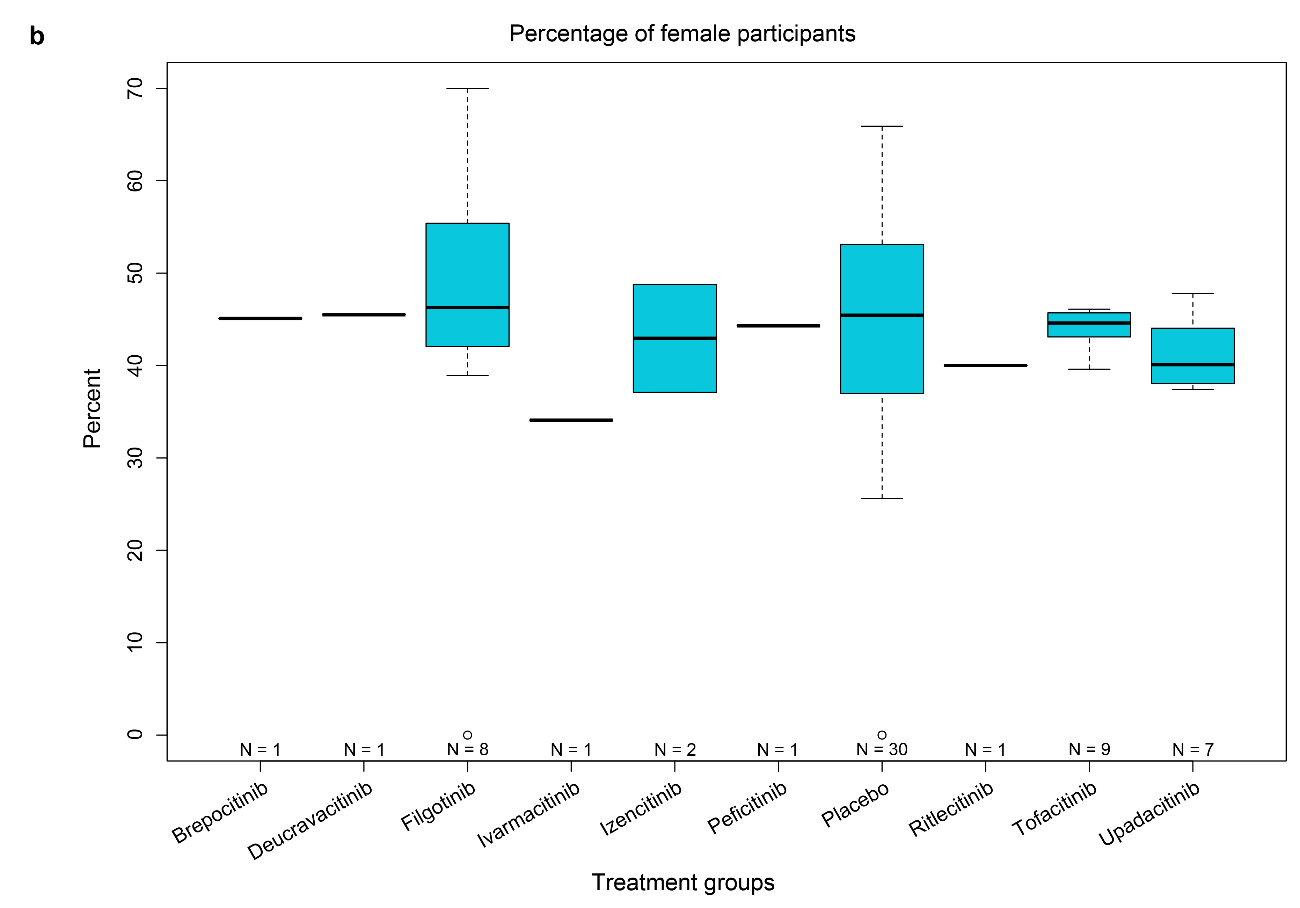
**

**
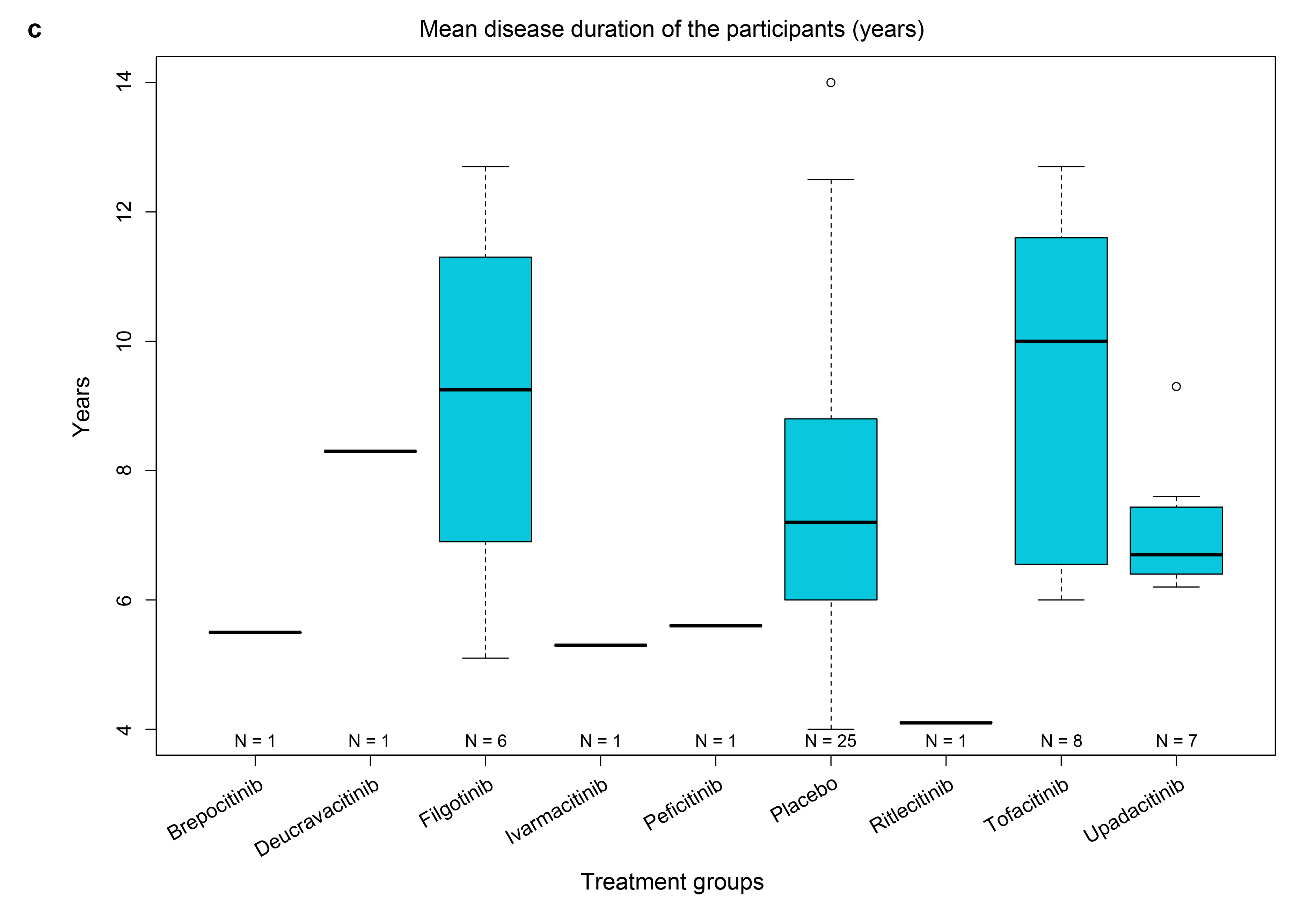
**

**
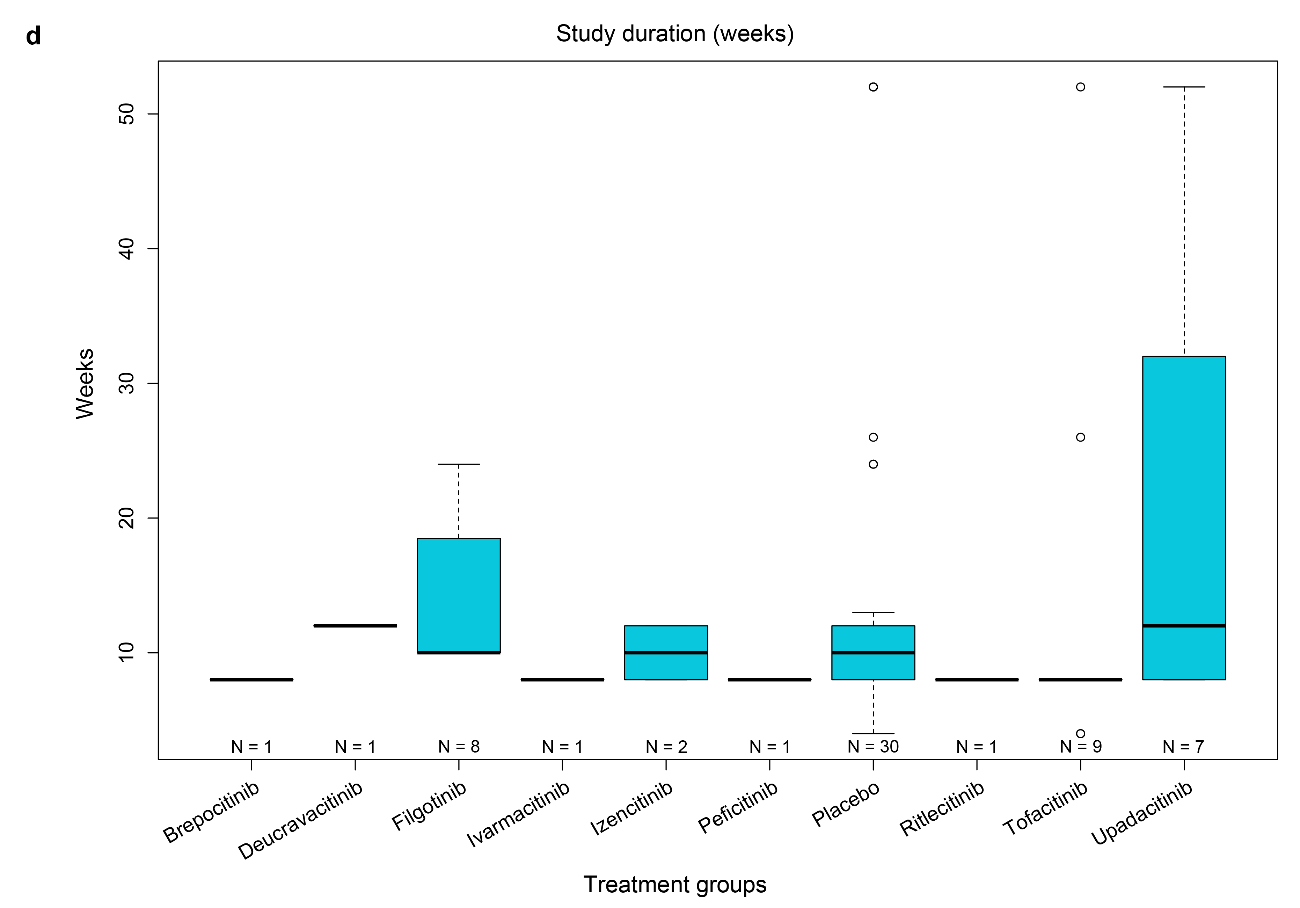
**

**Supplementary figure 2 Box plots of transitivity assessment (with dose consideration) for the mean age** **of participants (a), the percentage of female participants (b), the mean disease duration of participants (c) and the study duration (d)**

**
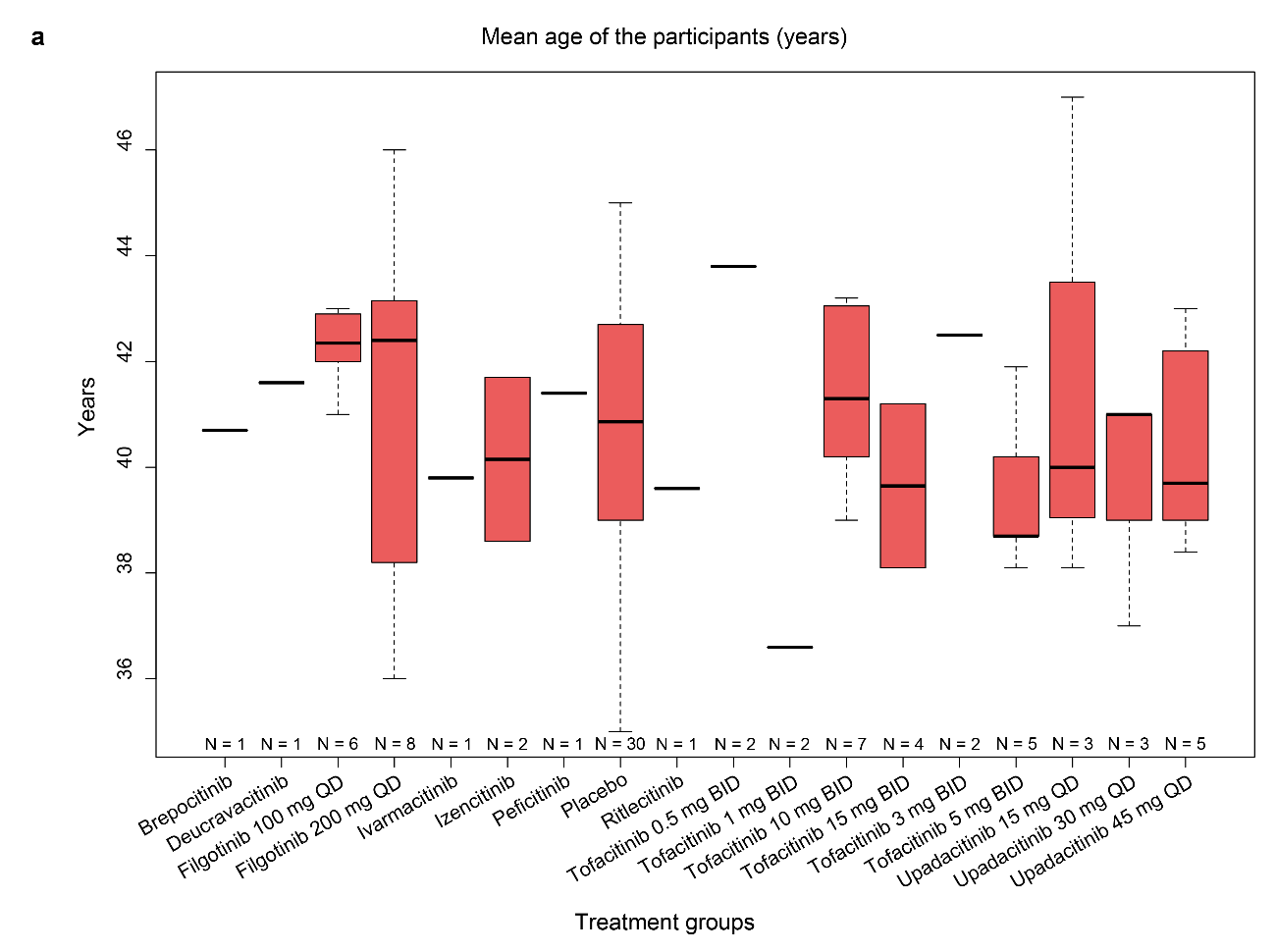

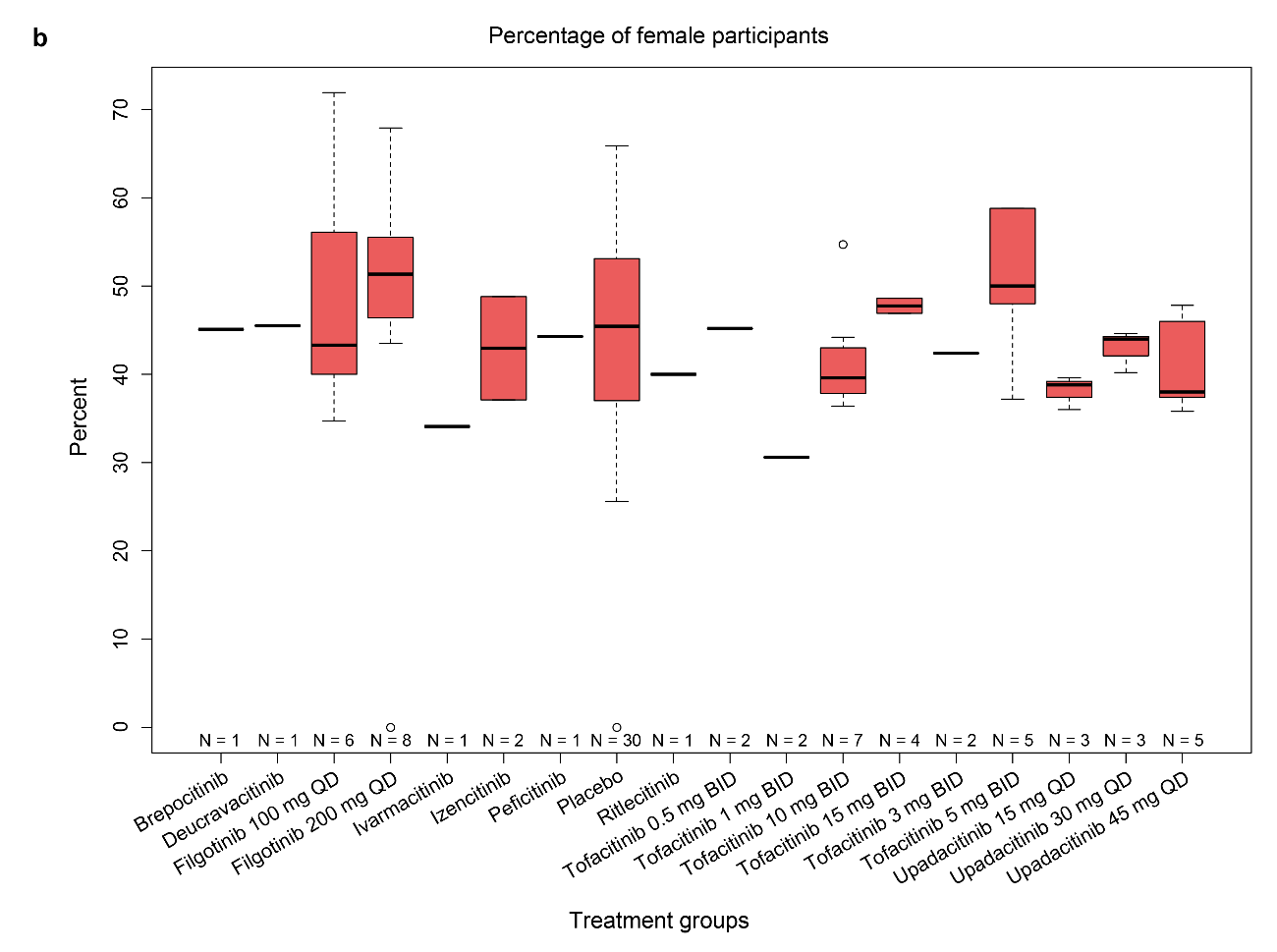
**

**
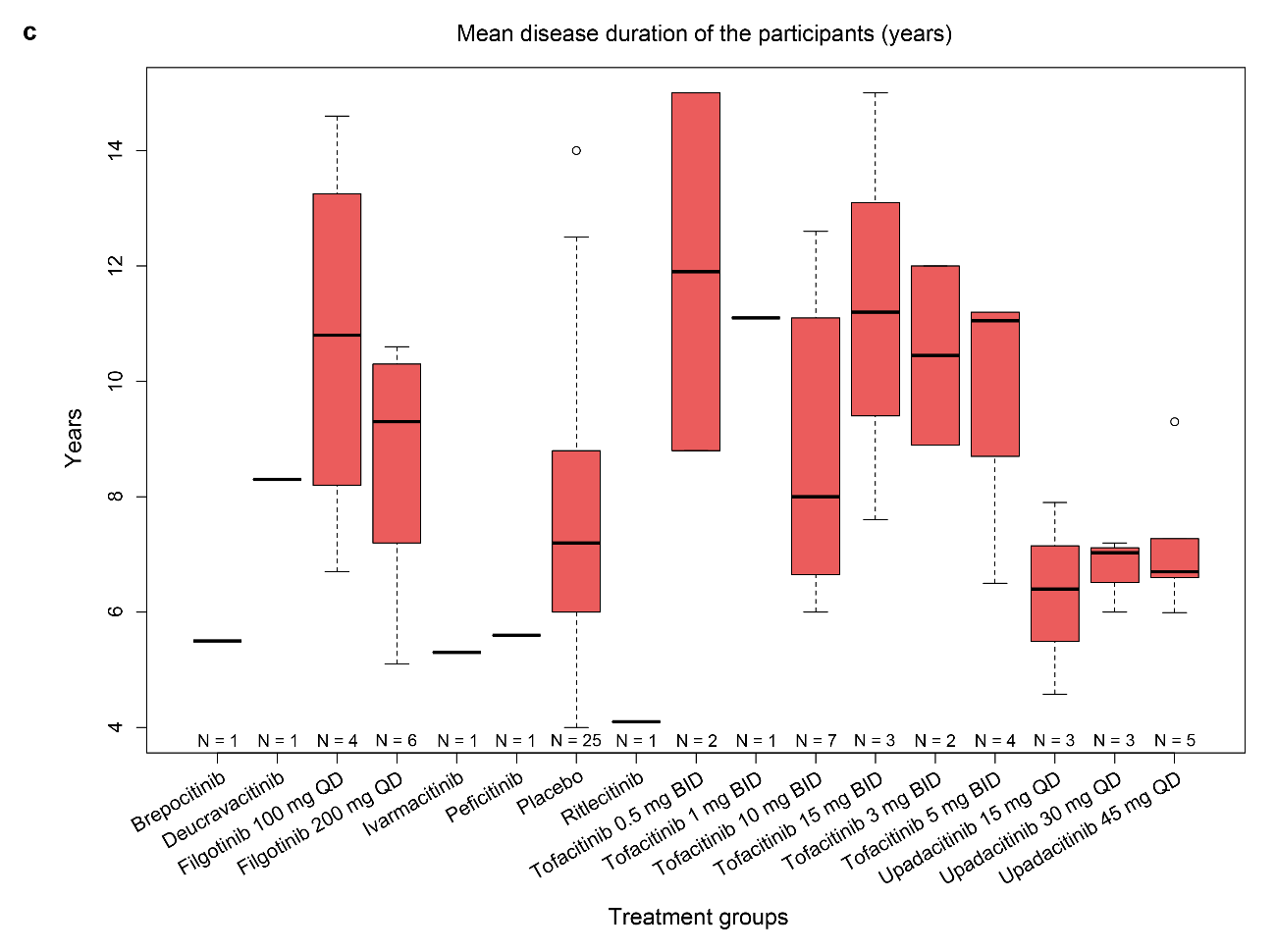
**

**
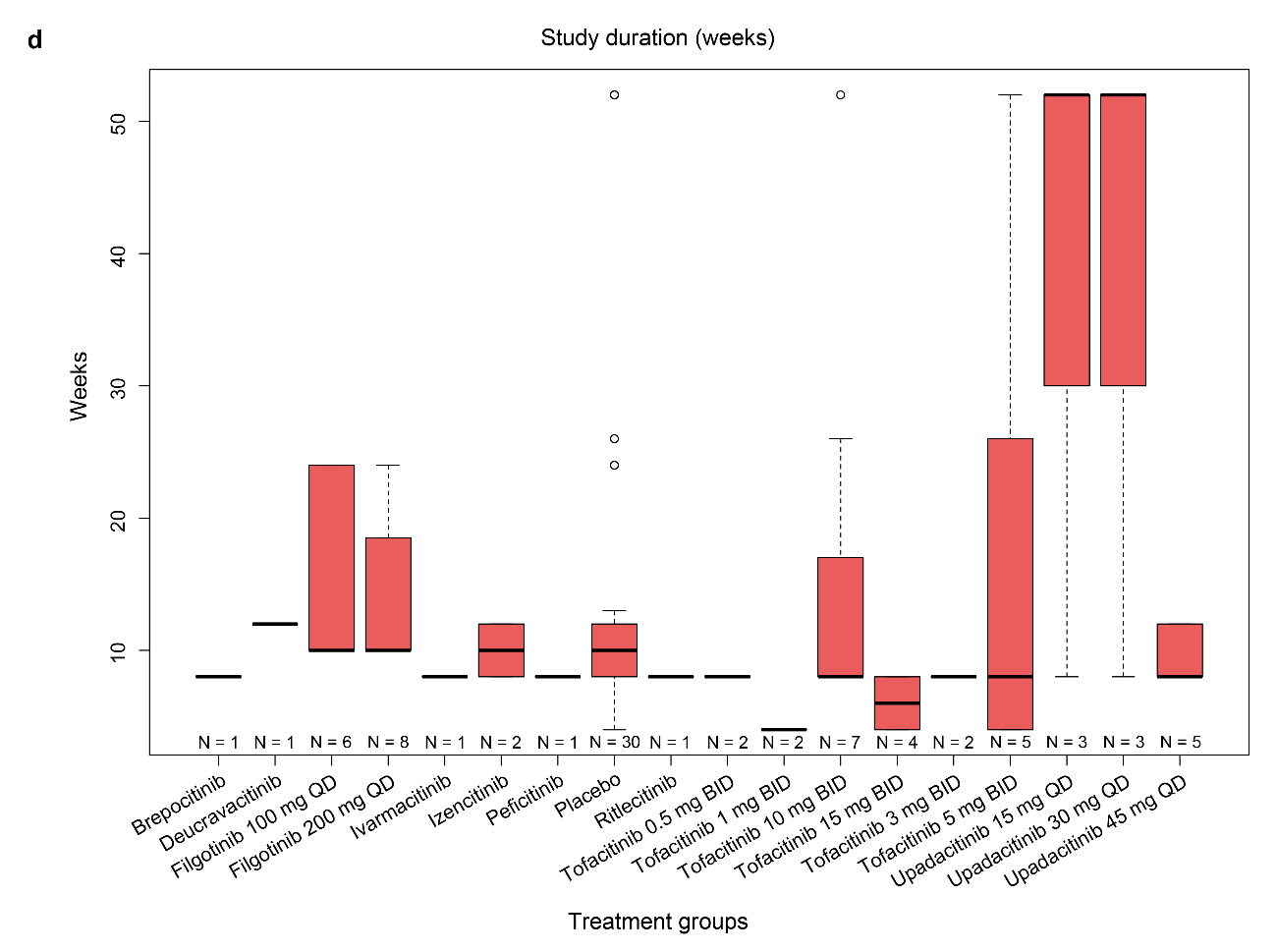
**

**Supplementary figure 3 The summary proportions of the risk of bias**

**
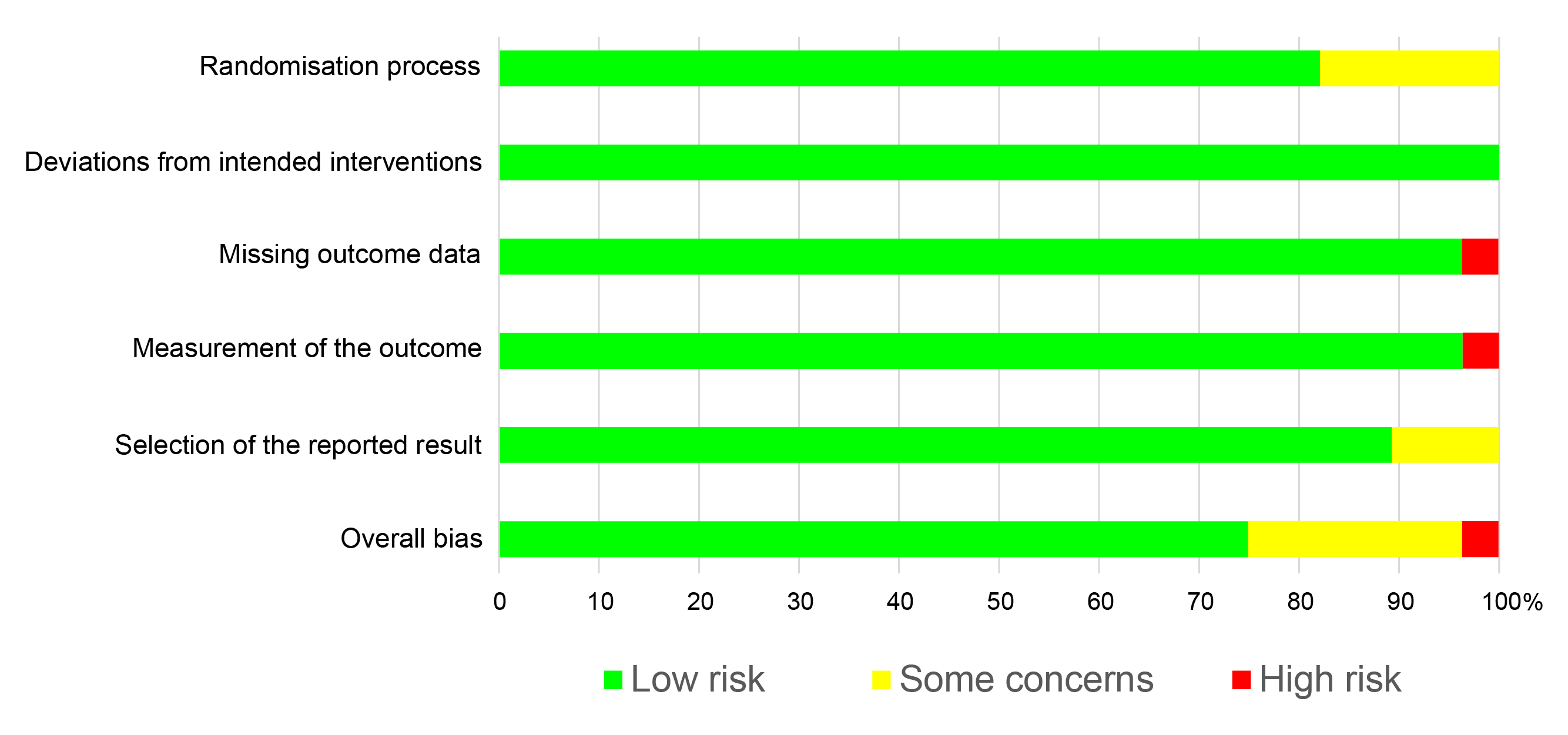
**

**Supplementary figure 4 The details of the risk of bias**


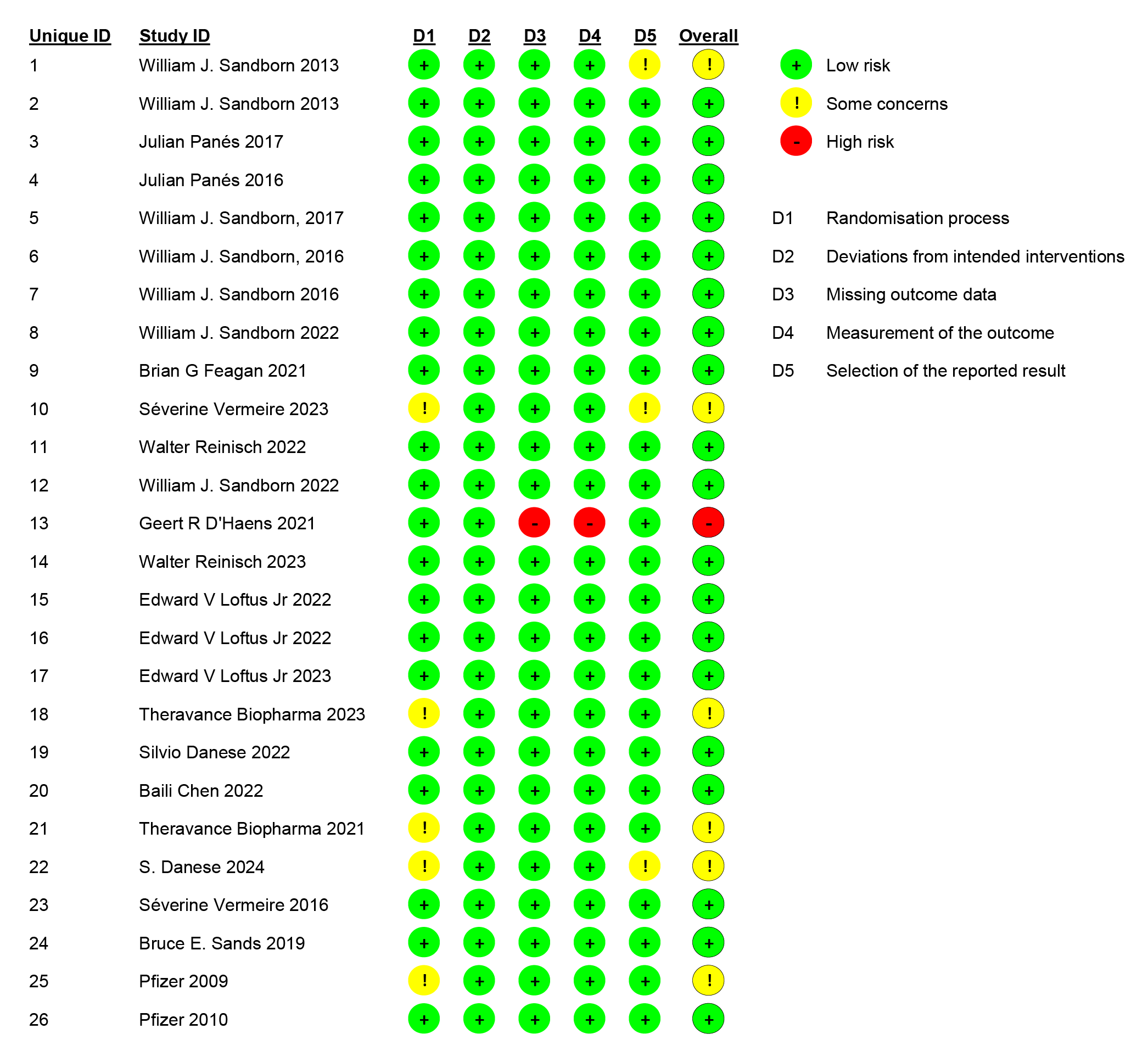


**Supplementary figure 5 Forest plots of comparisons for each included RCT (without dose consideration) for MACE (a), VTE (b) and CVE (c)**

**
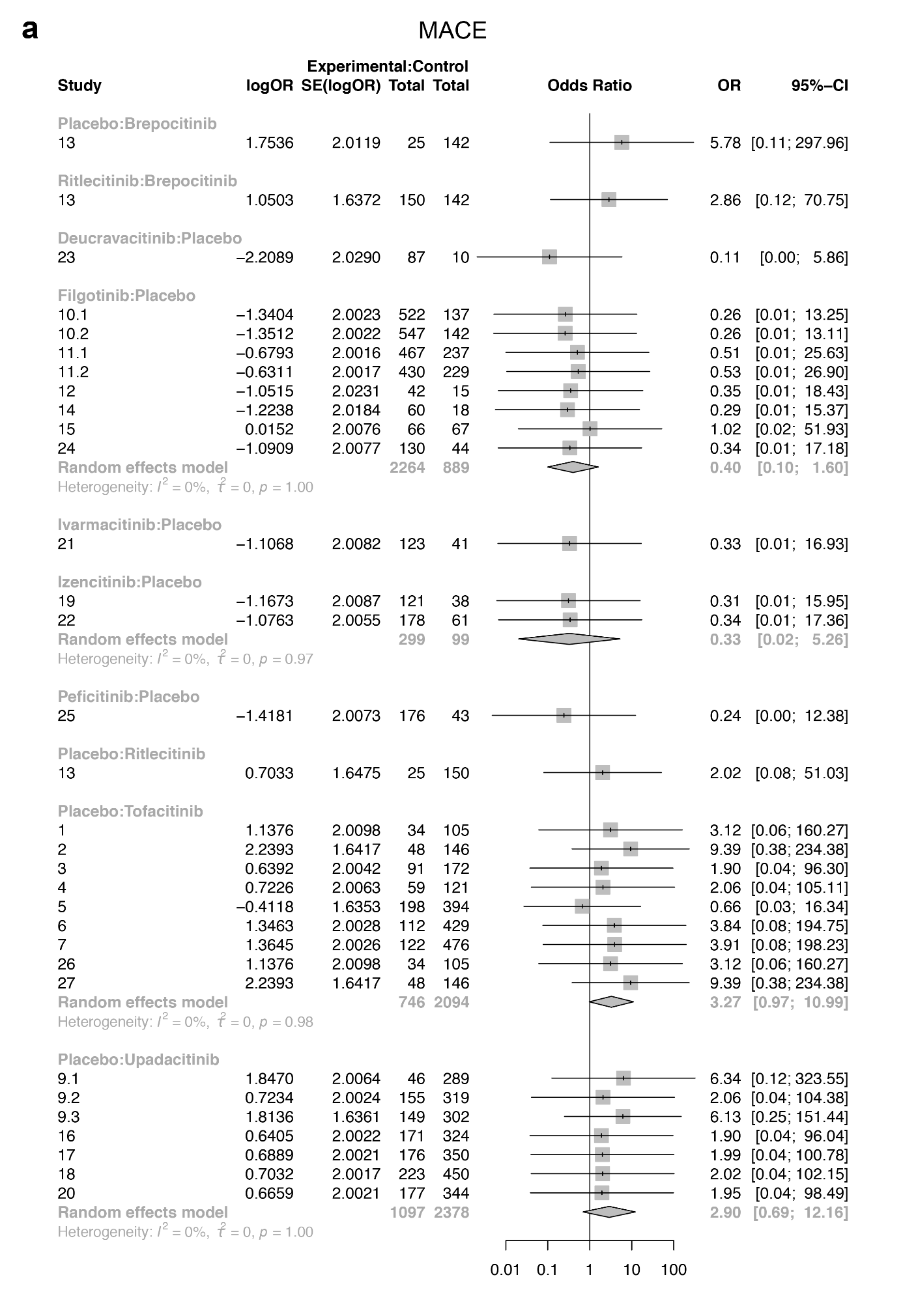
**

**
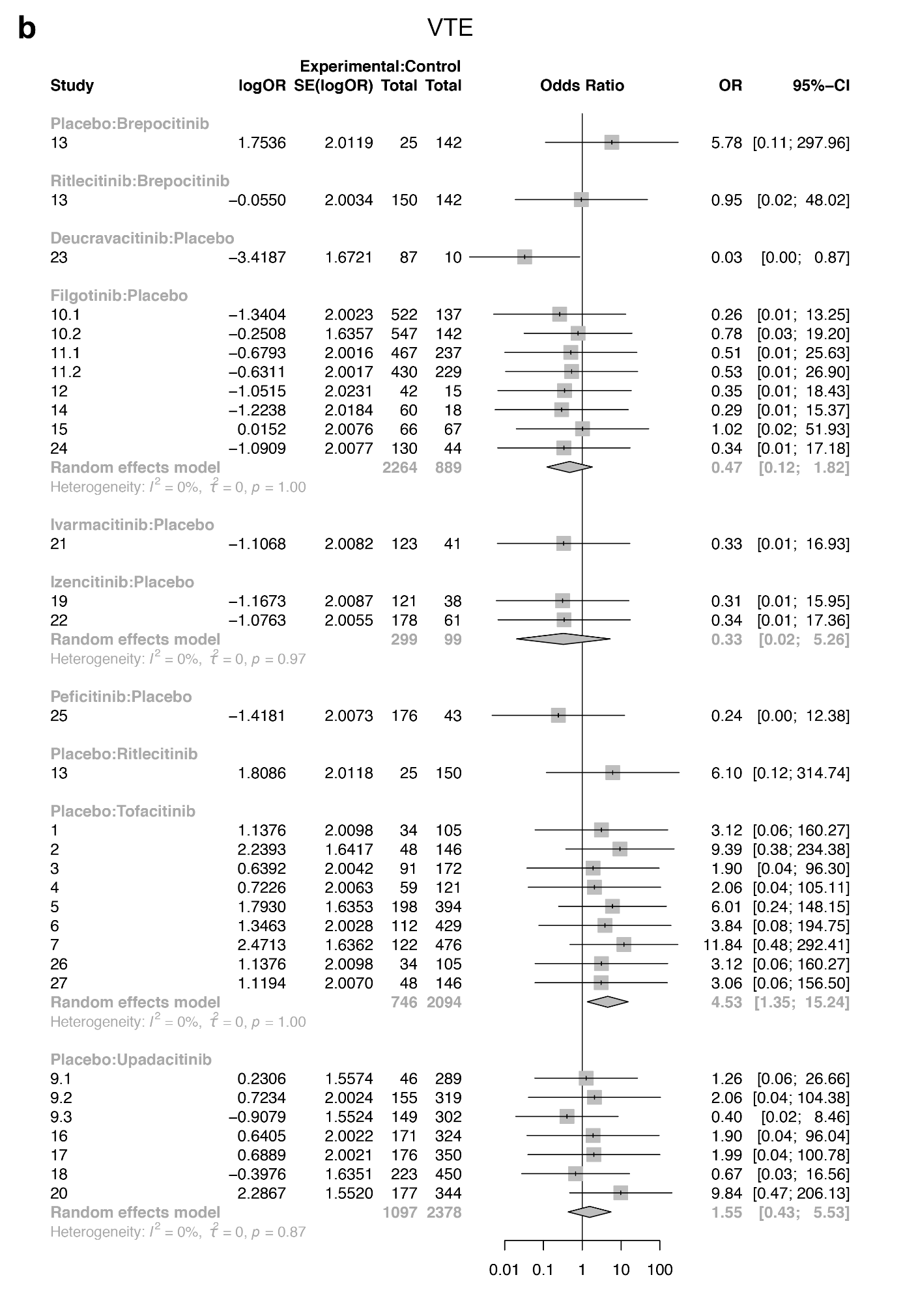
**

**
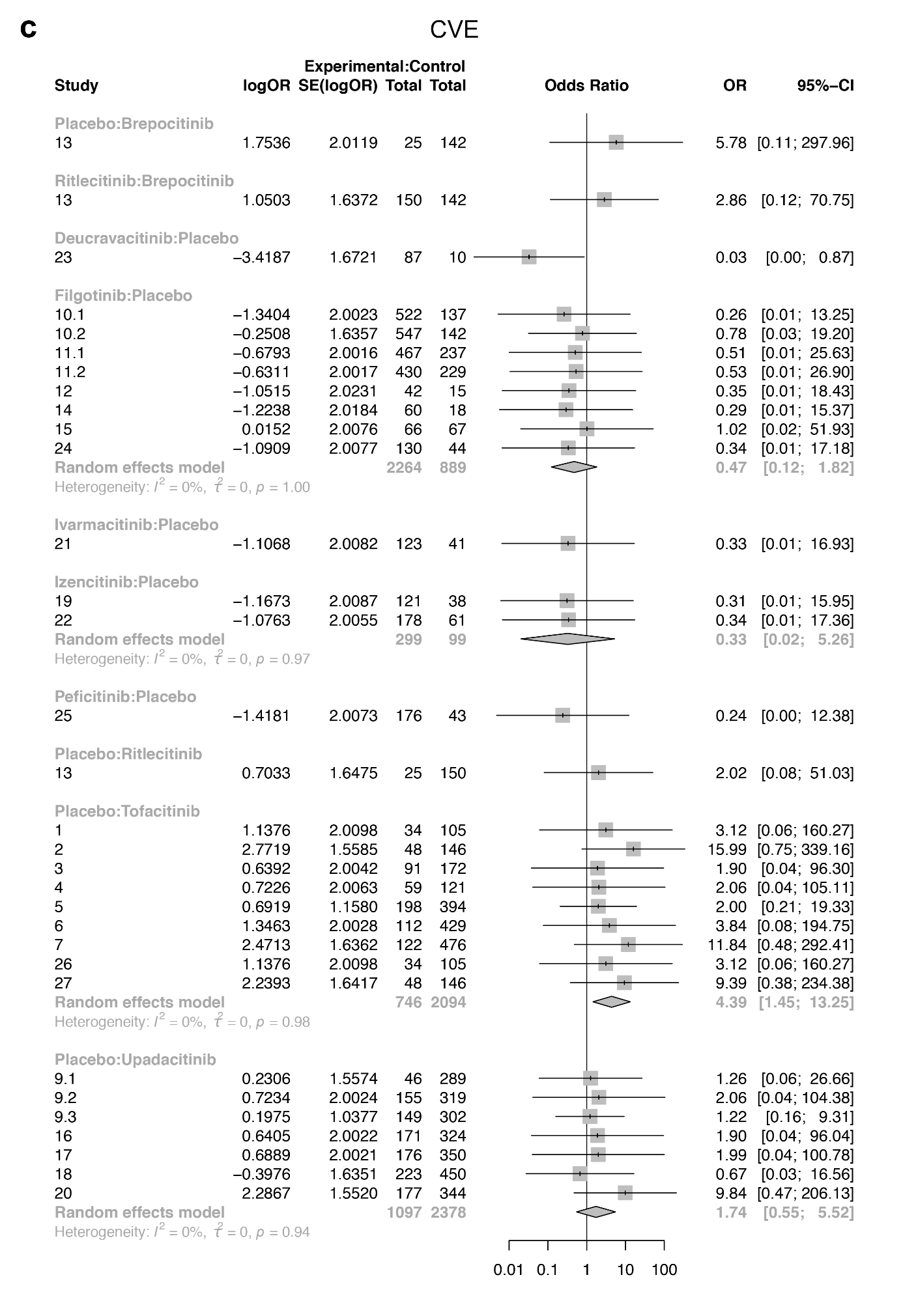
**

**Supplementary figure 6 Forest plots of comparisons for each included RCT (with dose consideration) for MACE (a), VTE (b) and CVE (c)**

**
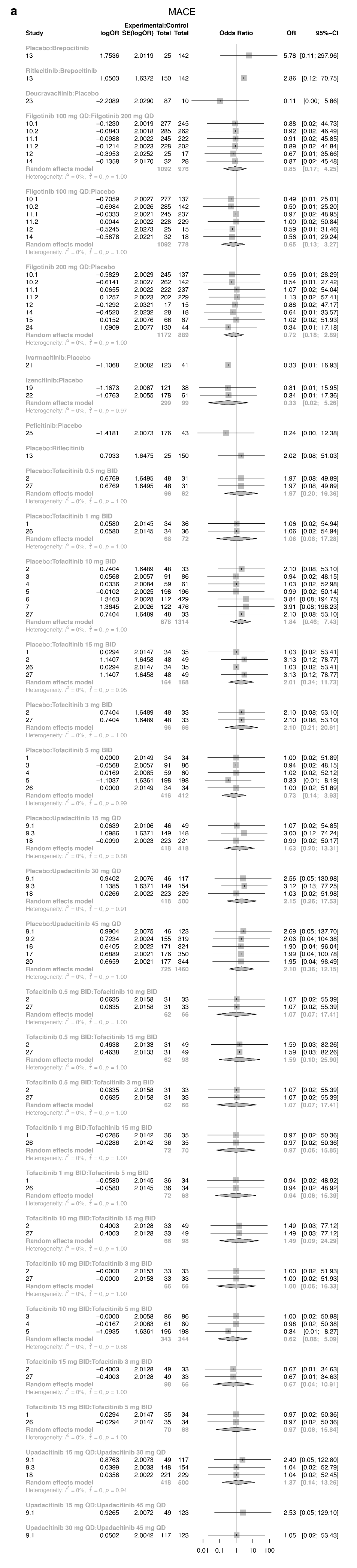
**

**
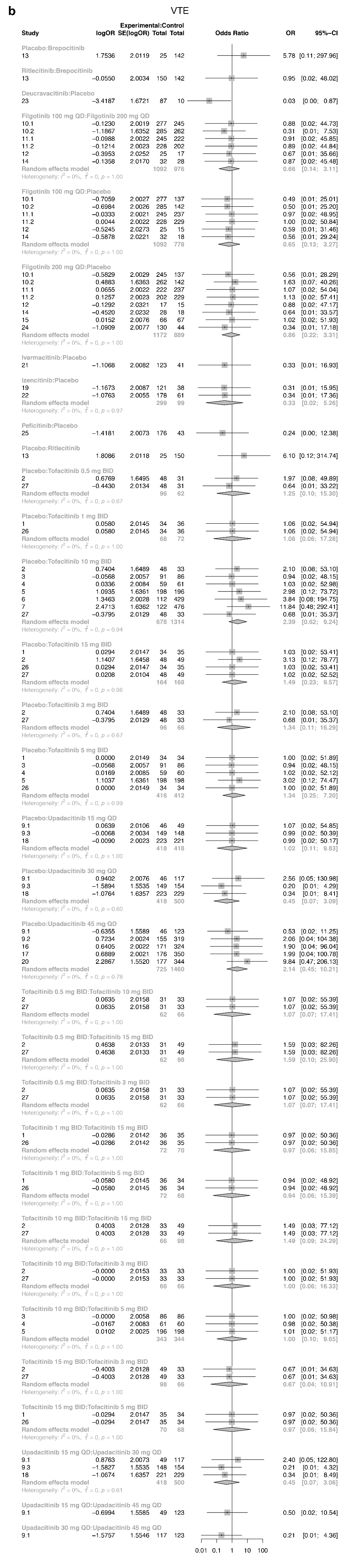
**

**
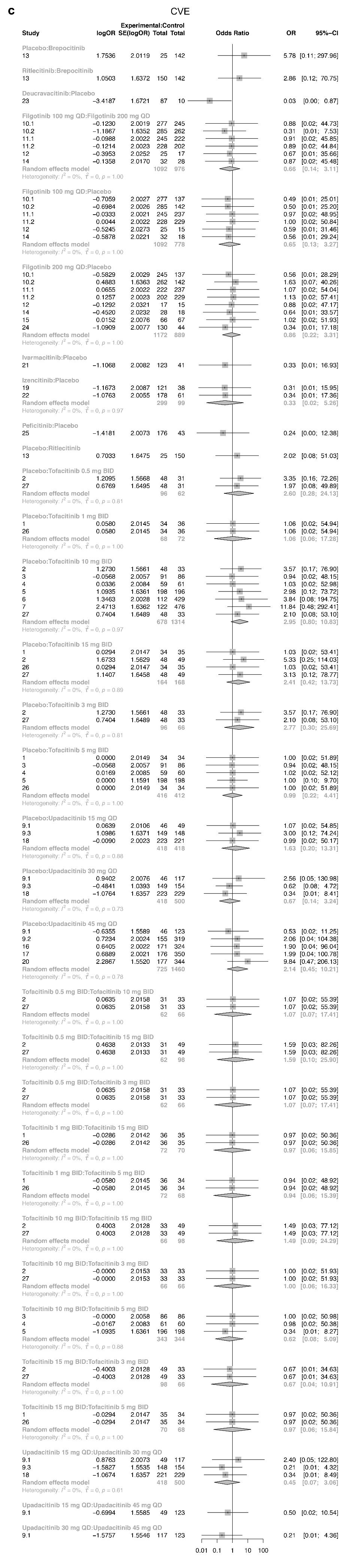
**

**Supplementary figure 7 Evidence proportion plots of network estimates (without dose consideration)**


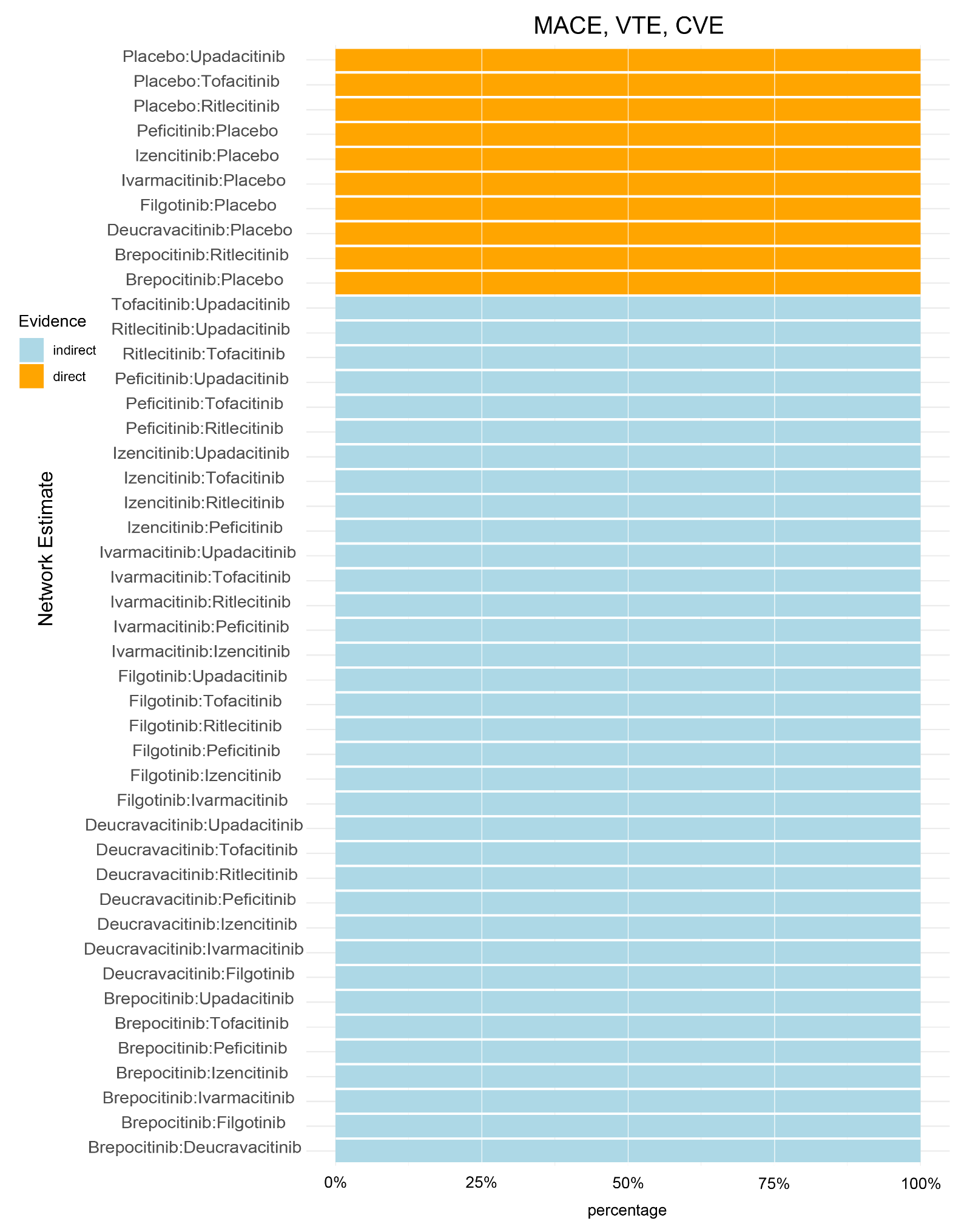


**Supplementary figure 8 Evidence proportion plots of network estimates (with dose consideration) for comparisons with direct comparisons for MACE (a), VTE (b) and CVE (c), as well as for comparisons without direct comparisons (d) for MACE, VTE and CVE**


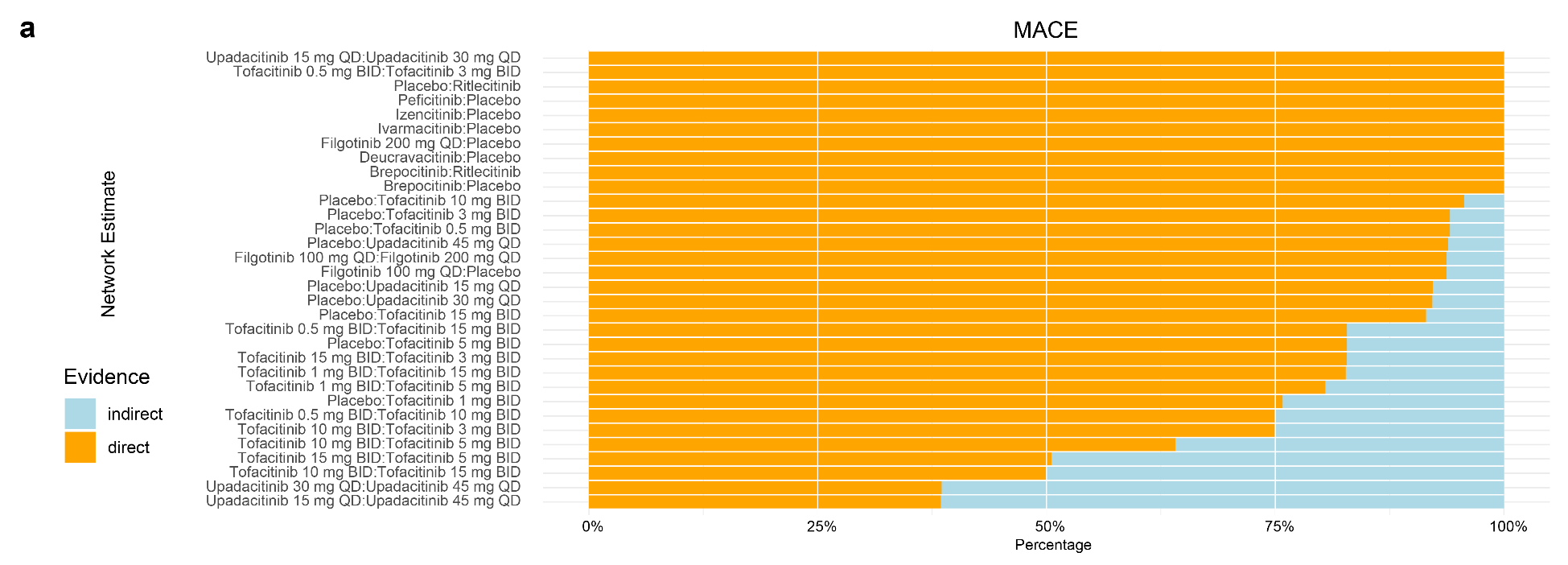


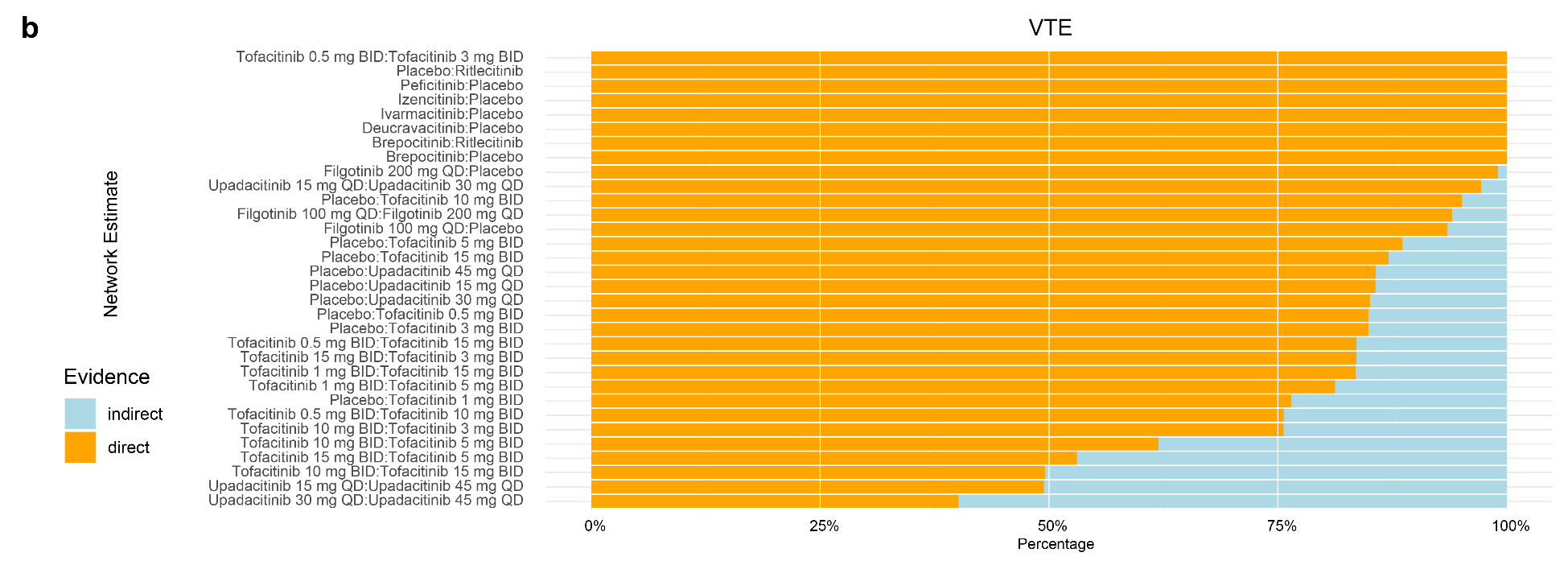


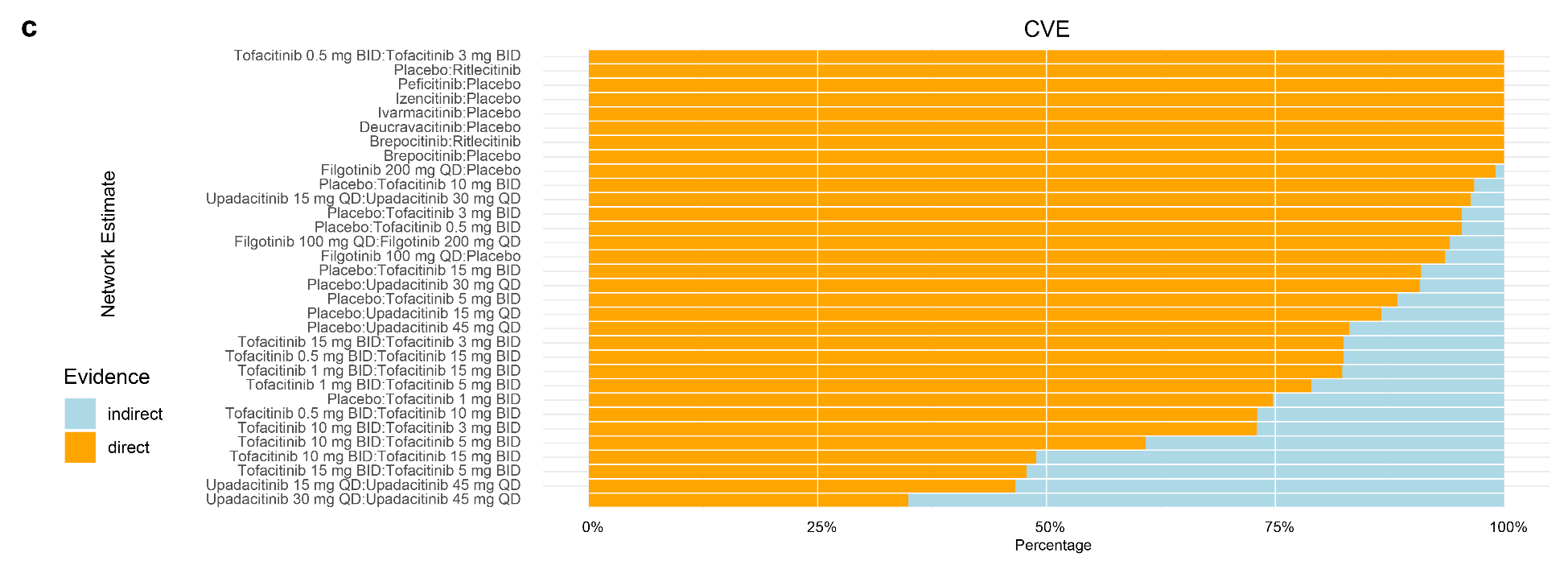


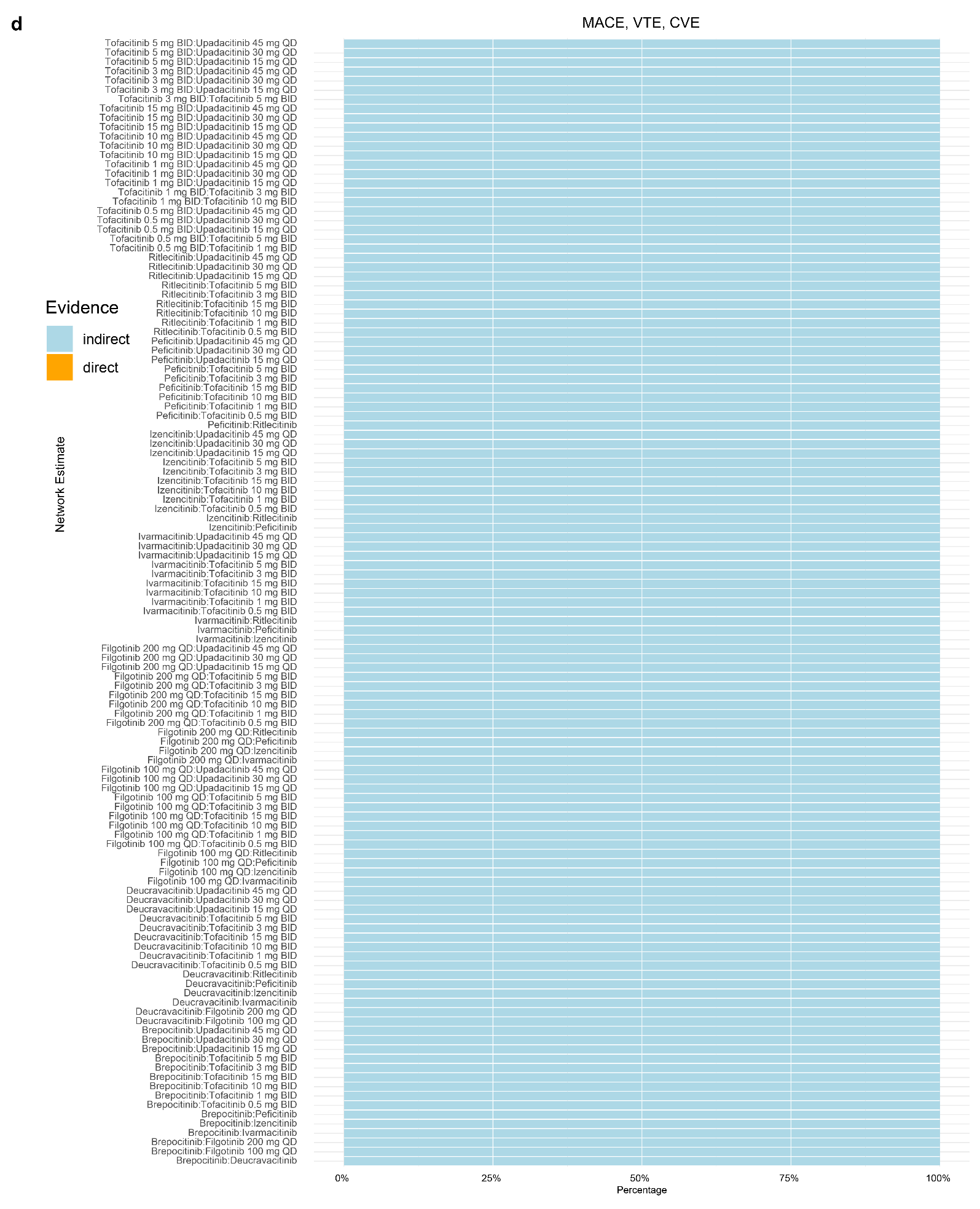


**Supplementary figure 9 Rankogram plots for all interventions (without dose consideration) for MACE (a), VTE (b) and CVE (c)**


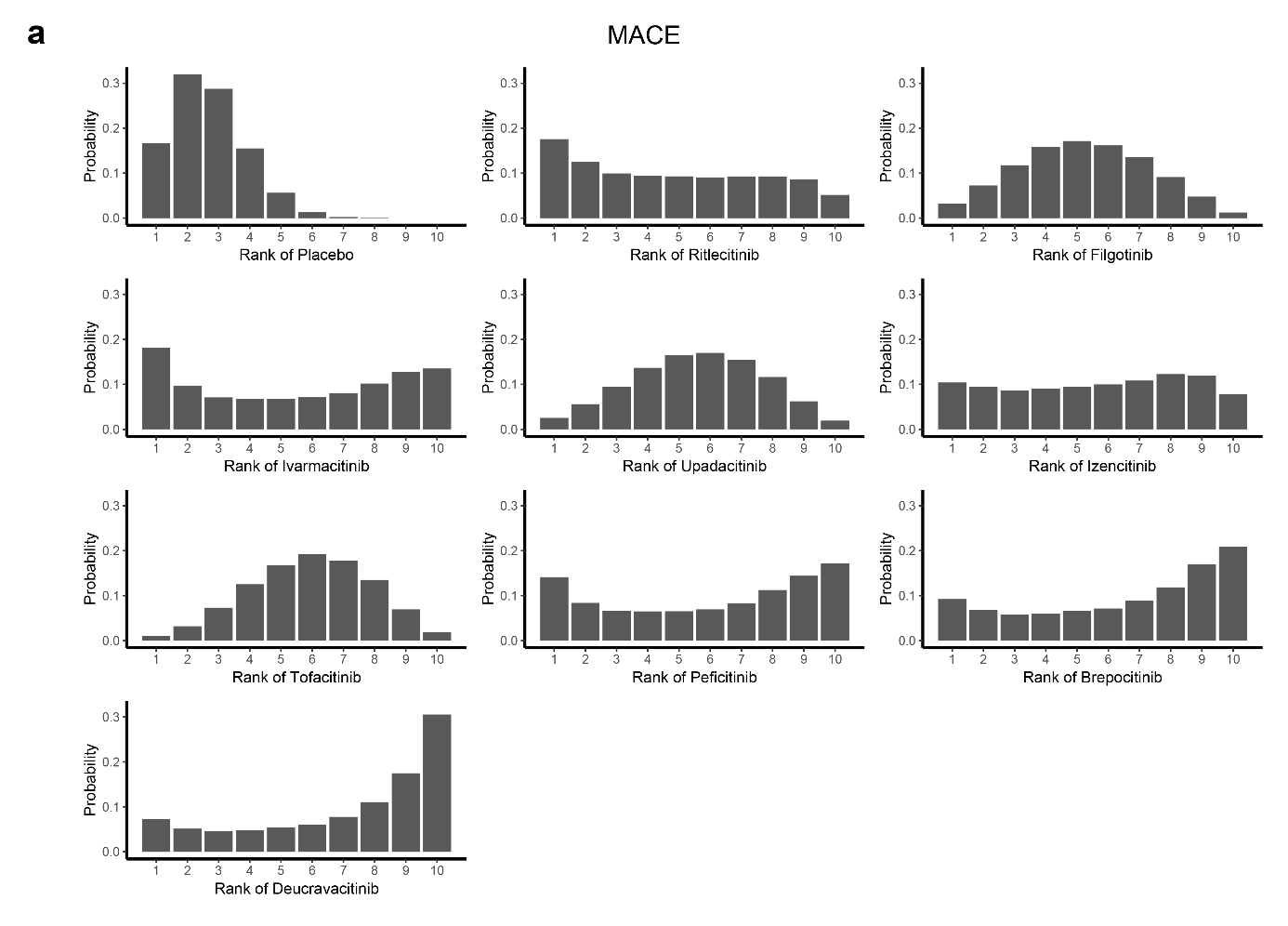


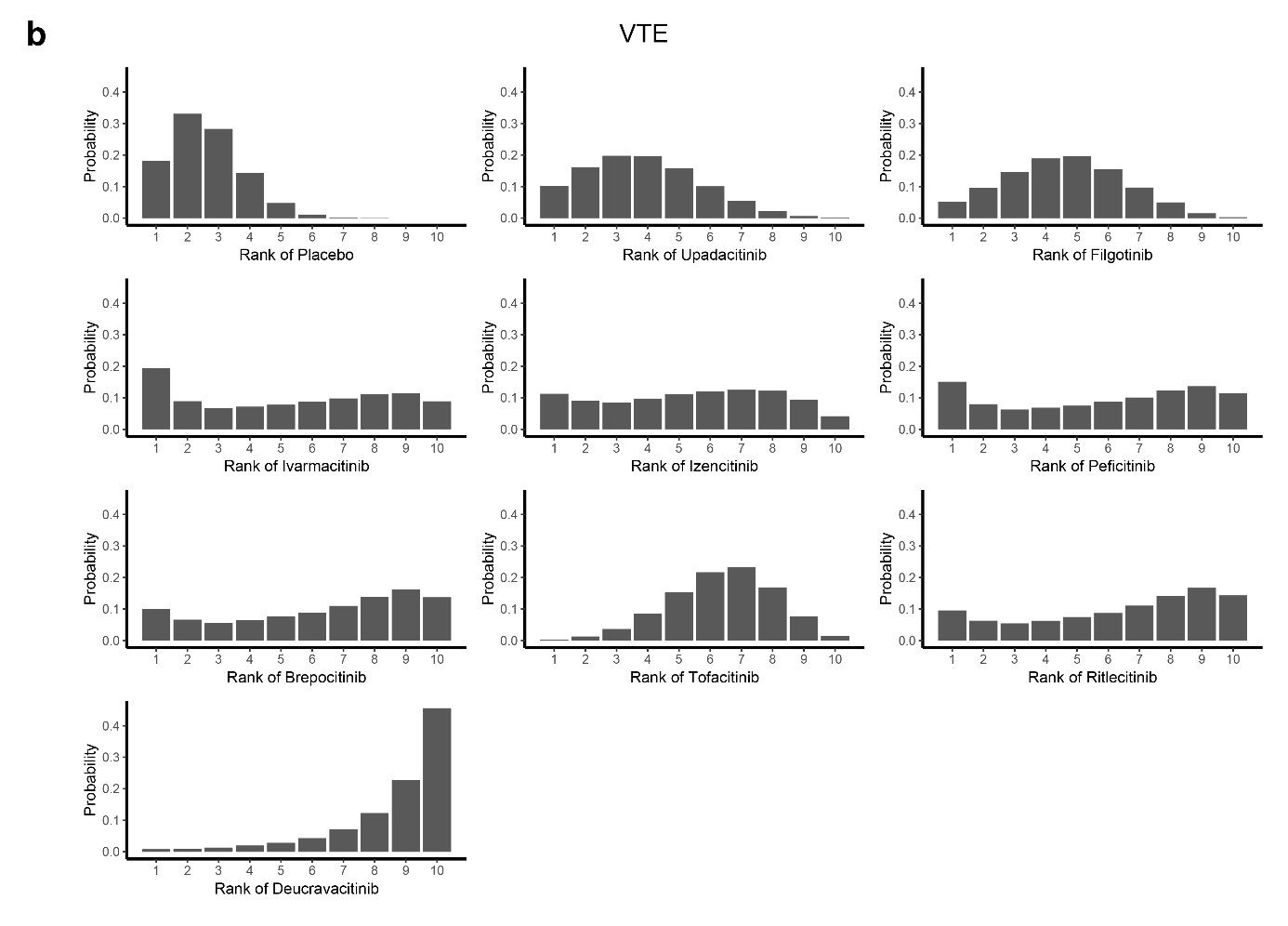


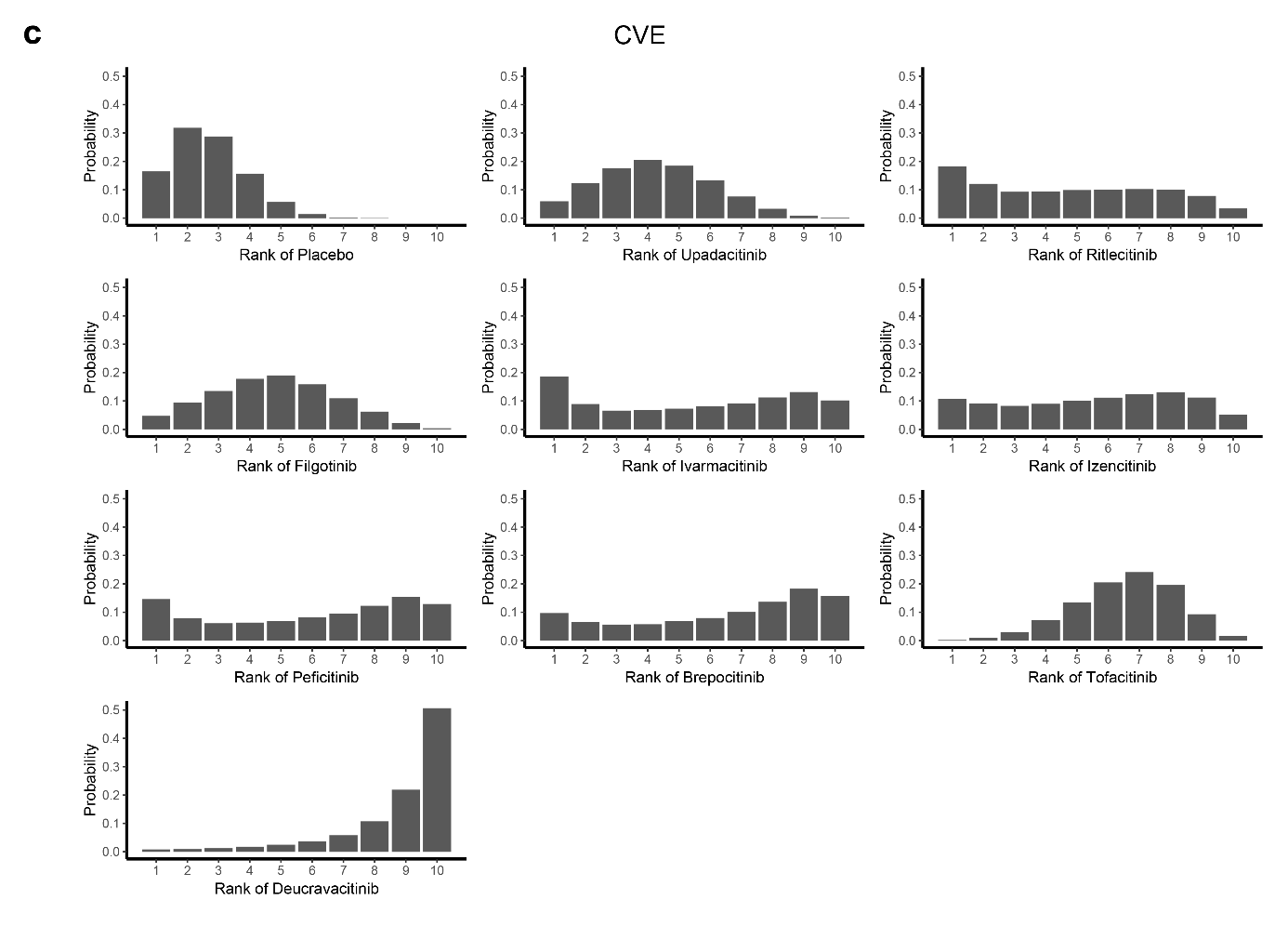


**Supplementary figure 10 Rankogram plots for all interventions (with dose consideration) for MACE (a), VTE (b) and CVE (c)**

**
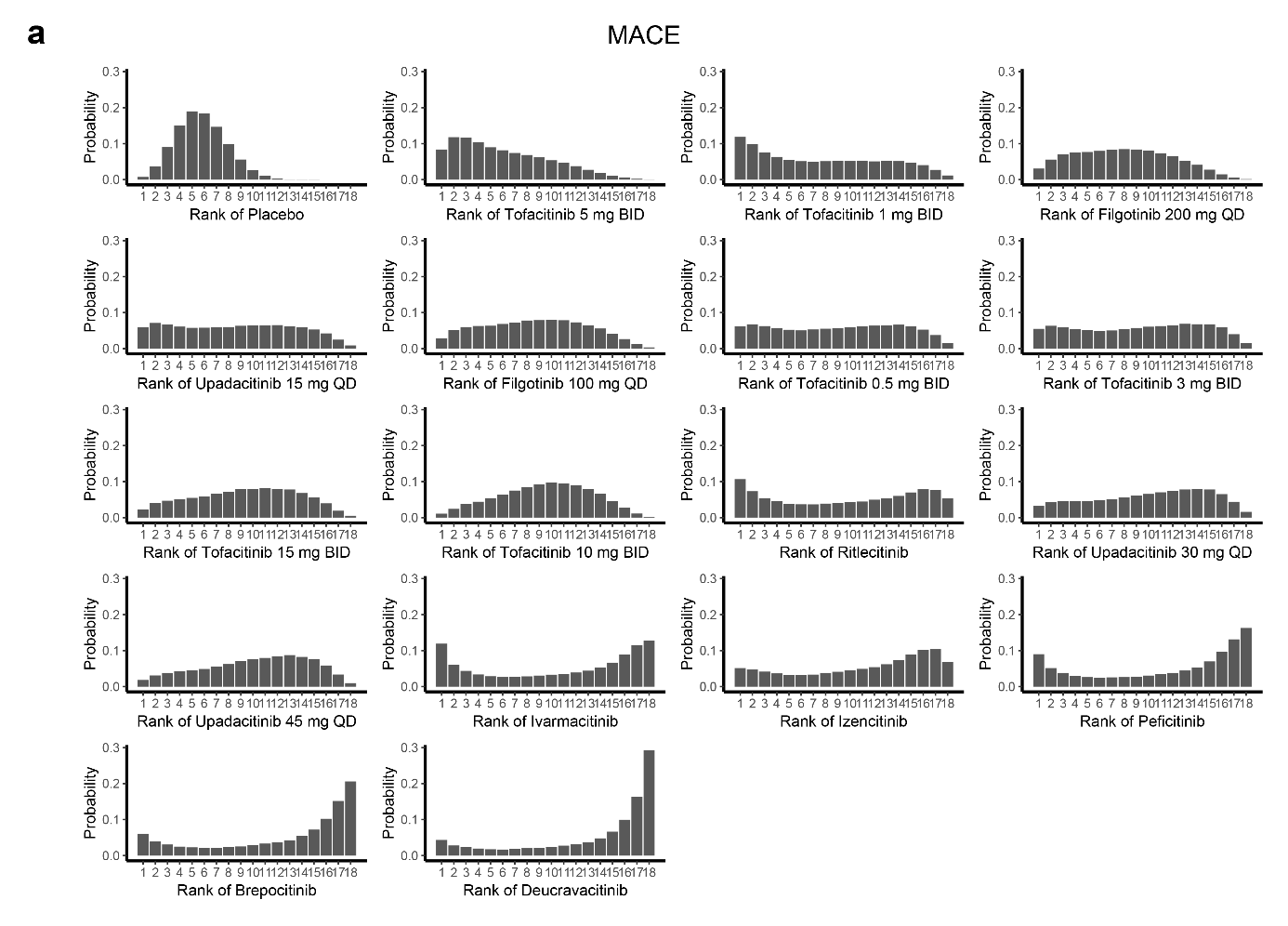
**

**
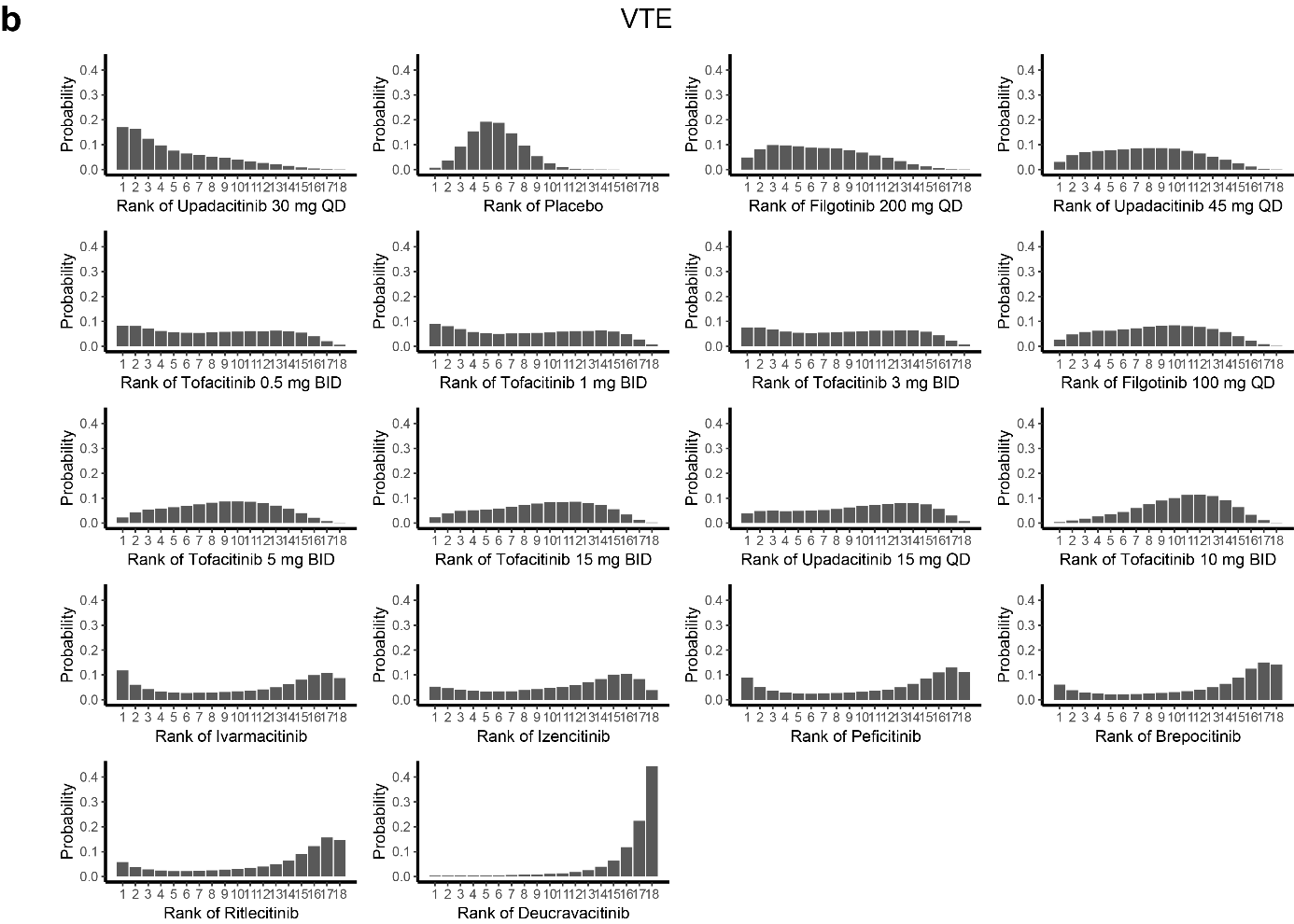
**

**
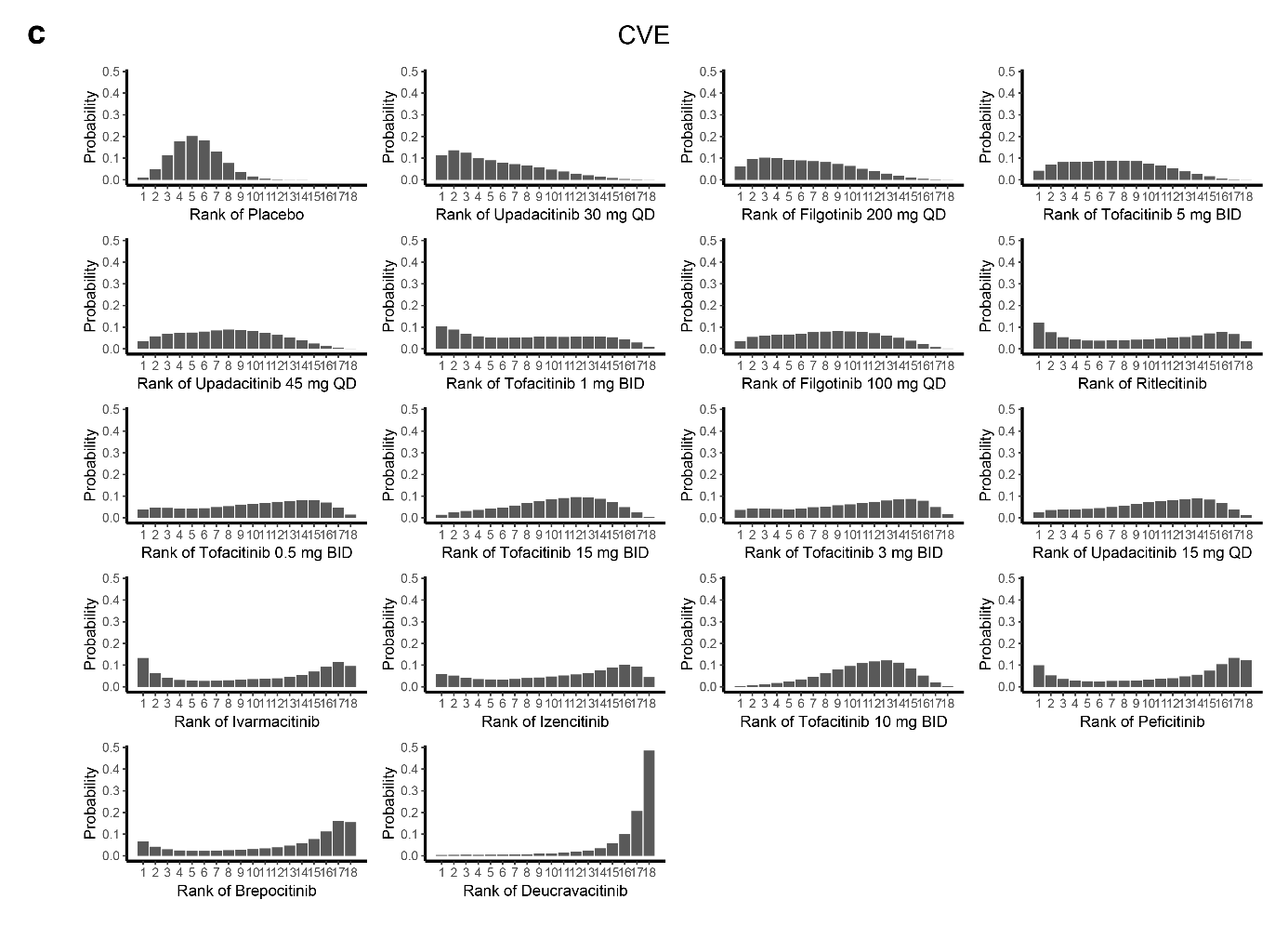
**

**Supplementary figure 11 Cumulative ranking plots for all interventions (without dose consideration) for MACE (a), VTE (b) and CVE (c)**

**
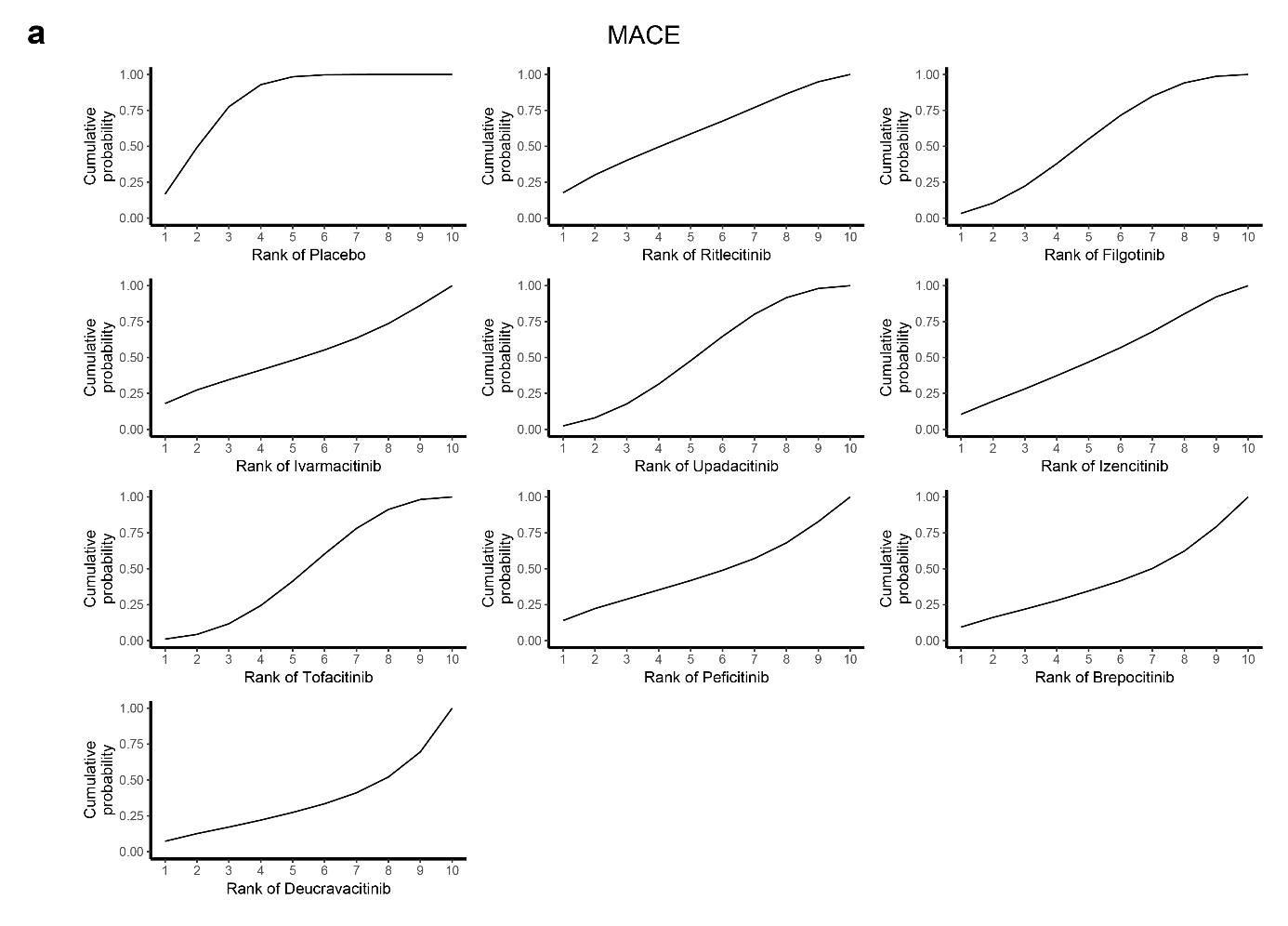
**

**
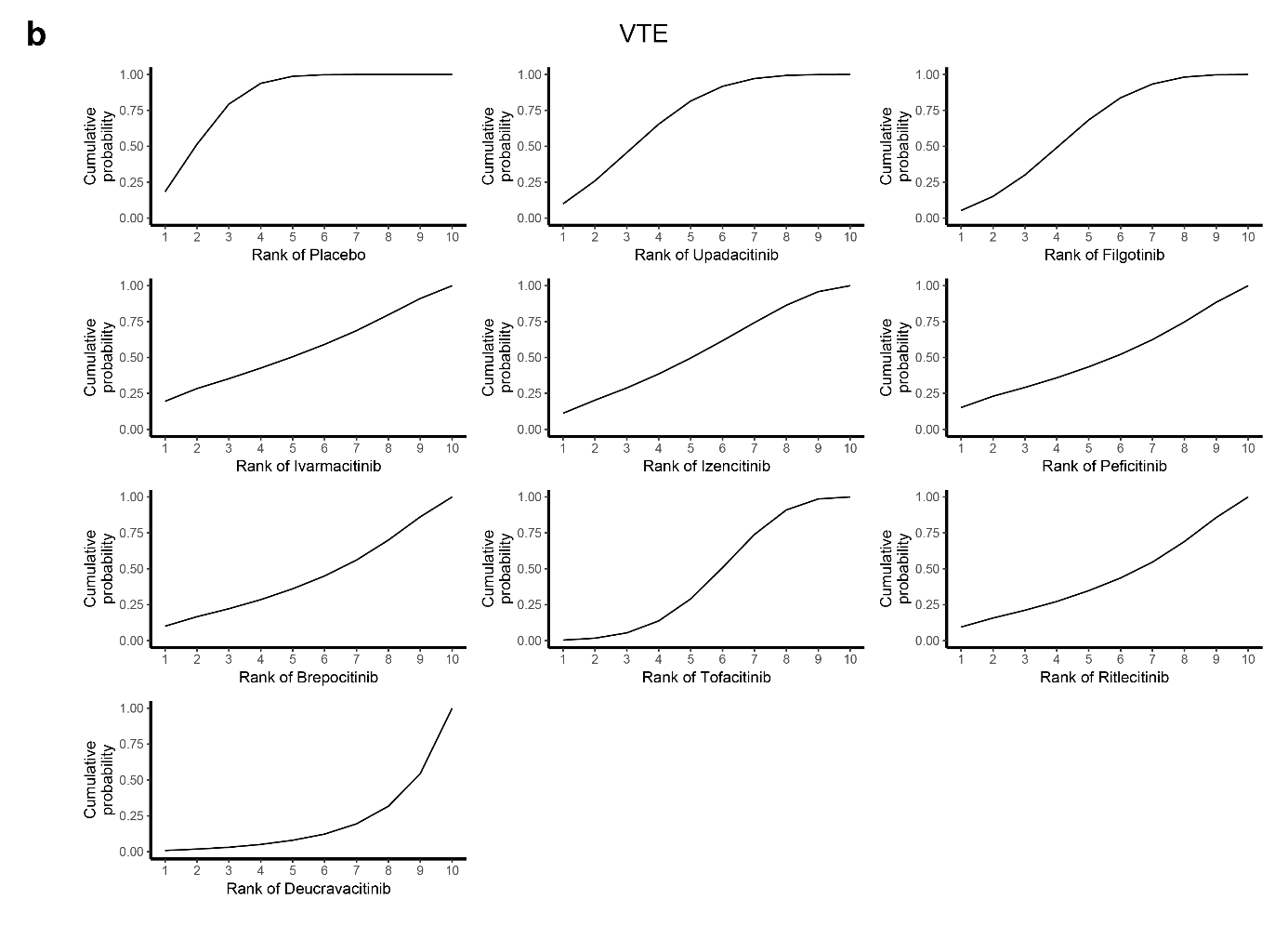
**

**
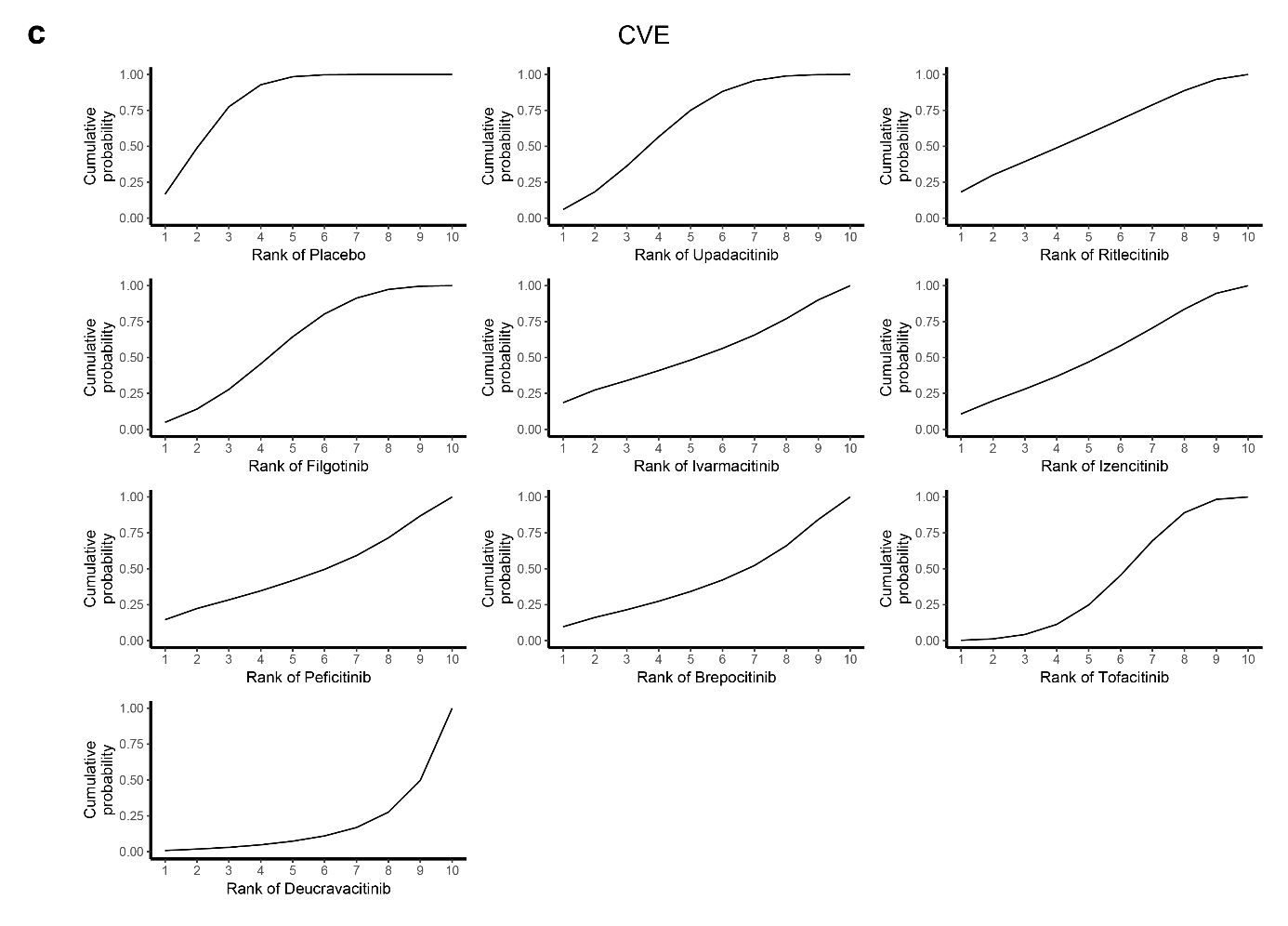
**

**Supplementary figure 12 Cumulative ranking plots for all interventions (with dose consideration) for MACE (a), VTE (b) and CVE (c)**

**
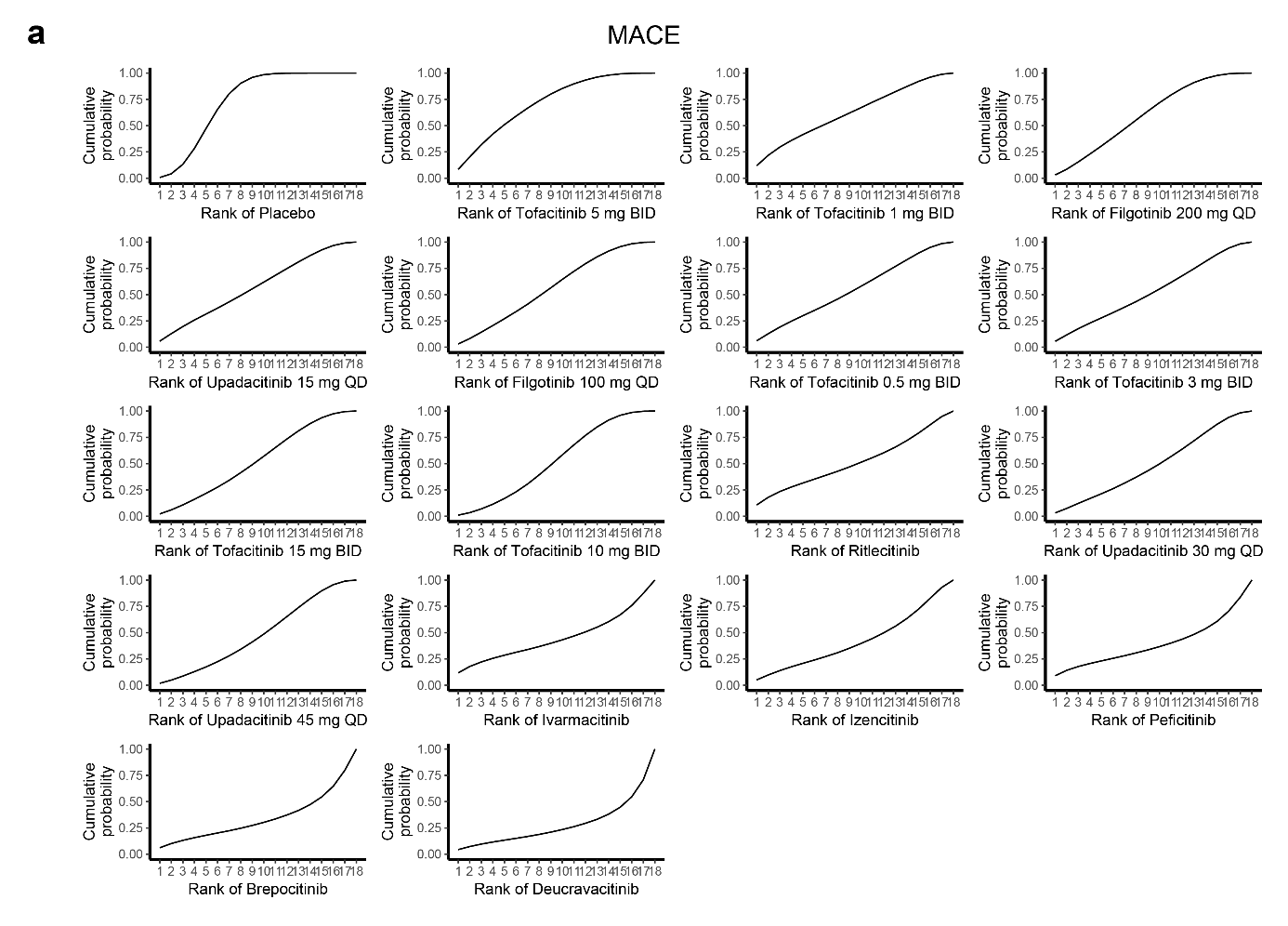
**

**
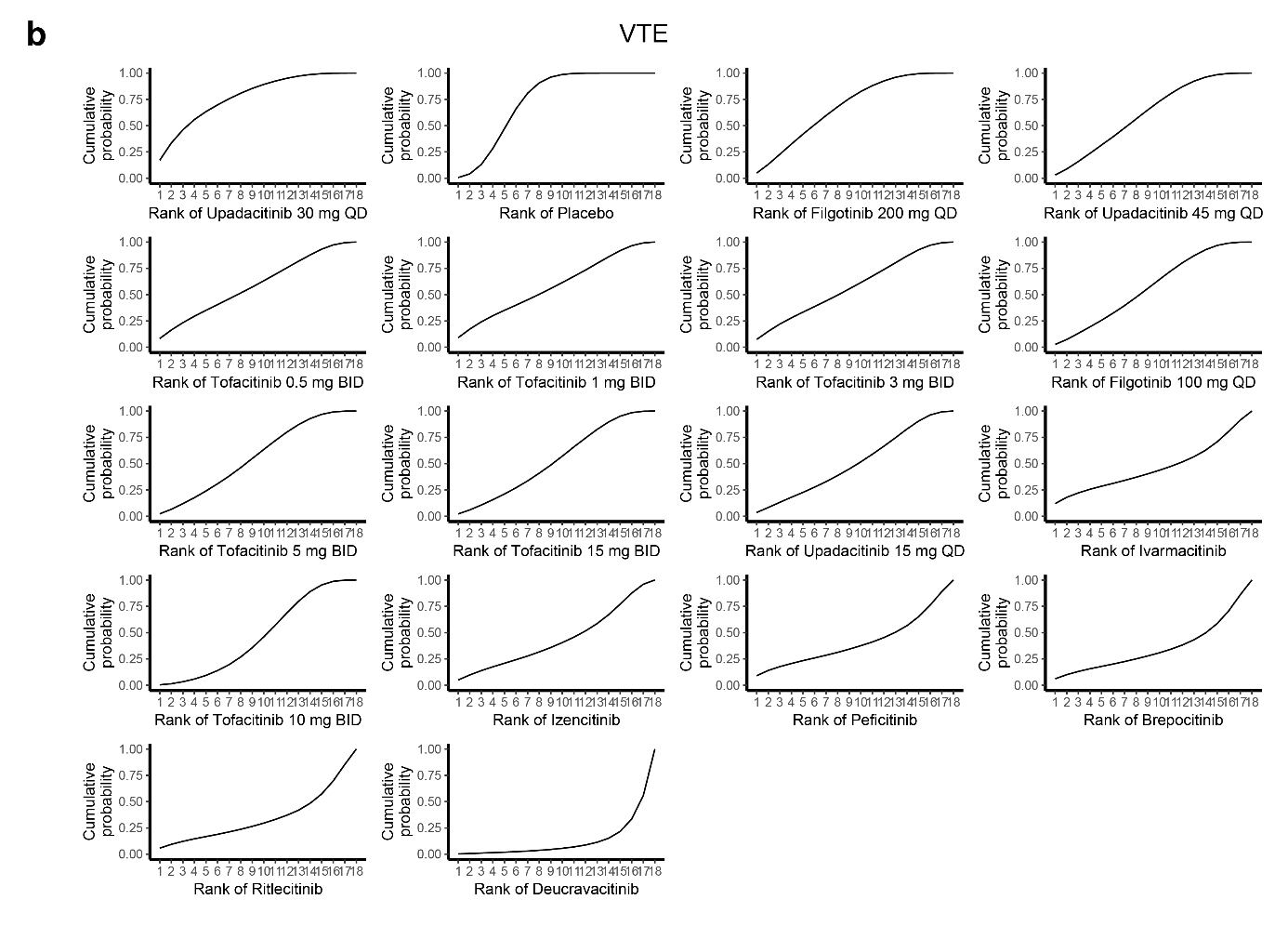
**

**
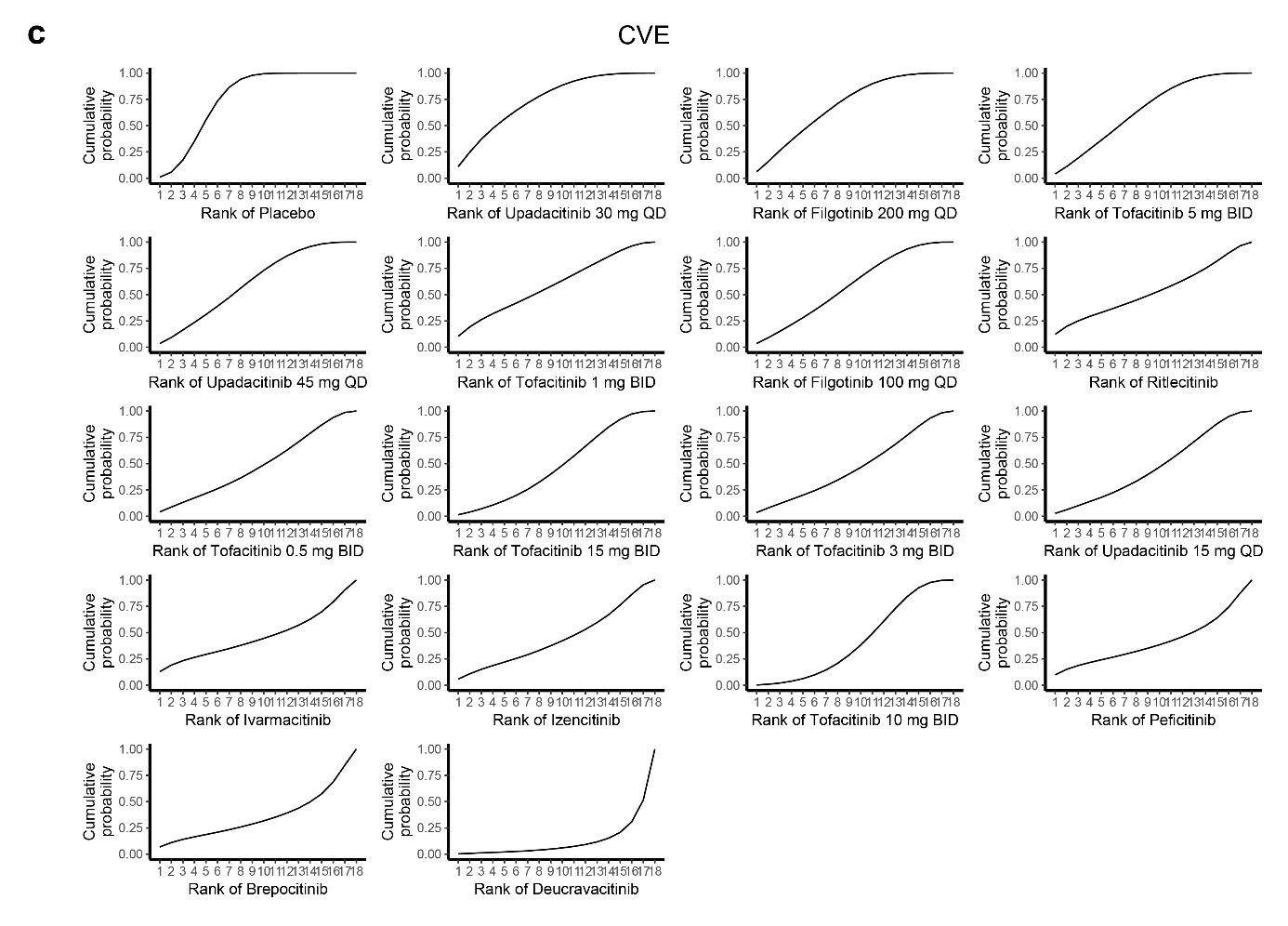
**

**Supplementary figure 13 Funnel plots and Egger’s tests of the included studies (without dose consideration) for MACE (a), VTE (b) and CVE (c)**

**
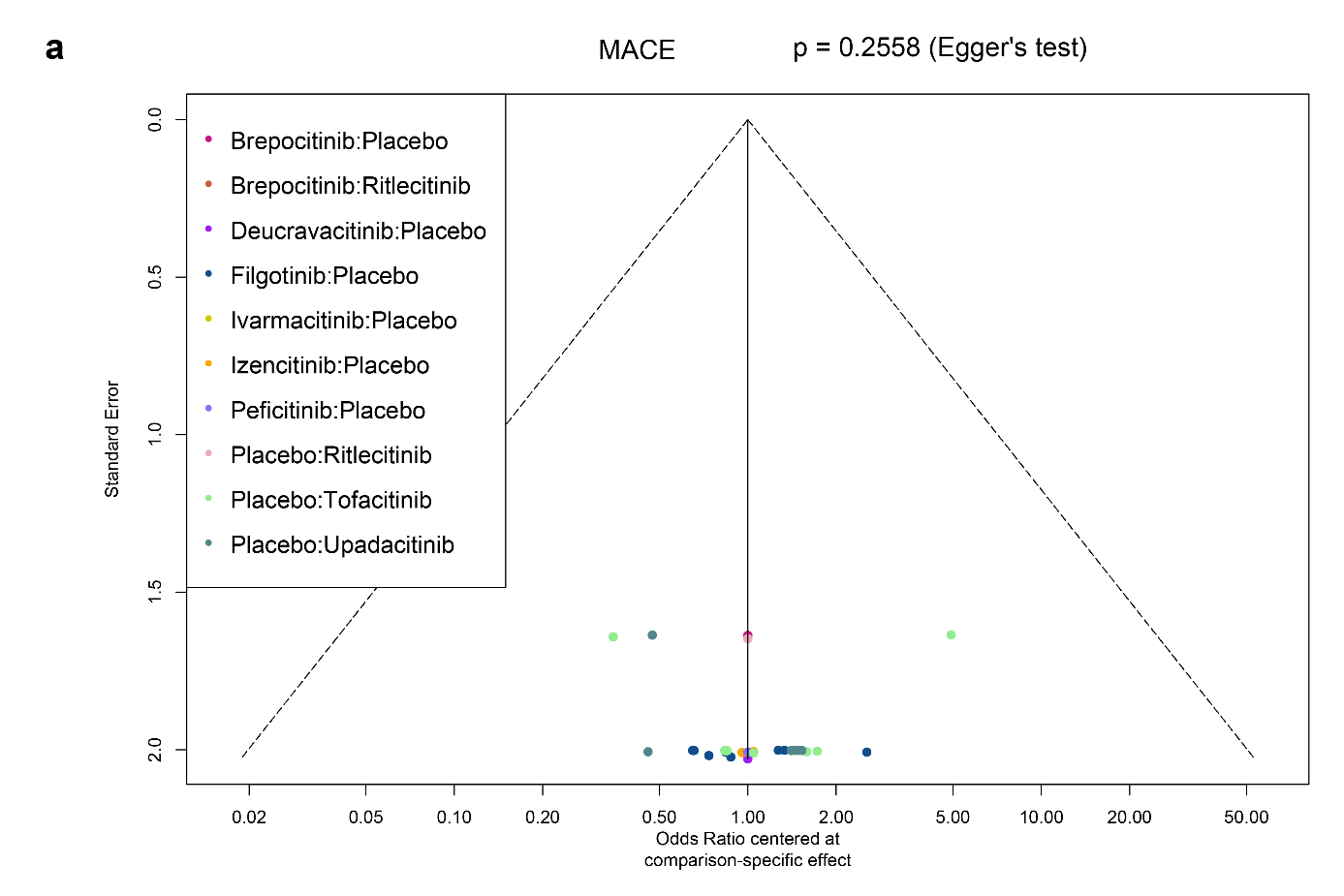
**

**
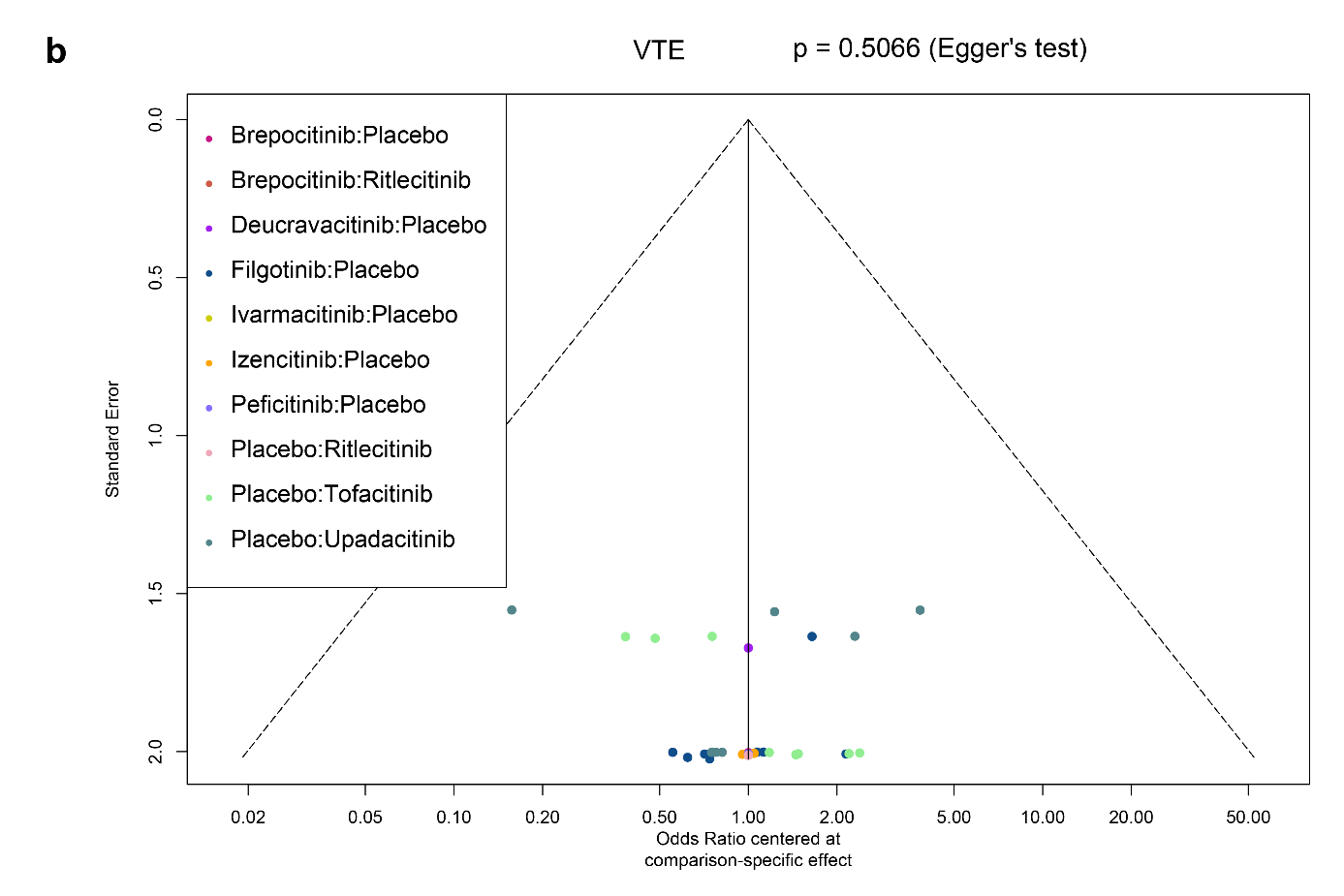
**

**
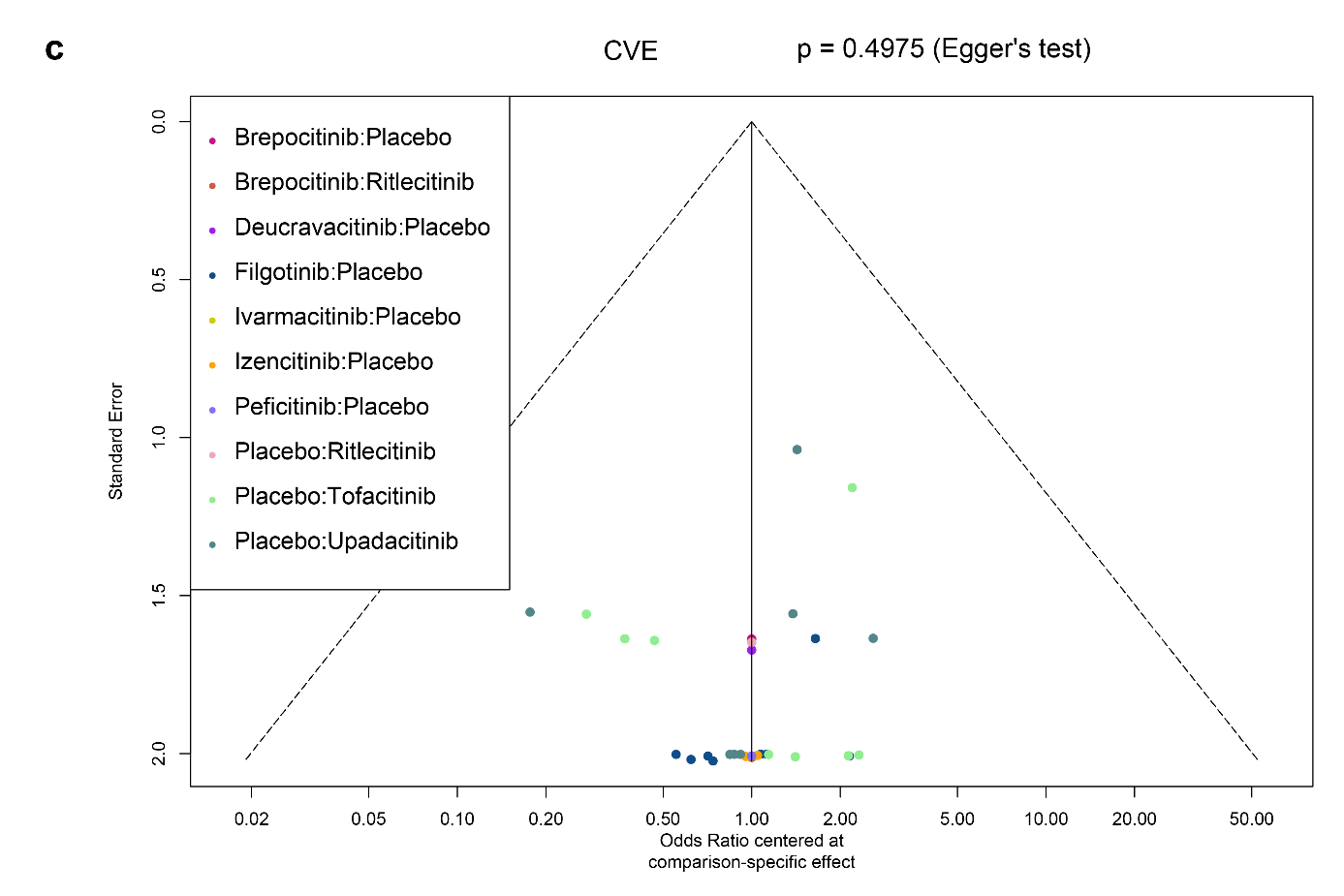
**

**Supplementary figure 14 Funnel plots and Egger’s tests of the included studies (with dose consideration) for MACE (a), VTE (b) and CVE (c)**

**
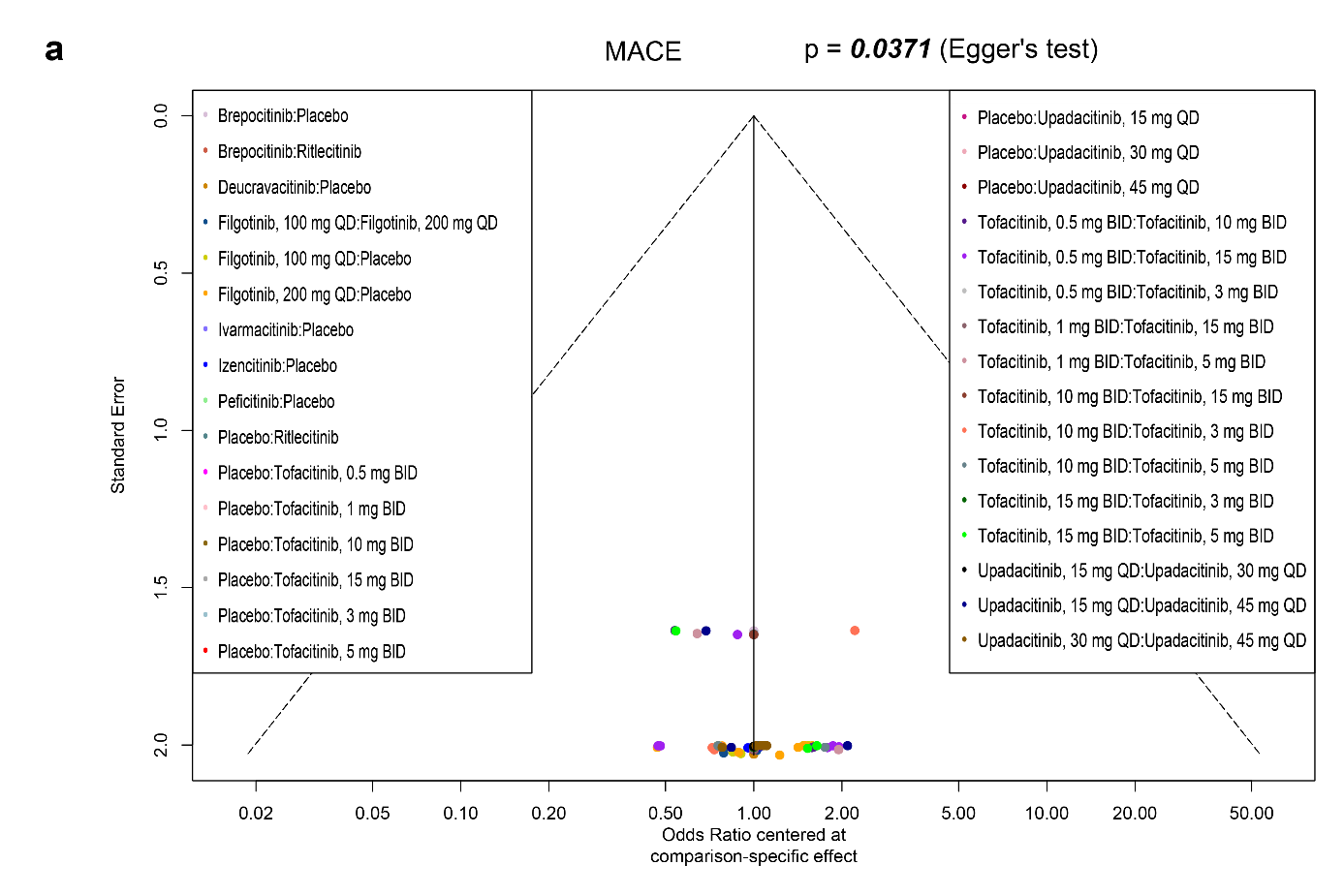
**


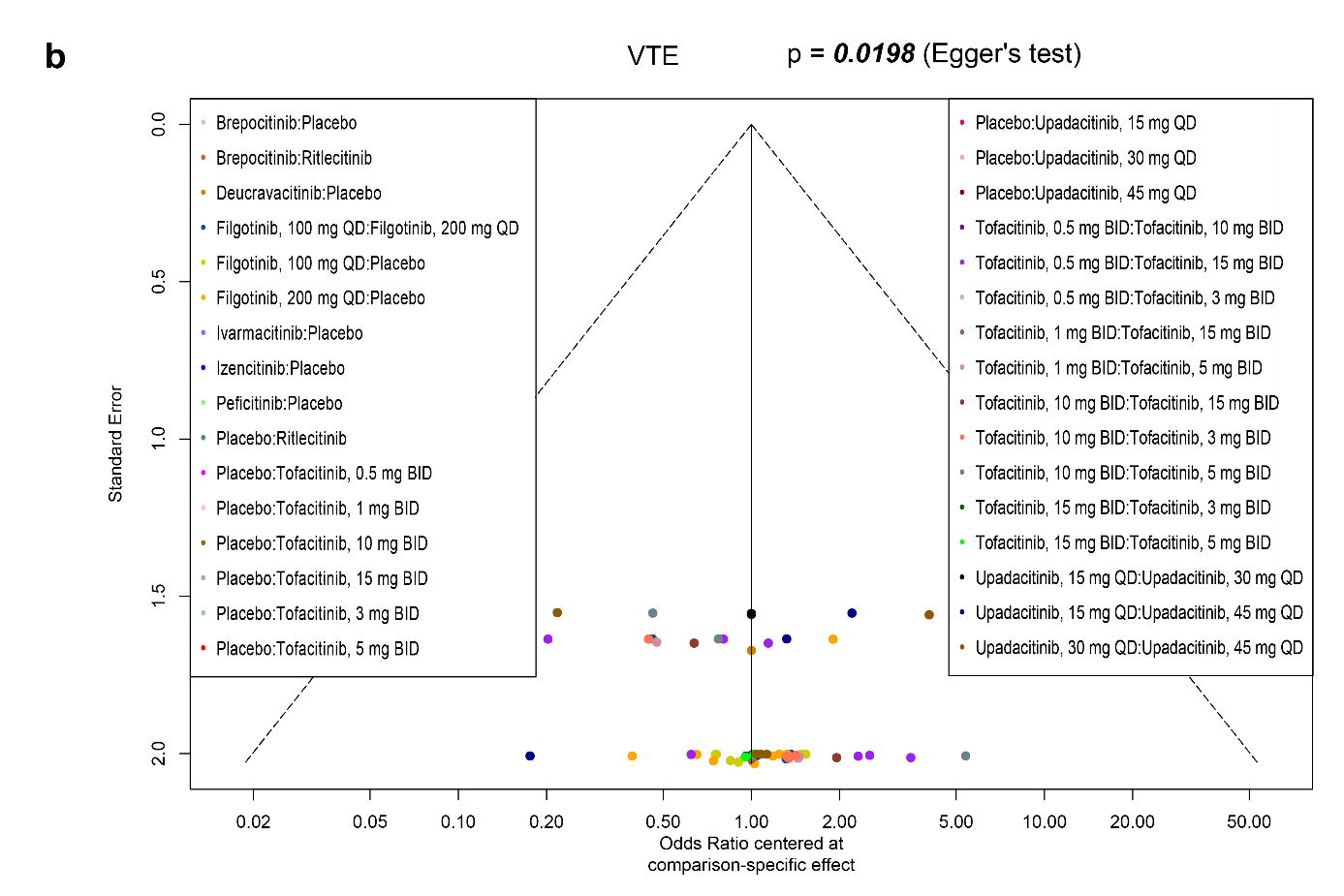


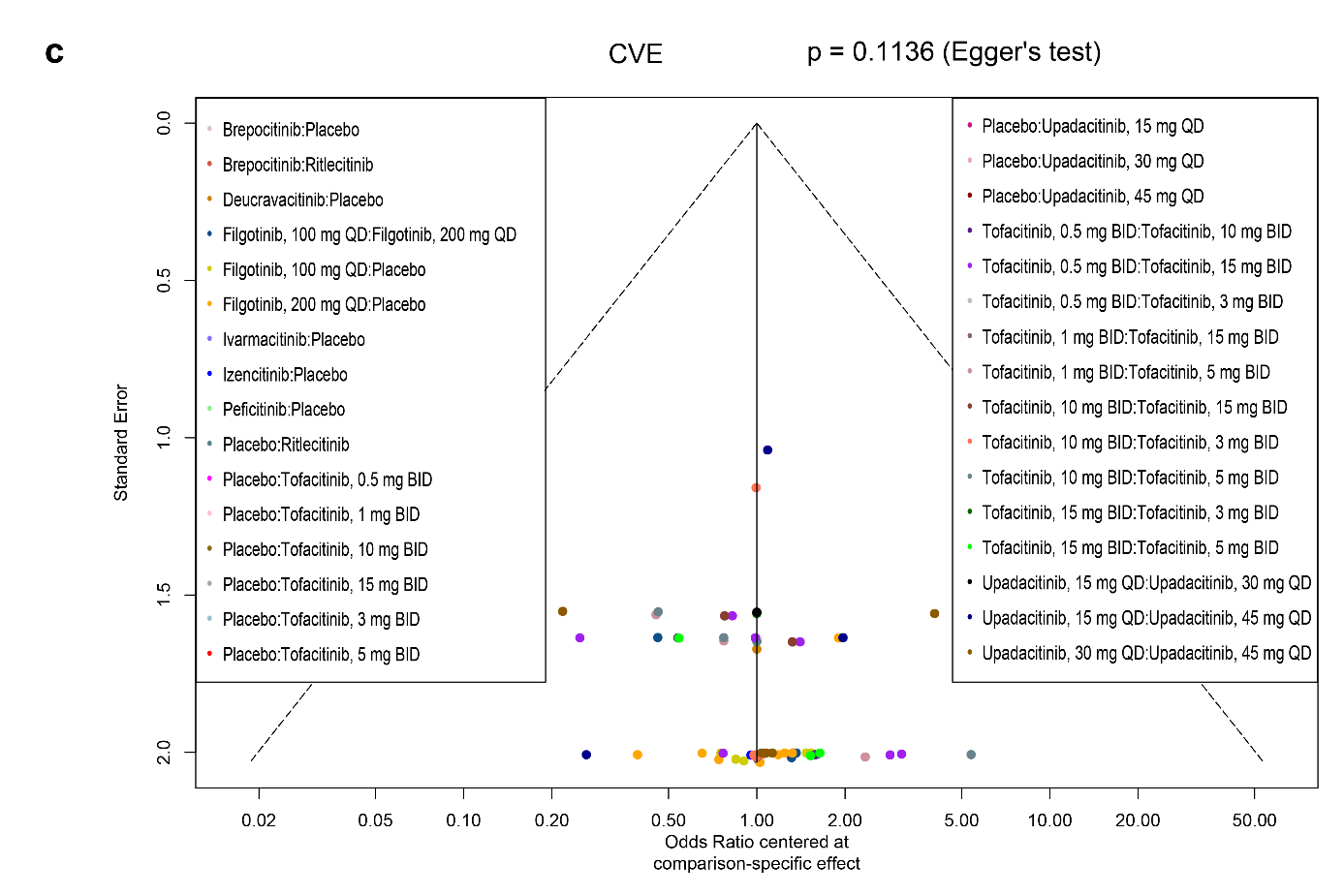


**Supplementary figure 15 Local inconsistency analyses of comparisons (without dose consideration) for MACE (a), VTE (b) and CVE (c)**


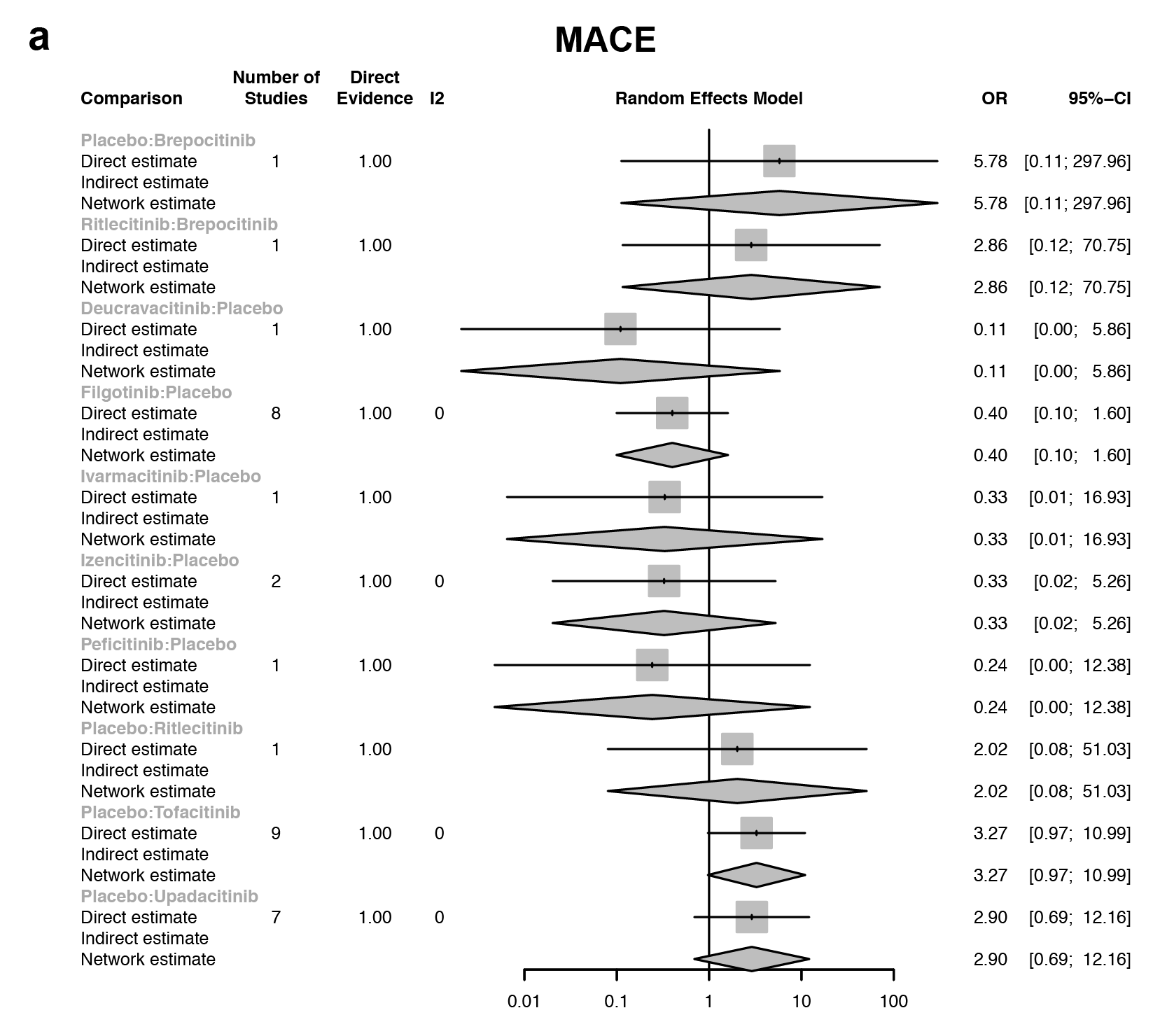


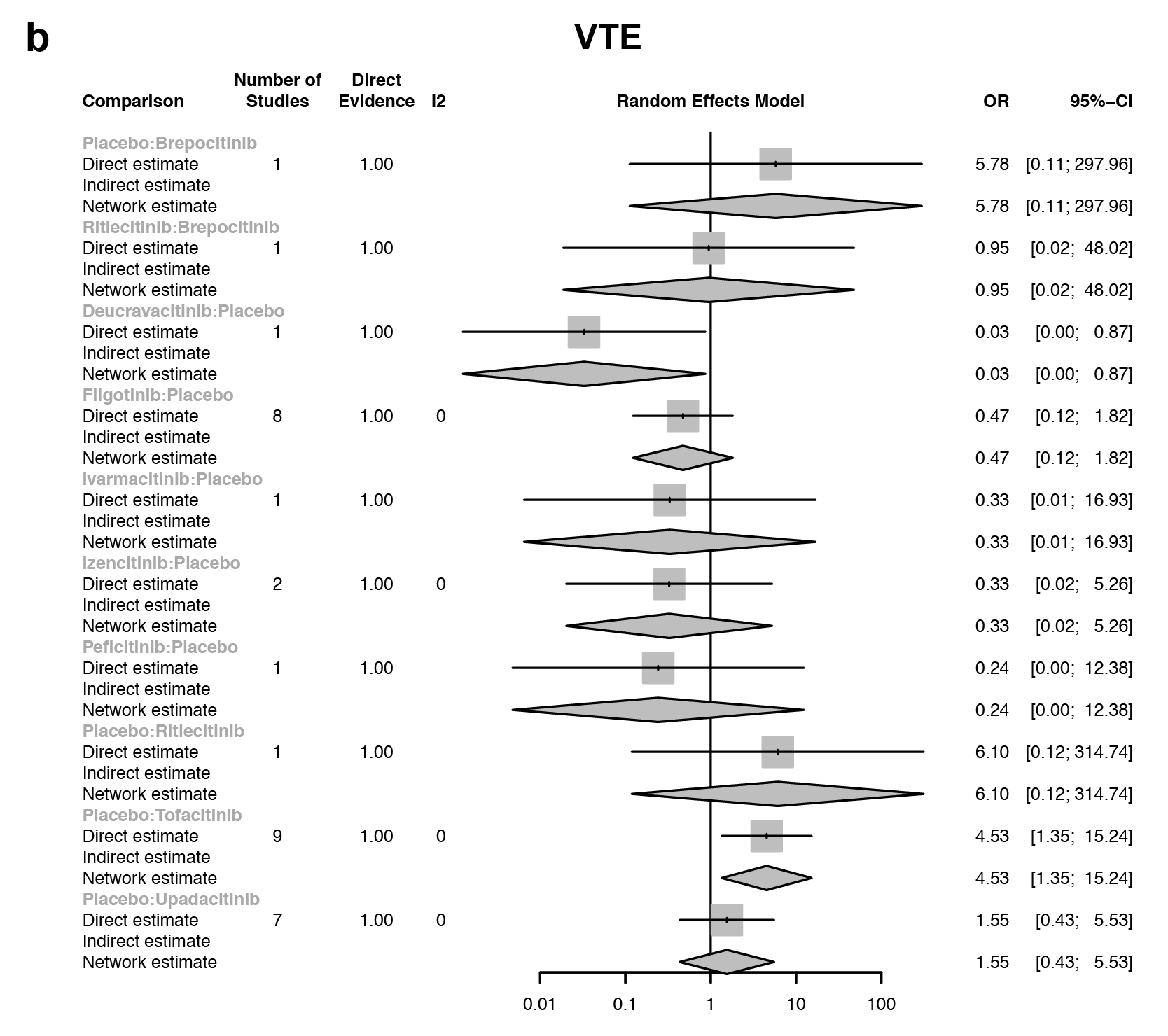


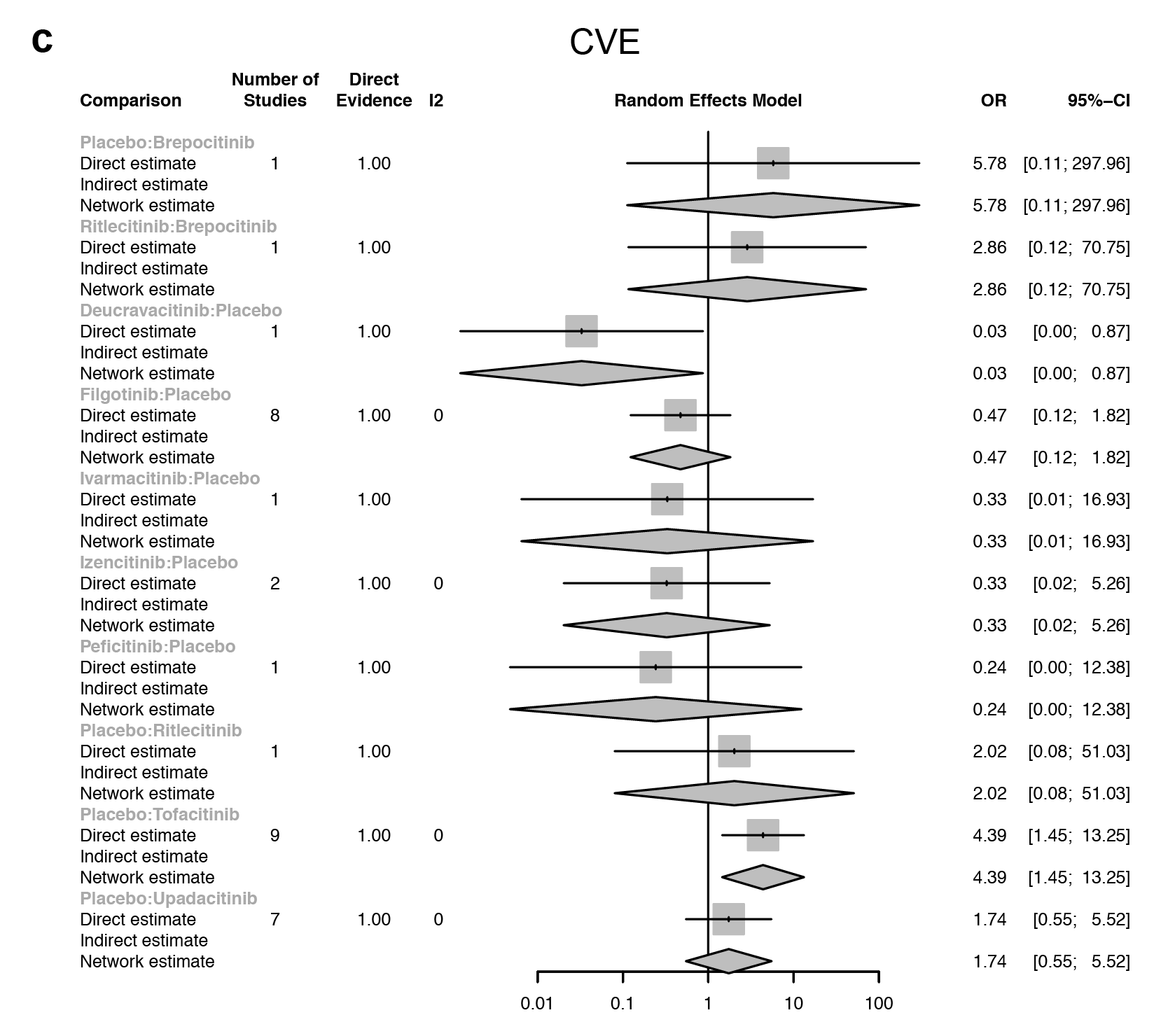


**Supplementary figure 16 Local inconsistency analyses of comparisons (with dose consideration) for MACE (a), VTE (b) and CVE (c)**


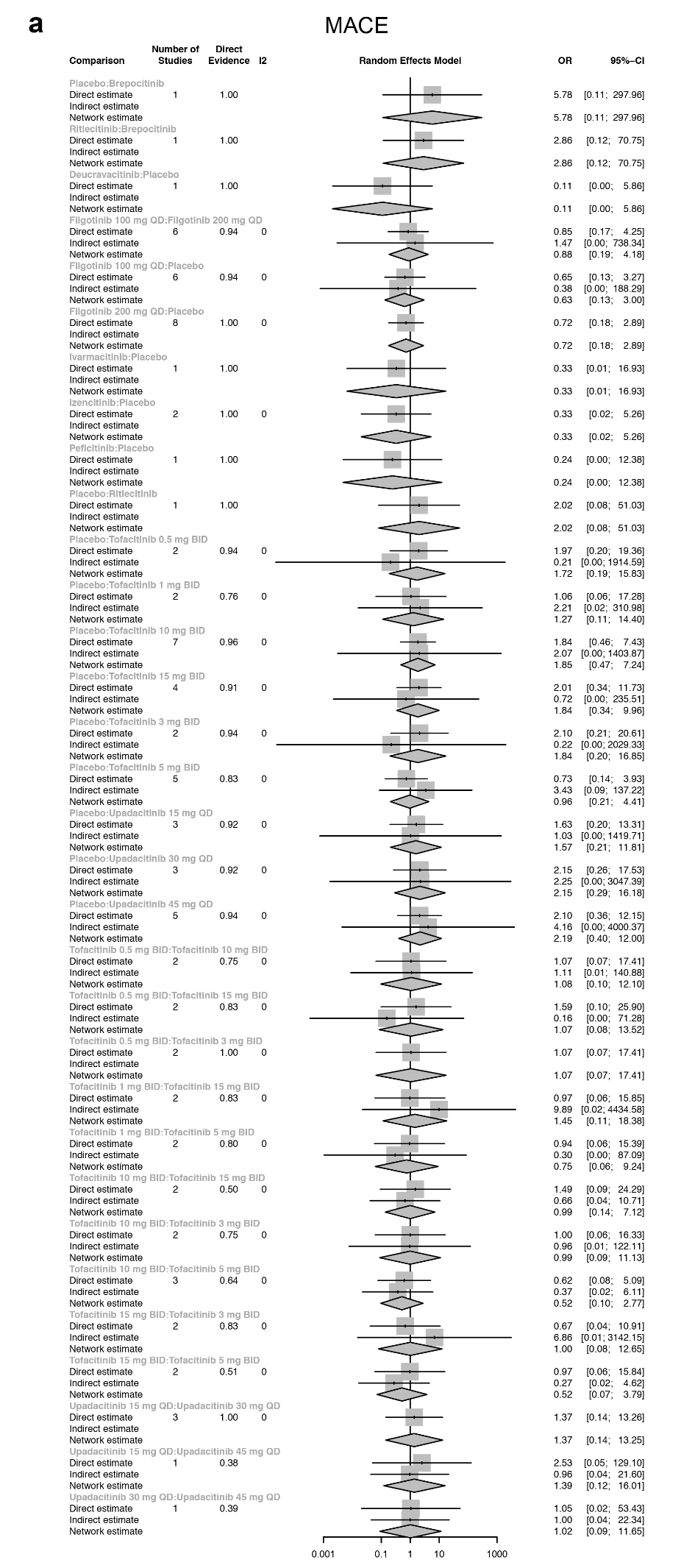


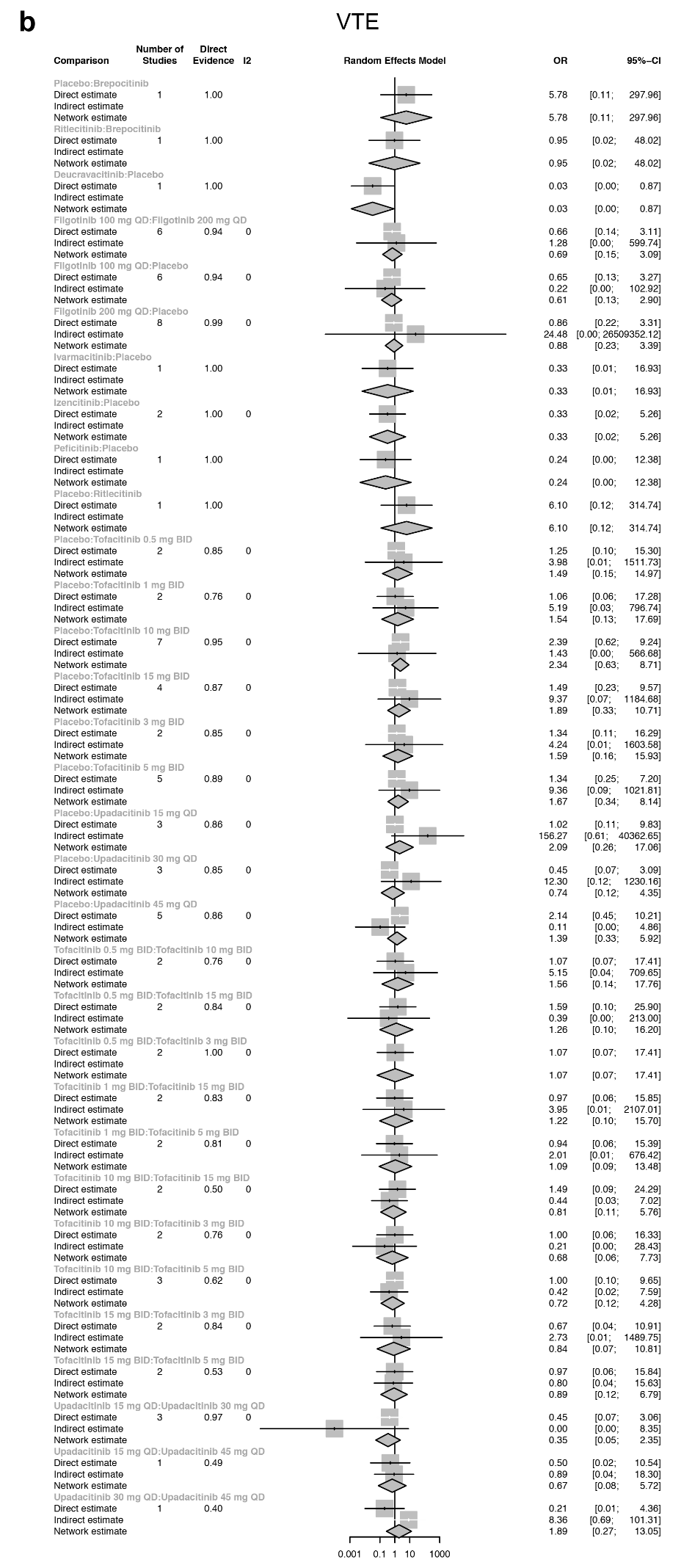


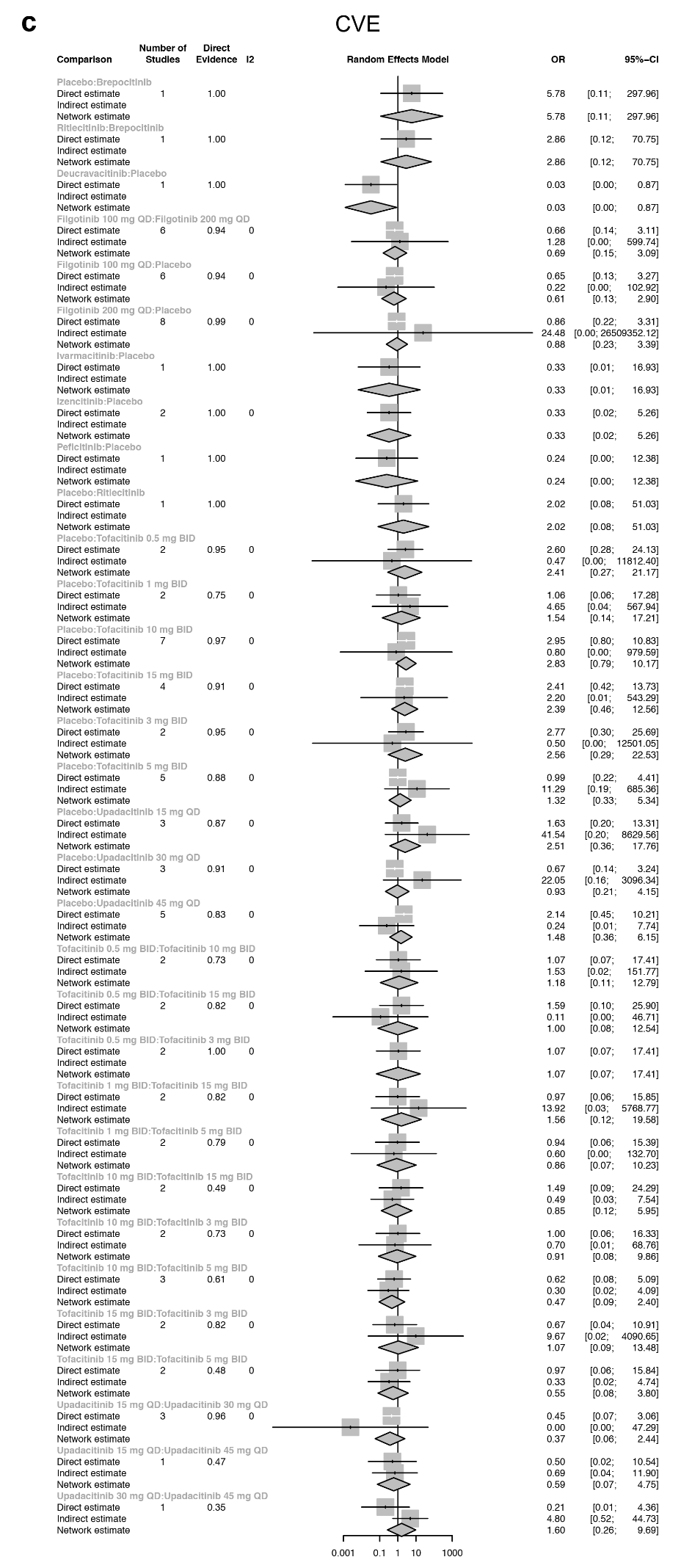


**Supplementary figure 17 Forest plots of the sensitivity analysis (no high risk of bias trial) (without dose consideration) for MACE (a), VTE (b) and CVE (c), as well as (with dose consideration) for MACE (d), VTE (e) and CVE (f)**


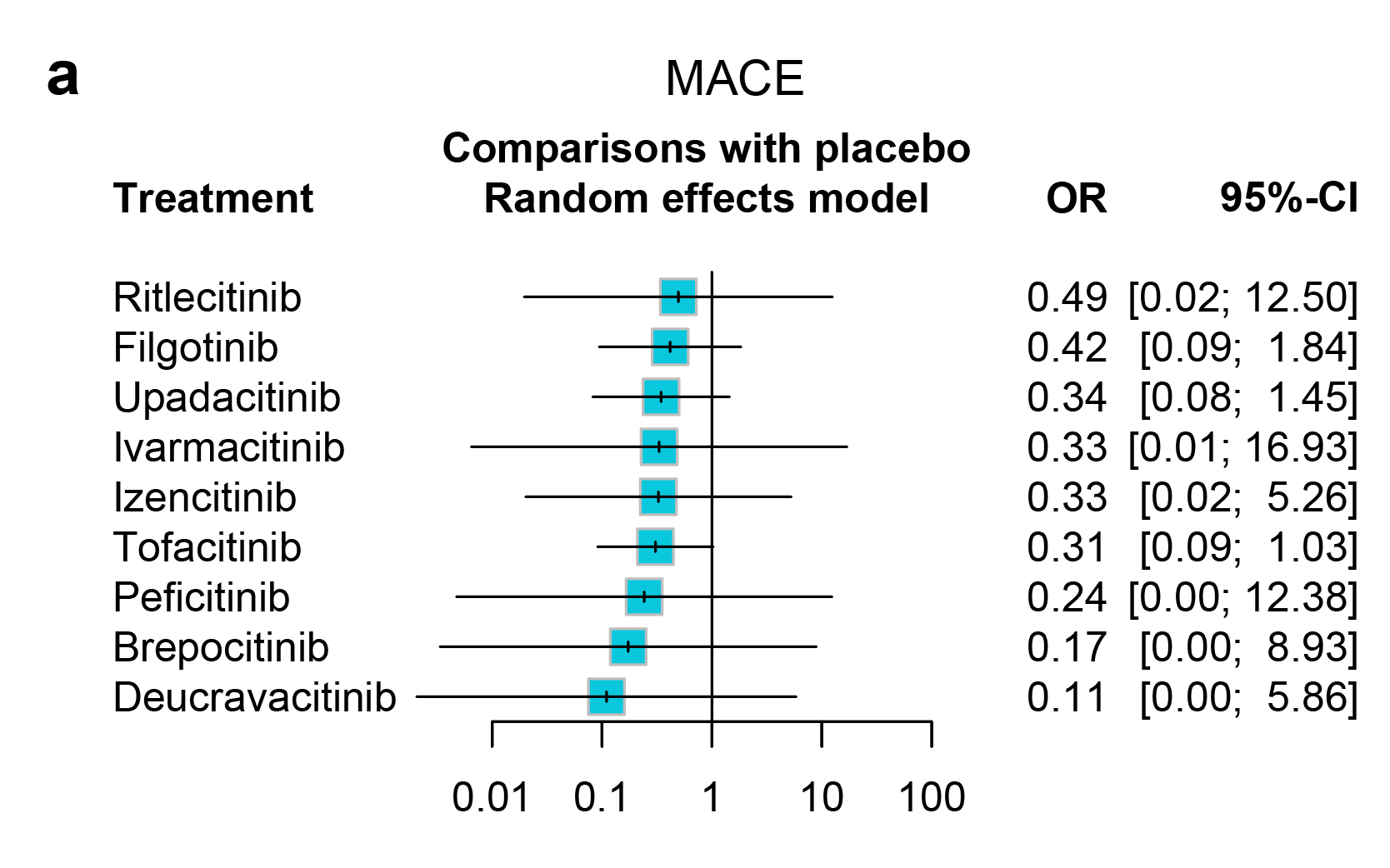


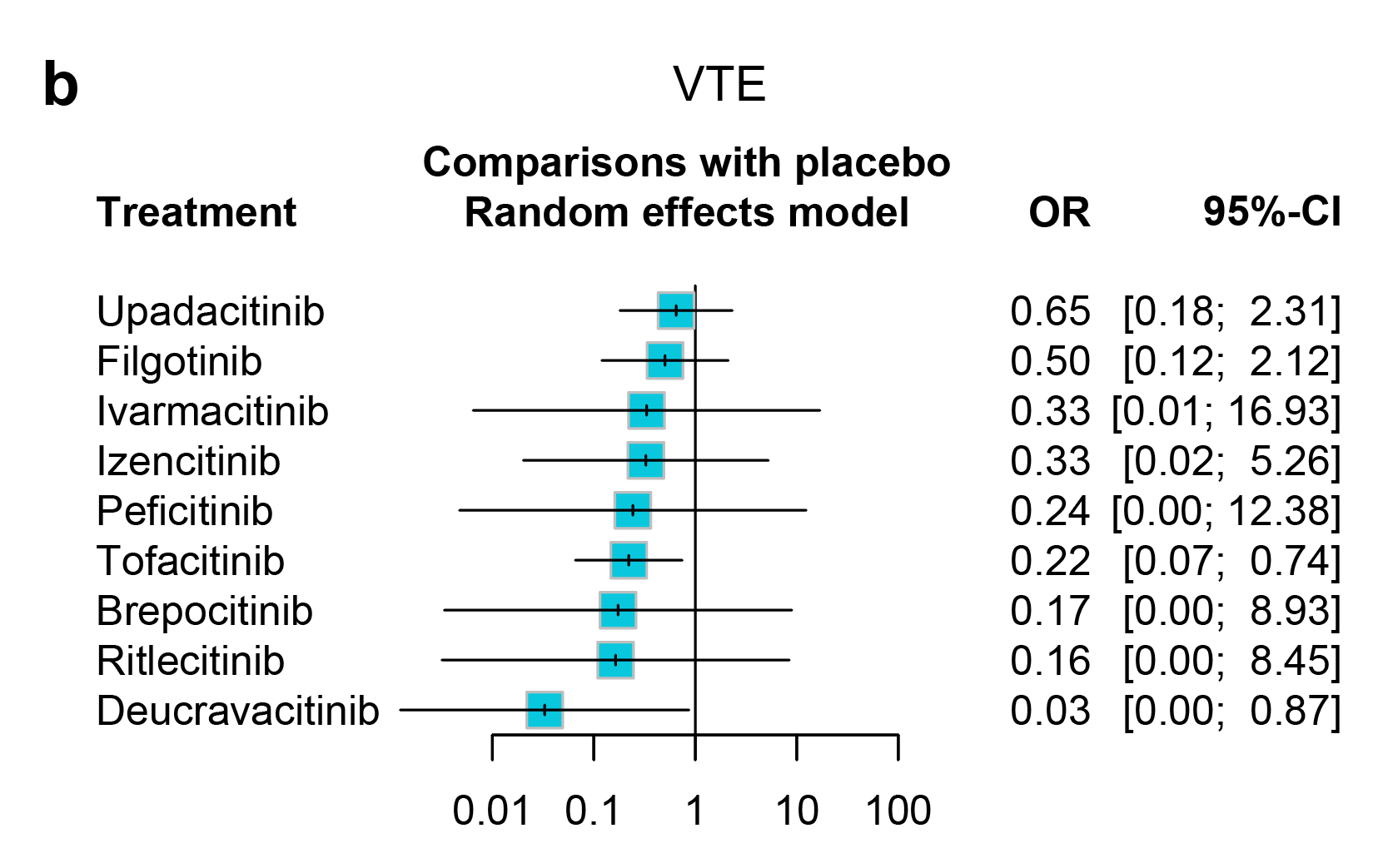


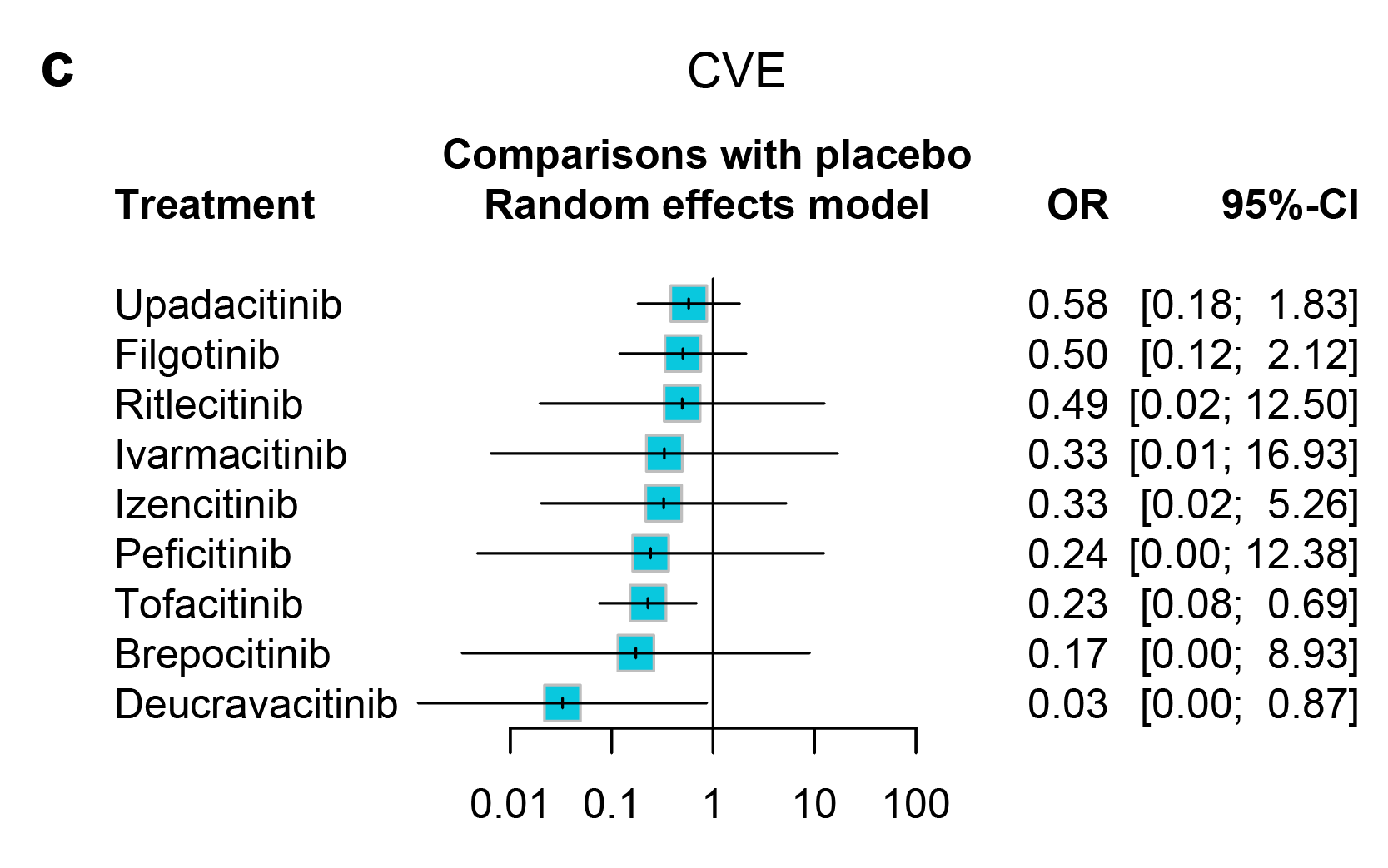


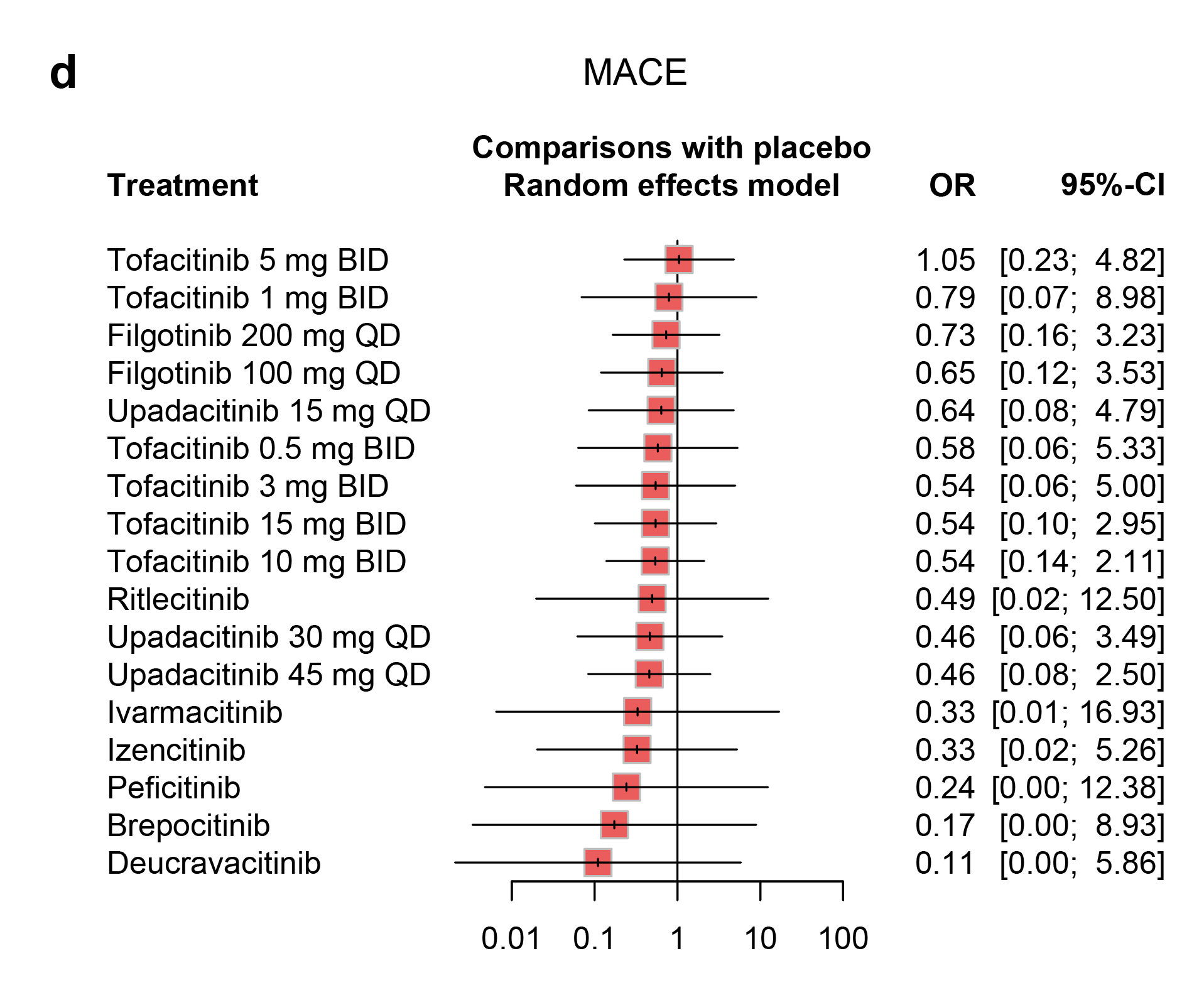


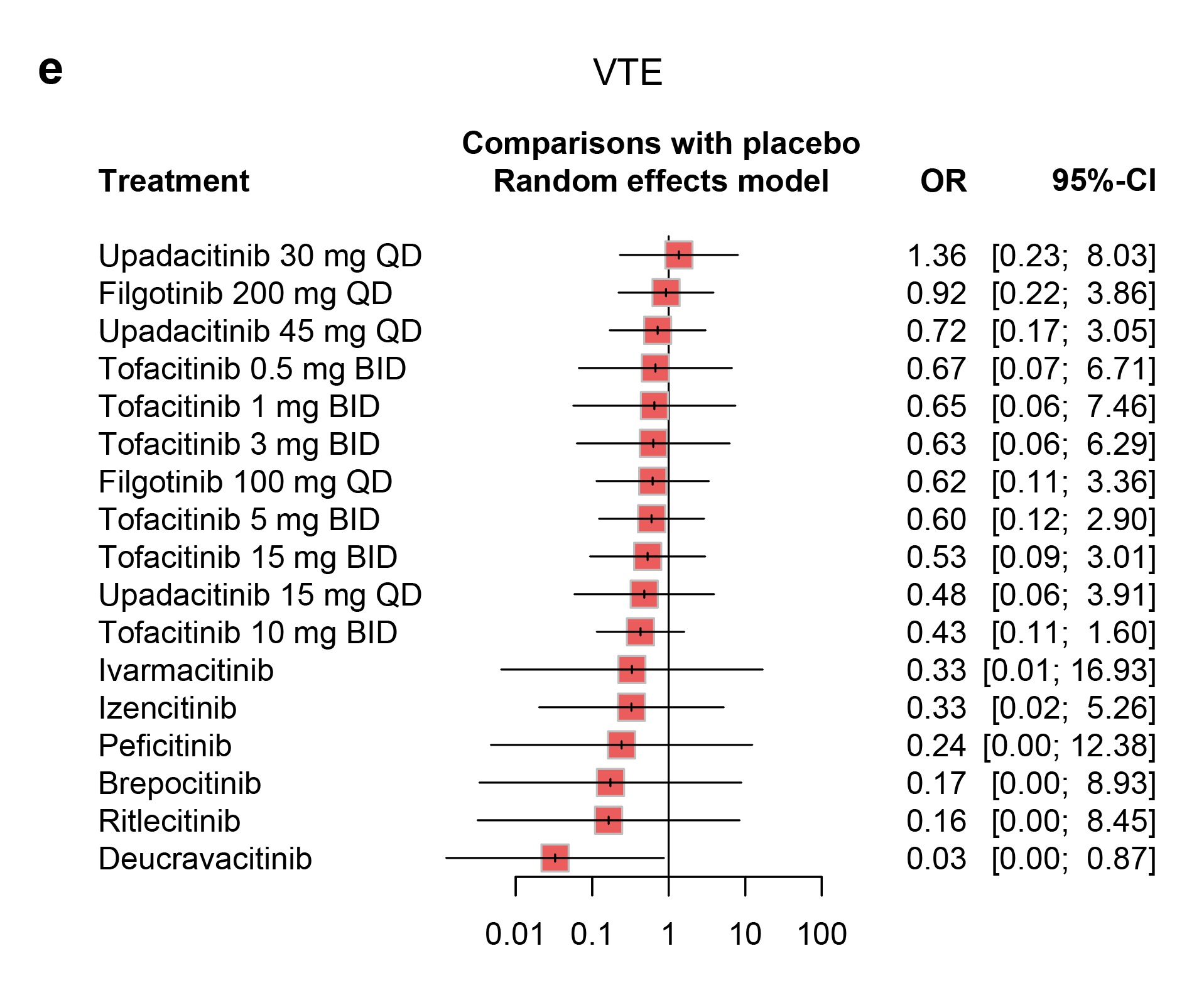


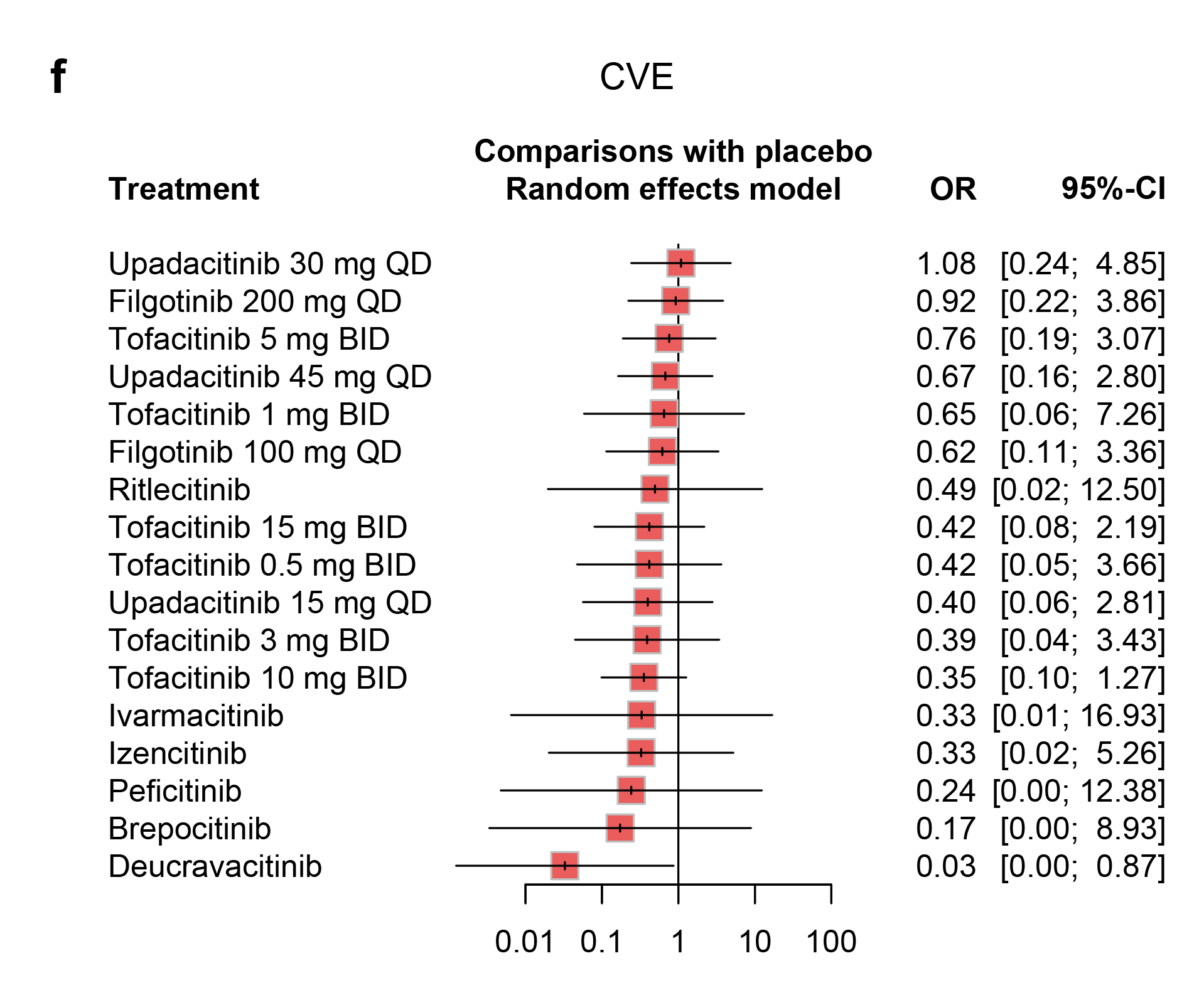


**Supplementary figure** **18 Forest plots of the subgroup analysis (UC patients) based on the IBD type (without dose consideration) for MACE (a), VTE (b) and CVE (c), as well as (with dose consideration) for MACE (d), VTE (e) and CVE (f)**


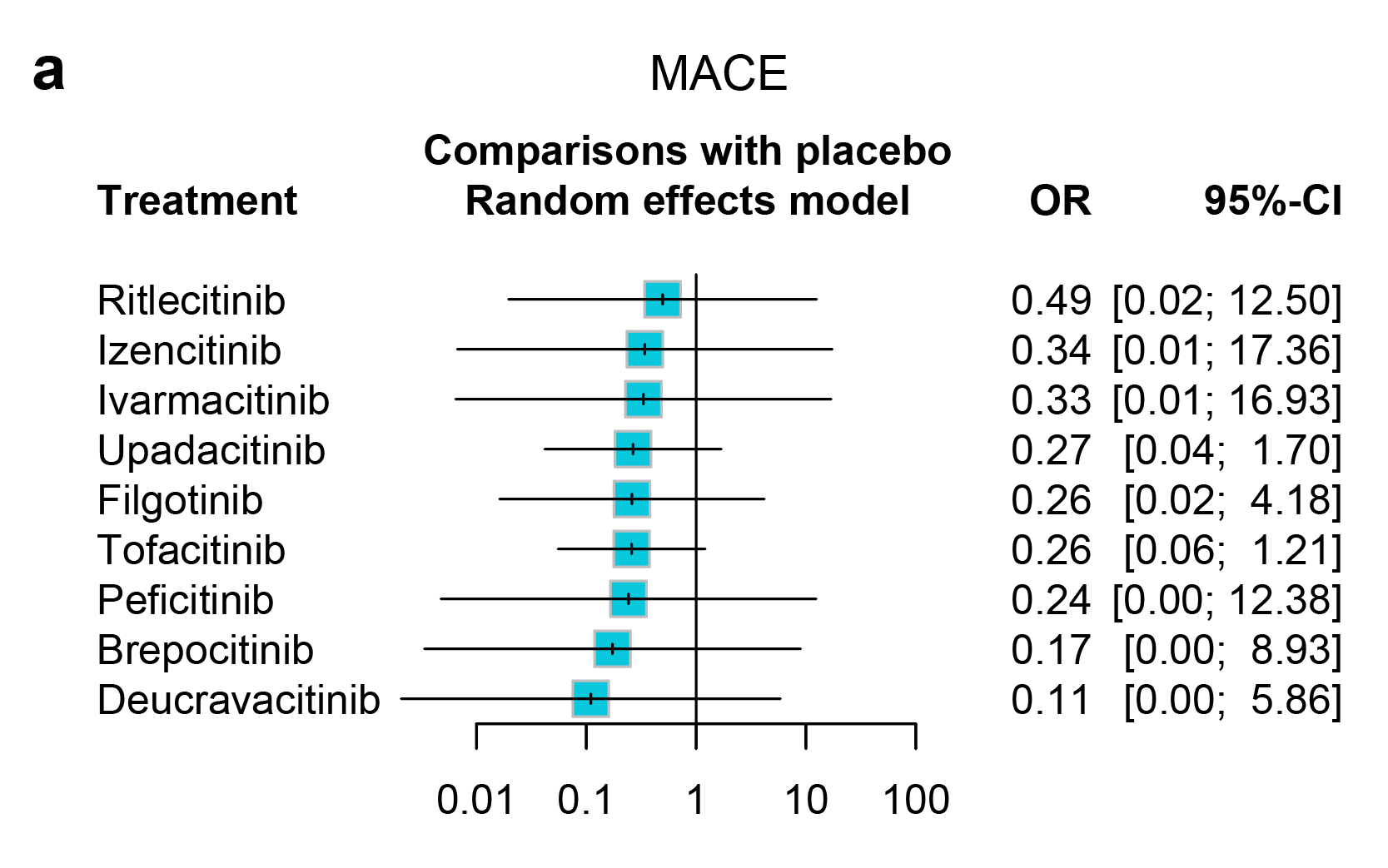


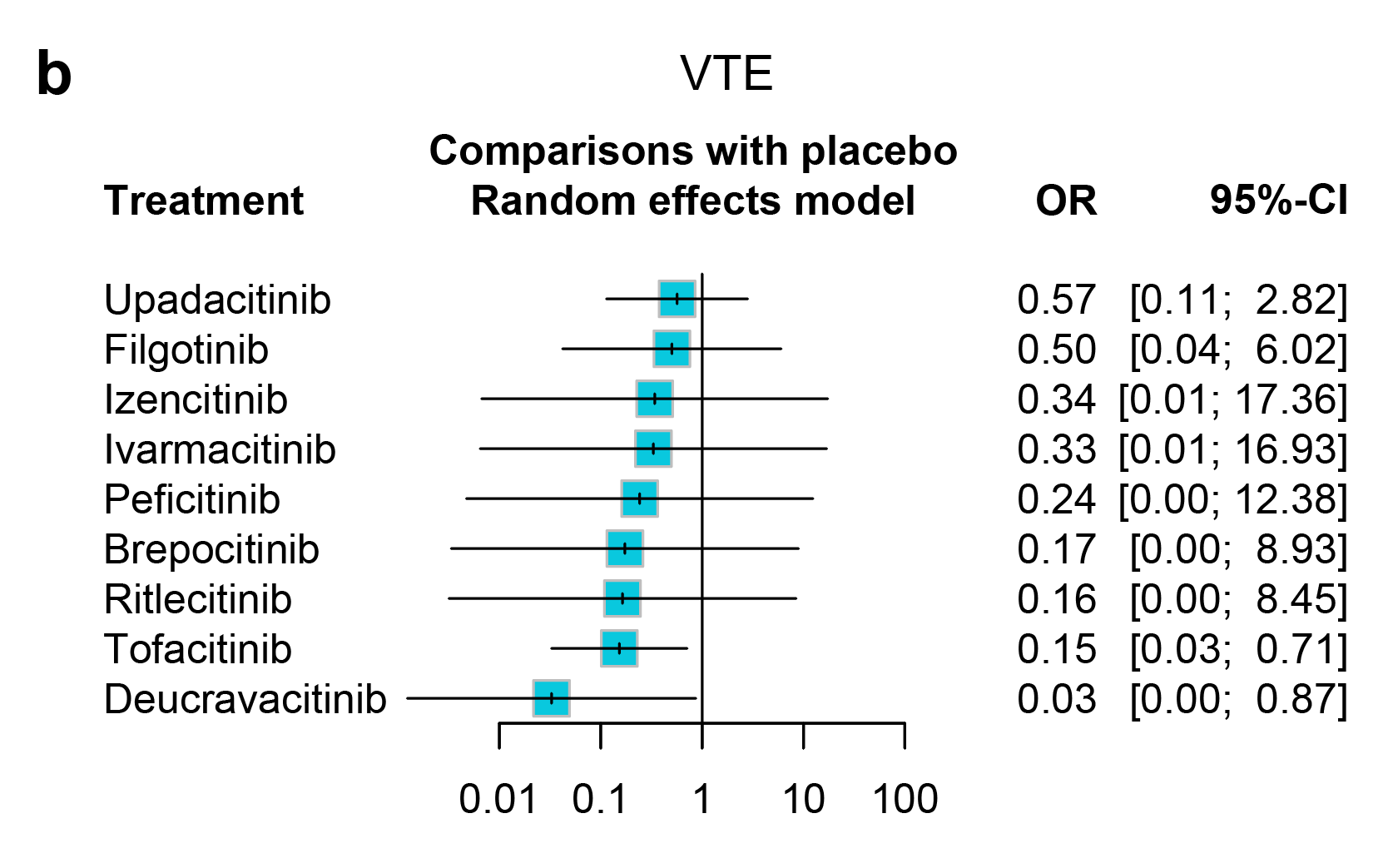


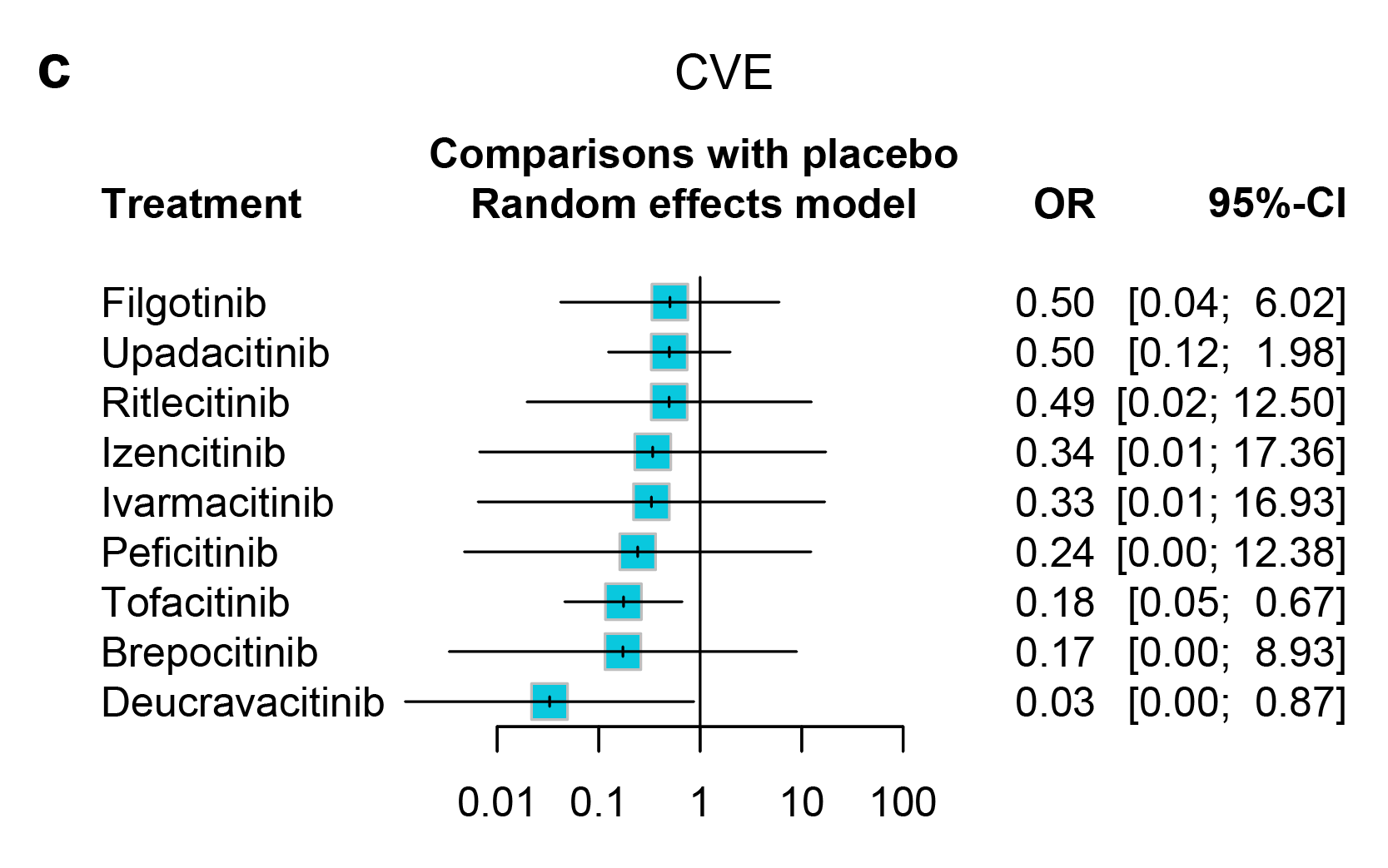


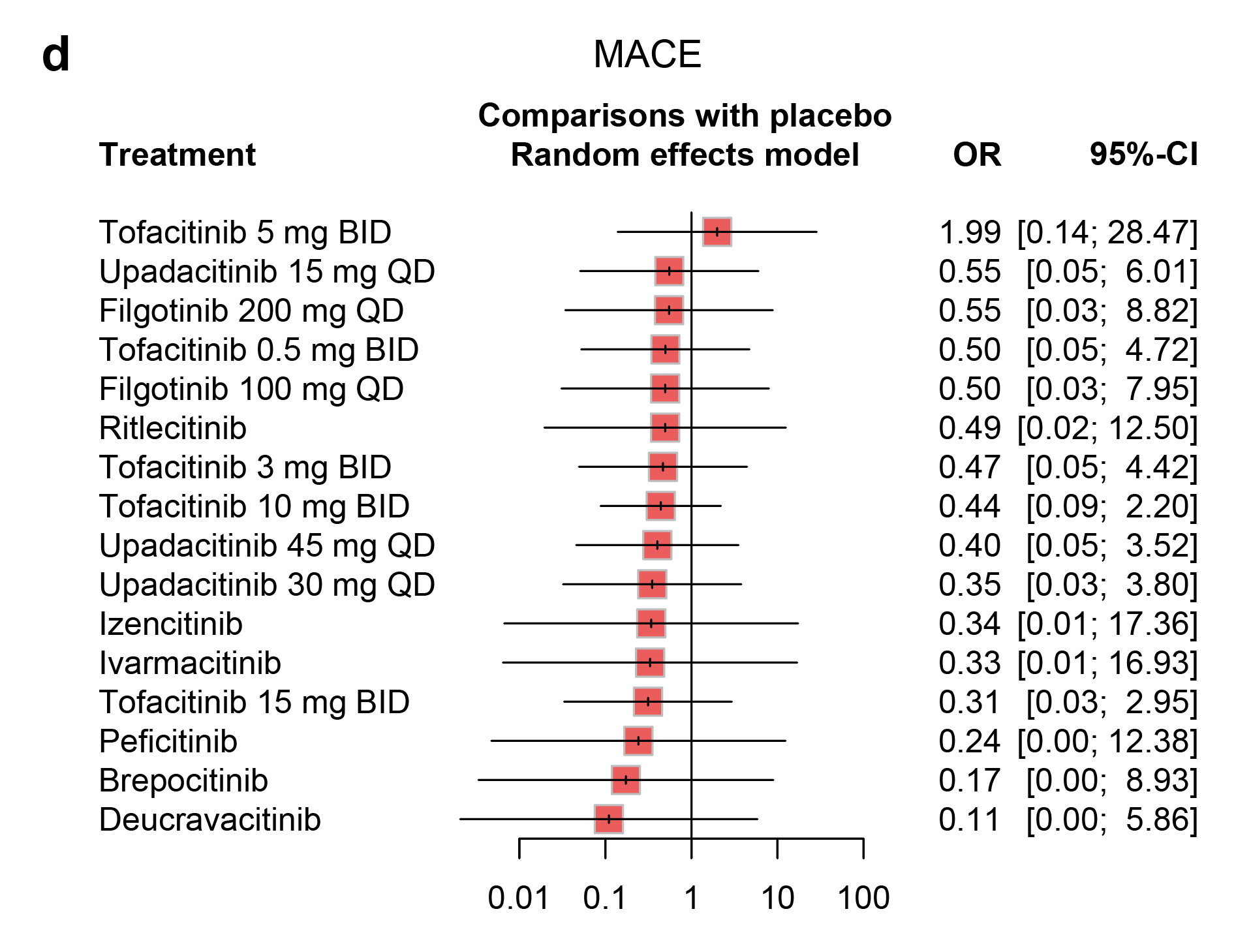


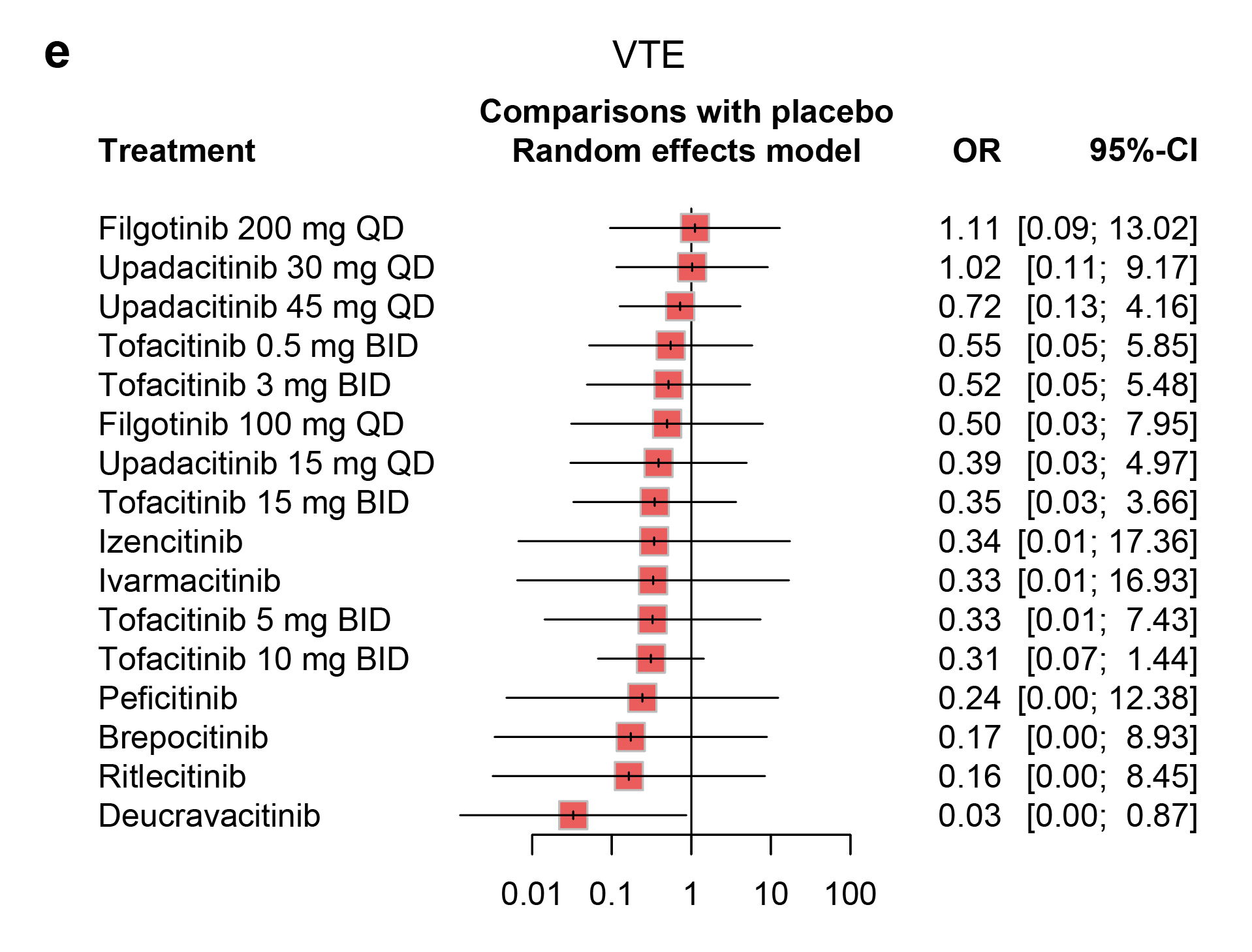


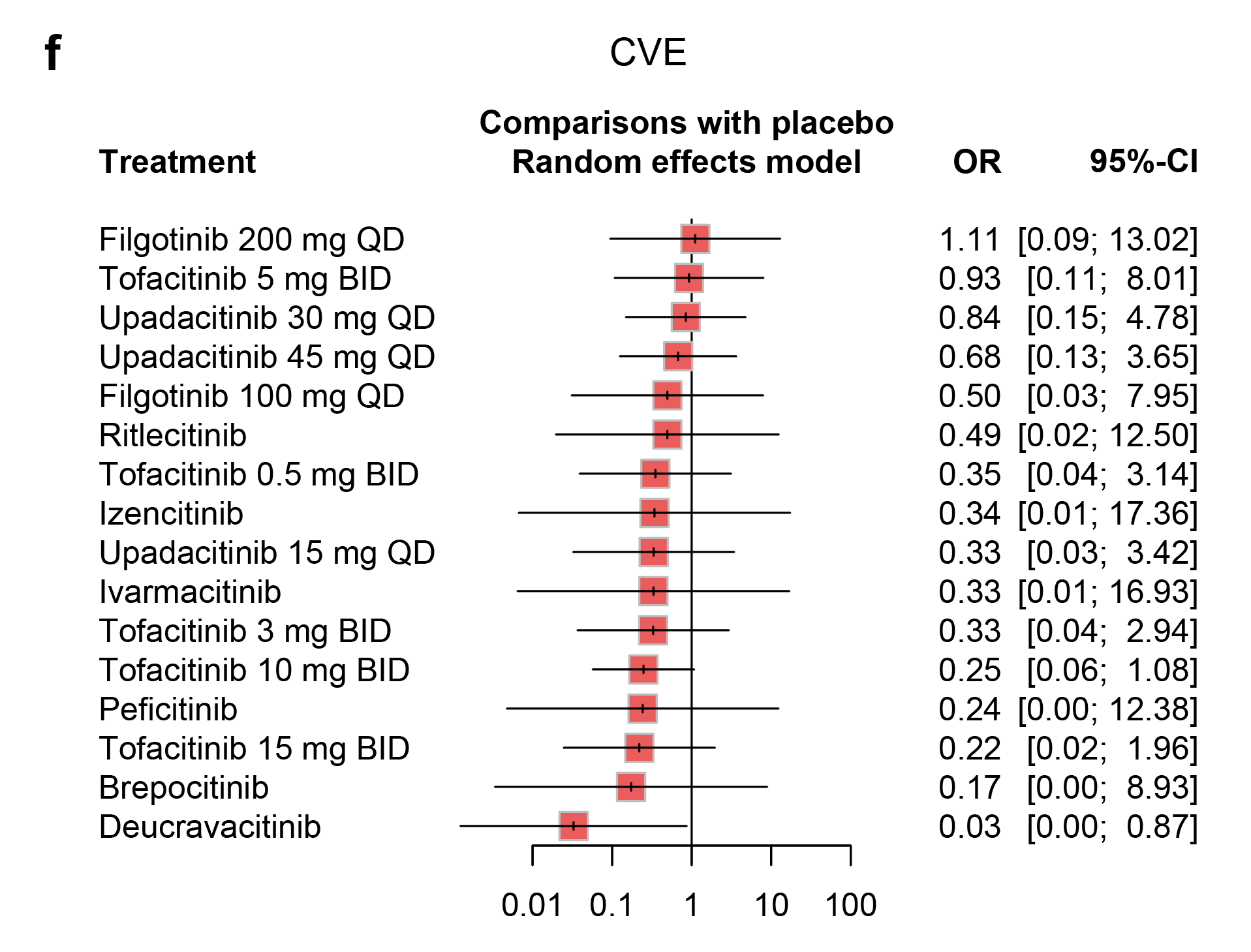


**Supplementary figure 19 Forest plots of the subgroup analysis (CD patients) based on the IBD type (without dose consideration) for MACE (a), VTE (b) and CVE (c), as well as (with dose consideration) for MACE (d), VTE (e) and CVE (f)**


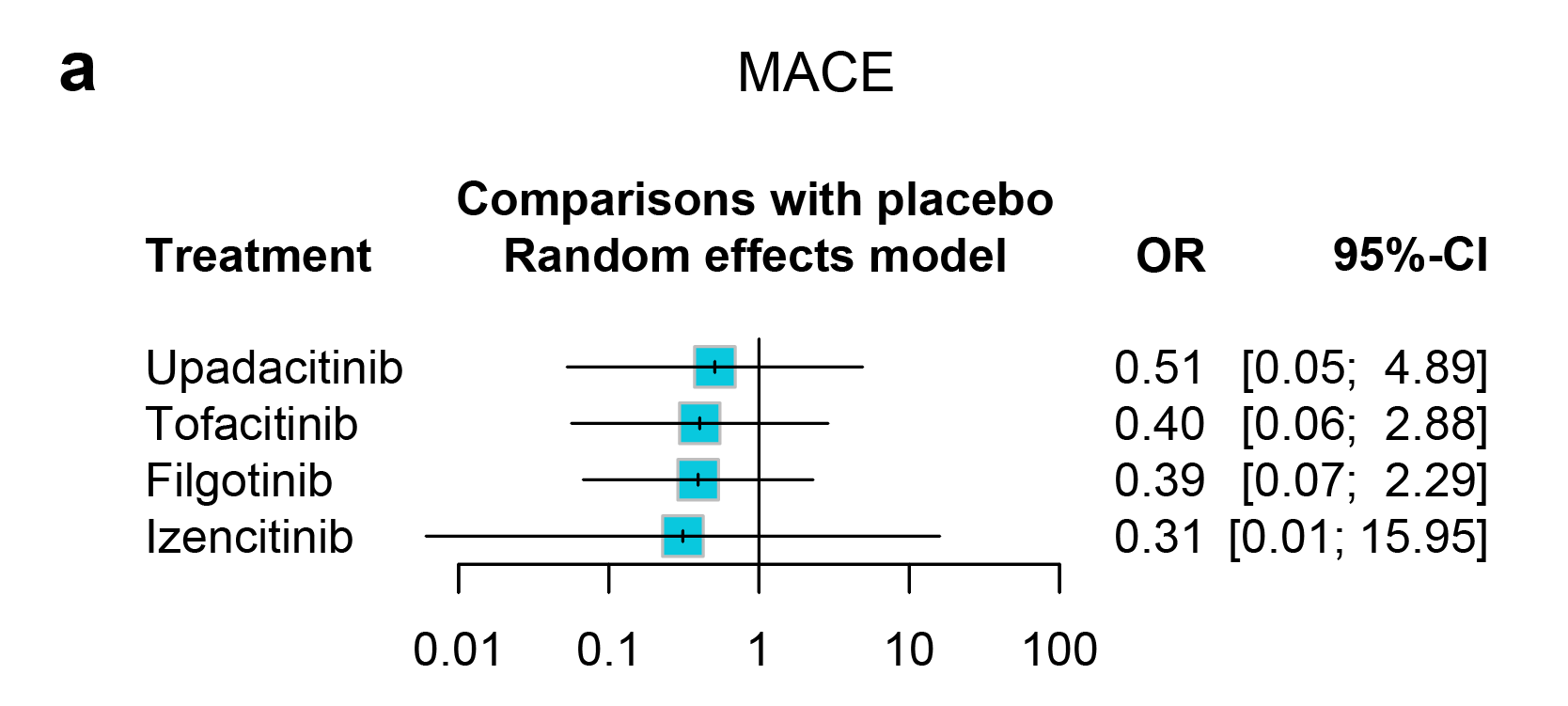


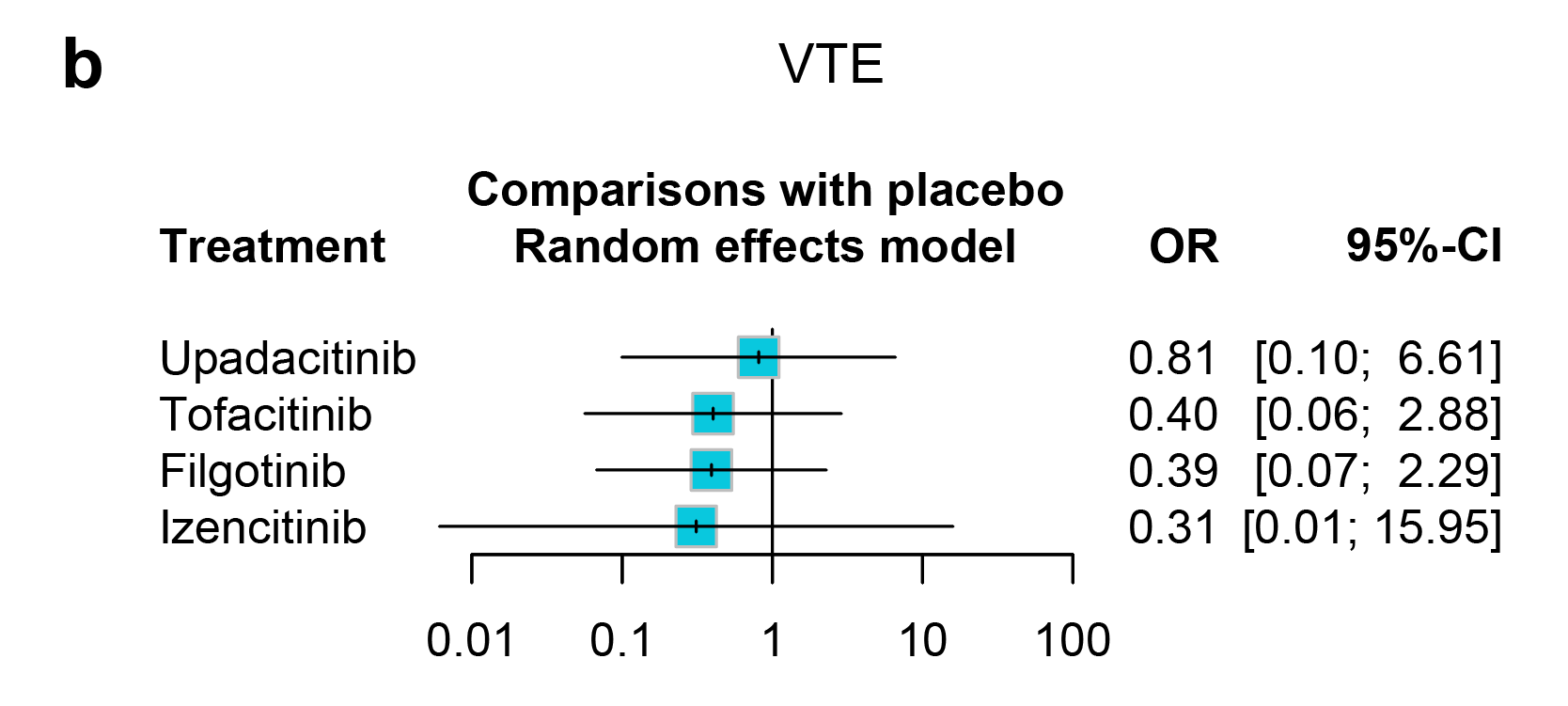


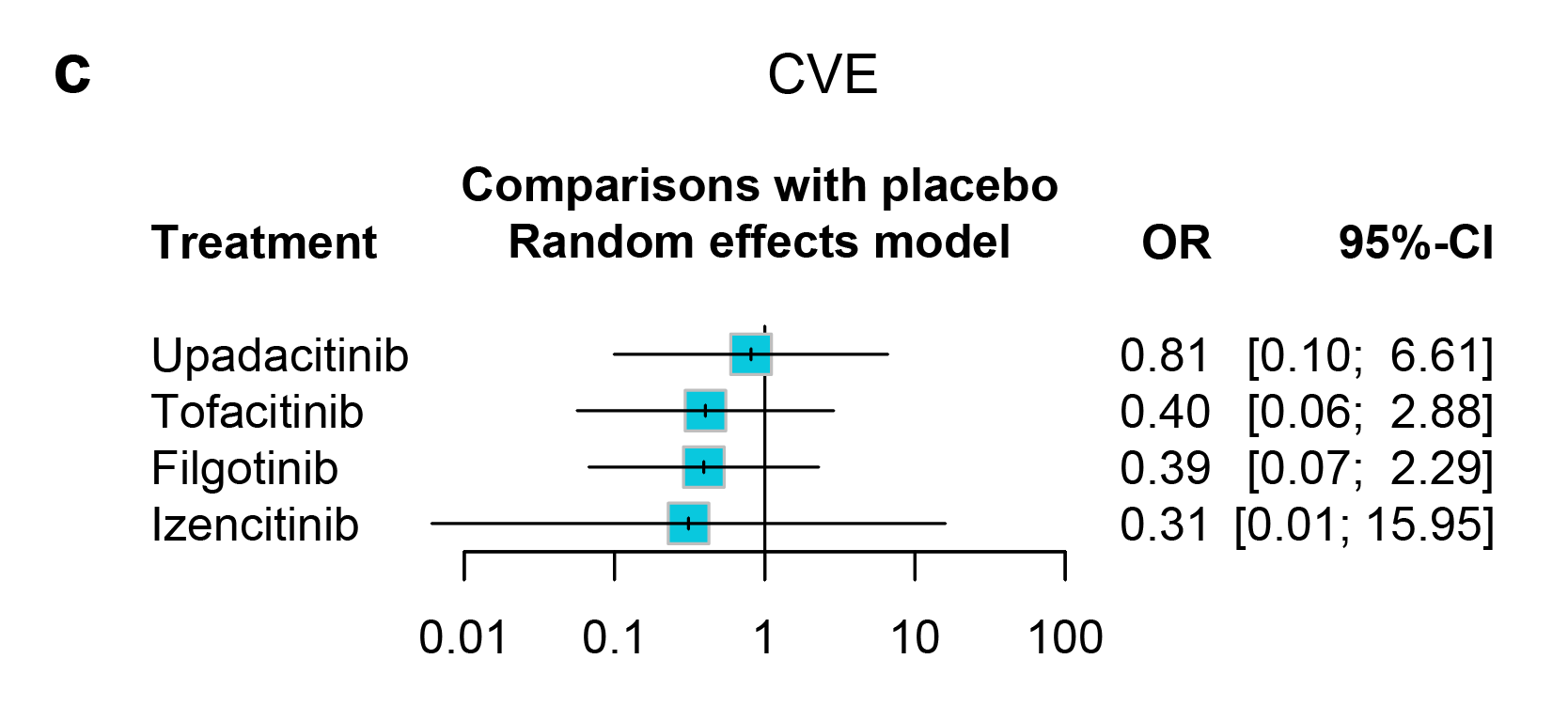


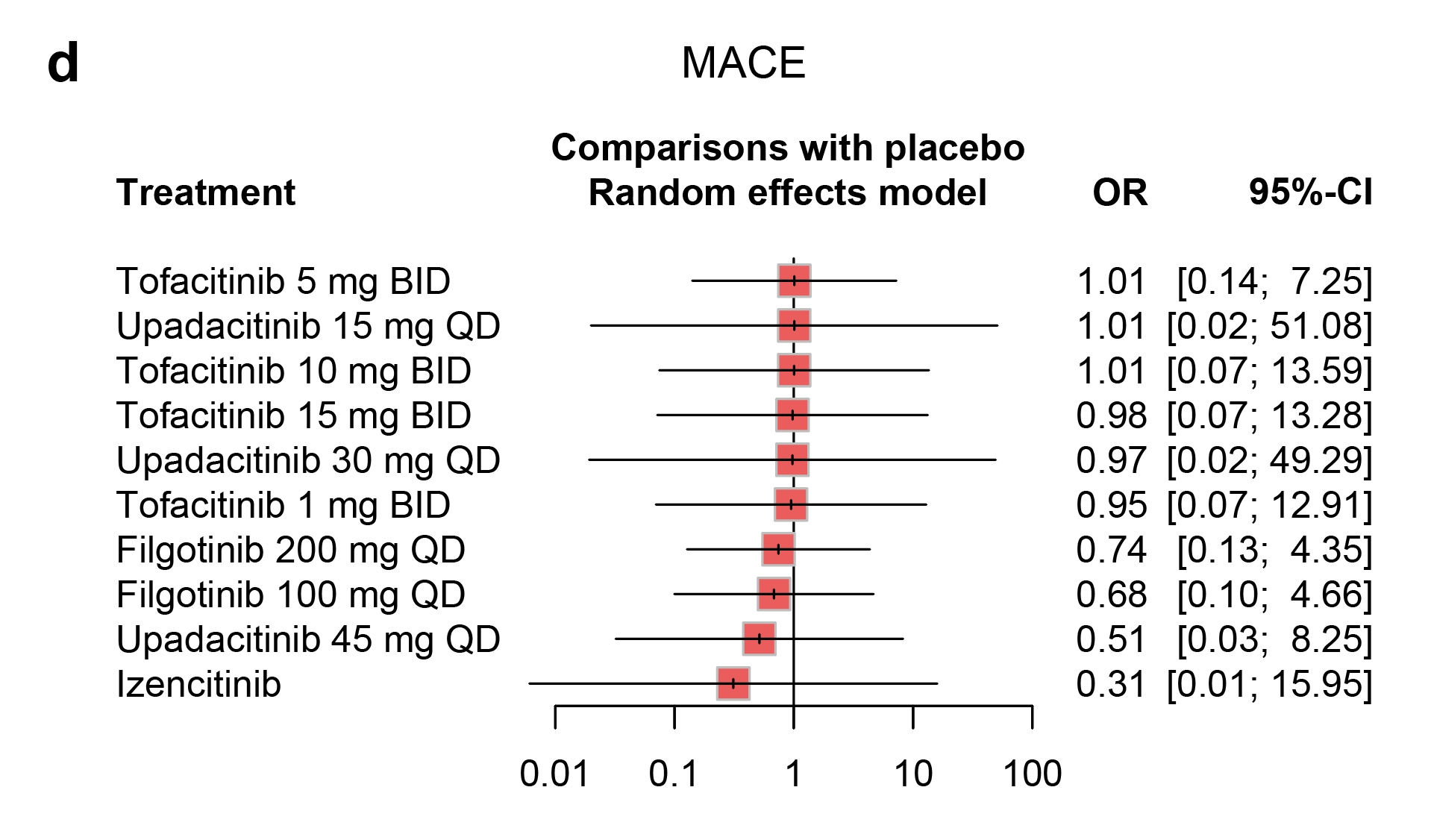


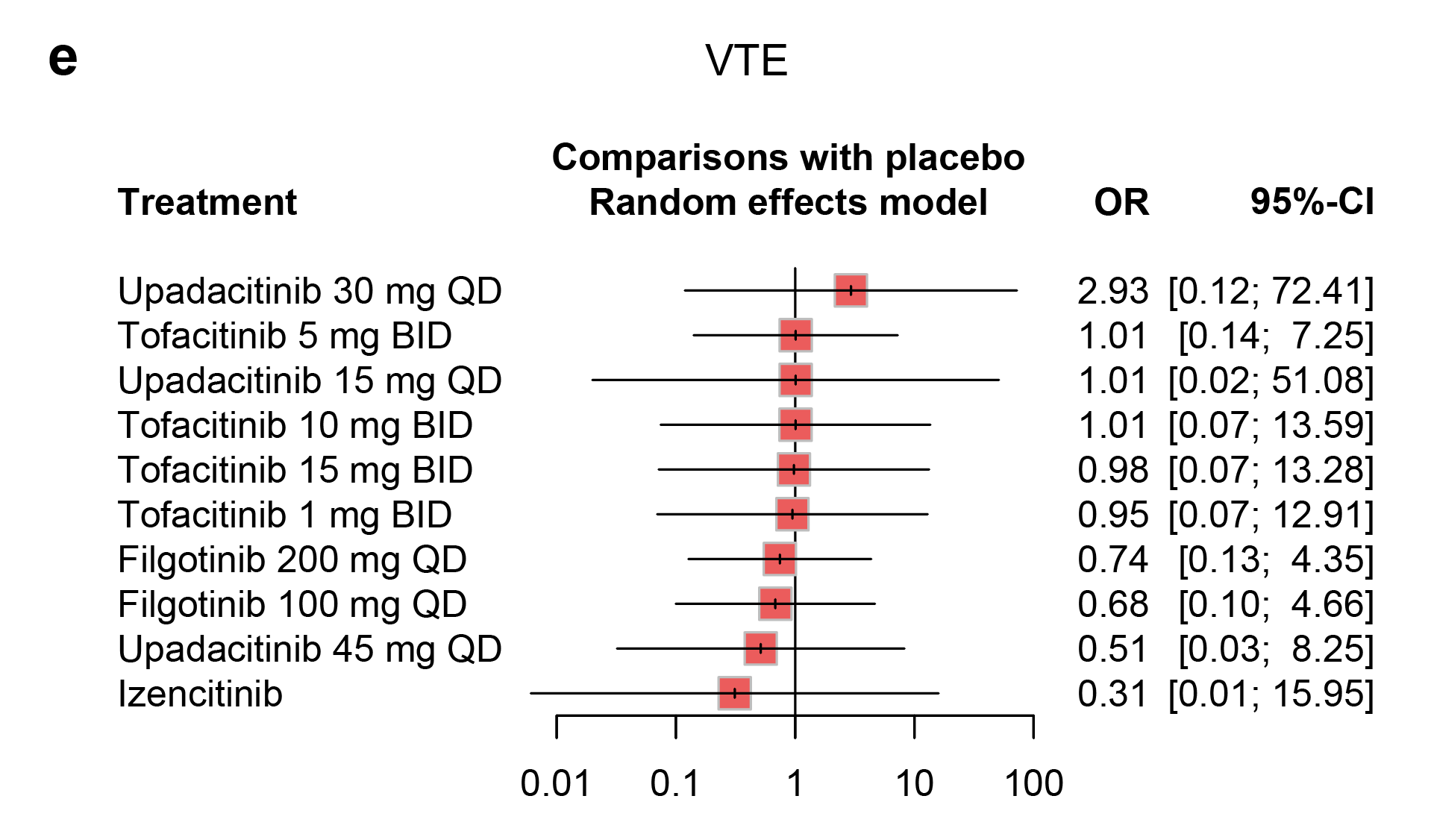


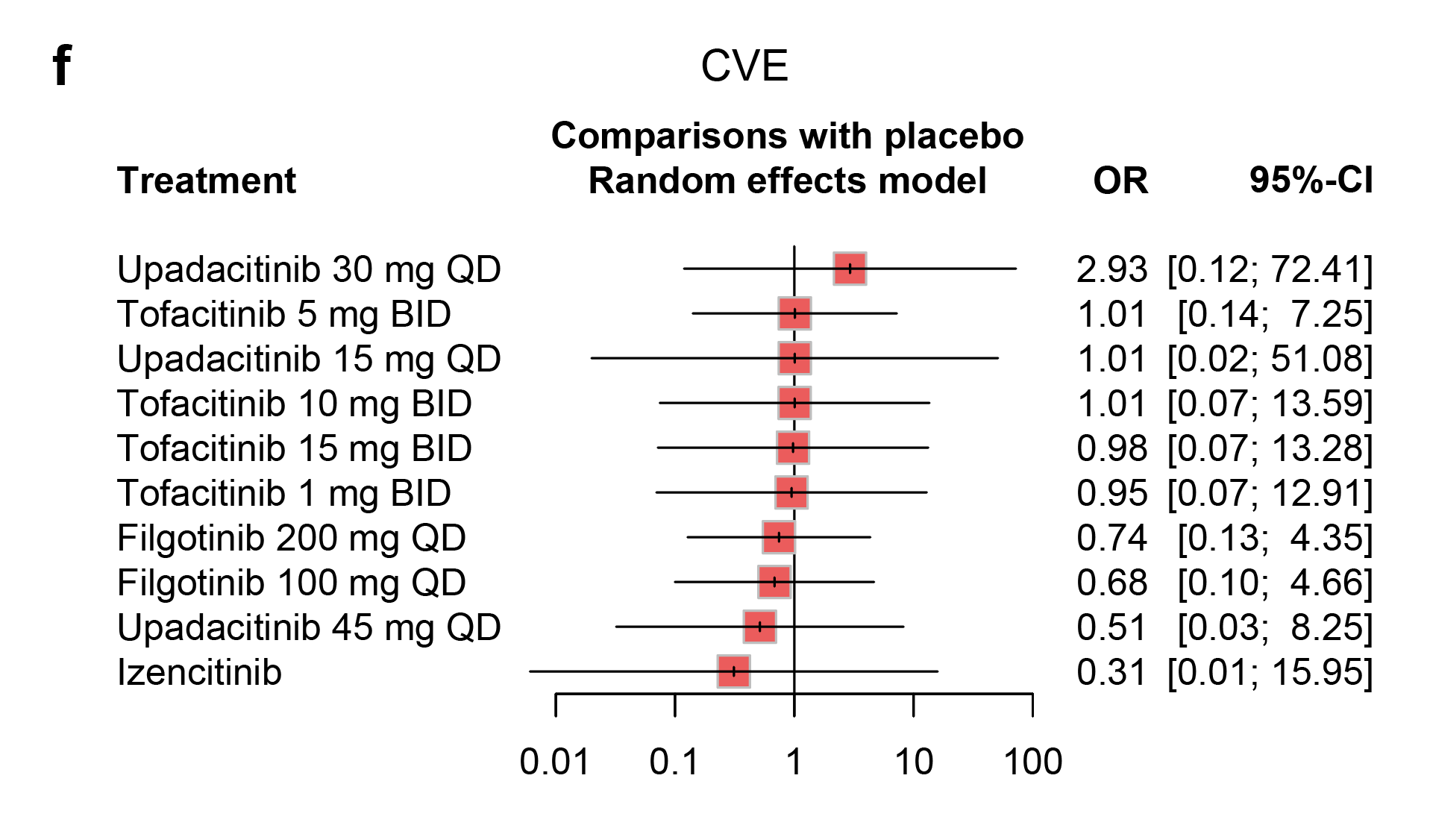


**Supplementary figure 20 Forest plots of the subgroup analysis (the induction** **phase) based on the study phase (without dose consideration) for MACE (a), VTE (b) and CVE (c), as well as (with dose consideration) for MACE (d), VTE (e) and CVE (f)**

**
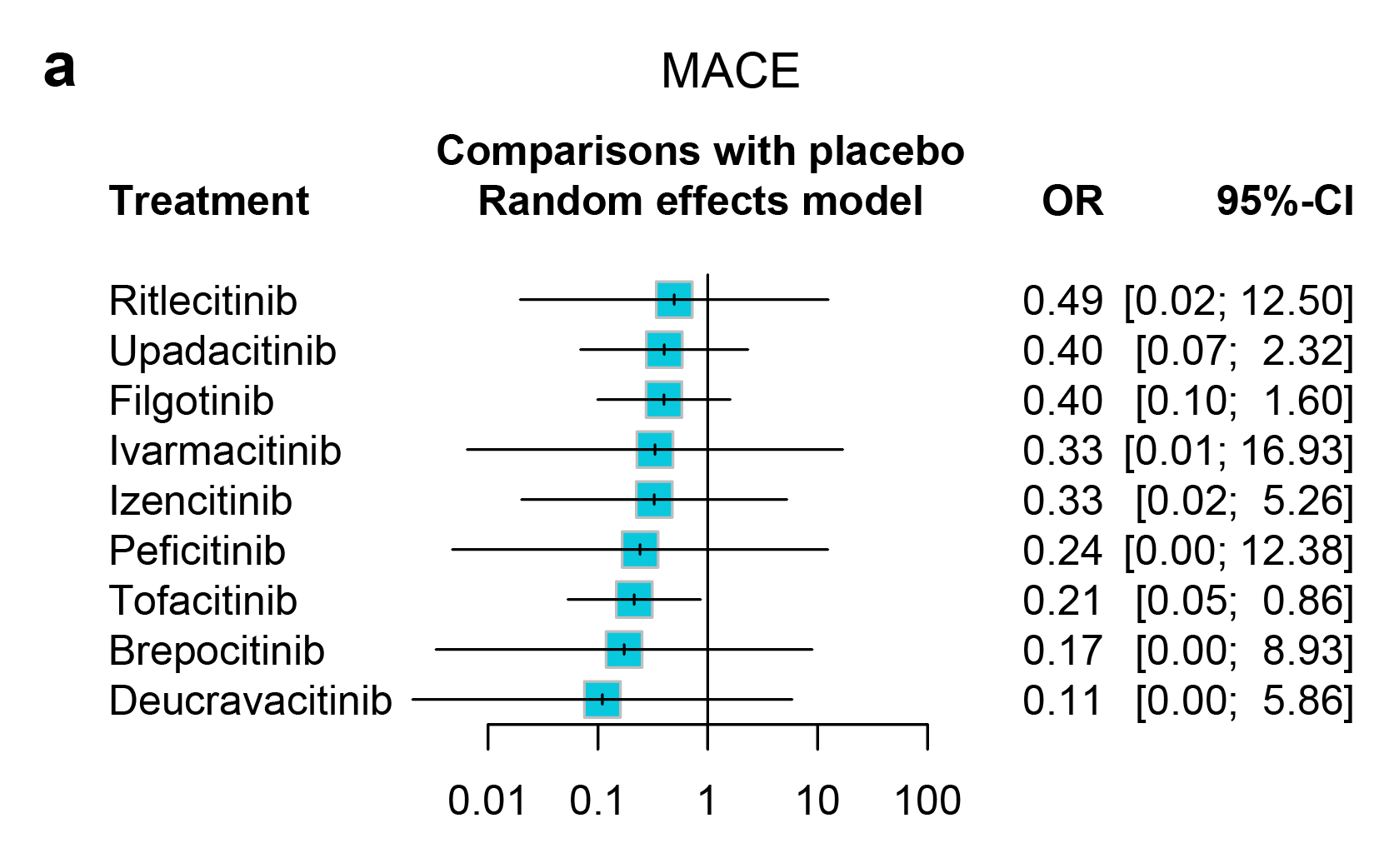
**


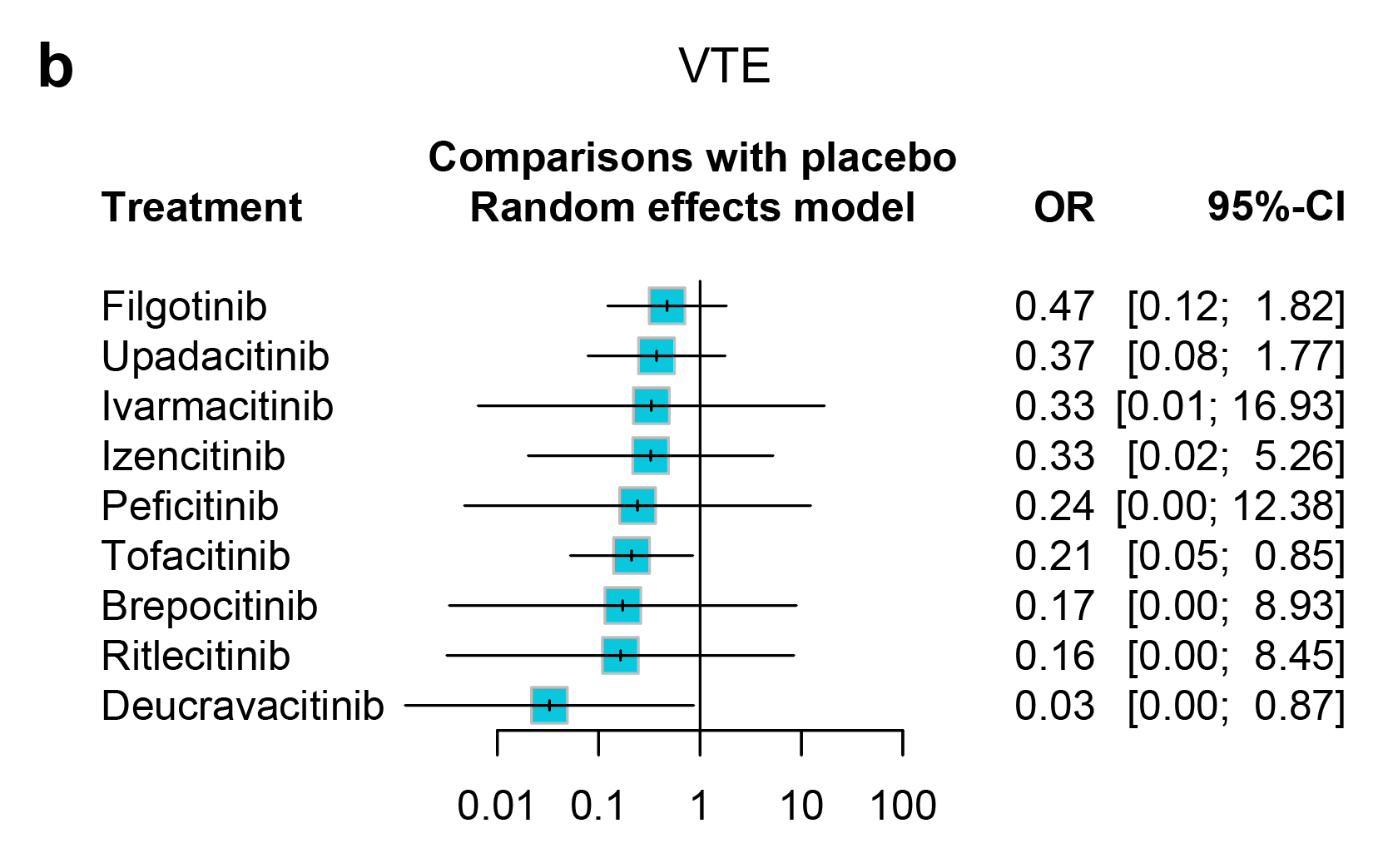


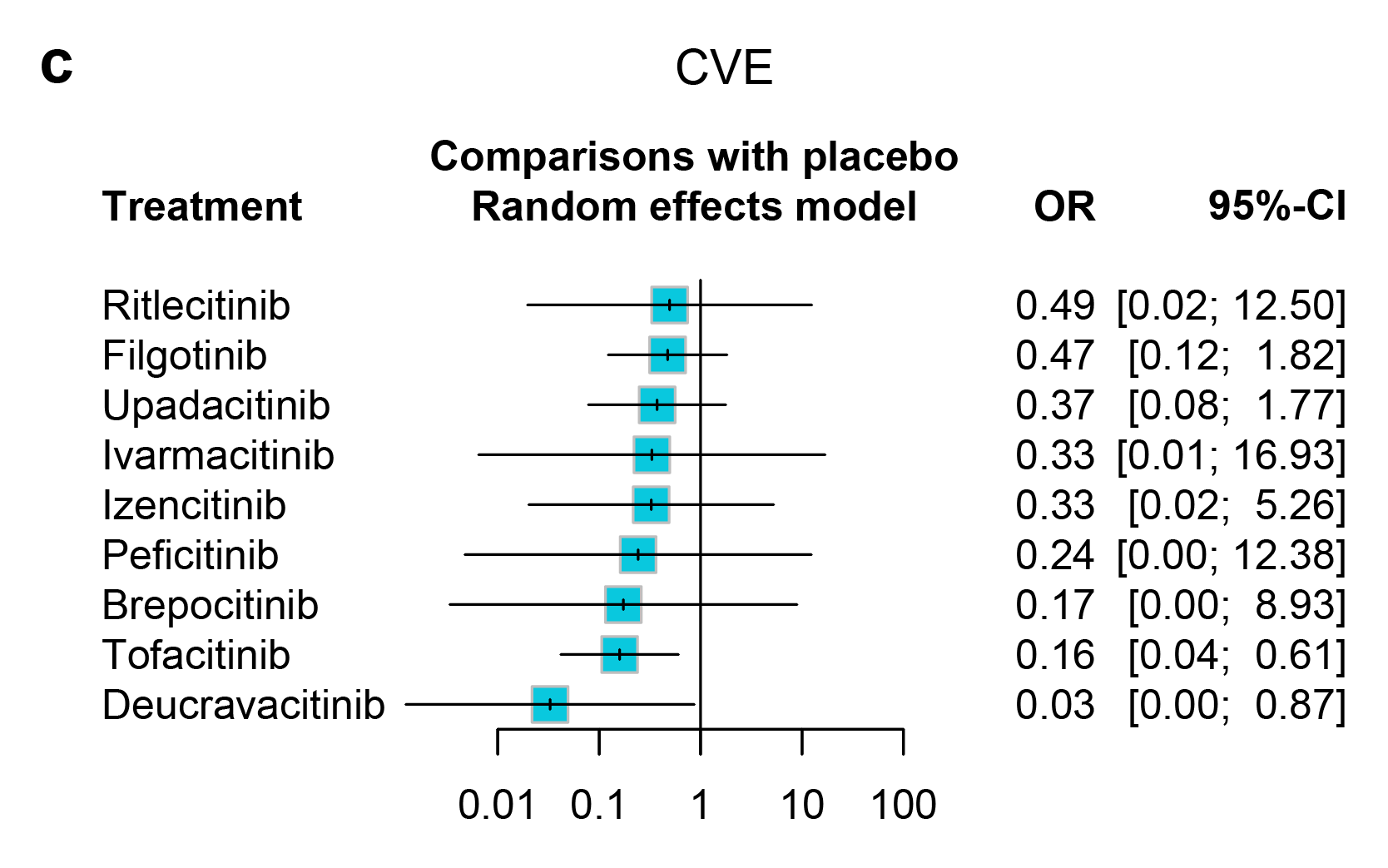


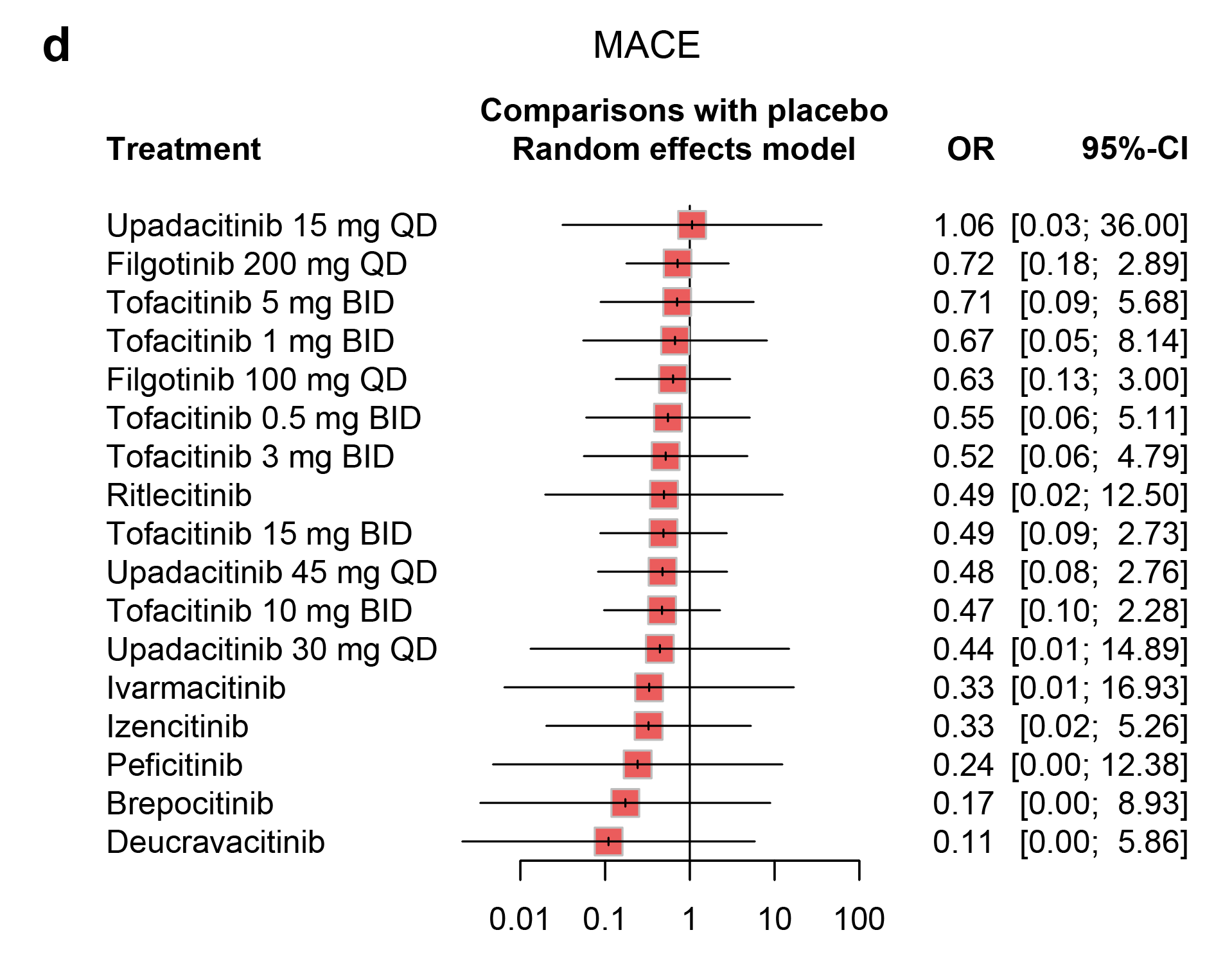


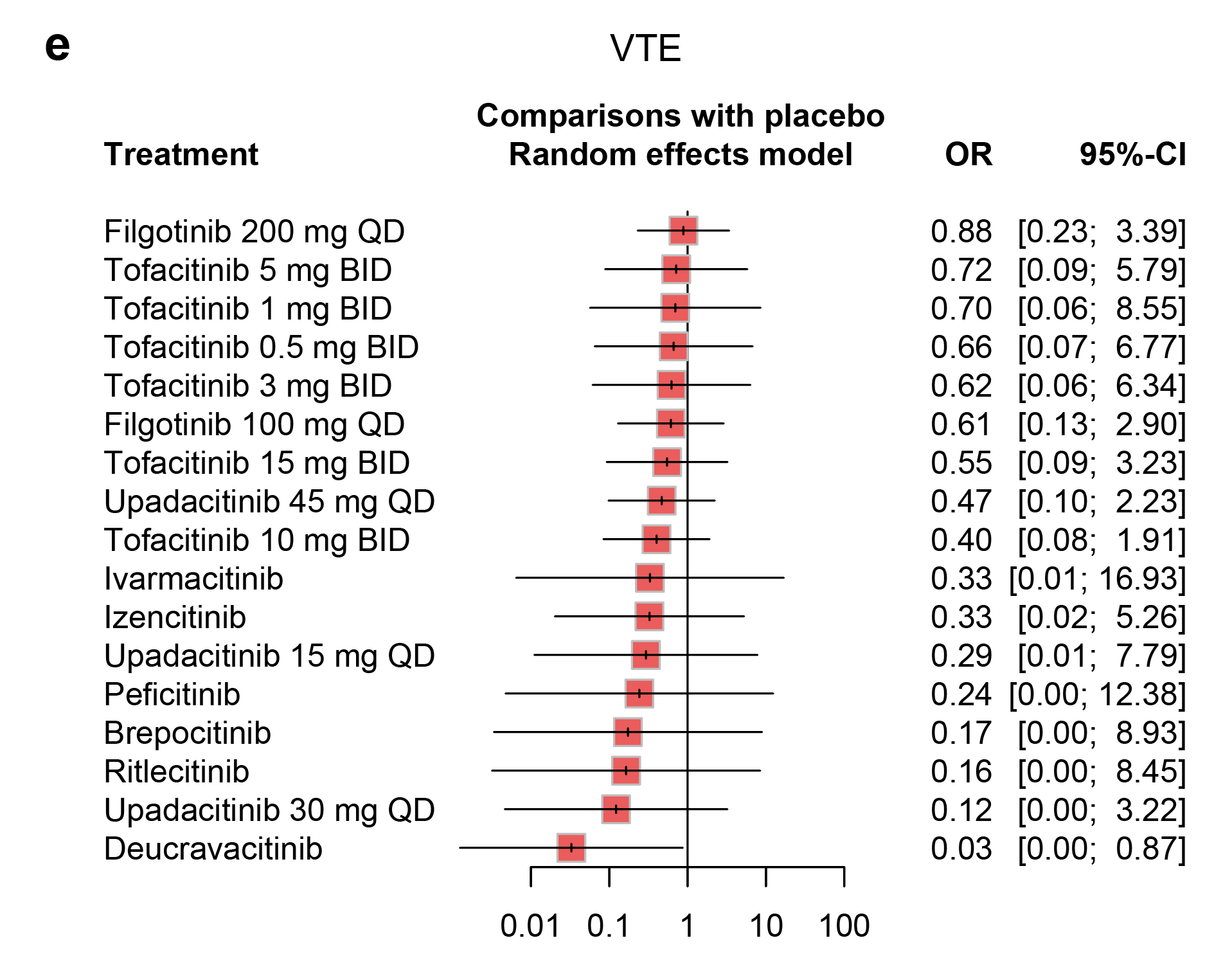


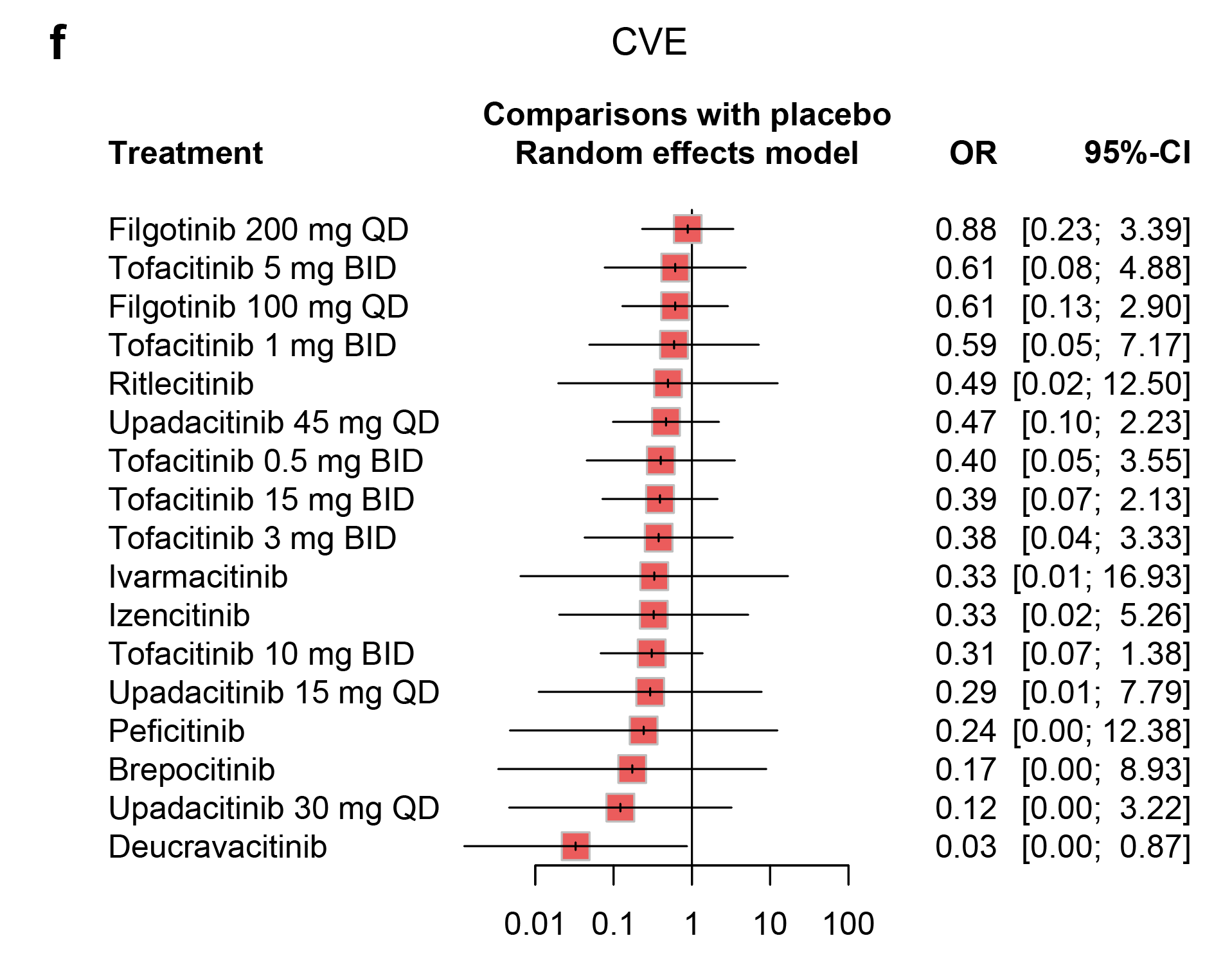


**Supplementary figure 21 Forest plots of the subgroup analysis (the maintenance** **phase) based on the study phase (without dose consideration) for MACE (a), VTE (b) and CVE (c), as well as (with dose consideration) for MACE (d), VTE (e) and CVE (f)**


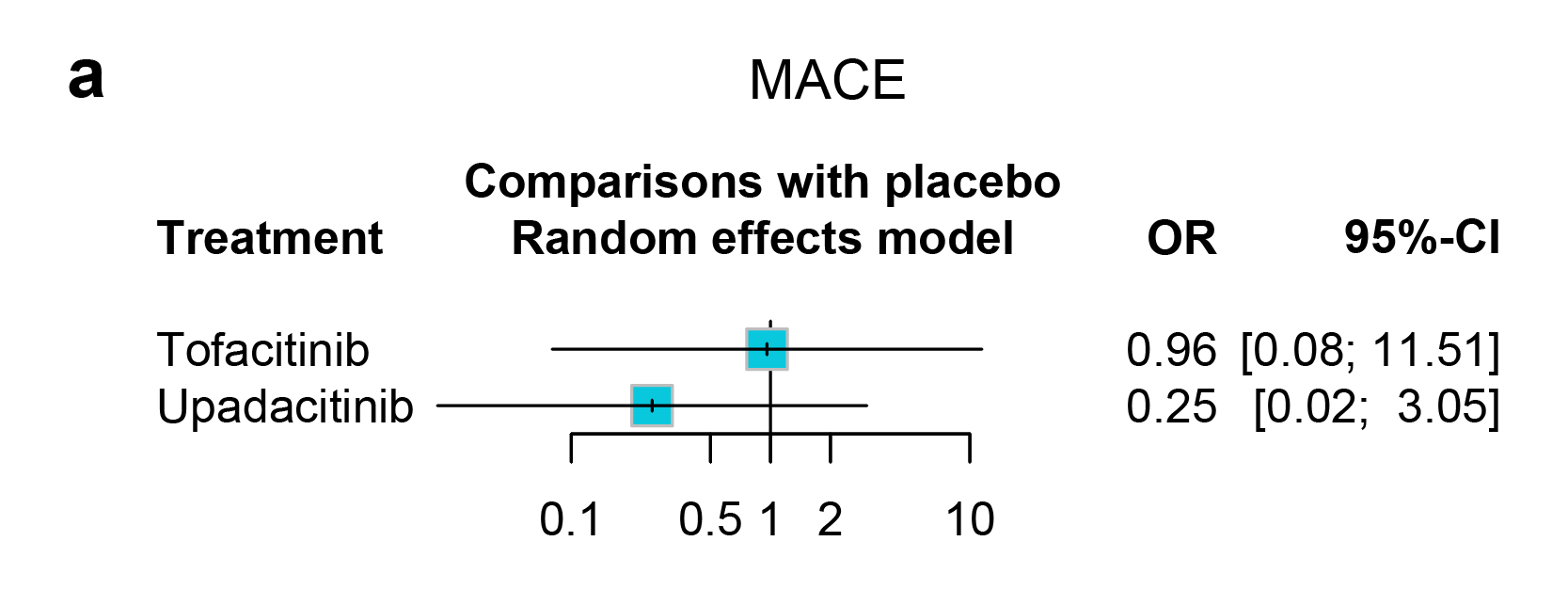


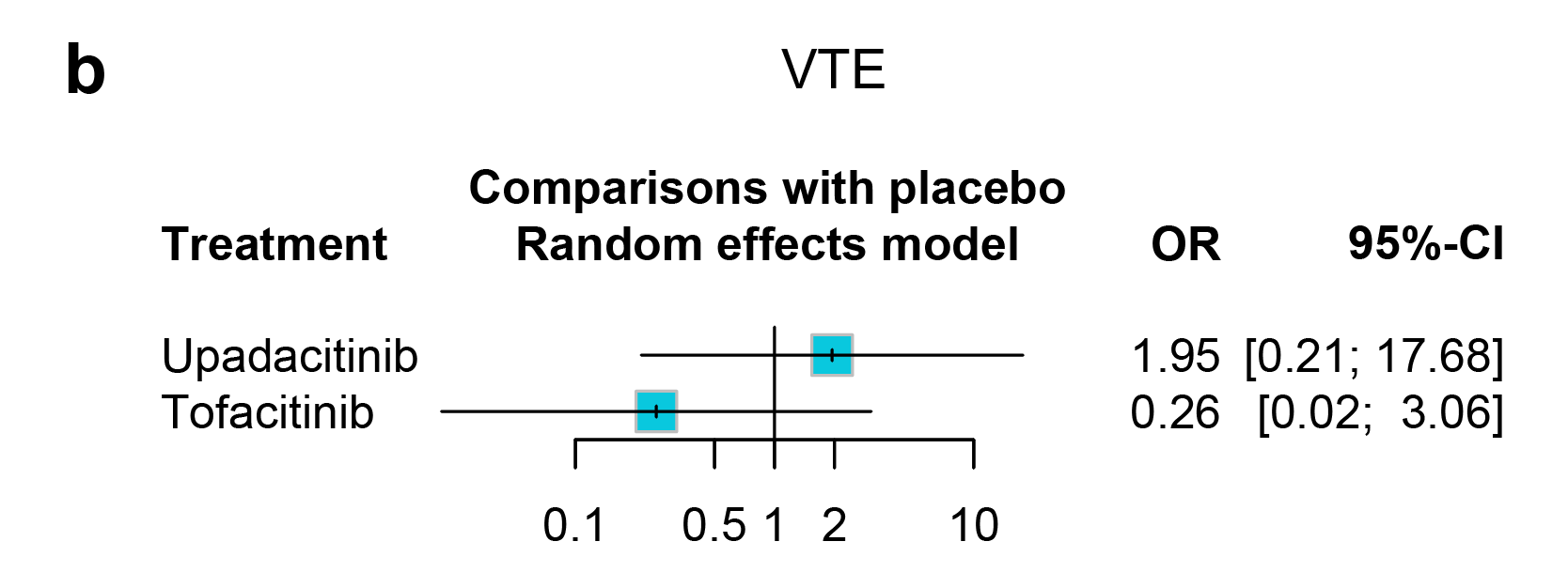


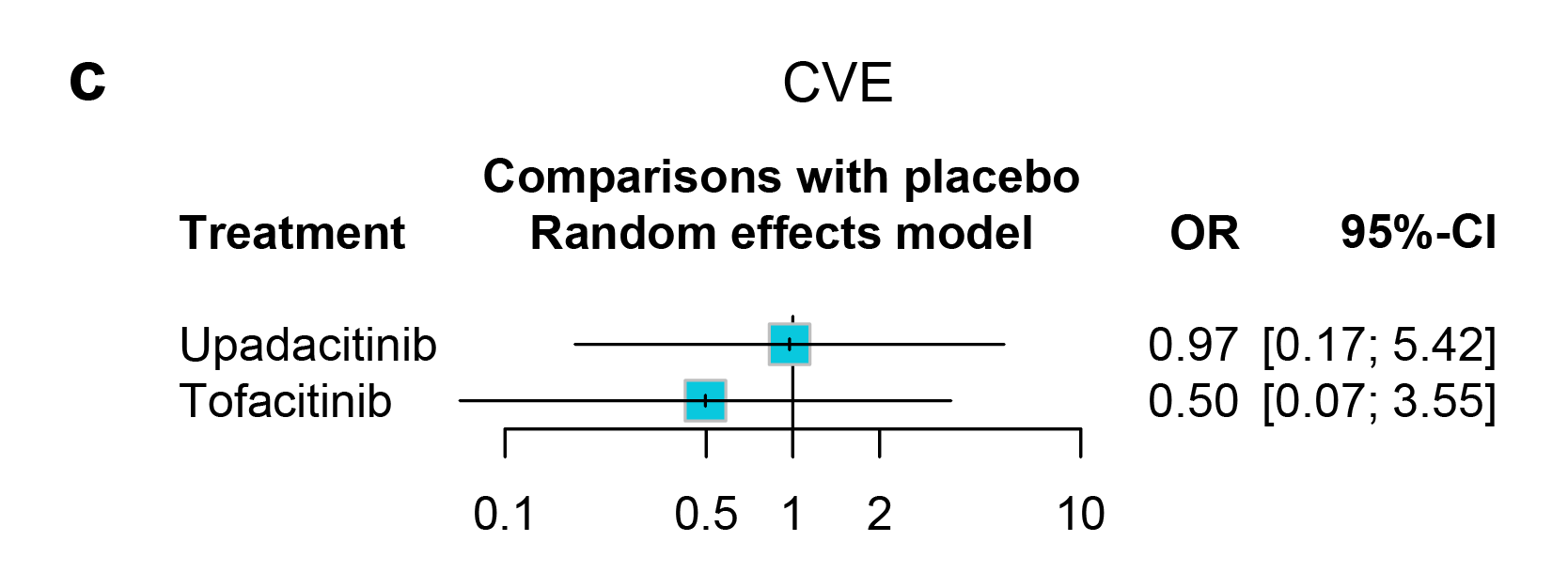


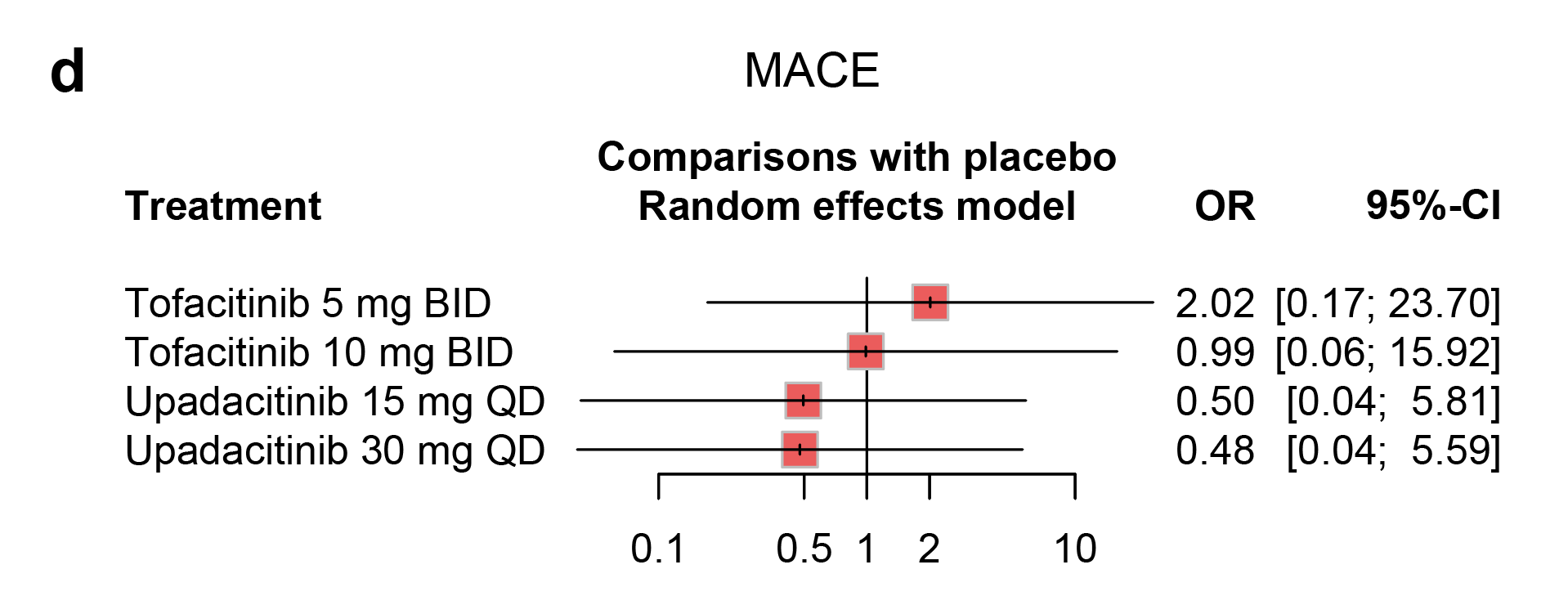


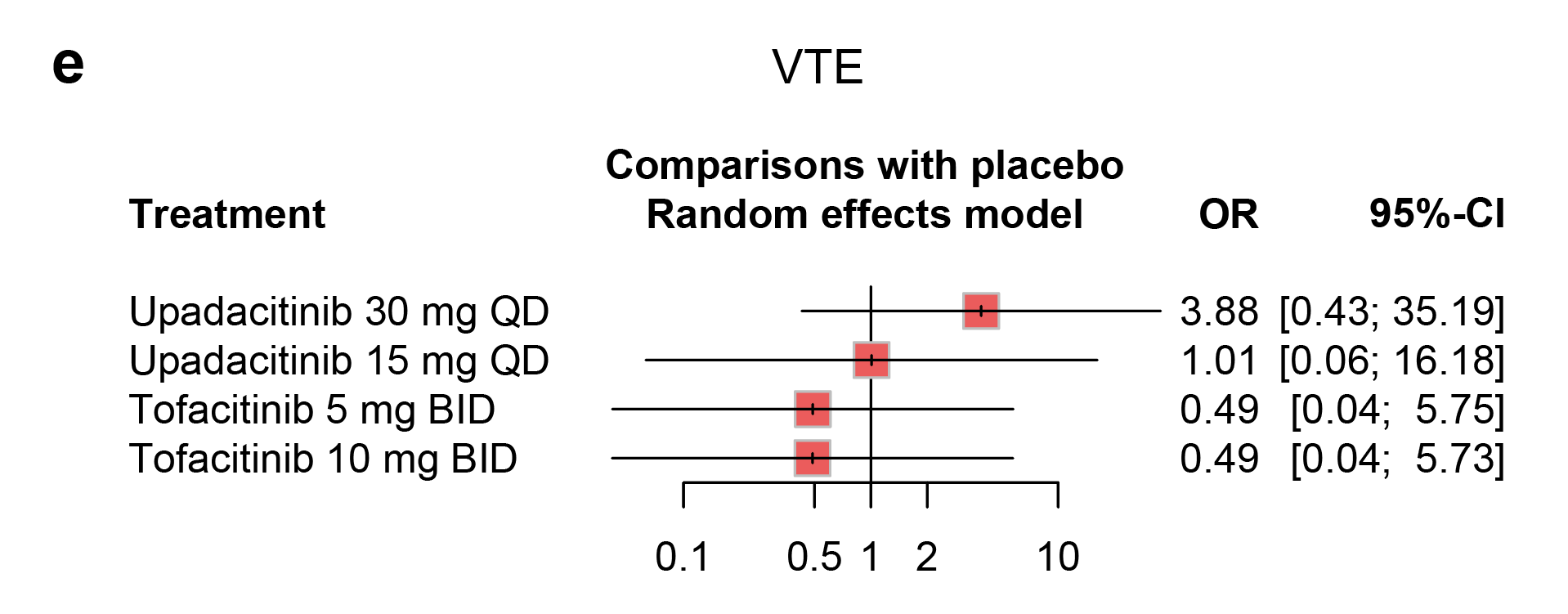


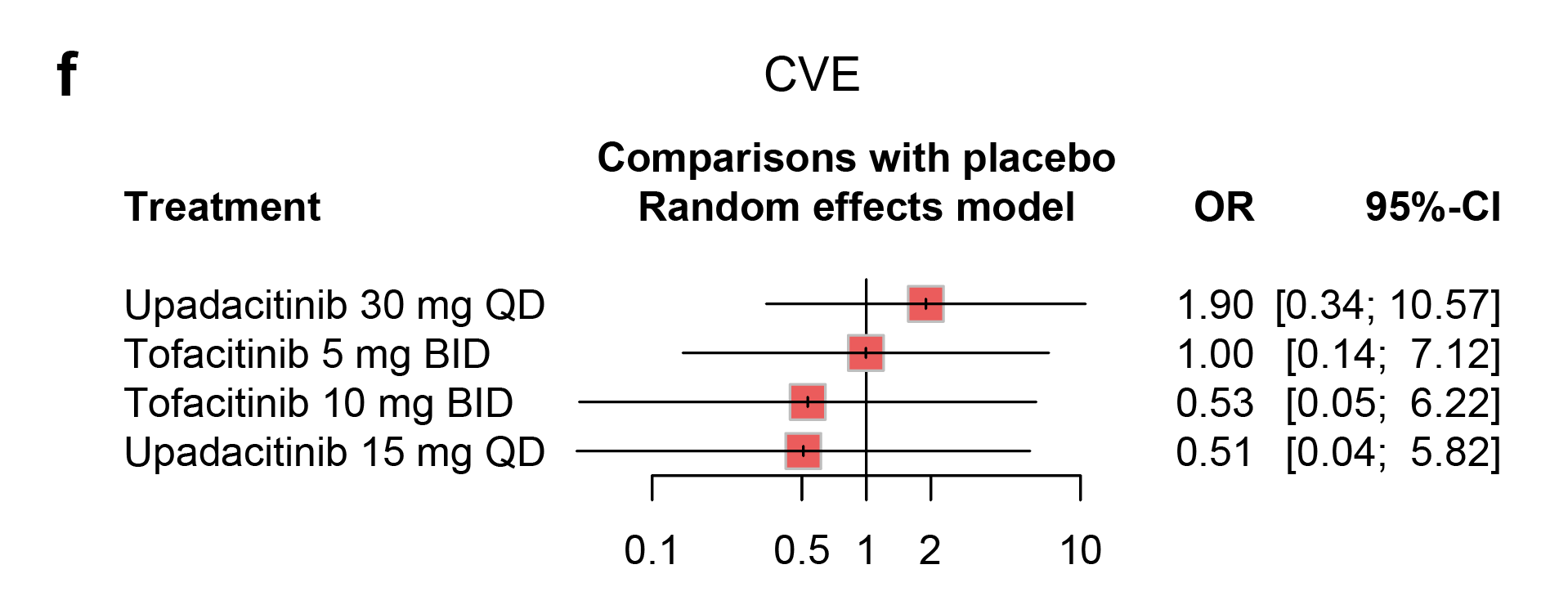

Supplement: Supplemental Material [file IANN_A_2455536_SM7544.zip › suppl_data/Supplementary figure.docx]
